# Supplementary material for: Lack of activity of recombinant HIF prolyl hydroxylases (PHDs) on reported non-HIF substrates
Source: eLife. 2019 Sep 10;8:e46490. doi: 10.7554/eLife.46490 (PMC6739866; doi:10.7554/eLife.46490)
Supplement: Table 1—source data 1. — Reported prolyl hydroxylation sites are indicated in red. [file elife-46490-table1-data1.docx]

**Table 1—source data 1**

| **Peptide Name** | **Sequence** |
| --- | --- |
| ACACB/438-462 | DEGLEAAERIGF**P**LMIKASEGGGGK |
| ACACB/445-462 | ERIGF**P**LMIKASEGGGGK |
| ACTB/295-319 | ANTVLSGGTTMY**P**GIADRMQKEITA |
| ACTB/310-334 | ADRMQKEITALA**P**STMKIKIIAPPE |
| ADRB2/370-394 | QEKENKLLCEDL**P**GTEDFVGHQGTV |
| ADRB2/383-407 | GTEDFVGHQGTV**P**SDNIDSQGRNCS |
| AKT1/113-137 | QEEEEMDFRSGS**P**SDNSGAEEMEVS |
| AKT1/115-134 | EEEMDFRSGS**P**SDNSGAEEM |
| AKT1/301-325 | KDGATMKTFCGT**P**EYLA**P**EVLEDND |
| AKT1/307-328 | KTFCGT**P**EYLA**P**EVLEDNDYGR |
| AKT1/311-330 | GT**P**EYLA**P**EVLEDNDYGRAV |
| ATF4/144-168 | LPESLTKPDQVA**P**FTFLQ**P**L**P**LS**P**G |
| ATF4/157-182 | TFLQ**P**L**P**LS**P**GVLSST**P**DHSFSLEL |
| CENPN/299-323_iso1 | SPHLLEALKSLA**P**AGIADAPLSPLL |
| CENPN/299-323_iso3 | SPHLLEALKSLA**P**AALVCRIQKLLC |
| CENPN/308-317_iso3 | SLA**P**AALVCR |
| CEP192/2303-2326 | DVKWHLSSLA**P**PYVKGVDESGDVF |
| CEP192/2306-2317 | WHLSSLA**P**PYVK |
| EEF2K/86-110 | KHAIQKAKHMPD**P**WAEFHLEDIATE |
| EPOR/434-458 | QLLRPWTLC**P**ELPPTP**P**HLKYLYLV |
| EPOR/441-455 | LC**P**ELPPTP**P**HLKYL |
| FLNA/P2310-2333 | VKFNEEHI**P**DSPFVV**P**VASPSGDAR |
| FOXO3/414-438 | FPYTTKGSGLGS**P**TSSFNSTVFG**P**S |
| FOXO3/425-448 | S**P**TSSFNSTVFG**P**SSLNSLRQSPMQ |
| HIF1A/556-574 | DLDLEMLA**P**YIPMDDDFQL |
| IKBKB/180-204 | TSFVGTLQYLA**P**ELLEQQKYTVTVD |
| MAPK6/13-37 | GFDLGSRYMDLK**P**LGCGGNGLVFSA |
| NDRG3/282-306 | TTLLKMADCGGL**P**QVVQPGKLTEAF |
| PDE4D/17-41 | GSDSAGGATLKA**P**KHLWRHEQHHQY |
| PDE4D/370-394 | LMHSSSLTNSSI**P**RFGVKTEQEDVL |
| PDE4D/407-431 | HVFRIAELSGNR**P**LTVIMHTIFQER |
| PKM/391-415 | HLQLFEELRRLA**P**ITSD**P**TEATAVG |
| PKM/396-422 | EELRRLA**P**ITSD**P**TEATAVGAVEASFK |
| PPP2R2A/308-332 | VKIWDLNMENR**P**VETYQVHEYLRSK |
| PPP2R2A/311-329 | WDLNMENR**P**VETYQVHEYL |
| POLR2A/1456-1476 | ENIMLGQLA**P**AGTGCFDLLLD |
| SPRY2/6-30 | QSGNGSQPLLQT**P**RDGGRQRGEPDP |
| SPRY2/132-156 | EQRLLGSSFSSG**P**VADGIIRVQPKS |
| SPRY2/148-172 | GIIRVQPKSELK**P**GELKPLSKEDLG |
| TELO2/362-386 | KAVLICLAQLGE**P**ELRDSRDELLAS |
| TELO2/409-433 | VAEVVSARIH**P**EG**P**PLKFQYEEDEL |
| THRA/149-173 | EMIRSLQQRPE**P**T**P**EEWDLIHIATE |
| TP53/347-371 | ALELKDAQAGKE**P**GGSRAHSSHLKS |
| TRPA1/382-406 | TVQQPYGLKNLR**P**EFMQMQQIKELV |
| TRPA1/386-405 | PYGLKNLR**P**EFMQMQQIKEL |
