# Supplementary material for: Lack of activity of recombinant HIF prolyl hydroxylases (PHDs) on reported non-HIF substrates
Source: eLife. 2019 Sep 10;8:e46490. doi: 10.7554/eLife.46490 (PMC6739866; doi:10.7554/eLife.46490)
Supplement: Figure 5—source data 1. [file elife-46490-fig5-data1.docx]

1. PHD1 hydroxylation assays

| Substrate | 3H-Hyp DPM / 1 x 10^6^ 3H-Pro DPM | |
| --- | --- | --- |
|  | Exp 1 | Exp 2 |
| WT HIF1A | 13 795 | 13 437 |
| PP/AG HIF1A | -196 | 250 |
| SPRY2 | -437 | 497 |
| IKBKB | 418 | 183 |
| TP53 | 545 | 202 |
| CEP192 | 353 | 356 |
| FOXO3 | 136 | 226 |

1. PHD2 hydroxylation assays

| Substrate | 3H-Hyp DPM / 1 x 10^6^ 3H-Pro DPM | |
| --- | --- | --- |
|  | Exp 1 | Exp 2 |
| WT HIF1A | 12 902 | 11 433 |
| PP/AG HIF1A | 213 | -16 |
| PDE4D | -234 | 60 |
| FLNA | 70 | 103 |
| EEF2K | -184 | 252 |
| CENPN | 410 | -122 |
| TRPA1 | 338 | - |
| THRA | 396 | -128 |
| NDRG3 | -159 | -39 |
| AKT1 | 238 | 49 |

1. PHD3 hydroxylation assays

| Substrate | 3H-Hyp DPM / 1 x 10^6^ 3H-Pro DPM | |
| --- | --- | --- |
|  | Exp 1 | Exp 2 |
| WT HIF1A | 8 969 | 3 667 |
| PP/AG HIF1A | 128 | 173 |
| EPOR | 140 | -11 |
| MAPK6 | 415 | -120 |
| SPRY2 | 186 | -552 |
| ATF4 | 286 | 198 |
| THRA | 335 | 85 |
| TP53 | 210 | 25 |
| ACACB | 78 | 526 |
| PKM | 96 | - |
| TELO2 | -368 | - |
| ADRB2 | 144 | -119 |
| ACTB | 188 | -210 |
