# Supplementary material for: Lack of activity of recombinant HIF prolyl hydroxylases (PHDs) on reported non-HIF substrates
Source: eLife. 2019 Sep 10;8:e46490. doi: 10.7554/eLife.46490 (PMC6739866; doi:10.7554/eLife.46490)
Supplement: Table 3—source data 1. — MSMS assignment frequency of artefactual oxidations observed on unmodified tryptic peptide standards; oxidations are stratified by residue (e.g., Met, Pro, other). Reported assignments (column 4) were not subject to PTM localisation (AScore) filtering. Oxidations assigned to target Pro residues are indicated in red. Variation in the total number of peptide assignments (Column 5) reflects differences in the amount of peptide injected and/or replicate runs. [file elife-46490-table3-data1.docx]

**Table 3—source data 1**

| **Peptide** | **Sequence** | **Variant** | **# Spectra assigned** | **# Total** |
| --- | --- | --- | --- | --- |
| ACACB/341-361 | RIPVQAVWAGWGHASENPKLPELLCK | Unox | 8 | 10 |
|  |  | Other | 2 |  |
| ACACB/436-454 | DVDEGLEAAERIGFPLMIK | Unox | 93 | 137 |
|  |  | P450ox | 3 |  |
|  |  | M452ox | 40 |  |
|  |  | Other | 1 |  |
| ACTB/292-312 | DLYANTVLSGGTTMYPGIADR | Unox | 176 | 257 |
|  |  | M305ox | 62 |  |
|  |  | Other | 19 |  |
| ACTB/316-326 | EITALAPSTMK | Unox | 17 | 36 |
|  |  | P322ox | 5 |  |
|  |  | M325ox | 8 |  |
|  |  | Other | 6 |  |
| ADRB2/376-404 | LLCEDLPGTEDFVGHQGTVPSDNIDSQGR | Unox | 43 | 48 |
|  |  | Other | 5 |  |
| AKT1/122-140 | SGSPSDNSGAEEMEVSLAK | Unox | 68 | 92 |
|  |  | M134ox | 23 |  |
|  |  | Other | 1 |  |
| AKT1/308-328 | TFCGTPEYLAPEVLEDNDYGR | Unox | 107 | 117 |
|  |  | P313ox | 6 |  |
|  |  | Other | 4 |  |
| AKT1/421-436 | LSPPFKPQVTSETDTR | Unox | 38 | 38 |
| CENPN/308-329 | SLAPAGIADAPLSPLLTCIPNKR | Unox | 19 | 21 |
|  |  | Other | 2 |  |
| CEP192/2306-2317 | WHLSSLAPPYVK | Unox | 107 | 108 |
|  |  | Other | 1 |  |
| EEF2K/94-111 | HMPDPWAEFHLEDIATER | Unox | 168 | 181 |
|  |  | M95ox | 9 |  |
|  |  | Other: P96ox | 2 |  |
|  |  | Other | 2 |  |
| FLNA/2311-2333 | FNEEHIPDSPFVVPVASPSGDAR | Unox | 269 | 276 |
|  |  | P2317ox; Other | 2 |  |
|  |  | P2324ox; Other | 1 |  |
|  |  | Other | 4 |  |
| FOXO3/420-444 | GSGLGSPTSSFNSTVFGPSSLNSLR | Unox | 42 | 44 |
|  |  | P426ox | 2 |  |
| IKBKB/172-198 | ELDQGSLCTSFVGTLQYLAPELLEQQK | Unox | 7 | 7 |
| MAPK6/20-45 | YMDLKPLGCGGNGLVFSAVDNDCDKR | Unox | 36 | 56 |
|  |  | P25ox | 3 |  |
|  |  | M21ox | 12 |  |
|  |  | Other | 5 |  |
| NDRG3/287-301 | MADCGGLPQVVQPGK | Unox | 19 | 30 |
|  |  | M287ox | 10 |  |
|  |  | Other | 1 |  |
| PDE4D/370-383 | LMHSSSLTNSSIPR | Unox | 64 | 93 |
|  |  | M371ox | 29 |  |
| PDE4D/411-431 | IAELSGNRPLTVIMHTIFQER | Unox | 10 | 13 |
|  |  | M424ox | 2 |  |
|  |  | Other | 1 |  |
| PKM/401-422 | LAPITSDPTEATAVGAVEASFK | Unox | 22 | 24 |
|  |  | P403ox; Other | 2 |  |
| PPP2R2A/310-330 | IWDLNMENRPVETYQVHEYLR | Unox | 26 | 33 |
|  |  | M315ox | 2 |  |
|  |  | Other | 5 |  |
| SPRY2/5-19 | AQSGNGSQPLLQTPR | Unox | 45 | 46 |
|  |  | Other | 1 |  |
| SPRY2/135-151 | LLGSSFSSGPVADGIIR | Unox | 14 | 15 |
|  |  | Other | 1 |  |
| SPRY2/156-168 | SELKPGELKPLSK | Unox | 134 | 134 |
| TELO2/363-377 | AVLICLAQLGEPELR | Unox | 18 | 19 |
|  |  | P374ox | 1 |  |
| THRA/153-176 | SLQQRPEPTPEEWDLIHIATEAHR | Unox | 12 | 13 |
|  |  | Other (diox) | 1 |  |
| TP53/358-370 | EPGGSRAHSSHLK | Unox | 88 | 88 |
| TRPA1/391-403 | NLRPEFMQMQQIK | Unox | 147 | 178 |
|  |  | M397ox/M399ox | 30 |  |
|  |  | Other | 1 |  |
