# Supplementary material for: Lack of activity of recombinant HIF prolyl hydroxylases (PHDs) on reported non-HIF substrates
Source: eLife. 2019 Sep 10;8:e46490. doi: 10.7554/eLife.46490 (PMC6739866; doi:10.7554/eLife.46490)
Supplement: Supplementary file 1. [file elife-46490-supp1.pdf]

Supplementary file 1

A-001: HIF1A$_{(548-575)}$: NPFSTQDTDLDLEMLAPYIPMDDDFQLR

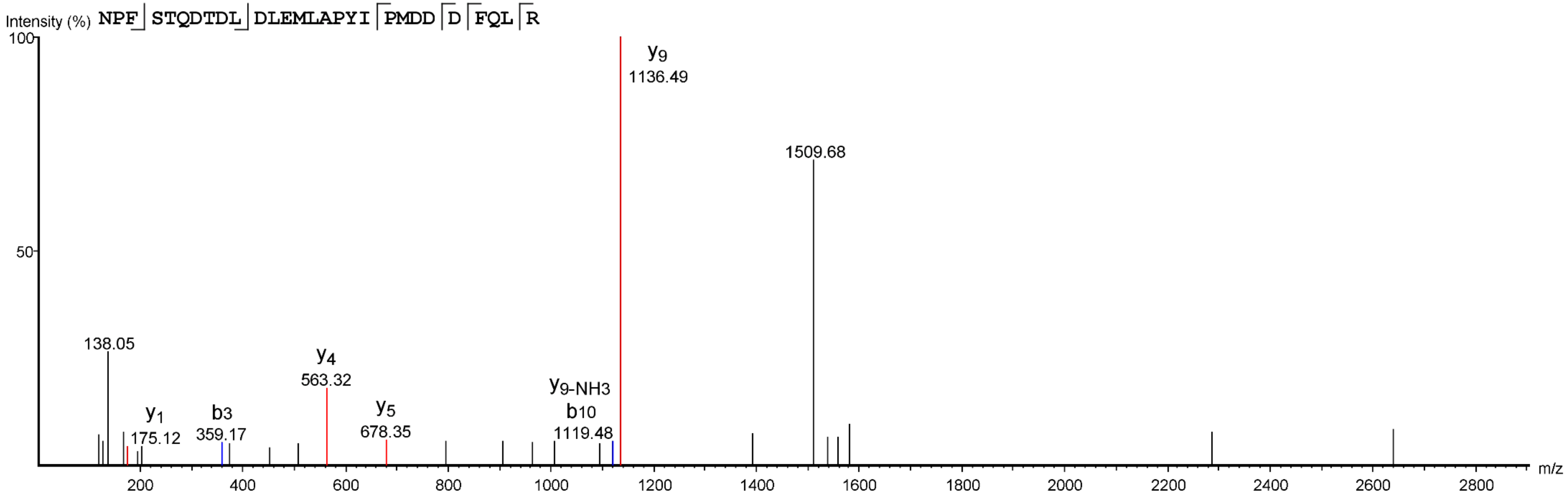

YMT72
Scan #26938

A-002: HIF1A$_{(548-575)}$: NPFSTQDTDLDLEM(+15.99)LAPYIPMDDDFQLR

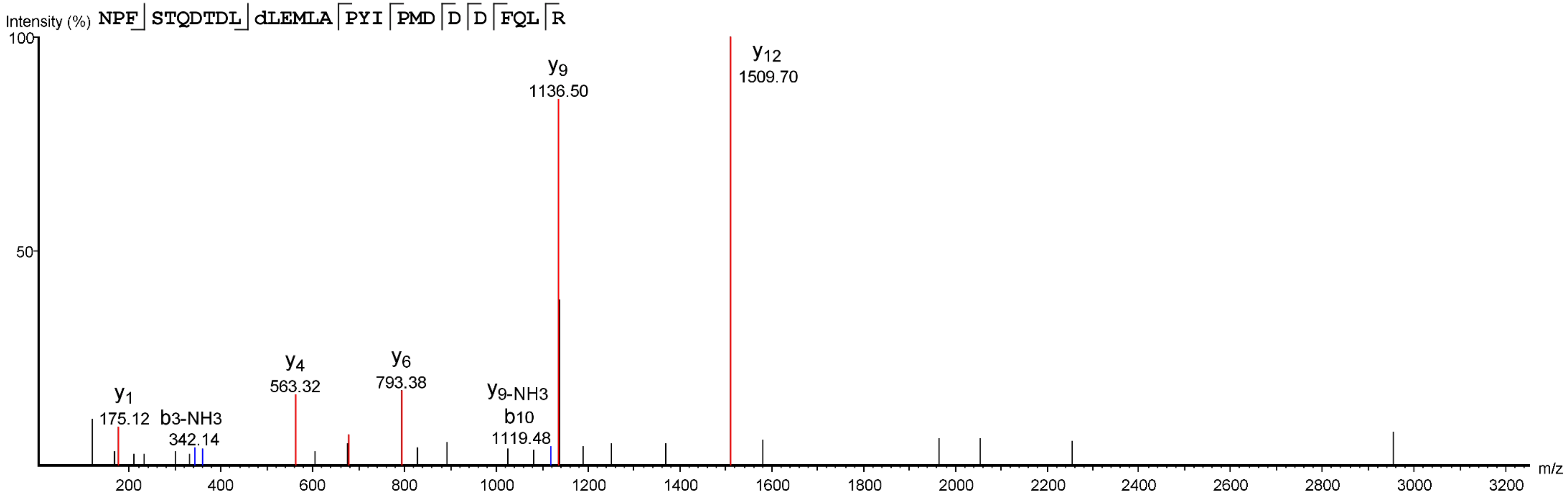

YMT71

Scan #25218

A-003: HIF1A$_{(548-575)}$: NPFSTQDTDLDLEM(+15.99)LAPYIPMDDDFQLR

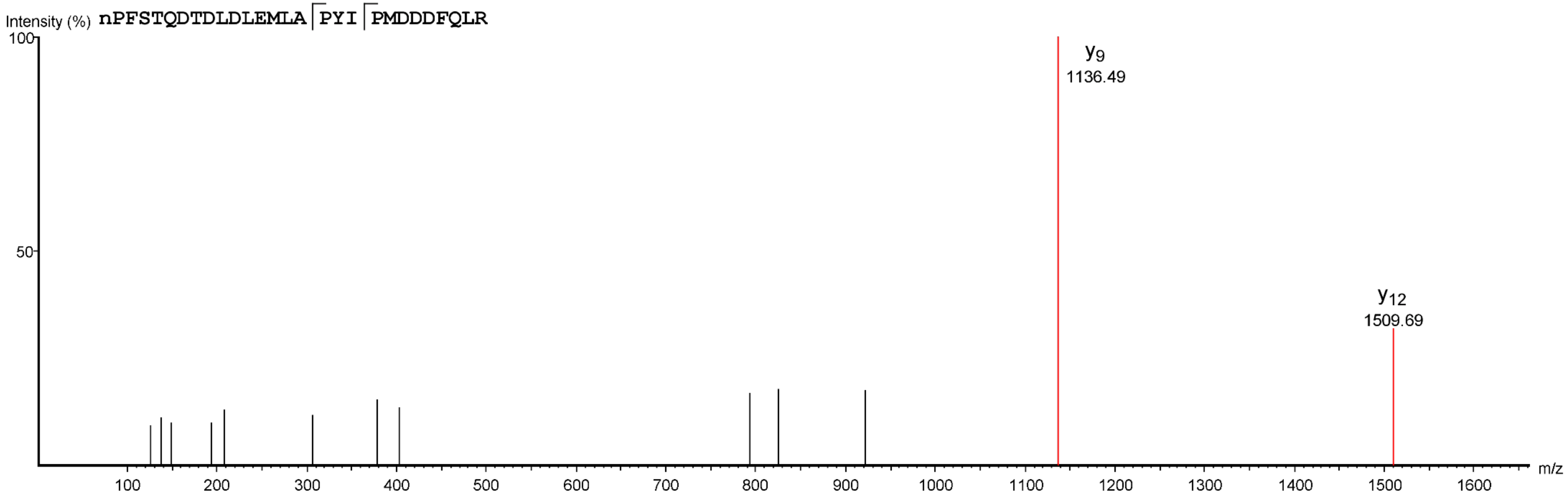

YMT72

Scan #25965

A-004: HIF1A$_{(548-575)}$: NPFSTQDTDLDLEMLAP(+15.99)YIPMDDDFQLR

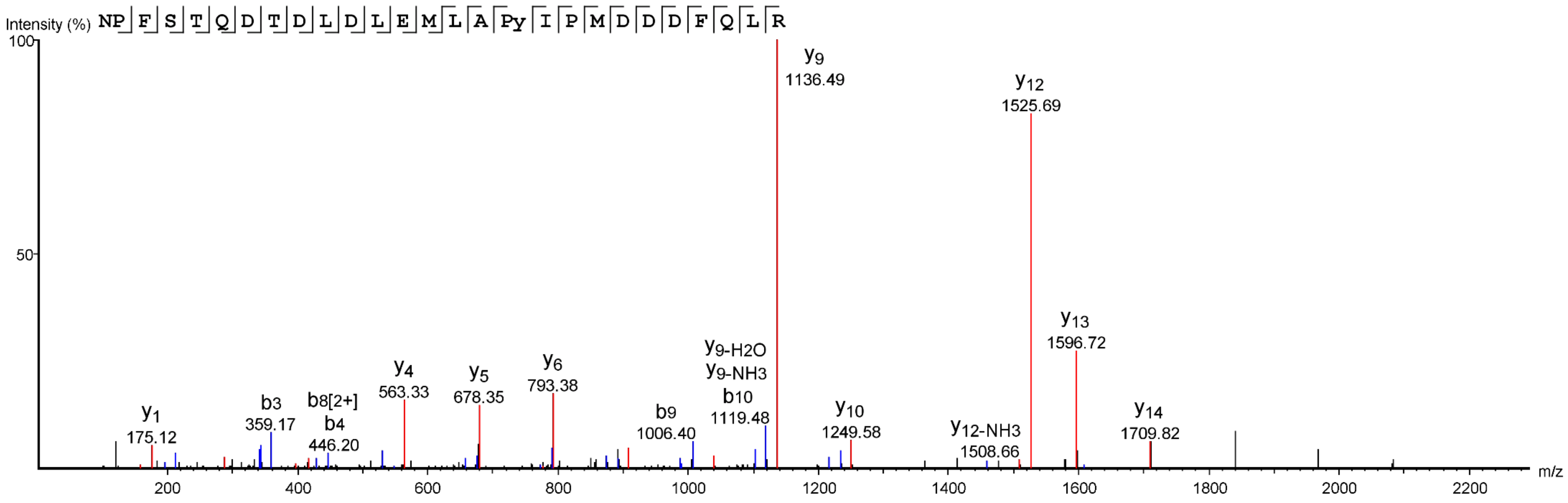

YMT72
Scan #26918

A-005: HIF1A(548-575): NPFSTQDTDLDLEMLAPYIPM(+15.99)DDDFQLR

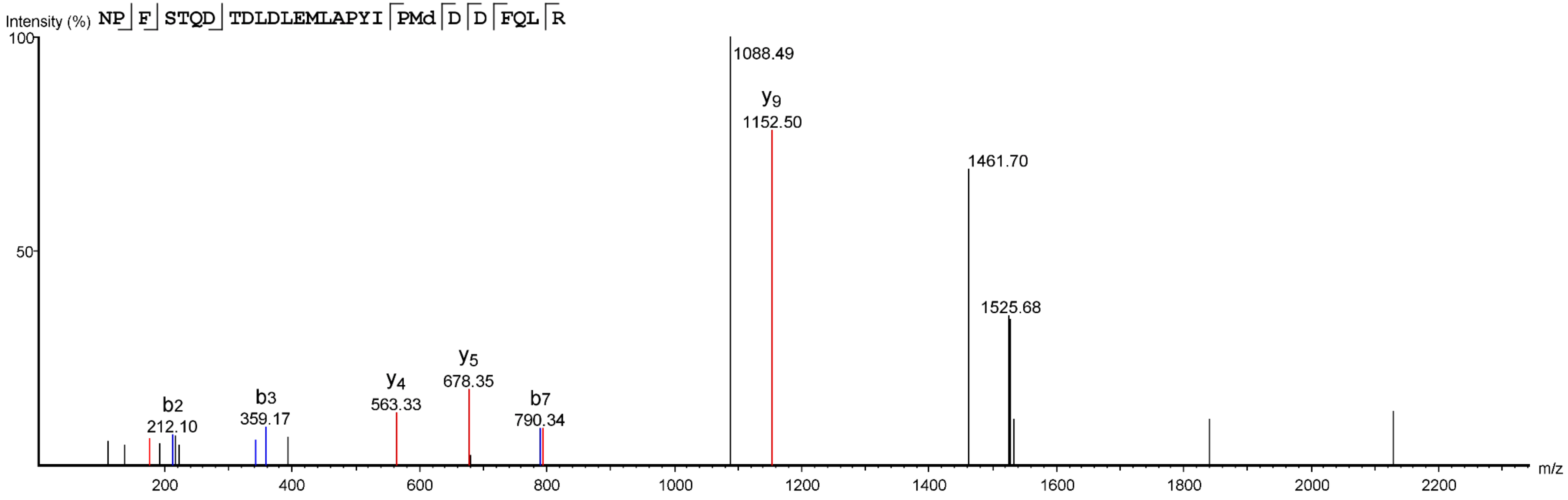

YMT71
Scan #25577

A-006: HIF1A$_{(548-575)}$: NPFSTQDTDLDLEM(+15.99)LAP(+15.99)YIPMDDDFQLR

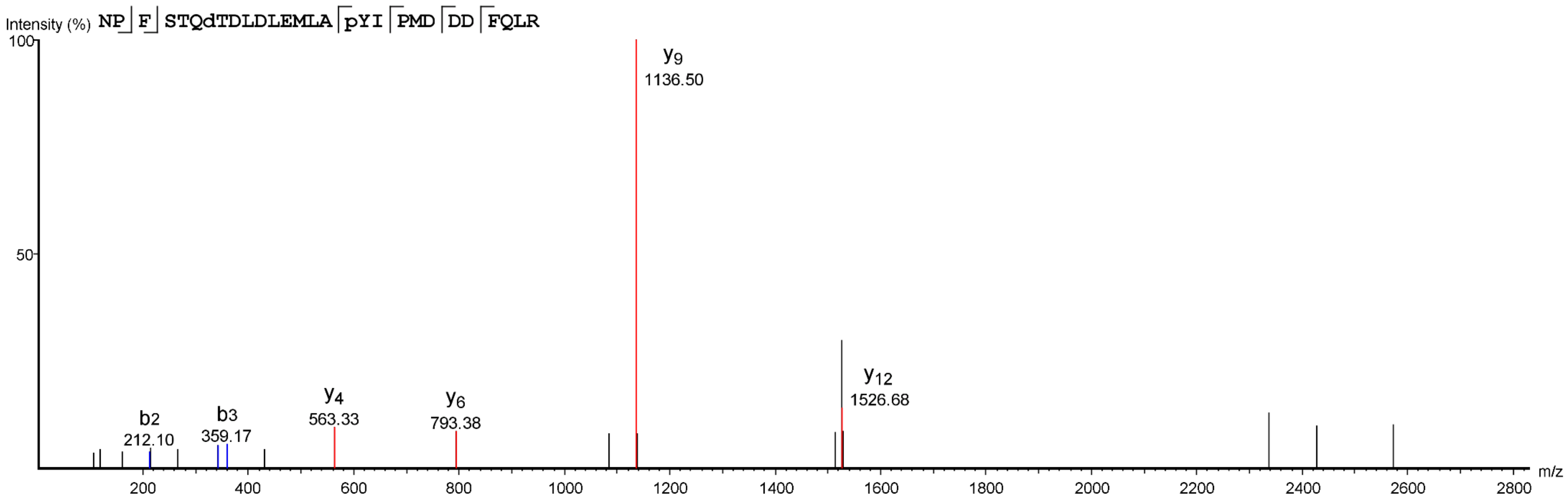

YMT71
Scan #24967

A-007: HIF1A(548-575): NPFSTQDTDLDLEM(+15.99)LAP(+15.99)YIPMDDDFQLR

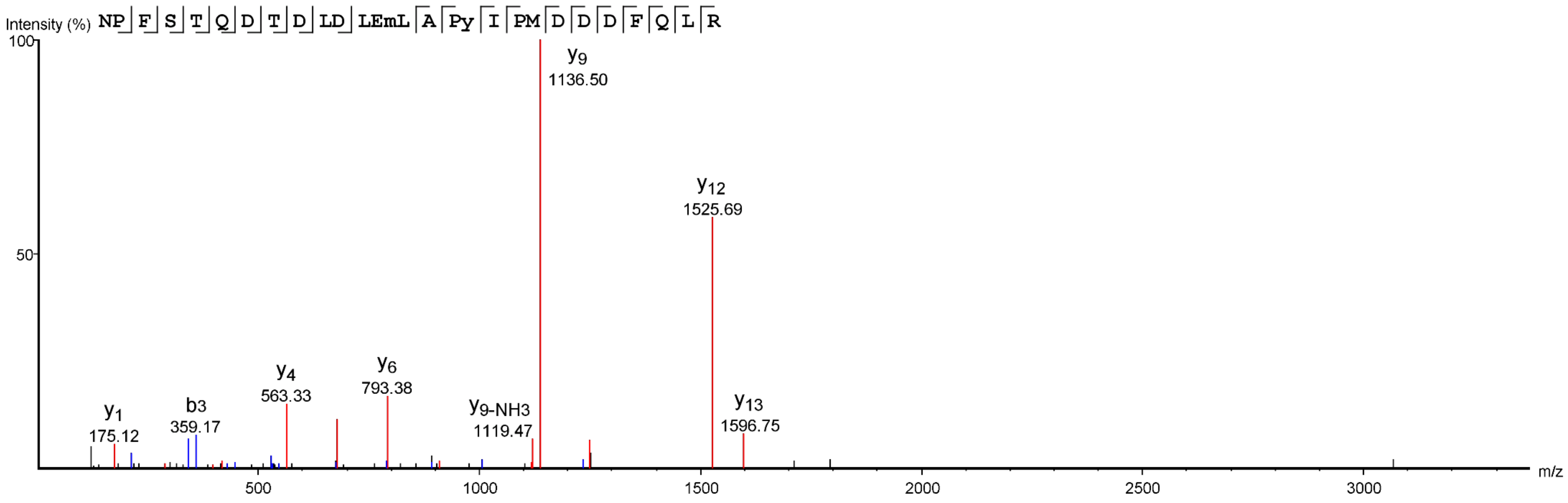

YMT72
Scan #25828

A-008: HIF1A$_{(548-575)}$: NPFSTQDTDLDLEM(+15.99)LAPYIPM(+15.99)DDDFQLR

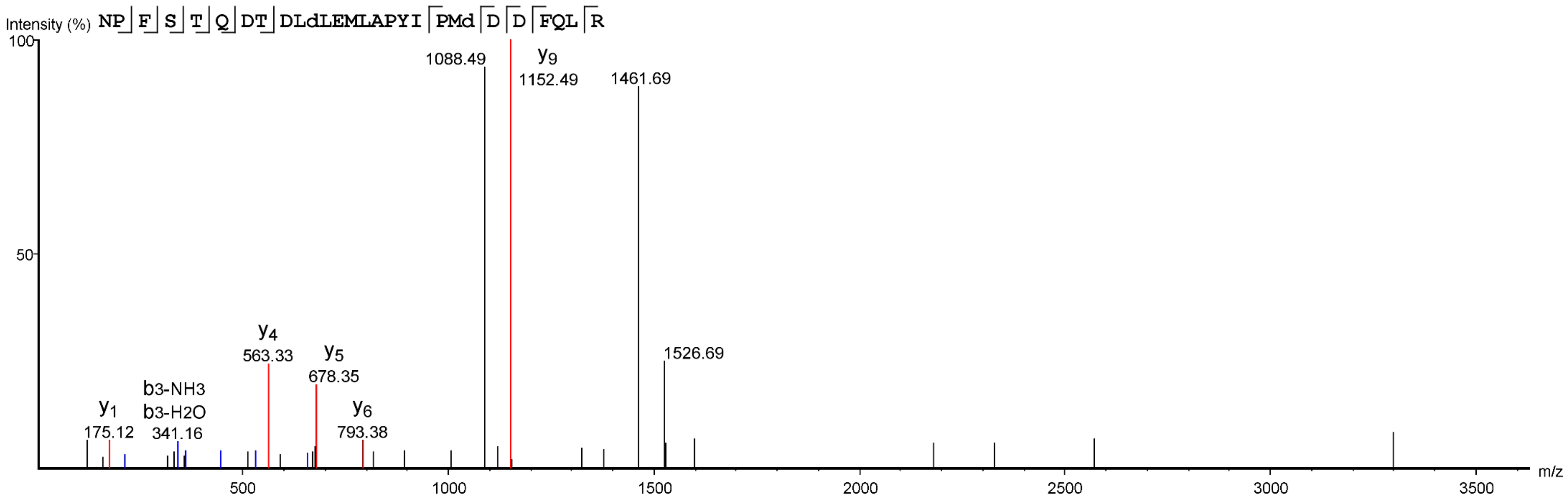

YMT71
Scan #23889

A-009: HIF1A(548-575): NPFSTQDTDLDLEMLAP(+15.99)YIPM(+15.99)DDDFQLR

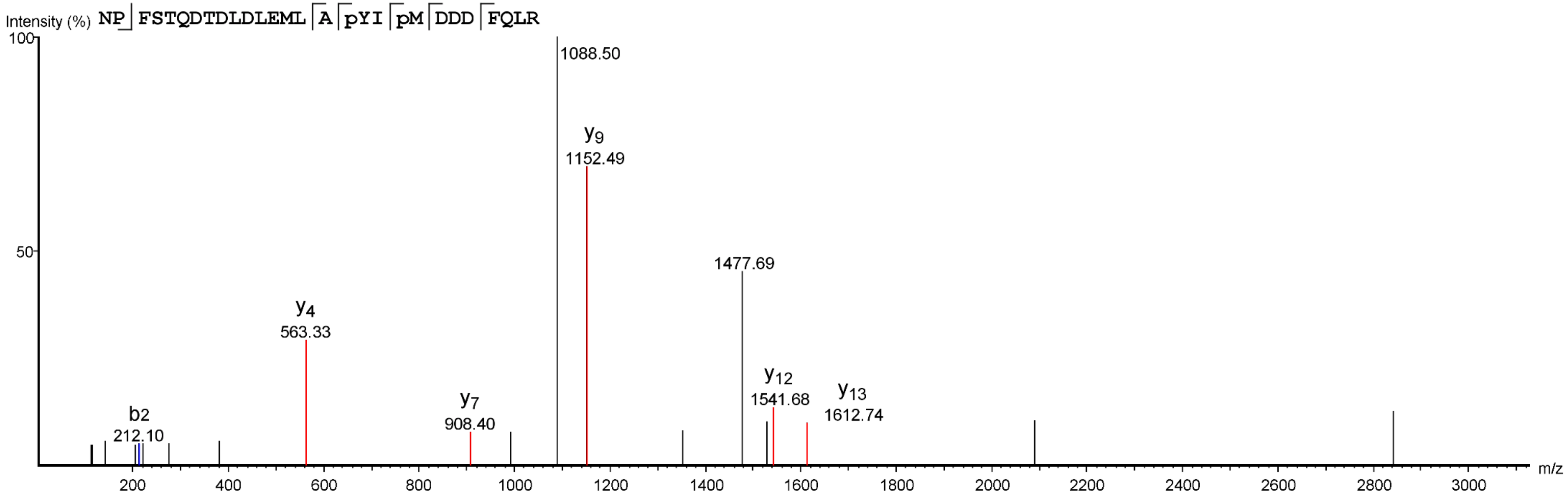

YMT71
Scan #25535

A-010: HIF1A(548-575): NPFSTQDTDLDLEMLAP(+15.99)YIPM(+15.99)DDDFQLR

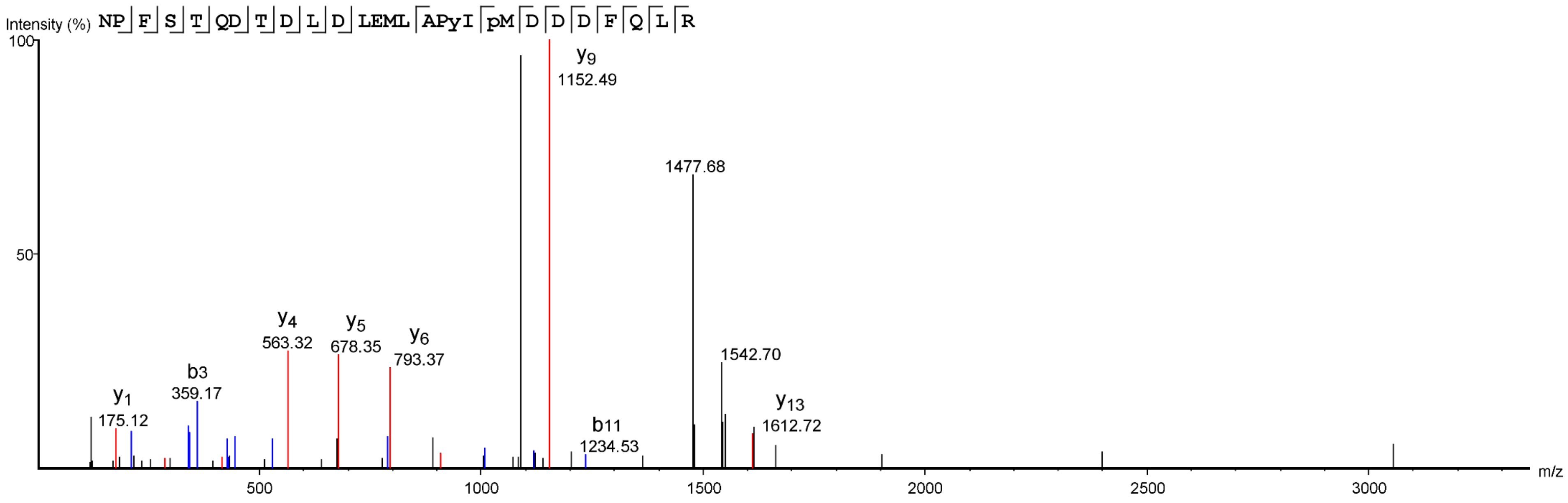

YMT72
Scan #26435

A-011: HIF1A(548-575): NPFSTQDTDLDLEM(+15.99)LAP(+15.99)YIPM(+15.99)DDDFQLR

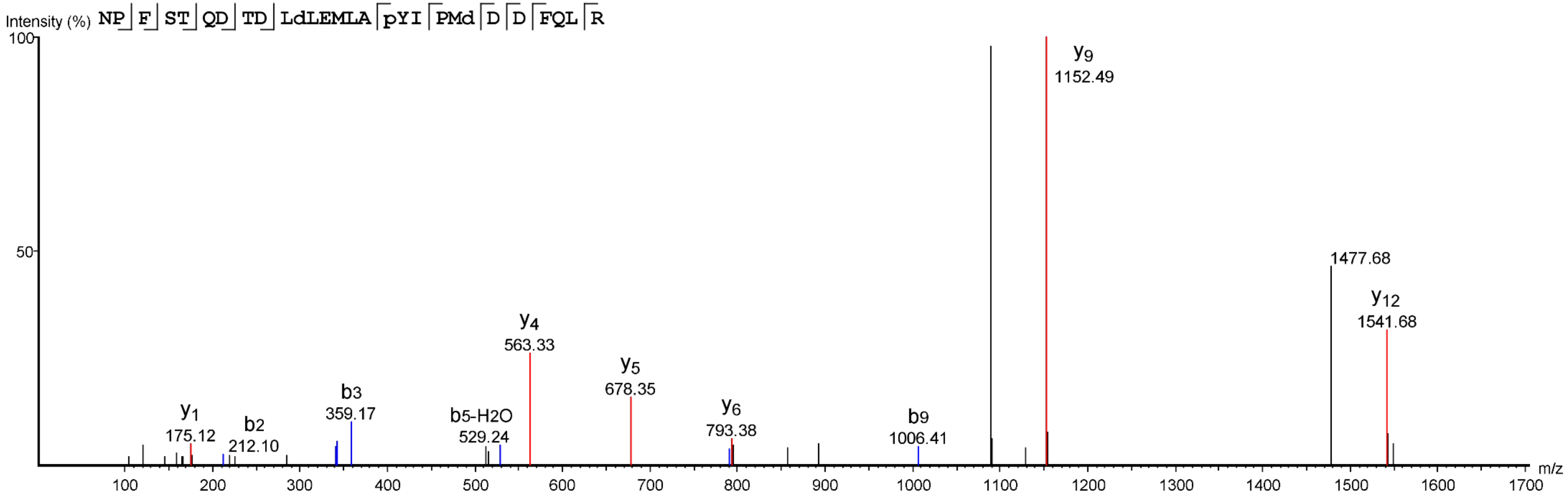

YMT71
Scan #23778

A-012: HIF1A$_{(548-575)}$: NPFSTQDTDLDLEM(+15.99)LAP(+15.99)YIPM(+15.99)DDDFQLR

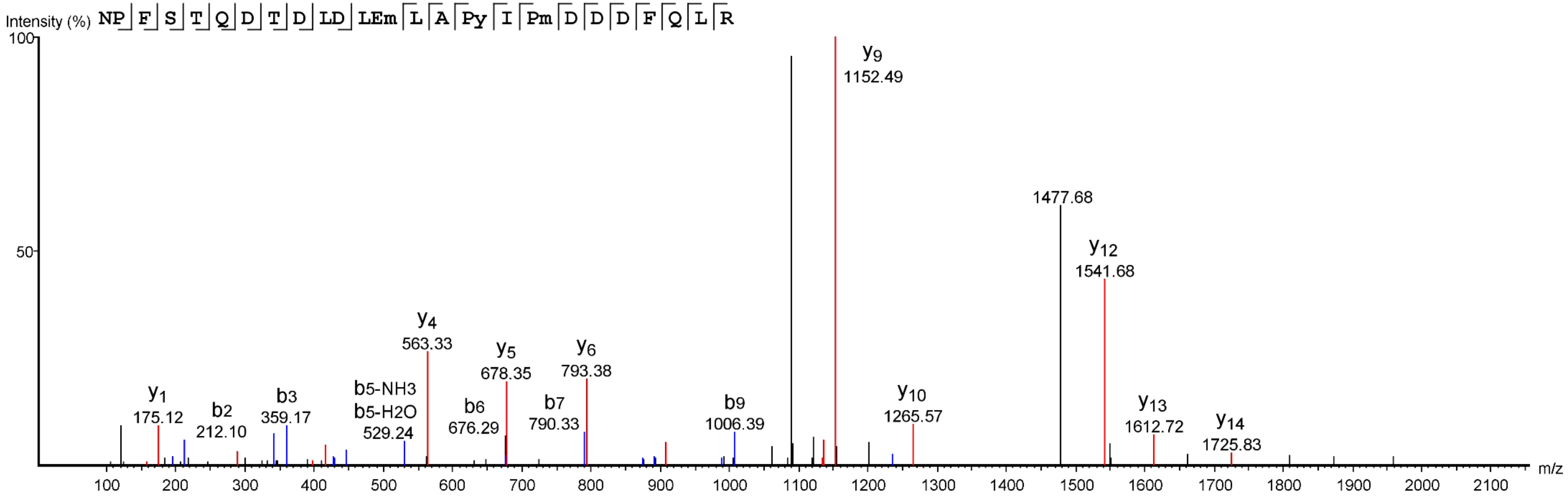

YMT72
Scan #24531

A-013: HIF1A*$_{(548-575)}$: NPFSTQDTDLDLEALAPYIPADDDFQLR

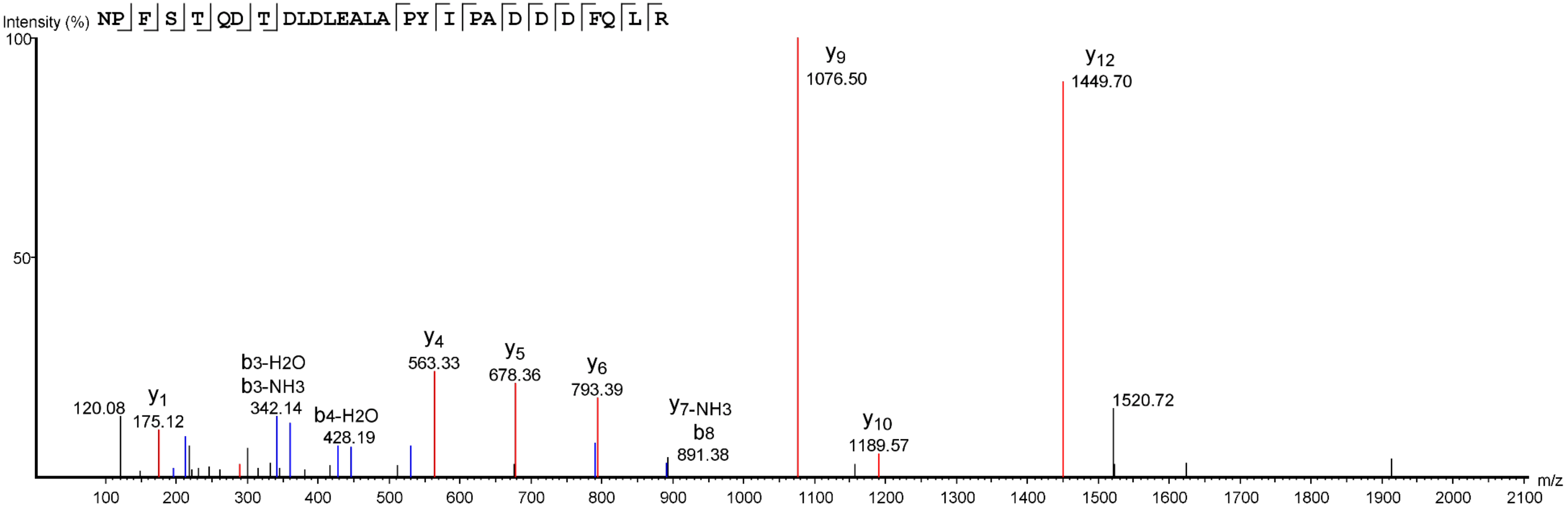

YMT57

Scan #25762

A-014: HIF1A*(548-575): NPFSTQDTDLDLEALAPYIPADDDFQLR

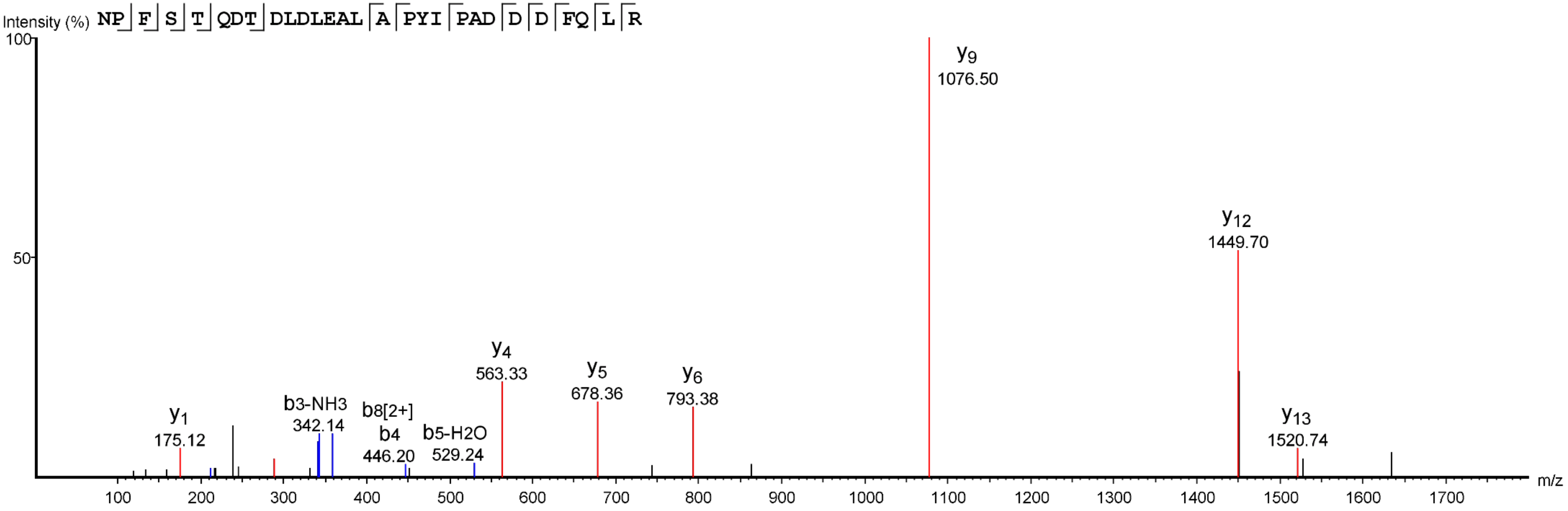

YMT58
Scan #27179

A-015: HIF1A*$_{(548-575)}$: NPFSTQDTDLDLEALAP(+15.99)YIPADDDFQLR

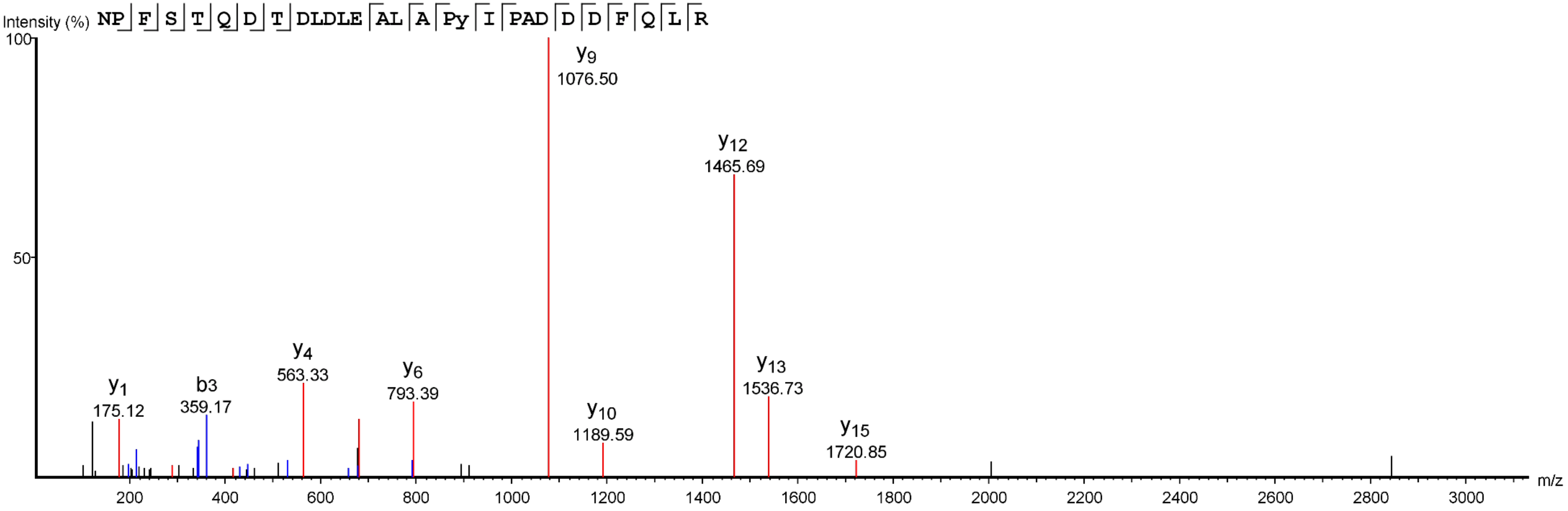

YMT57
Scan #25747

A-016: HIF1A*(548-575): NPFSTQDTDLDLEALAP(+15.99)YIPADDDFQLR

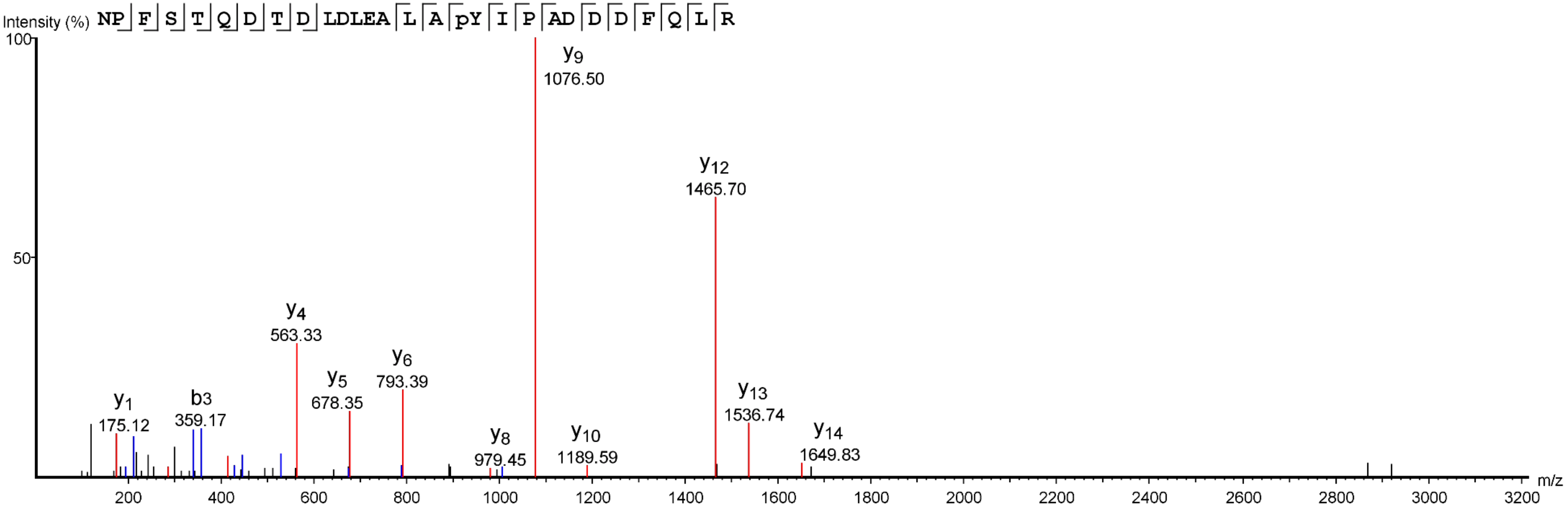

YMT58
Scan #27163

A-017: HIF1A*$_{(392-403)}$: KEPDALTLLAPA

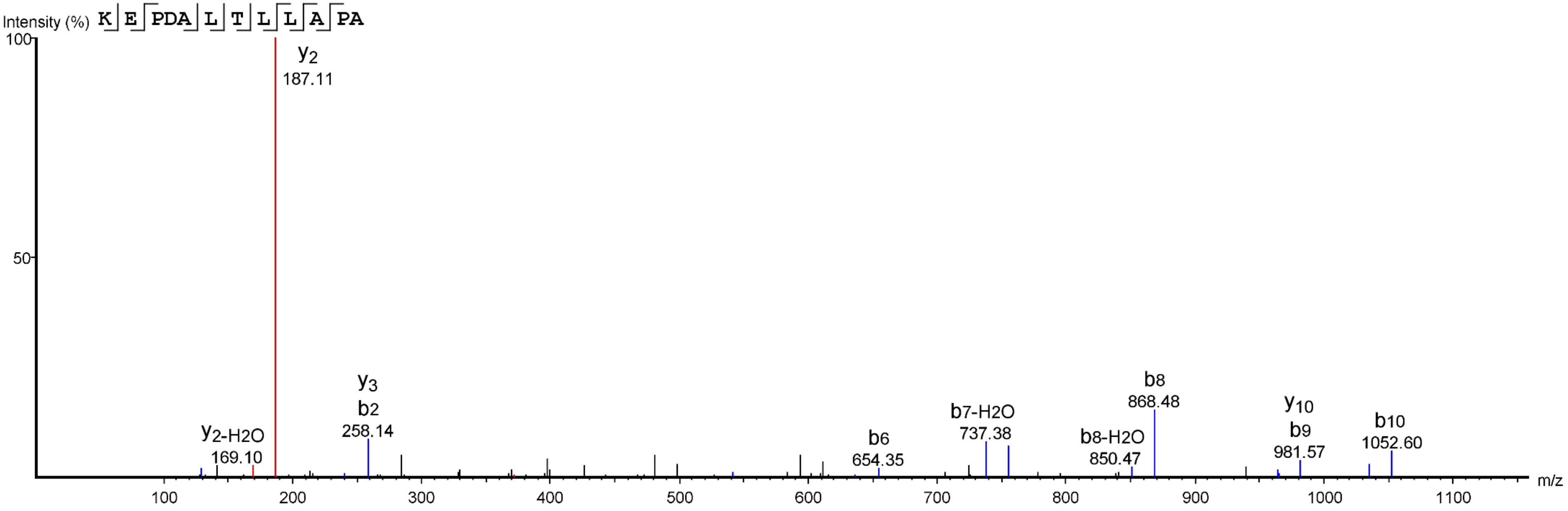

YMT57

Scan #18017

A-018: HIF1A*$_{(392-403)}$: KEPDALTLLAPA

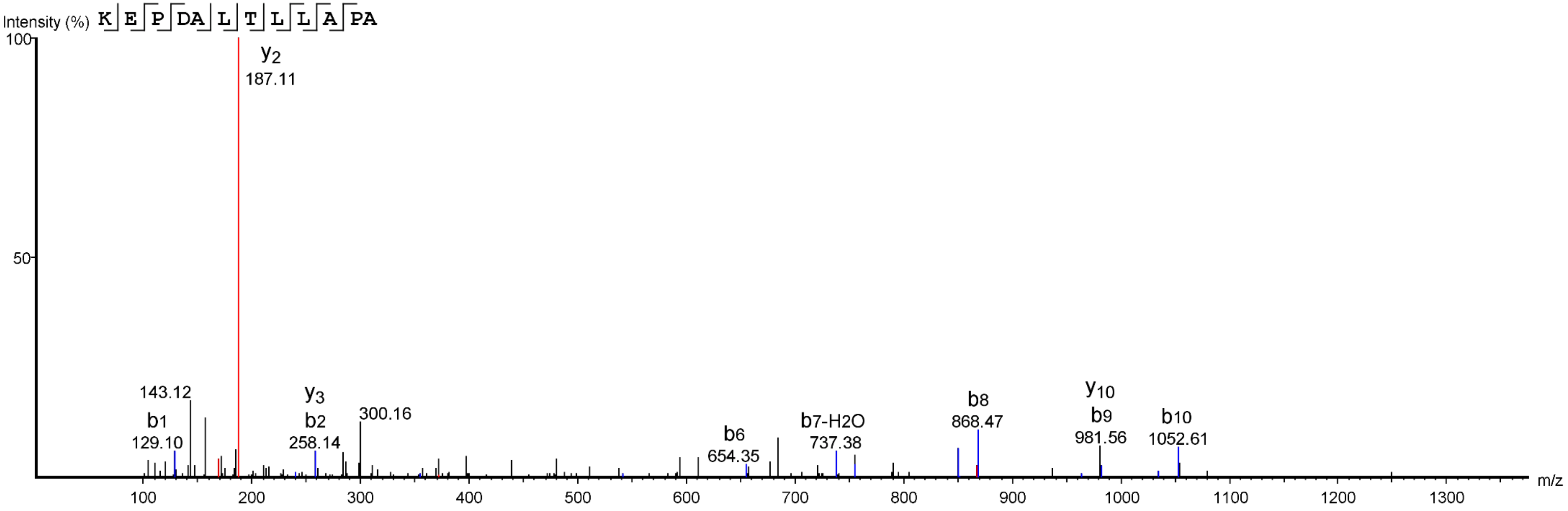

YMT58
Scan #19119

A-019: HIF1A*$_{(392-403)}$: KEPDALTLLAP(+15.99)A

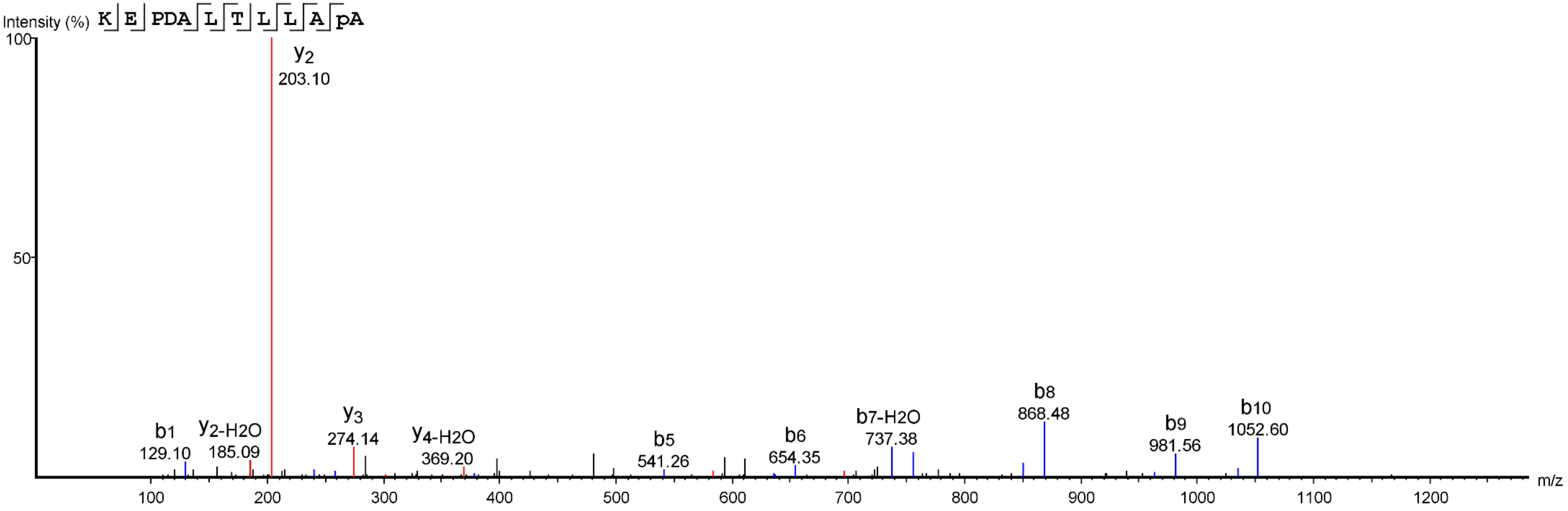

YMT57
Scan #16535

A-020: HIF1A*$_{(392-403)}$: KEPDALTLLAP(+15.99)A

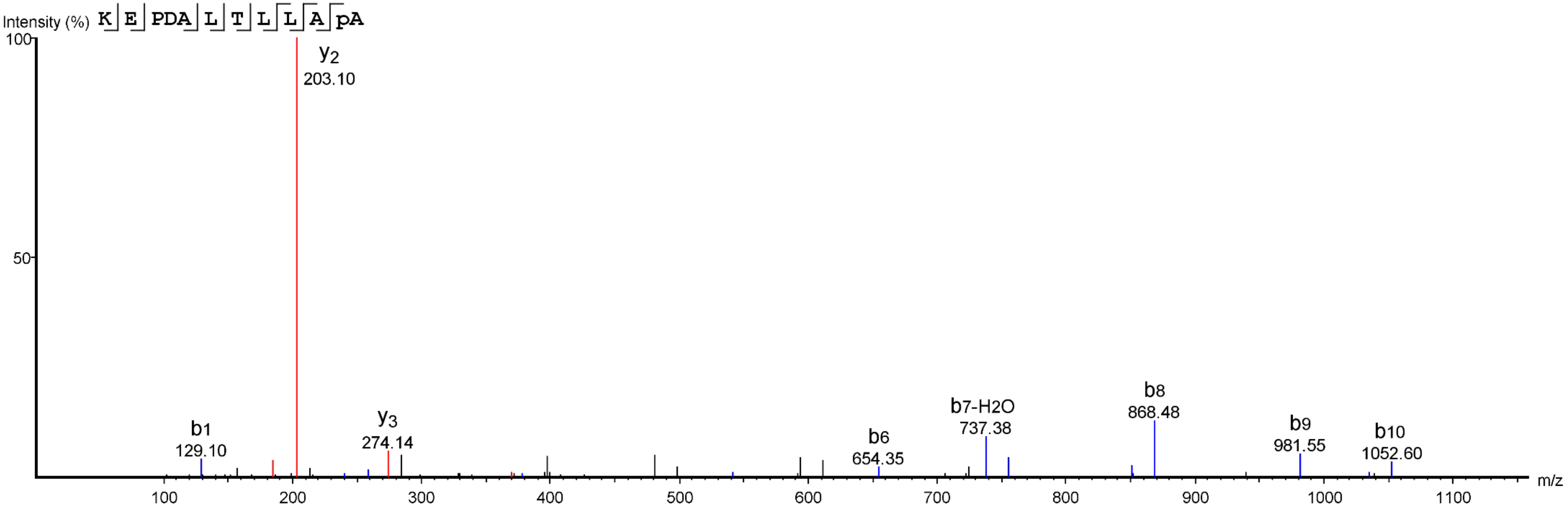

YMT58
Scan #17486

A-021: HIF1A*$_{(548-575)}$: NPFSTQDTDLDLEALAPYIPADDDFQLR

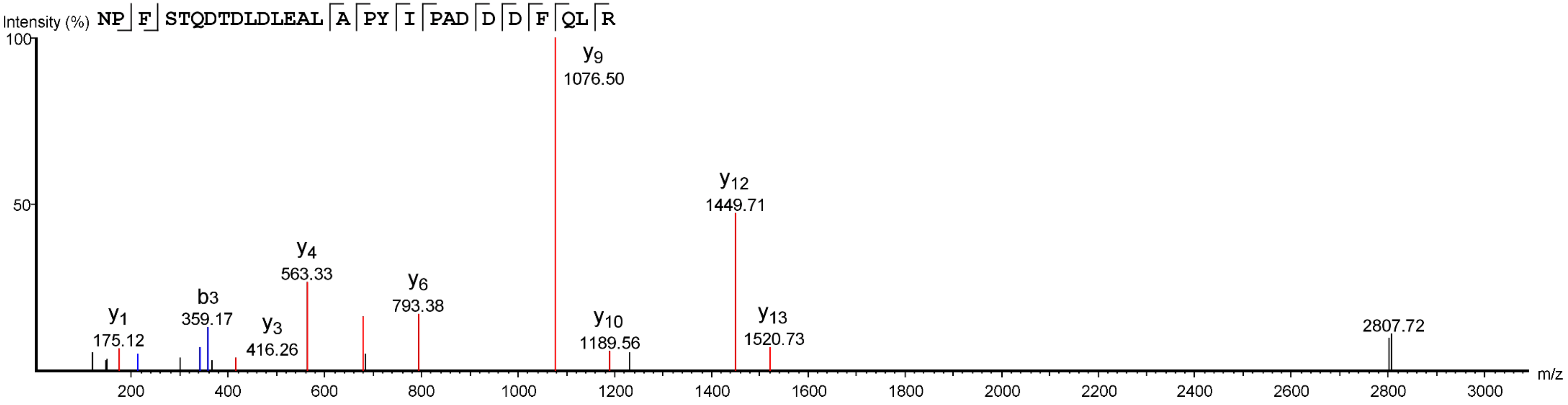

YMT25
Scan #26114

A-022: HIF1A*(548-575): NPFSTQDTDLDLEALAPYIPADDDFQLR

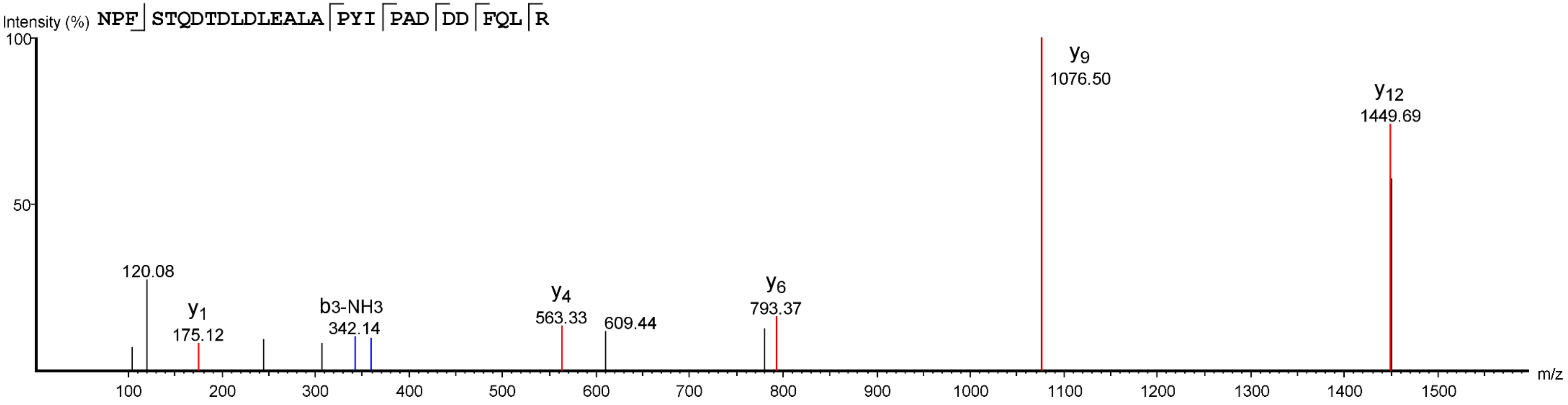

YMT26
Scan #25868

A-023: HIF1A*$_{(548-575)}$: NPFSTQDTDLDLEALAP(+15.99)YIPADDDFQLR

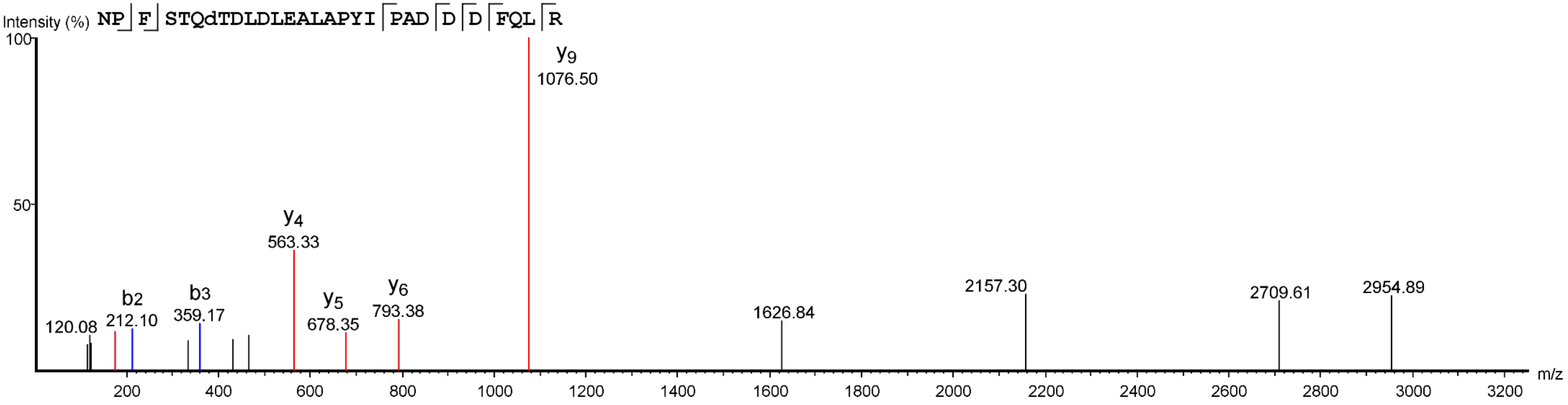

YMT25
Scan #26084

A-024: HIF1A*$_{(548-575)}$: NPFSTQDTDLDLEALAP(+15.99)YIPADDDFQLR

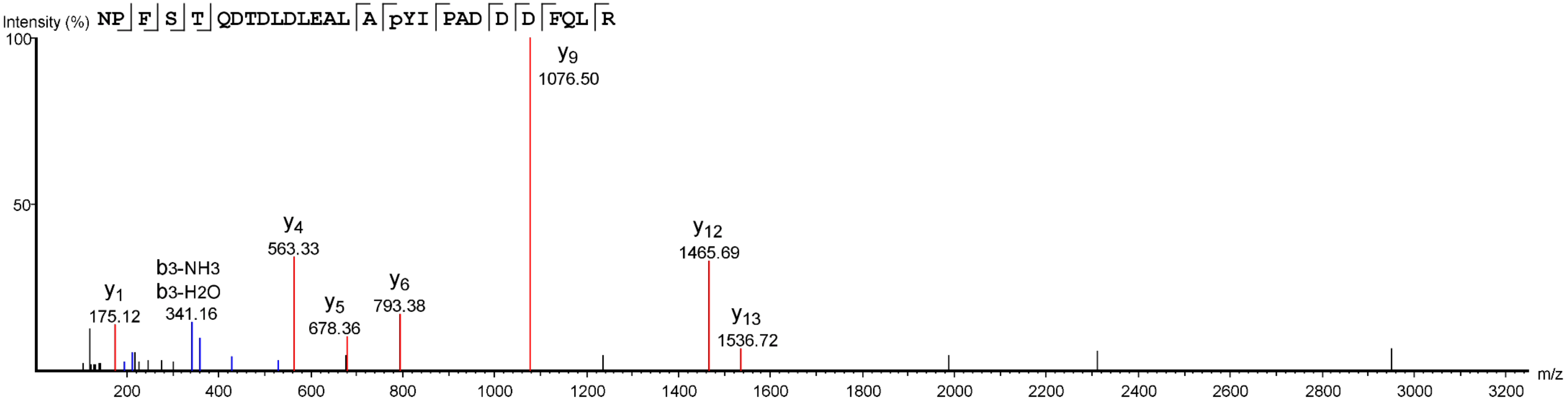

YMT26

Scan #25866

A-025: HIF1A*$_{(548-575)}$: NPFSTQDTDLDLEALAP(+15.99)YIPADDDFQLR

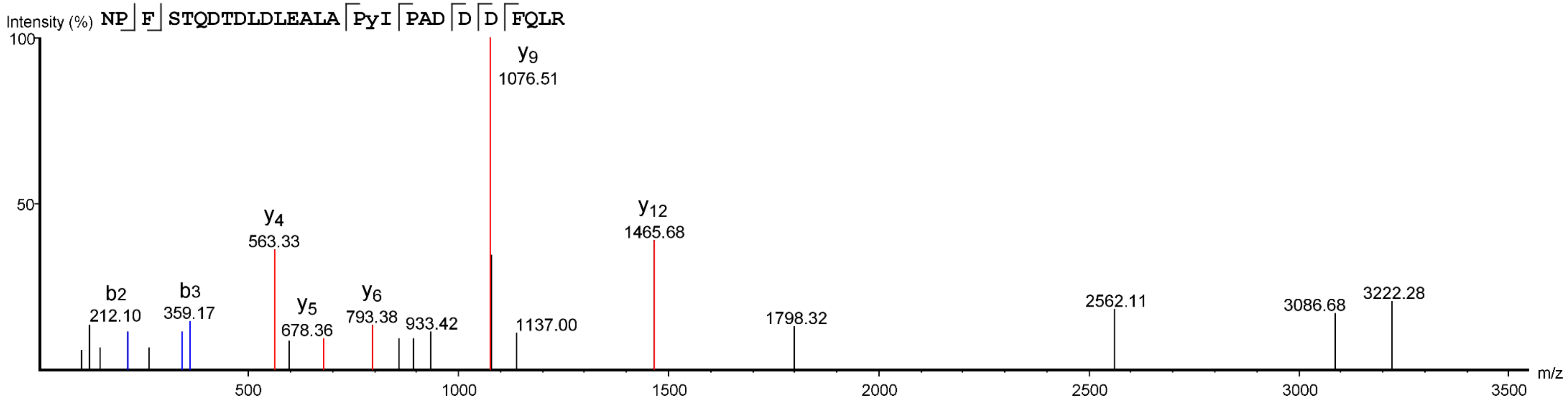

YMT28
Scan #25885

A-026: HIF1A*$_{(392\text{-}403)}$: KEPDALTLLAPA

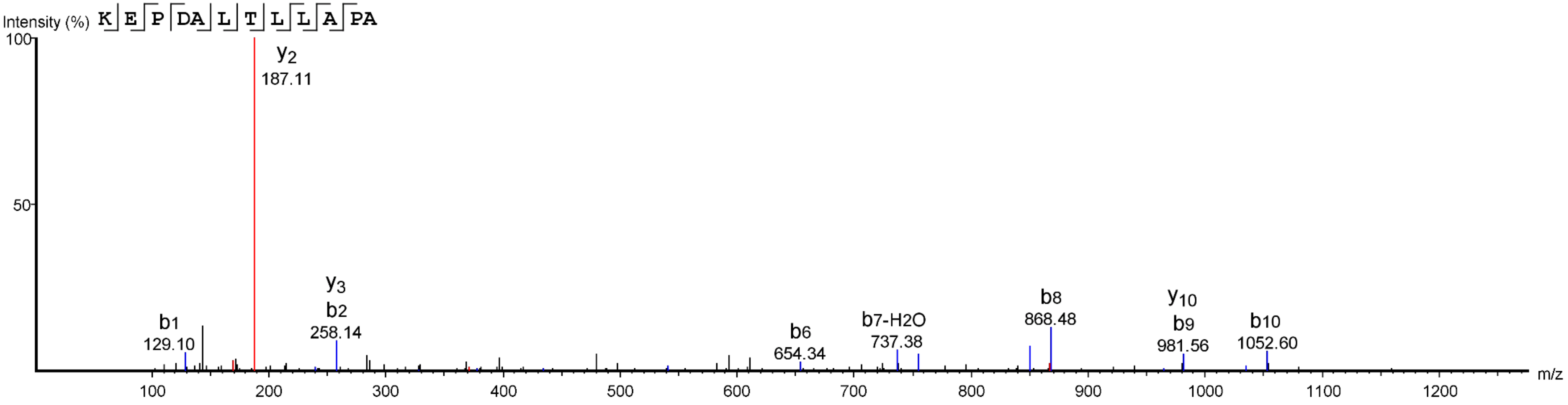

YMT25
Scan #18201

A-027: HIF1A*$_{(392-403)}$: KEPDALTLLAPA

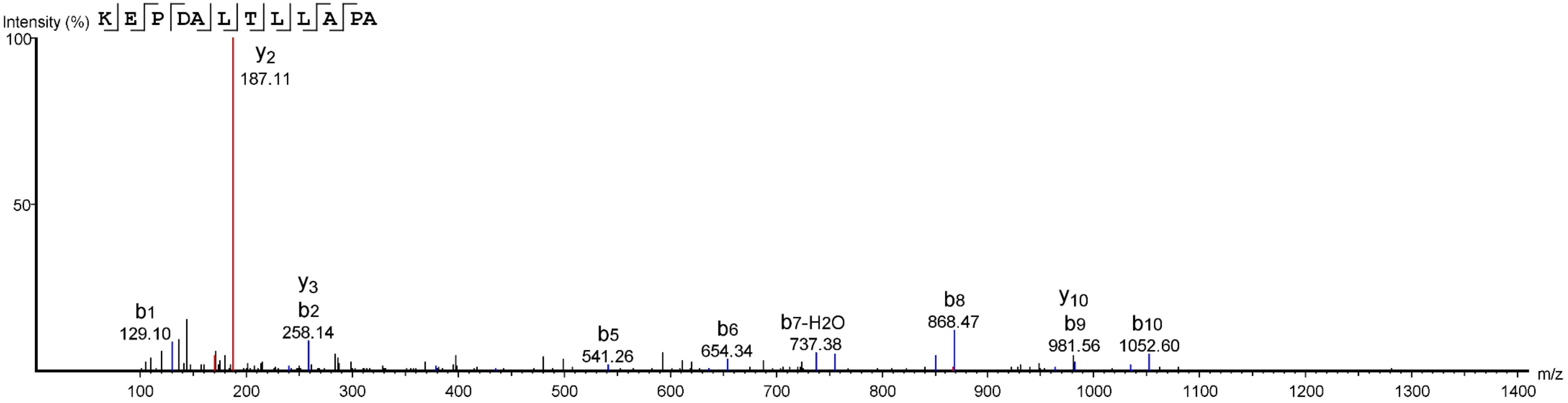

YMT28
Scan #18017

A-028: HIF1A*$_{(392-403)}$: KEPDALTLLAP(+15.99)A

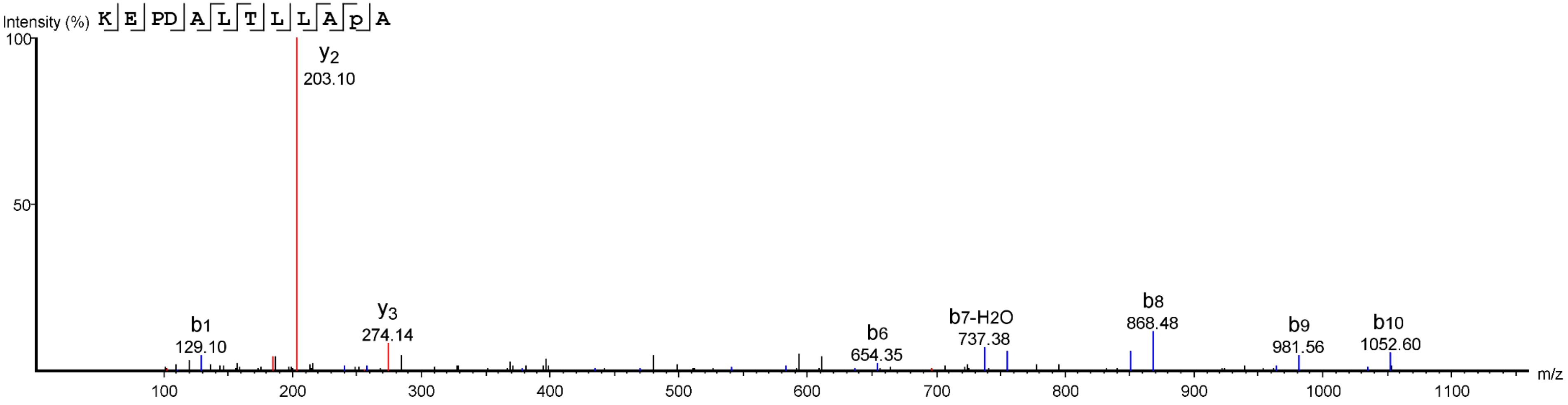

YMT26

Scan #16342

A-029: HIF1A*$_{(548-575)}$: NPFSTQDTDLDLEALAPYIPADDDFQLR

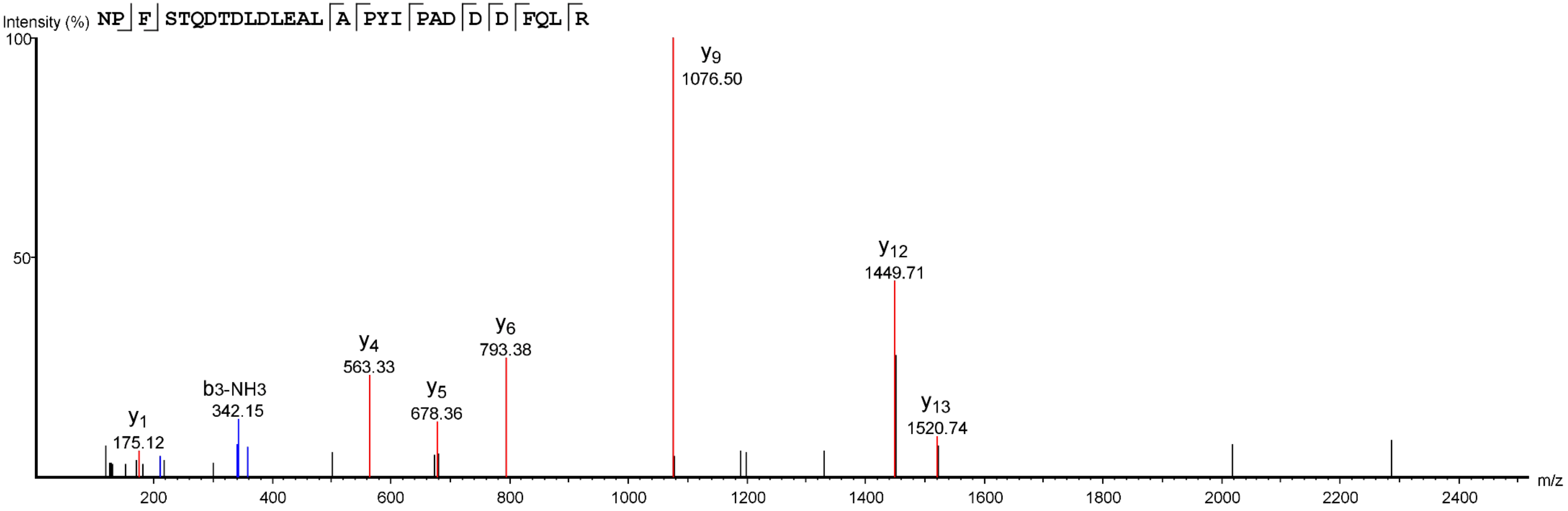

YMT51
Scan #26692

A-030: HIF1A*$_{(548-575)}$: NPFSTQDTDLDLEALAPYIPADDDFQLR

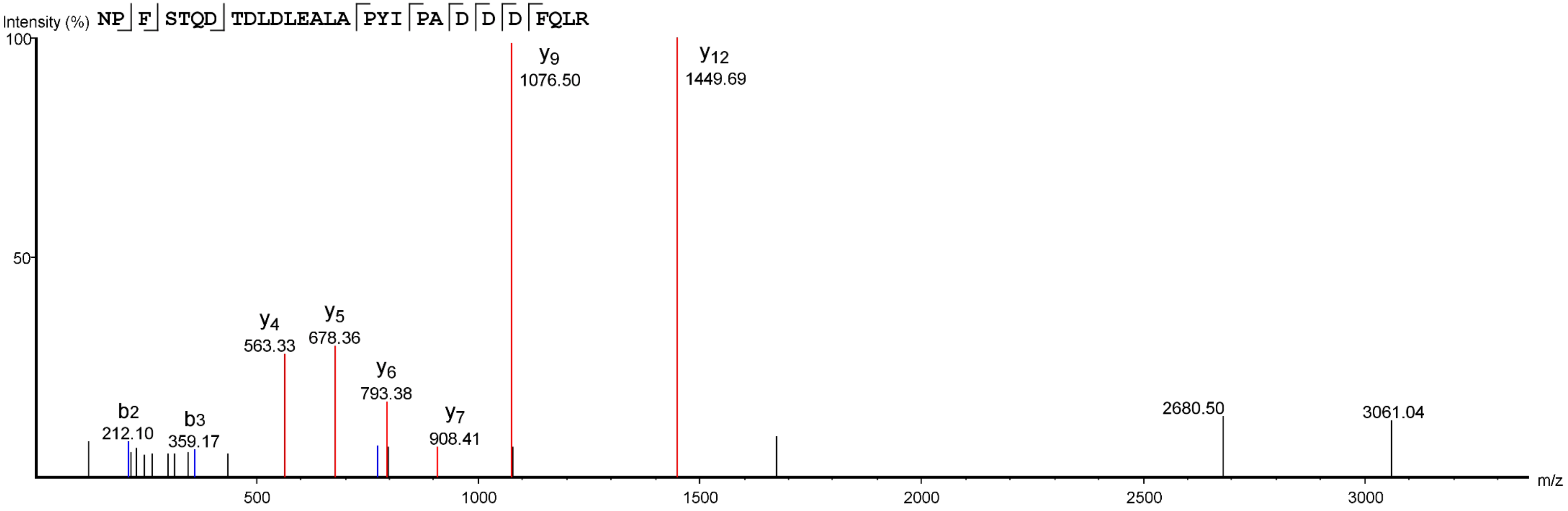

YMT52
Scan #26577

A-031: HIF1A*$_{(548-575)}$: NPFSTQDTDLDLEALAP(+15.99)YIPADDDFQLR

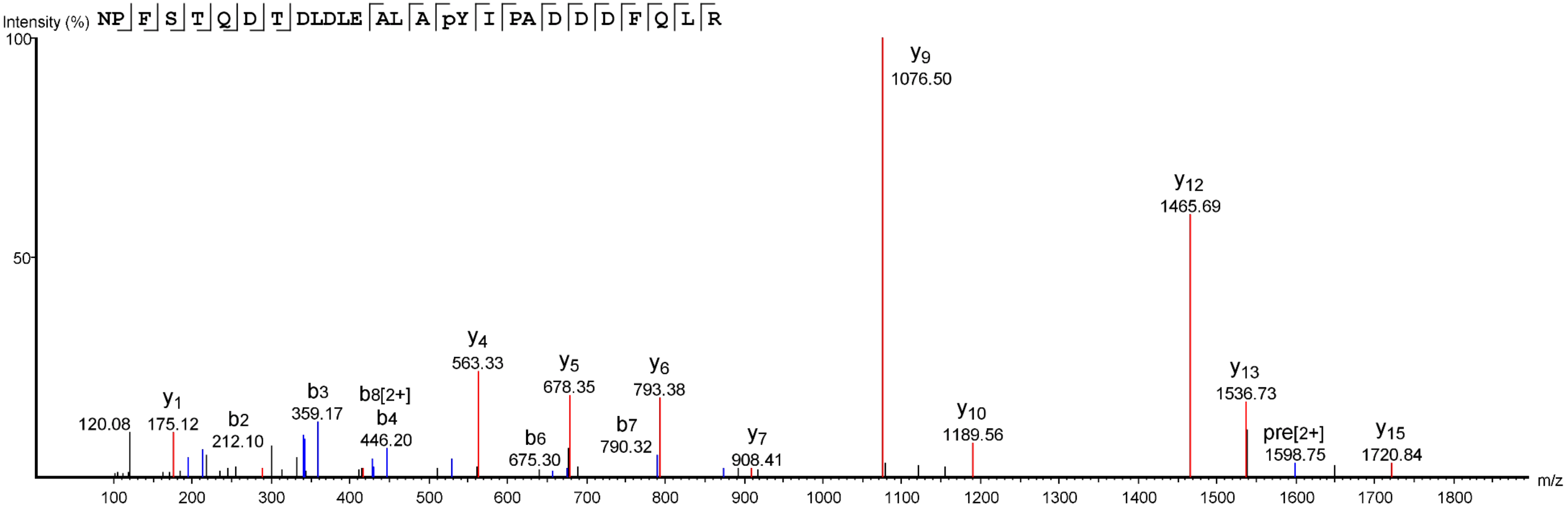

YMT51
Scan #26560

A-032: HIF1A*(548-575): NPFSTQDTDLDLEALAPYIPADDDFQLR

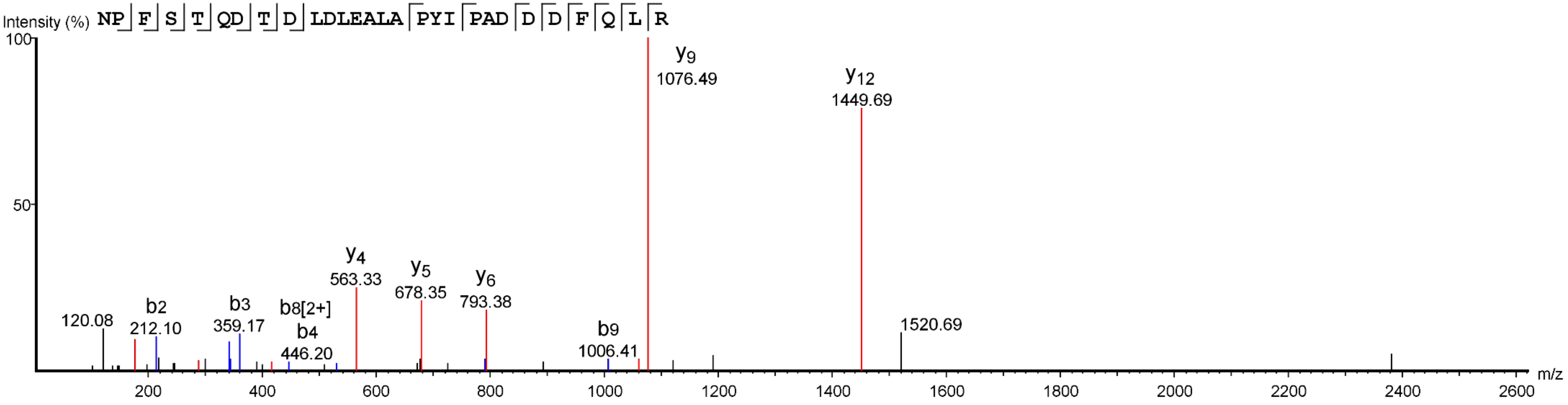

YMT41
Scan #26077

A-033: HIF1A*(548-575): NPFSTQDTDLDLEALAPYIPADDDFQLR

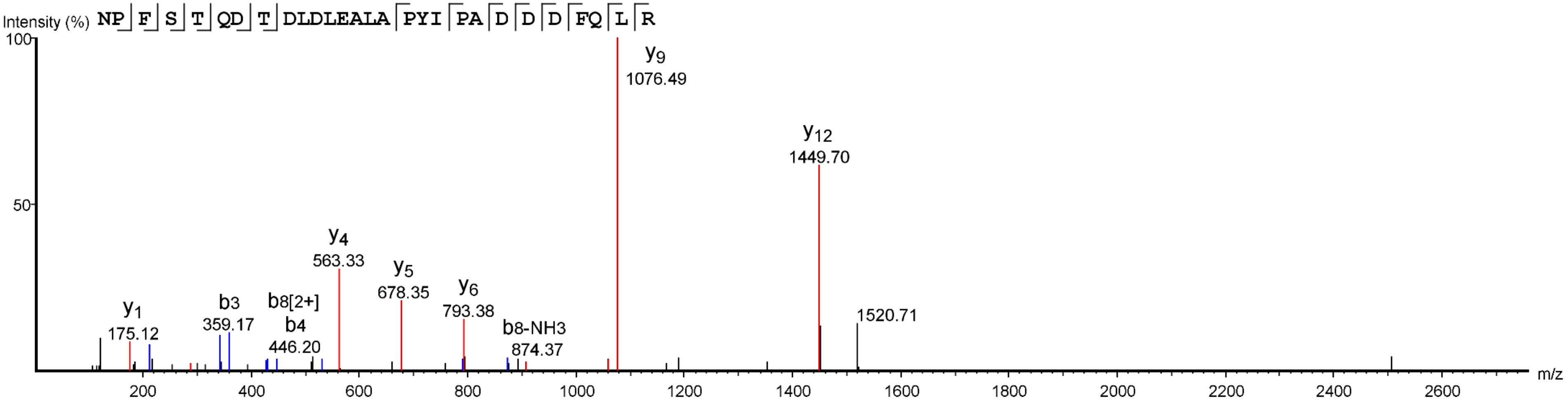

YMT42
Scan #26788

A-034: HIF1A*$_{(548-575)}$: NPFSTQDTDLDLEALAP(+15.99)YIPADDDFQLR

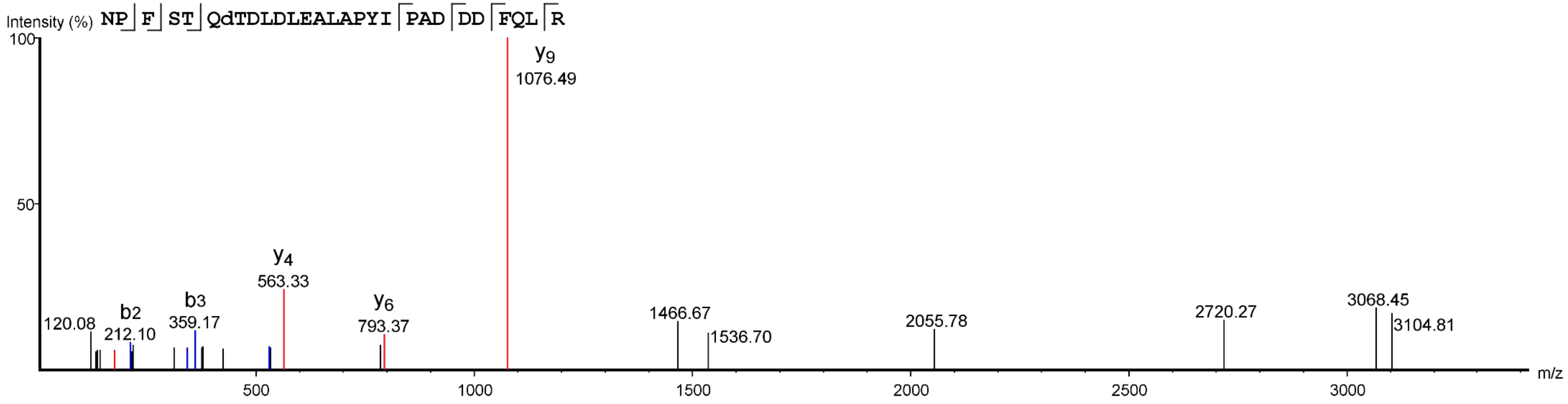

YMT41
Scan #26016

A-035: HIF1A*$_{(548-575)}$: NPFSTQDTDLDLEALAP(+15.99)YIPADDDFQLR

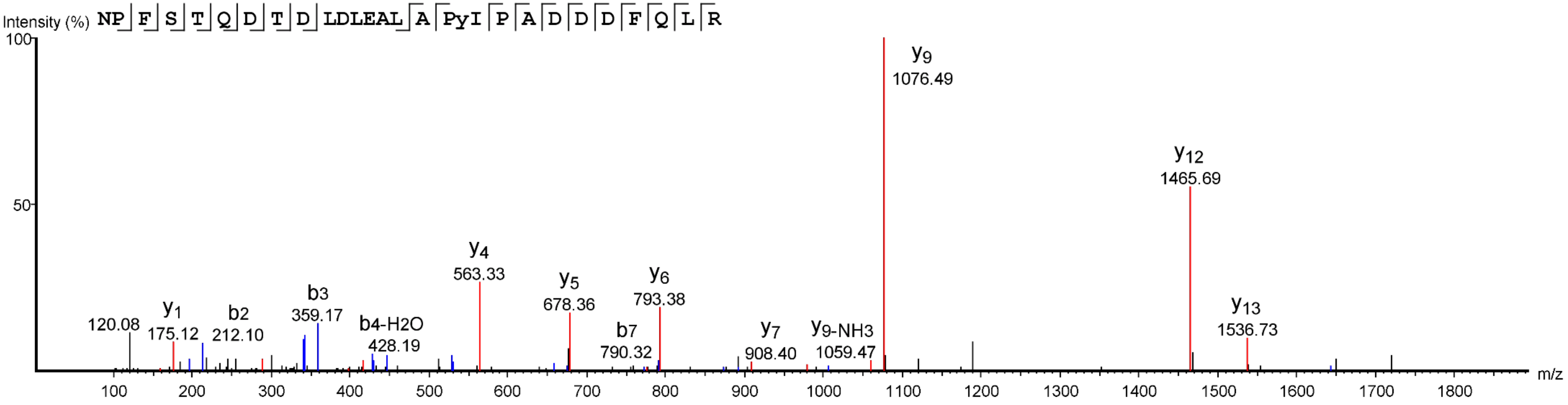

YMT42
Scan #26785

A-036: HIF1A*$_{(392-403)}$: KEPDALTLLAPA

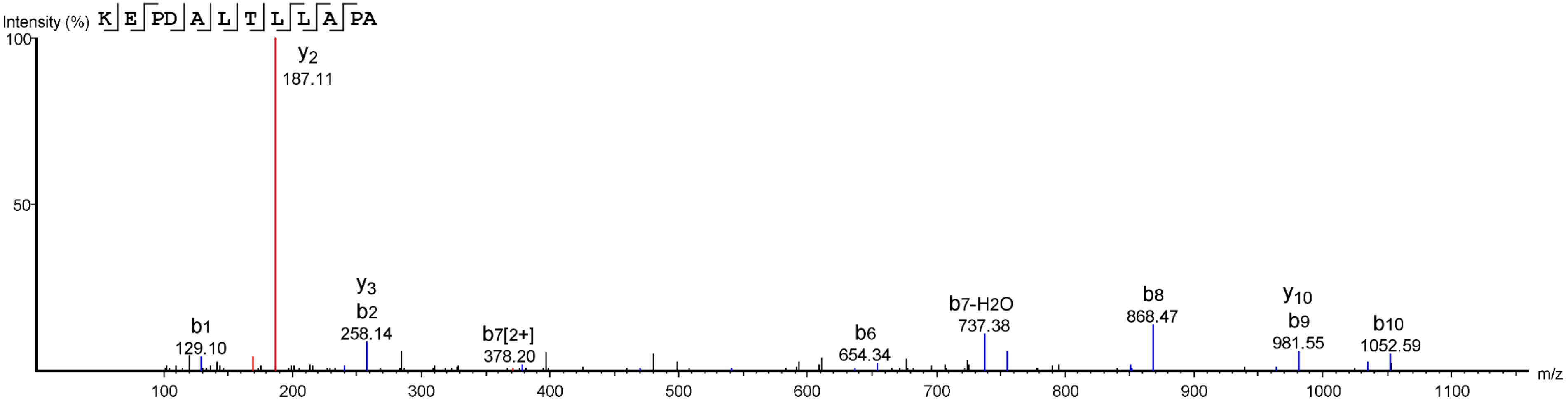

YMT41
Scan #18035

A-037: HIF1A*$_{(548-575)}$: NPFSTQDTDLDLEALAPYIPADDDFQLR

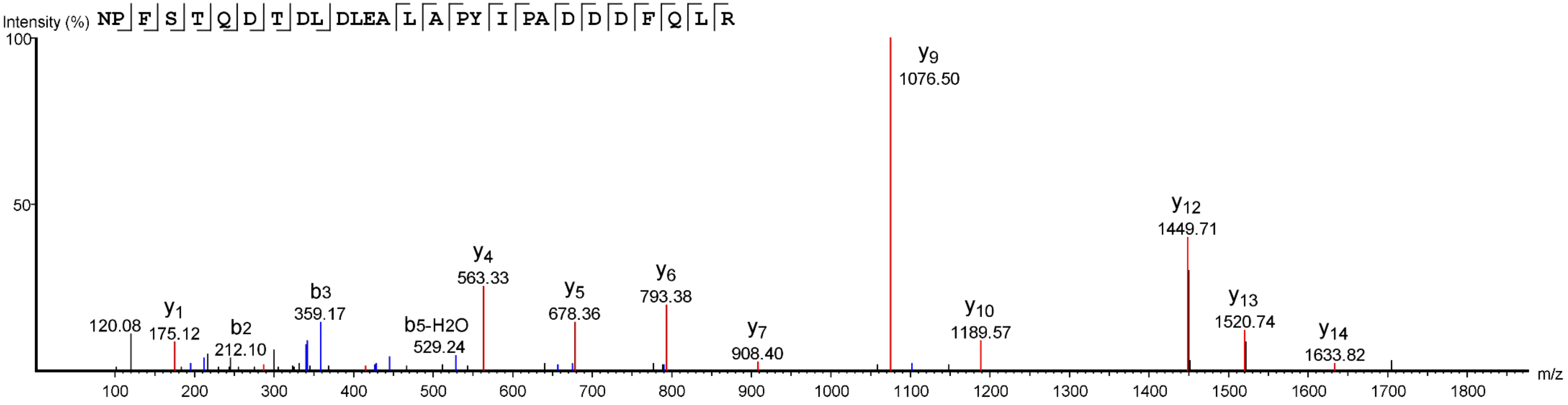

YMT65
Scan #23880

A-038: HIF1A*$_{(548-575)}$: NPFSTQDTDLDLEALAPYIPADDDFQLR

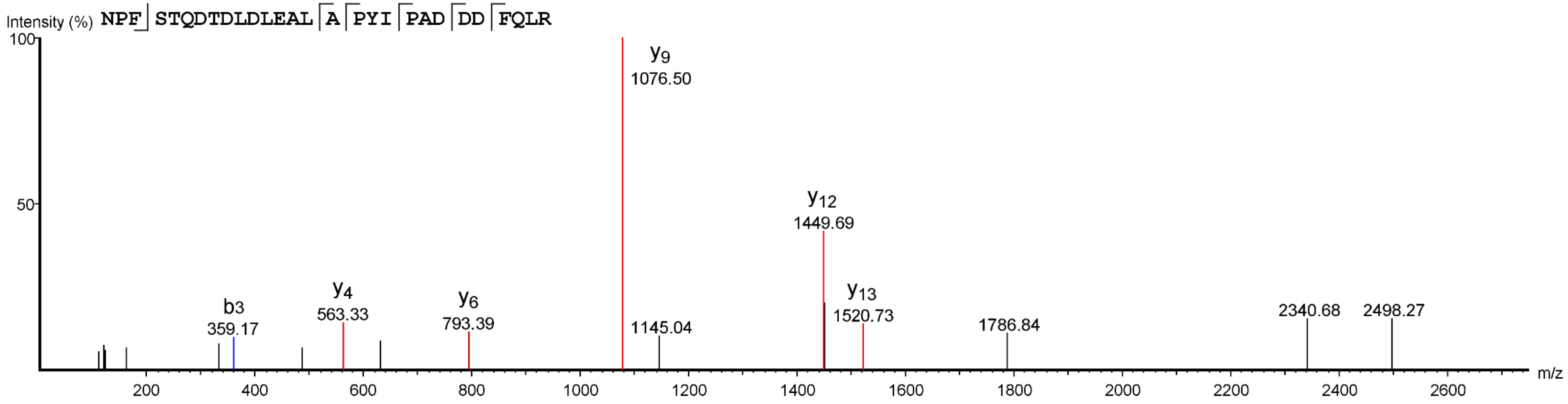

YMT66
Scan #23824

A-039: HIF1A*$_{(548-575)}$: NPFSTQDTDLDLEALAP(+15.99)YIPADDDFQLR

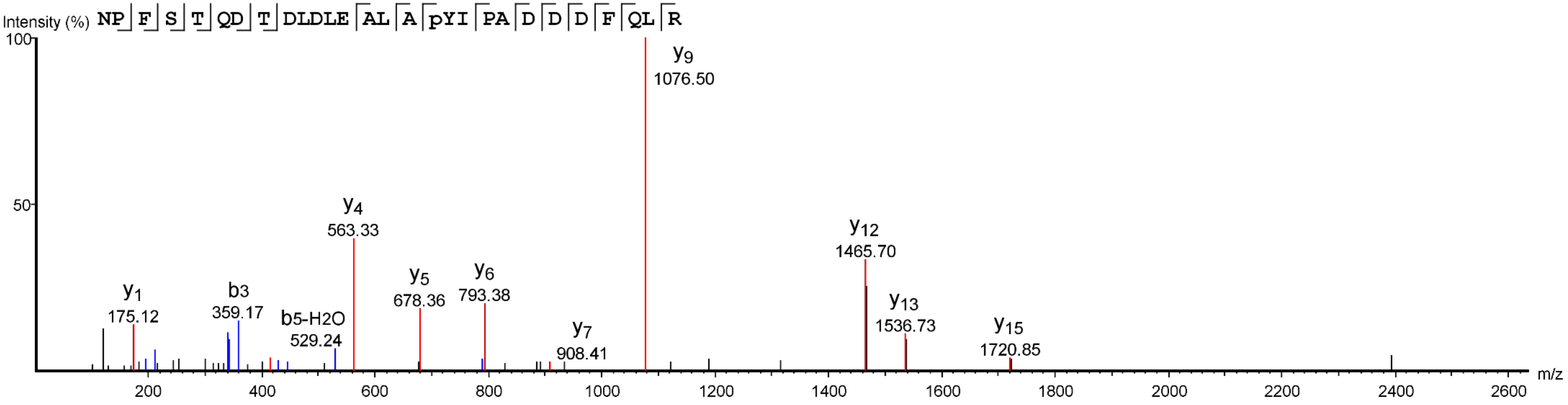

YMT65
Scan #23864

A-040: HIF1A*$_{(548-575)}$: NPFSTQDTDLDLEALAP(+15.99)YIPADDDFQLR

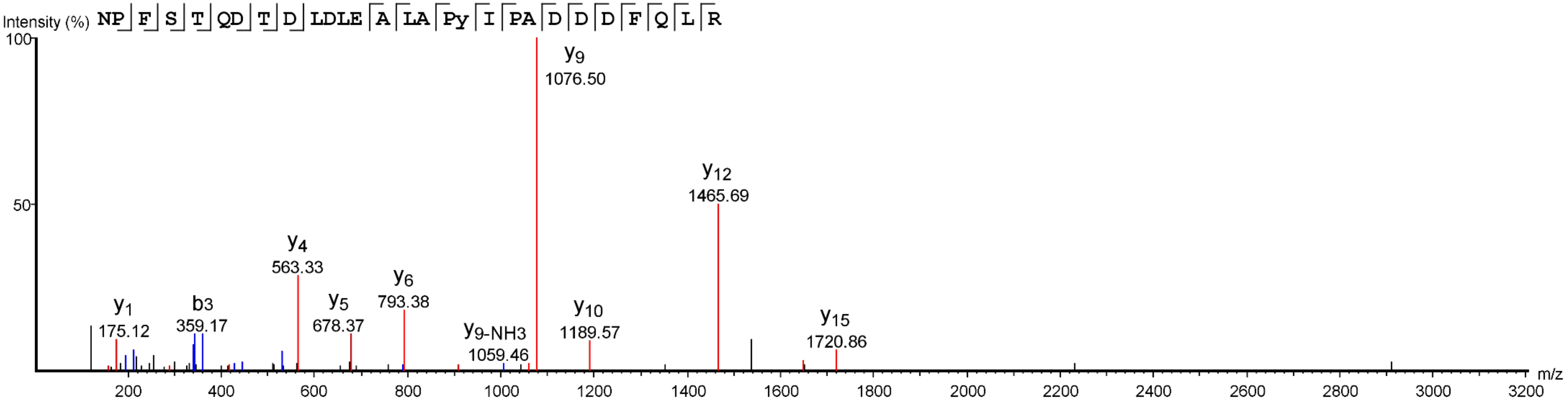

YMT66
Scan #23758

A-041: HIF1A*$_{(548-575)}$: NPFSTQDTDLDLEALAPYIPADDDFQLR

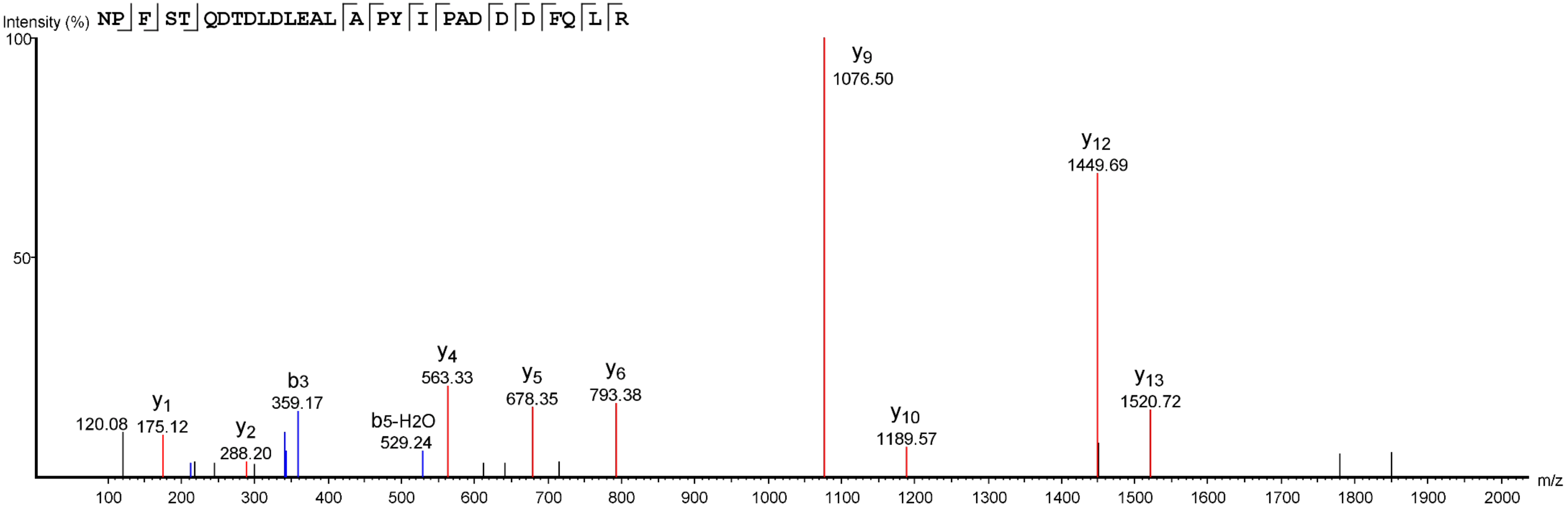

YMT61
Scan #23277

A-042: HIF1A*$_{(548-575)}$: NPFSTQDTDLDLEALAPYIPADDDFQLR

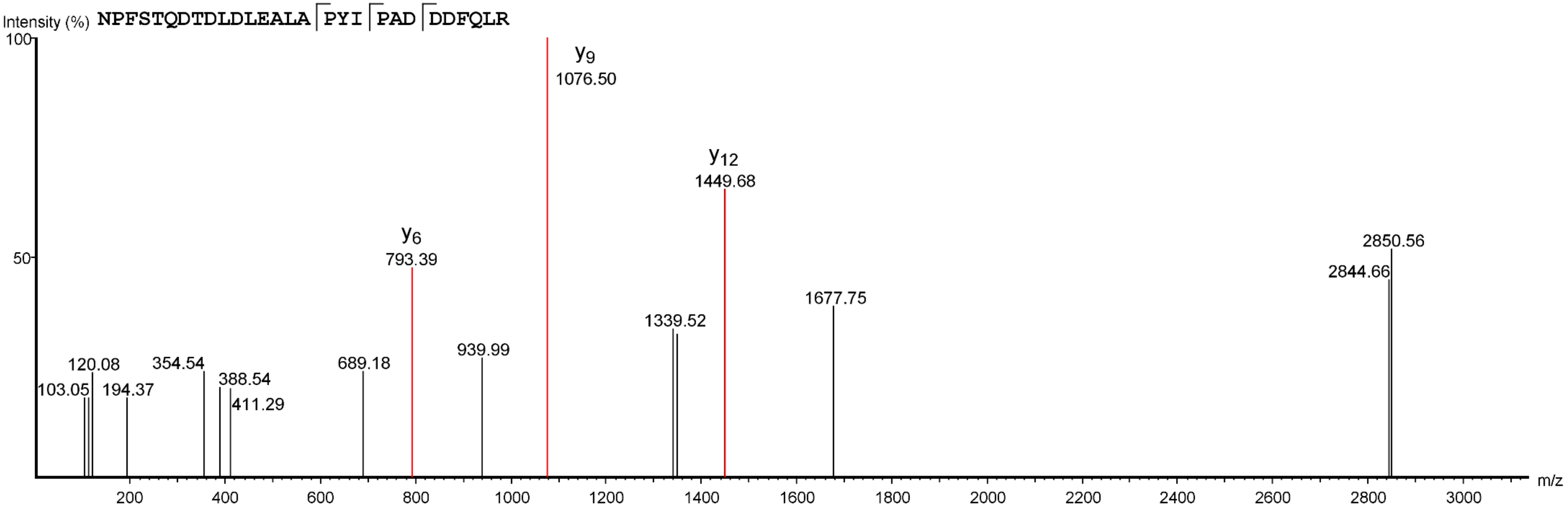

YMT62
Scan #22916

A-043: HIF1A*$_{(548-575)}$: NPFSTQDTDLDLEALAP(+15.99)YIPADDDFQLR

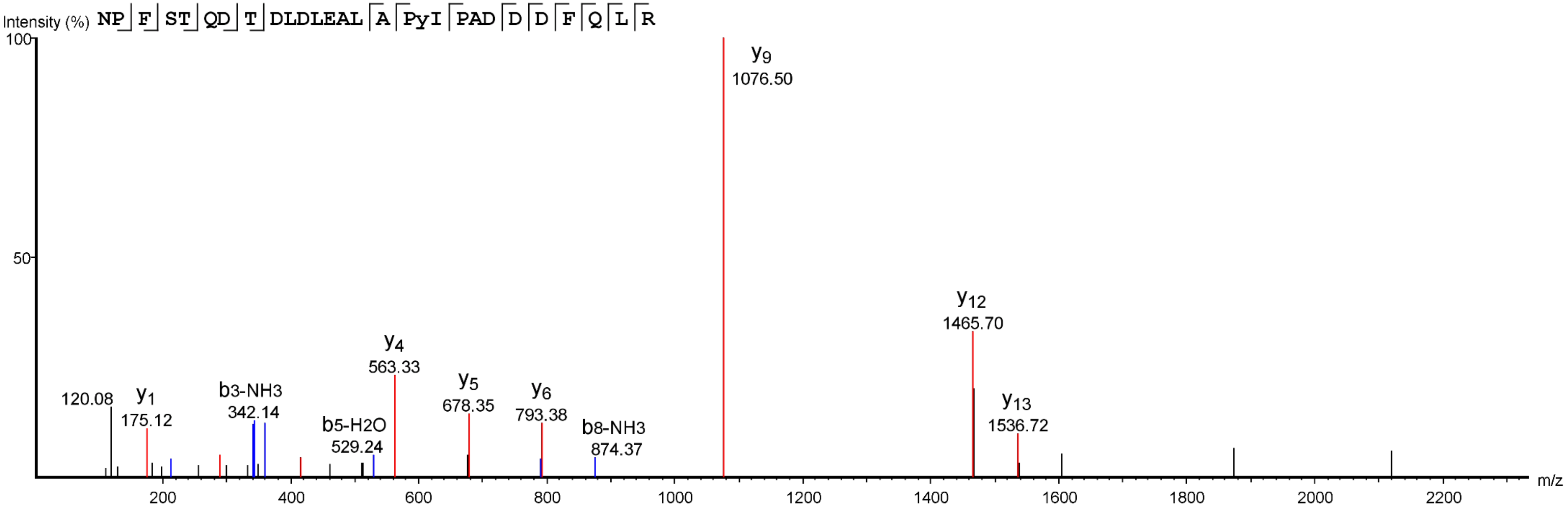

YMT61
Scan #23268

A-044: HIF1A*$_{(548-575)}$: NPFSTQDTDLDLEALAP(+15.99)YIPADDDFQLR

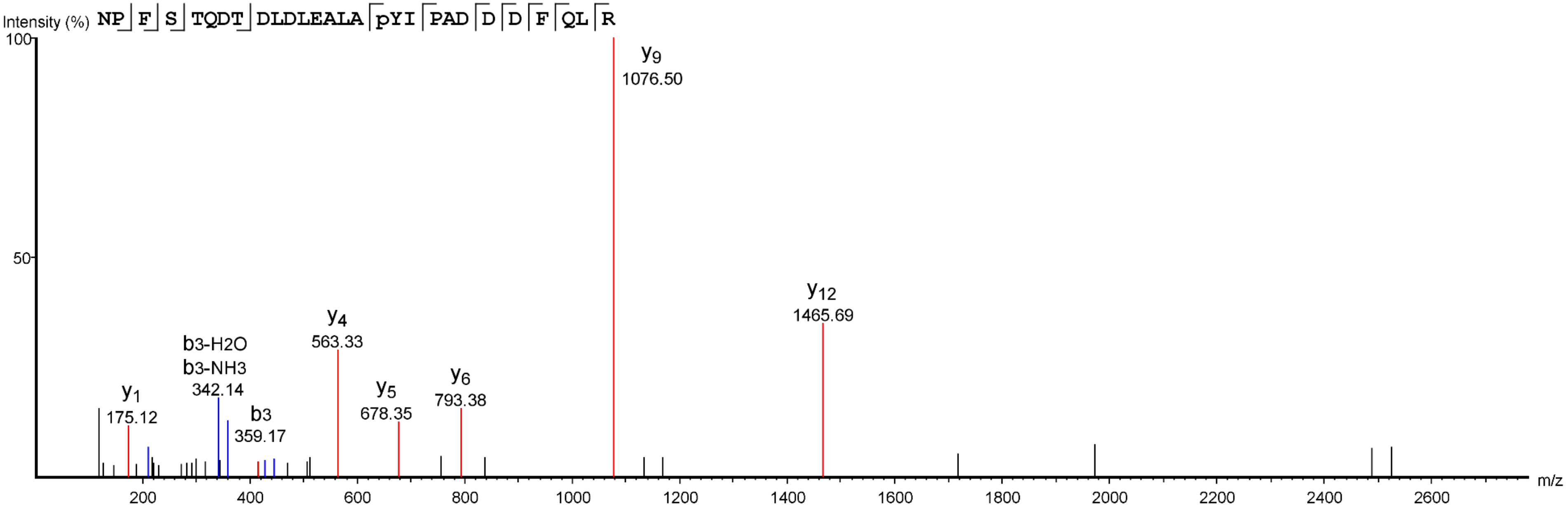

YMT62
Scan #22900

A-045: HIF1A*$_{(392-403)}$: KEPDALTLLAP(+15.99)A

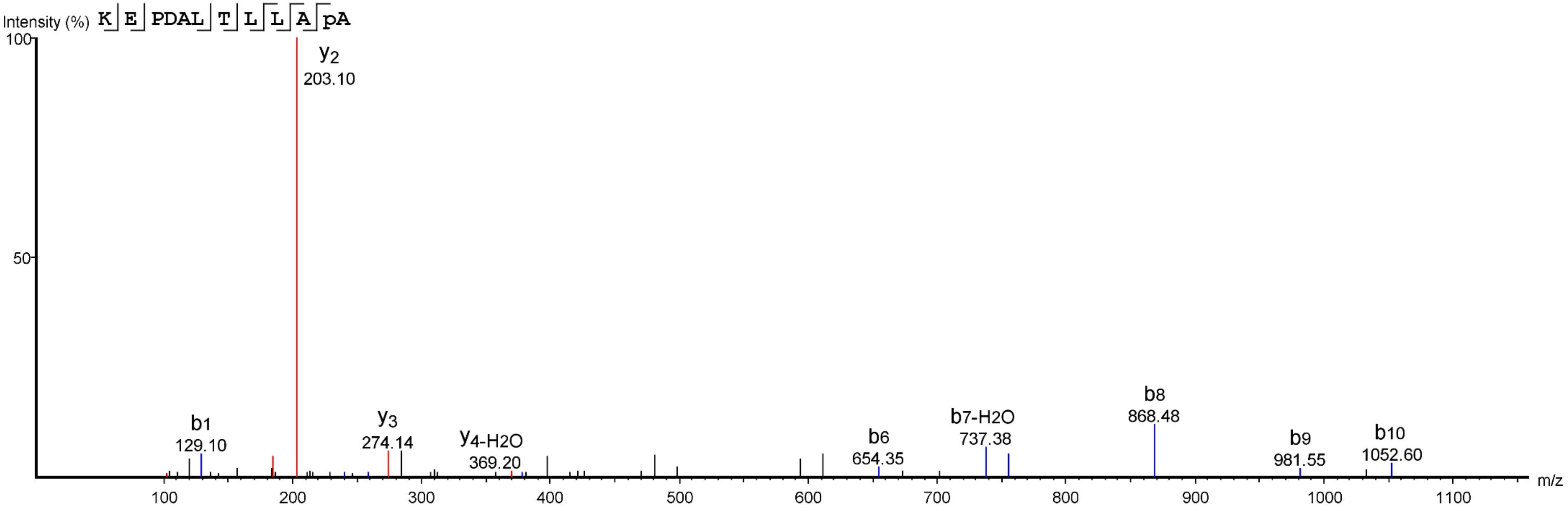

YMT62
Scan #13681

A-046: HIF1A*$_{(392-411)}$: KEPDALTLLAP(+15.99)AAGDTIISL

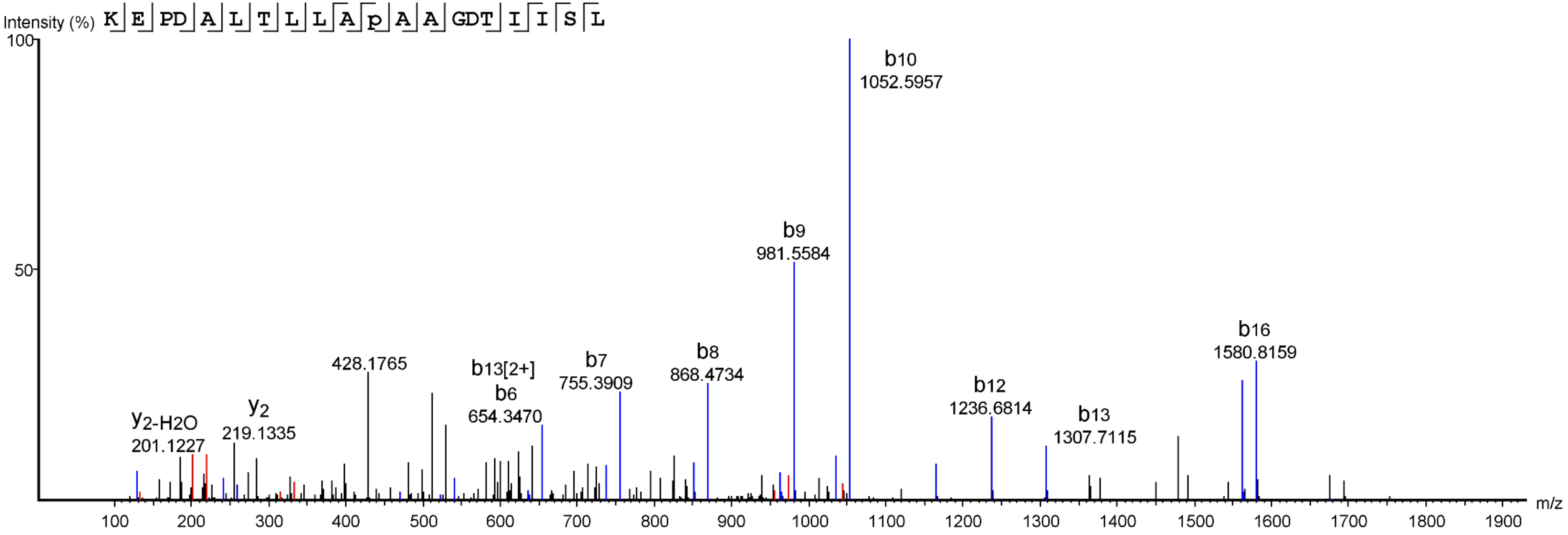

YMT109
Scan #25298

A-047: HIF1A*$_{(392-411)}$: KEPDALTLLAP(+15.99)AAGDTIISL

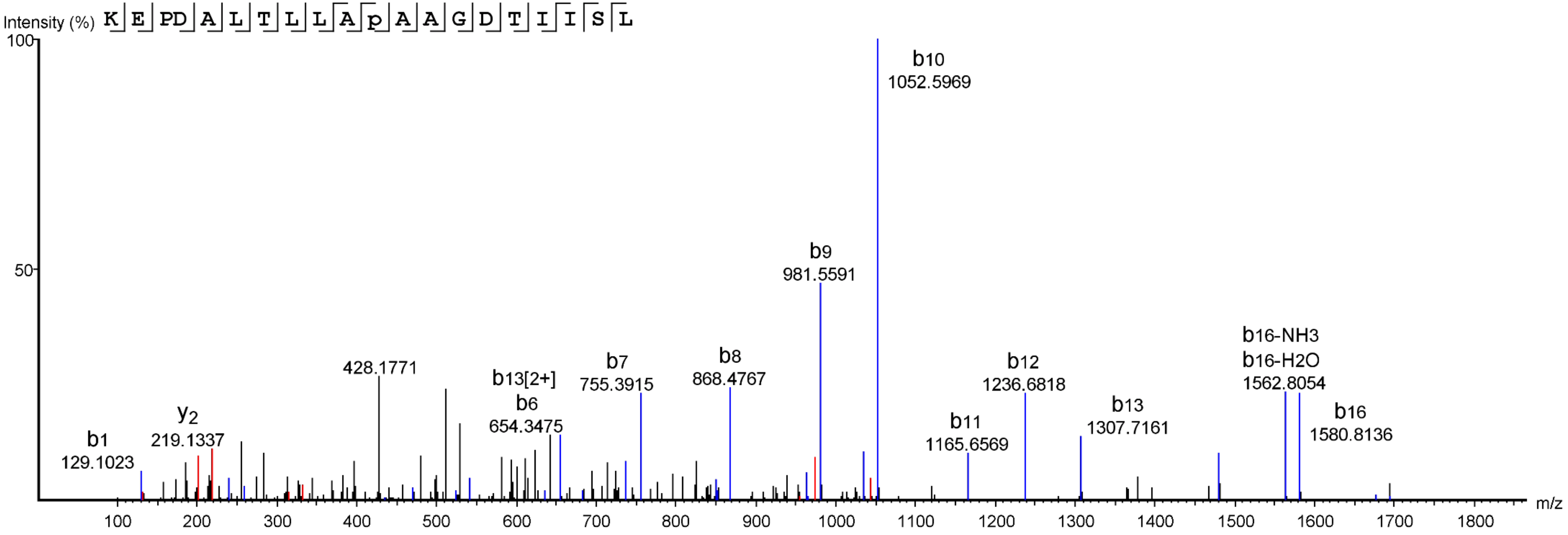

YMT110
Scan #25316

A-048: HIF1A*$_{(548-575)}$: NPFSTQDTDLDLEALAPYIPADDDFQLR

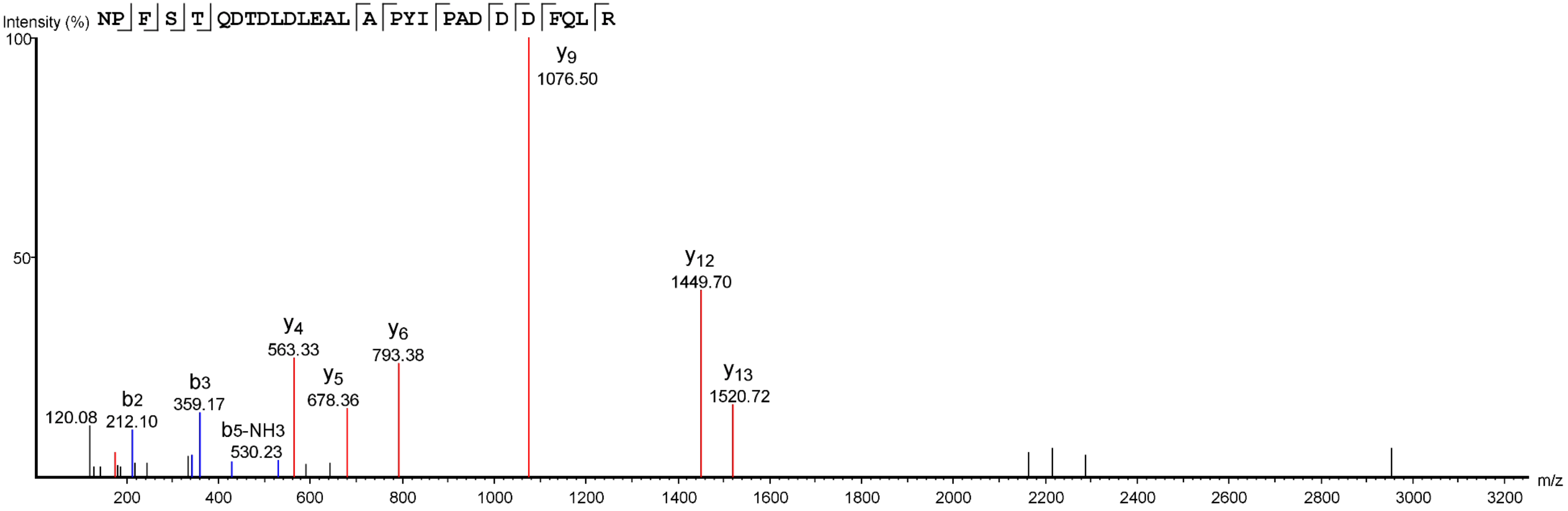

YMT59
Scan #24828

A-049: HIF1A*(548-575): NPFSTQDTDLDLEALAPYIPADDDFQLR

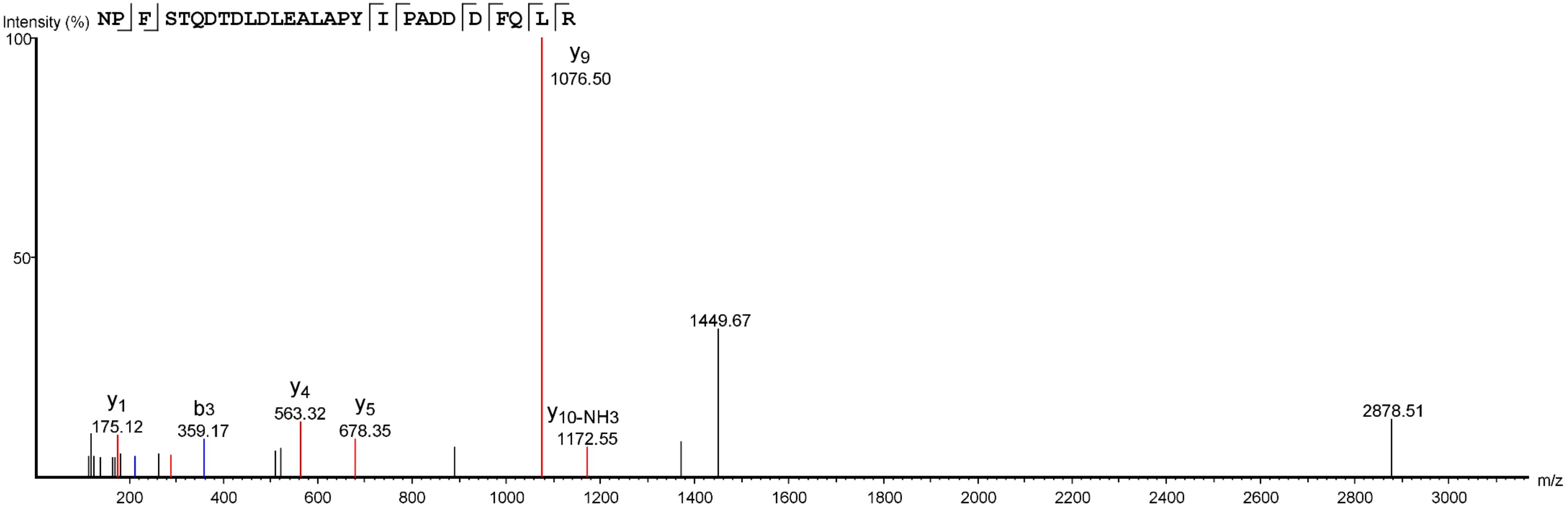

YMT60
Scan #25256

A-050: HIF1A*(548-575): NPFSTQDTDLDLEALAP(+15.99)YIPADDDFQLR

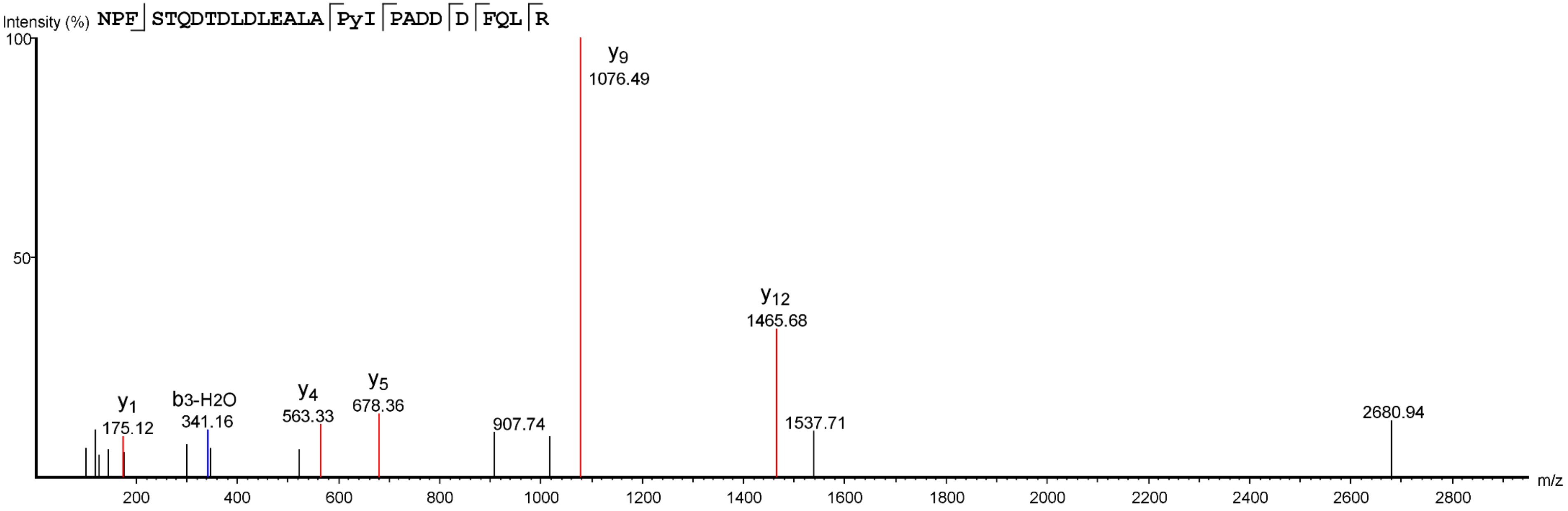

YMT59
Scan #24786

A-051: HIF1A*(548-575): NPFSTQDTDLDLEALAP(+15.99)YIPADDDFQLR

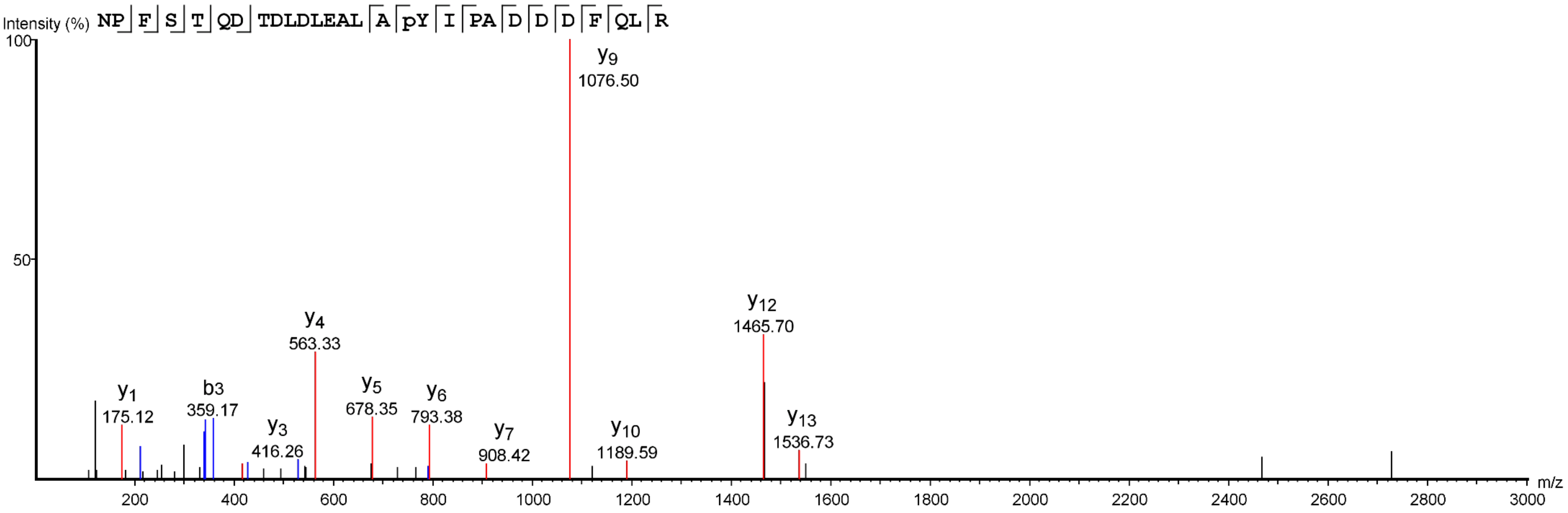

YMT60

Scan #25207

A-052: HIF1A*(392-403): KEPDALTLLAPA

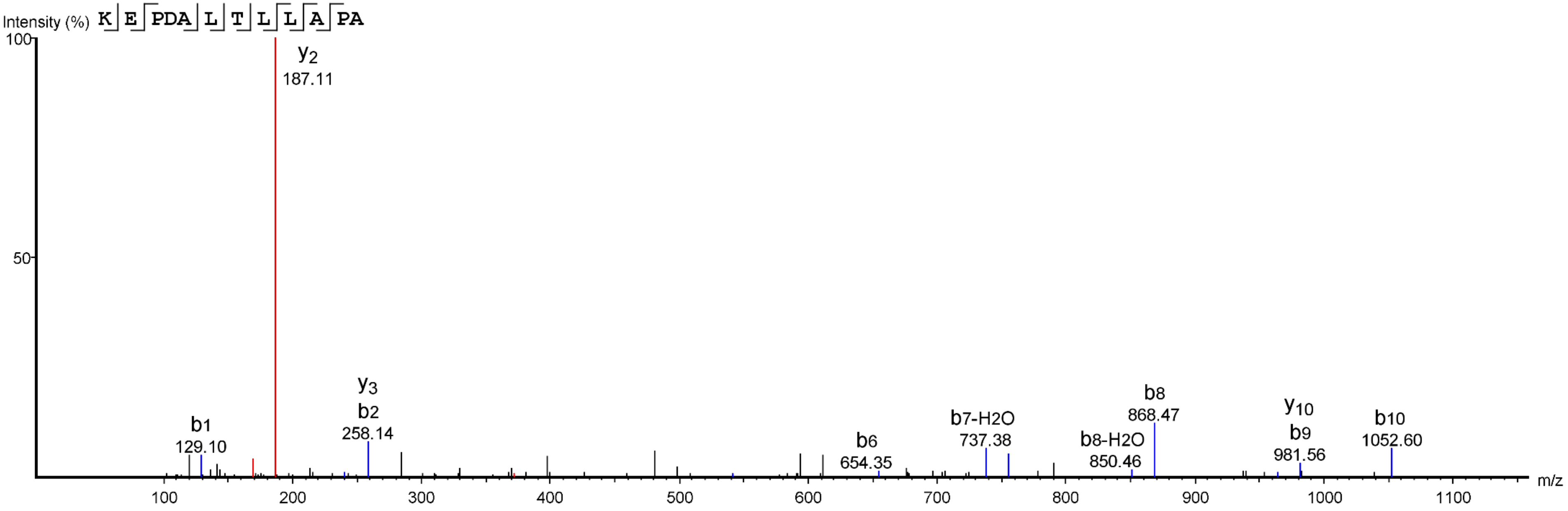

YMT59
Scan #17060

A-053: HIF1A*$_{(392-403)}$: KEPDALTLLAP(+15.99)A

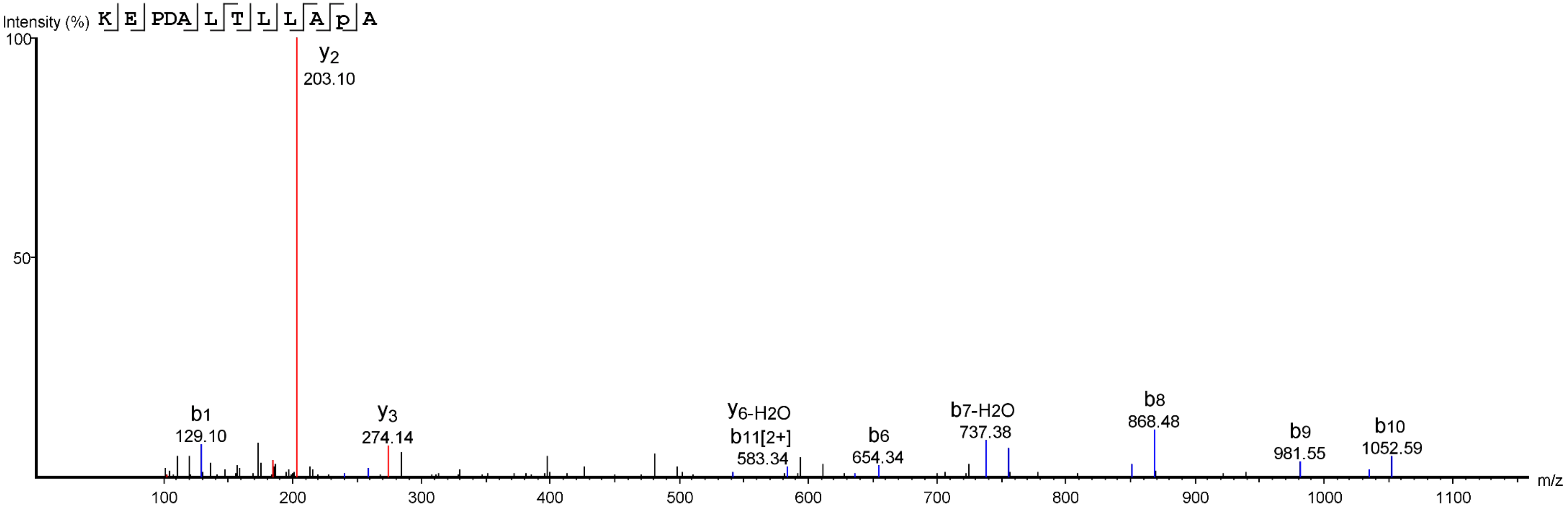

YMT60
Scan #15751

A-054: HIF1A*$_{(548-575)}$: NPFSTQDTDLDLEALAPYIPADDDFQLR

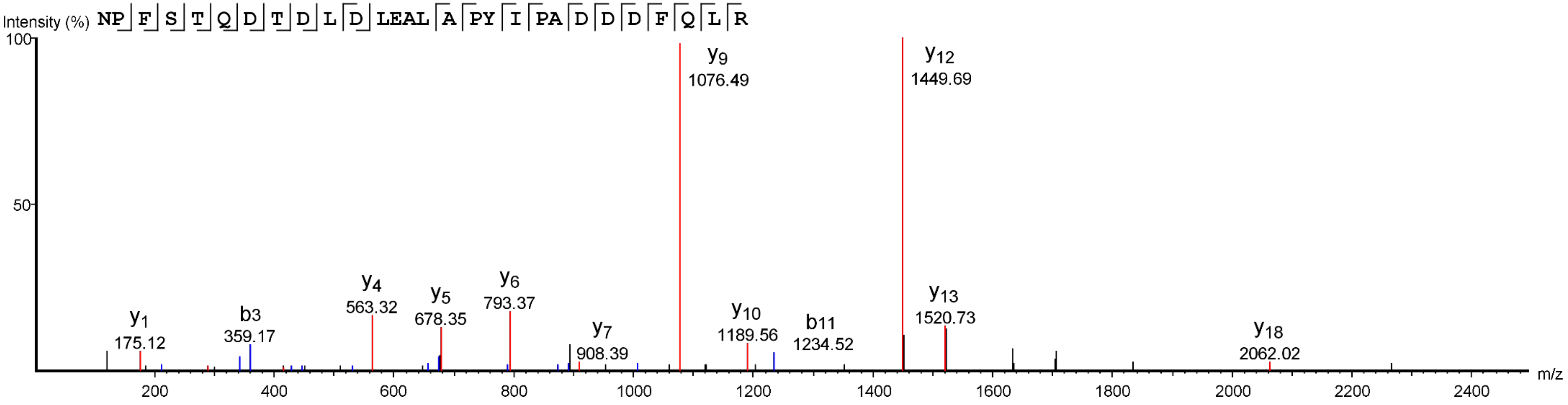

YMT69
Scan #25372

A-055: HIF1A*$_{(548-575)}$: NPFSTQDTDLDLEALAPYIPADDDFQLR

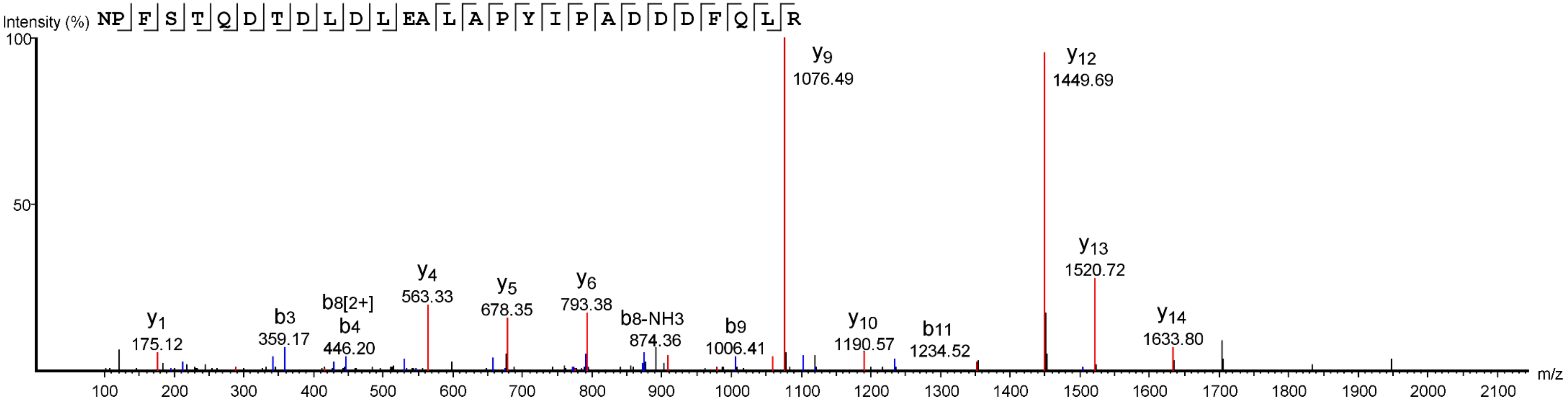

YMT70
Scan #25511

A-056: HIF1A*$_{(548-575)}$: NPFSTQDTDLDLEALAP(+15.99)YIPADDDFQLR

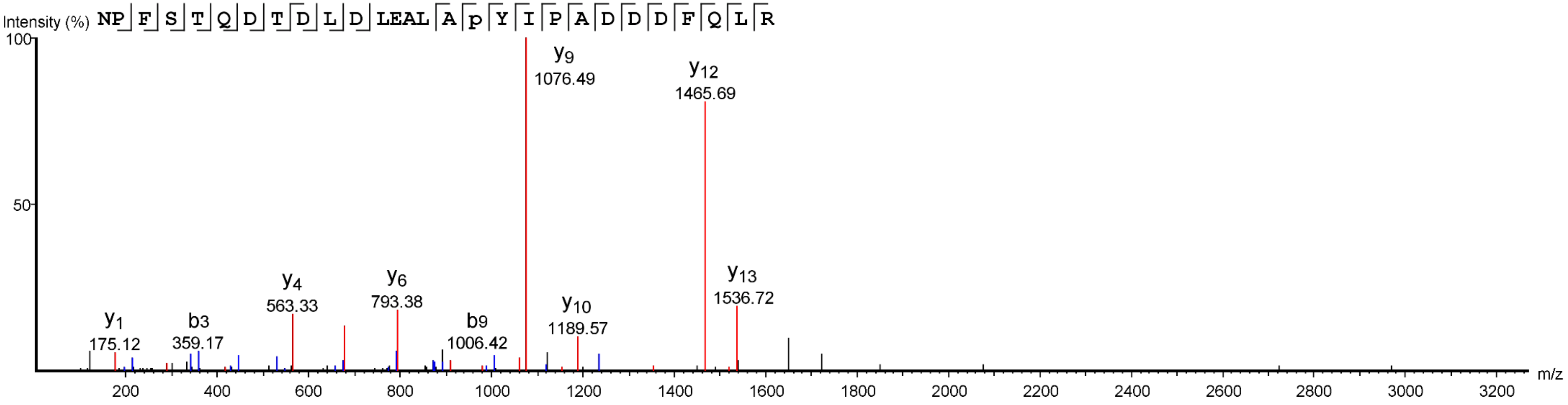

YMT69
Scan #25308

A-057: HIF1A*$_{(548-575)}$: NPFSTQDTDLDLEALAP(+15.99)YIPADDDFQLR

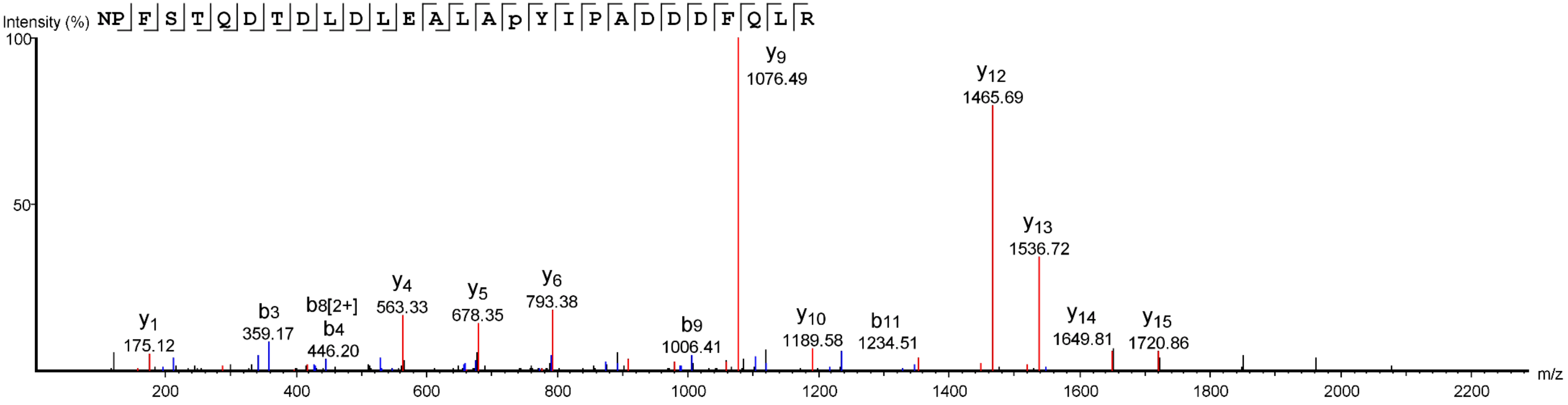

YMT70
Scan #25461

A-058: HIF1A*$_{(392-411)}$: KEPDALTLLAPAAGDTIISL

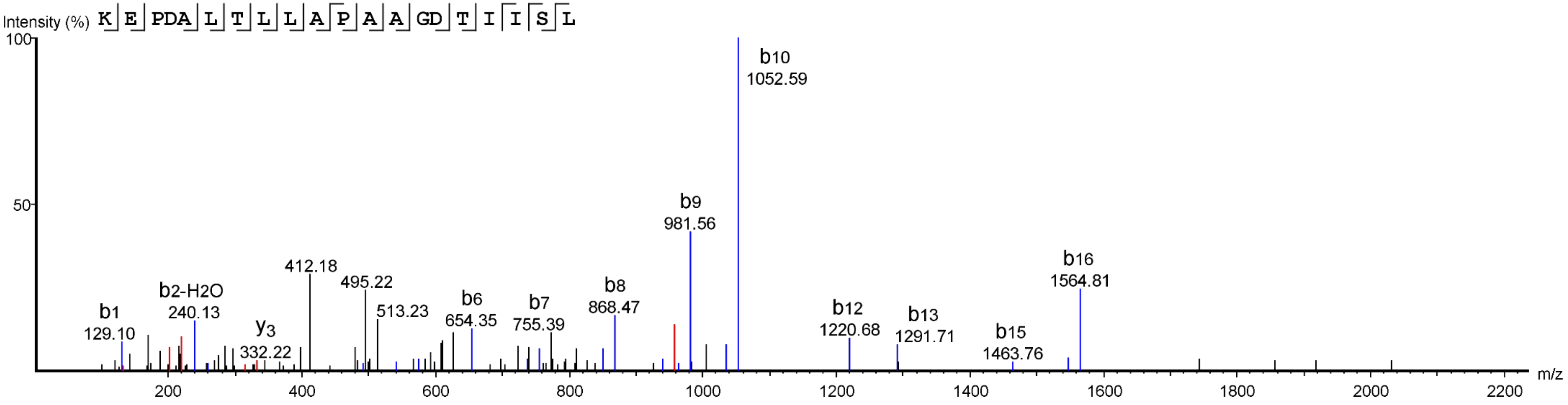

YMT69
Scan #23873

A-059: HIF1A*$_{(392-411)}$: KEPDALTLLAPAAGDTIISL

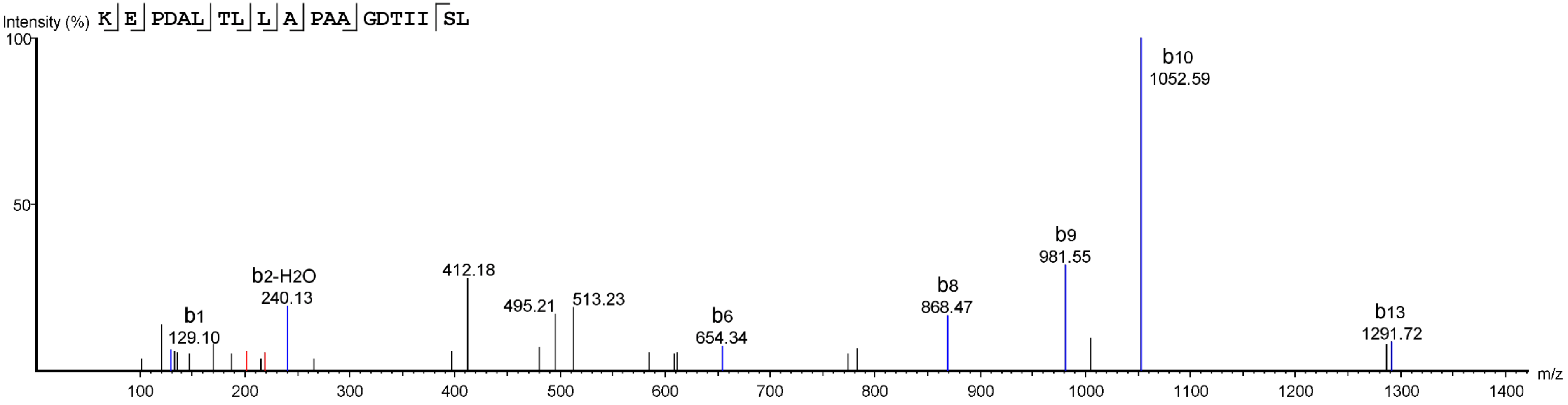

YMT70
Scan #24139

A-060: HIF1A*$_{(392-411)}$: KEPDALTLLAP(+15.99)AAGDTIISL

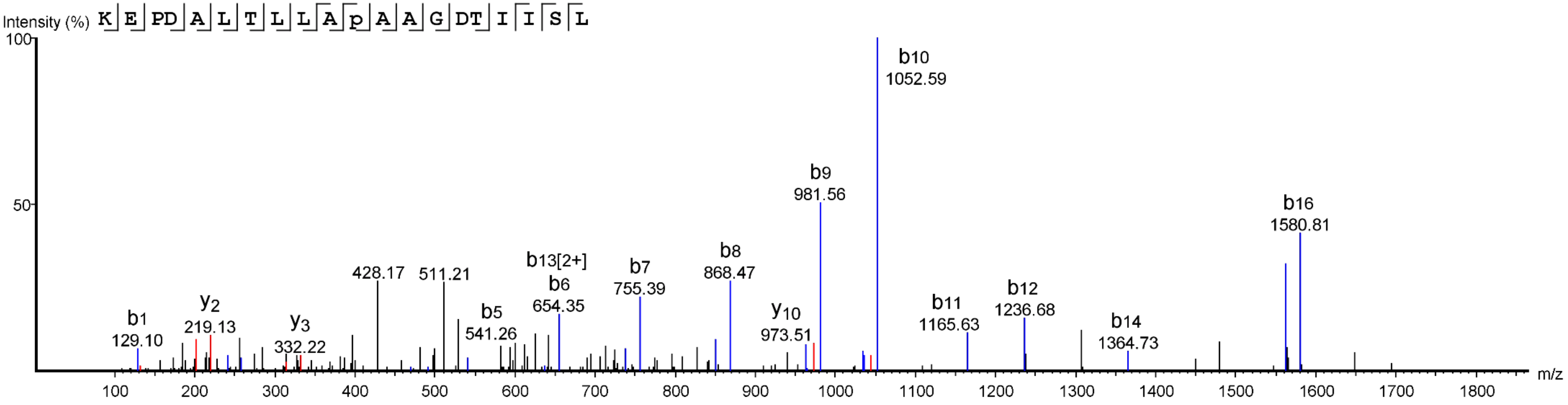

YMT70

Scan #23402

A-061: HIF1A*$_{(548-575)}$: NPFSTQDTDLDLEALAPYIPADDDFQLR

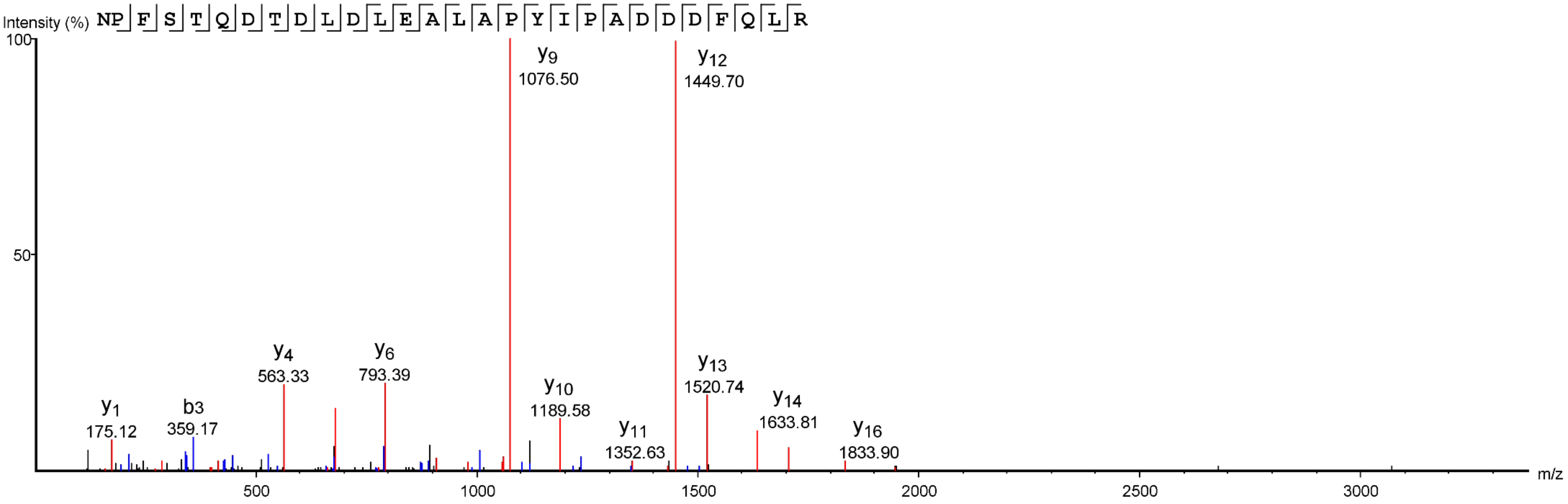

YMT82
Scan #23297

A-062: HIF1A*$_{(548-575)}$: NPFSTQDTDLDLEALAPYIPADDDFQLR

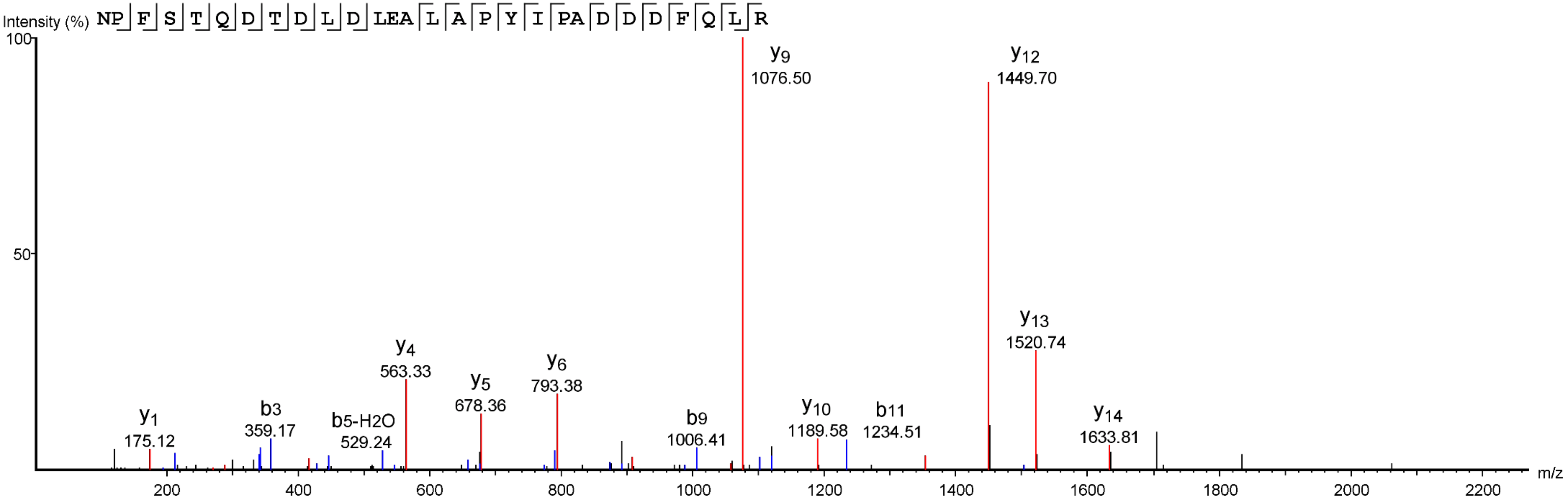

PEP14

Scan #22836

A-063: HIF1A*(548-575): NPFSTQDTDLDLEALAP(+15.99)YIPADDDFQLR

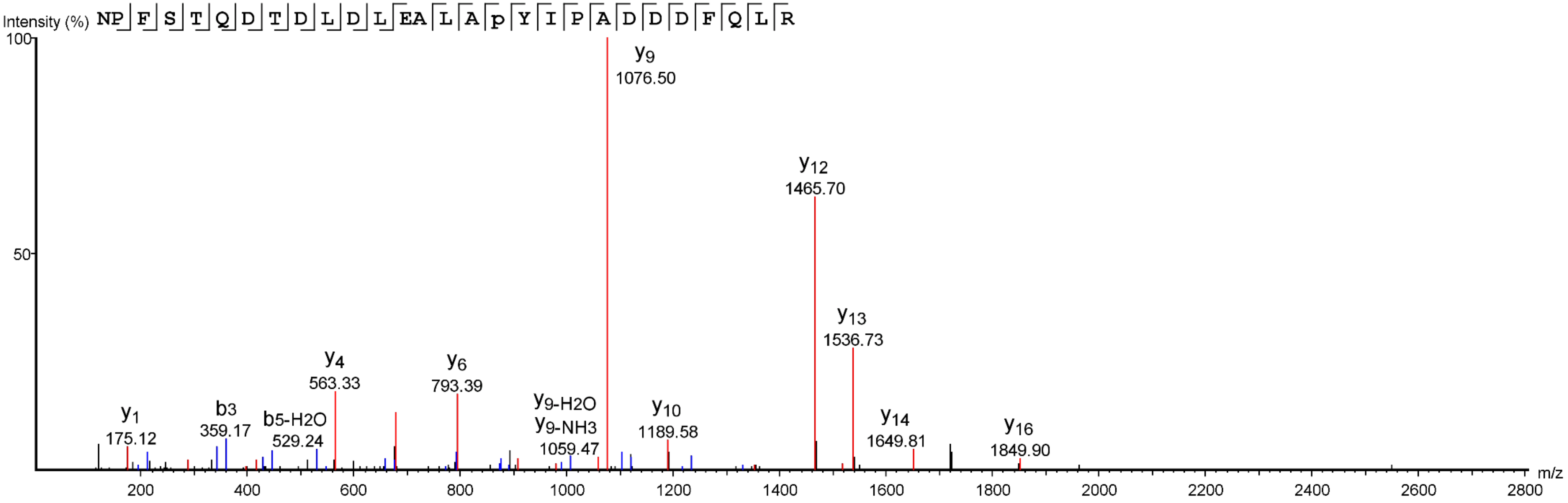

YMT82

Scan #23282

A-064: HIF1A*$_{(548-575)}$: NPFSTQDTDLDLEALAP(+15.99)YIPADDDFQLR

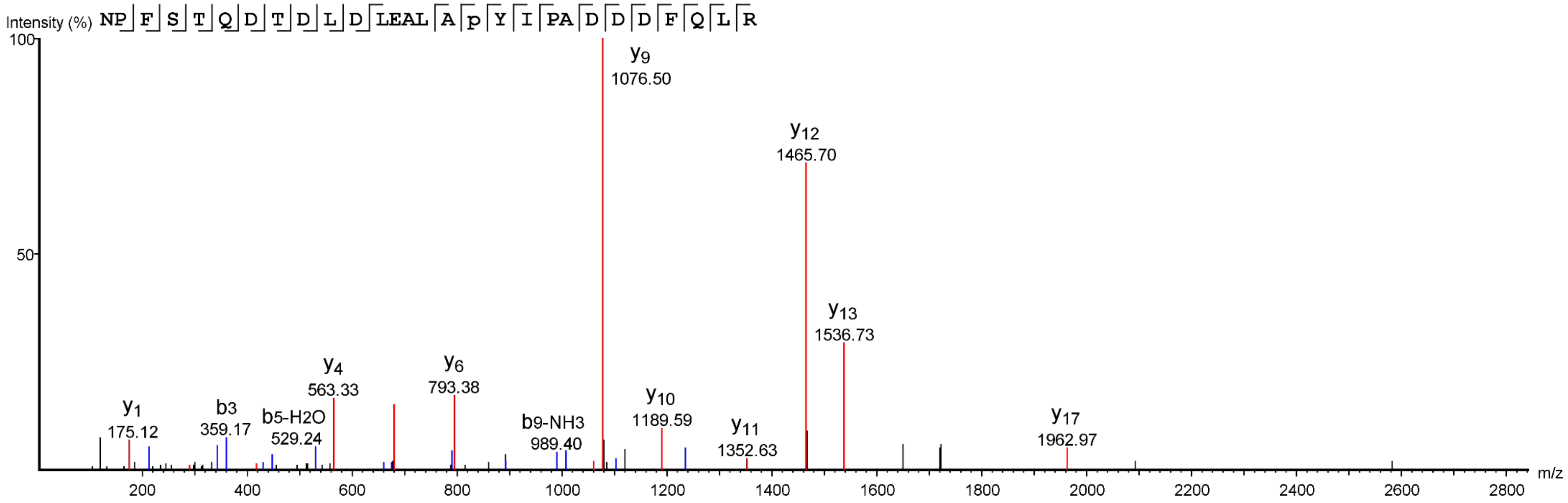

PEP14

Scan #22835

A-065: HIF1A*$_{(392-411)}$: KEPDALTLLAPAAGDTIISL

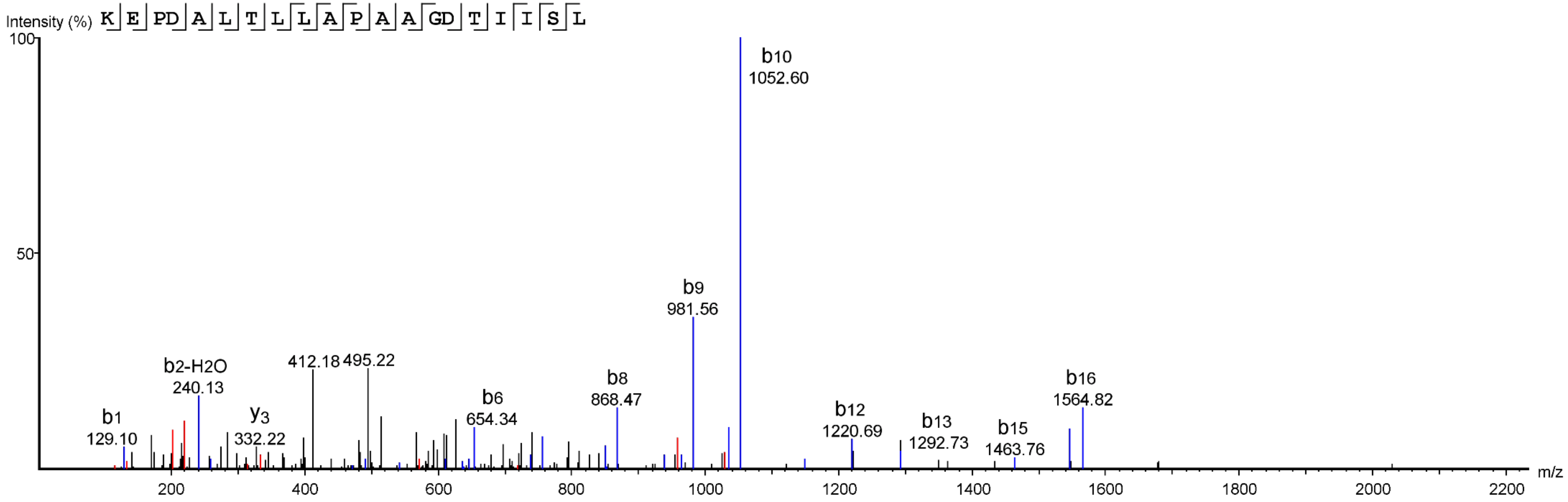

YMT82
Scan #22097

A-066: HIF1A*(392-411): KEPDALTLLAPAAGDTIISL

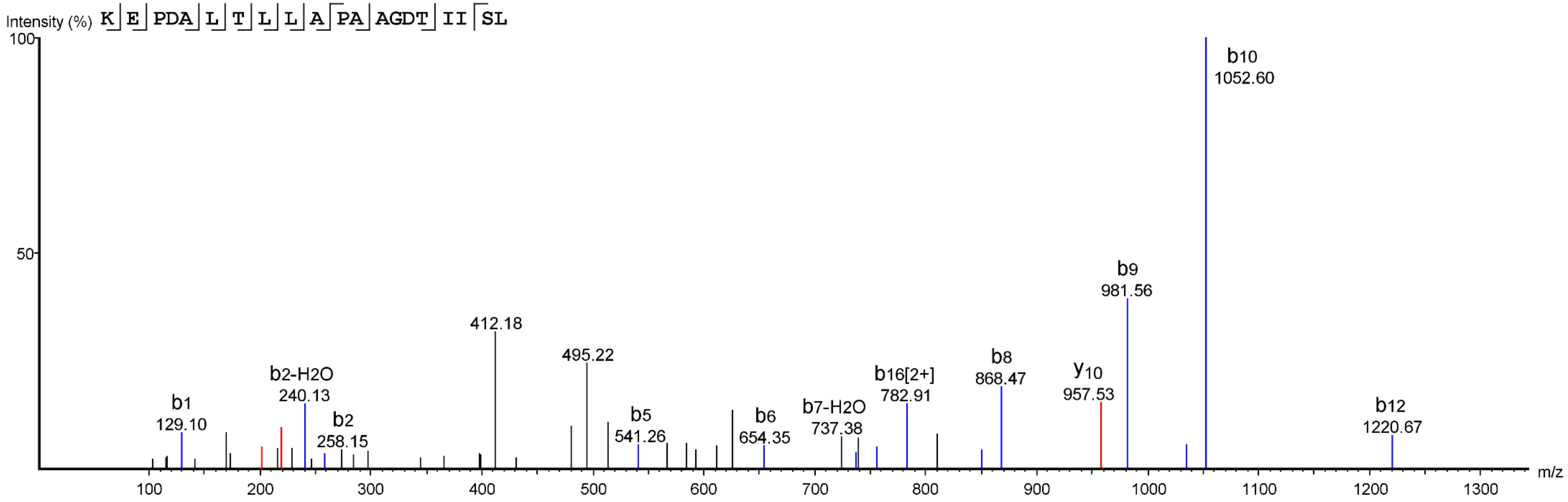

PEP14

Scan #21571

A-067: HIF1A*$_{(392-411)}$: KEPDALTLLAP(+15.99)AAGDTIISL

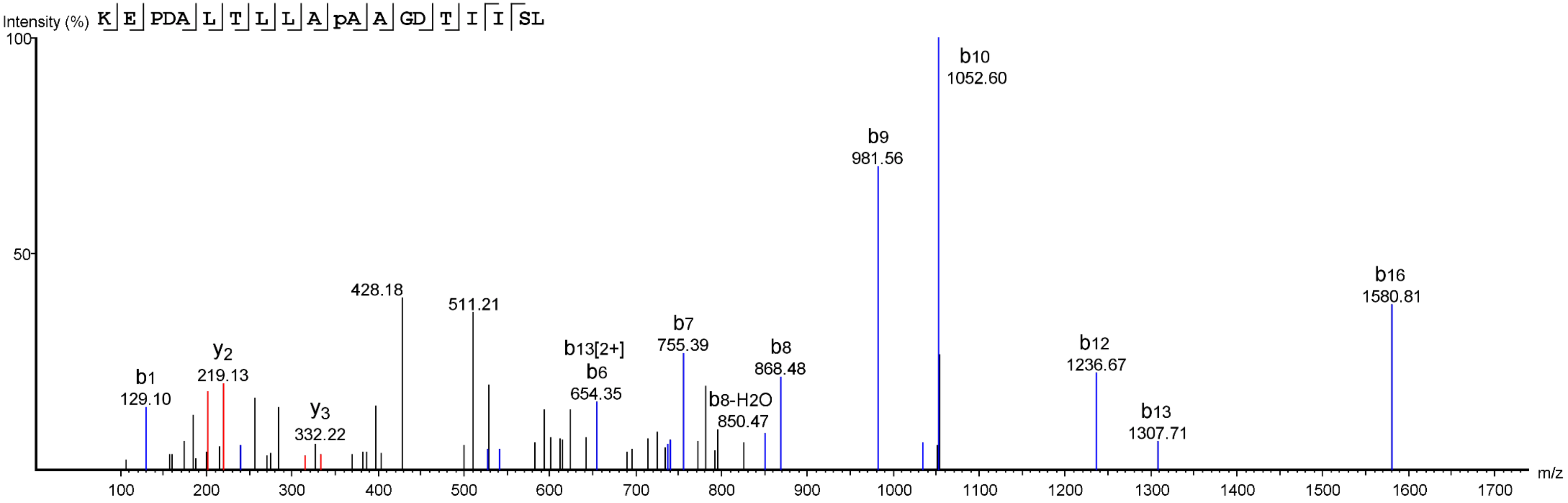

YMT82
Scan #21336

A-068: HIF1A*$_{(392-411)}$: KEPDALTLLAP(+15.99)AAGDTIISL

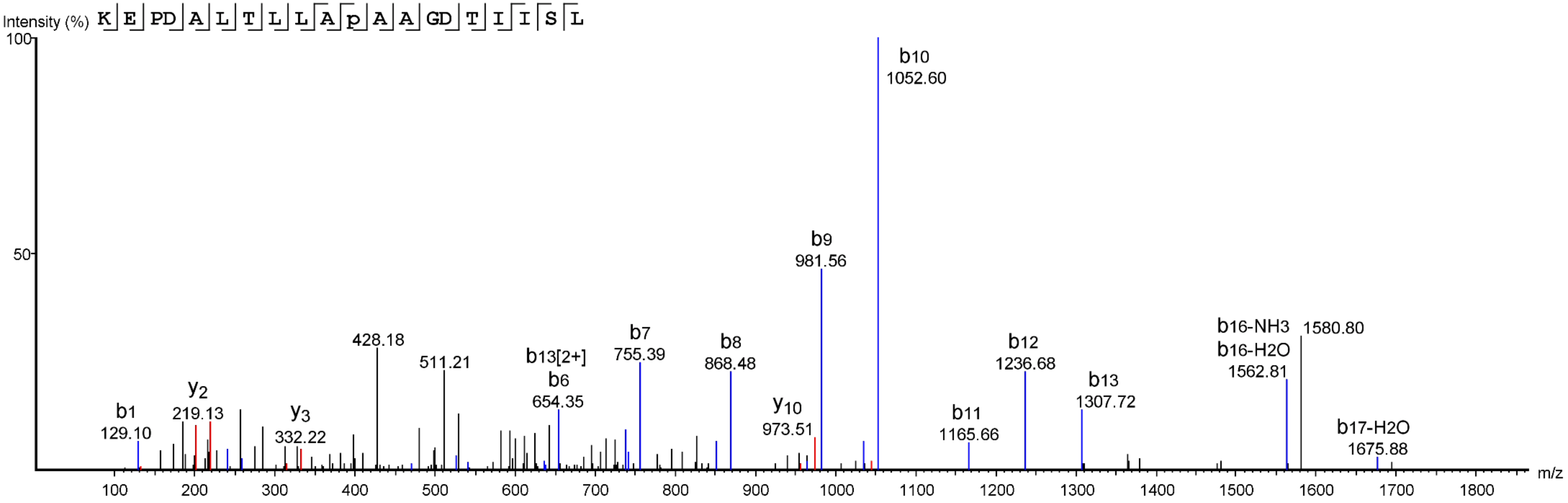

PEP14

Scan #20867

A-069: HIF1A*$_{(548-575)}$: NPFSTQDTDLDLEALAPYIPADDDFQLR

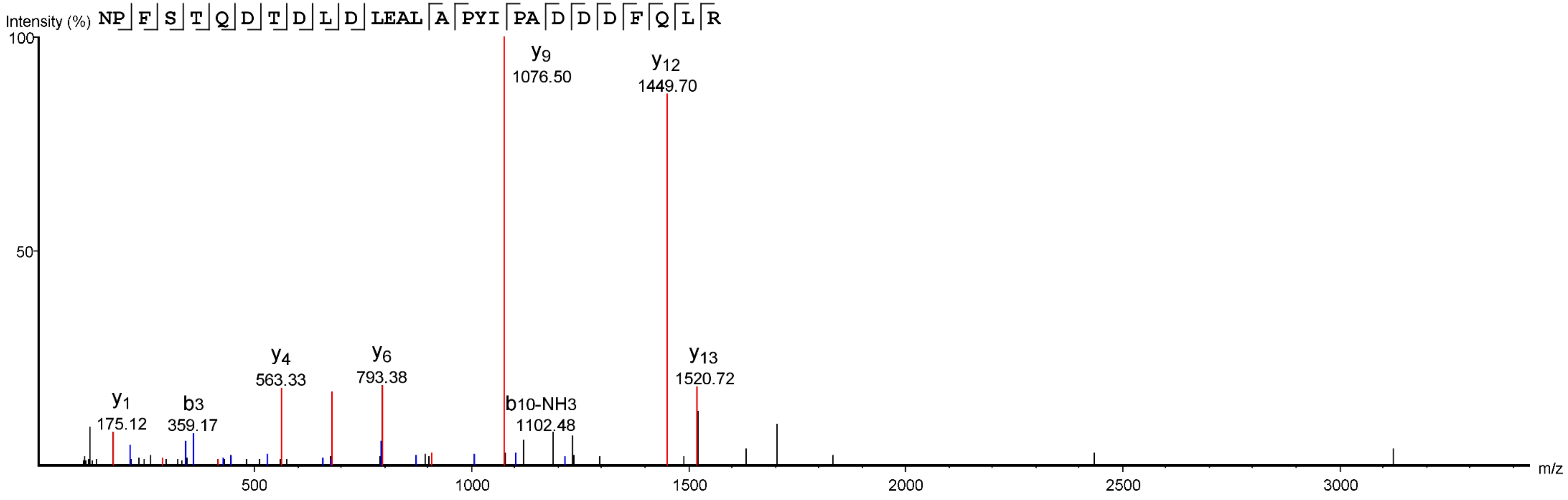

YMT83
Scan #27832

A-070: HIF1A*$_{(548-575)}$: NPFSTQDTDLDLEALAPYIPADDDFQLR

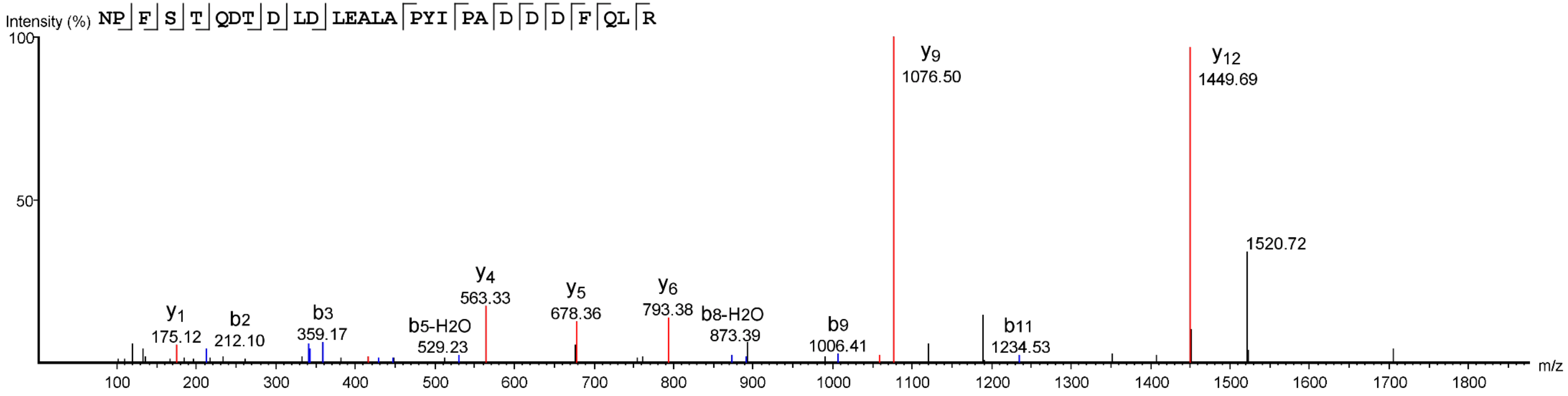

YMT84

Scan #26593

A-071: HIF1A*$_{(548\text{-}575)}$: NPFSTQDTDLDLEALAP(+15.99)YIPADDDFQLR

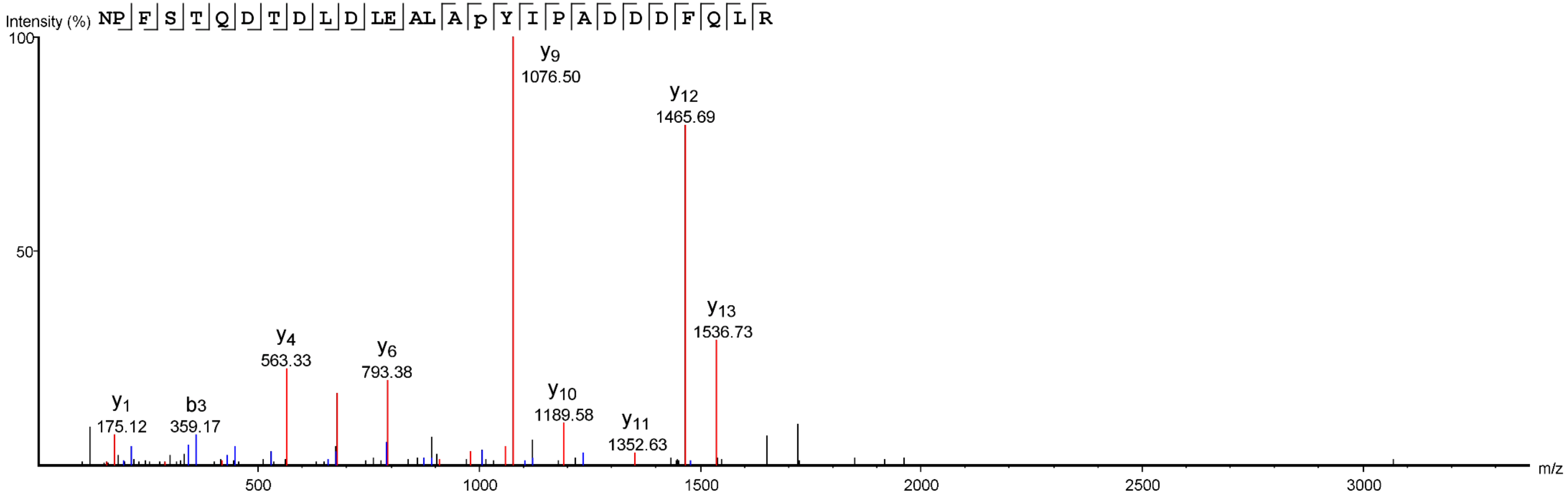

YMT83

Scan #27769

A-072: HIF1A*$_{(548-575)}$: NPFSTQDTDLDLEALAP(+15.99)YIPADDDFQLR

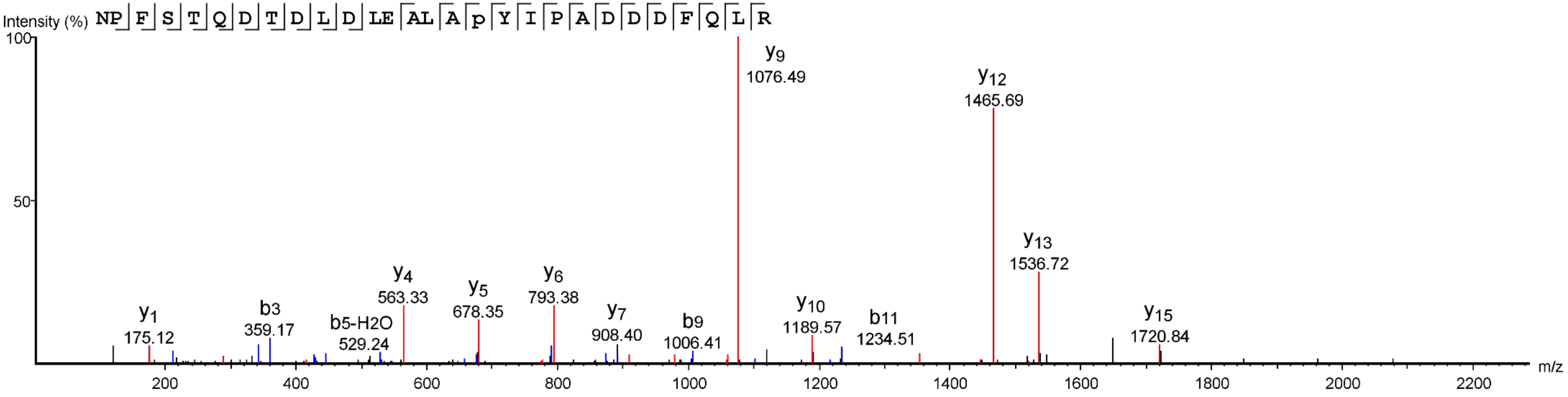

YMT84
Scan #26544

A-073: HIF1A*$_{(392-403)}$: KEPDALTLLAPA

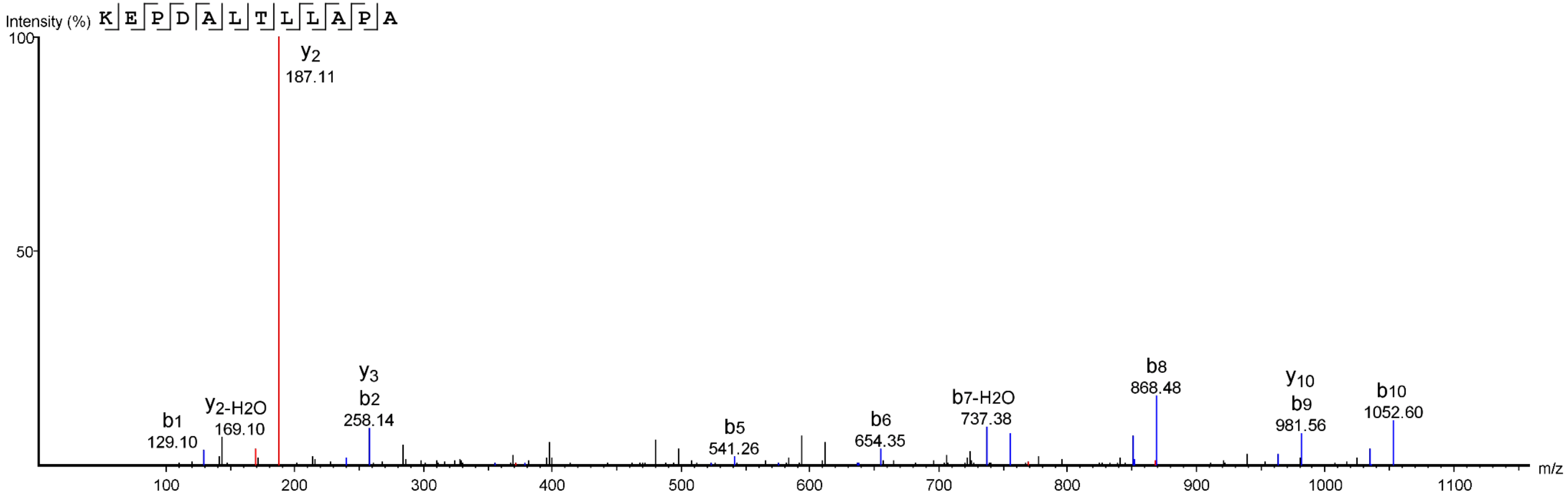

YMT83
Scan #19706

A-074: HIF1A*$_{(392-403)}$: KEPDALTLLAPA

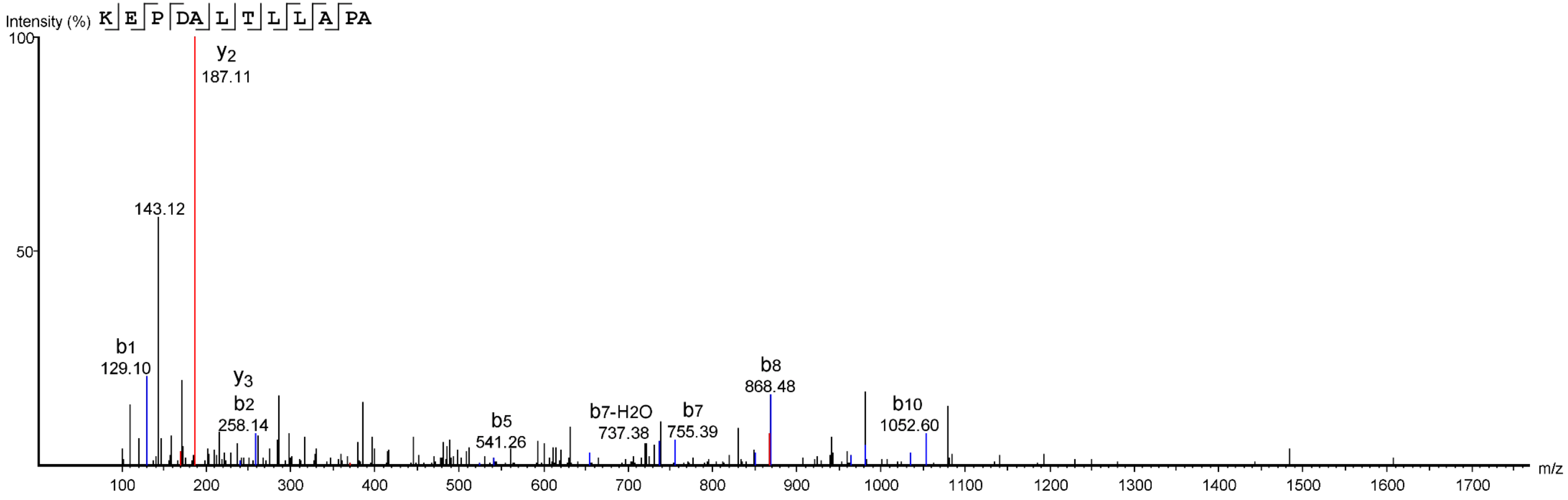

YMT84
Scan #18811

A-075: HIF1A*$_{(392-403)}$: KEPDALTLLAP(+15.99)A

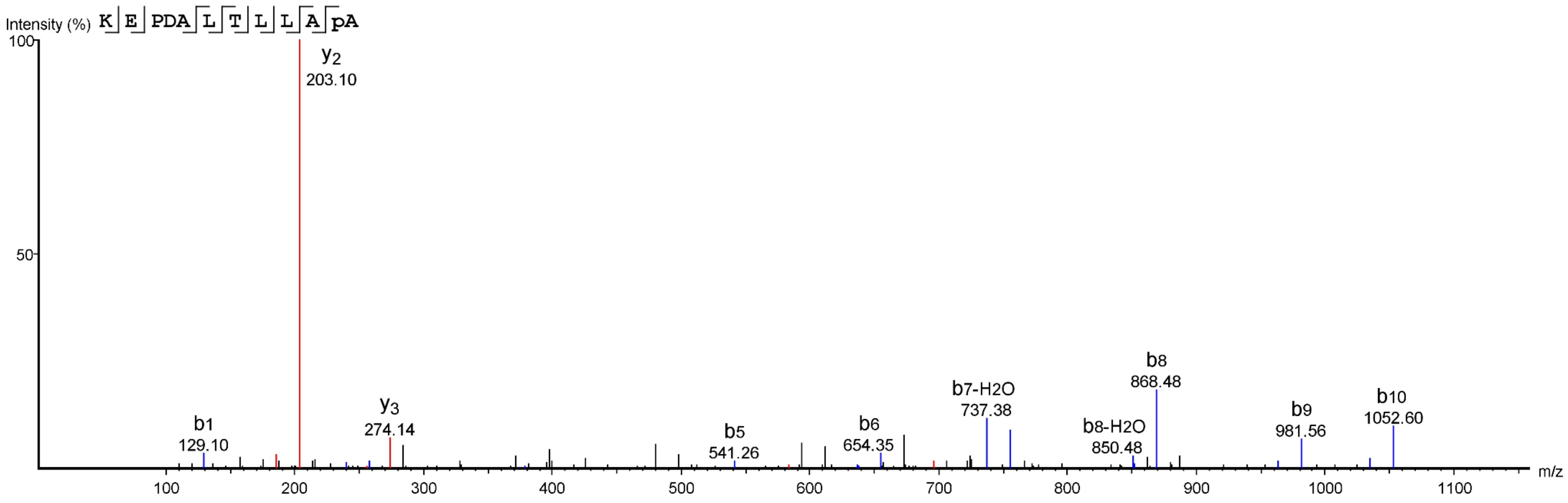

YMT83
Scan #18125

A-076: HIF1A*$_{(392-403)}$: KEPDALTLLAP(+15.99)A

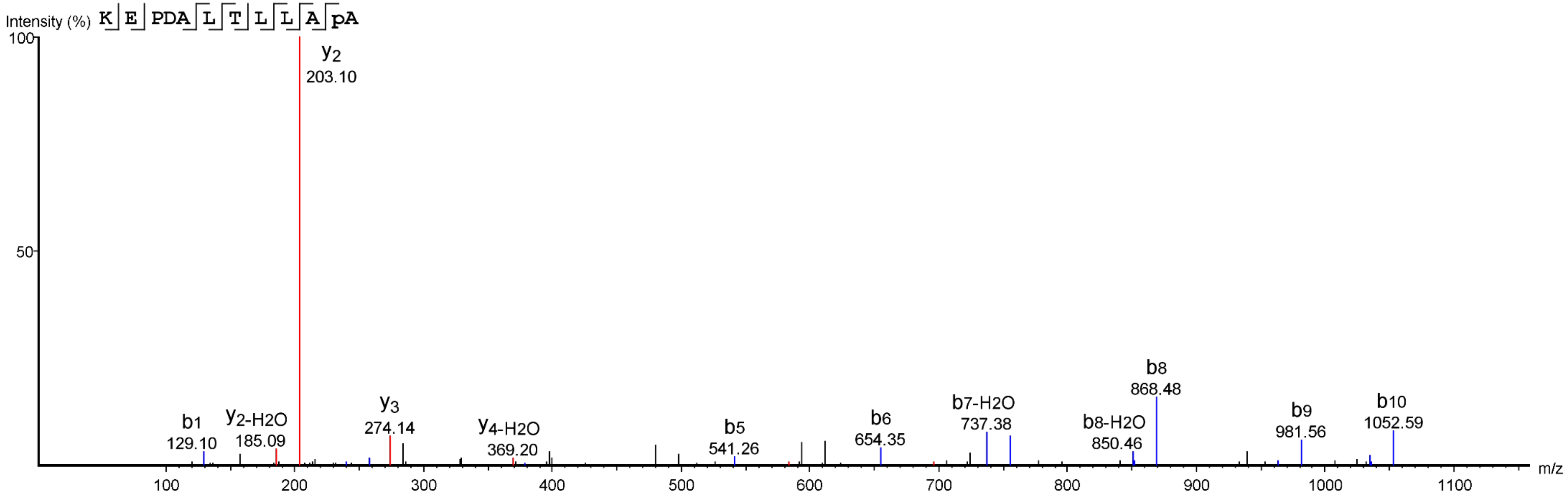

YMT84
Scan #17426

A-077: HIF1A*$_{(548-575)}$: NPFSTQDTDLDLEALAPYIPADDDFQLR

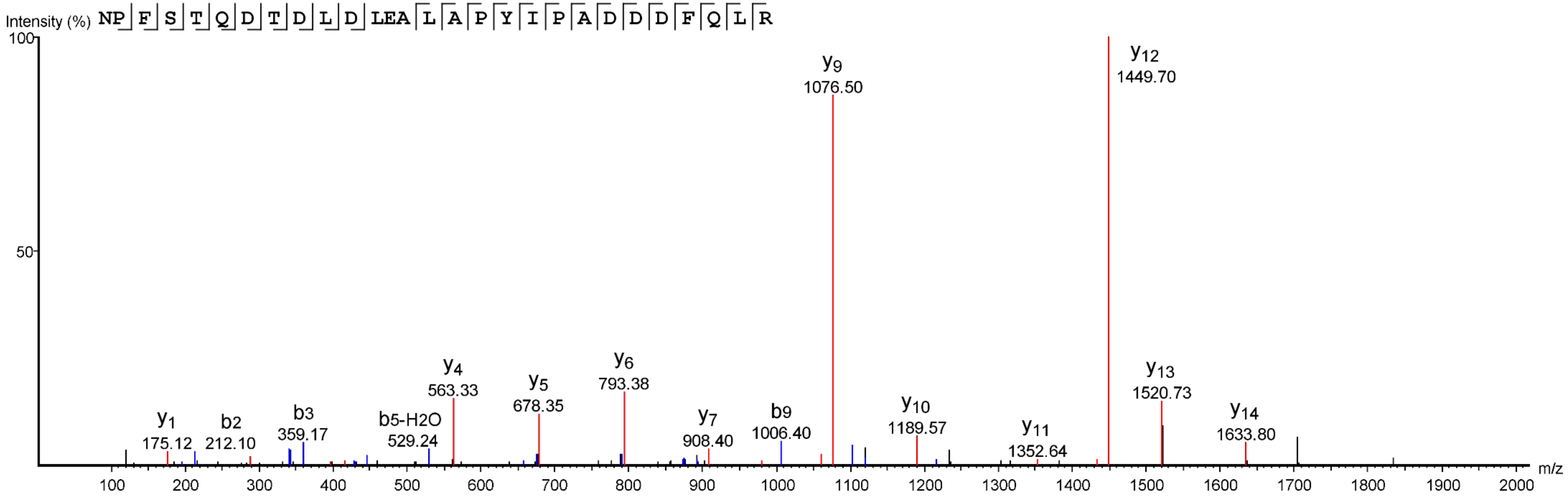

YMT86
Scan #28863

A-078: HIF1A*$_{(548-575)}$: NPFSTQDTDLDLEALAPYIPADDDFQLR

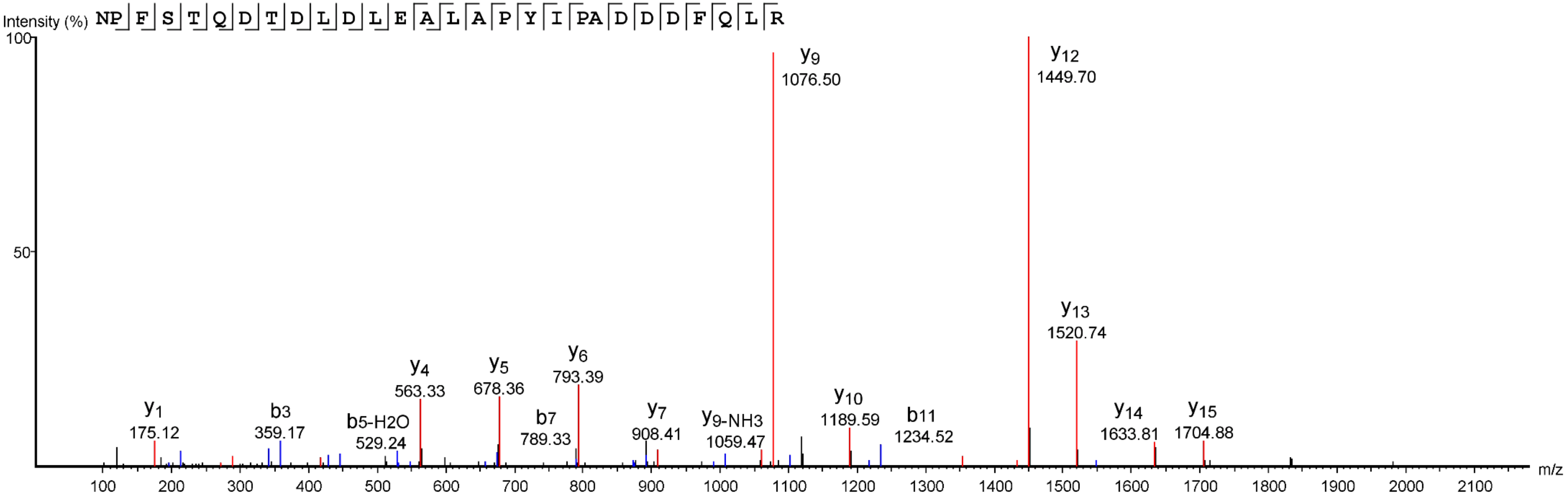

YMT88

Scan #27079

A-079: HIF1A*(548-575): NPFSTQDTDLDLEALAP(+15.99)YIPADDDFQLR

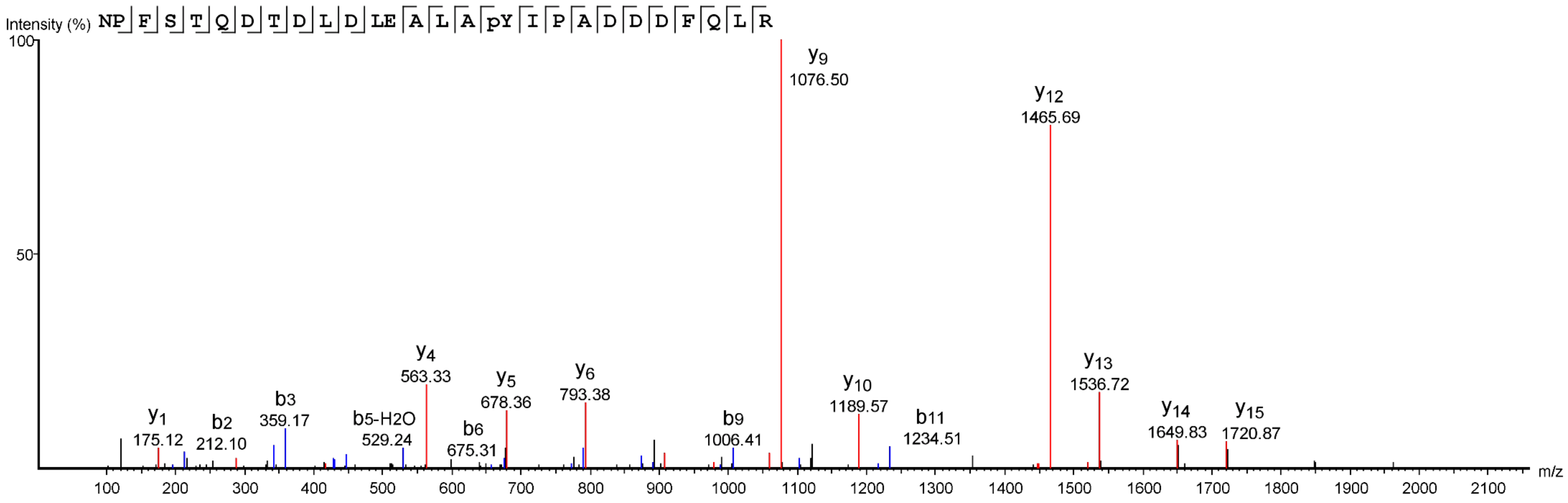

YMT86
Scan #28815

A-080: HIF1A*$_{(548-575)}$: NPFSTQDTDLDLEALAP(+15.99)YIPADDDFQLR

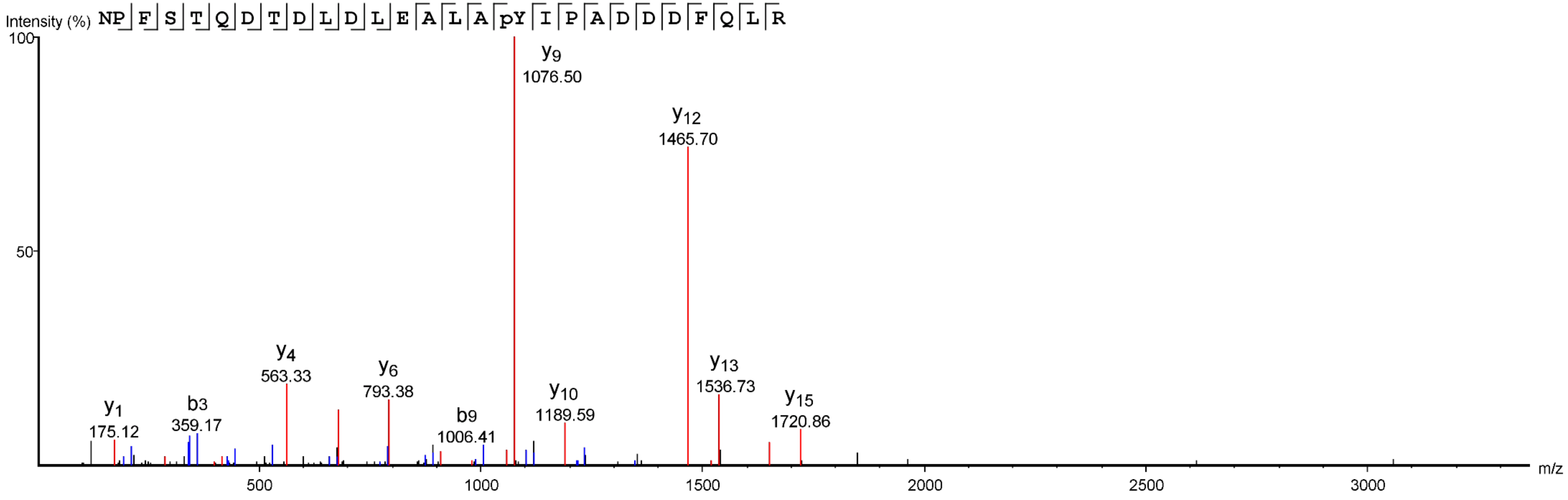

YMT88

Scan #27030

A-081: HIF1A*$_{(392-403)}$: KEPDALTLLAPA

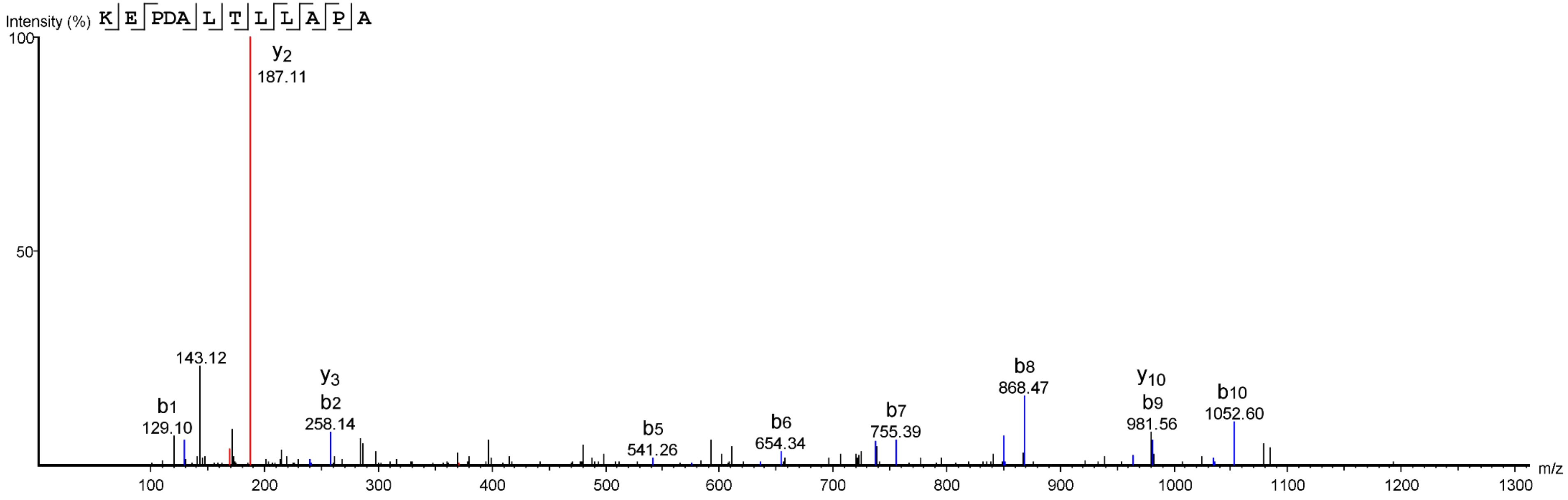

YMT86
Scan #20659

A-082: HIF1A*$_{(392-403)}$: KEPDALTLLAPA

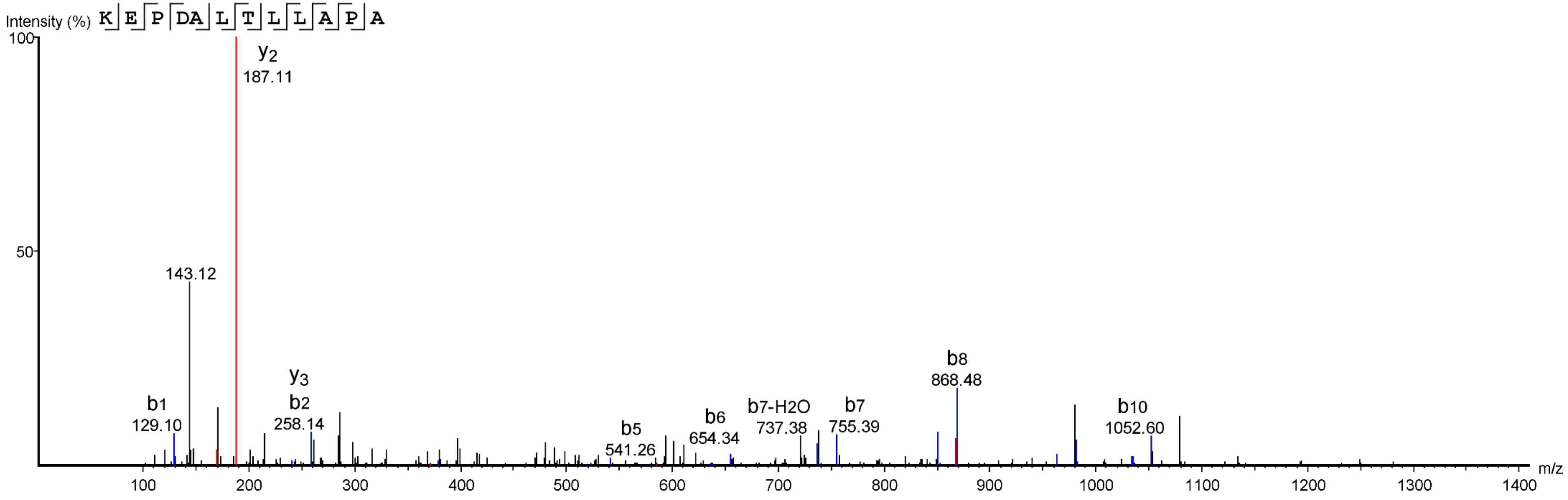

YMT88

Scan #19243

A-083: HIF1A*$_{(392-403)}$: KEPDALTLLAP(+15.99)A

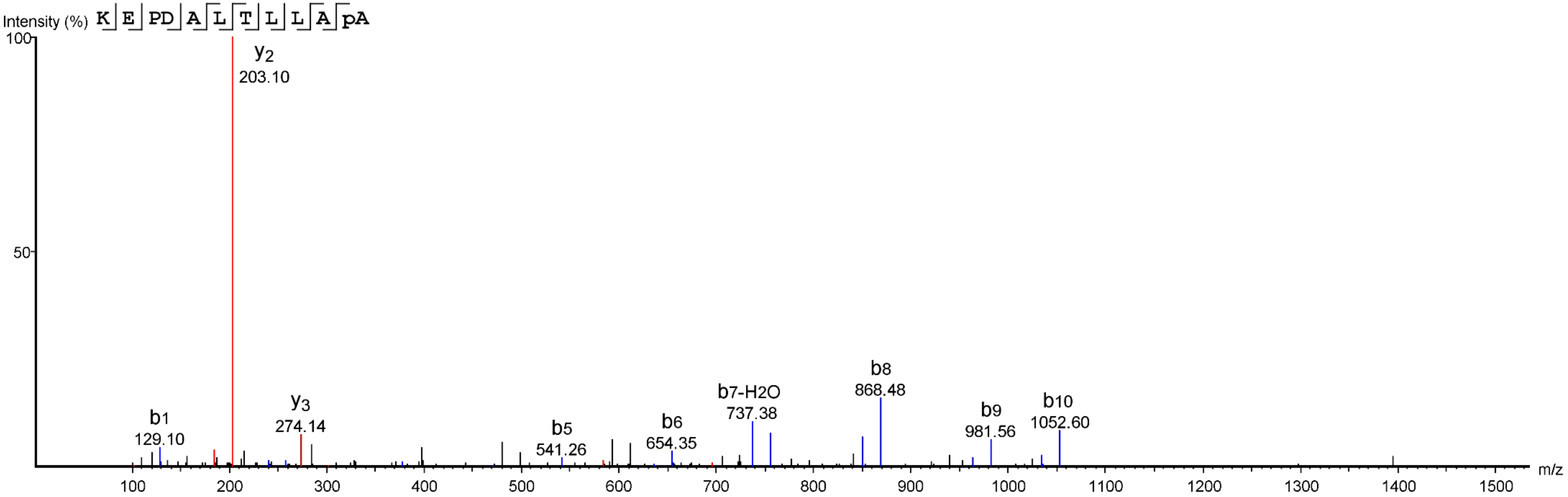

YMT86
Scan #19065

A-084: HIF1A*$_{(392-403)}$: KEPDALTLLAP(+15.99)A

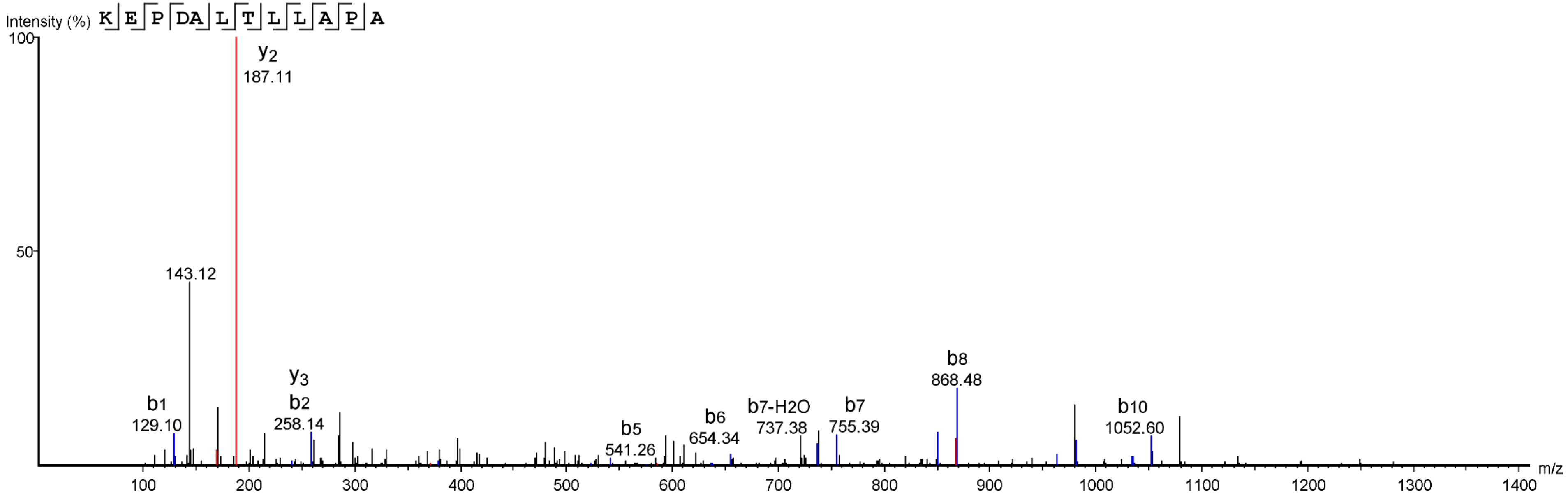

YMT88

Scan #17777

A-085: HIF1A*(548-575): NPFSTQDTDLDLEALAPYIPADDDFQLR

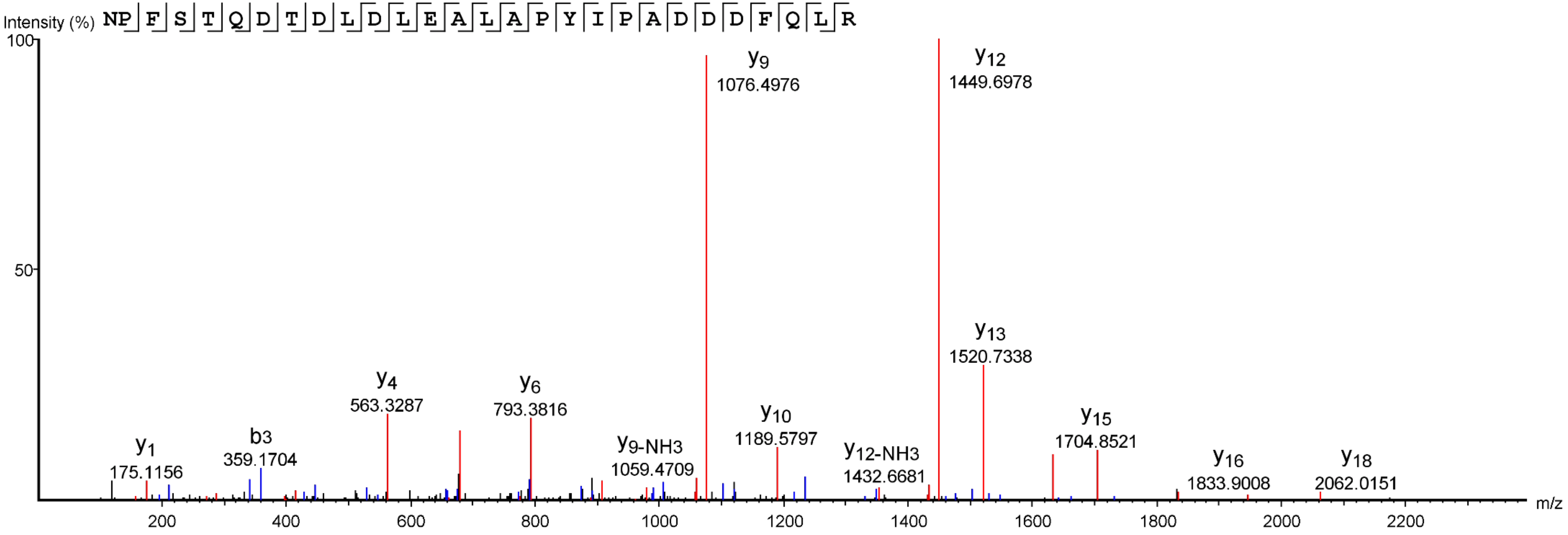

YMT109
Scan #27280

A-086: HIF1A*$_{(548-575)}$: NPFSTQDTDLDLEALAPYIPADDDFQLR

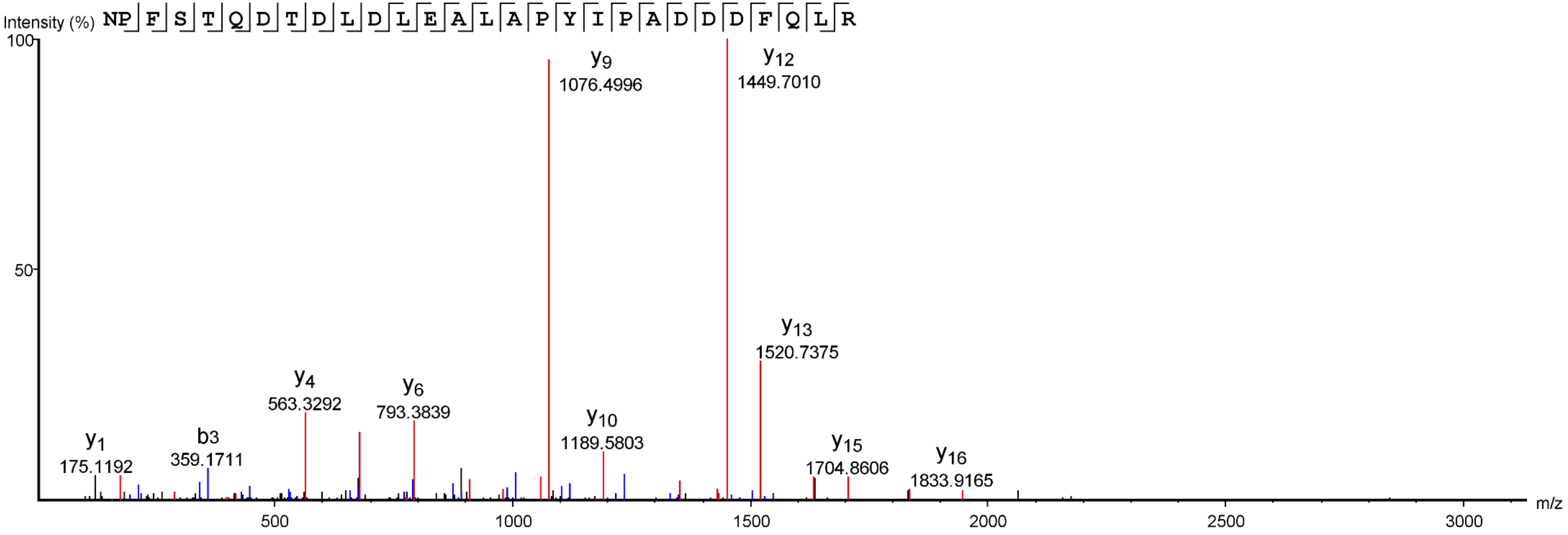

YMT110
Scan #27304

A-087: HIF1A*$_{(548-575)}$: NPFSTQDTDLDLEALAP(+15.99)YIPADDDFQLR

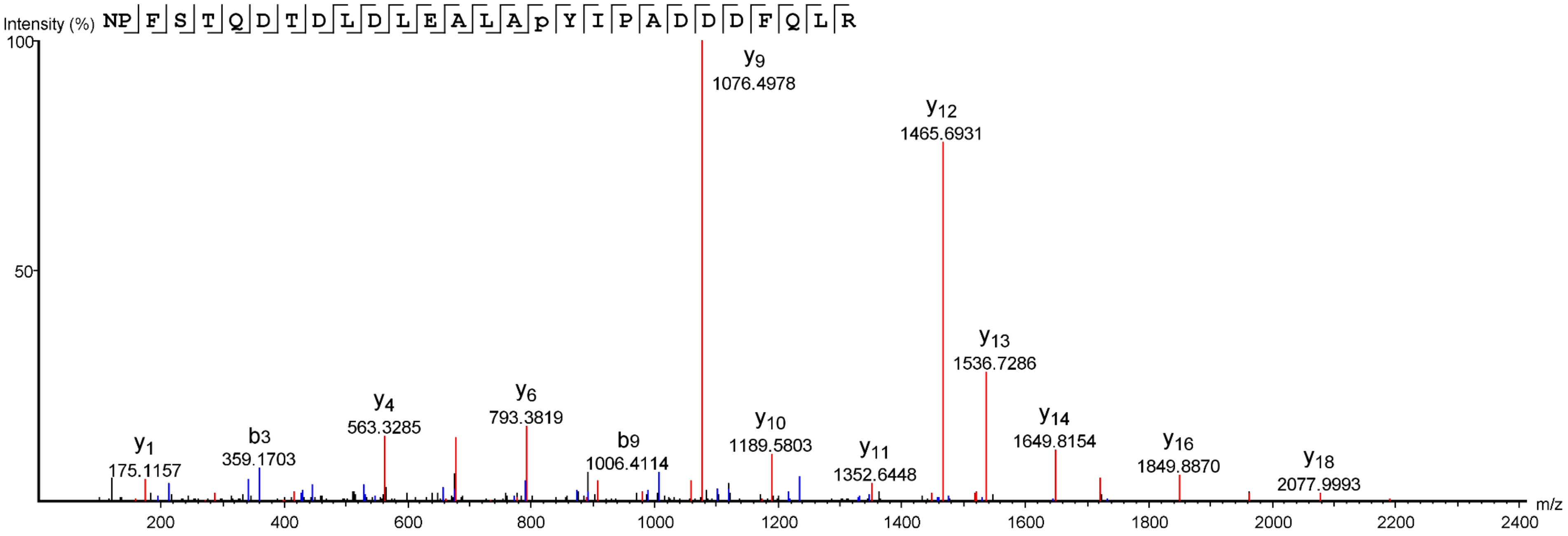

YMT109
Scan #27248

A-088: HIF1A*$_{(548-575)}$: NPFSTQDTDLDLEALAP(+15.99)YIPADDDFQLR

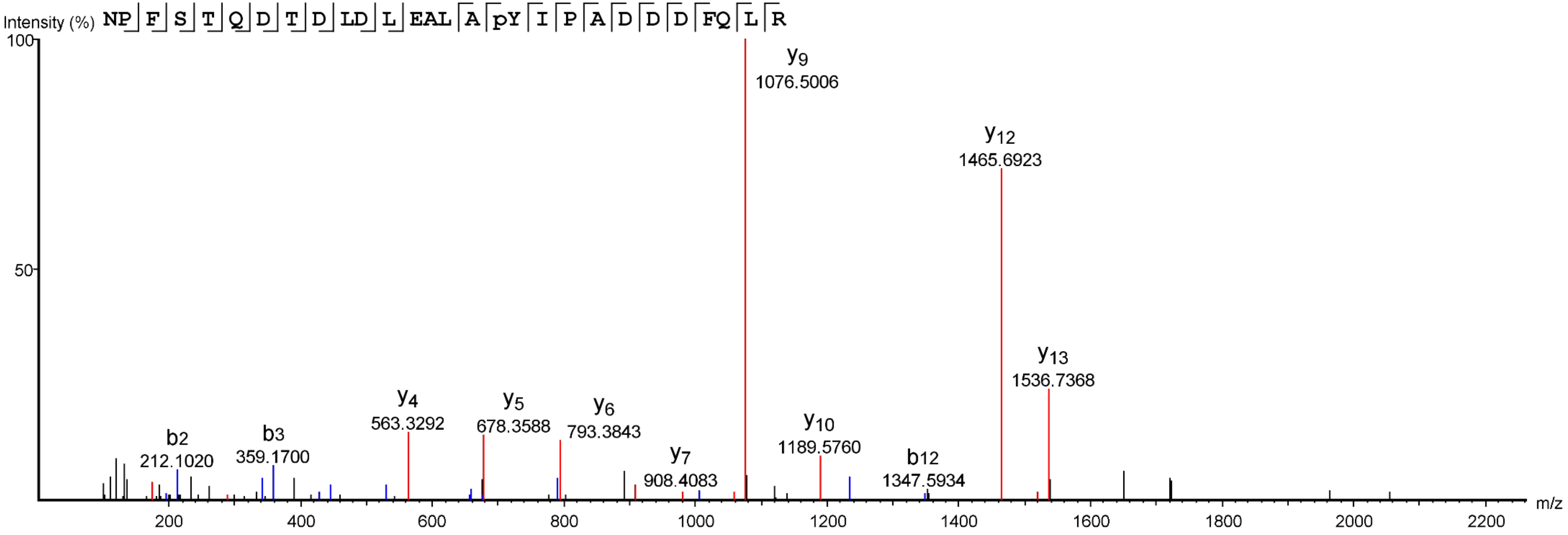

YMT110
Scan #27354

A-089: HIF1A*(392-411): KEPDALTLLAPAAGDTIISL

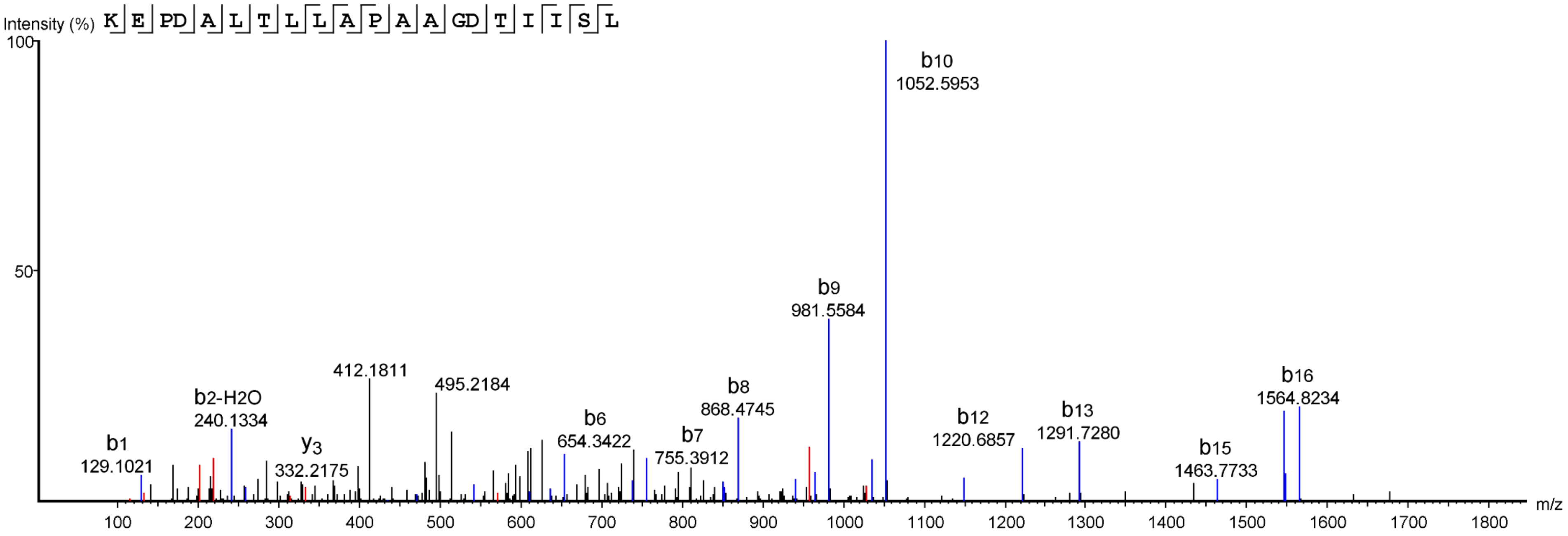

YMT109
Scan #26030

A-090: HIF1A*$_{(392-411)}$: KEPDALTLLAPAAGDTIISL

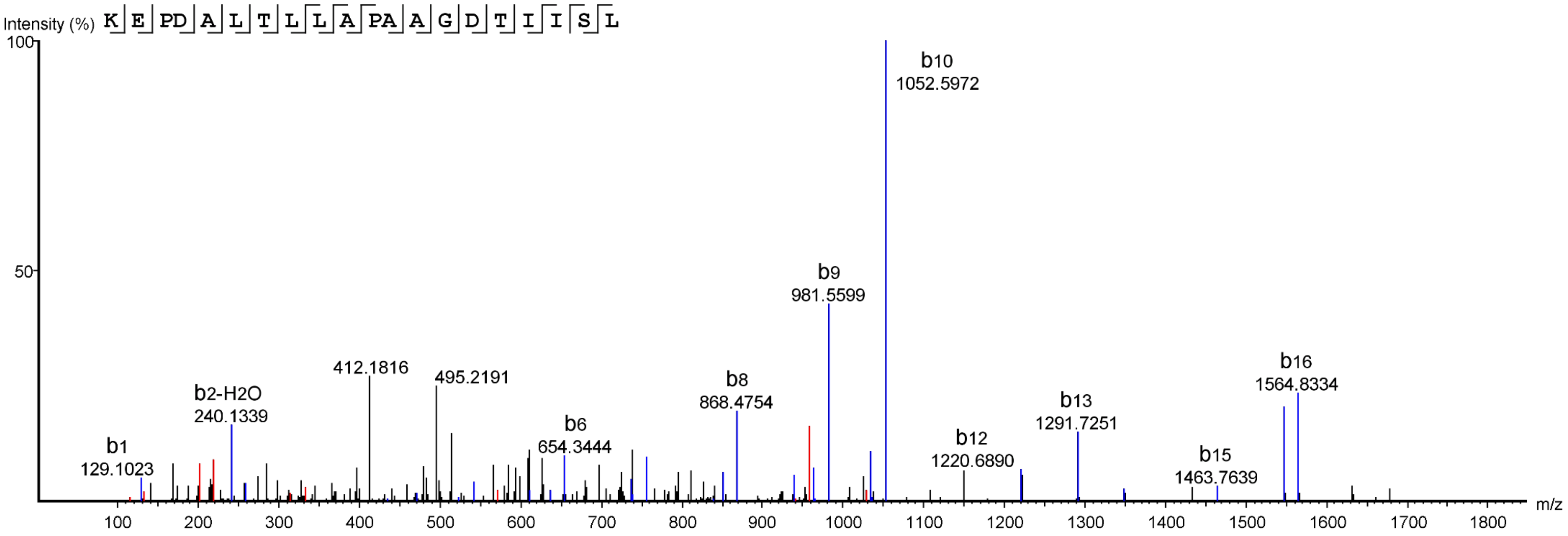

YMT110
Scan #26036

A-092: HIF1A*(548-575): NPFSTQDTDLDLEALAPYIPADDDFQLR

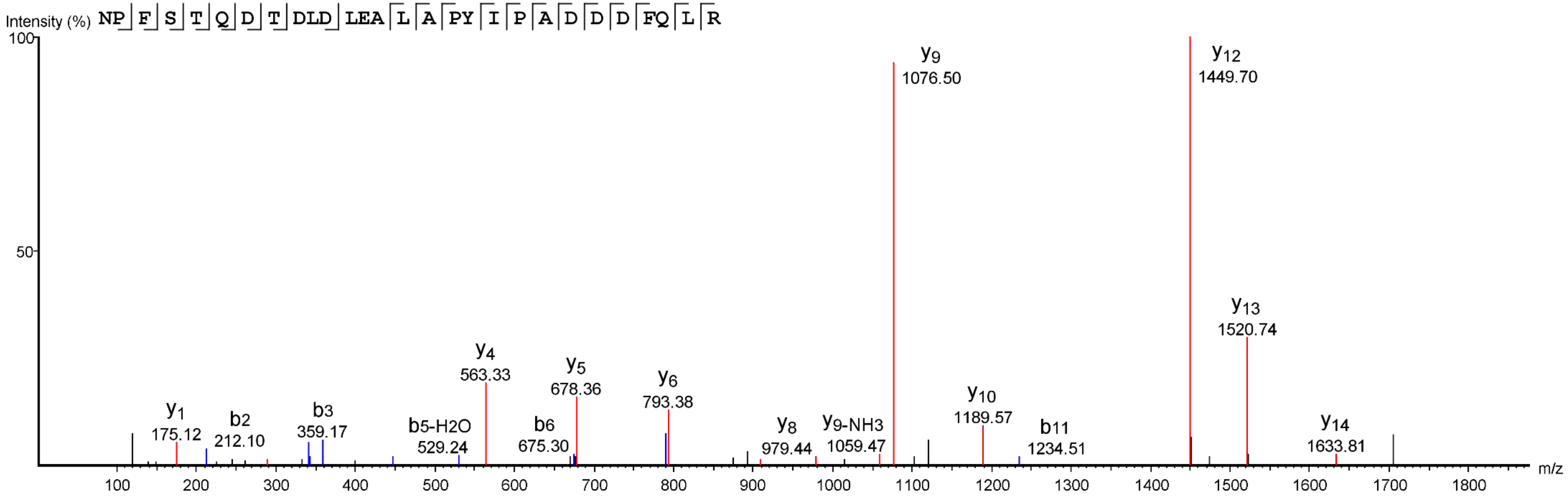

YMT93
Scan #26868

A-093: HIF1A*$_{(548-575)}$: NPFSTQDTDLDLEALAPYIPADDDFQLR

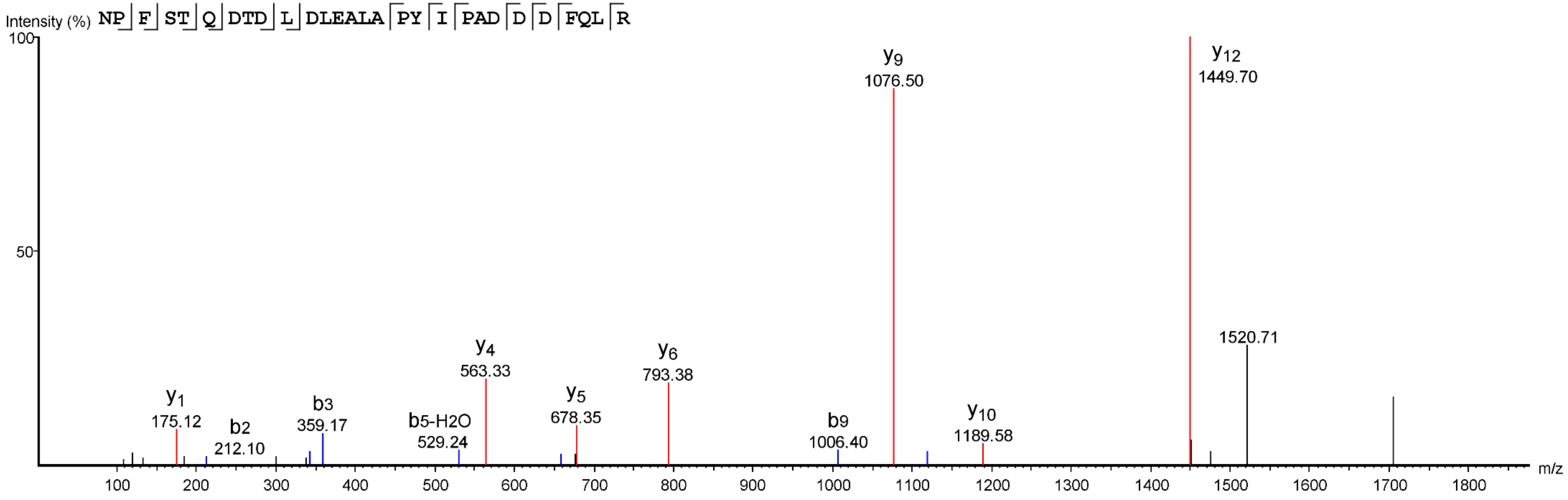

YMT94
Scan #26755

A-094: HIF1A*$_{(548-575)}$: NPFSTQDTDLDLEALAPYIPADDDFQLR

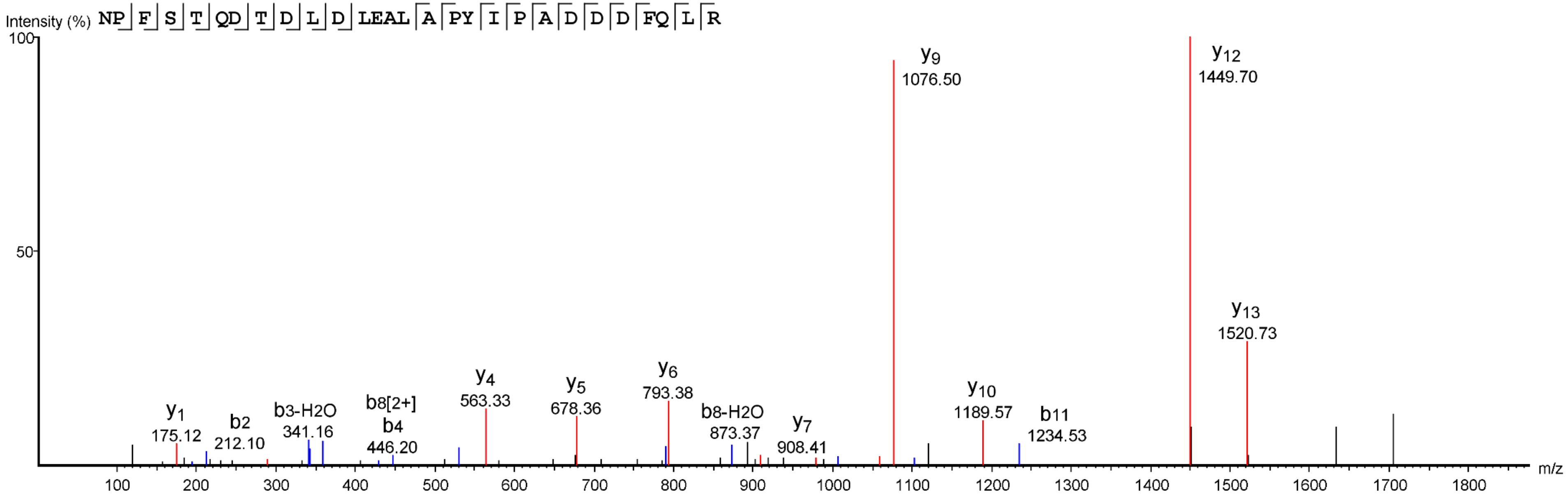

YMT95
Scan #27381

A-095: HIF1A*$_{(548-575)}$: NPFSTQDTDLDLEALAP(+15.99)YIPADDDFQLR

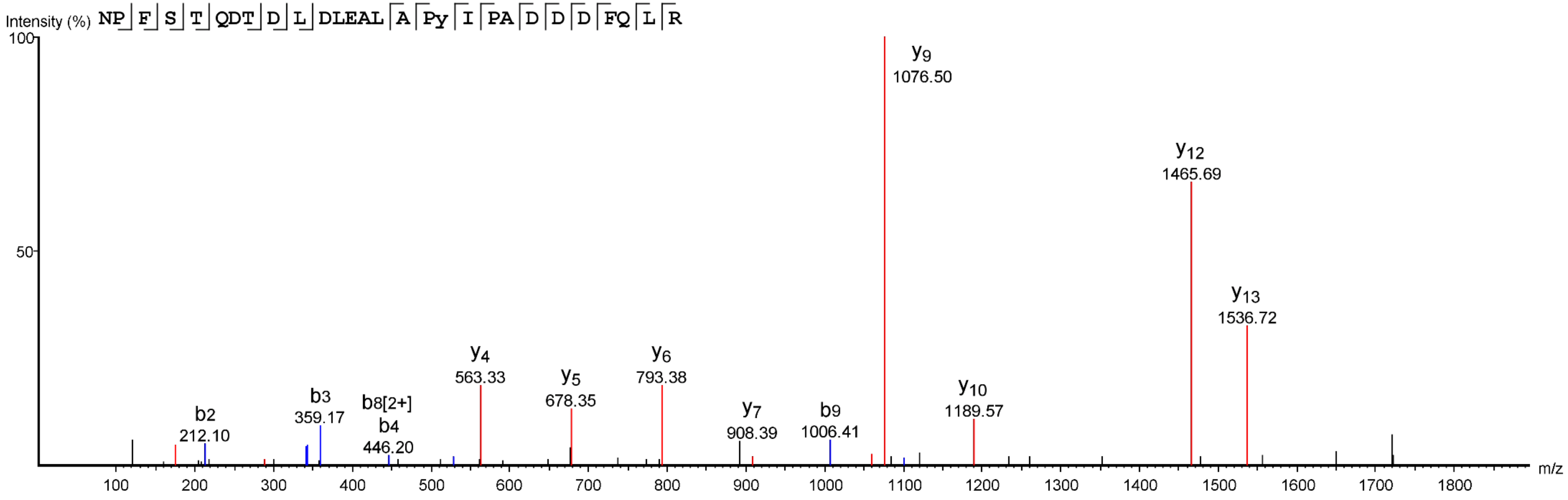

YMT93
Scan #26836

A-096: HIF1A*$_{(548-575)}$: NPFSTQDTDLDLEALAP(+15.99)YIPADDDFQLR

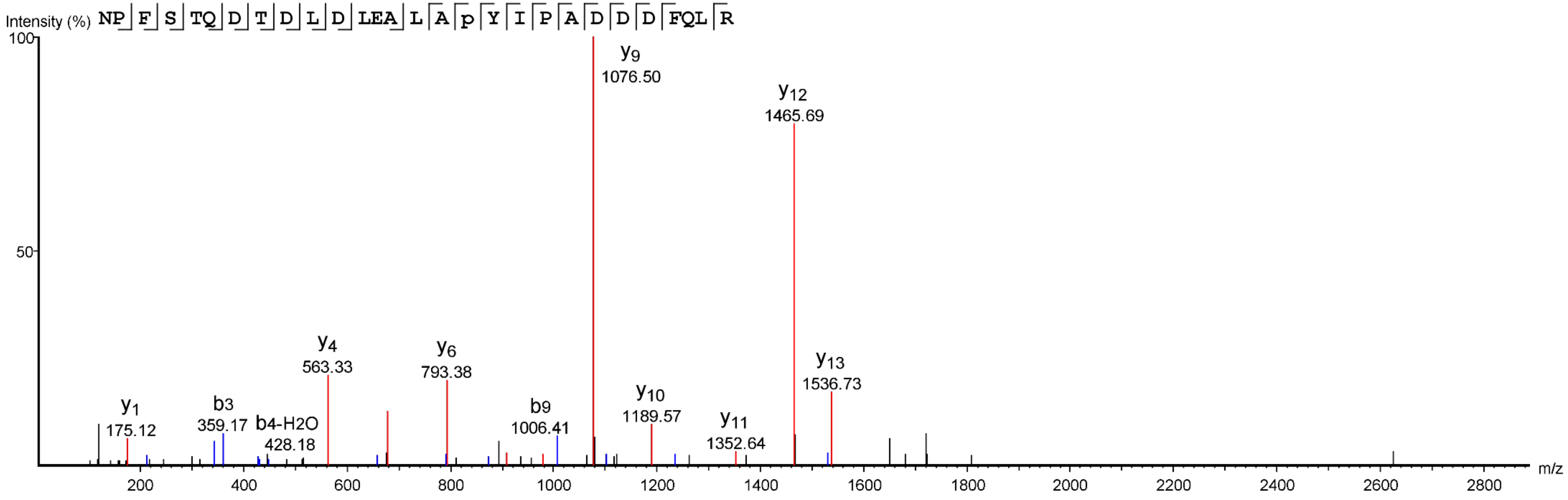

YMT94
Scan #26629

A-097: HIF1A*$_{(548-575)}$: NPFSTQDTDLDLEALAP(+15.99)YIPADDDFQLR

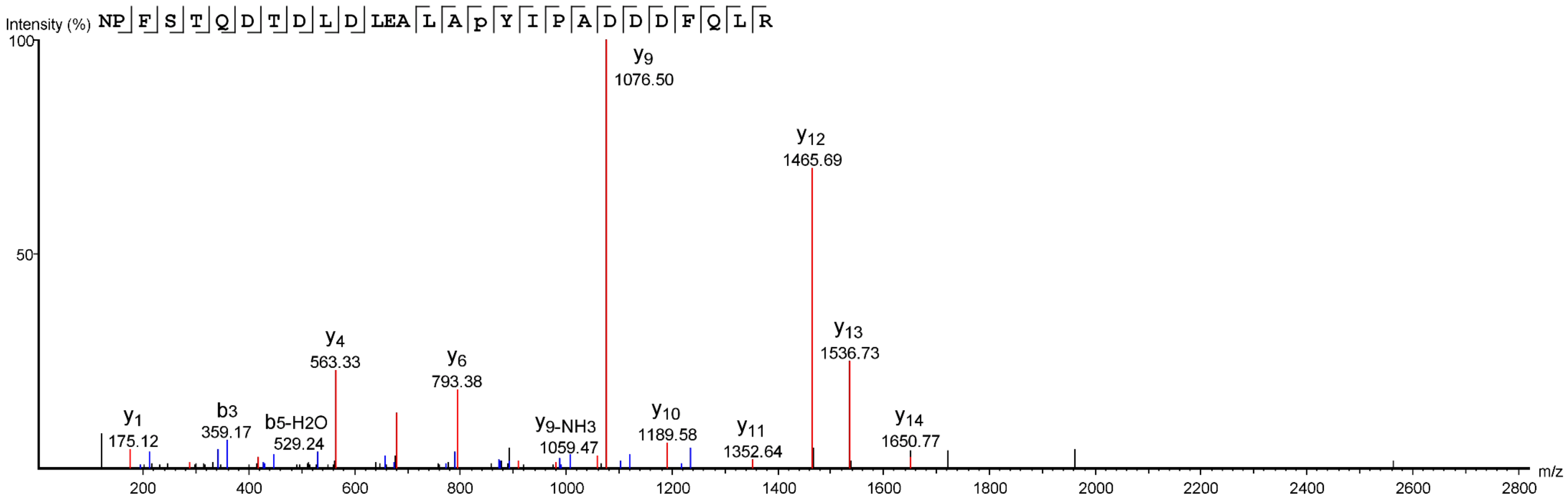

YMT95
Scan #27317

A-098: HIF1A*$_{(392-403)}$: KEPDALTLLAPA

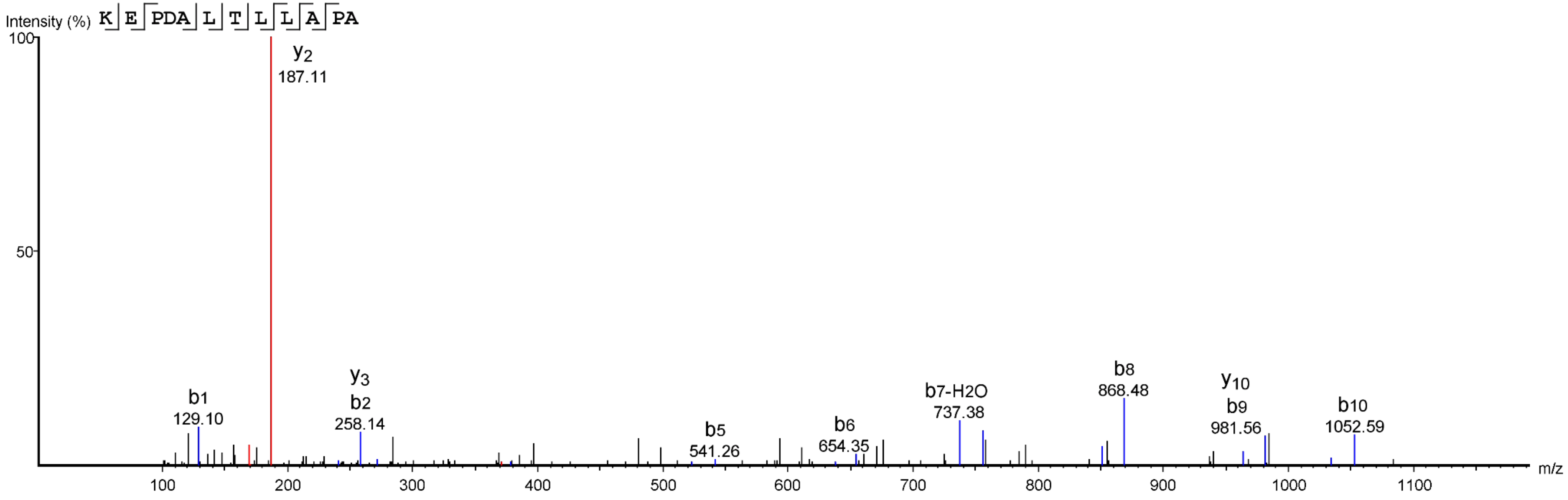

YMT93
Scan #17853

A-099: HIF1A*$_{(392-403)}$: KEPDALTLLAPA

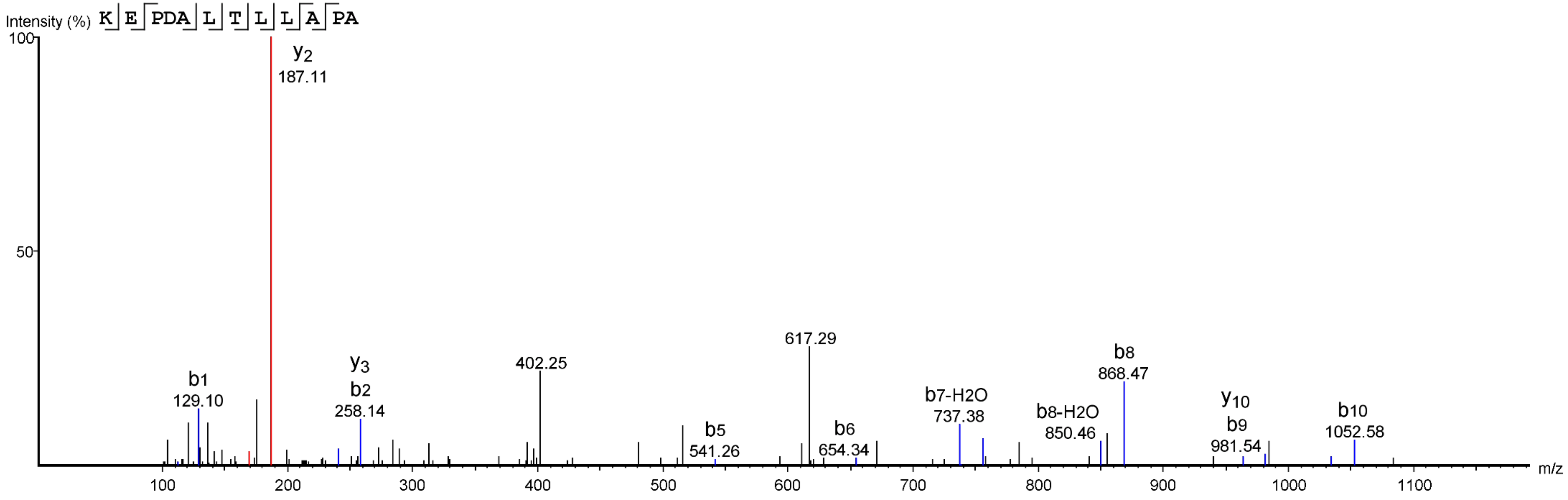

YMT95
Scan #18172

A-100: HIF1A*$_{(392\text{-}403)}$: KEPDALTLLAP(+15.99)A

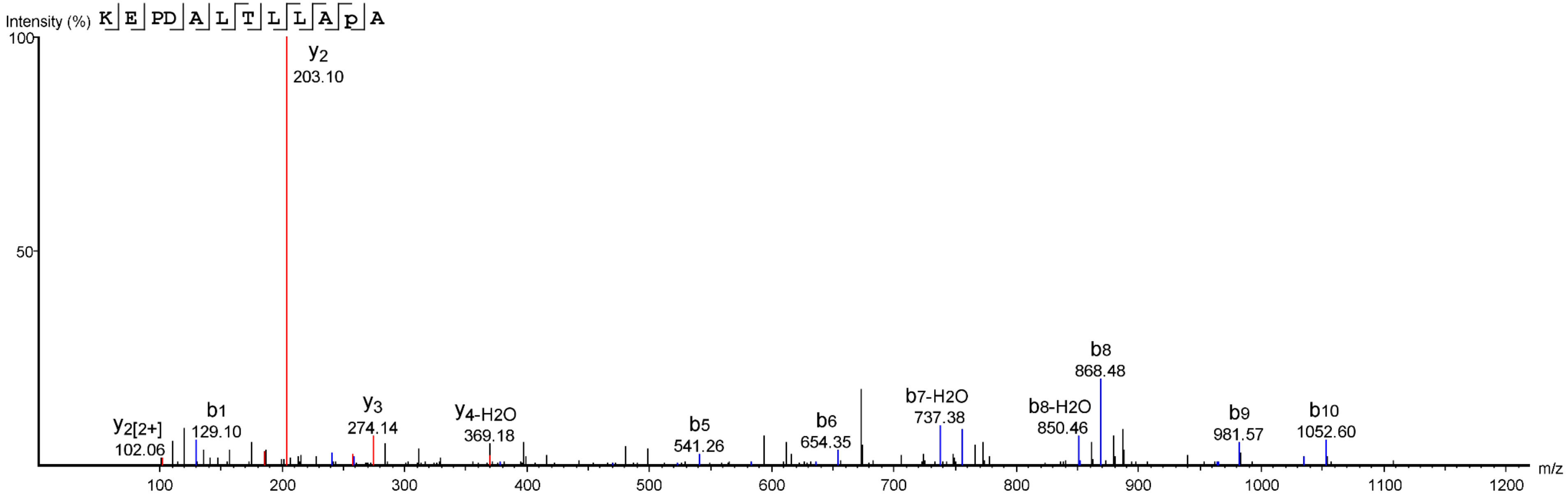

YMT94
Scan #16057

A-101: ACACB$_{(341-366)}$: RIPVQAVWAGWGHASENPKLPELLC(+57.02)K

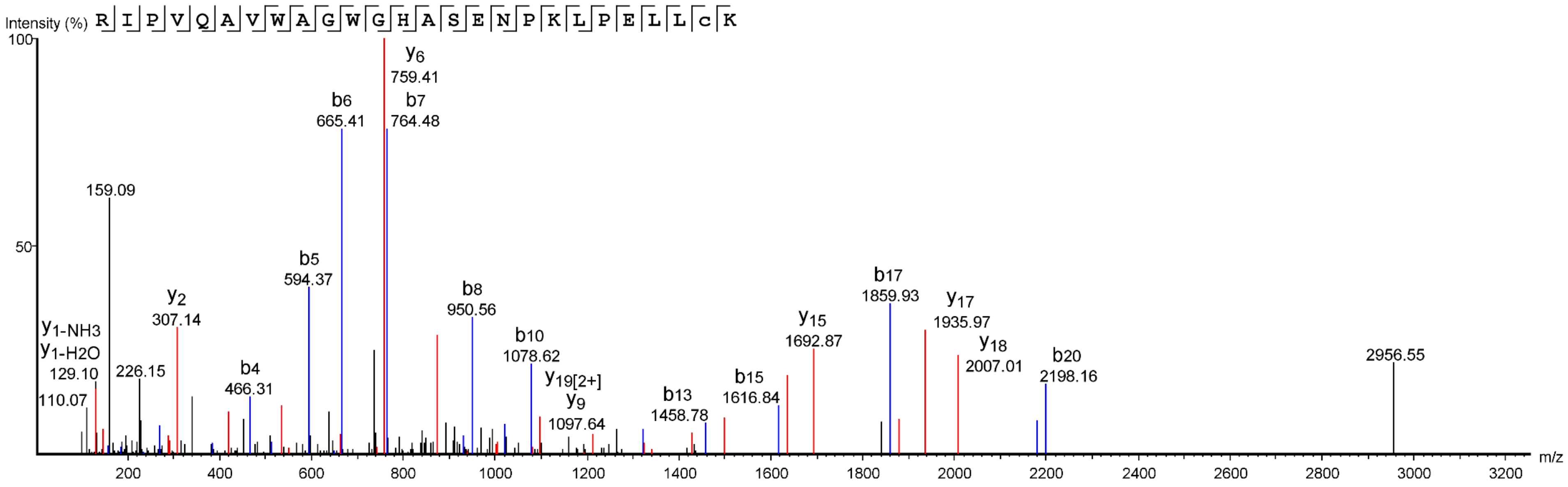

Pep17
Scan
#14304

A-102: ACACB$_{(341-366)}$: RIP(+15.99)VQAVWAGWGHASENPKLPELLC(+57.02)K

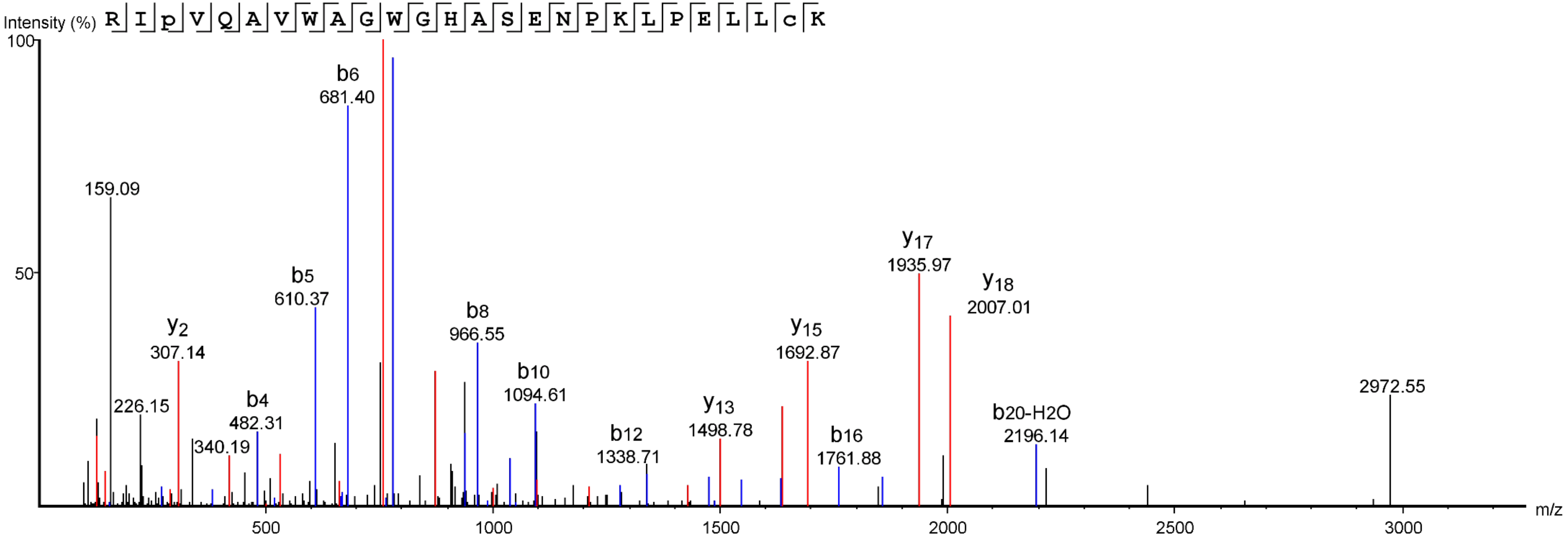

Pep17
Scan #13775

A-103: ACACB$_{(341-366)}$: RIPVQAVWAGWGHASENPKLPELLC(+57.02)K

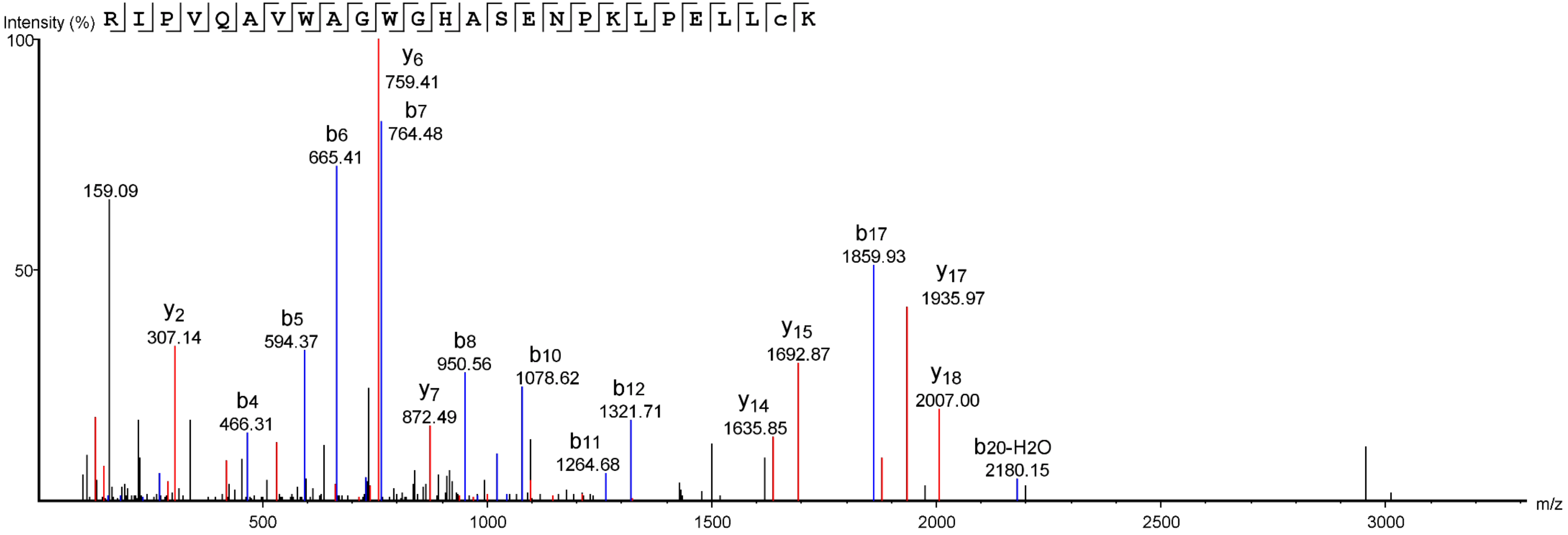

YMT86
Scan #19280

A-104: ACACB(341-366): RIPVQAVWAGWGHASENPKLPELLC(+57.02)K

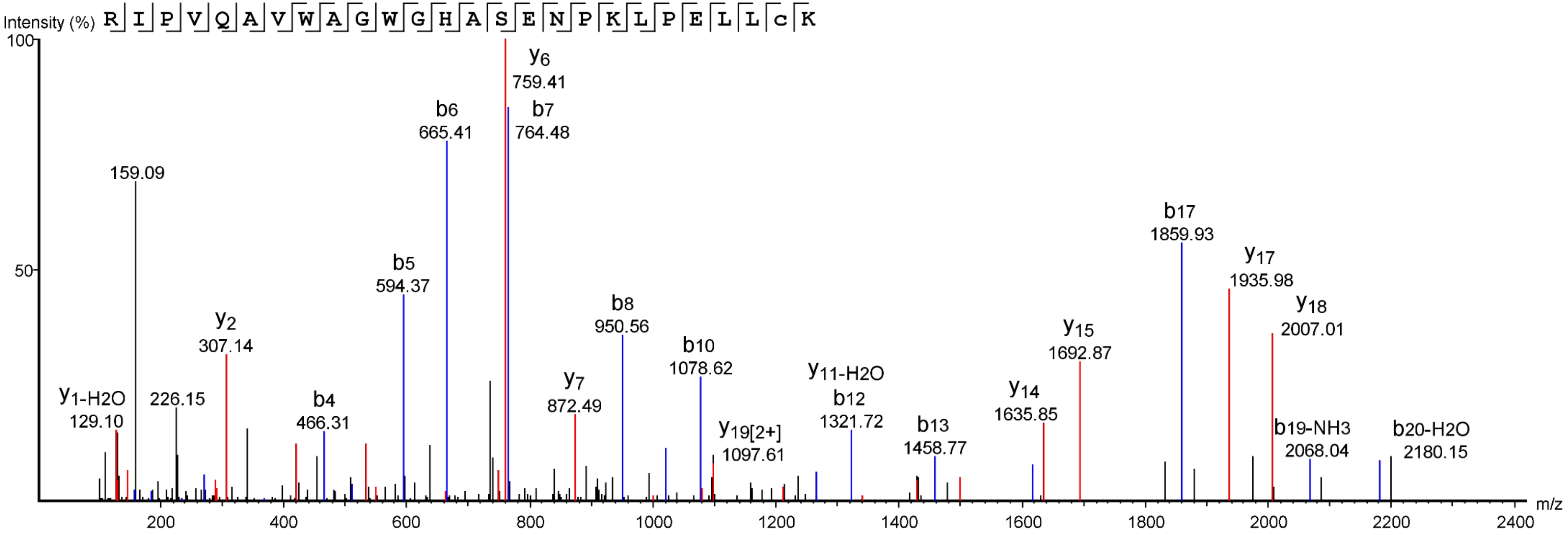

YMT88
Scan #18054

A-105: ACACB$_{(436-454)}$: DVDEGLEAAERIGFPLMIK

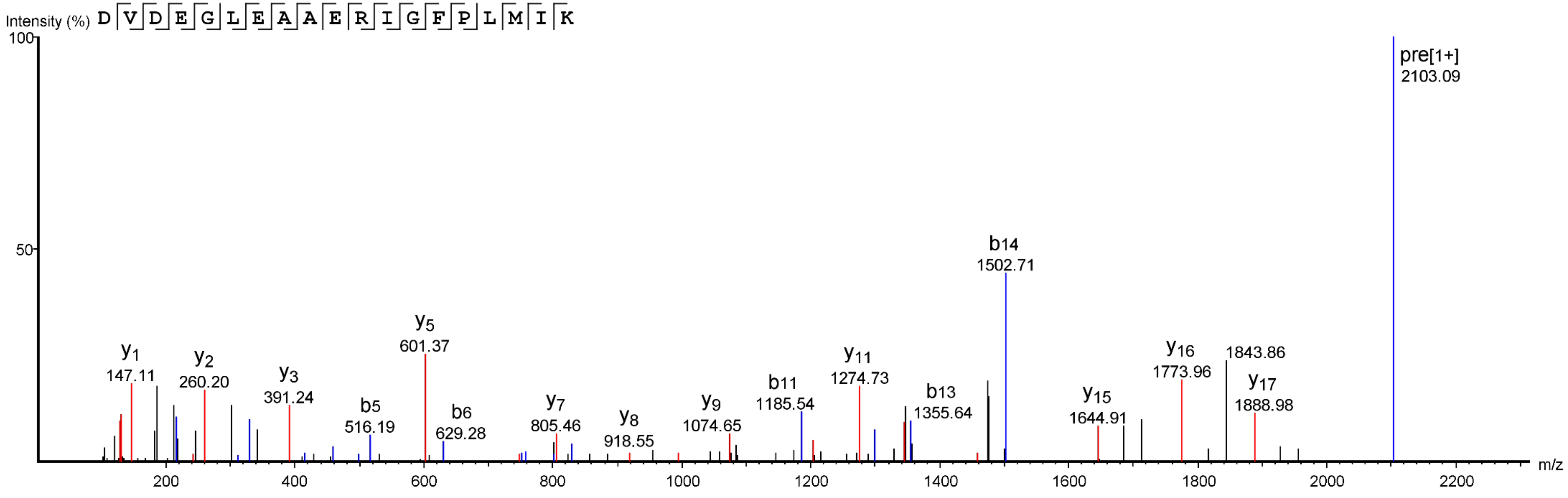

Pep17
Scan #19689

A-106: ACACB$_{(436-454)}$: DVDEGLEAAERIGFP(+15.99)LMIK

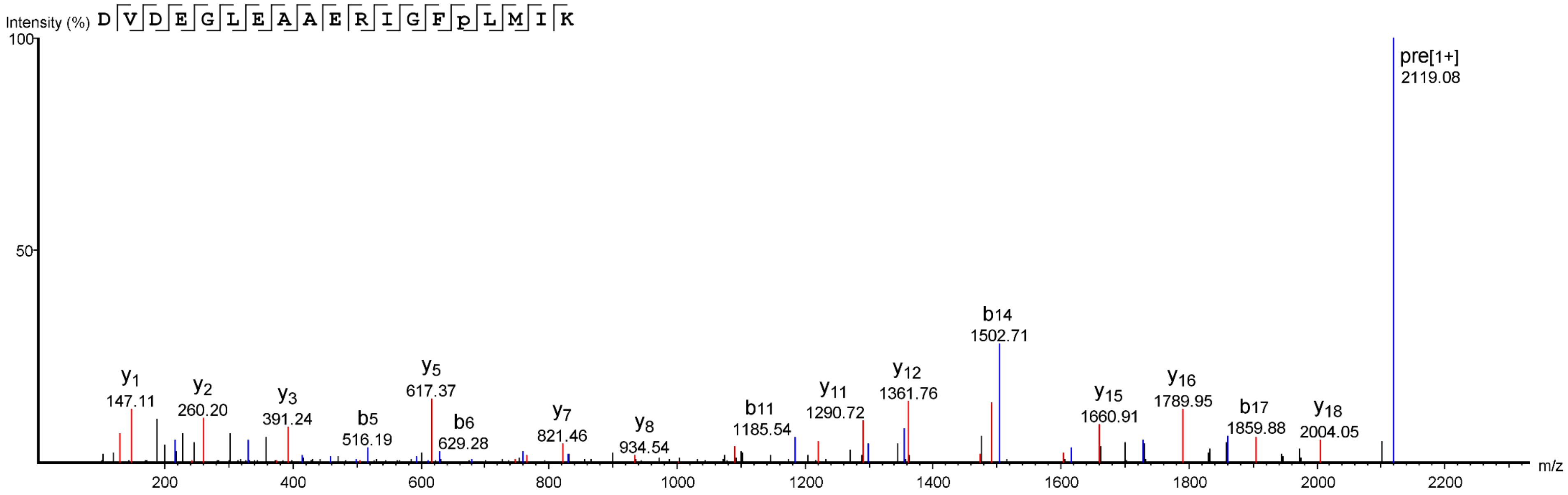

Pep17
Scan #18888

A-107: ACACB(436-454): DVDEGLEAAERIGFPLM(+15.99)IK

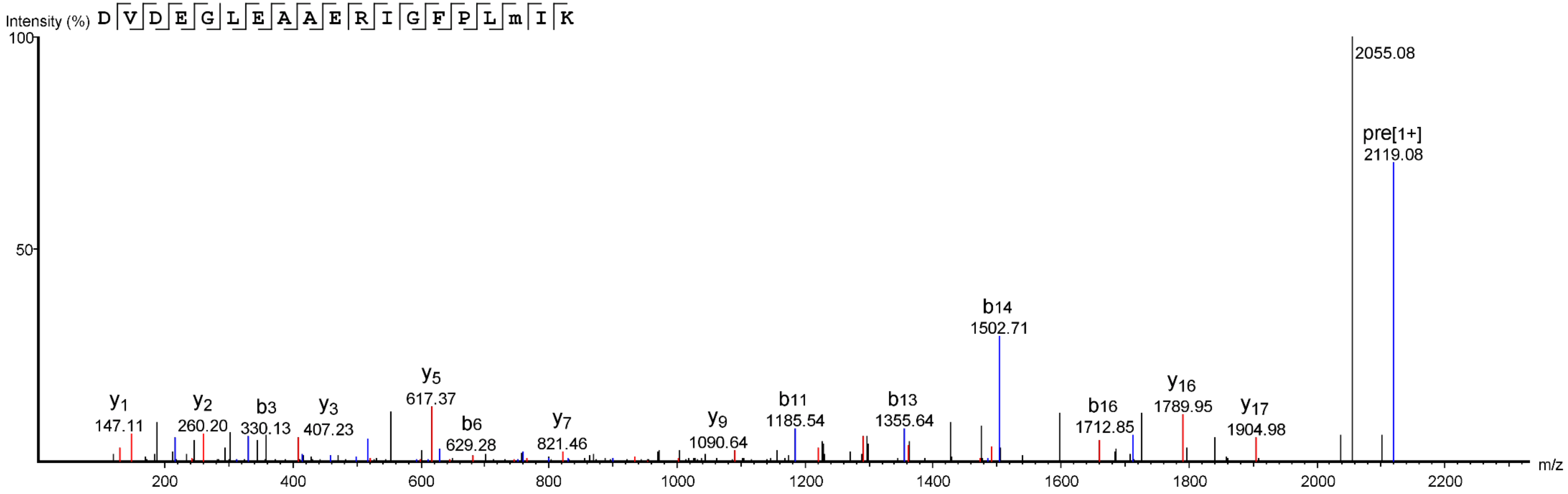

Pep17
Scan #18219

A-108: ACACB$_{(436-454)}$: DVDEGLEAAERIGFP(+15.99)LM(+15.99)IK

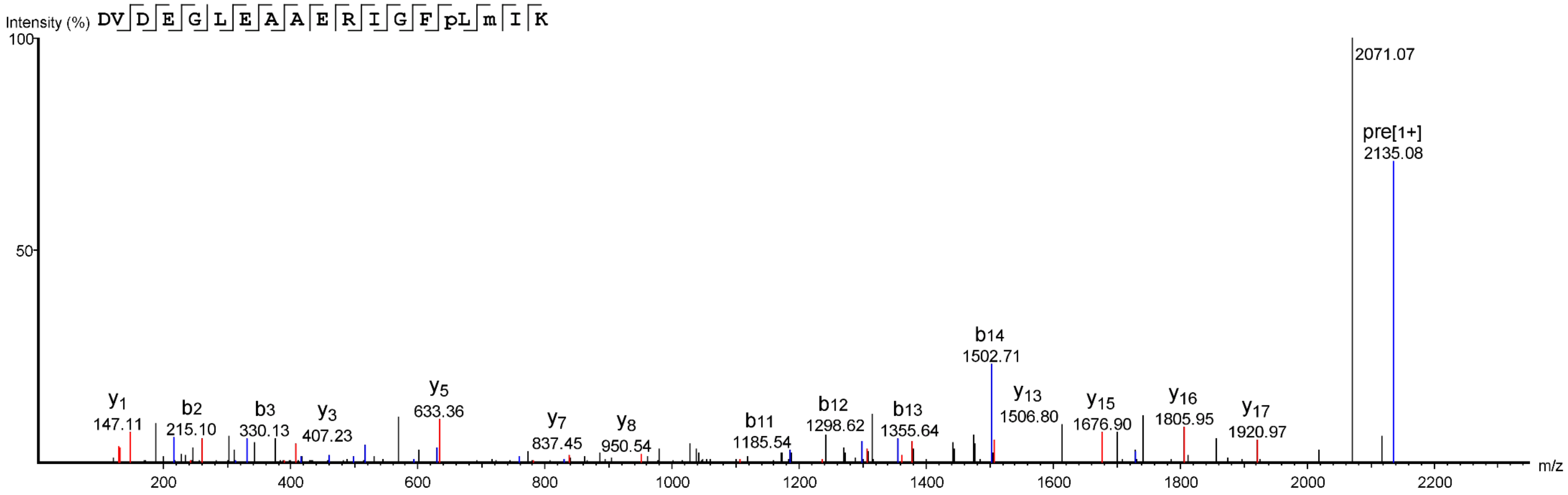

Pep17
Scan #17184

A-109: ACACB$_{(436-454)}$: DVDEGLEAAERIGFPLMIK

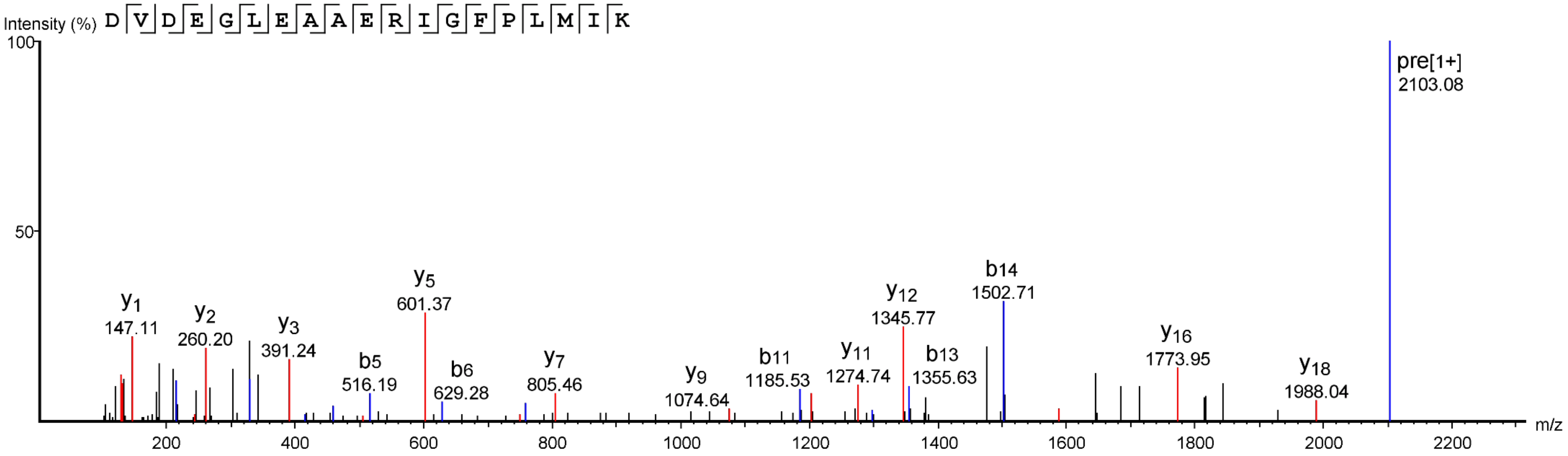

YMT86
Scan #25906

A-110: ACACB$_{(436-454)}$: DVDEGLEAAERIGFPLMIK

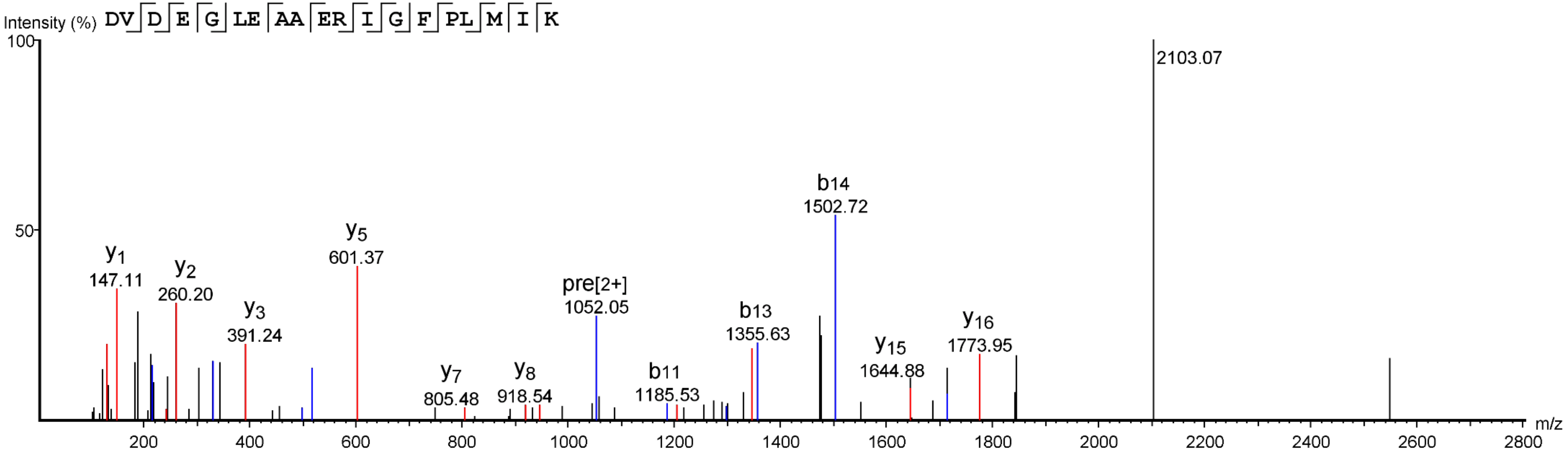

YMT88
Scan #24140

A-111: ACACB$_{(436-454)}$: DVDEGLEAAERIGFPLM(+15.99)IK

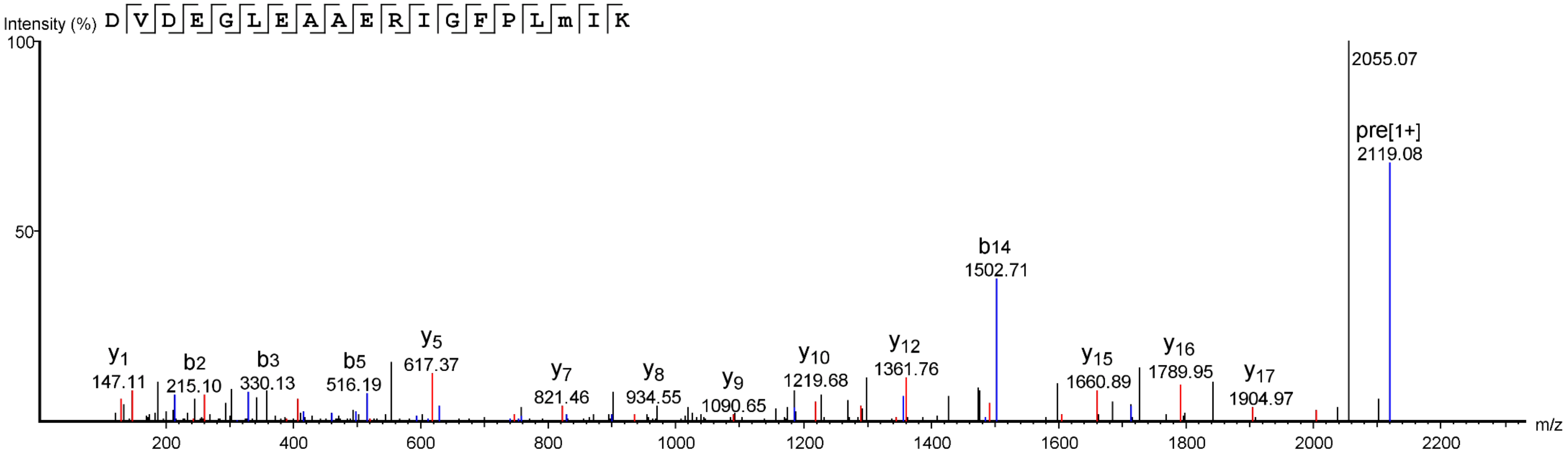

YMT86
Scan #24339

A-112: ACACB$_{(436-454)}$: DVDEGLEAAERIGFPLM(+15.99)IK

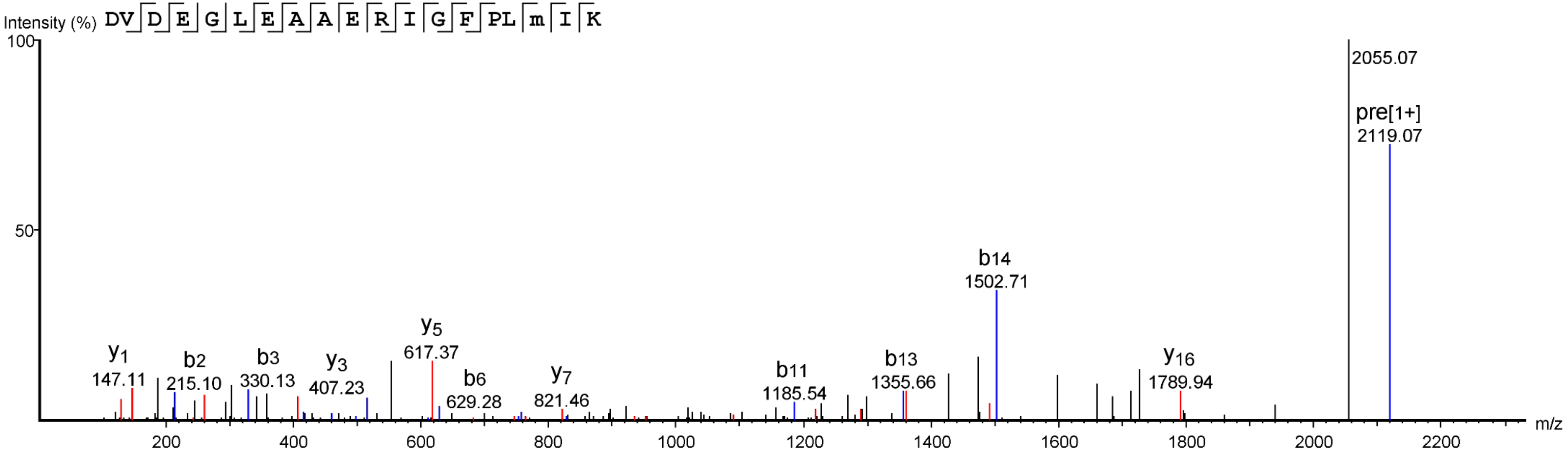

YMT88
Scan #22680

A-113: $ACTB_{(292-312)}$: DLYANTVLSGGTTMYPGIADR

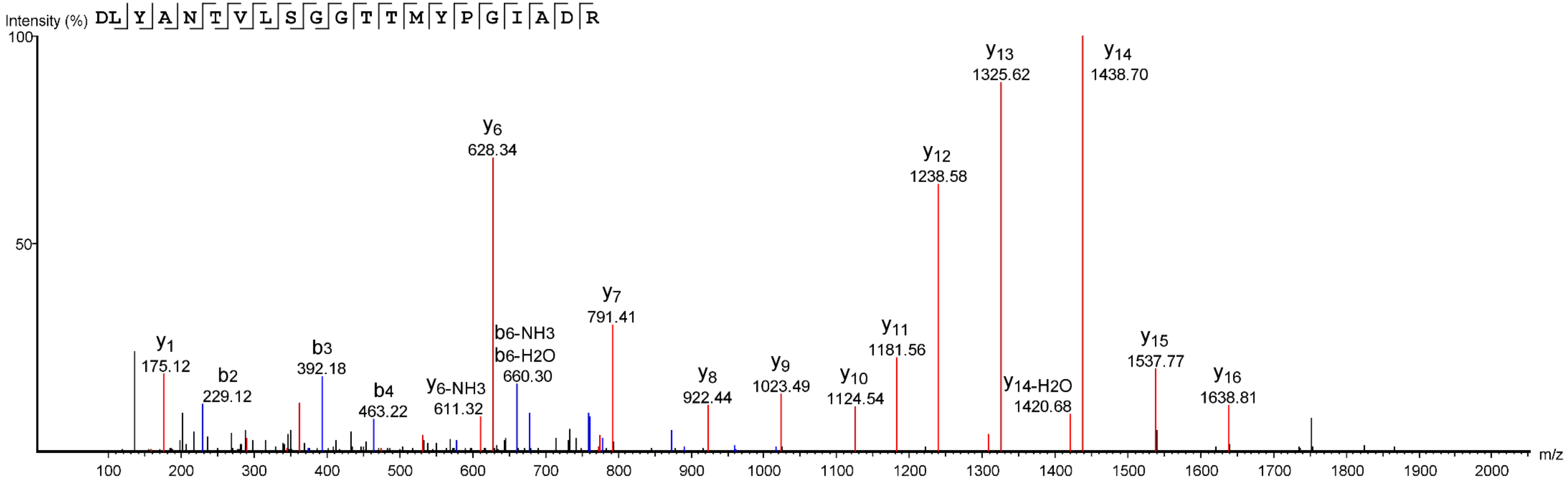

Pep20
Scan #10685

A-114: ACTB$_{(292\text{-}312)}$: DLYANTVLSGGTTM(+15.99)YPGIADR

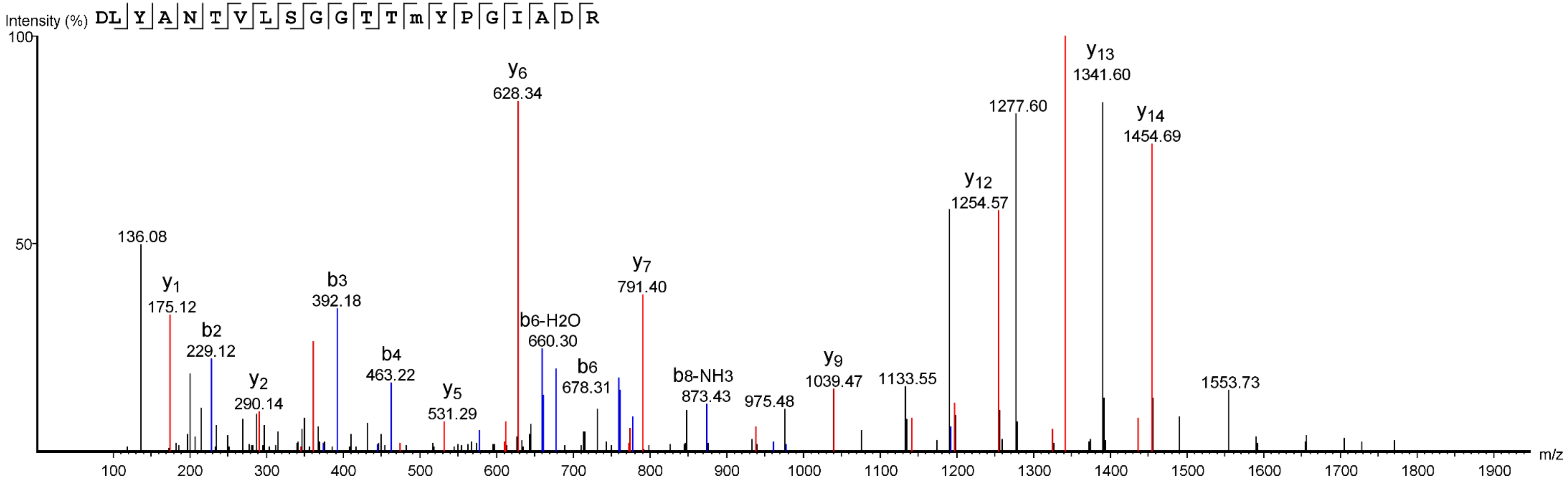

Pep20
Scan #9564

A-115: ACTB(292-312): DLYANTVLSGGTTMYP(+15.99)GIADR

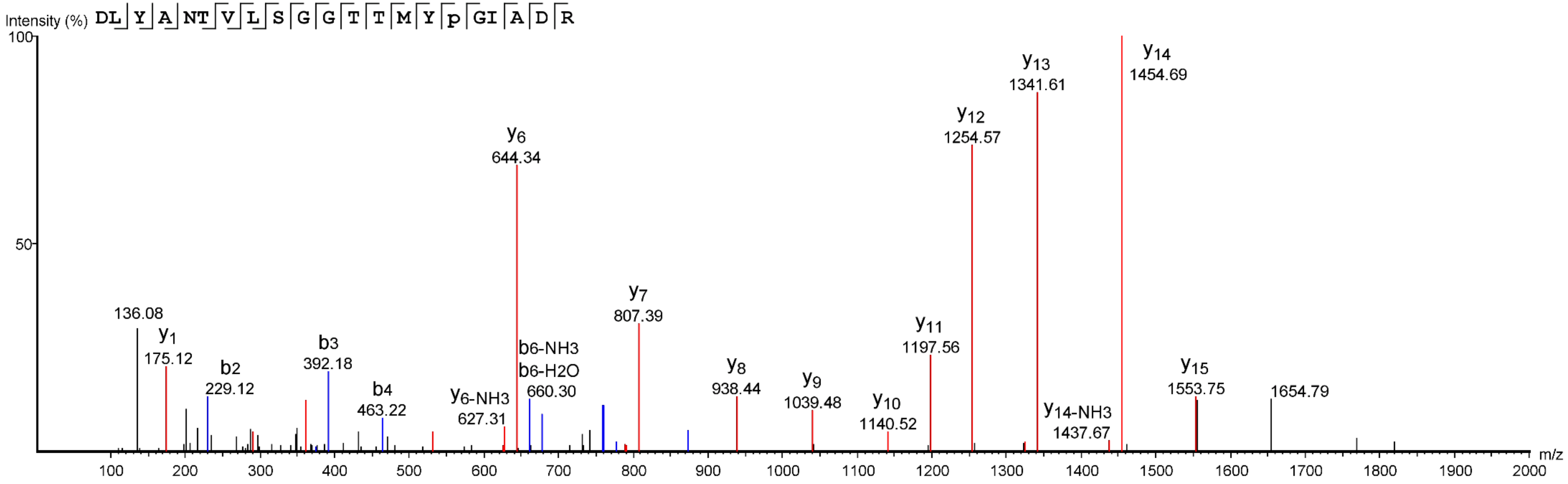

Pep20

Scan #10295

A-116: ACTB$_{(292-312)}$: DLYANTVLSGGTTM(+15.99)YP(+15.99)GIADR

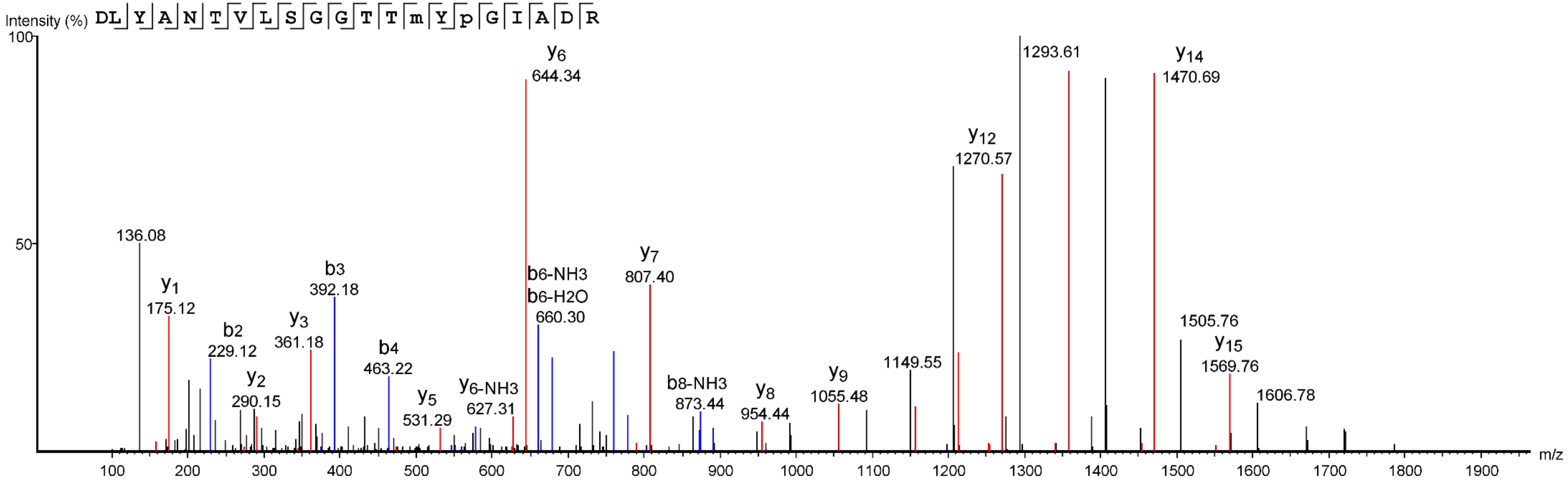

Pep20

Scan #9055

A-117: ACTB(292-312): DLYANTVLSGGTTMYPGIADR

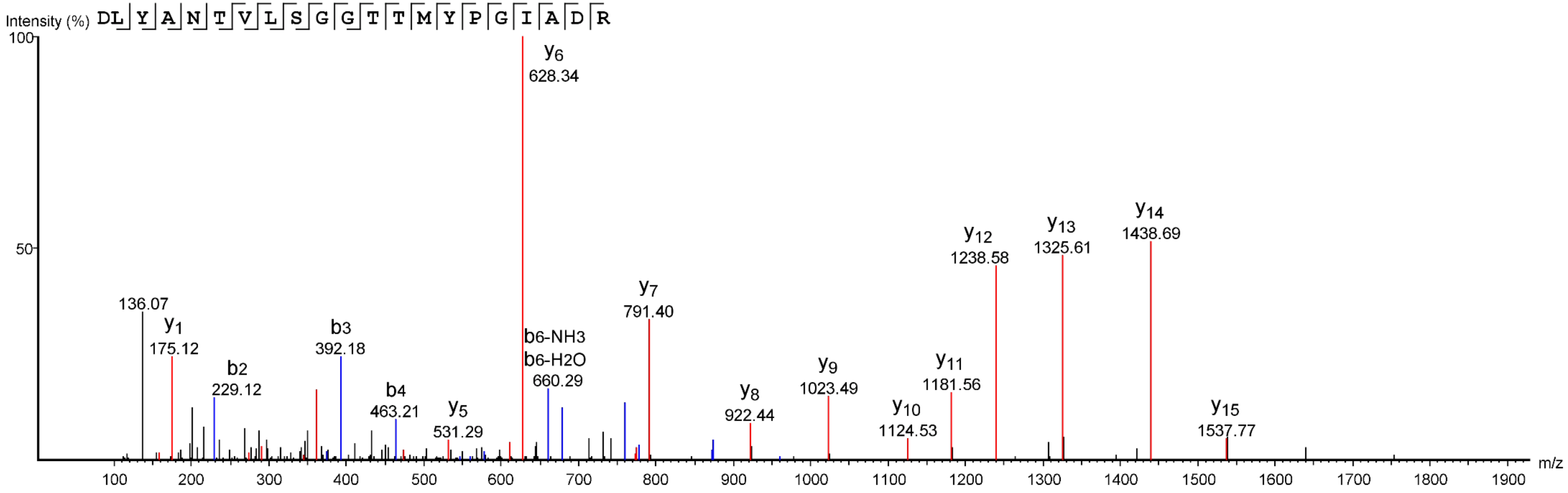

YMT41
Scan #20318

A-118: ACTB$_{(292-312)}$: DLYANTVLSGGTTMYPGIADR

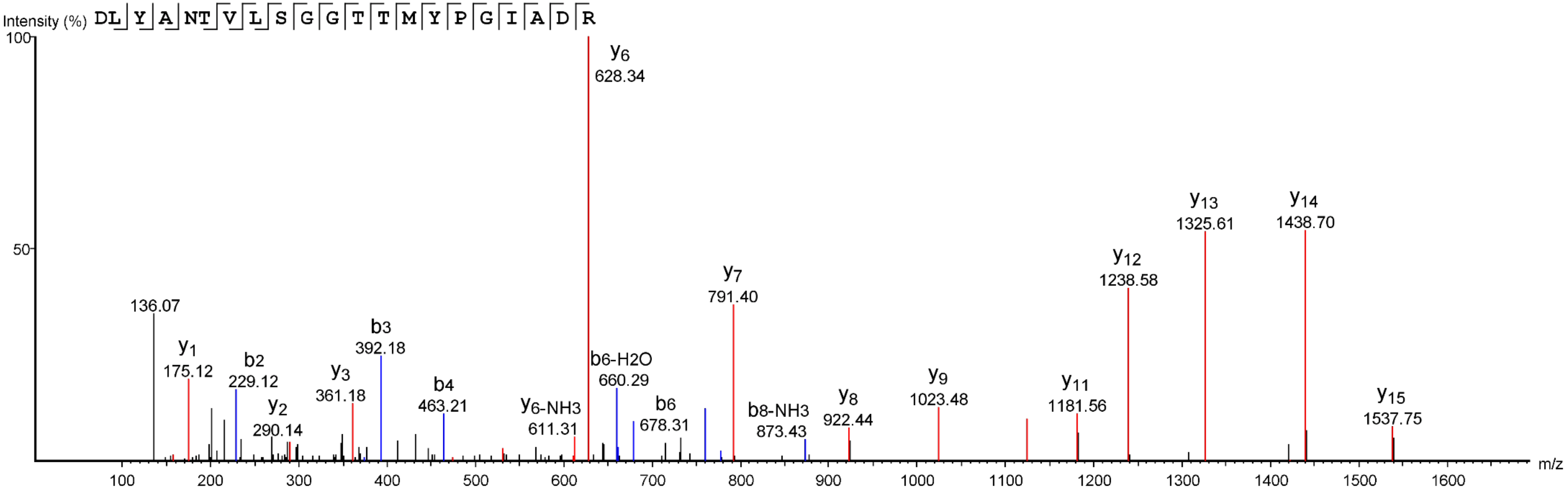

YMT42
Scan #20913

A-119: ACTB$_{(292-312)}$: DLYANTVLSGGTTM(+15.99)YPGIADR

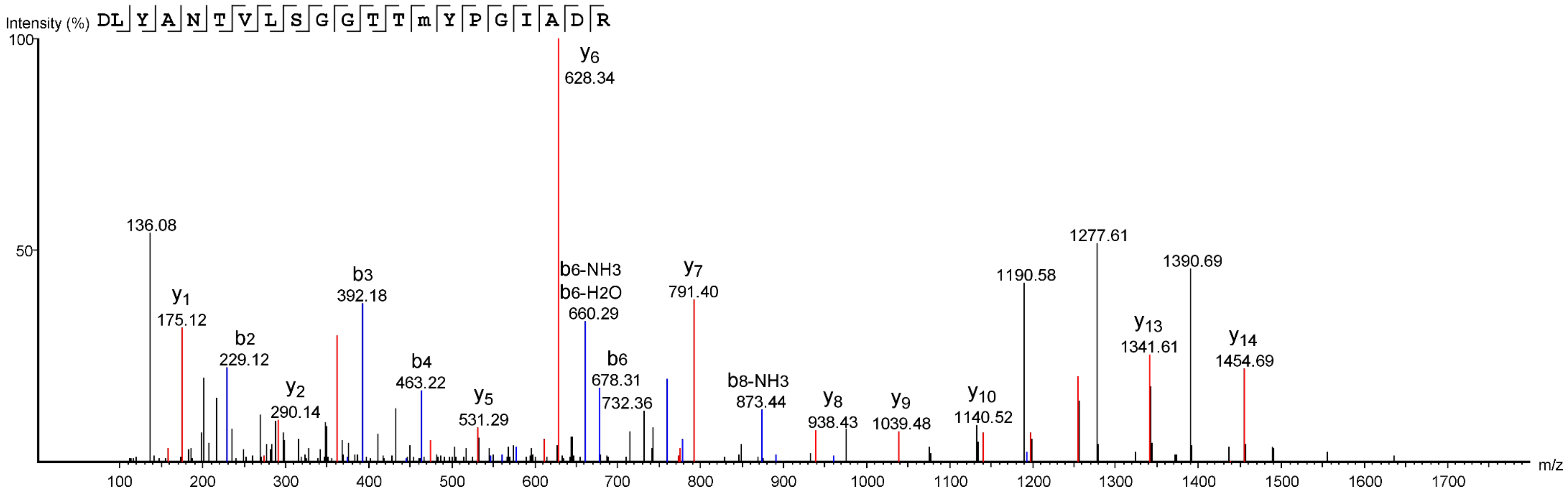

YMT41
Scan #18734

A-120: ACTB$_{(292-312)}$: DLYANTVLSGGTTM(+15.99)YPGIADR

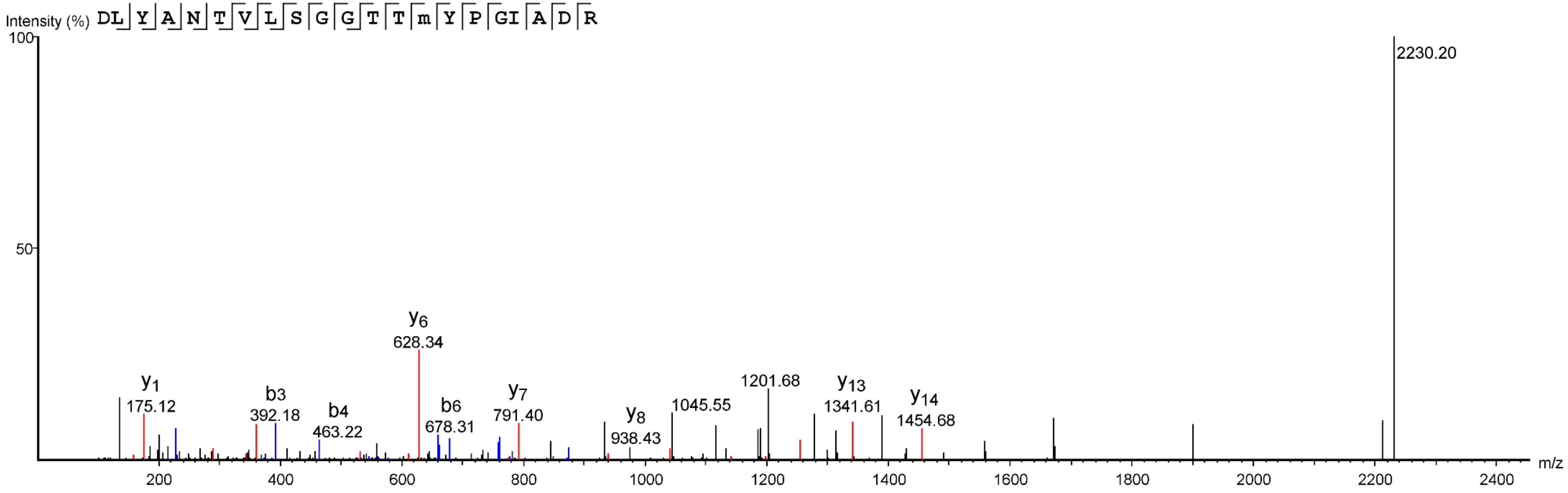

YMT42
Scan #19446

A-121: ACTB$_{(316-326)}$: EITALAPSTMK

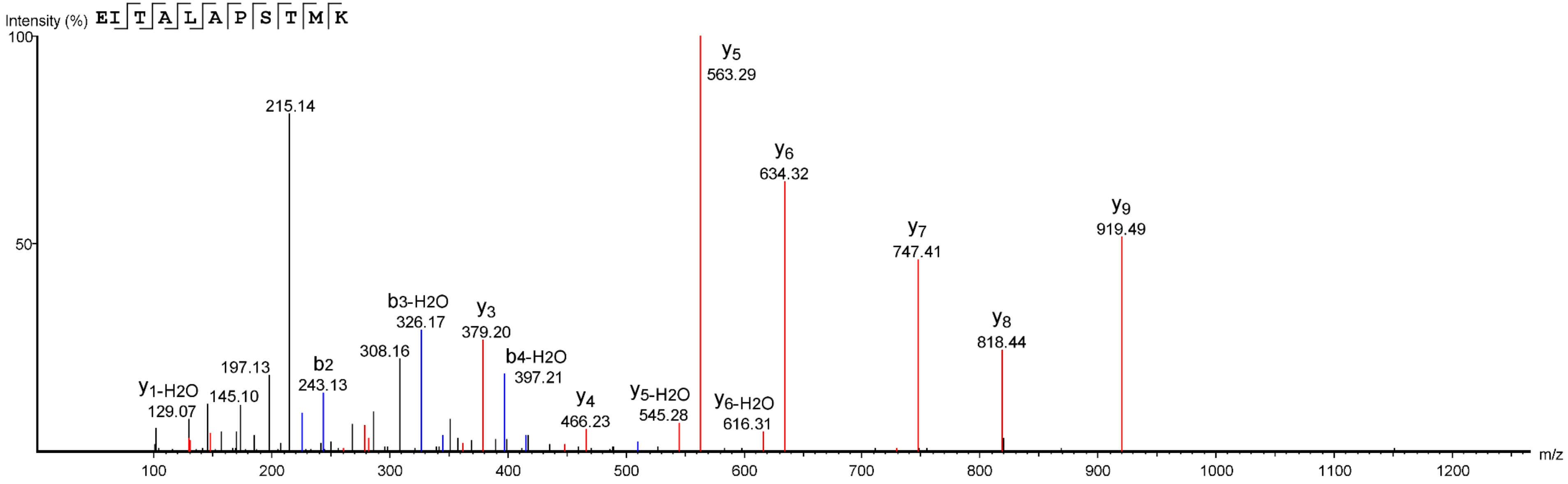

Pep20
Scan #5455

A-122: ACTB$_{(316-326)}$: EITALAP(+15.99)STMK

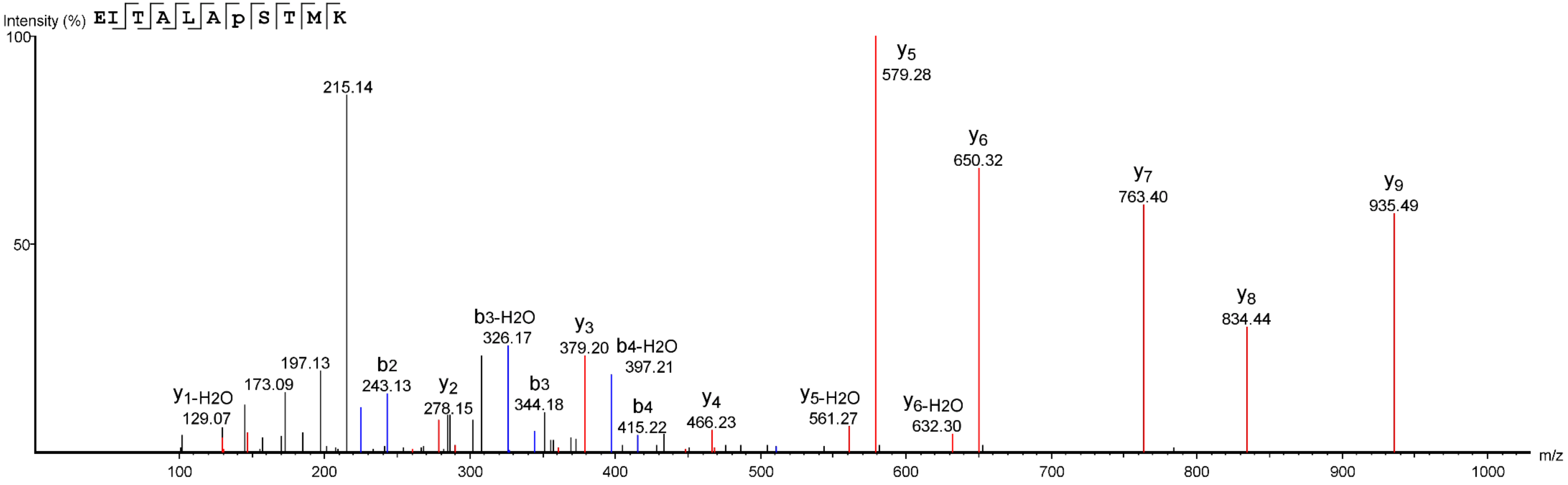

Pep20
Scan #4813

A-123: ACTB(316-326): EITALAPSTM(+15.99)K

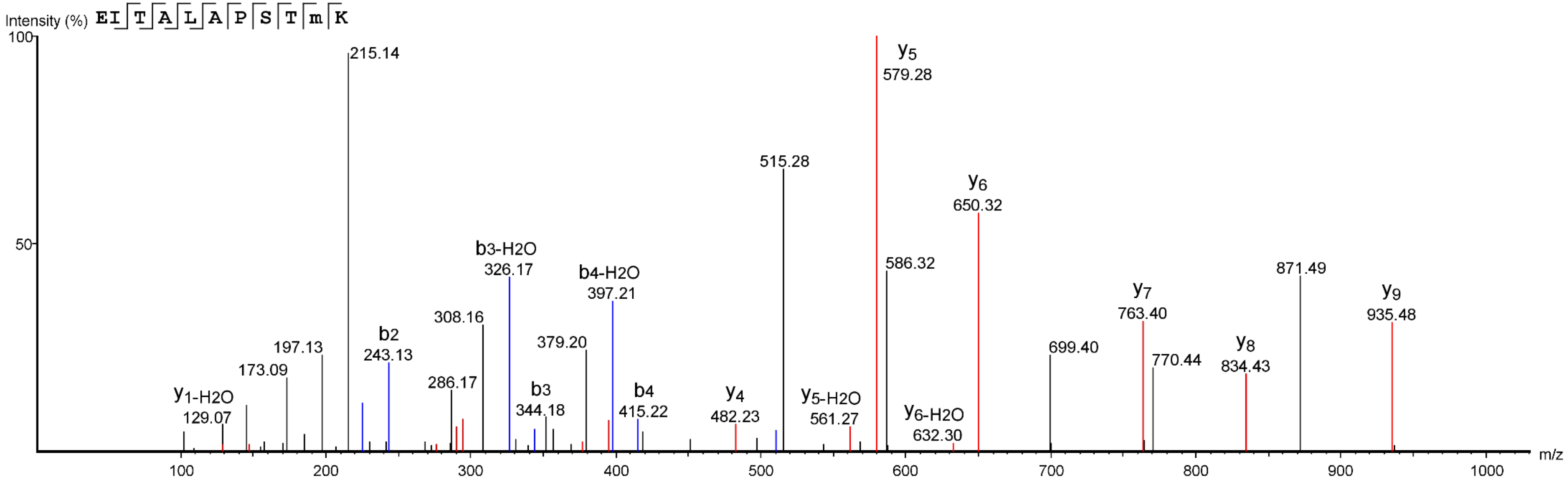

Pep20
Scan #4594

A-124: ACTB$_{(316\text{-}326)}$: EITALAP(+15.99)STM(+15.99)K

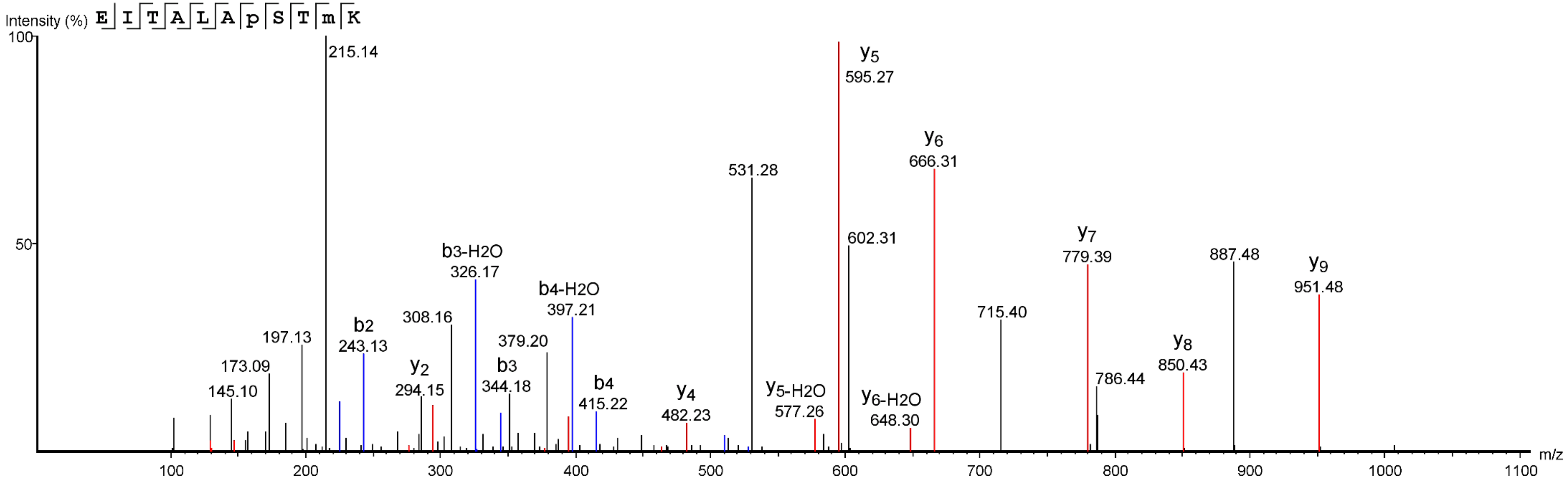

Pep20
Scan #4047

A-125: ACTB(316-326): EITALAPSTMK

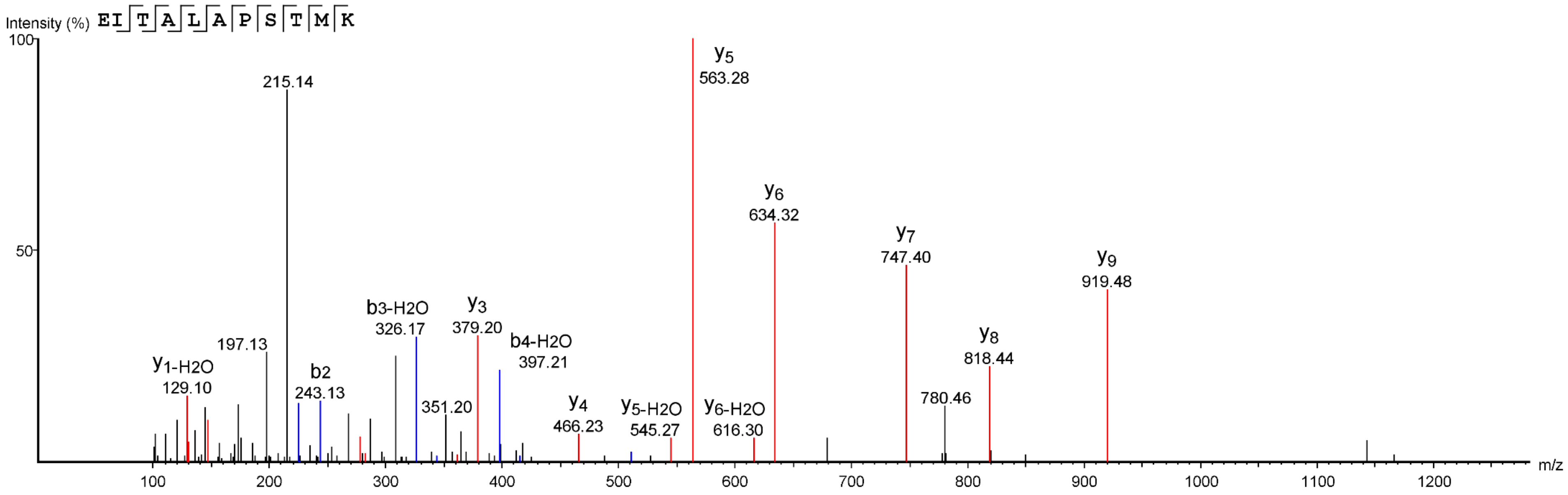

YMT41

Scan #11523

A-126: $ACTB_{(316\text{-}326)}$: EITALAPSTMK

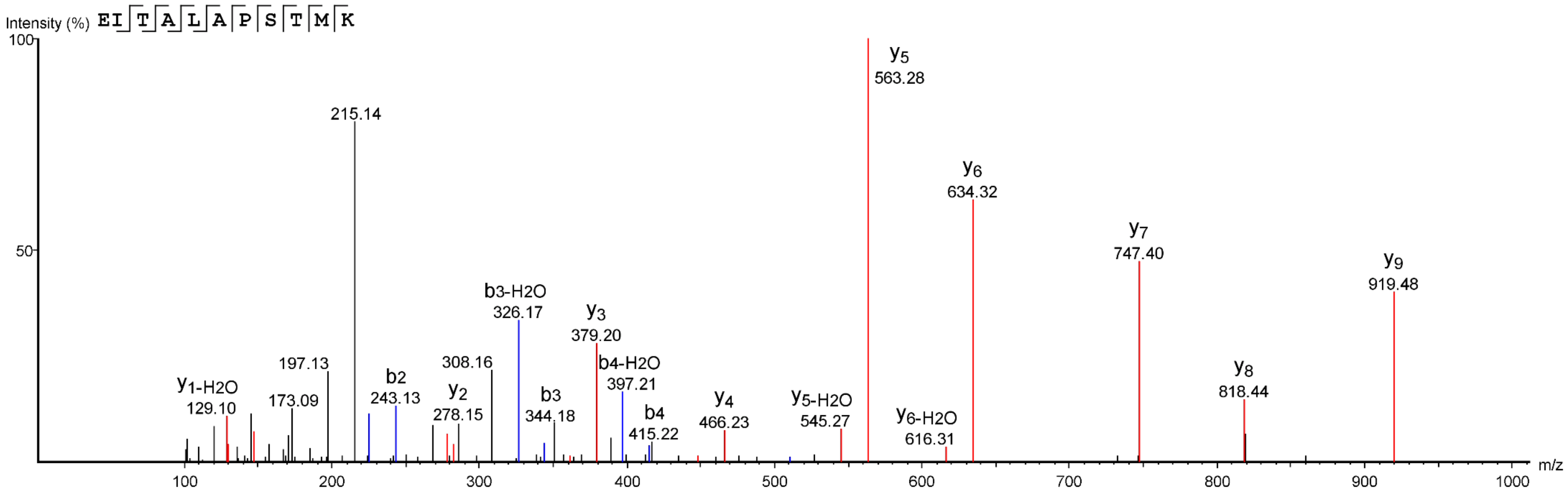

YMT42

Scan #11329

A-127: ACTB$_{(316-326)}$: EITALAPSTM(+15.99)K

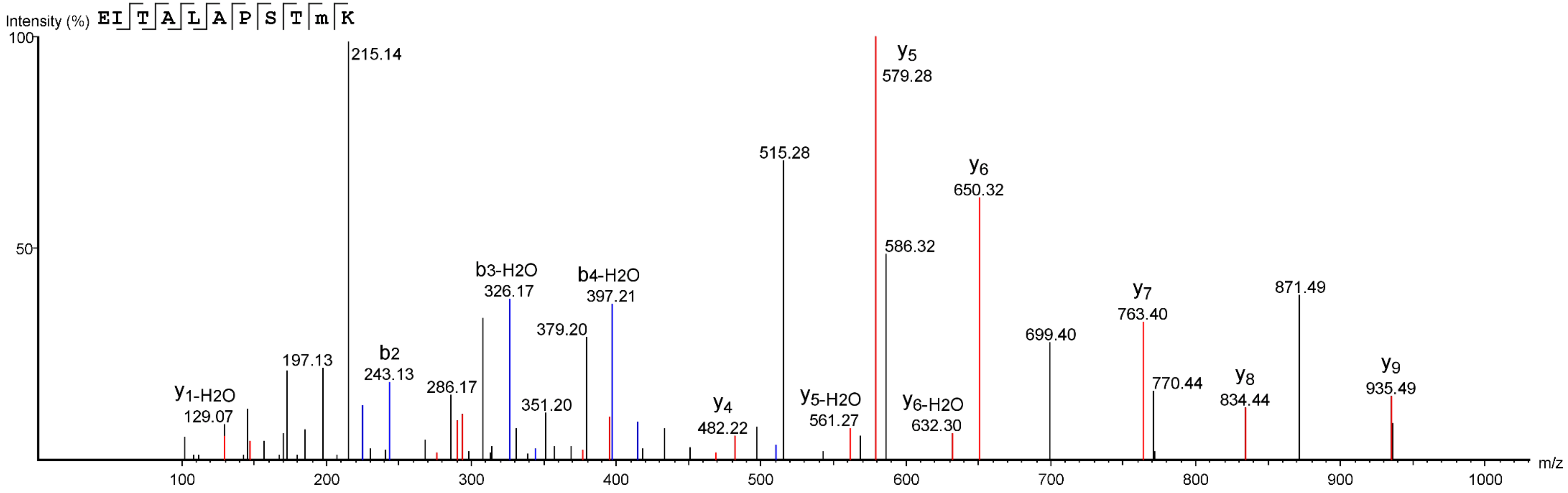

YMT41
Scan #8734

A-128: ACTB$_{(316\text{-}326)}$: EITALAPSTM(+15.99)K

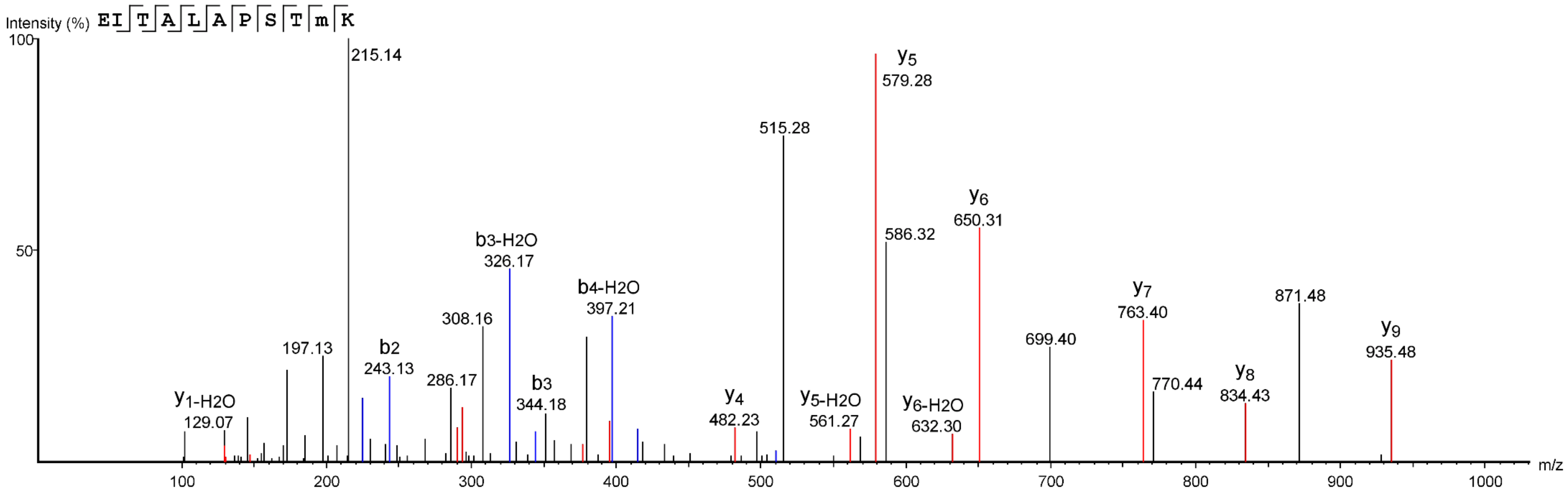

YMT42
Scan #8481

A-129: ADRB2$_{(376-404)}$: LLC(+57.02)EDLPGTEDFVGHQGTVPSDNIDSQGR+

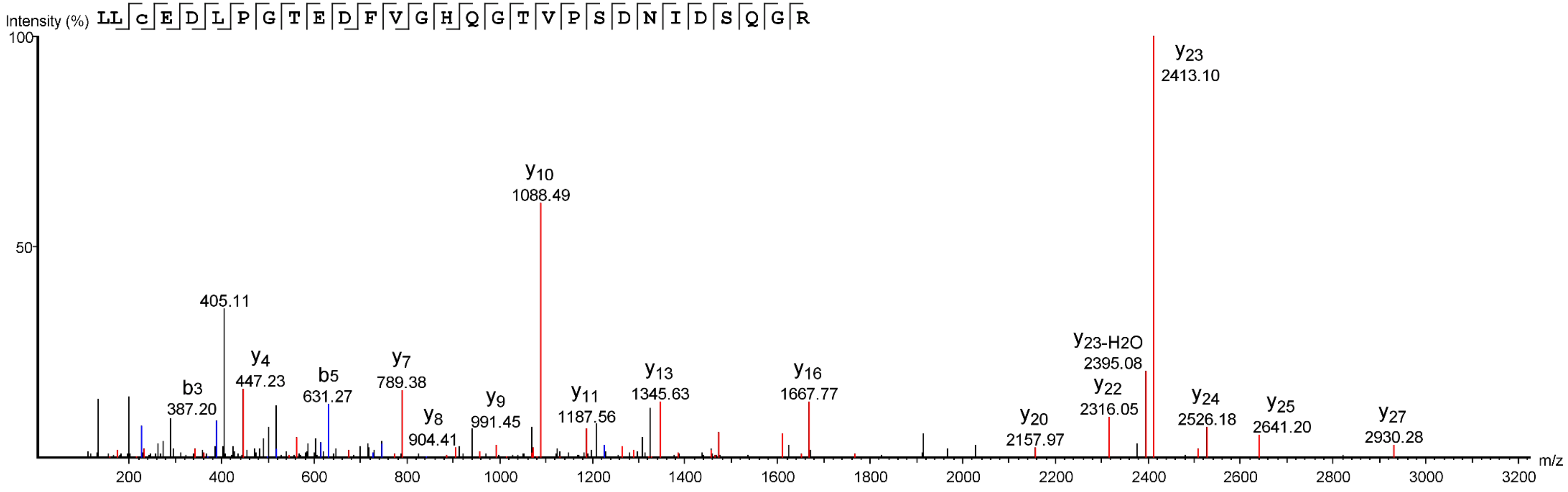

Pep11
Scan #14519

A-130: ADRB2$_{(376-404)}$: LLC(+57.02)EDLP(+15.99)GTEDFVGHQGTVPSDNIDSQGR+

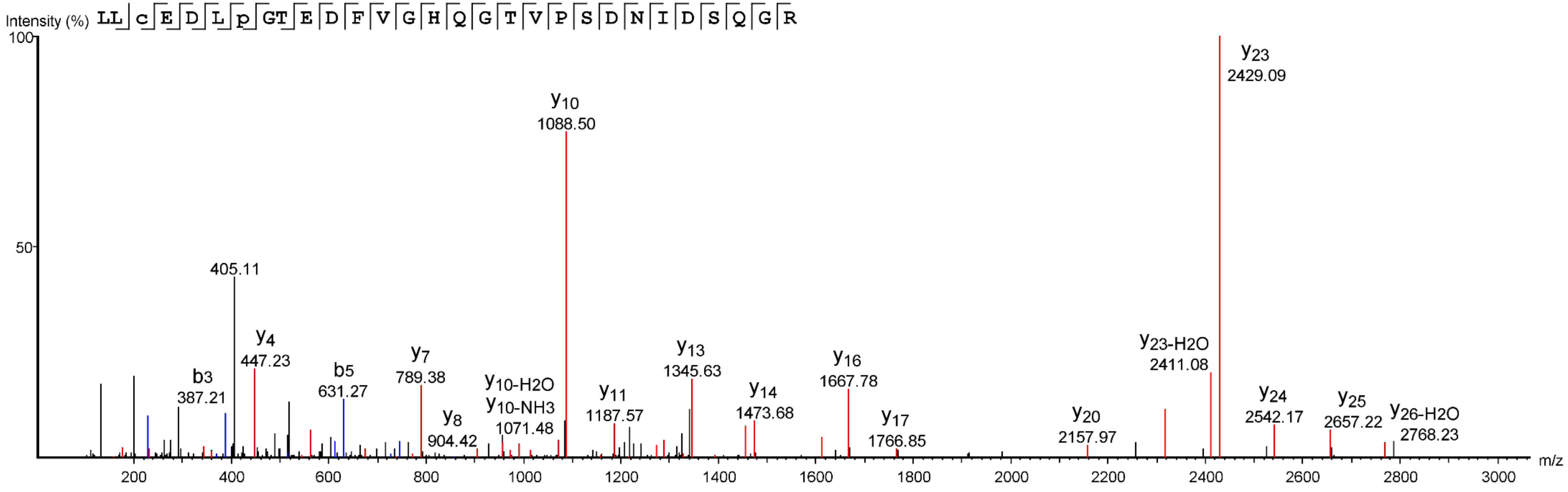

Pep11
Scan #13797

A-131: ADRB2$_{(376-404)}$: LLC(+57.02)EDLPGTEDFVGHQGTVP(+15.99)SDNIDSQGR+

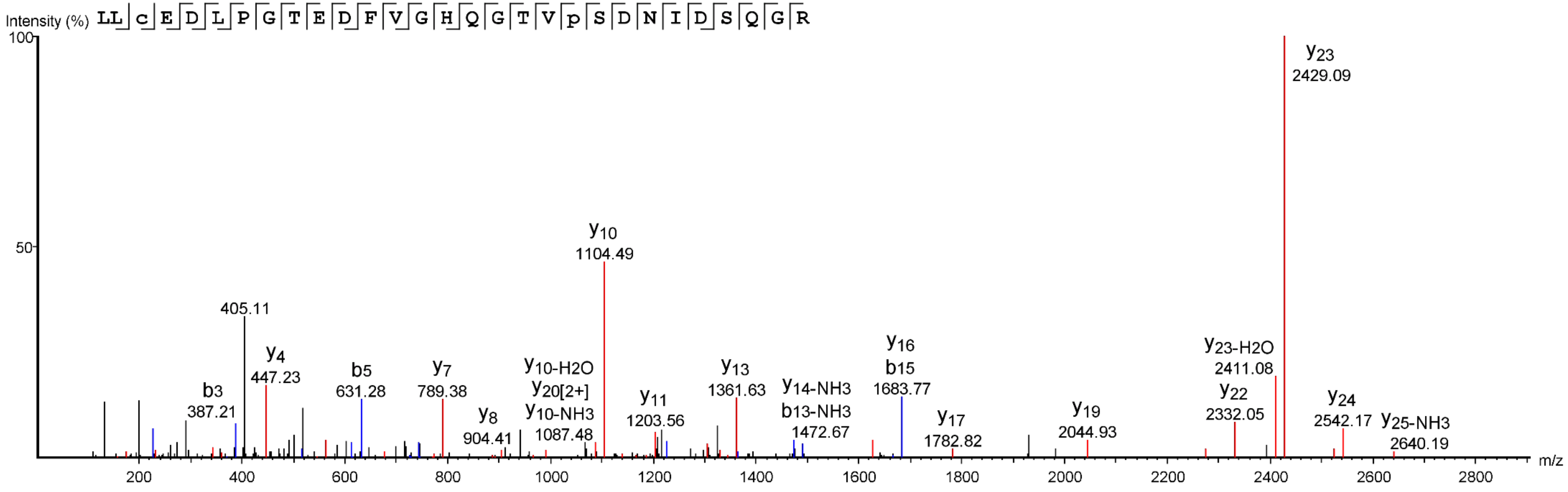

Pep11
Scan #13989

A-132: ADRB2$_{(376-404)}$: LLC(+57.02)EDLP(+15.99)GTEDFVGHQGTVP(+15.99)SDNIDSQGR+

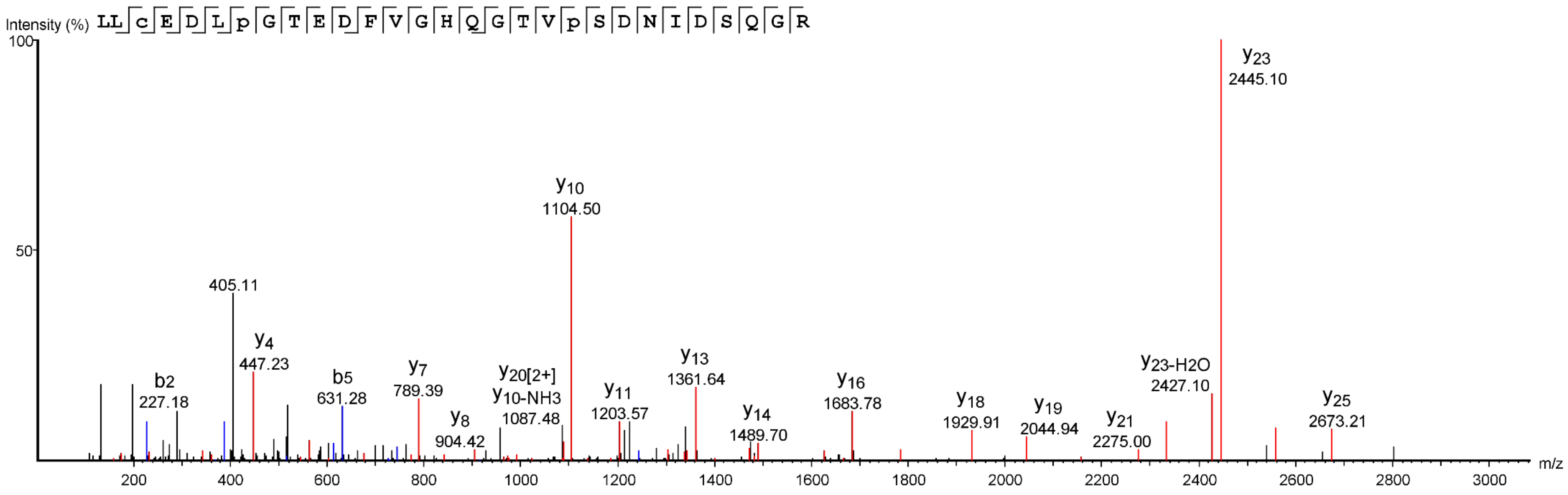

Pep11

Scan #13301

A-133: ADRB2$_{(376-404)}$: LLC(+57.02)EDLPGTEDFVGHQGTVPSDNIDSQGR+

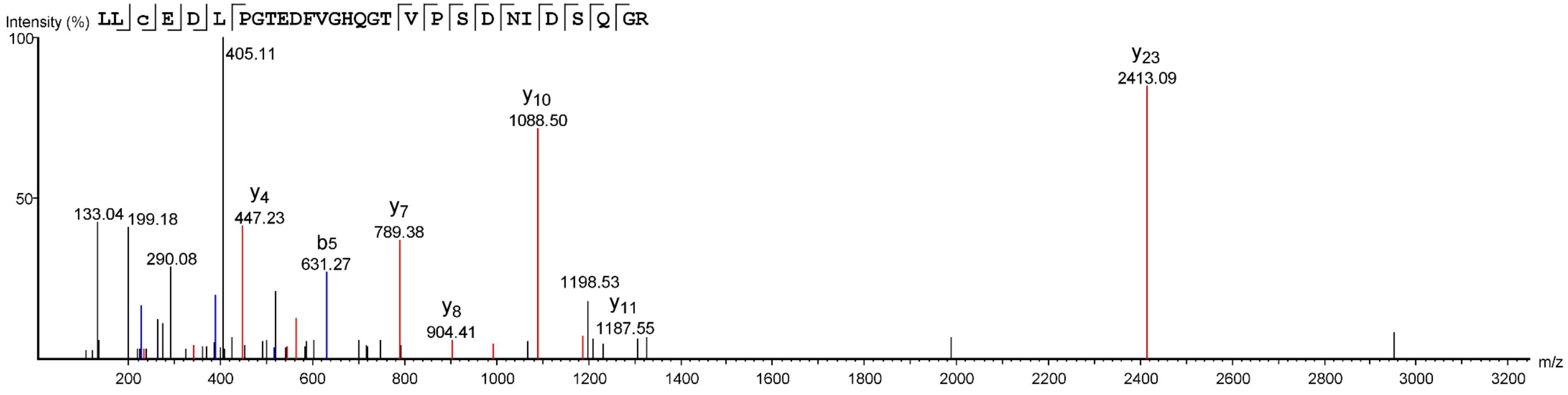

YMT65
Scan #14574

A-134: ADRB2$_{(376-404)}$: LLC(+57.02)EDLPGTEDFVGHQGTVPSDNIDSQGR+

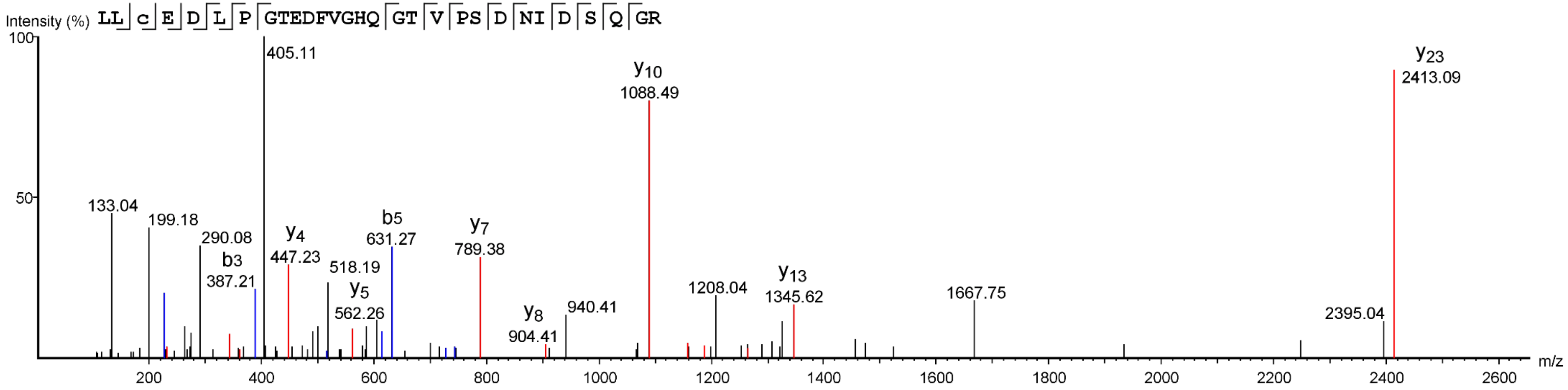

YMT66
Scan #14387

A-135: ADRB2$_{(376-404)}$: LLC(+57.02)ED(+15.99)LPGTEDFVGHQGTVPSDNIDSQGR+

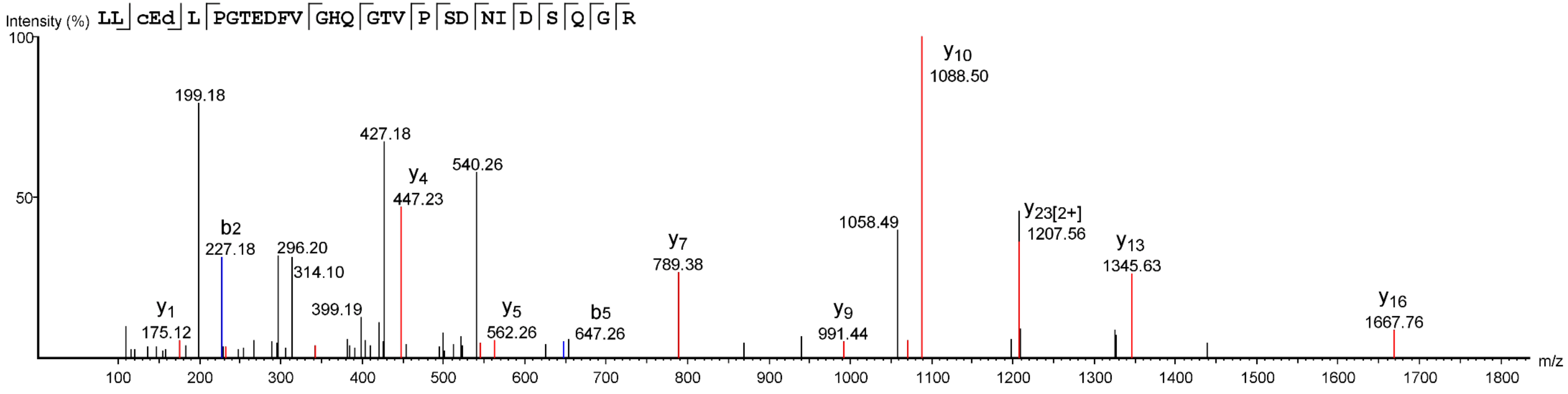

YMT65
Scan #14273

A-136: ADRB2$_{(376-404)}$: LLC(+57.02)ED(+15.99)LPGTEDFVGHQGTVPSDNIDSQGR+

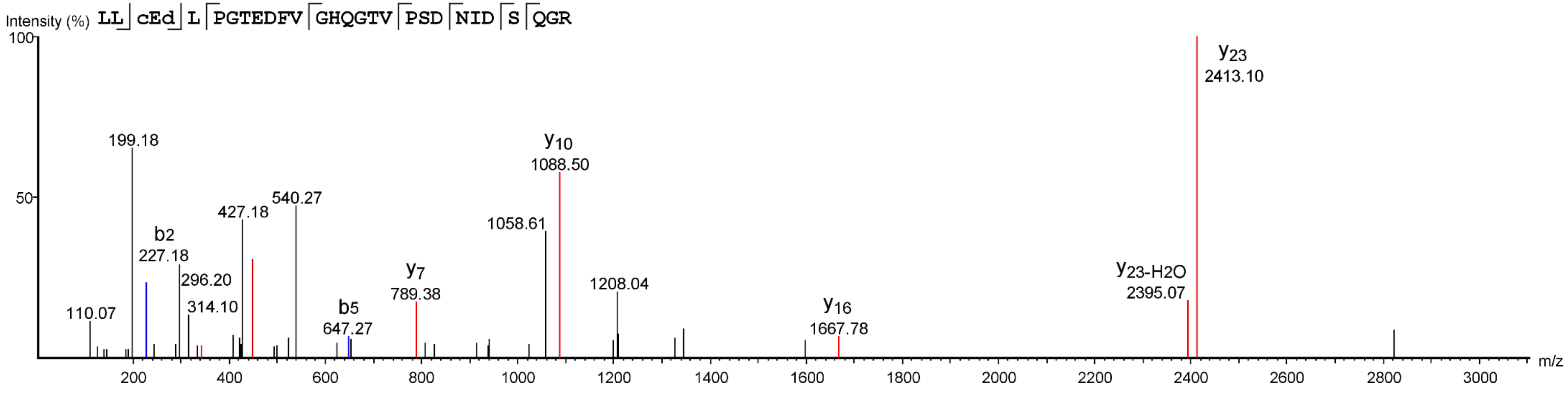

YMT66
Scan #13937

A-137: AKT1$_{(122-140)}$: SGSPSDNSGAEEMEVSLAK

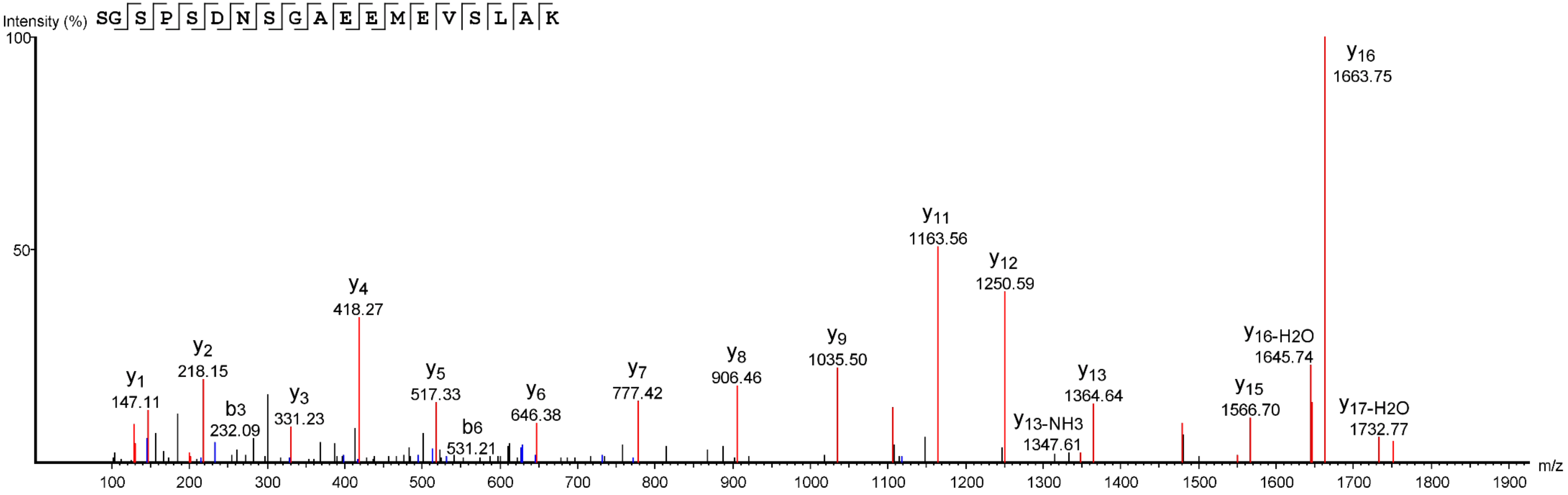

Pep17
Scan #9951

A-138: AKT1$_{(122-140)}$: SGSP(+15.99)SDNSGAEEMEVSLAK

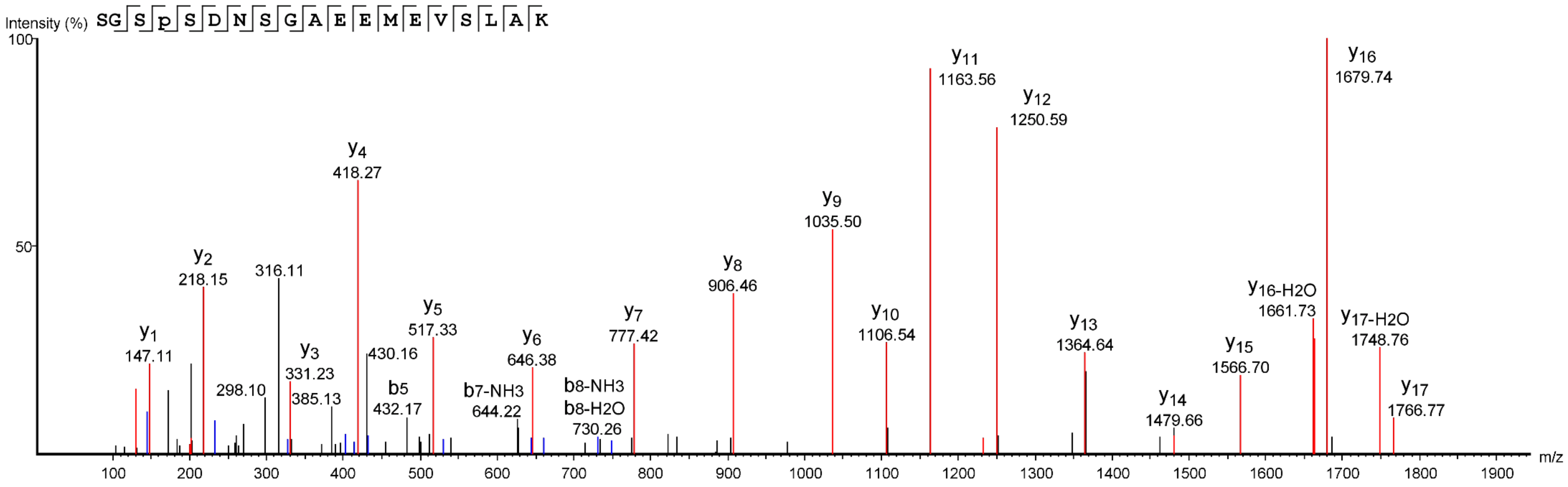

Pep17

Scan #9840

A-139: AKT1$_{(122-140)}$: SGSPSDNSGAEEM(+15.99)EVSLAK

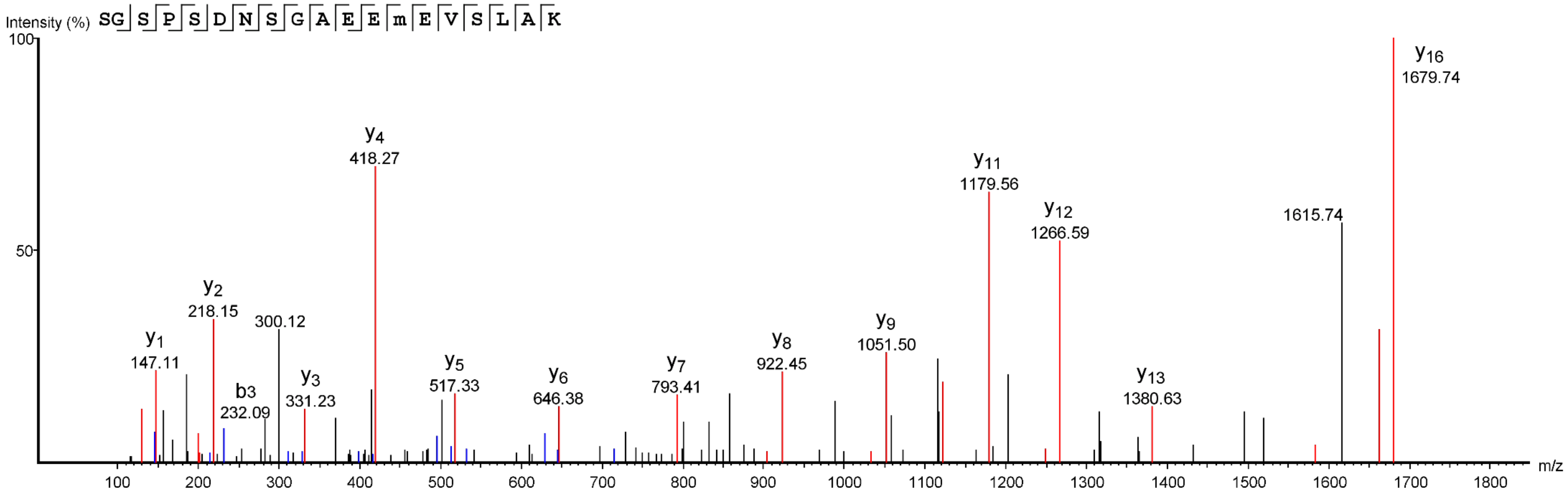

Pep17
Scan #6949

A-140: AKT1$_{(122-140)}$: SGSP(+15.99)SDNSGAEEM(+15.99)EVSLAK

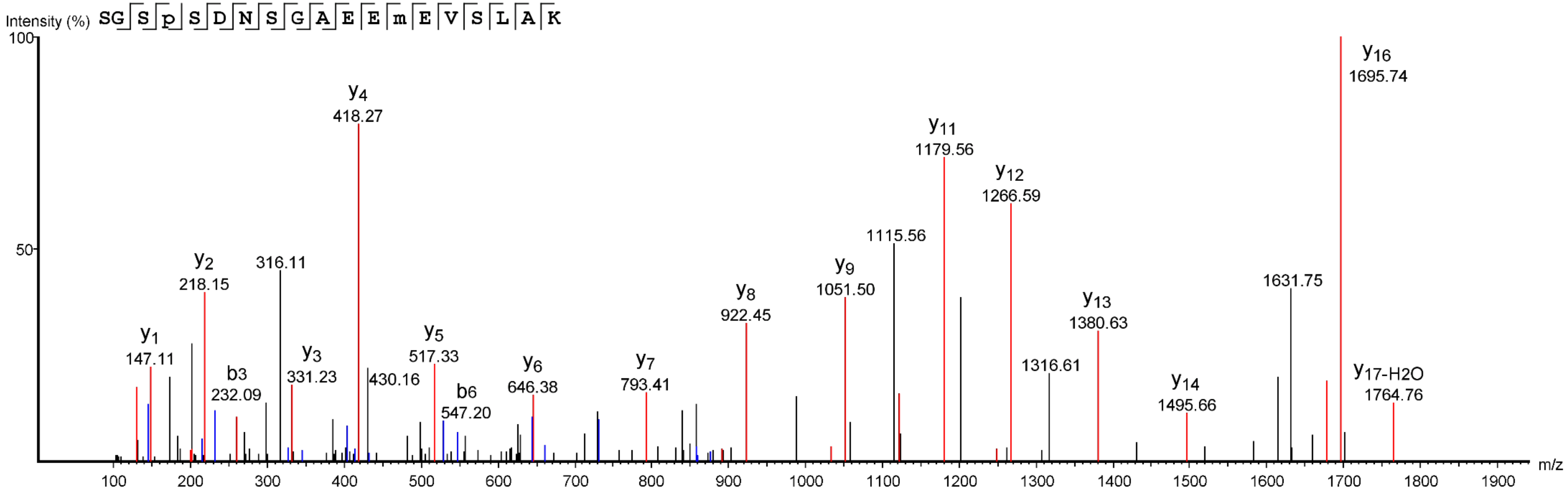

Pep17
Scan #6548

A-141: AKT1$_{(122-140)}$: SGSPSDNSGAEEMEVSLAK

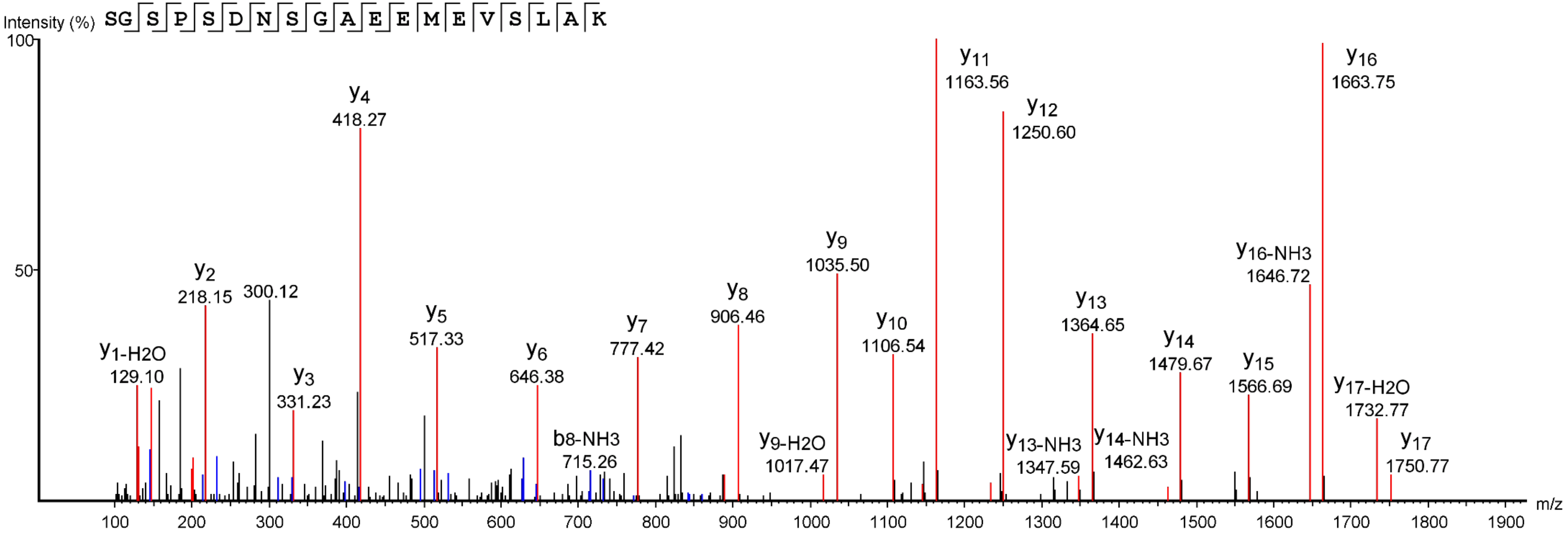

YMT83
Scan #12847

A-142: AKT1(122-140): SGSPSDNSGAEEMEVSLAK

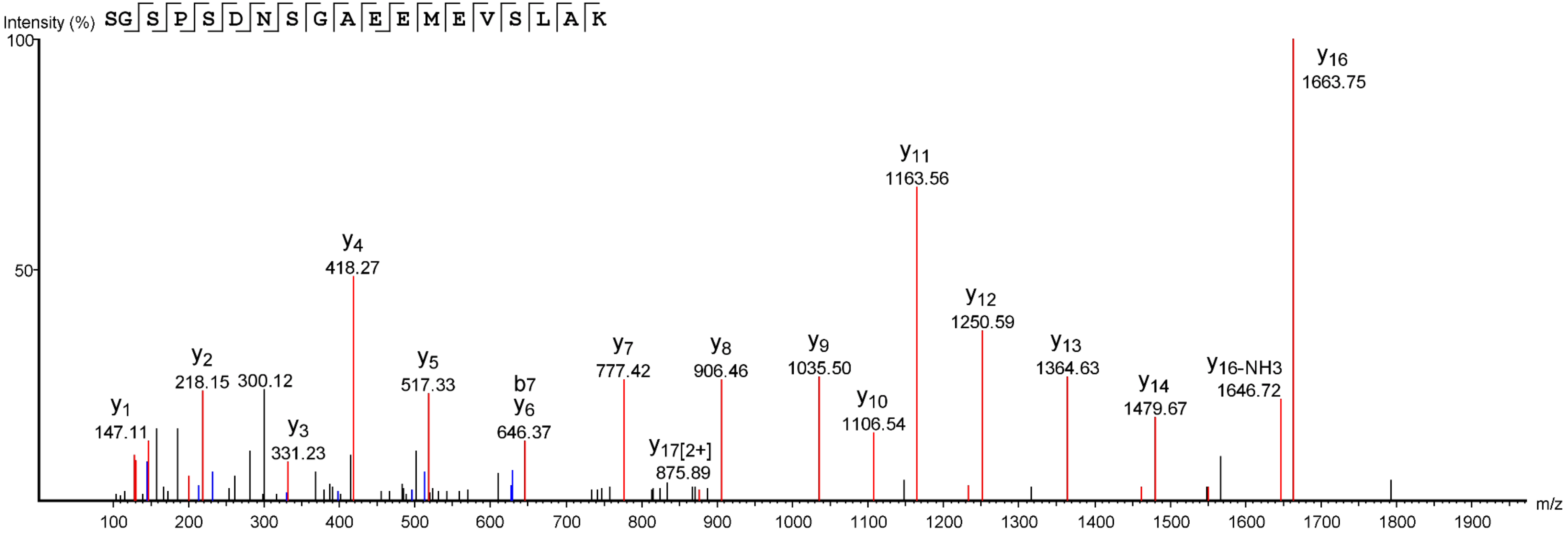

YMT84
Scan #12609

A-143: AKT1$_{(122-140)}$: SGSPSDNSGAEEM(+15.99)EVSLAK

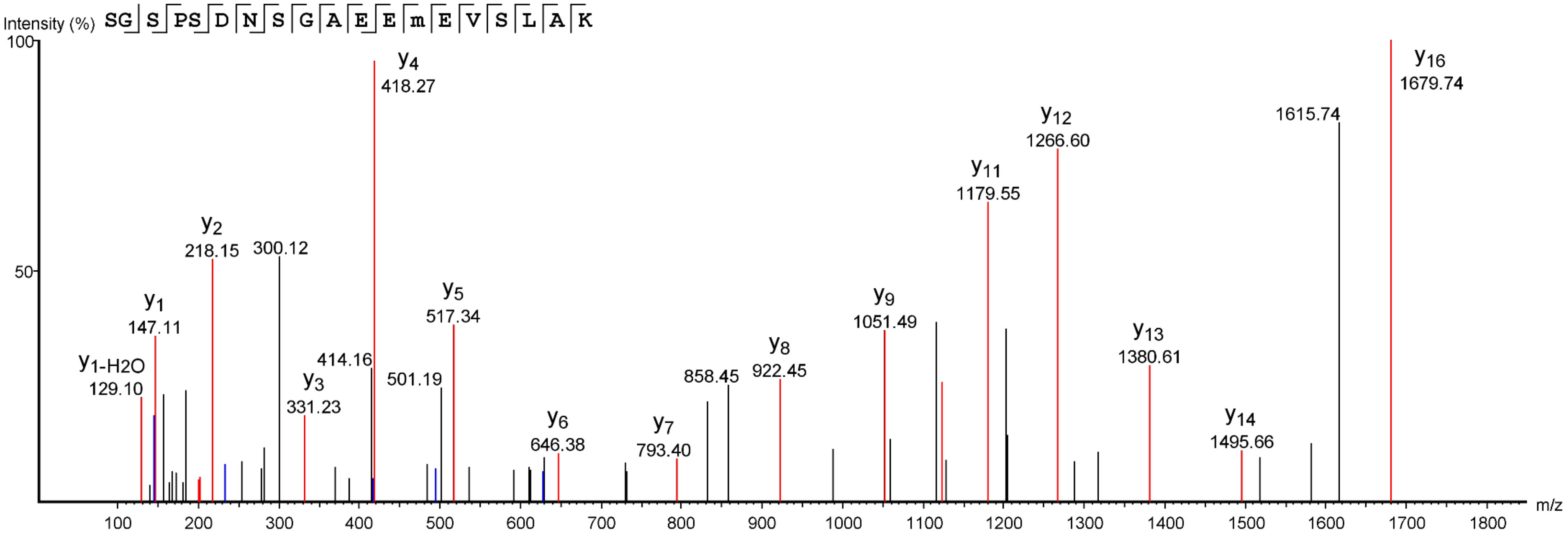

YMT83
Scan #8732

A-144: AKT1(122-140): SGSPSDNSGAEEM(+15.99)EVSLAK

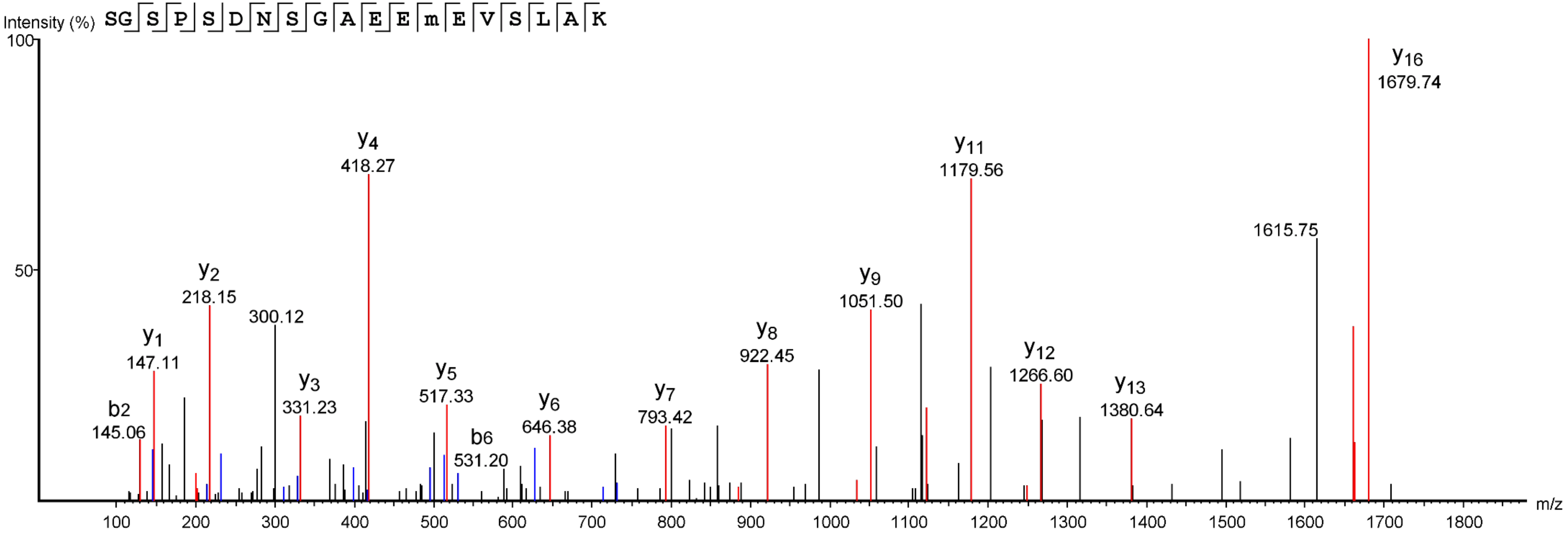

YMT84
Scan #8742

A-145: AKT1(308-328): TFC(+57.02)GTPEYLAPEVLEDNDYGR

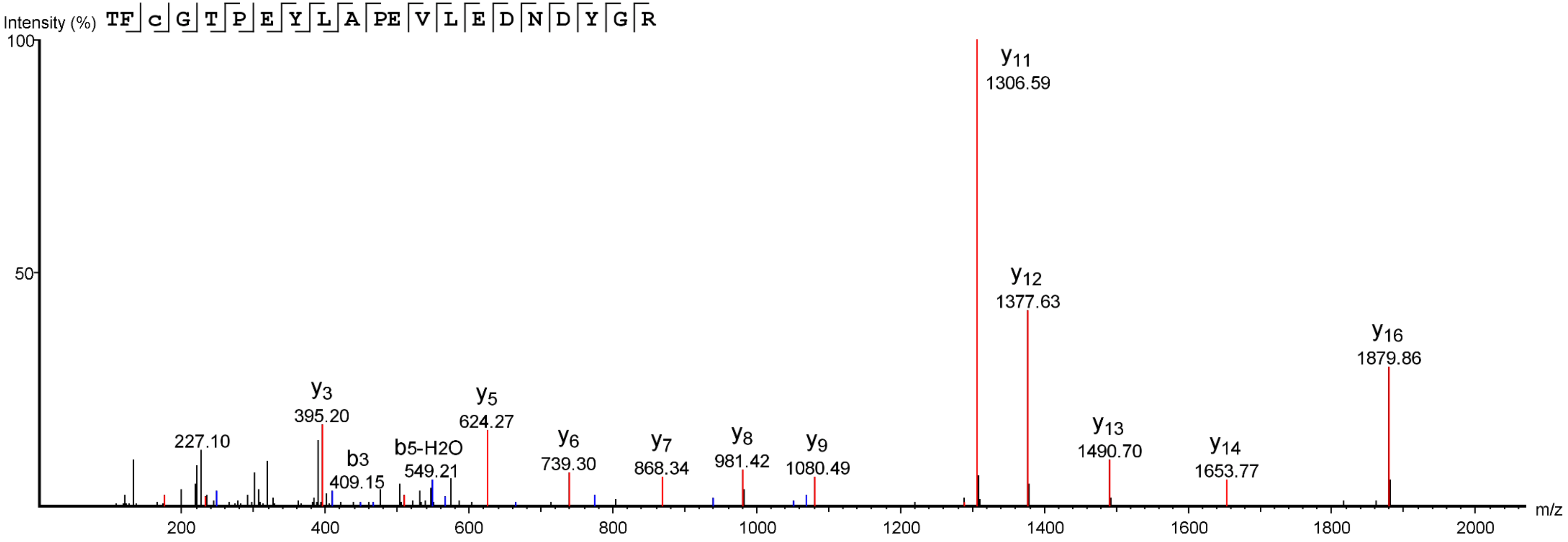

Pep17
Scan
#16799

A-146: AKT1$_{(308-328)}$: TFC(+57.02)GTP(+15.99)EYLAPEVLEDNDYGR

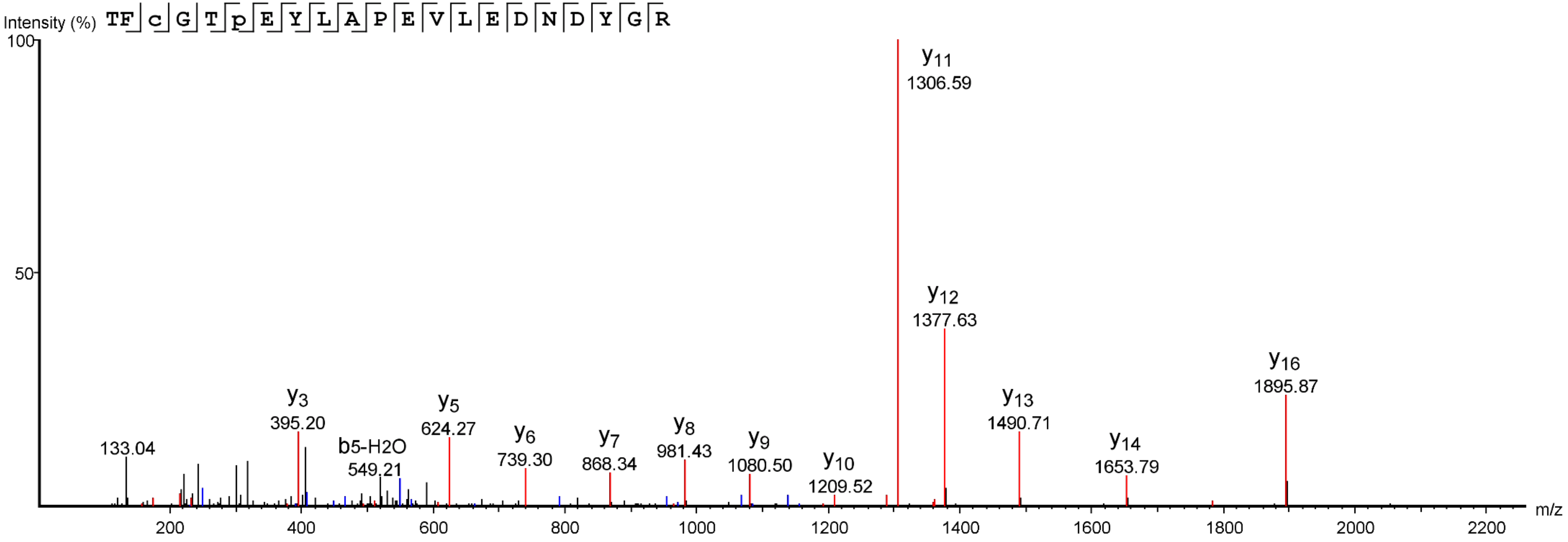

Pep17
Scan
#15407

A-147: AKT1(308-328): TFC(+57.02)GTPEYLAP(+15.99)EVLEDNDYGR

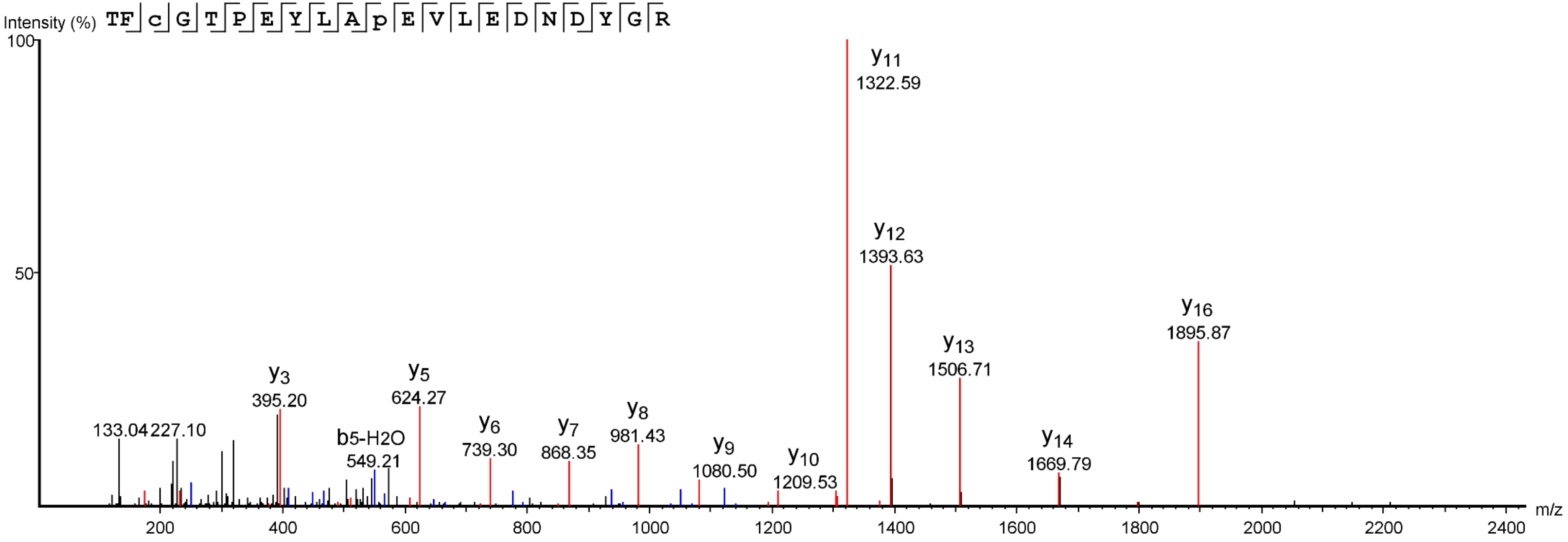

Pep17
Scan
#15119

A-148: AKT1$_{(308-328)}$: TFC(+57.02)GTP(+15.99)EYLAP(+15.99)EVLEDNDYGR

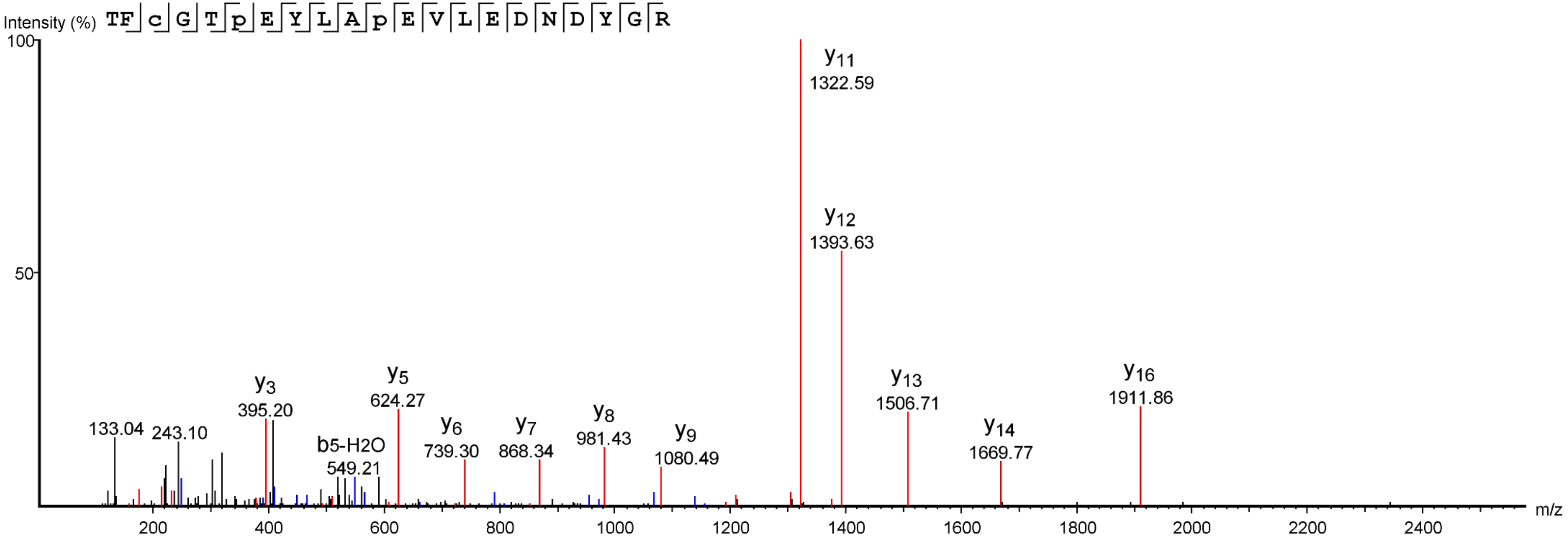

Pep17
Scan
#14031

A-151: AKT1$_{(421-436)}$: LSPPFKPQVTSETDTR+

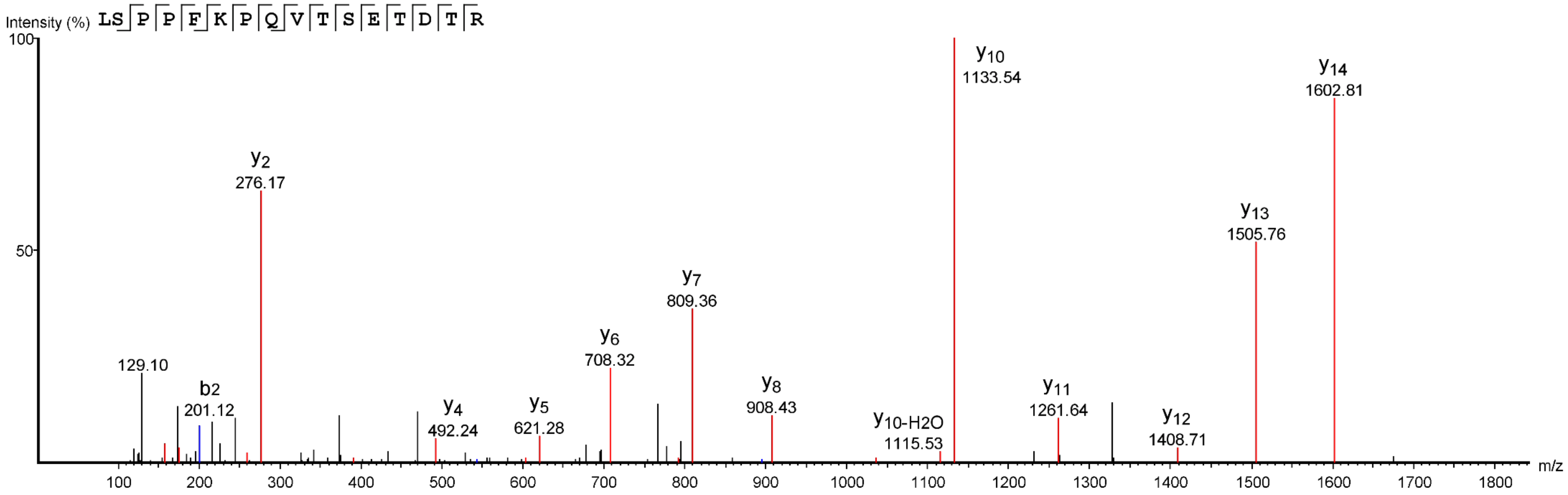

Pep17

Scan #8324

A-152: AKT1$_{(421-436)}$: LSPPFKP(+15.99)QVTSETDTR+

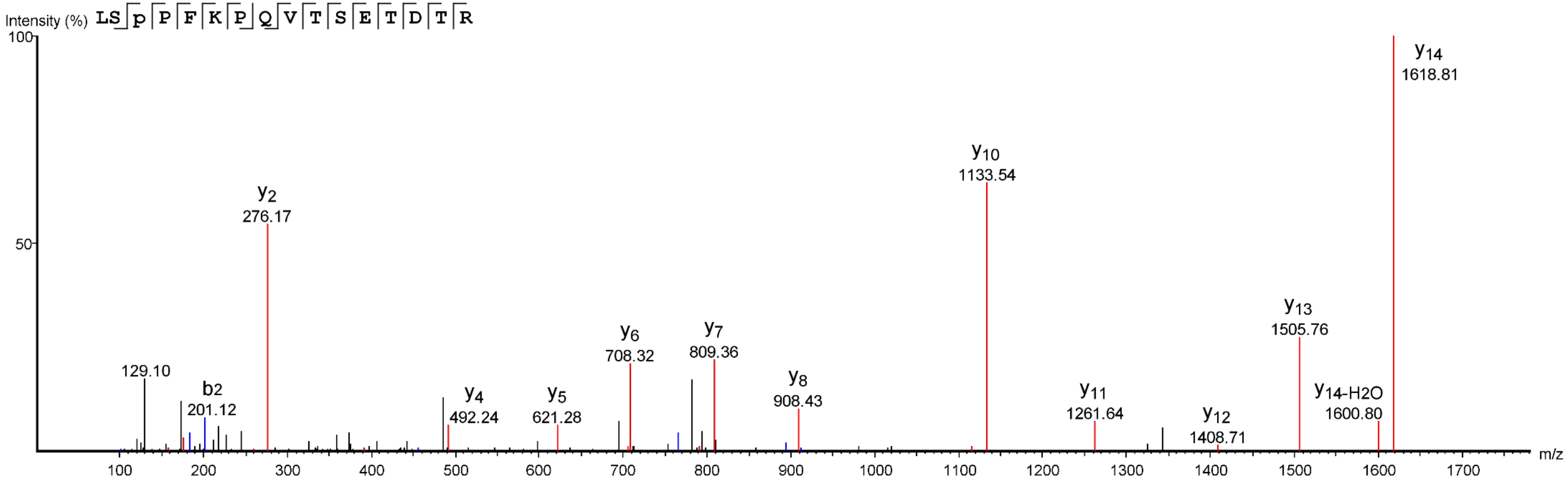

Pep17
Scan #6901

A-153: AKT1$_{(421-436)}$: LSPPFKPQVTSETDTR+

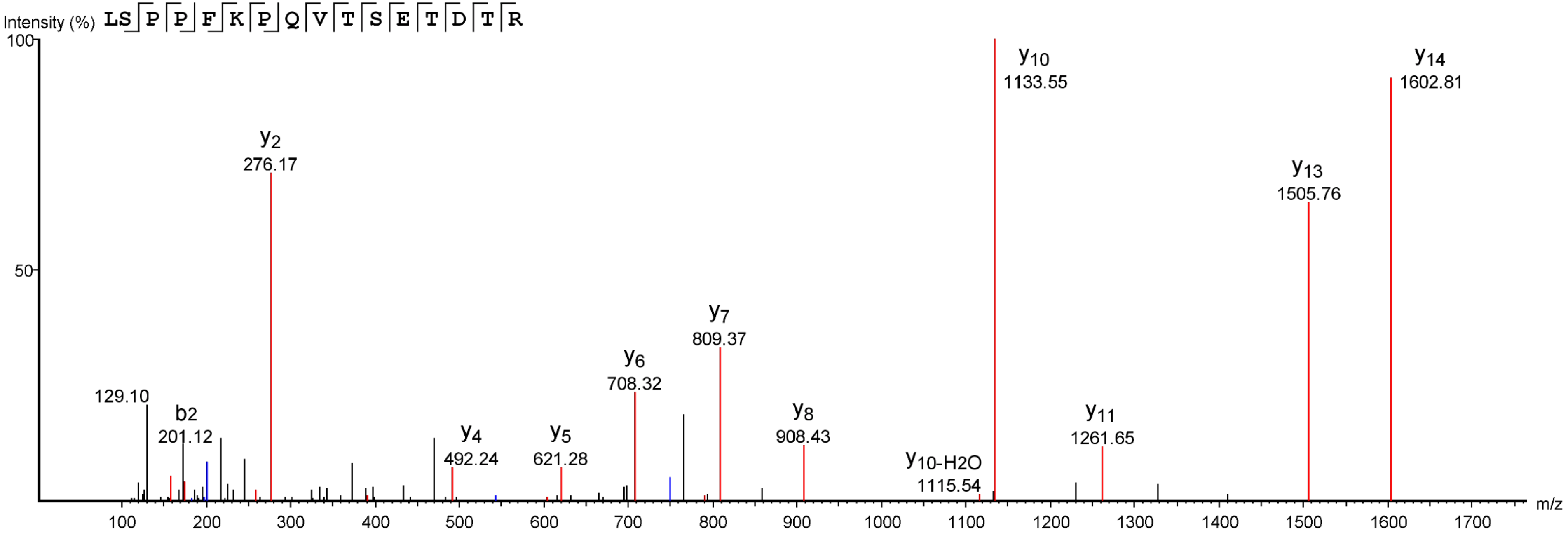

YMT83

Scan #10489

A-154: AKT1$_{(421-436)}$: LSPPFKPQVTSETDTR+

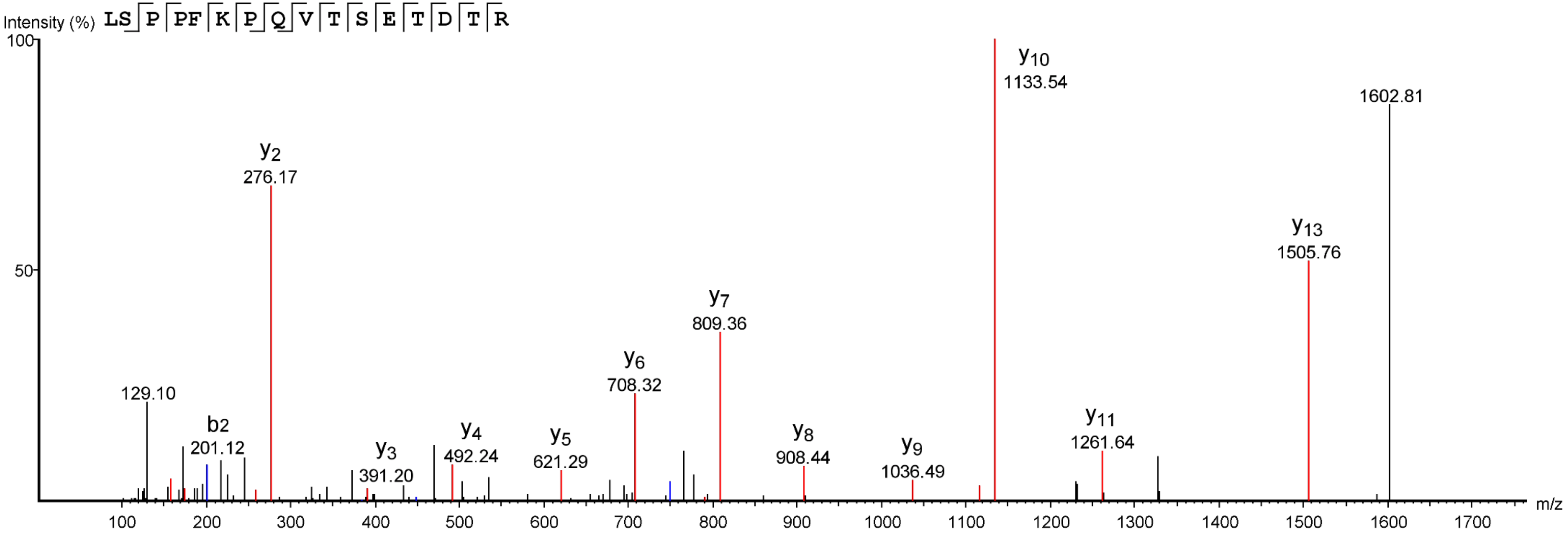

YMT84
Scan #10449

A-155: ATF4$_{(142-168)}$: GHLPESLTKPDQVAPFTFLQPLPLSPG+

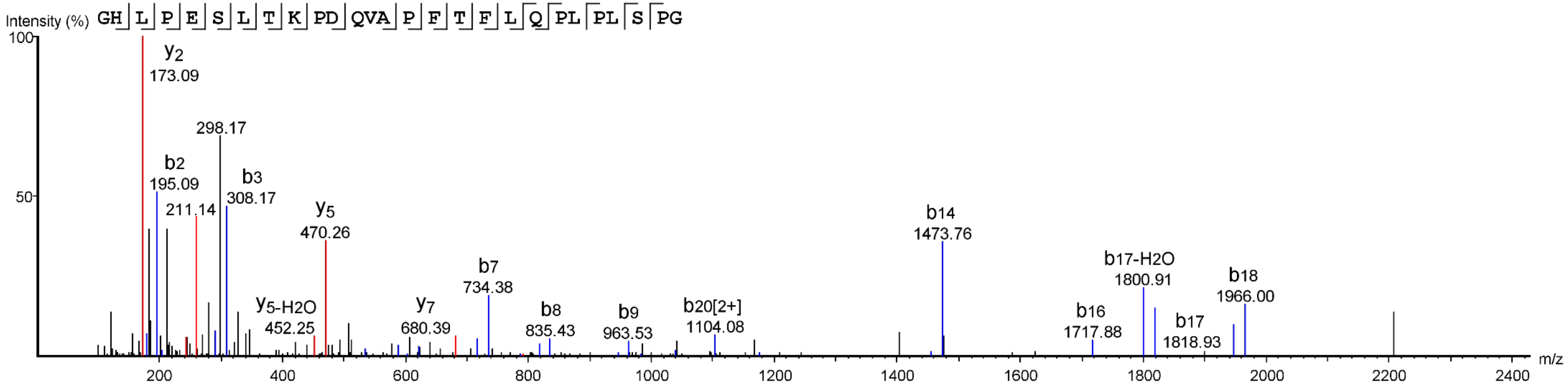

YMT45
Scan
#24007

A-156: ATF4$_{(142-168)}$: GHLPESLTKPDQVAPFTFLQPLPLSPG+

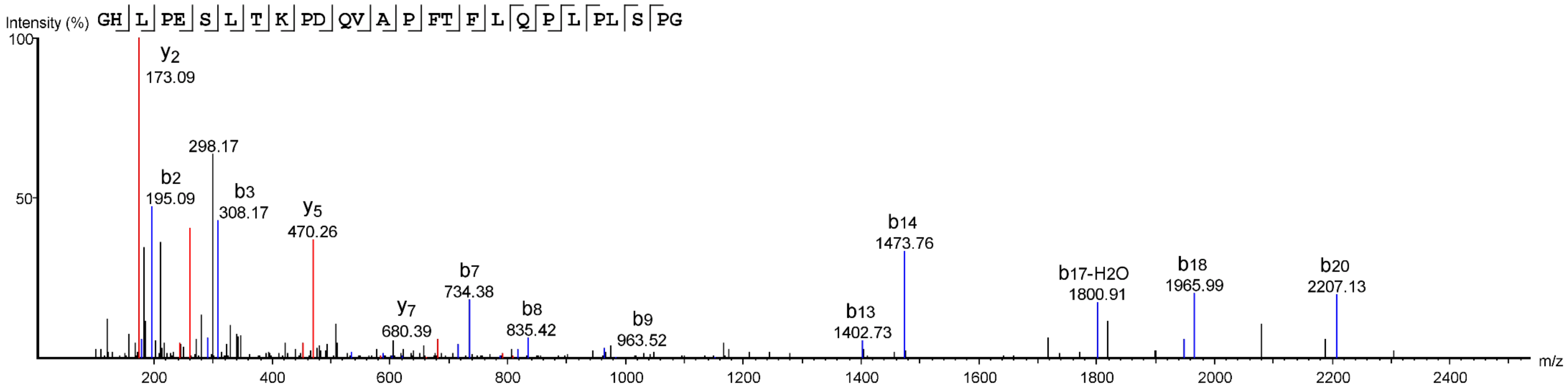

YMT46
Scan
#24252

A-157: ATF4$_{(172-198)}$: STPDHSFSLELGSEVDITEGDRKPDYT

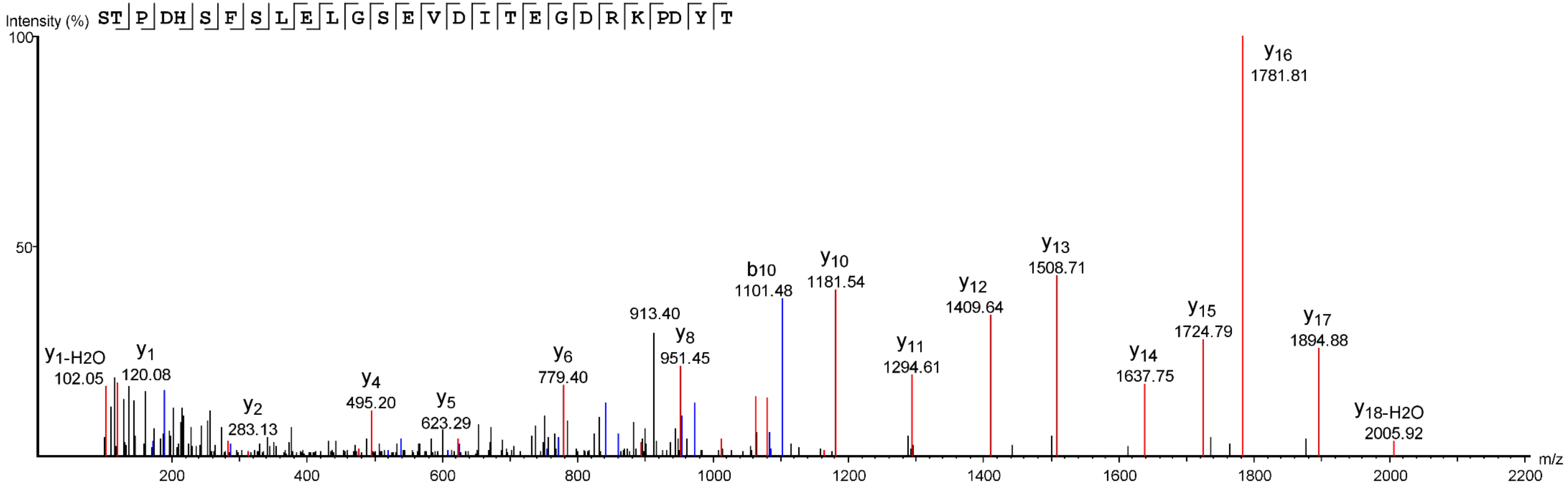

YMT45
Scan
#17806

A-158: ATF4(172-198): STPDHSFSLELGSEVDITEGDRKPDYT

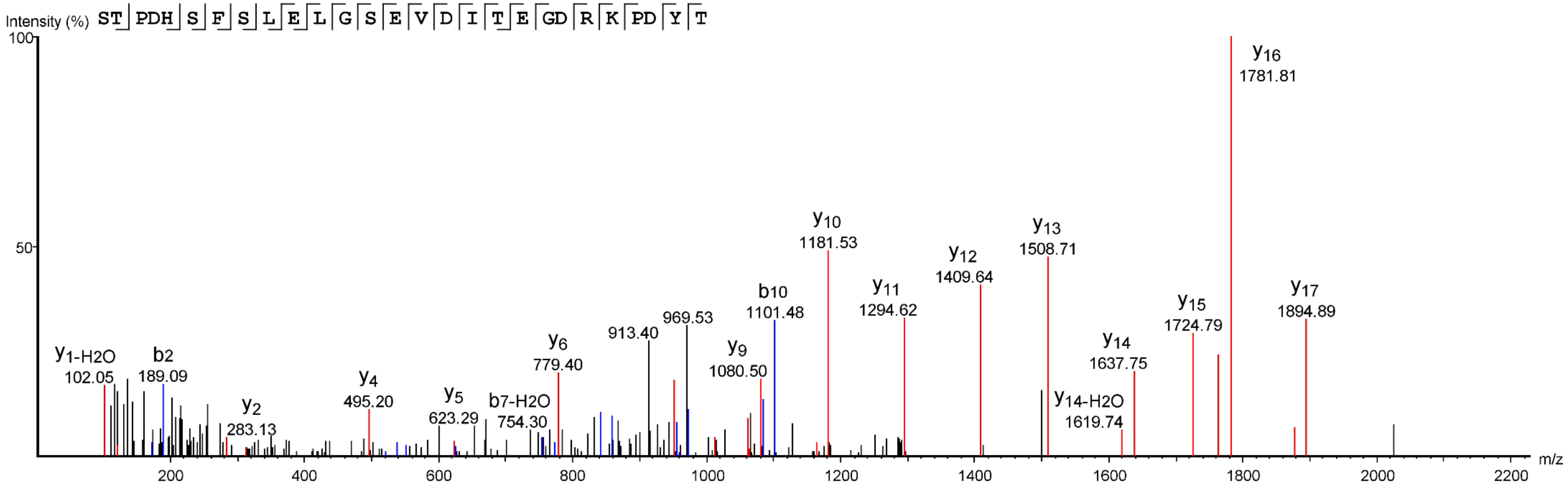

YMT46

Scan #

A-159: CENPN$_{(308-329)}$: SLAPAGIADAPLSPLLTC(+57.02)IPNKR

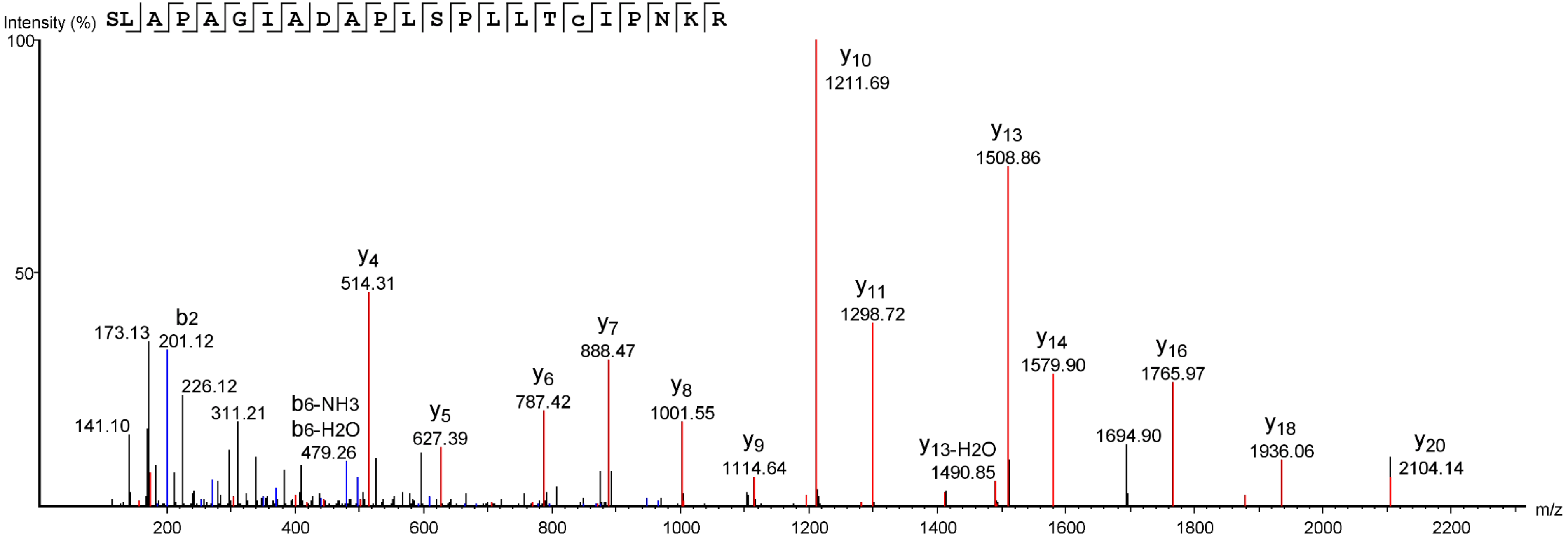

Pep14
Scan #15395

A-160: CENPN$_{(308-329)}$: SLAP(+15.99)AGIADAPLSPLLTC(+57.02)IPNKR

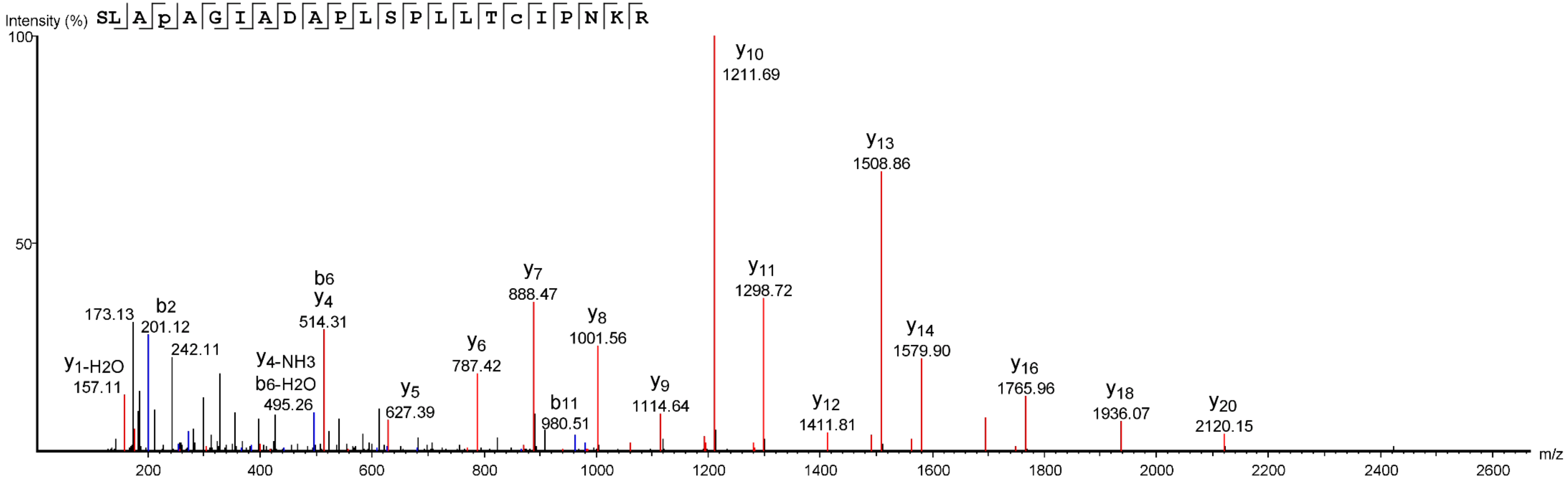

Pep14
Scan #14581

A-161: CENPN$_{(308-329)}$: SLAPAGIADAPLSPLLTC(+57.02)IPNKR

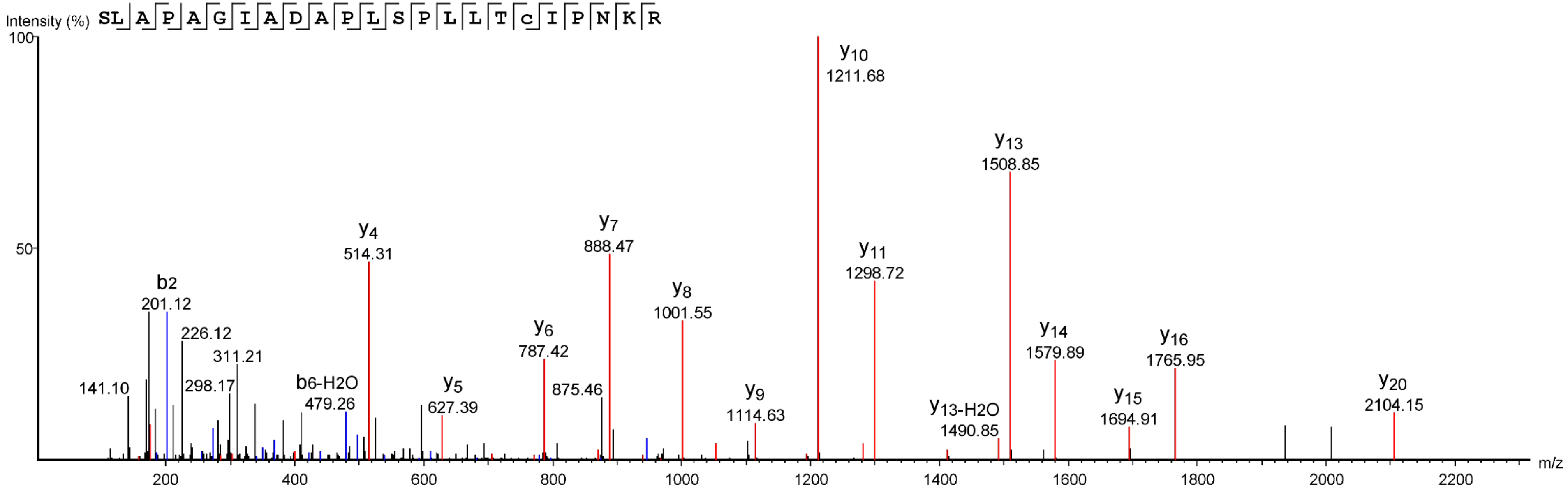

YMT39
Scan #20298

A-162: CENPN$_{(308-329)}$: SLAPAGIADAPLSPLLTC(+57.02)IPNKR

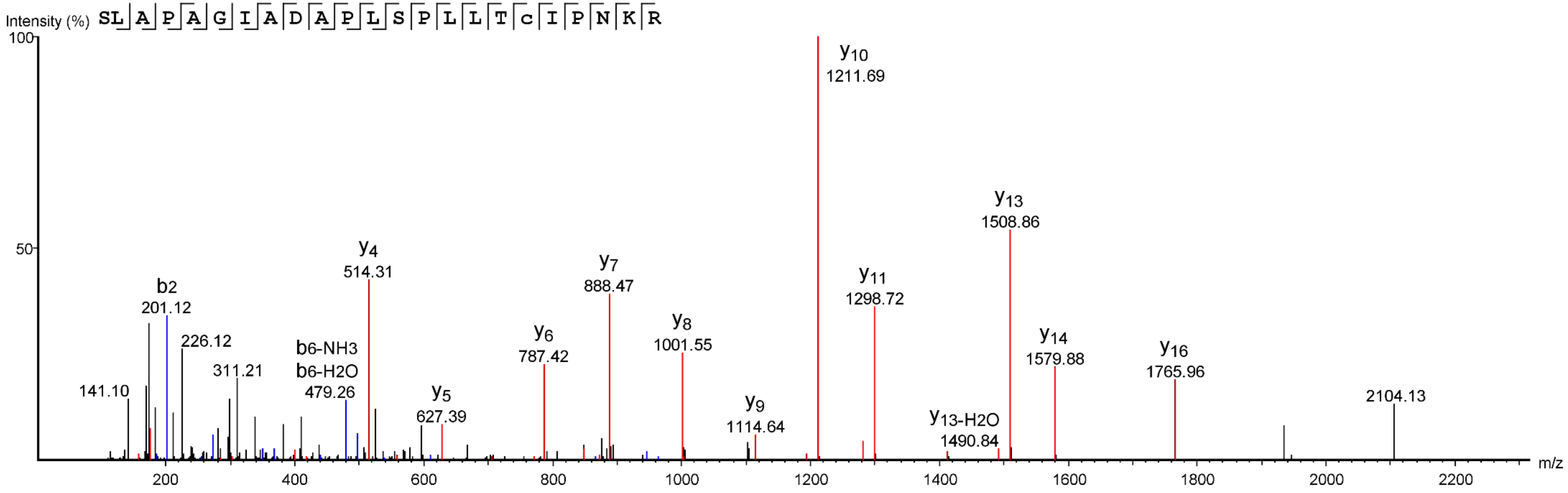

YMT40
Scan #19546

A-163: CEP192$_{(1710-1721)}$: WHLSSLAPPYVK

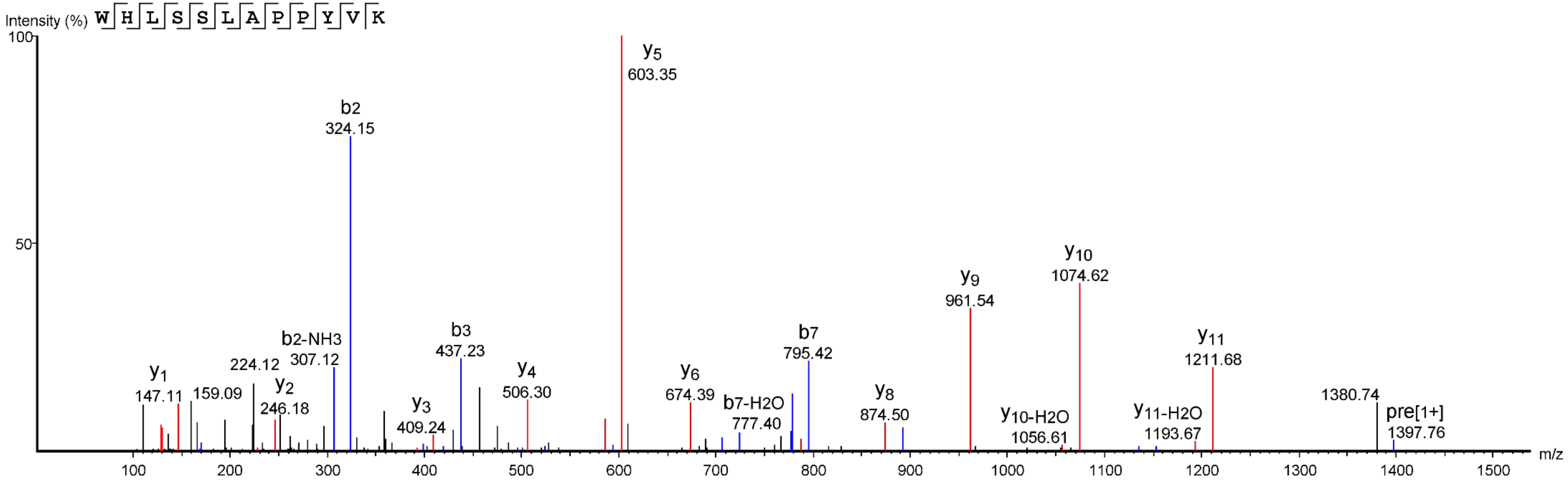

Pep11

Scan #13349

A-164: CEP192$_{(1710-1721)}$: WHLSSLAP(+15.99)PYVK

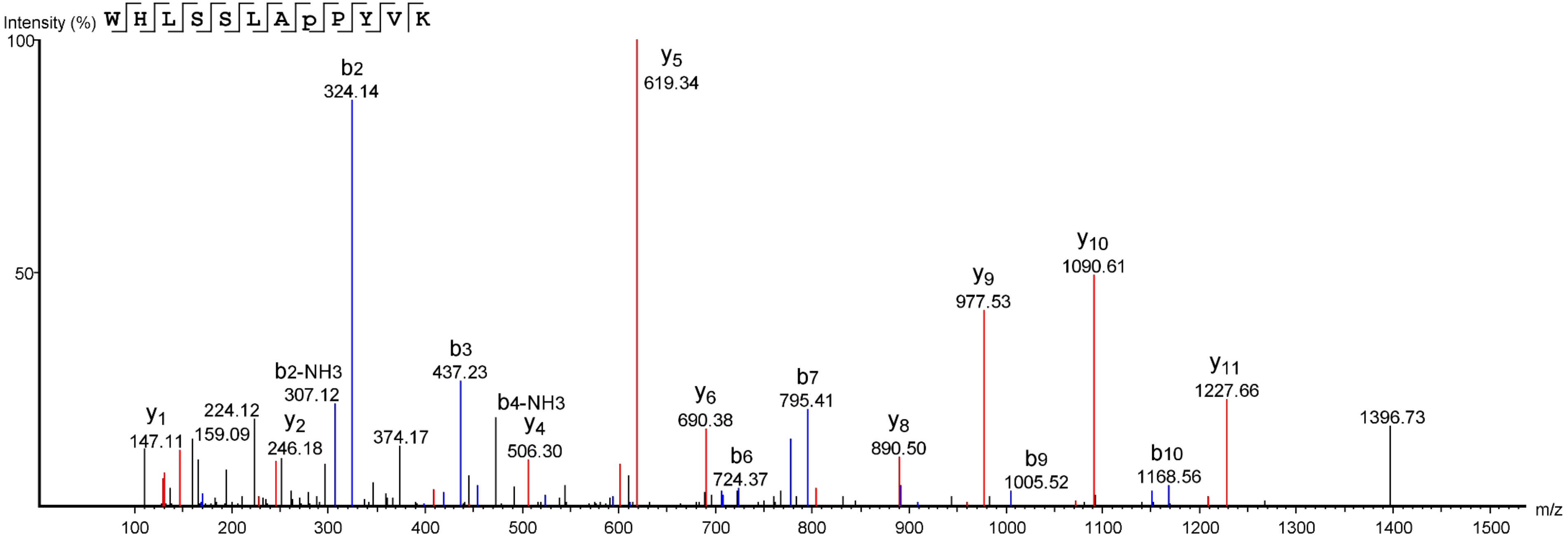

Pep11

Scan #11317

A-165: $CEP192_{(1710-1721)}$: WHLSSLAPPYVK

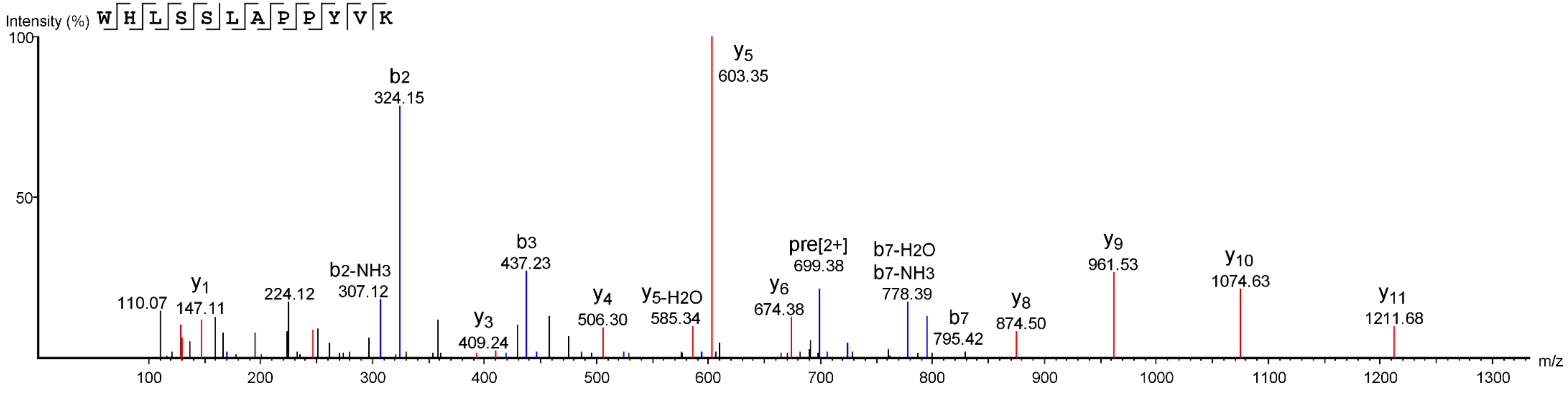

YMT61

Scan #12668

A-166: CEP192$_{(1710-1721)}$: WHLSSLAPPYVK

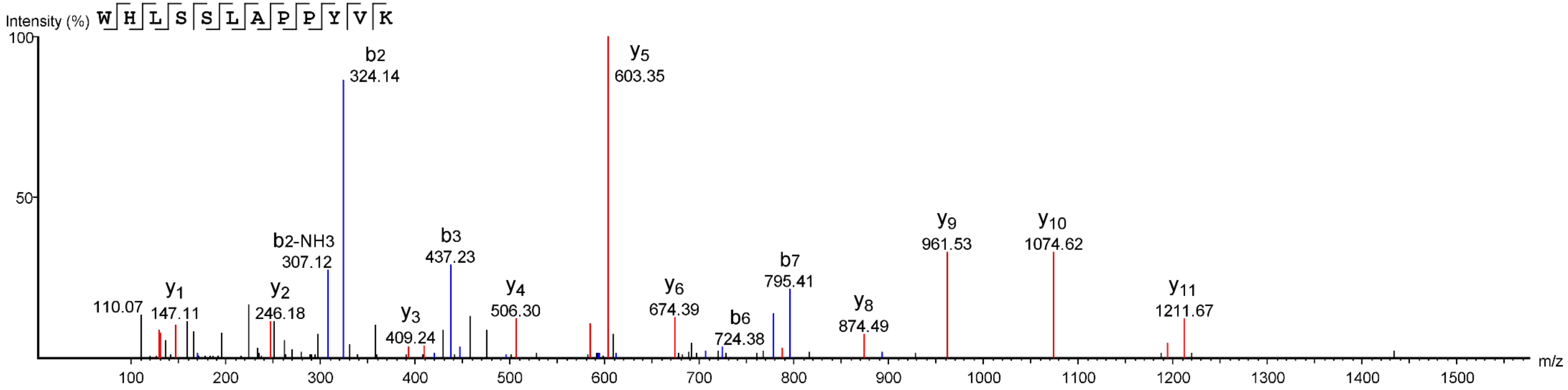

YMT62
Scan #12324

A-167: EEF2K$_{(94-111)}$: HMPDPWAEFHLEDIATER

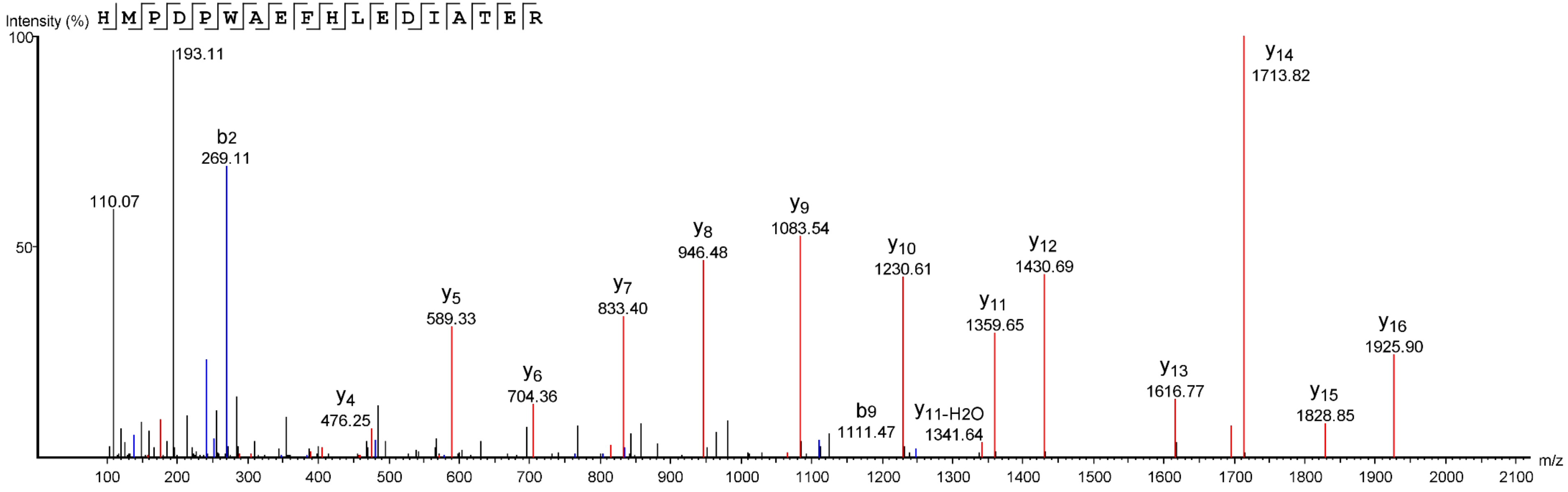

Pep17
Scan
#15551

A-168: EEF2K$_{(94\text{-}111)}$: HM(+15.99)PDPWAEFHLEDIATER

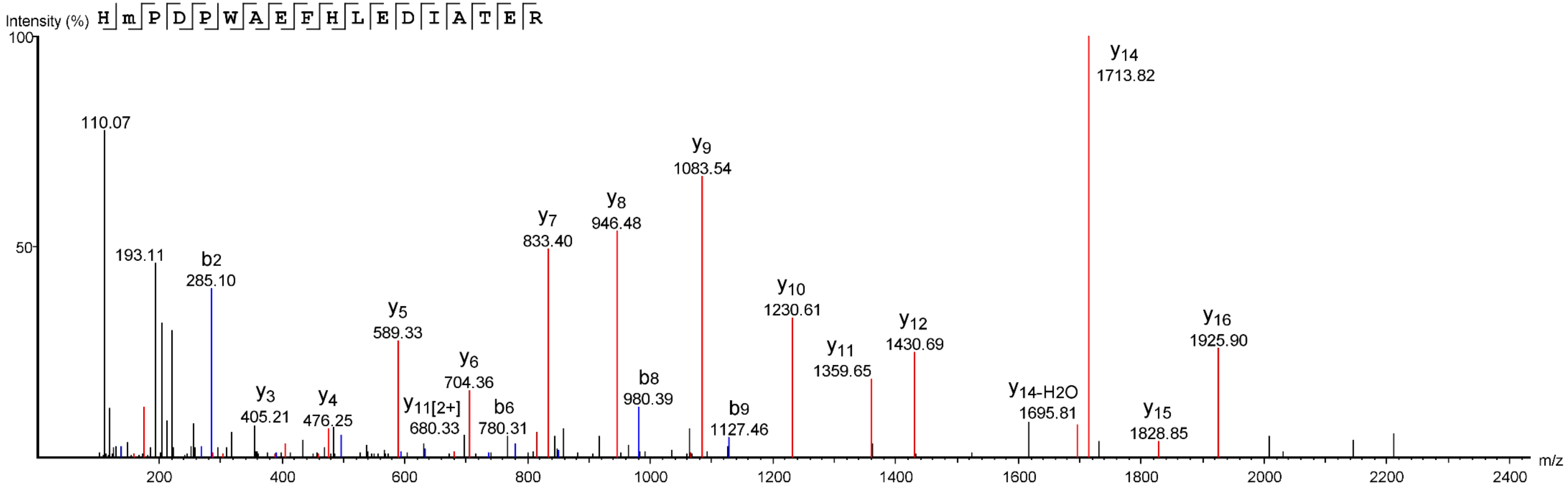

Pep17
Scan
#14303

A-169: EEF2K$_{(94-111)}$: HMPDP(+15.99)WAEFHLEDIATER

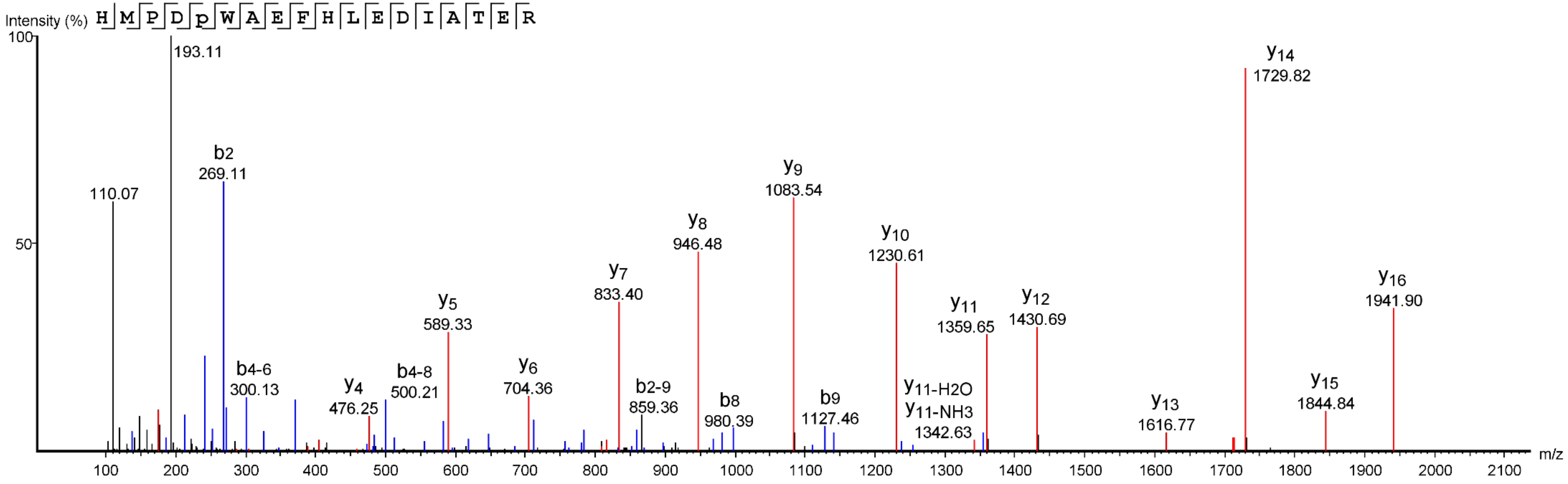

Pep17
Scan
#14096

A-170: EEF2K$_{(94-111)}$: HM(+15.99)PDP(+15.99)WAEFHLEDIATER

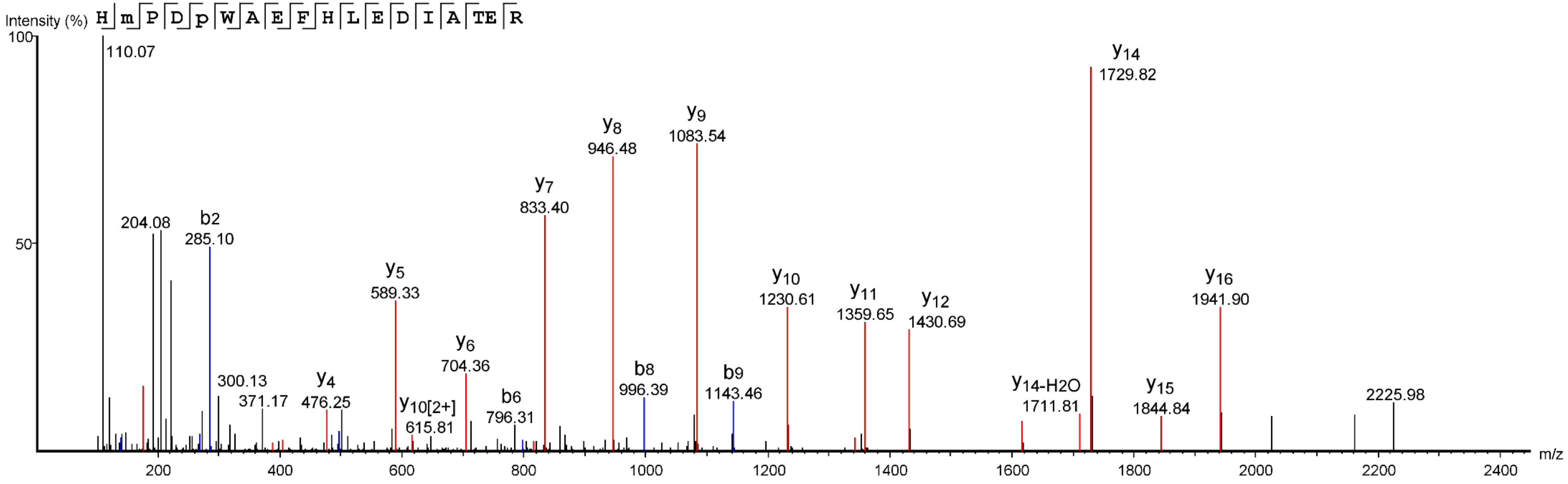

Pep17
Scan
#13291

A-171: EEF2K$_{(94\text{-}111)}$: HMPDPWAEFHLEDIATER

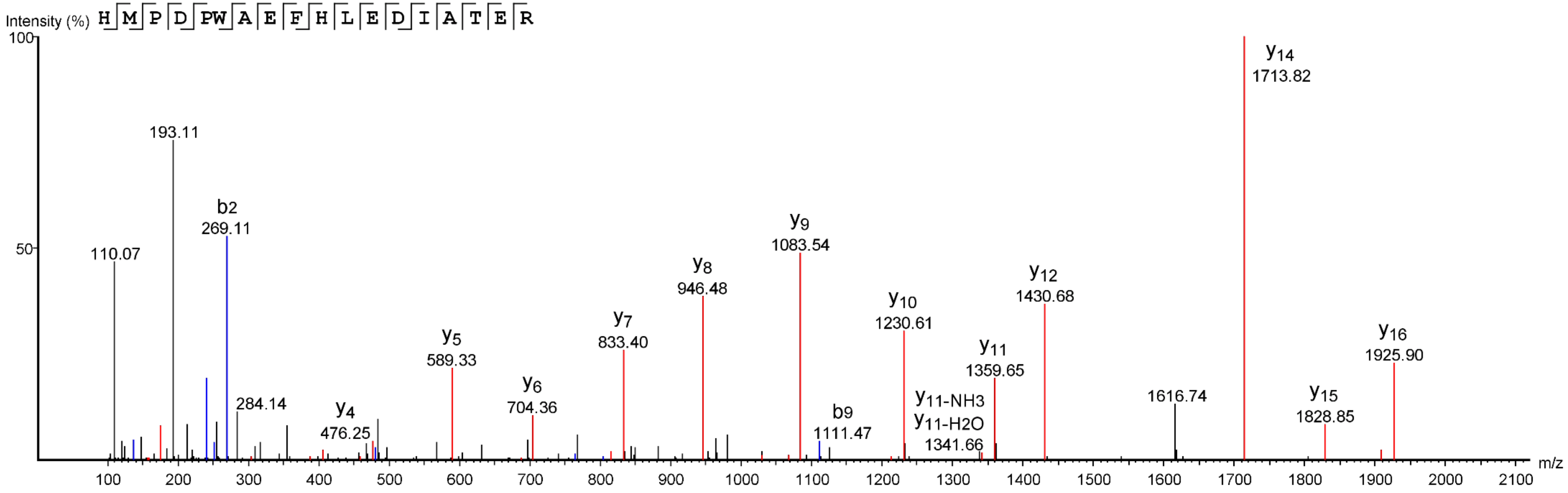

YMT69
Scan
#17085

A-172: EEF2K(94-111): HMPDPWAEFHLEDIATER

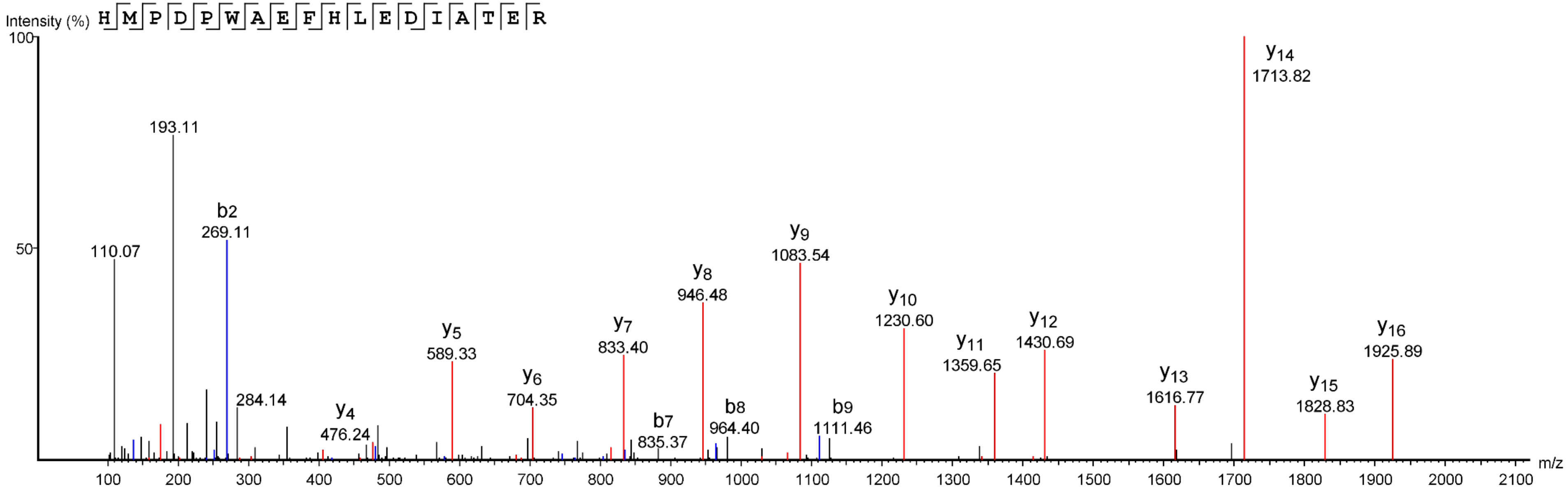

YMT70
Scan
#17382

A-173: EEF2K$_{(94-111)}$: HM(+15.99)PDPWAEFHLEDIATER

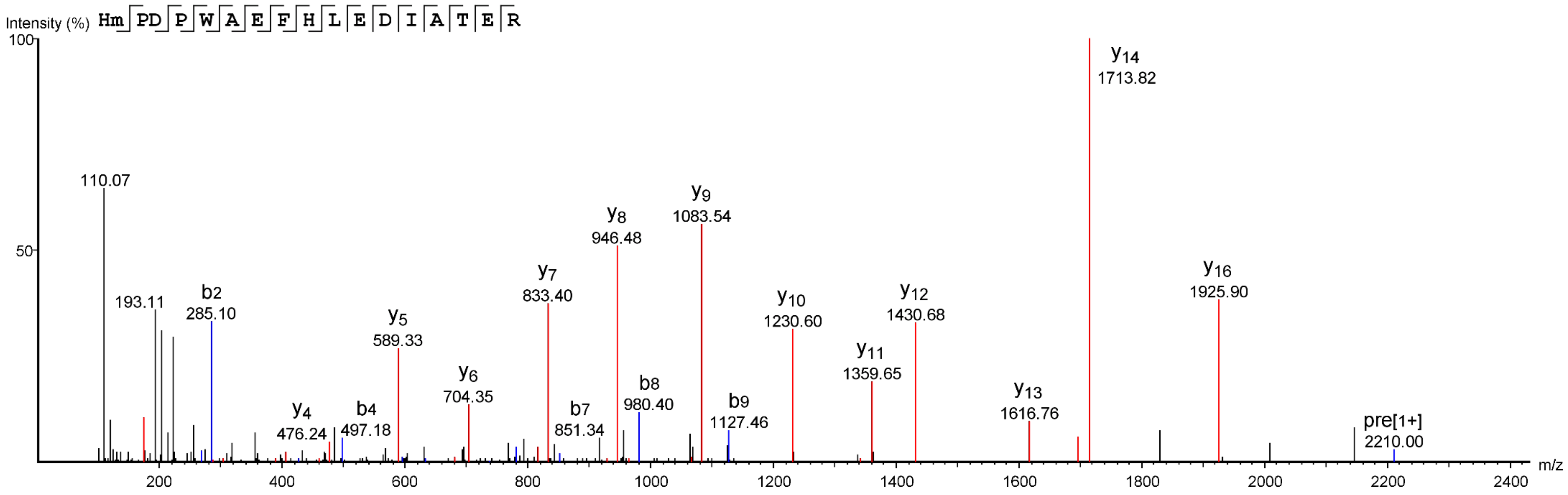

YMT69
Scan
#15852

A-174: EEF2K$_{(94\text{-}111)}$: HM(+15.99)PDPWAEFHLEDIATER

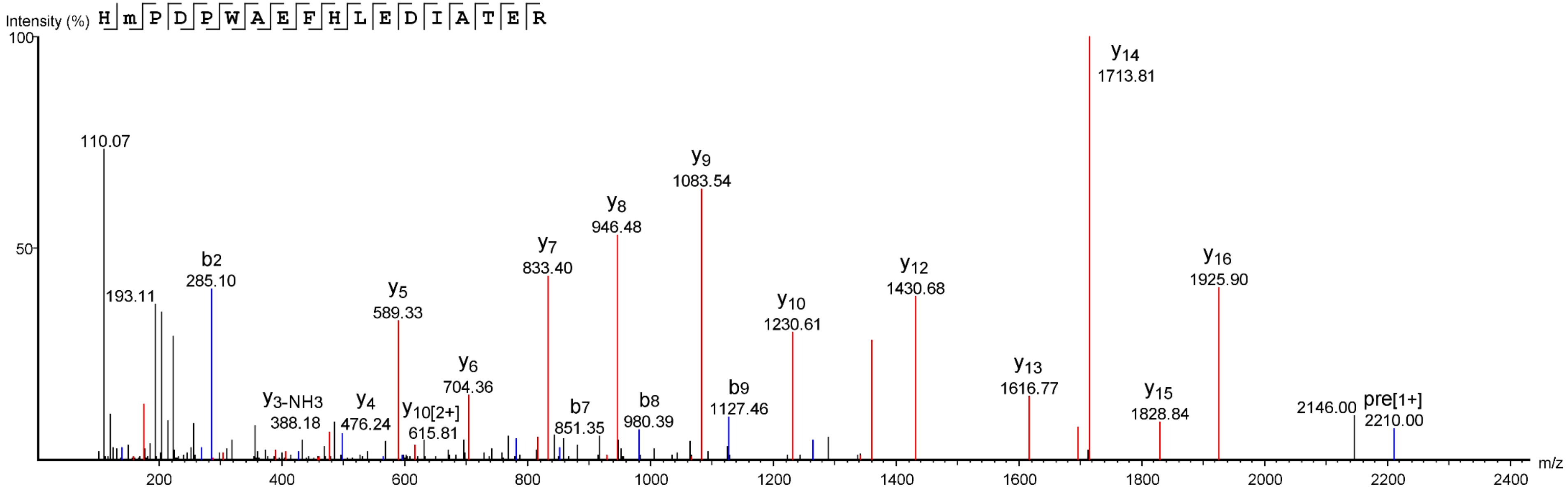

YMT70
Scan
#15909

A-175: EPOR$_{(426-453)}$: YTILDPSSQLLRPWTLC(+57.02)PELPPTPPHLK

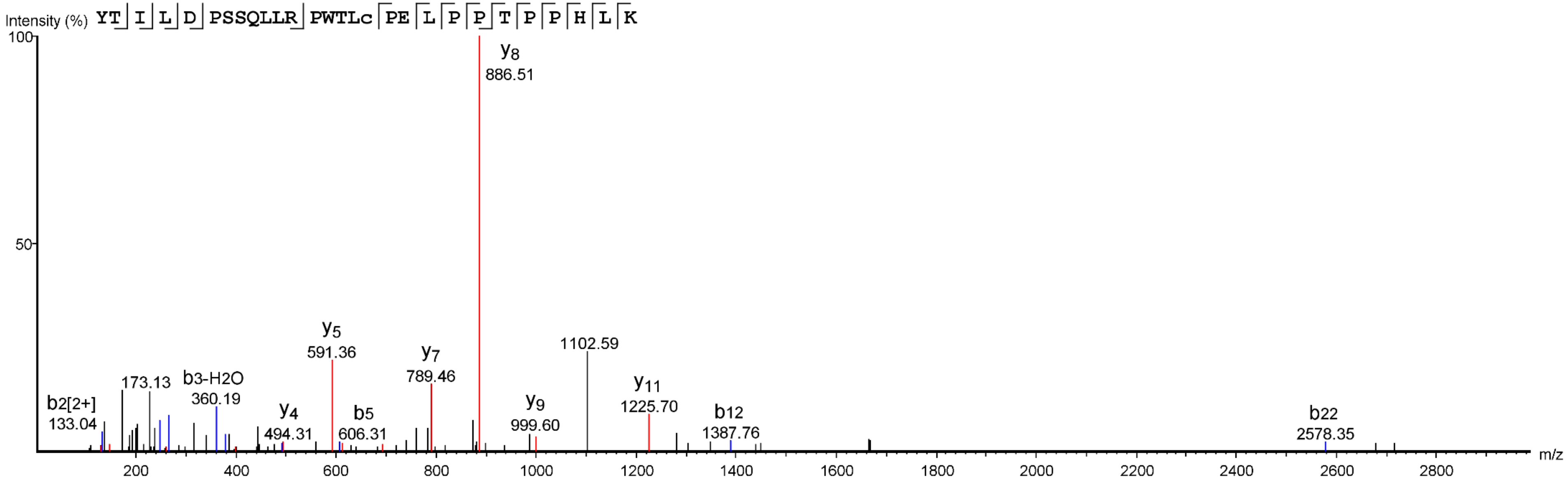

Pep14
Scan #18555

A-176: EPOR(426-453): YTILDPSSQLLRPWTLC(+57.02)P(+15.99)ELPPTPPHLK

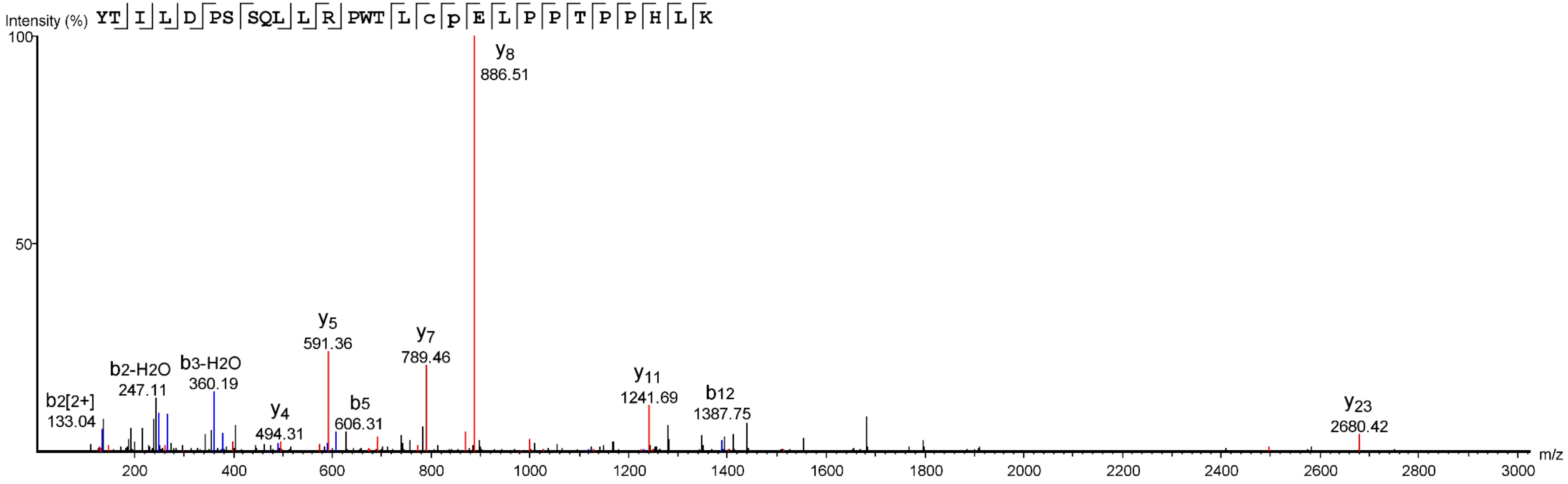

Pep14
Scan #17203

A-177: EPOR$_{(426\text{-}453)}$: YTILDPSSQLLRPWTLC(+57.02)PELPPTPP(+15.99)HLK

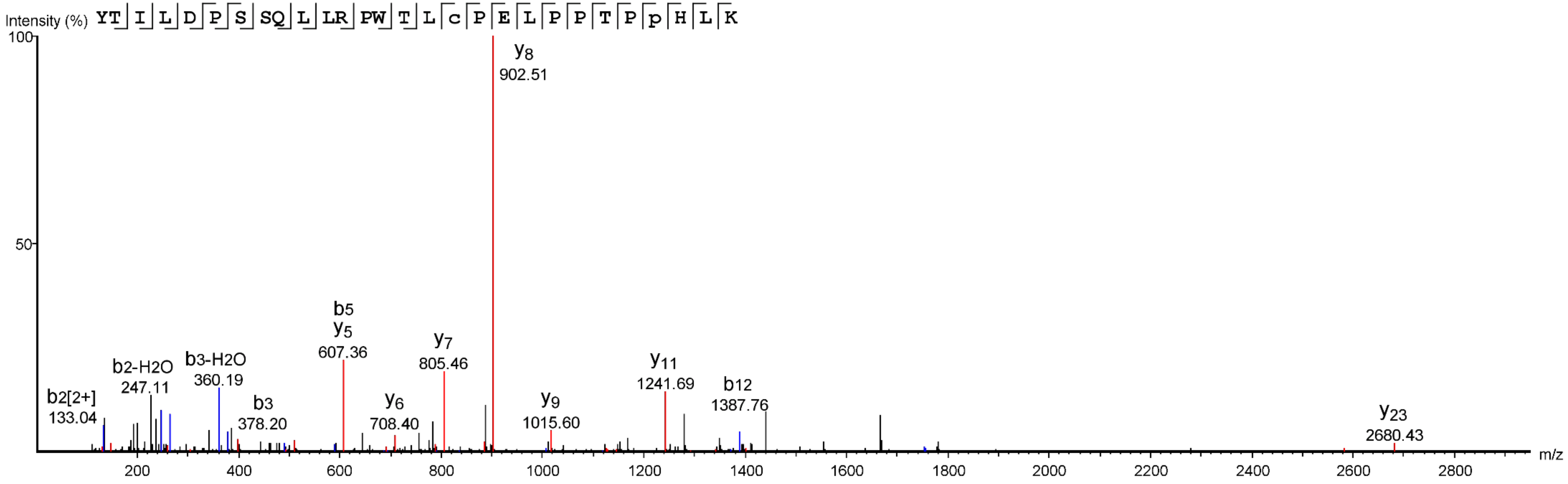

Pep14
Scan #17651

A-178: EPOR(426-453): YTILDPSSQLLRPWTLC(+57.02)P(+15.99)ELPPTPP(+15.99)HLK

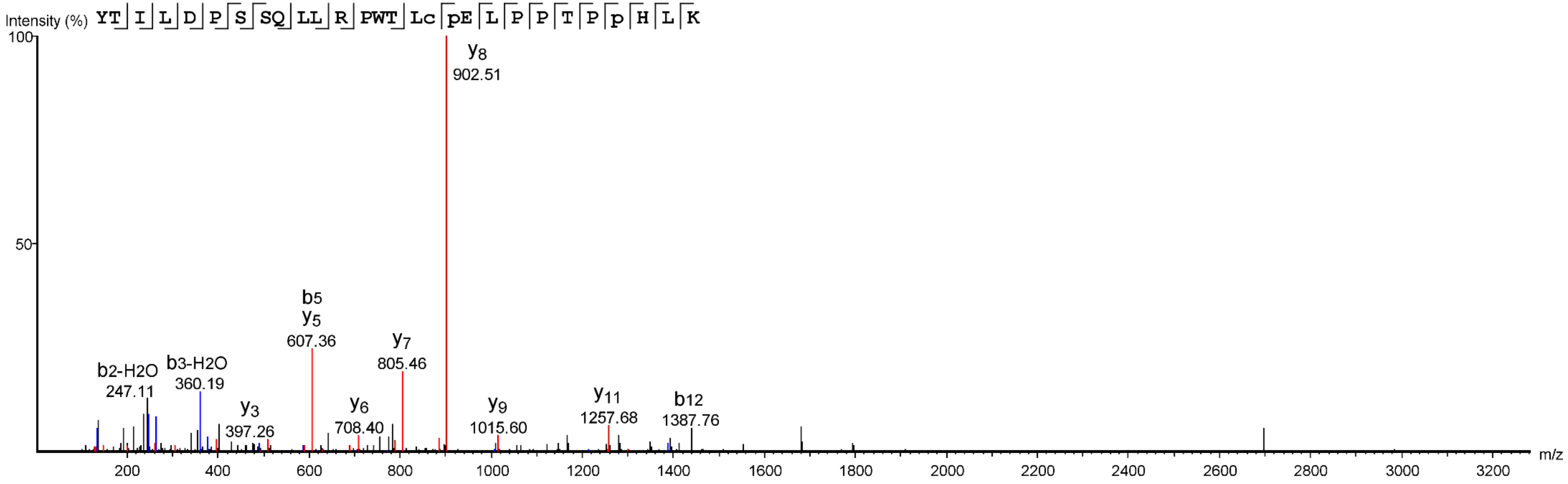

Pep14
Scan #16787

A-183: FLNA$_{(2311-2333)}$: FNEEHIPDSPFVVPVASPSGDAR+

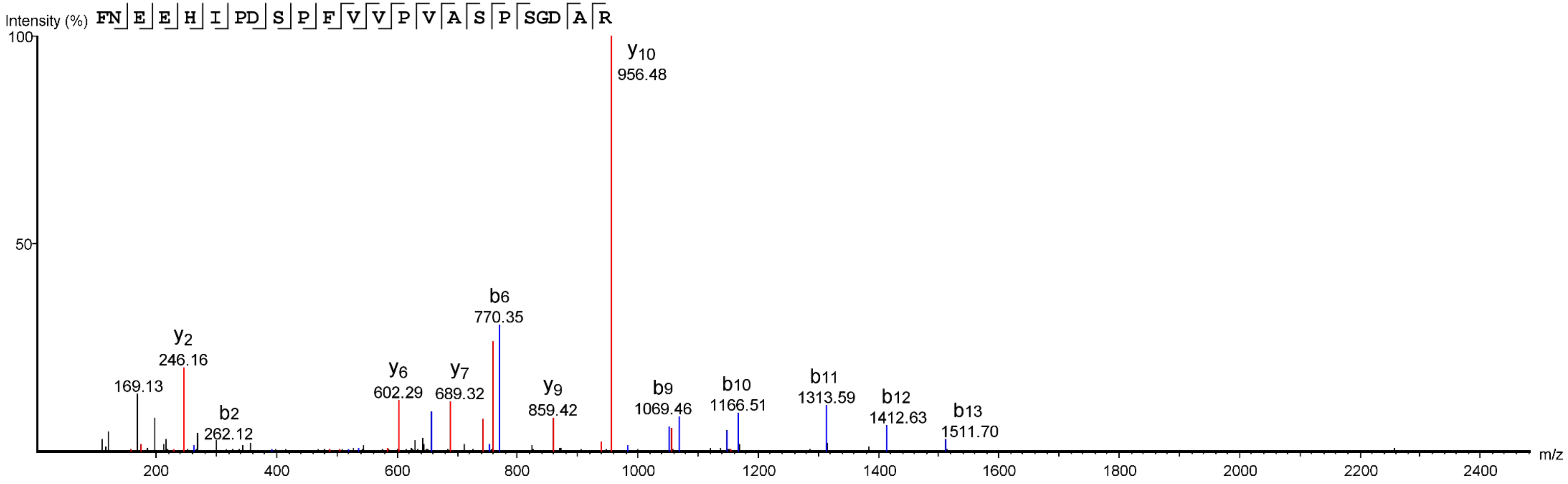

Pep20
Scan #8685

A-184: FLNA$_{(2311-2333)}$: FNEEHIP(+15.99)DSPFVVPVASPSGDAR+

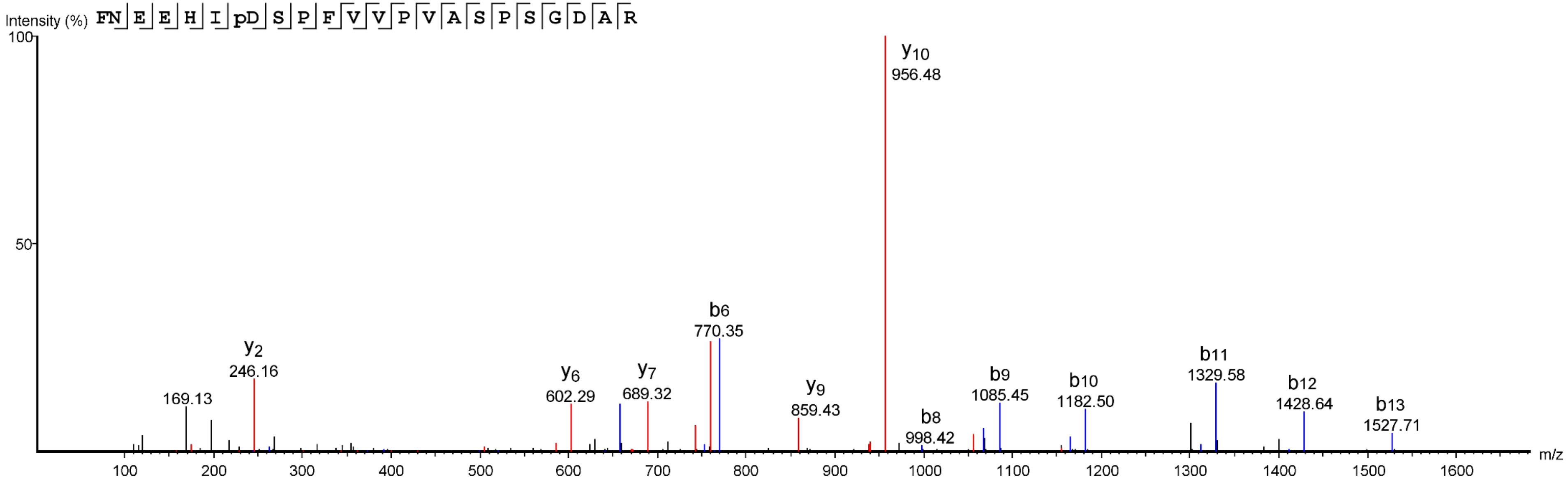

Pep20
Scan #8161

A-185: FLNA$_{(2311-2333)}$: FNEEHIPDSPFVVP(+15.99)VASPSGDAR+

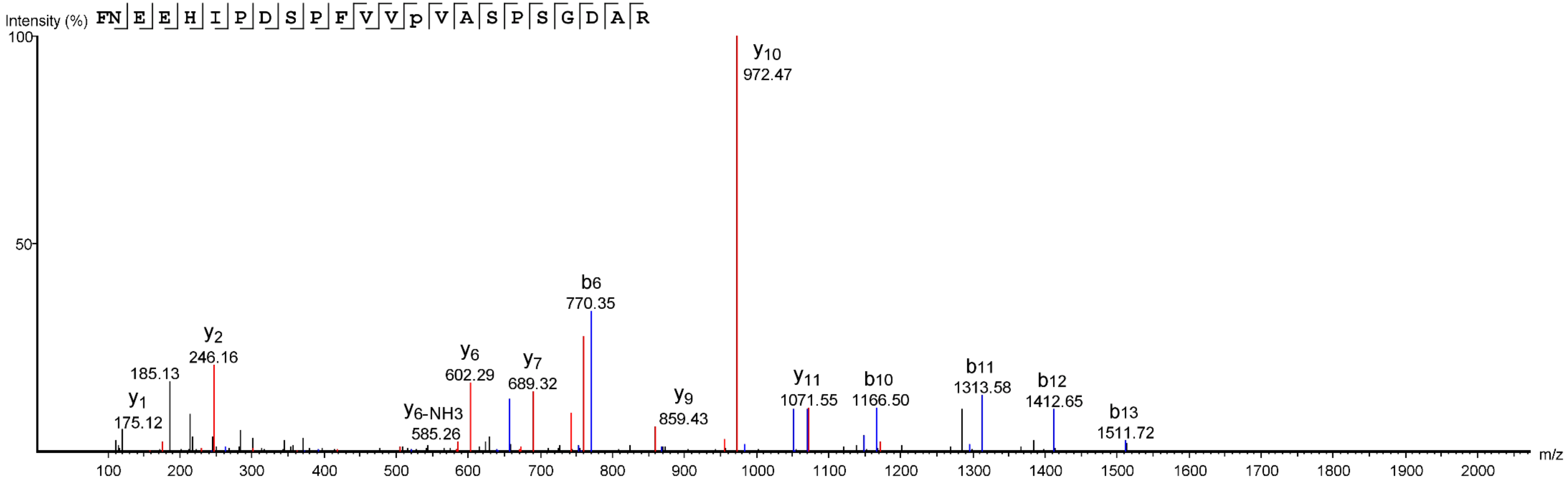

Pep20
Scan #7848

A-186: FLNA(2311-2333): FNEEHIP(+15.99)DSPFVVP(+15.99)VASPSGDAR+

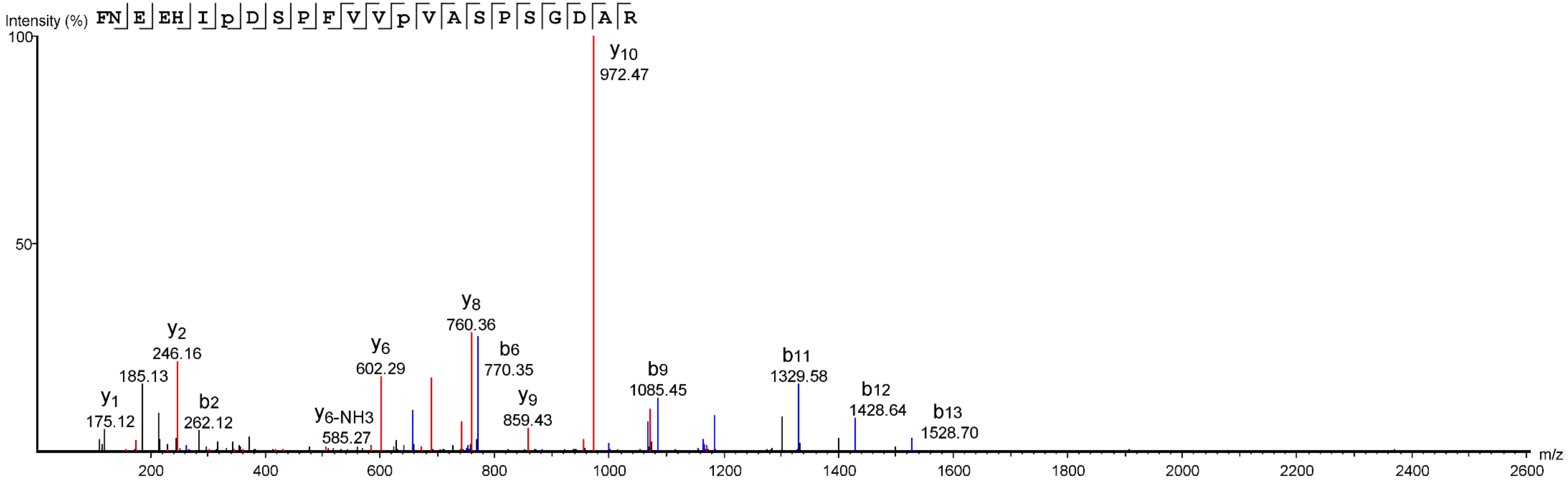

Pep20
Scan #7563

A-187: FLNA$_{(2311-2333)}$: FNEEHIPDSPFVVPVASPSGDAR+

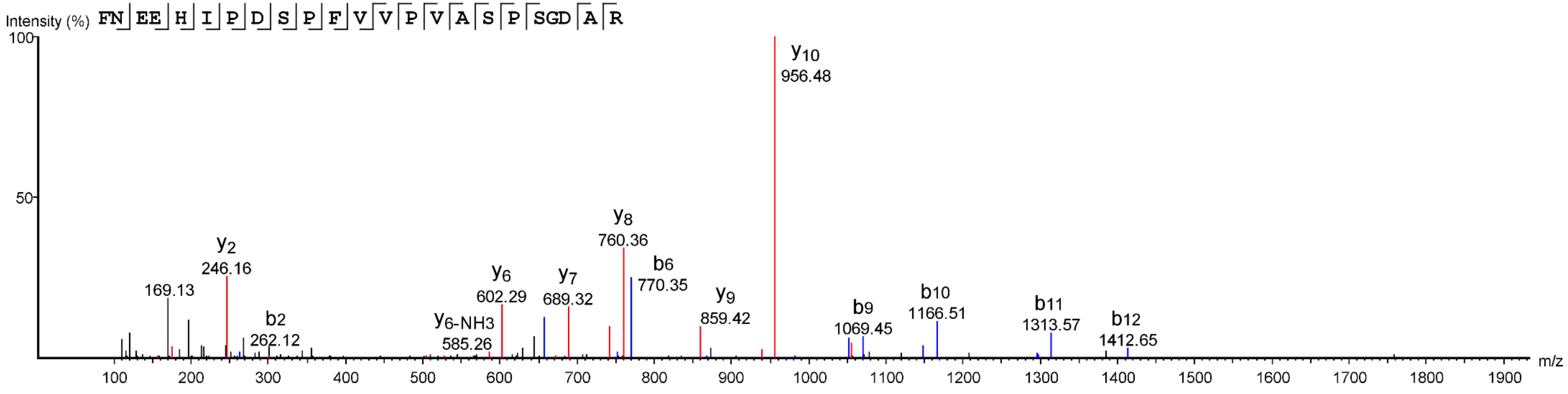

YMT65
Scan #15168

A-188: FLNA$_{(2311-2333)}$: FNEEHIPDSPFVVPVASPSGDAR+

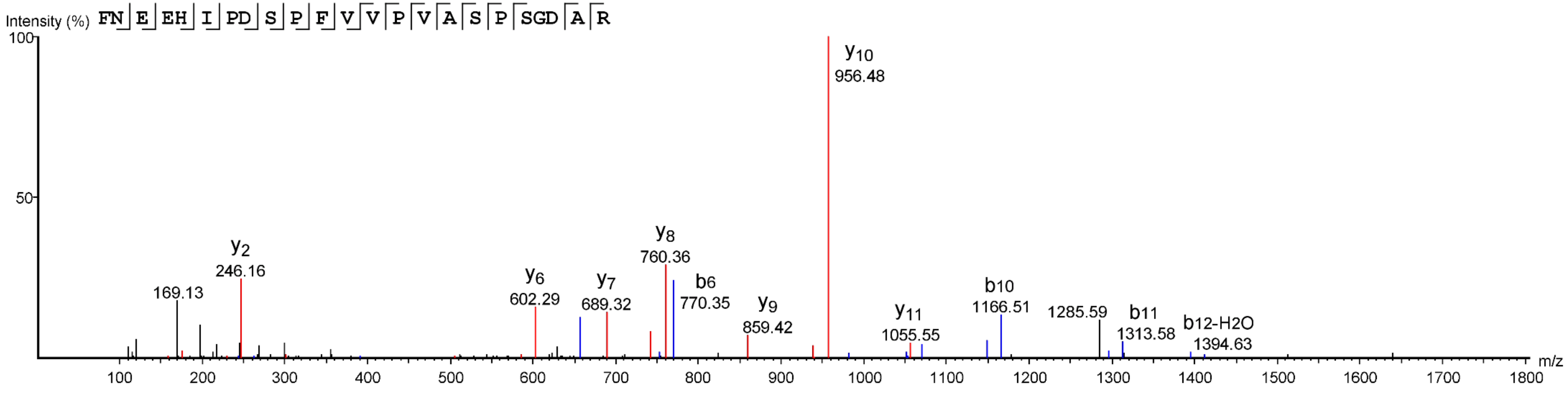

YMT66
Scan #15187

A-189: FOXO3$_{(420-444)}$: GSGLGSPTSSFNSTVFGPSSLNSLR

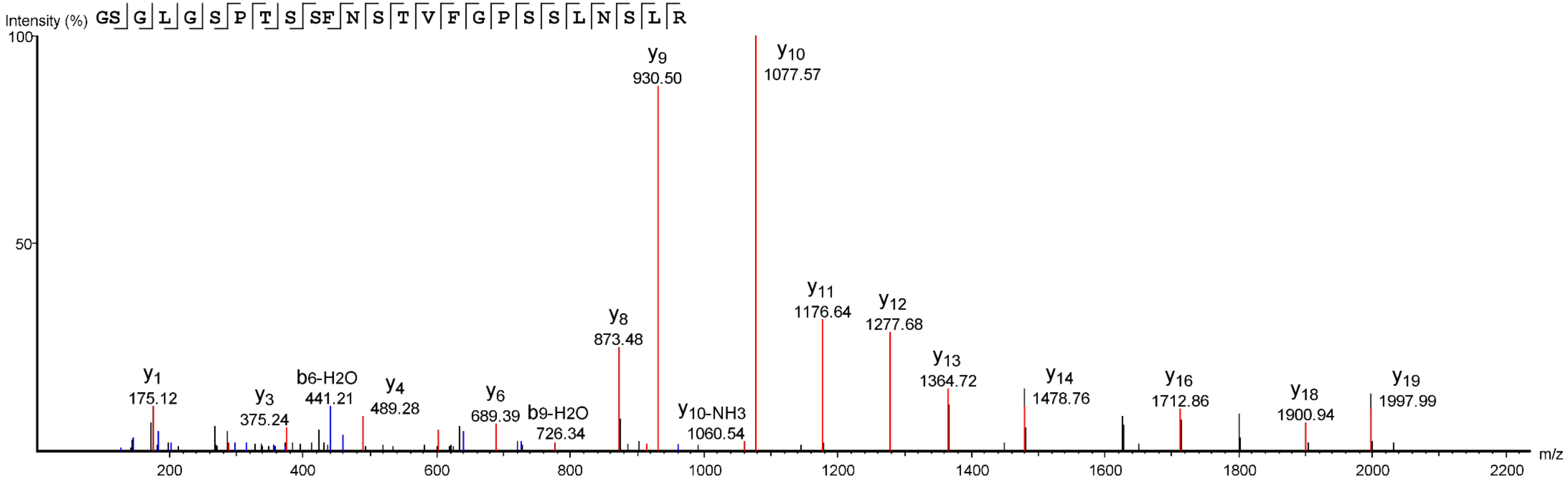

Pep14
Scan
#15621

A-190: FOXO3$_{(420-444)}$: GSGLGSP(+15.99)TSSFNSTVFGPSSLNSLR

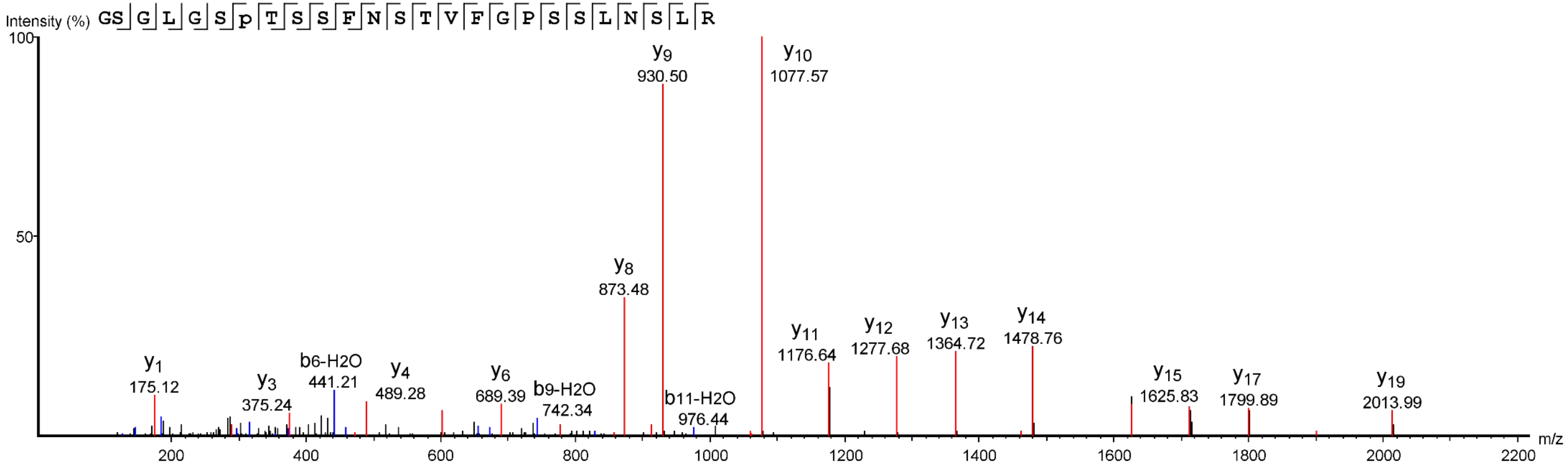

Pep14
Scan
#15012

A-191: FOXO3$_{(420-444)}$: GSGLGSPTSSFNSTVFGP(+15.99)SSLNSLR

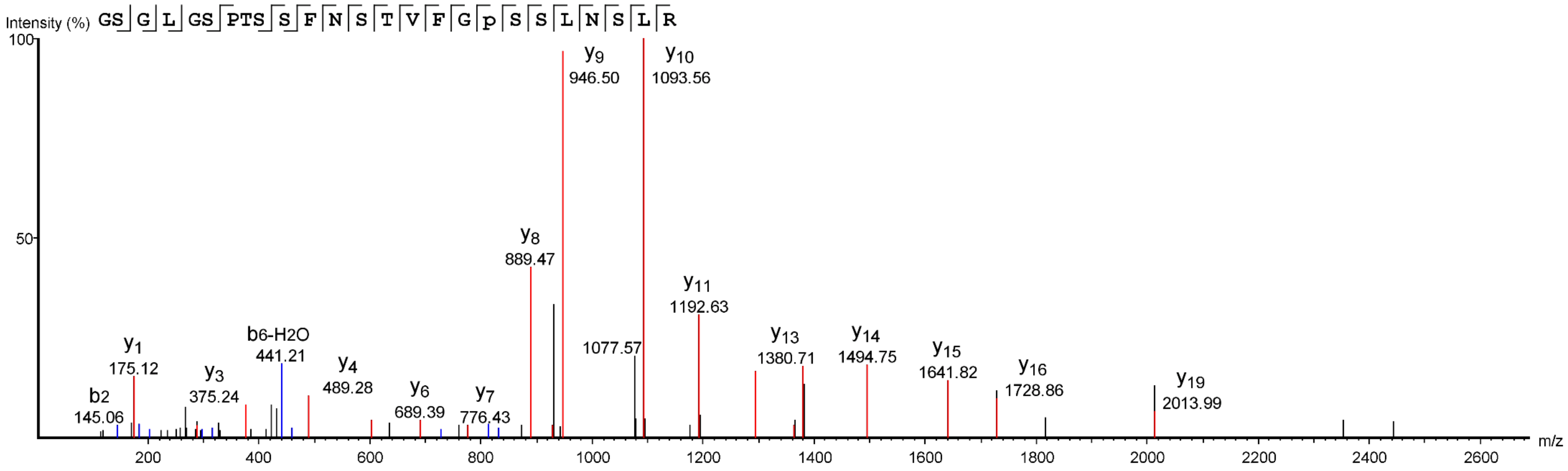

Pep14
Scan
#14963

A-192: FOXO3$_{(420-444)}$: GSGLGSP(+15.99)TSSFNSTVFGP(+15.99)SSLNSLR

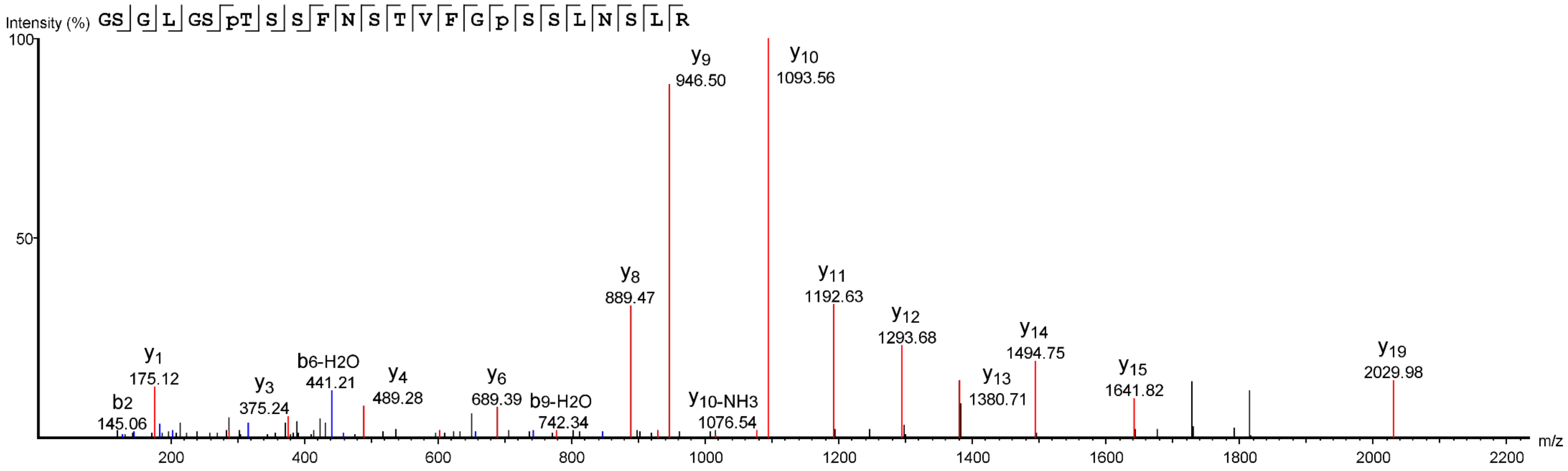

Pep14
Scan
#13988

A-193: FOXO3$_{(420-444)}$: GSGLGSPTSSFNSTVFGPSSLNSLR

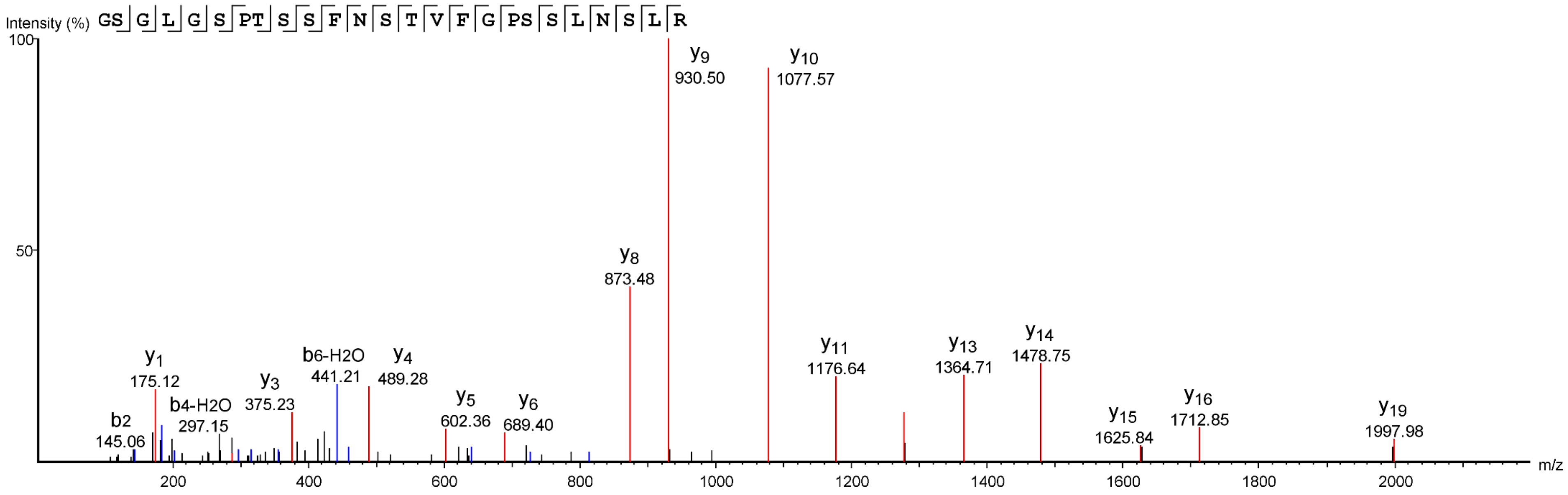

YMT59
Scan
#18279

A-194: FOXO3$_{(420-444)}$: GSGLGSPTSSFNSTVFGPSSLNSLR

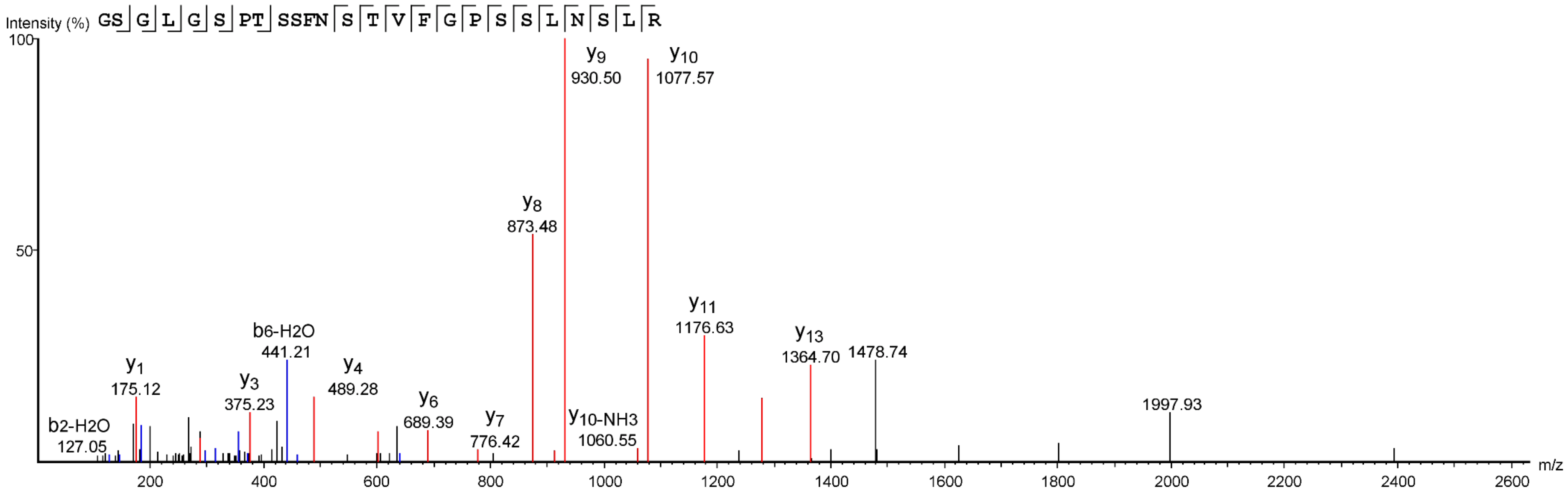

YMT60
Scan
#18433

A-195: IKBKB$_{(172-198)}$: ELDQGSLC(+57.02)TSFVGTLQYLAPELLEQQK

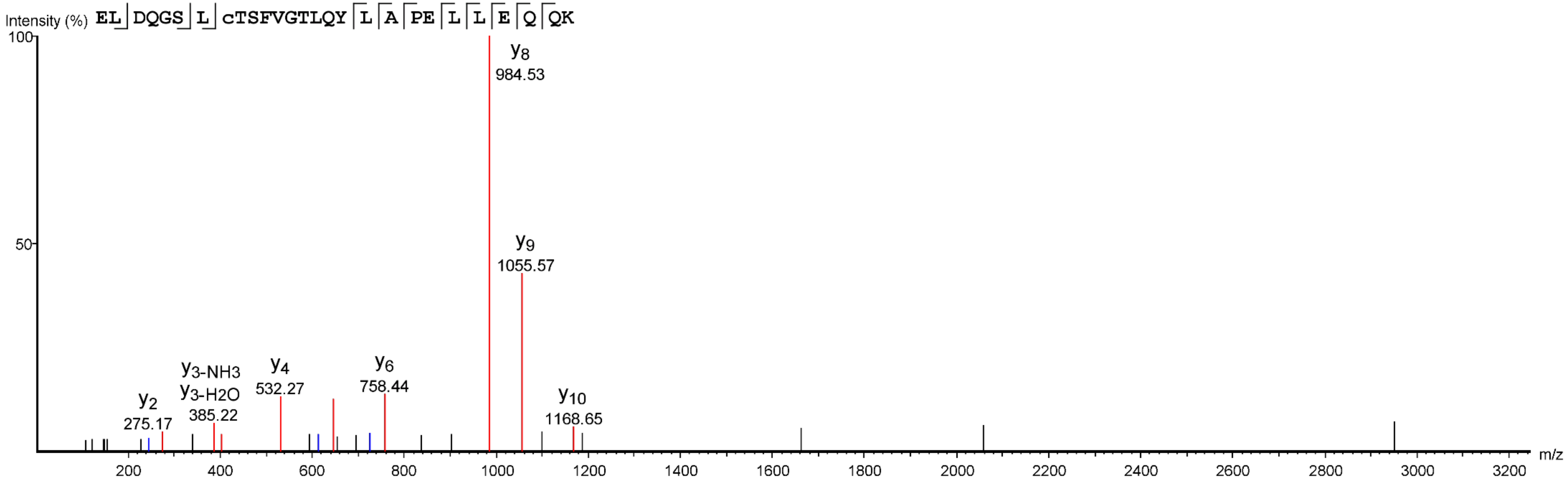

Pep27:
Scan #24625

A-196: IKBKB$_{(172-198)}$: ELDQGSLC(+57.02)TSFVGTLQYLAP(+15.99)ELLEQQK

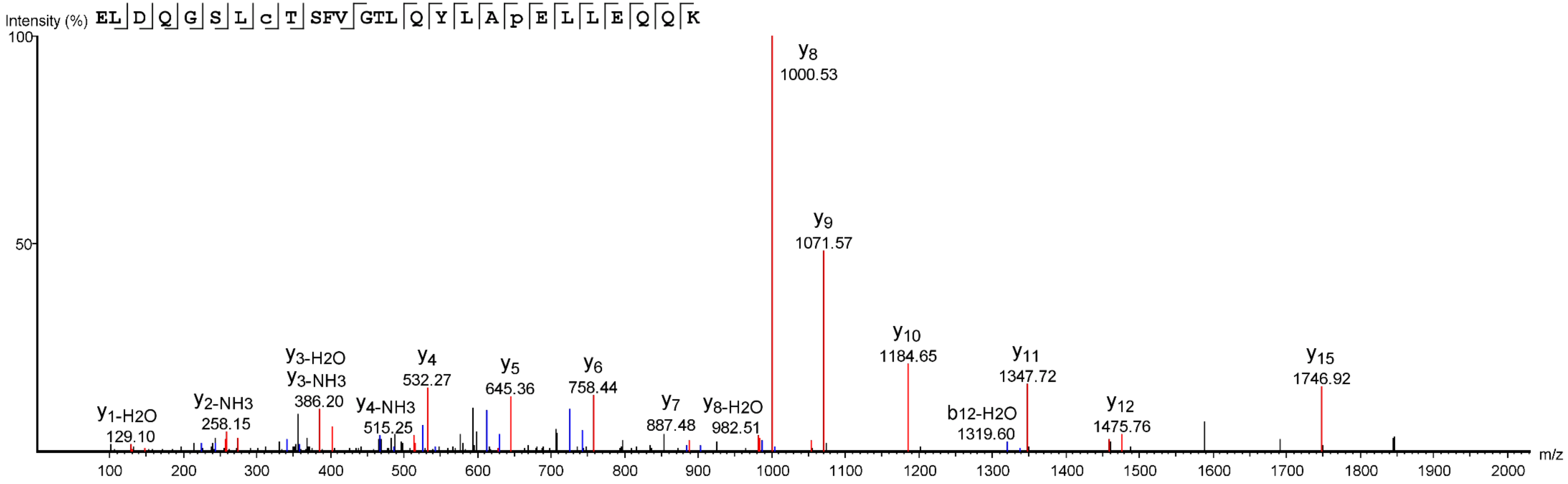

Pep27:

Scan #24433

A-197: IKBKB(172-198): ELDQGSLC(+57.02)TSFVGTLQYLAPELLEQQK

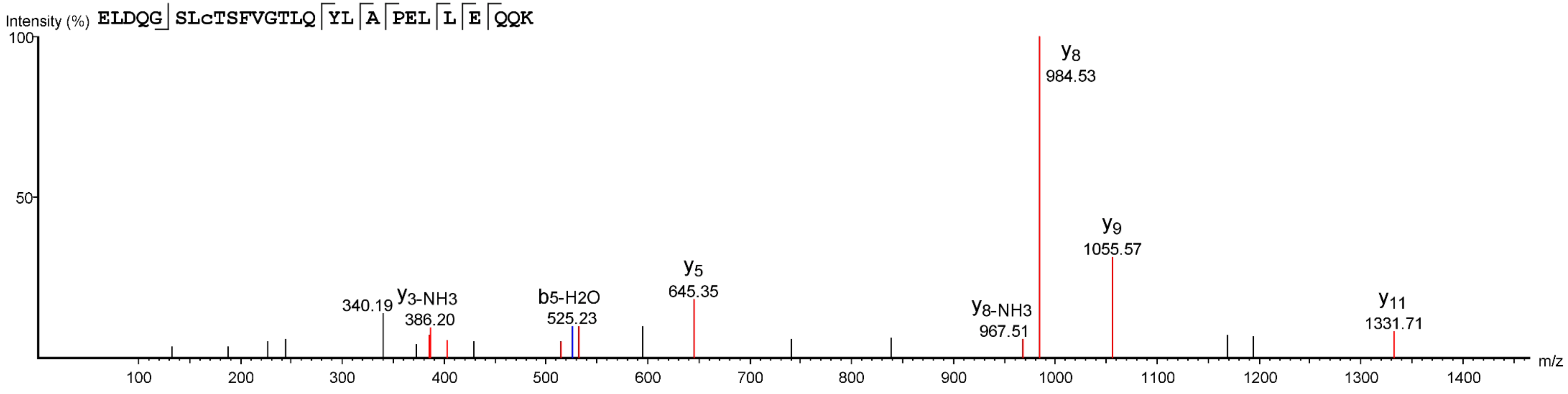

YMT59:
Scan #25756

A-198: IKBKB$_{(172-198)}$: ELDQGSLC(+57.02)TSFVGTLQYLAPELLEQQK

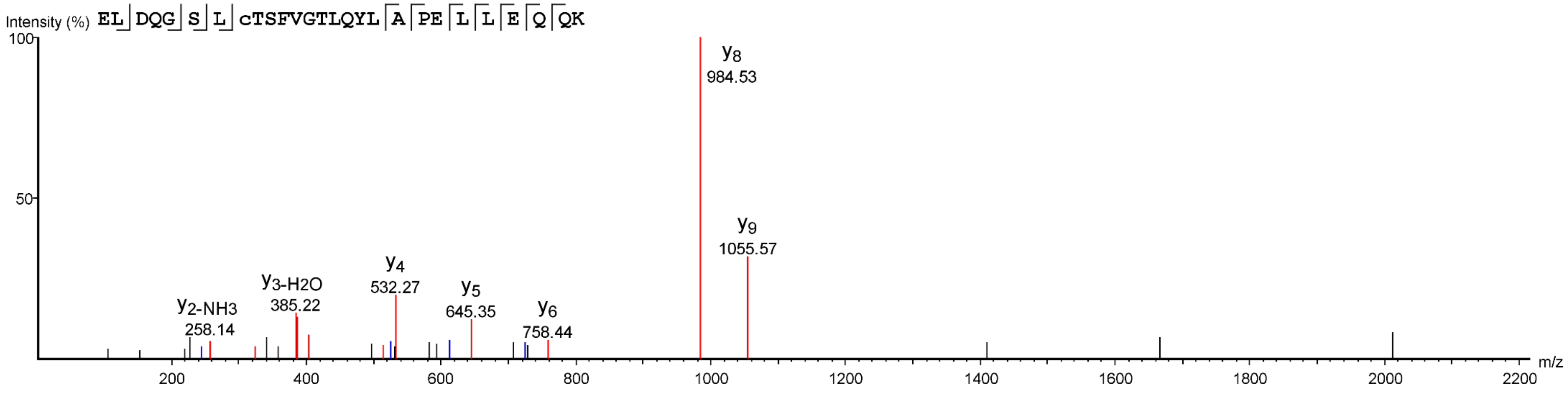

YMT60:
Scan #26206

A-199: MAPK6$_{(20-45)}$: YMDLKPLGC(+57.02)GGNGLVFSAVDNDC(+57.02)DKR

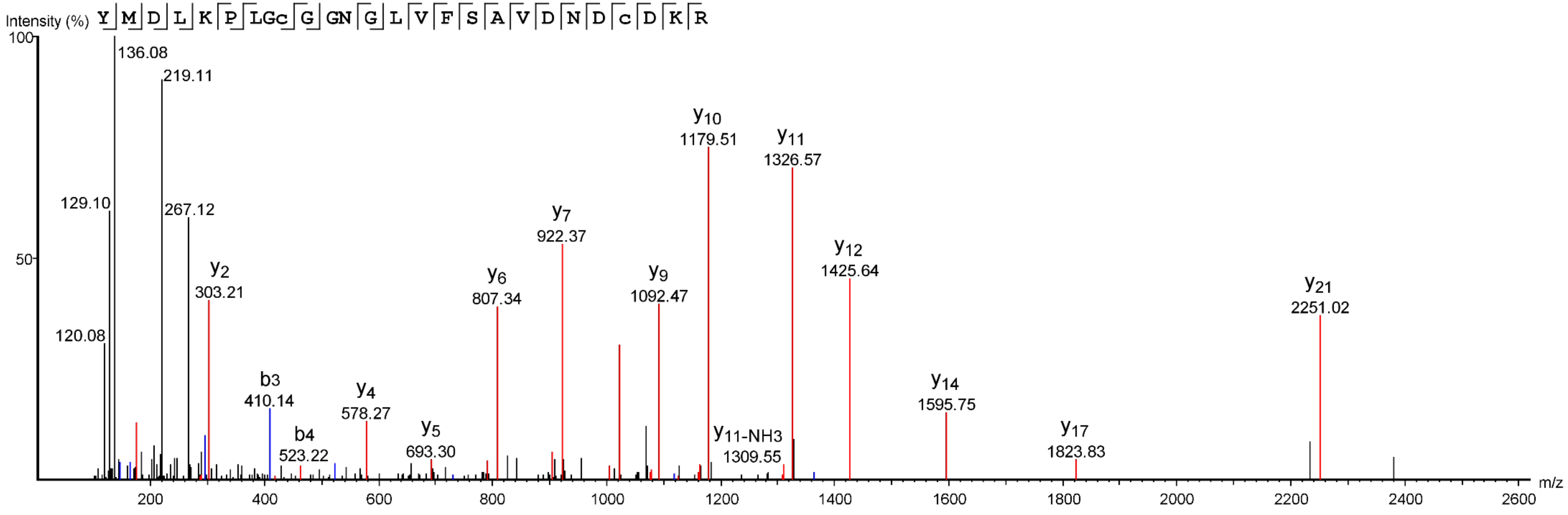

Pep17

Scan

#13503

A-200: MAPK6$_{(20-45)}$: YM(+15.99)DLKPLGC(+57.02)GGNGLVFSAVDNDC(+57.02)DKR

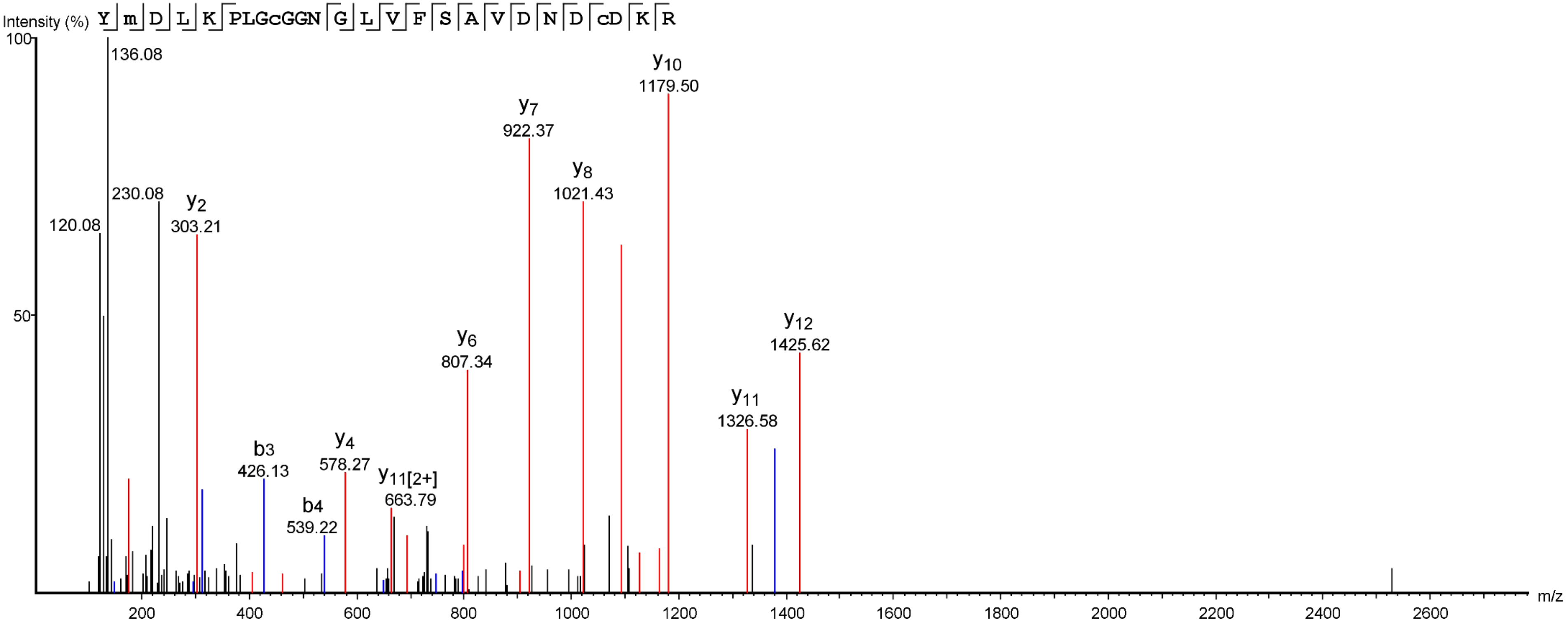

Pep17
Scan
#11999

A-201: MAPK6$_{(20-45)}$: YMDLKP(+15.99)LGC(+57.02)GGNGLVFSAVDNDC(+57.02)DKR

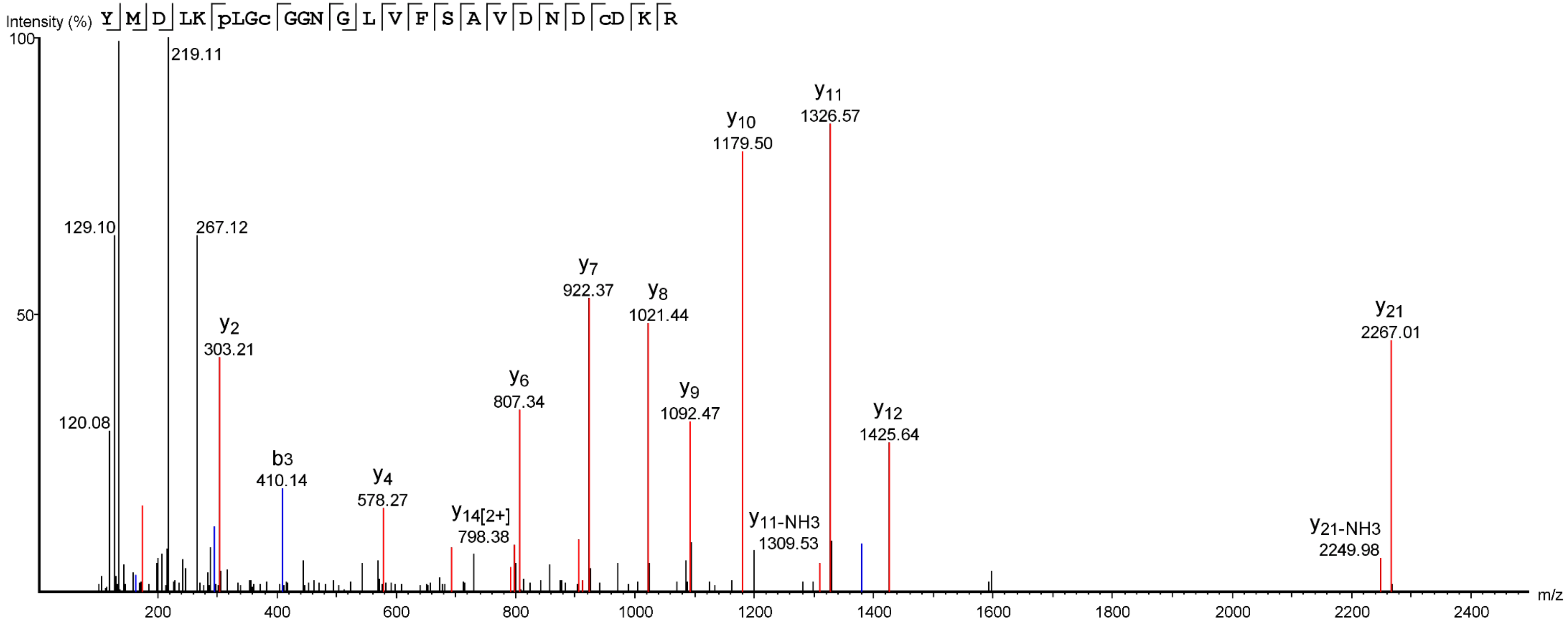

Pep17
Scan
#13087

A-202: MAPK6(20-45): YM(+15.99)DLKP(+15.99)LGC(+57.02)GGNGLVFSAVDNDC(+57.02)DKR

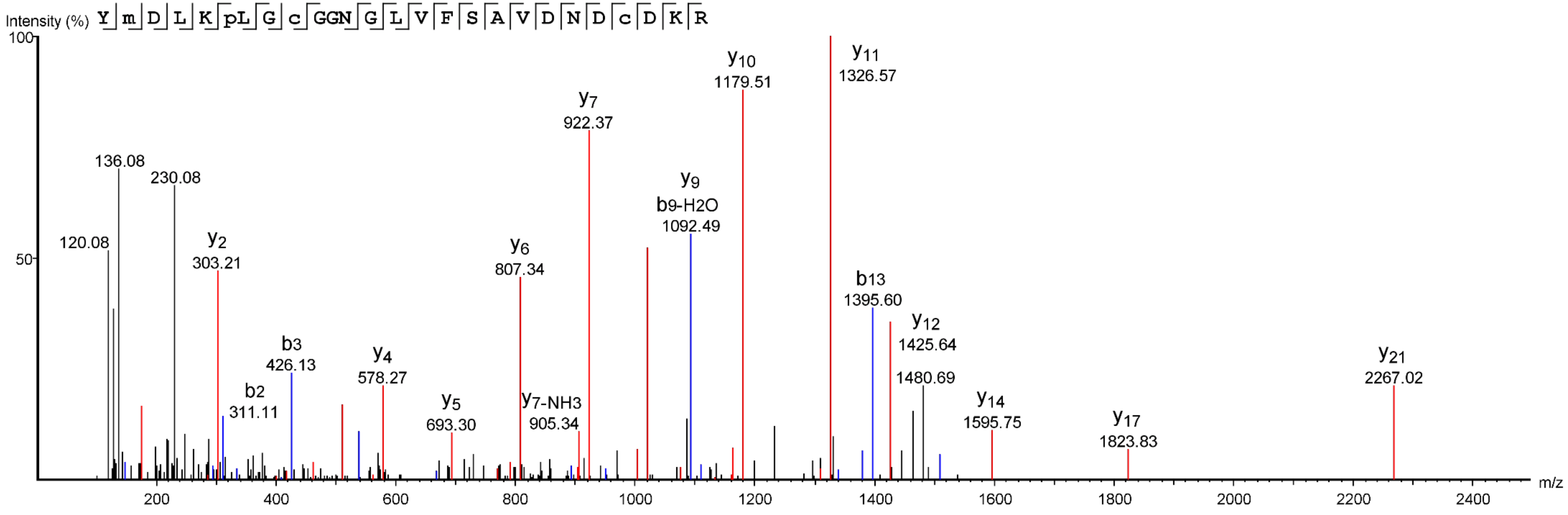

Pep17
Scan
#11327

A-203: MAPK6$_{(20-45)}$: YMDLKPLGC(+57.02)GGNGLVFSAVDNDC(+57.02)DKR

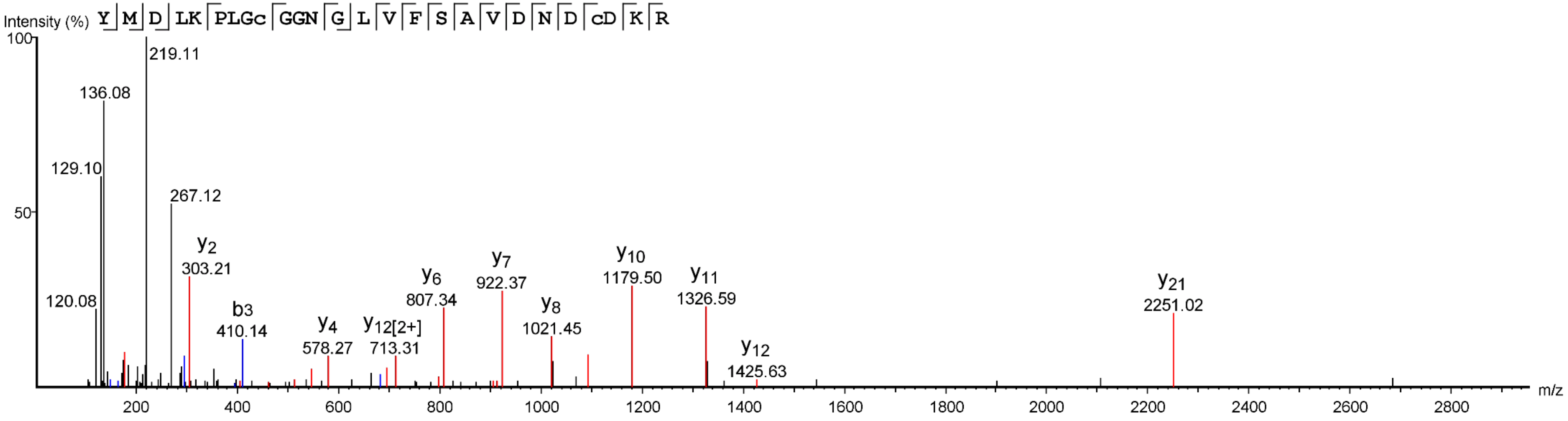

YMT31
Scan
#15317

A-204: MAPK6$_{(20-45)}$: YMDLKPLGC(+57.02)GGNGLVFSAVDNDC(+57.02)DKR

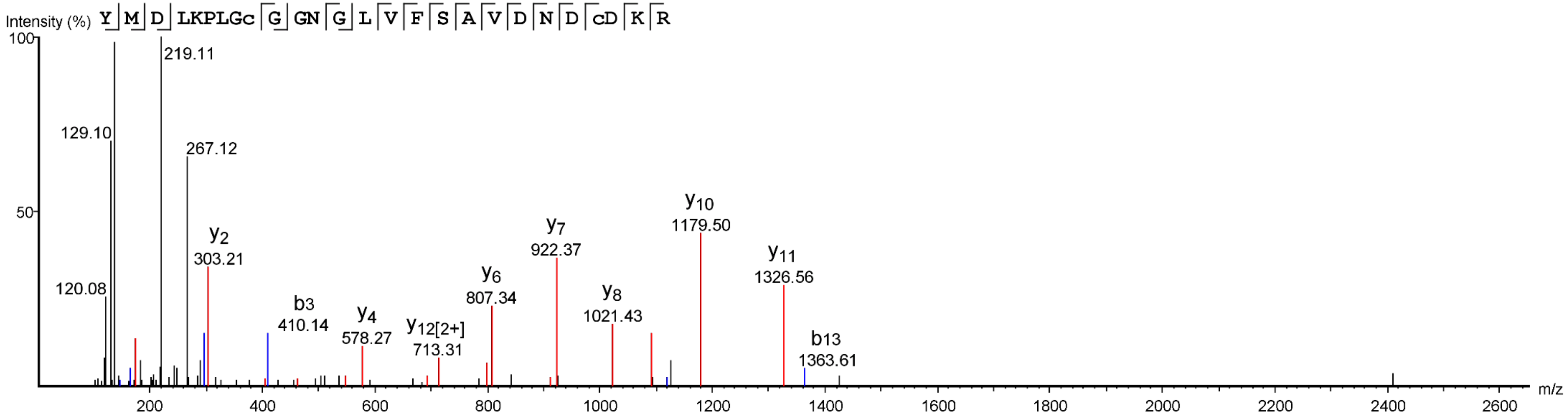

YMT32
Scan
#16233

A-205: MAPK6(20-45): YM(+15.99)DLKPLGC(+57.02)GGNGLVFSAVDNDC(+57.02)DKR

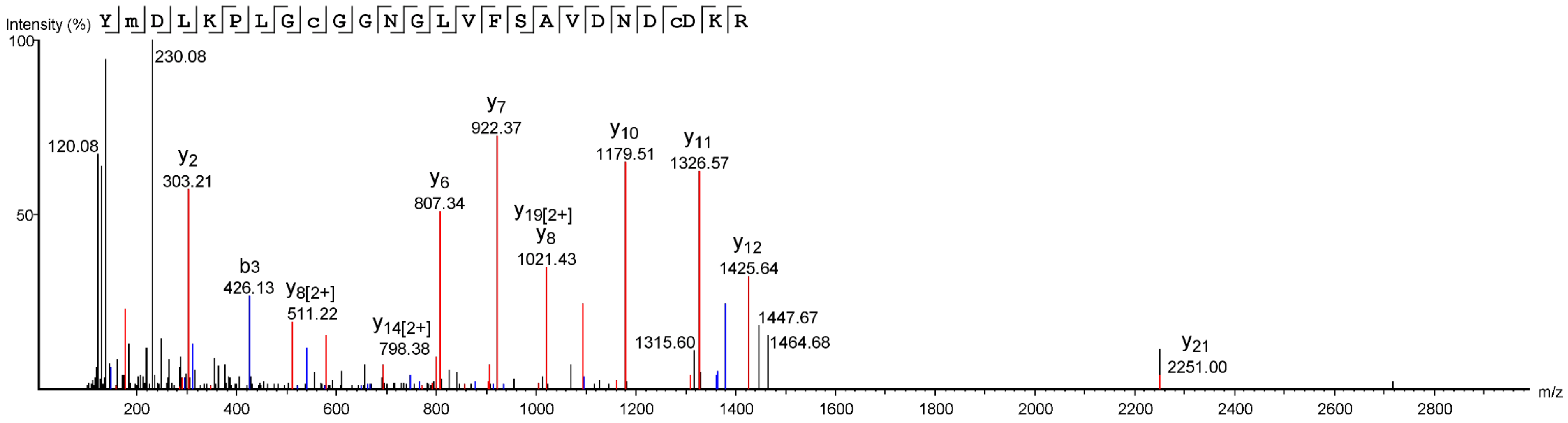

YMT31
Scan
#13401

A-206: MAPK6$_{(20-45)}$: YM(+15.99)DLKPLGC(+57.02)GGNGLVFSAVDNDC(+57.02)DKR

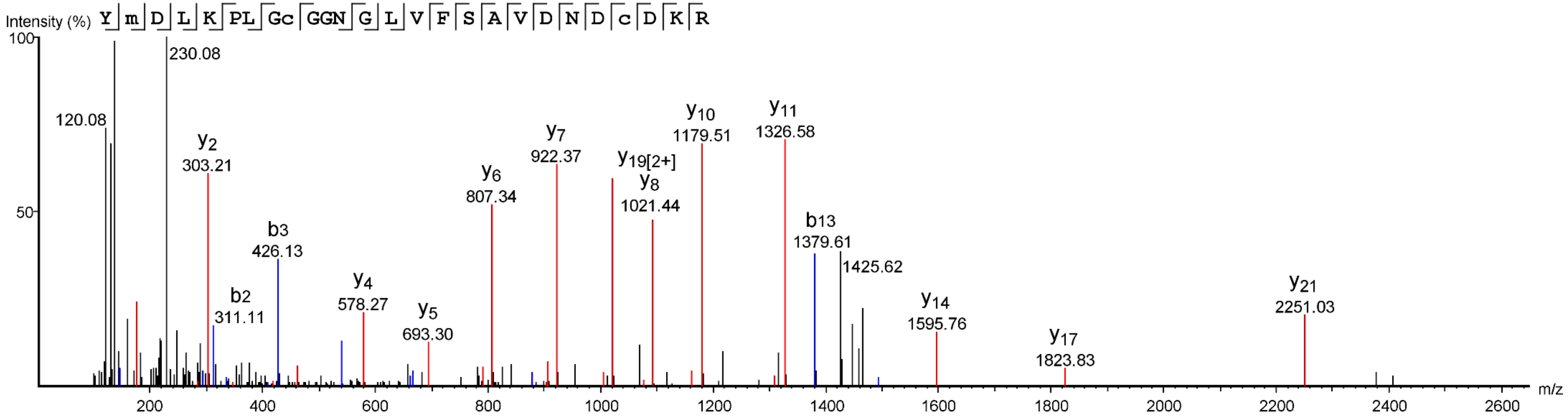

YMT32
Scan
#14193

A-207: NDRG3$_{(287-301)}$: MADC(+57.02)GGLPQVVQPGK

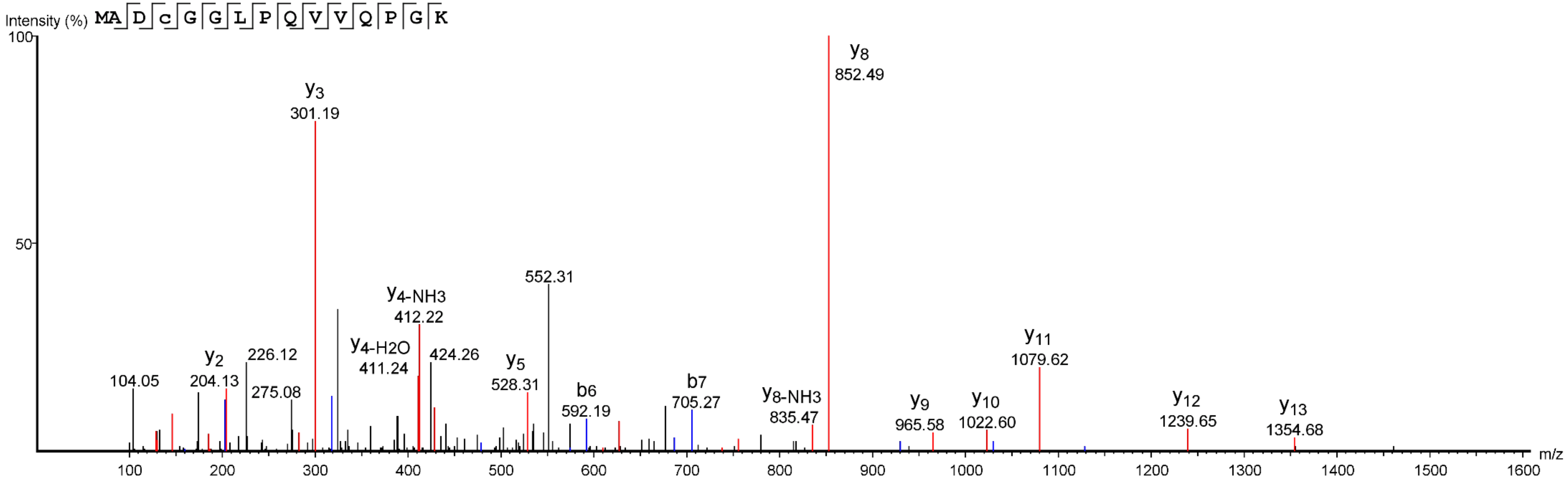

Pep11
Scan #11045

A-208: NDRG3$_{(287-301)}$: M(+15.99)ADC(+57.02)GGLPQVVQPGK

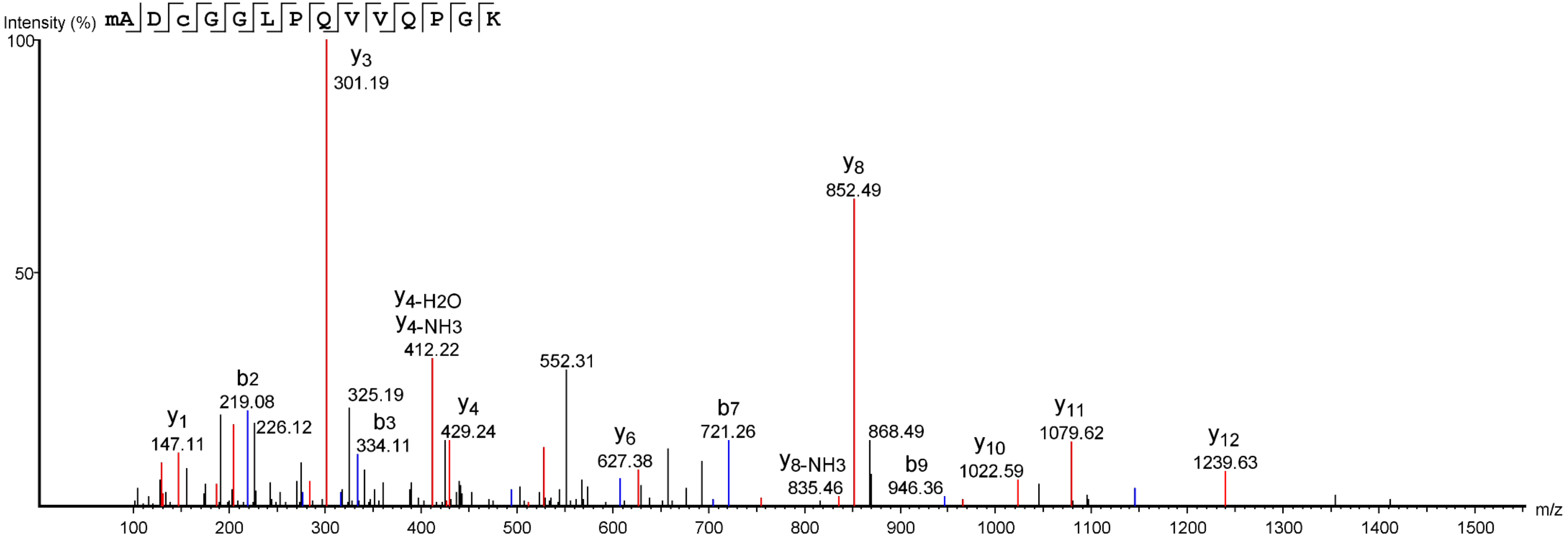

Pep11
Scan #10037

A-209: NDRG3$_{(287\text{-}301)}$: MADC(+57.02)GGLP(+15.99)QVVQPGK

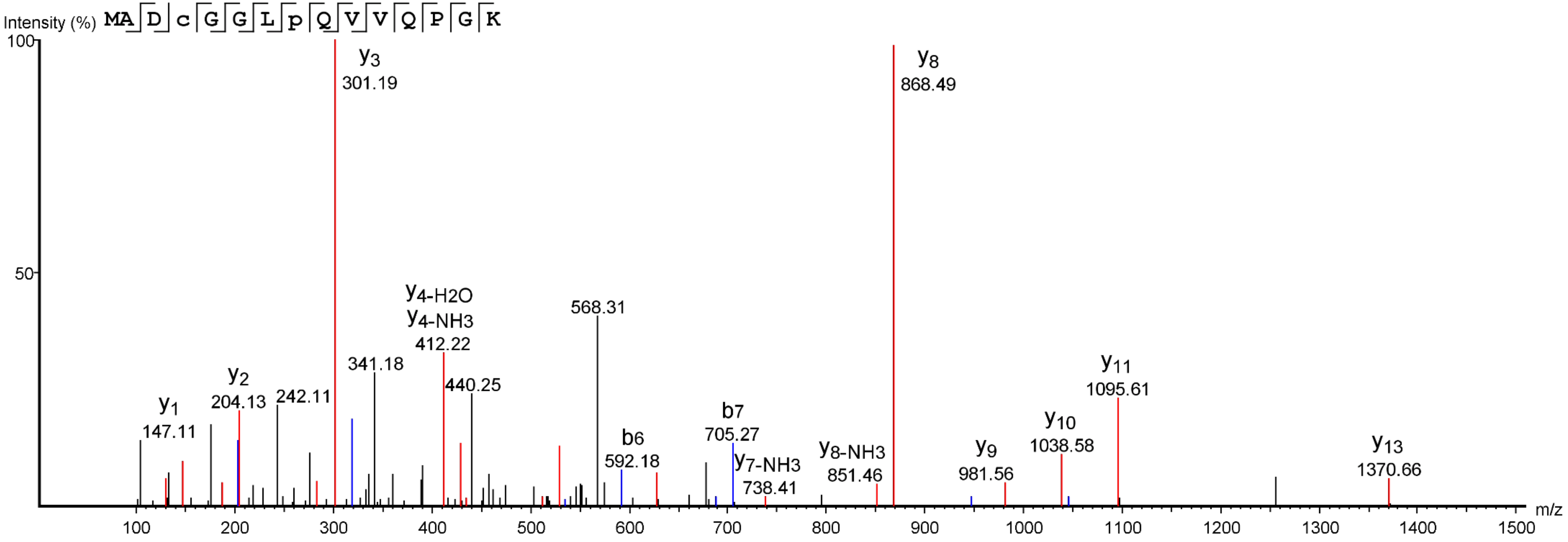

Pep11

Scan #9733

A-210: NDRG3$_{(287-301)}$: M(+15.99)ADC(+57.02)GGLP(+15.99)QVVQPGK

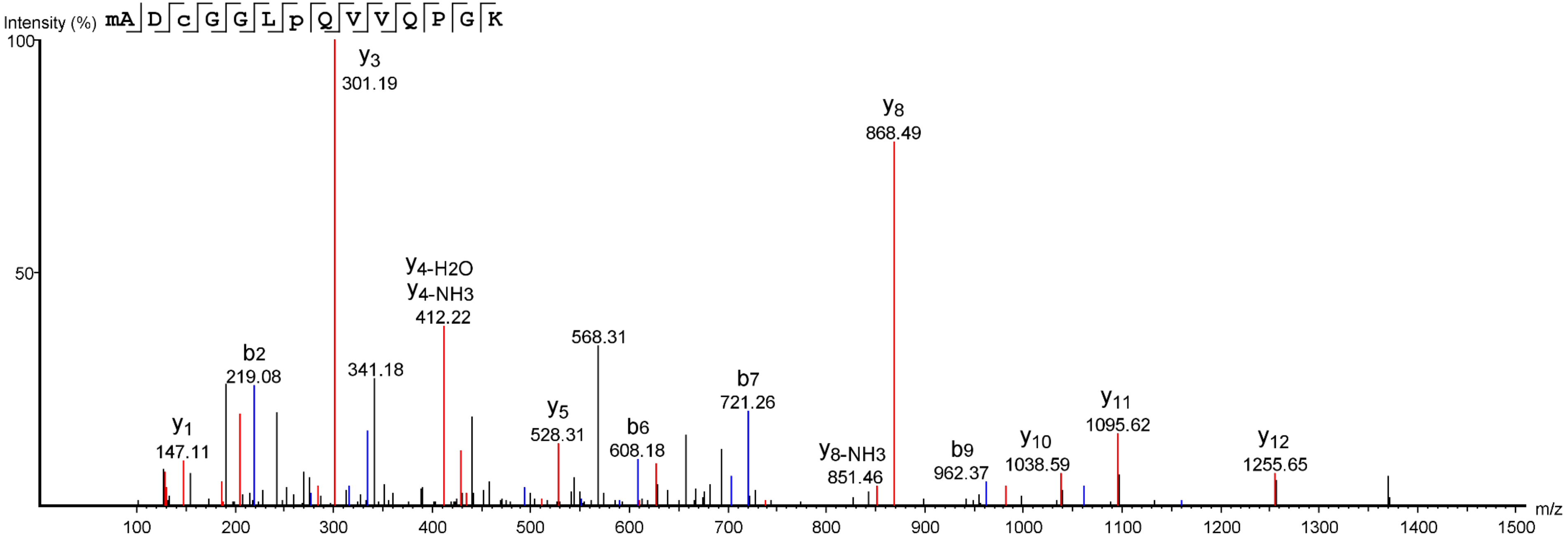

Pep11
Scan #8646

A-211: NDRG3$_{(287-301)}$: MADC(+57.02)GGLPQVVQPGK

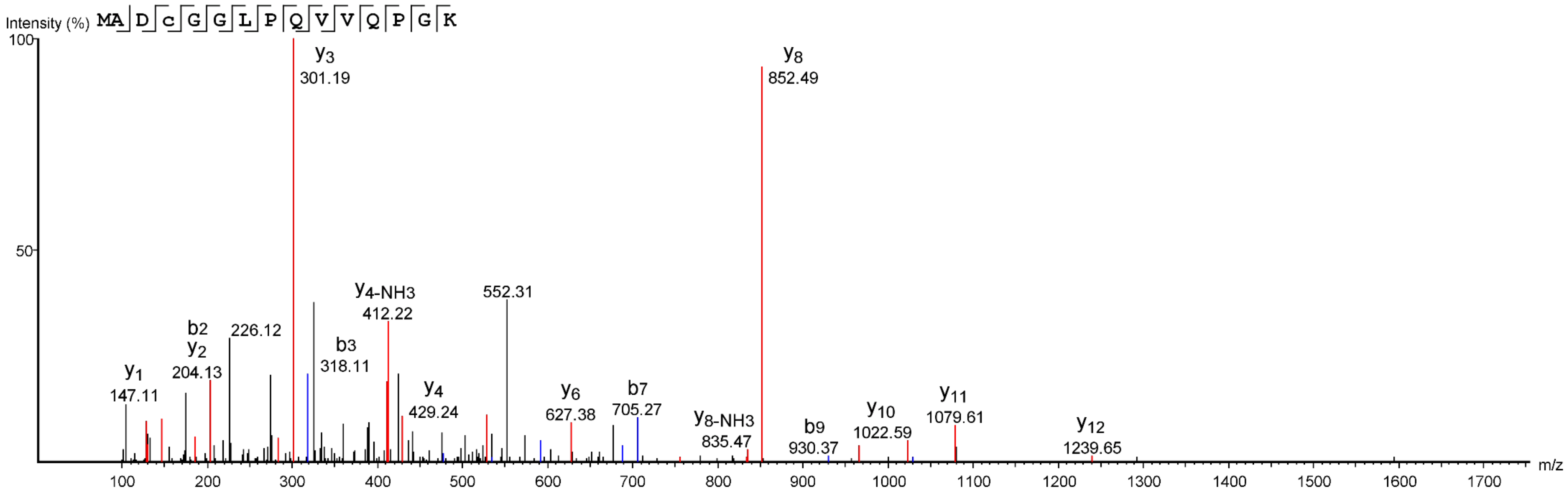

YMT23
Scan #11180

A-212: NDRG3$_{(287-301)}$: MADC(+57.02)GGLPQVVQPGK

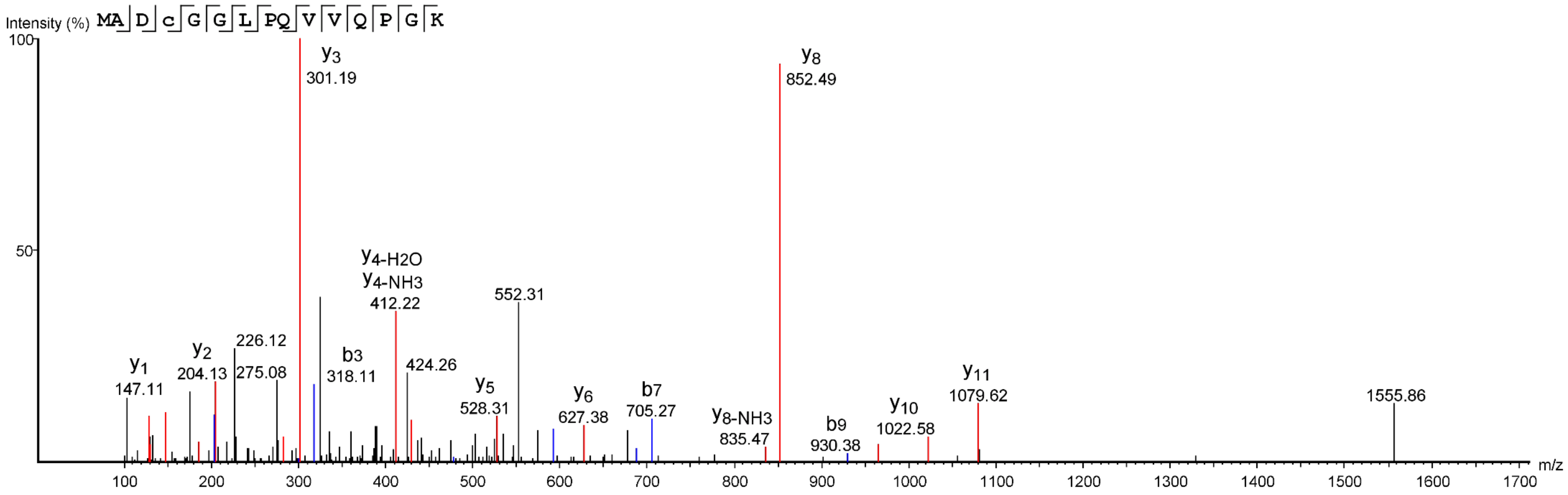

YMT24
Scan #11301

A-213: NDRG3$_{(287-301)}$: M(+15.99)ADC(+57.02)GGLPQVVQPGK

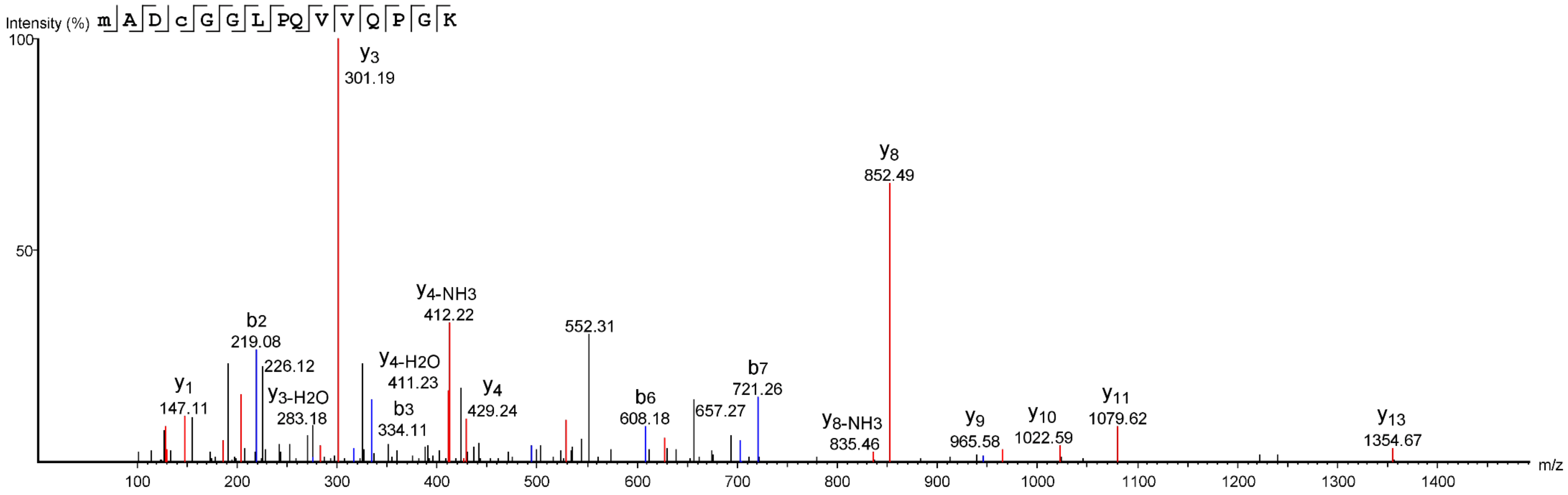

YMT23

Scan #10124

A-214: NDRG3$_{(287-301)}$: M(+15.99)ADC(+57.02)GGLPQVVQPGK

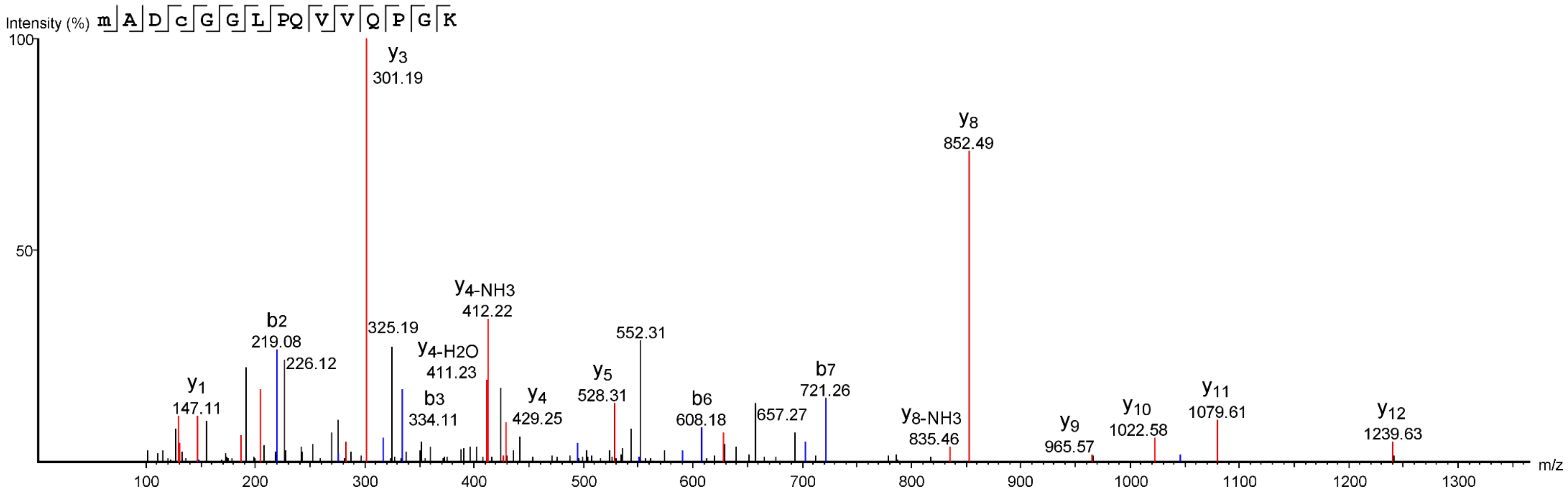

YMT24
Scan #10247

A-215: PDE4D$_{(370-383)}$: LMHSSSLTNSSIPR+

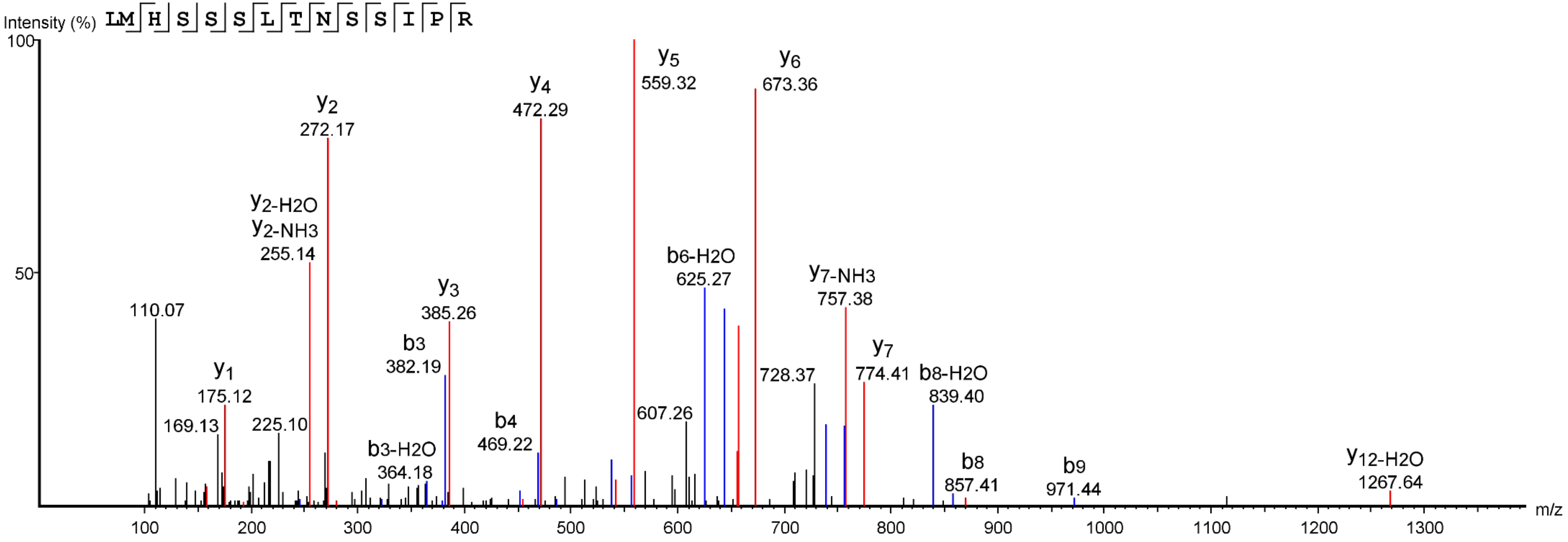

Pep11
Scan #7494

A-216: PDE4D$_{(370-383)}$: LM(+15.99)HSSSLTNSSIPR+

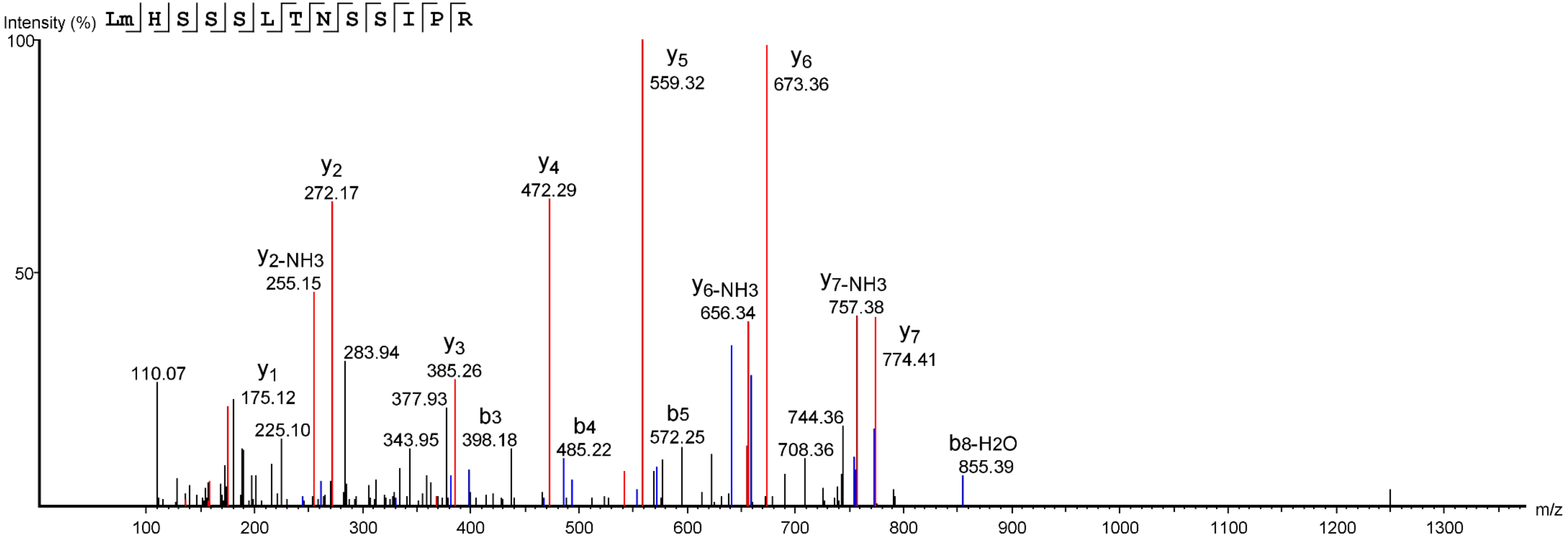

Pep11
Scan #6294

A-217: PDE4D$_{(370-383)}$: LMHSSSLTNSSIP(+15.99)R+

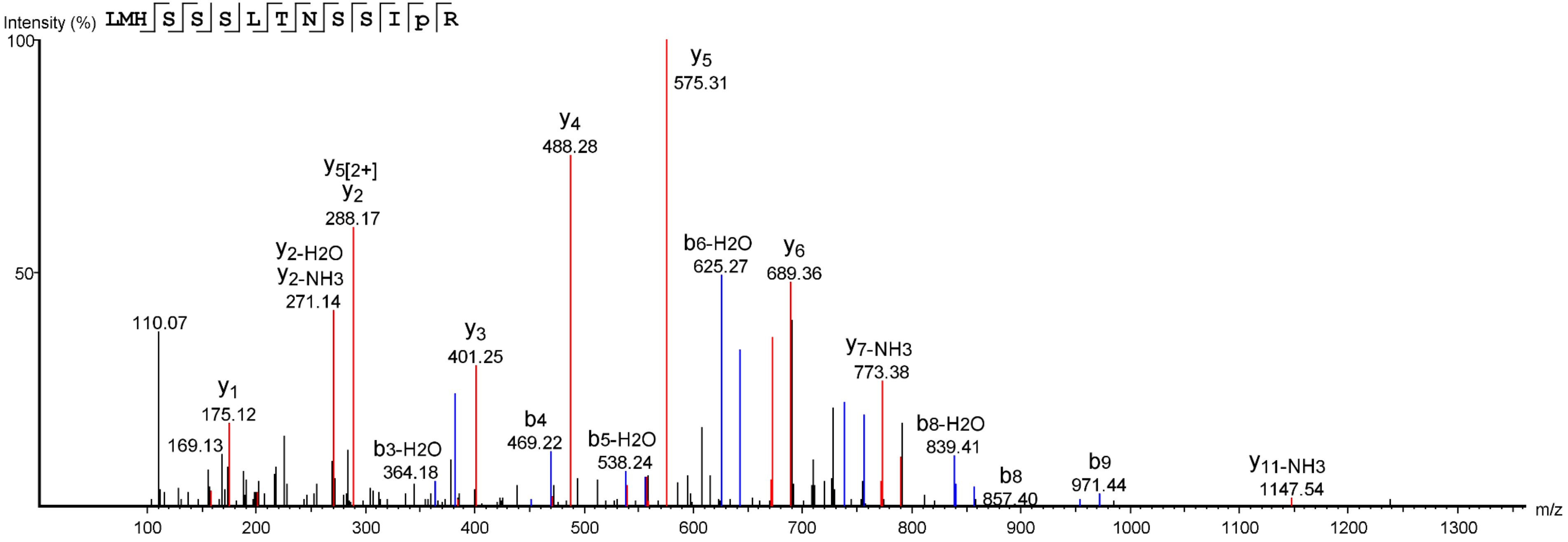

Pep11
Scan #7113

A-218: PDE4D$_{(370-383)}$: LM(+15.99)HSSSLTNSSIP(+15.99)R+

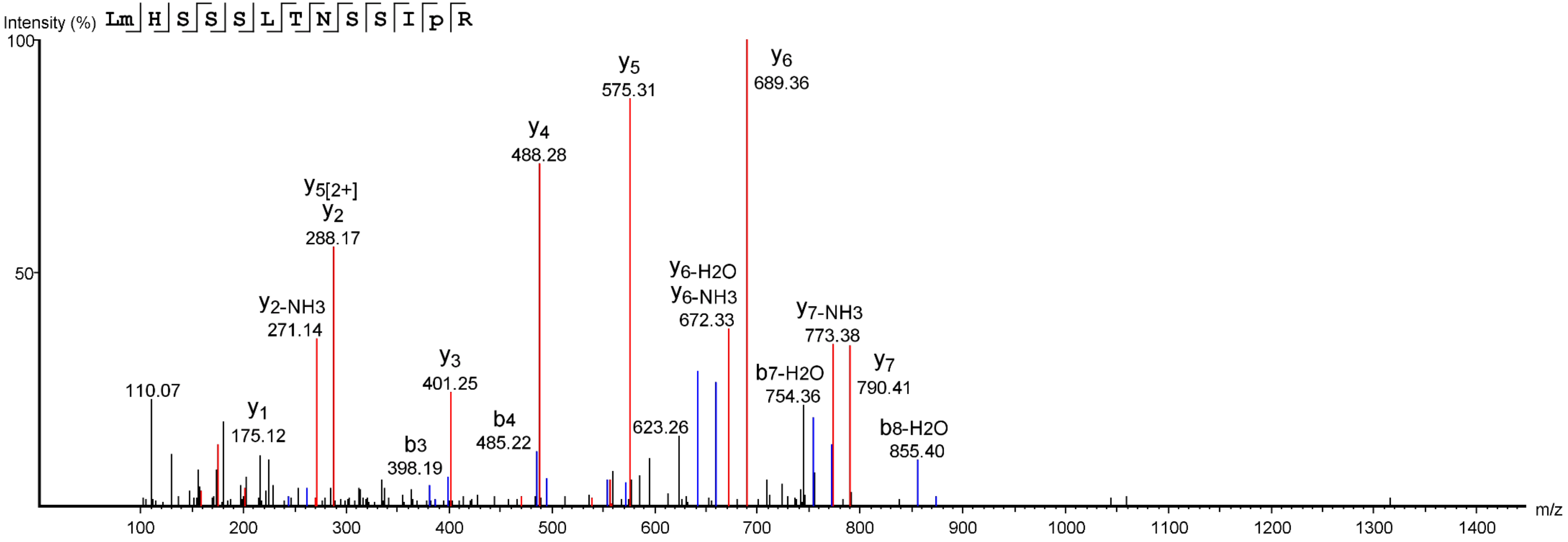

Pep11
Scan #5700

A-219: PDE4D$_{(370-383)}$: LMHSSSLTNSSIPR+

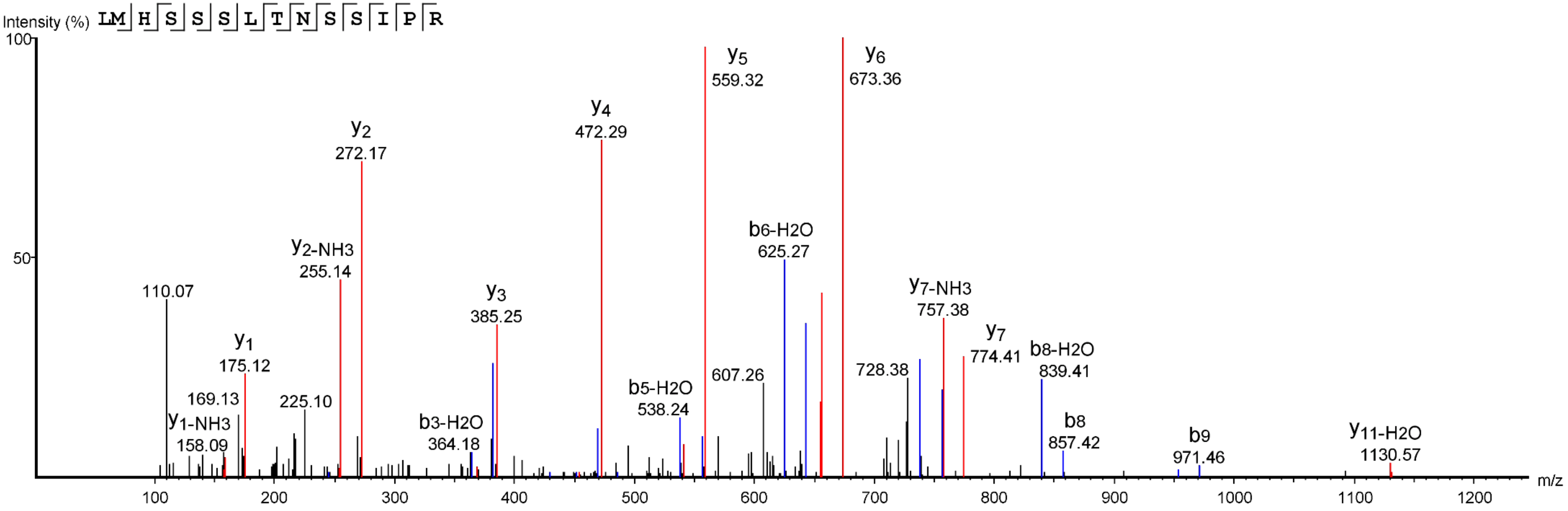

YMT69
Scan #7231

A-220: PDE4D$_{(370-383)}$: LMHSSSLTNSSIPR+

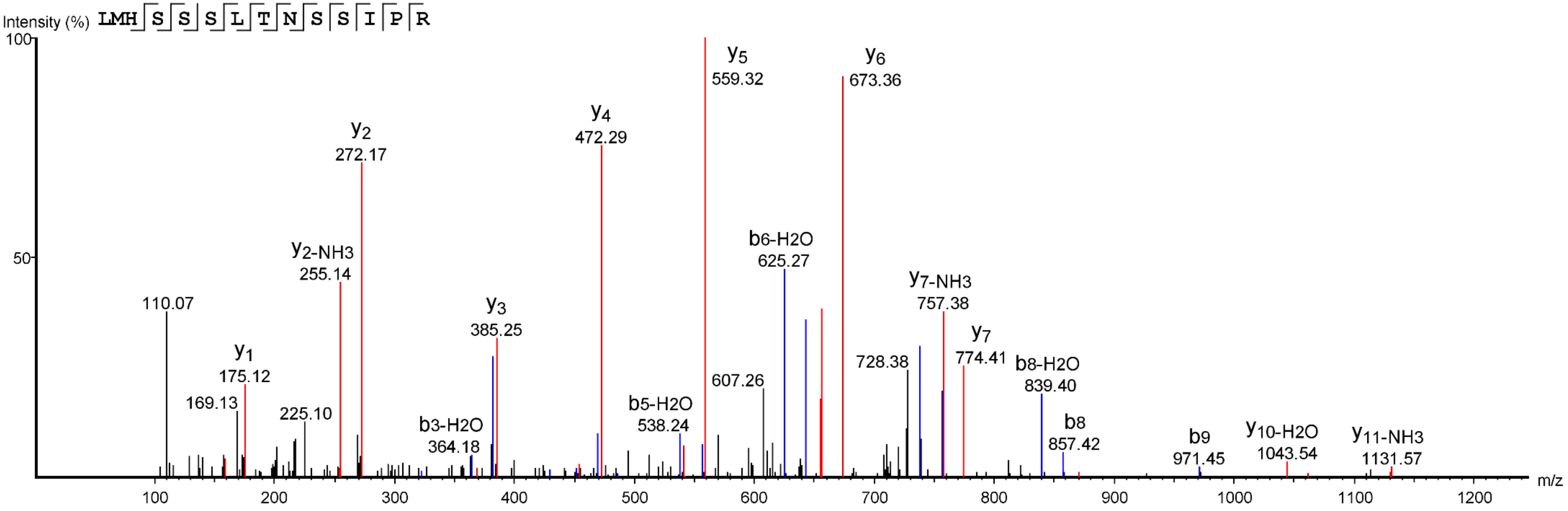

YMT70

Scan #7209

A-221: PDE4D$_{(370-383)}$: LMHSSSLTNSSIPR+

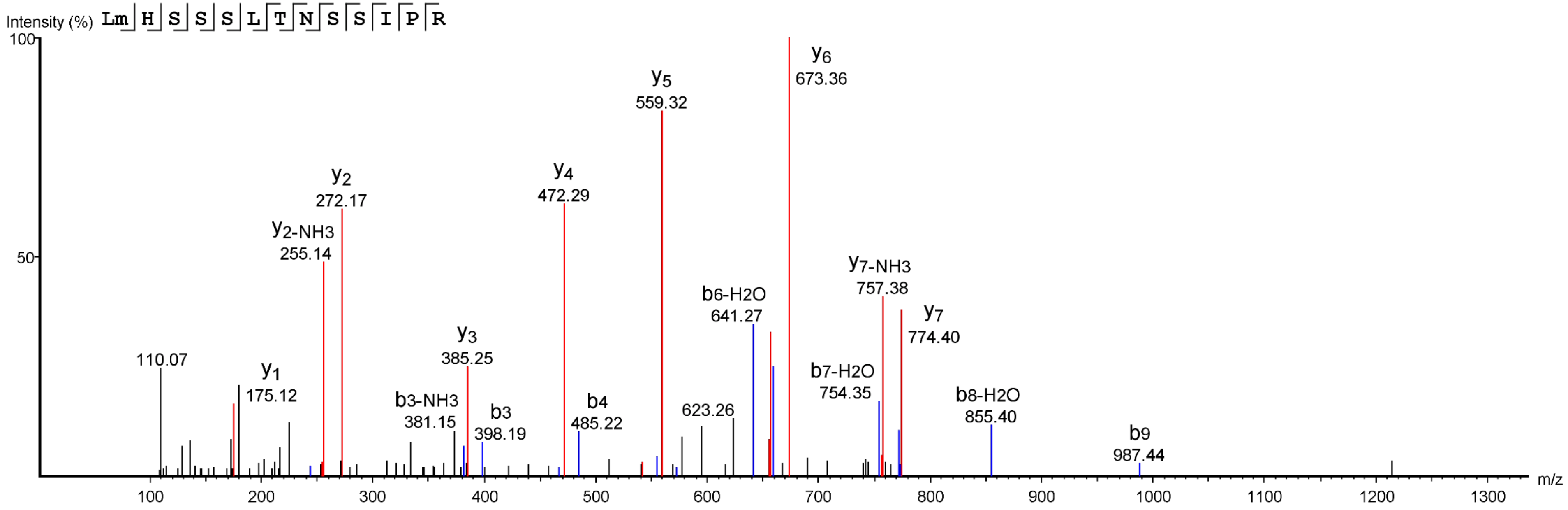

YMT69
Scan #6204

A-222: PDE4D(370-383): LMHSSSLTNSSIPR+

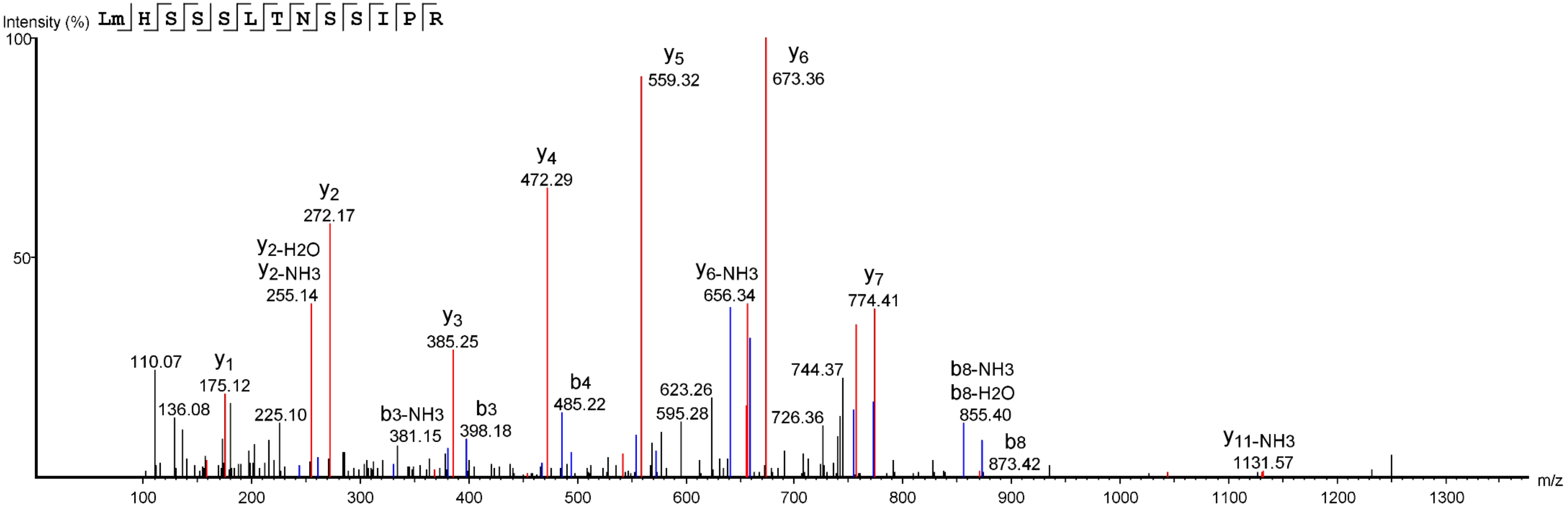

YMT70
Scan #6073

A-223: PDE4D$_{(411-431)}$: IAELSGNRPLTVIMHTIFQER

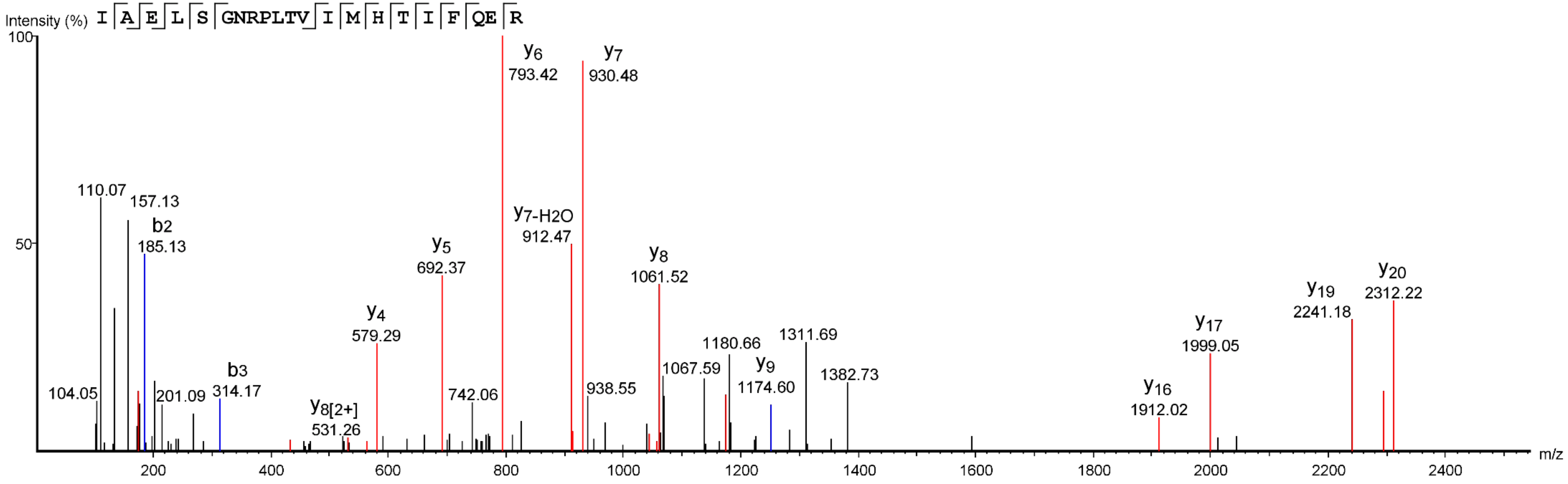

Pep17
Scan
#17727

A-224: PDE4D$_{(411-431)}$: IAELSGNRP(+15.99)LTVIMHTIFQER

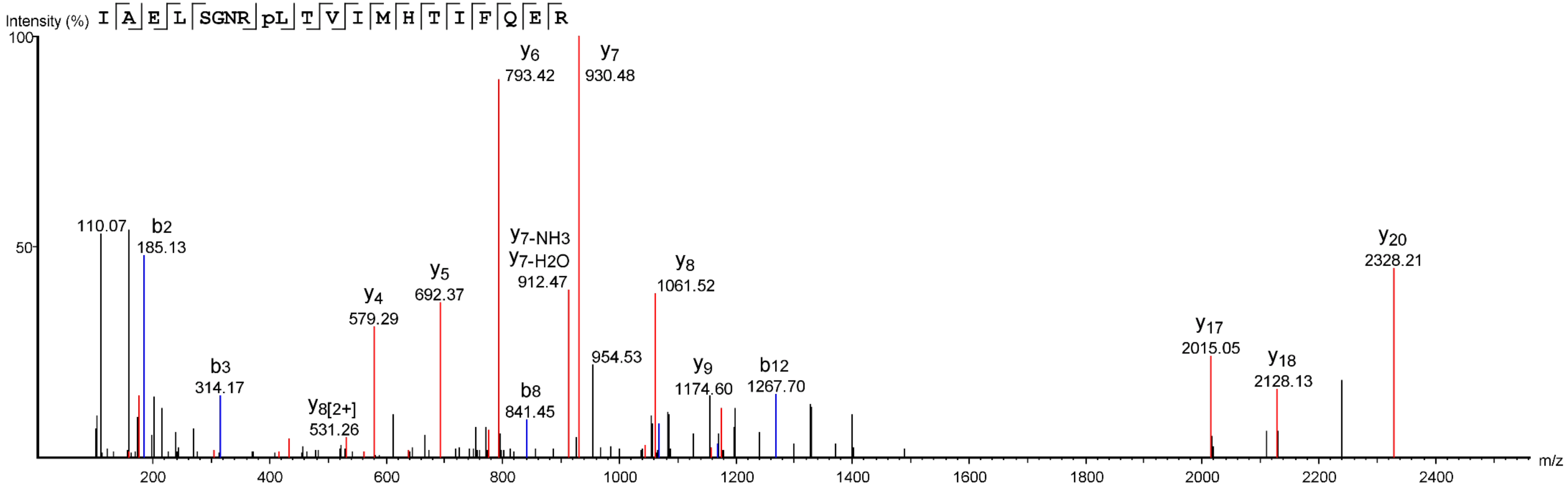

Pep17
Scan
#16768

A-225: PDE4D$_{(411-431)}$: IAELSGNRPLTVIM(+15.99)HTIFQER

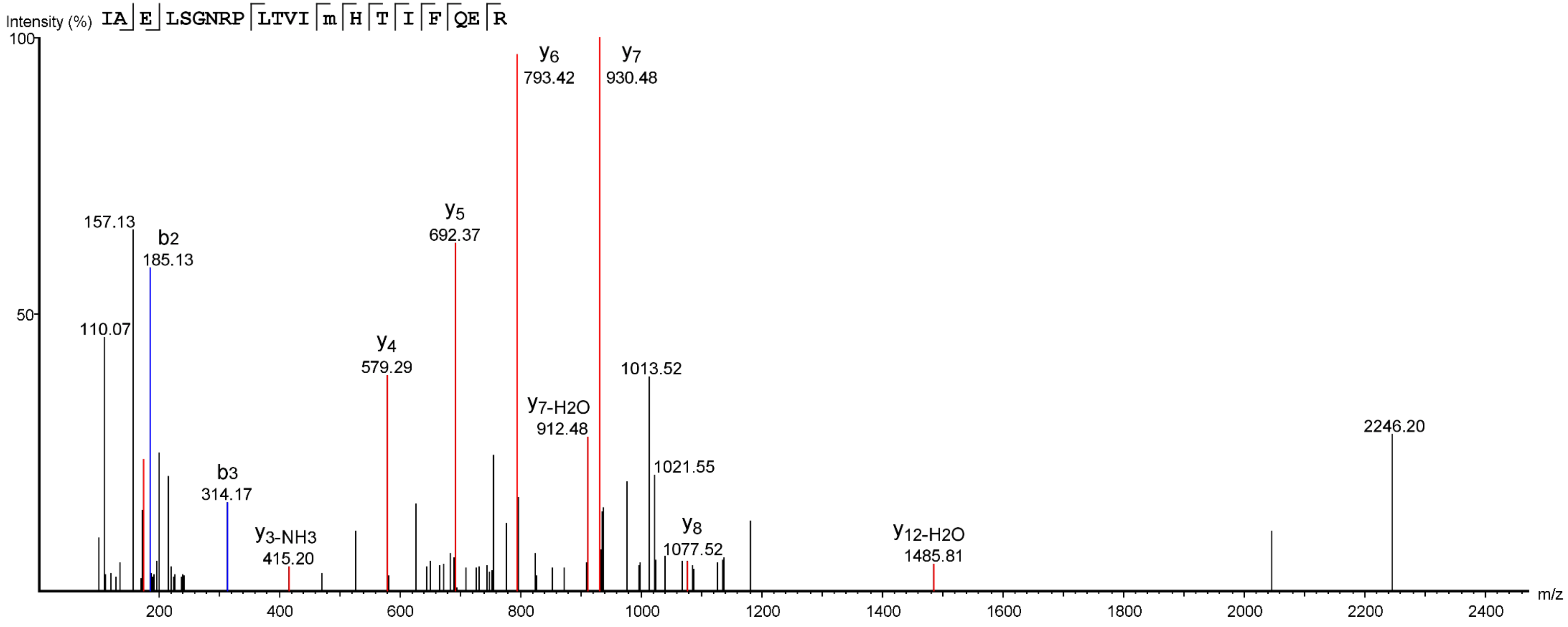

Pep17
Scan
#14357

A-226: PDE4D$_{(411-431)}$: IAELSGNRP(+15.99)LTVIM(+15.99)HTIFQER

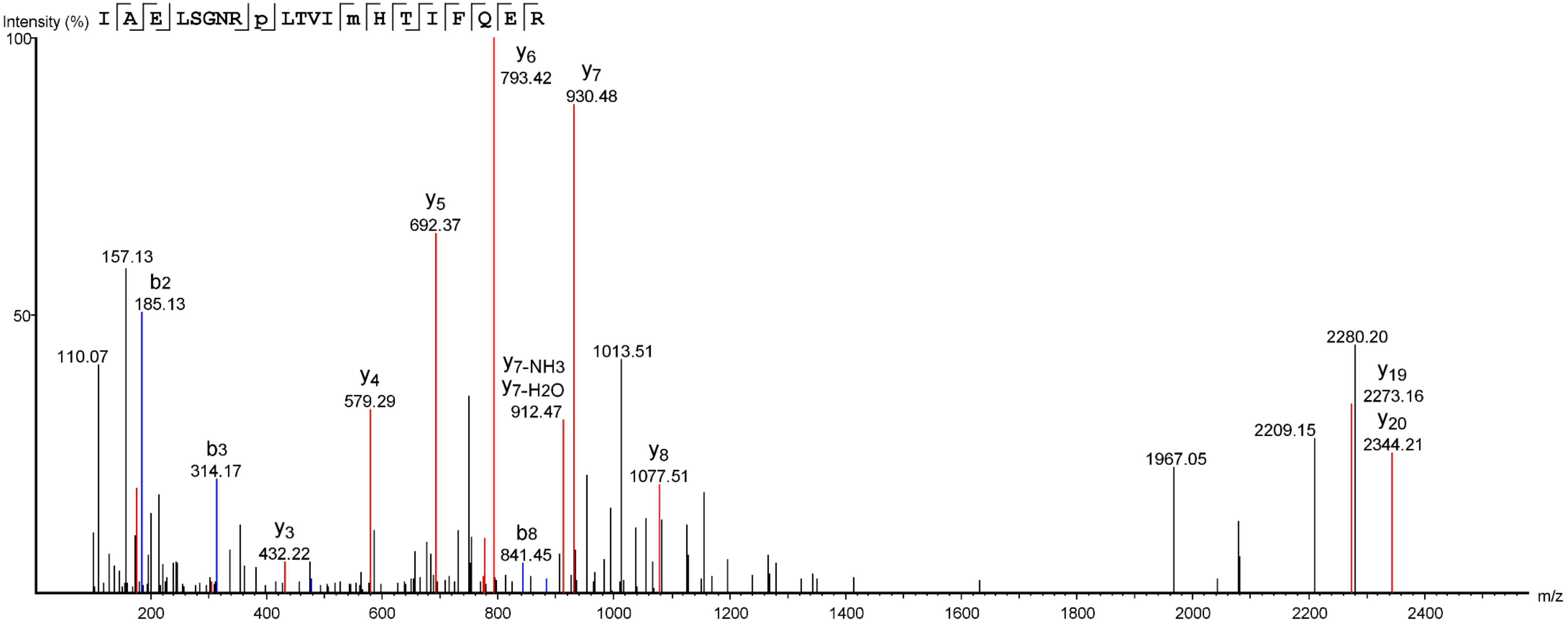

Pep17
Scan
#12980

A-227: PDE4D(411-431): IAELSGNRPLTVIMHTIFQER

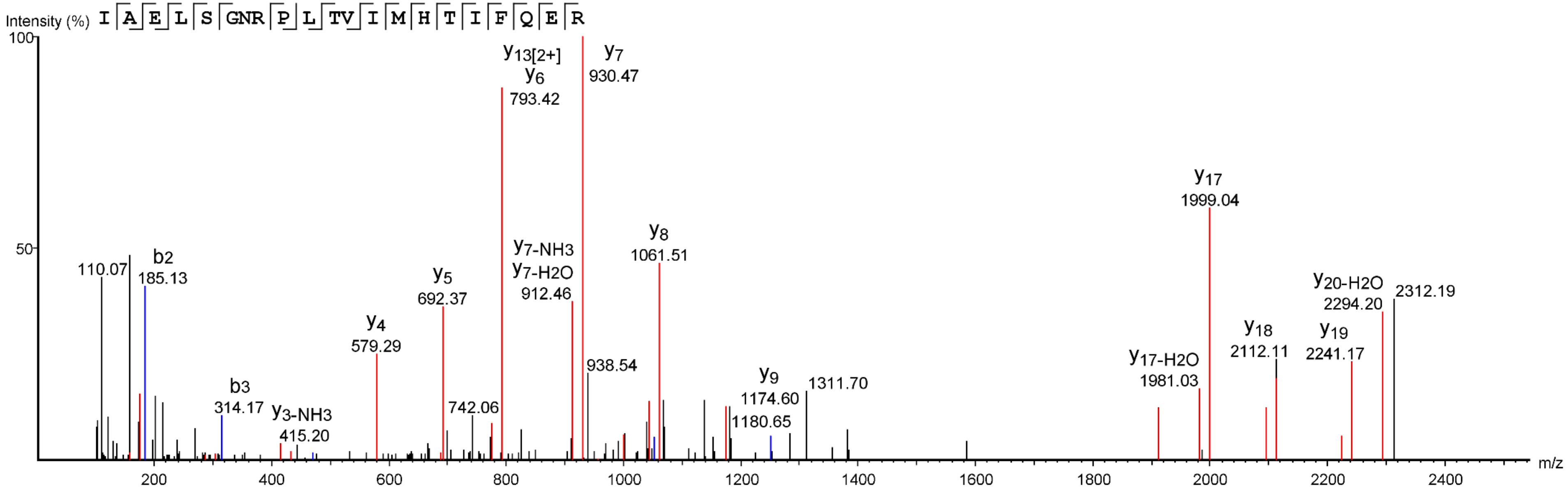

YMT69
Scan
#19407

A-228: PDE4D$_{(411-431)}$: IAELSGNRPLTVIMHTIFQER

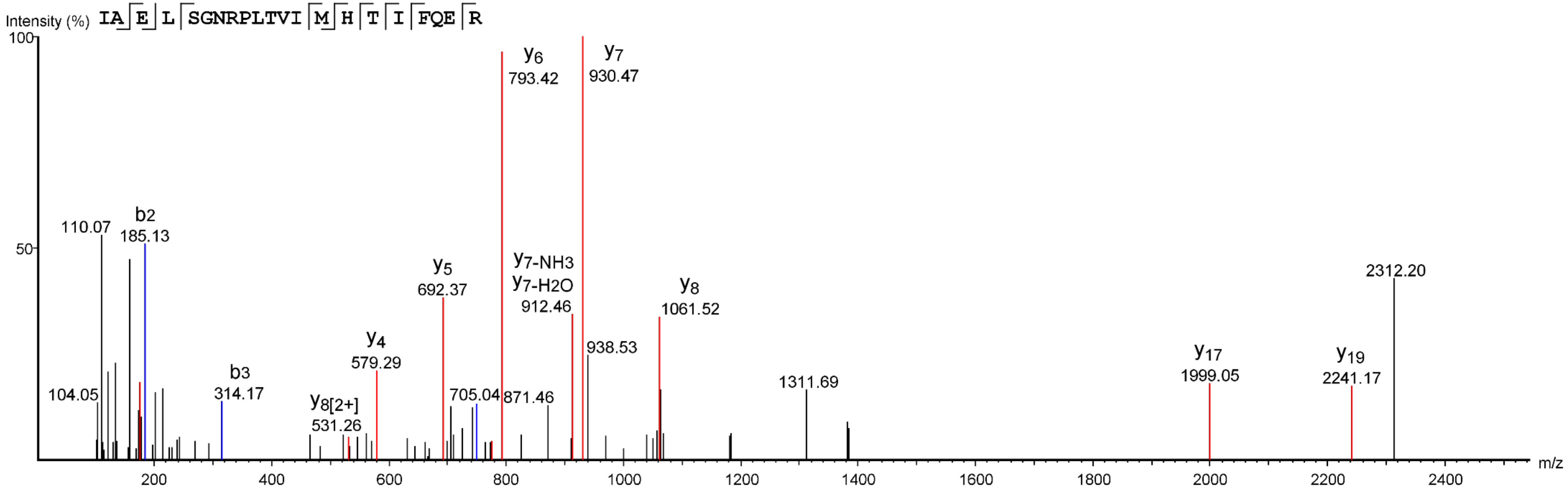

YMT70
Scan
#19782

A-229: PDE4D$_{(411-431)}$: IAELSGNRPLTVIM(+15.99)HTIFQER

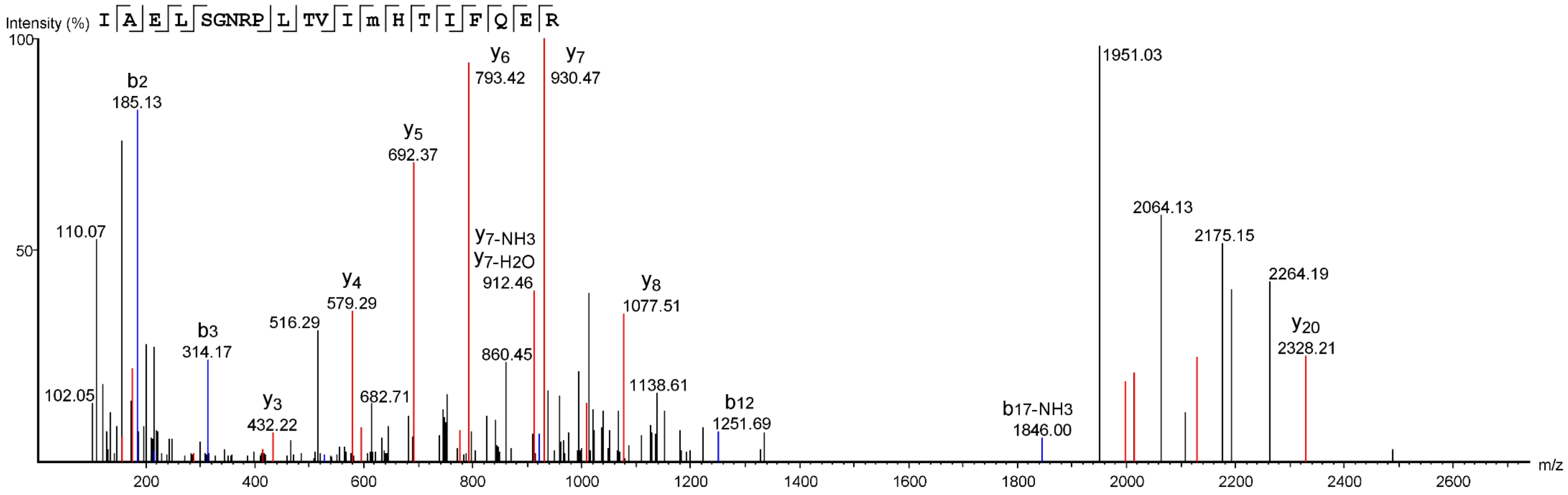

YMT69
Scan
#15539

A-230: PDE4D$_{(411-431)}$: IAELSGNRPLTVIM(+15.99)HTIFQER

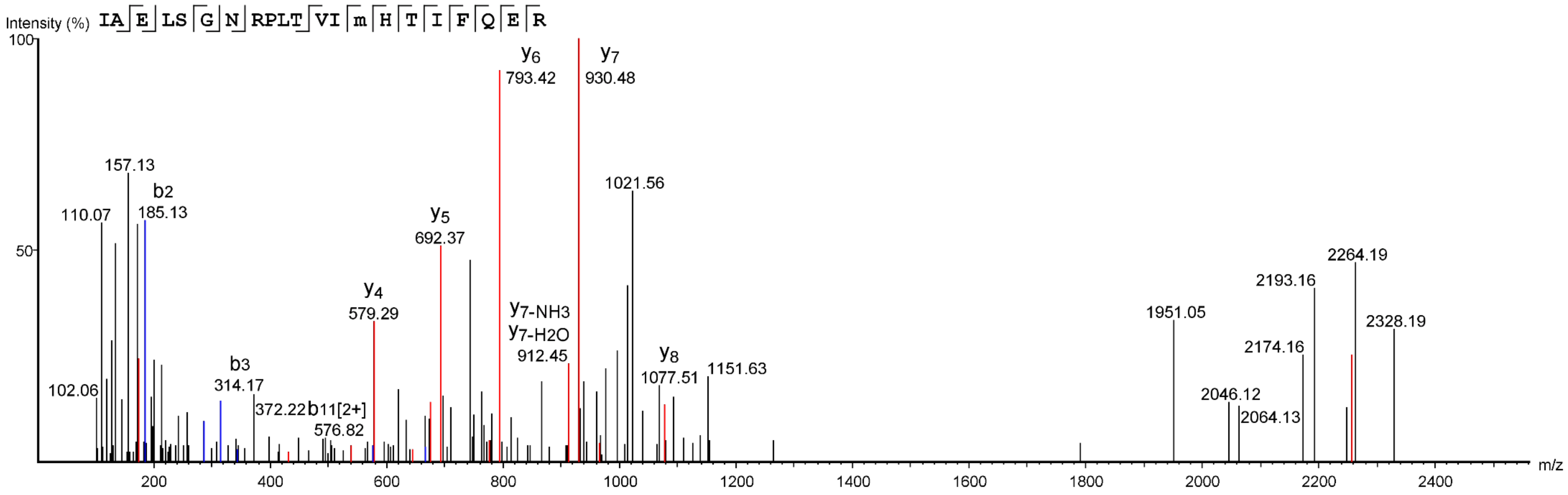

YMT70
Scan
#15807

A-231: PKM$_{(401-422)}$: LAPITSDPTEATAVGAVEASFK

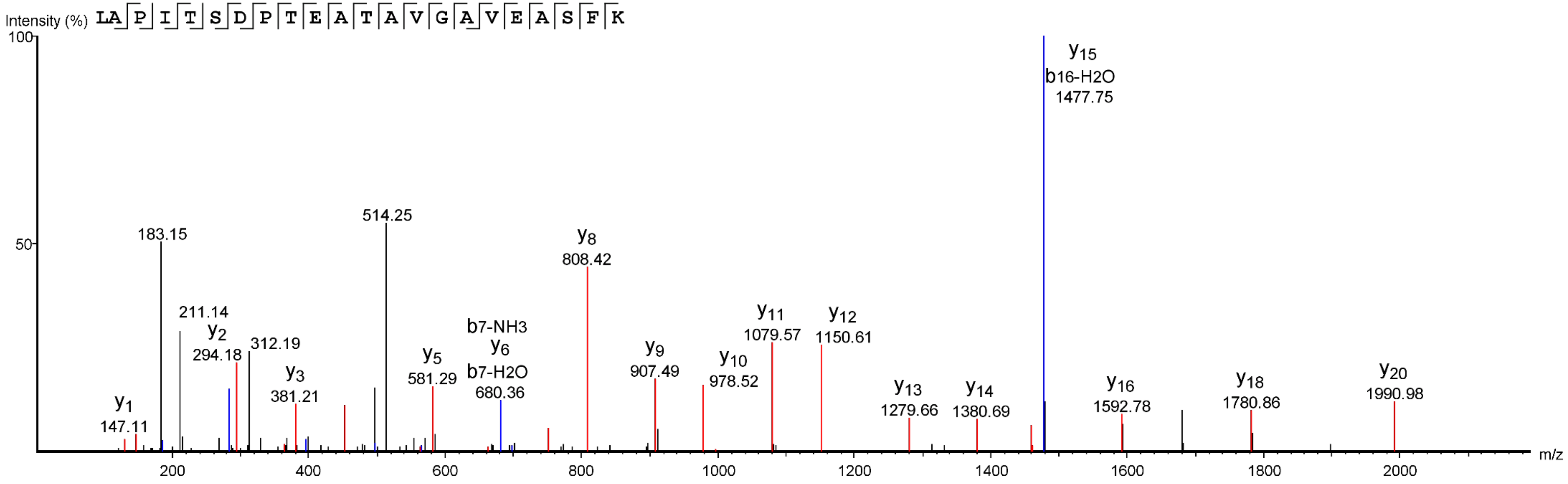

Pep20
Scan #10698

A-232: $PKM_{(401-422)}$: LAP(+15.99)ITSDPTEATAVGAVEASFK

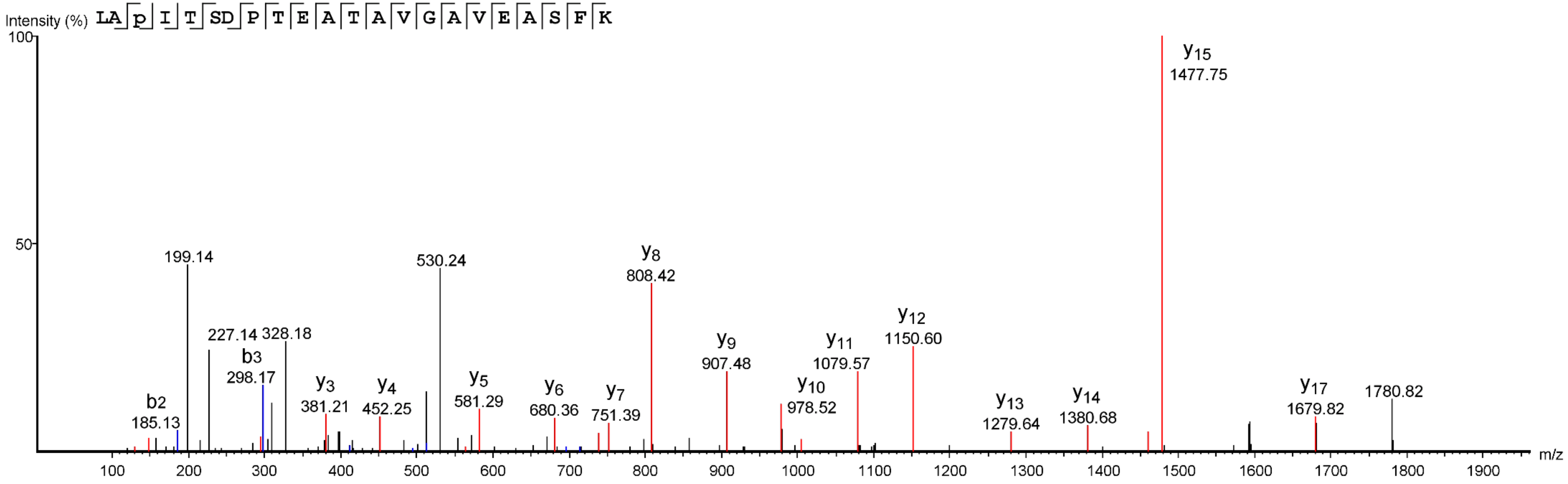

Pep20
Scan #10334

A-233: PKM$_{(401-422)}$: LAPITSDP(+15.99)TEATAVGAVEASFK

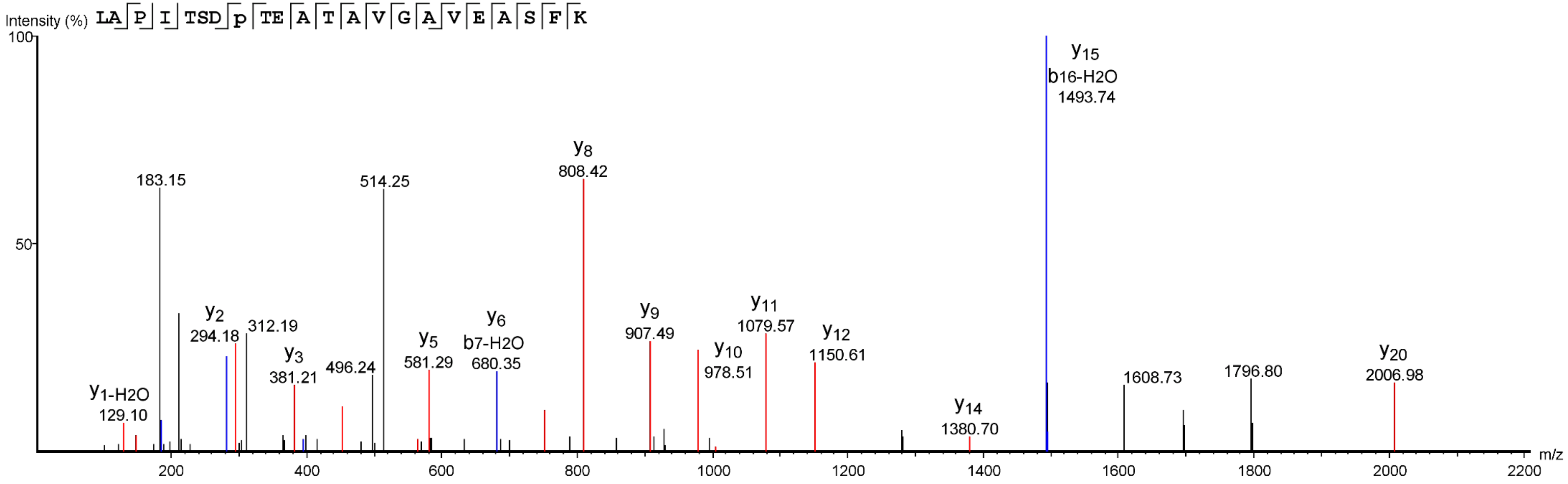

Pep20
Scan #10401

A-234: PKM$_{(401-422)}$: LAP(+15.99)ITSDP(+15.99)TEATAVGAVEASFK

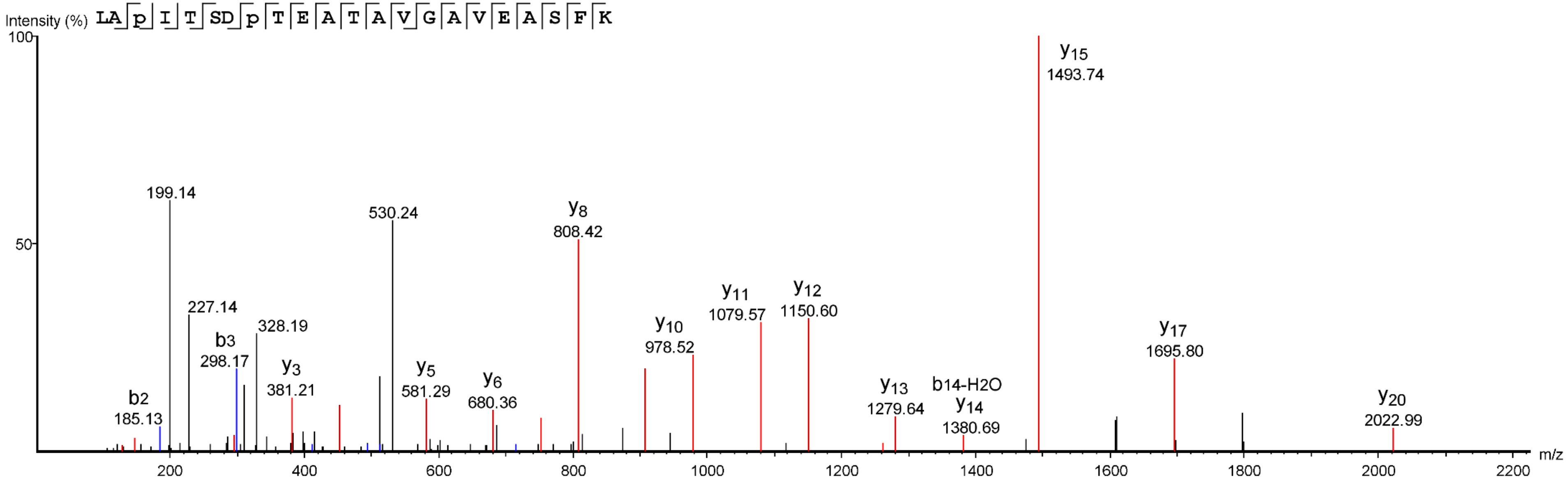

Pep20
Scan #9917

A-235: PKM(401-422): LAPITSDPTEATAVGAVEASFK

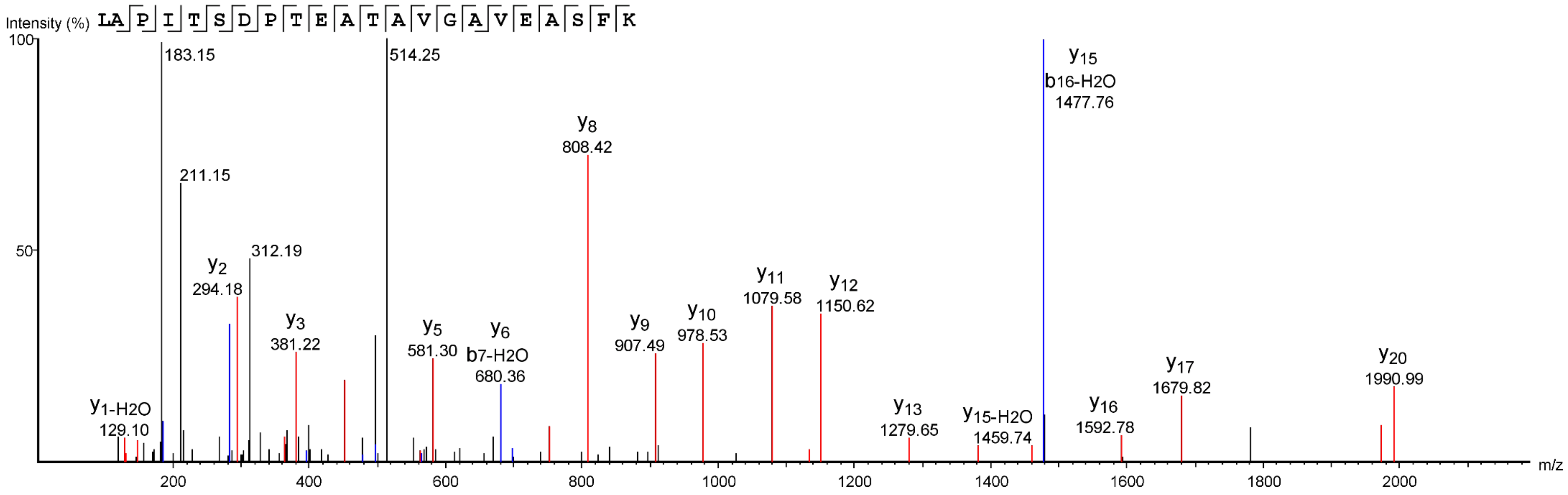

YMT55
Scan #16822

A-236: PKM(401-422): LAPITSDPTEATAVGAVEASFK

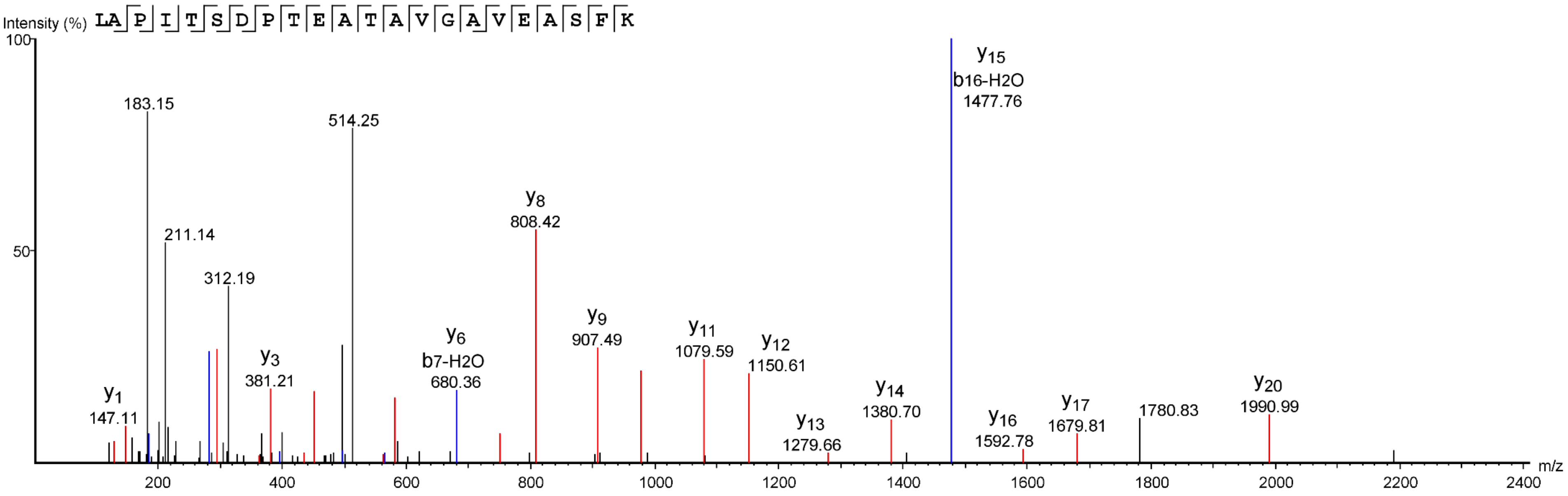

YMT56
Scan #17542

A-237: PPP2R2A$_{(310-330)}$: IWDLNMENRPVETYQVHEYLR

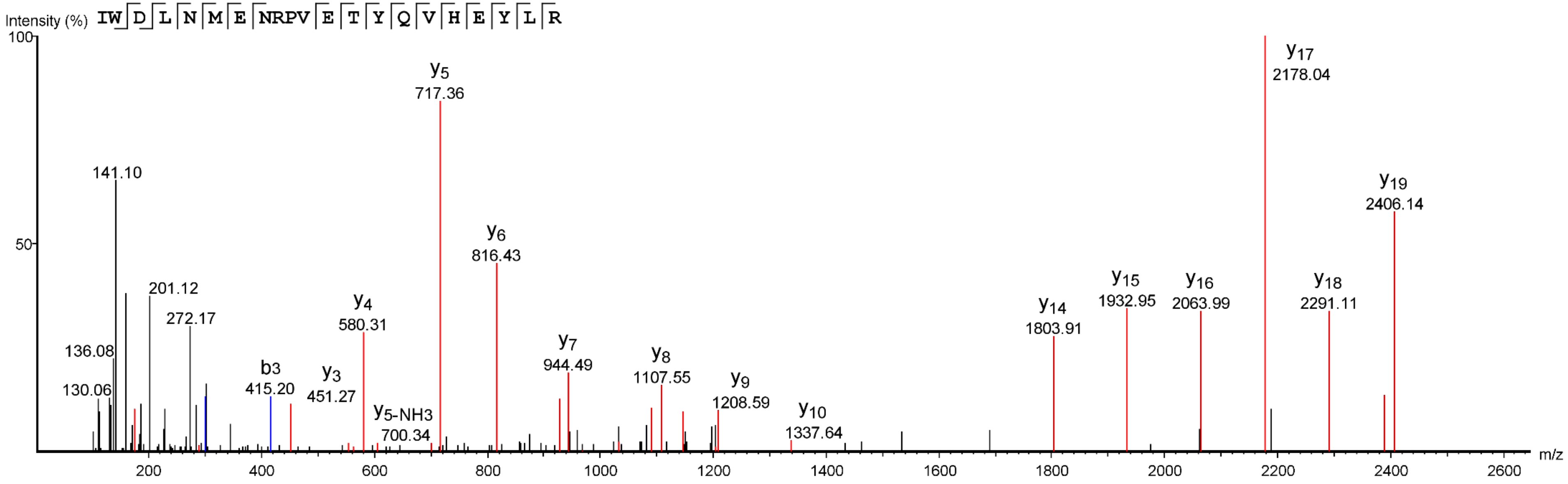

Pep27
Scan #14794

A-238: PPP2R2A$_{(310-330)}$: IWDLNM(+15.99)ENRPVETYQVHEYLR

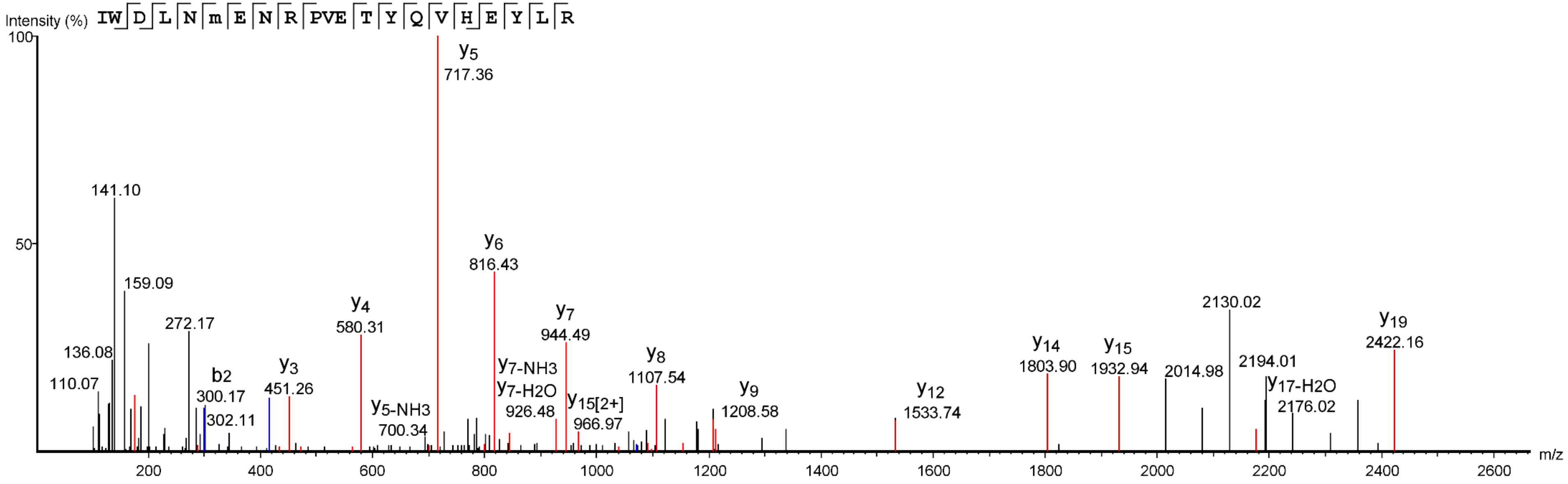

Pep27

Scan #13418

A-239: PPP2R2A(310-330): IWDLNMENRP(+15.99)VETYQVHEYLR

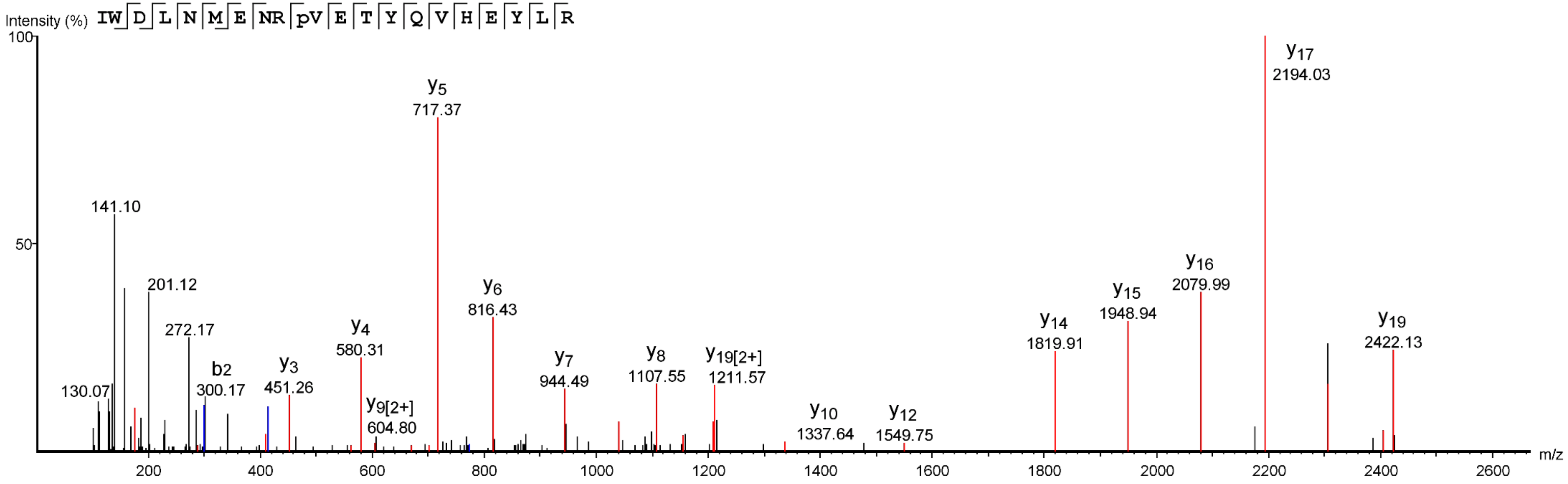

Pep27
Scan #14298

A-240: PPP2R2A(310-330): IWDLNM(+15.99)ENRP(+15.99)VETYQVHEYLR

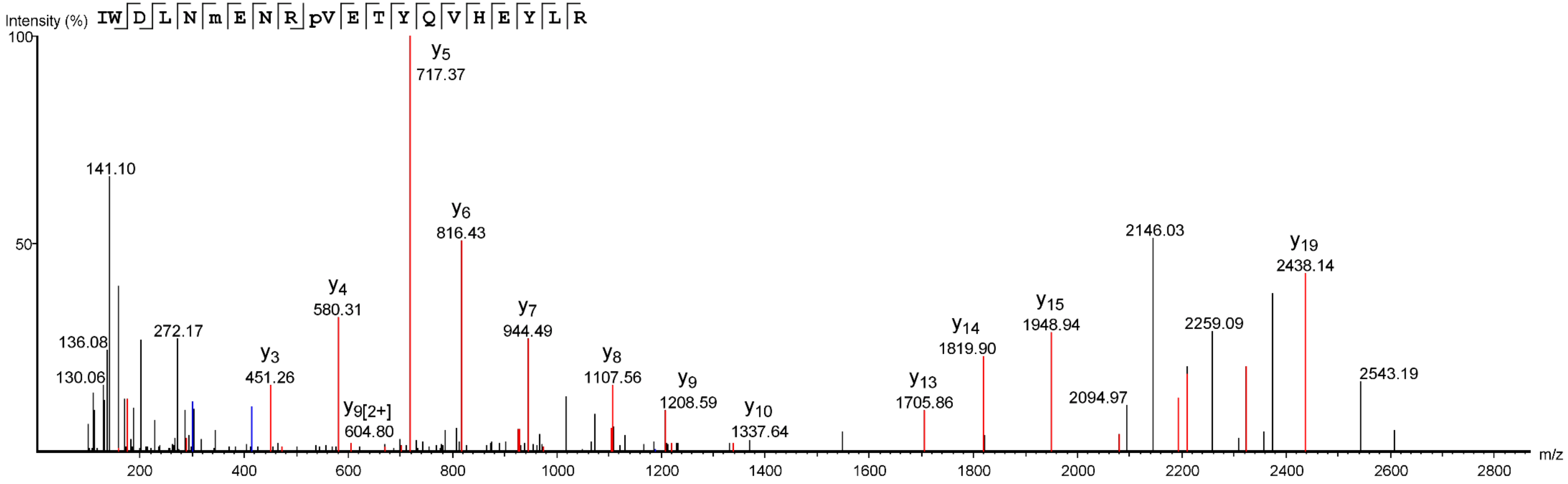

Pep27
Scan #12986

A-241: PPP2R2A$_{(310-330)}$: IWDLNMENRPVETYQVHEYLR

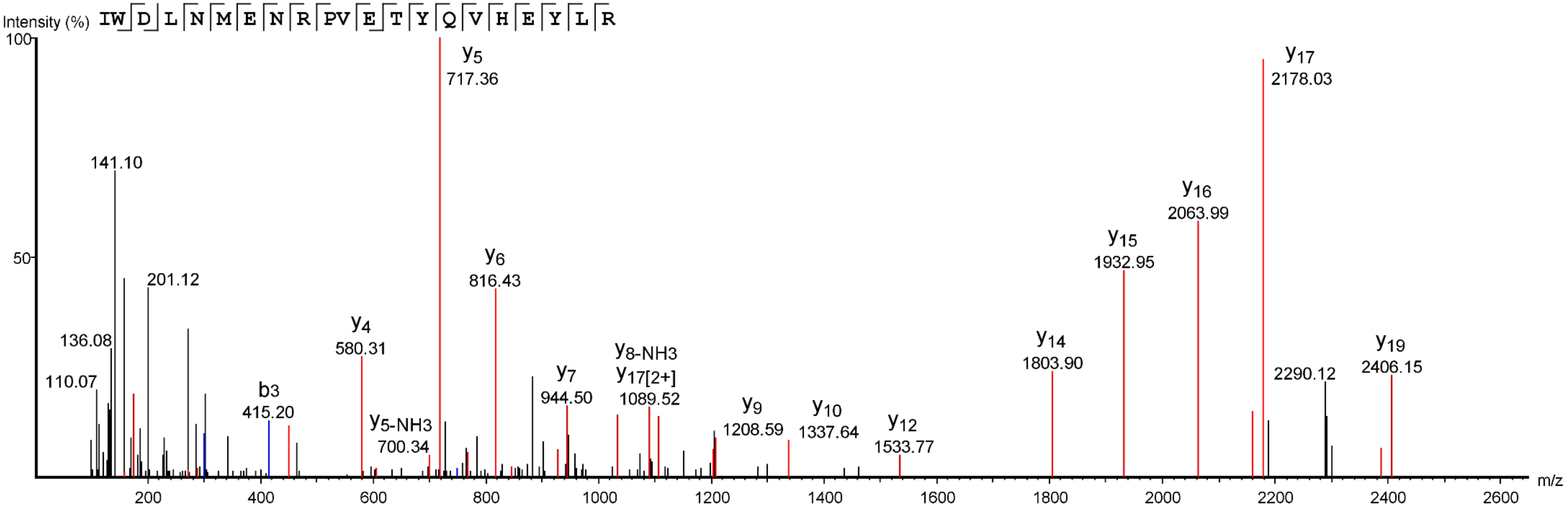

YMT79
Scan #15132

A-242: PPP2R2A(310-330): IWDLNMENRPVETYQVHEYLR

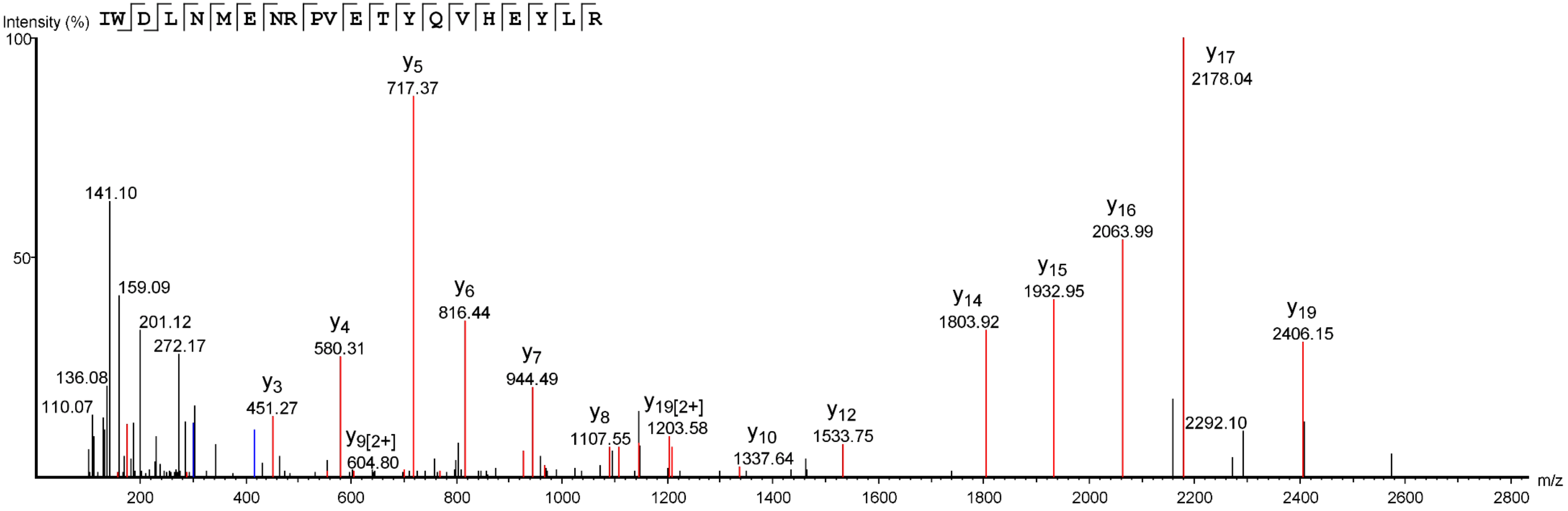

YMT80
Scan #14837

A-243: PPP2R2A$_{(310-330)}$: IWDLNM(+15.99)ENRPVETYQVHEYLR

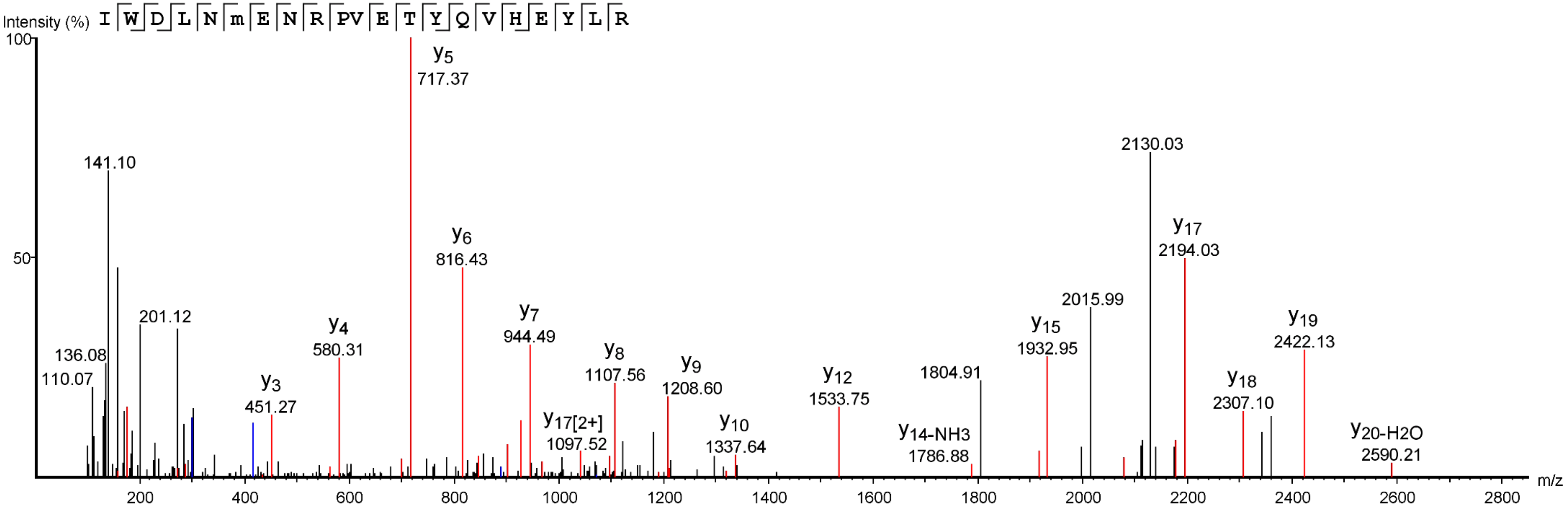

YMT79

Scan #13664

A-244: PPP2R2A$_{(310-330)}$: IWDLNM(+15.99)ENRPVETYQVHEYLR

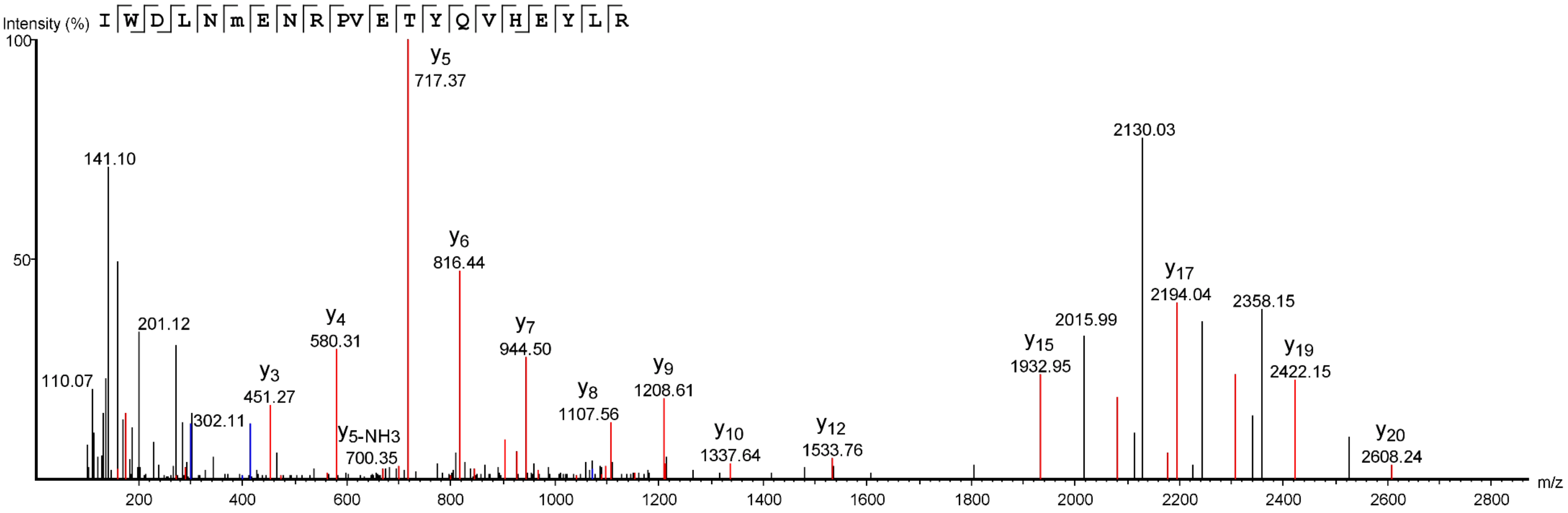

YMT80
Scan #13370

A-245: SPRY2$_{(5-19)}$: AQSGNGSQPLLQTPR

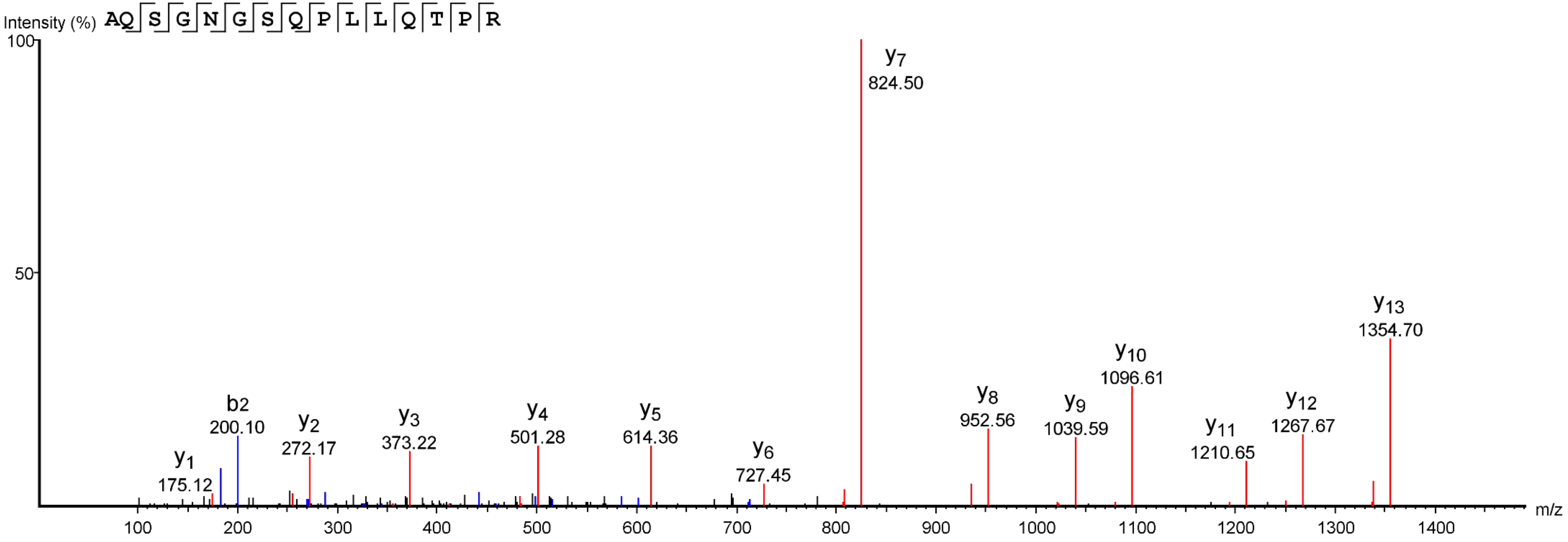

Pep11
Scan #8134

A-246: SPRY2$_{(5-19)}$: AQSGNGSQPLLQTP(+15.99)R

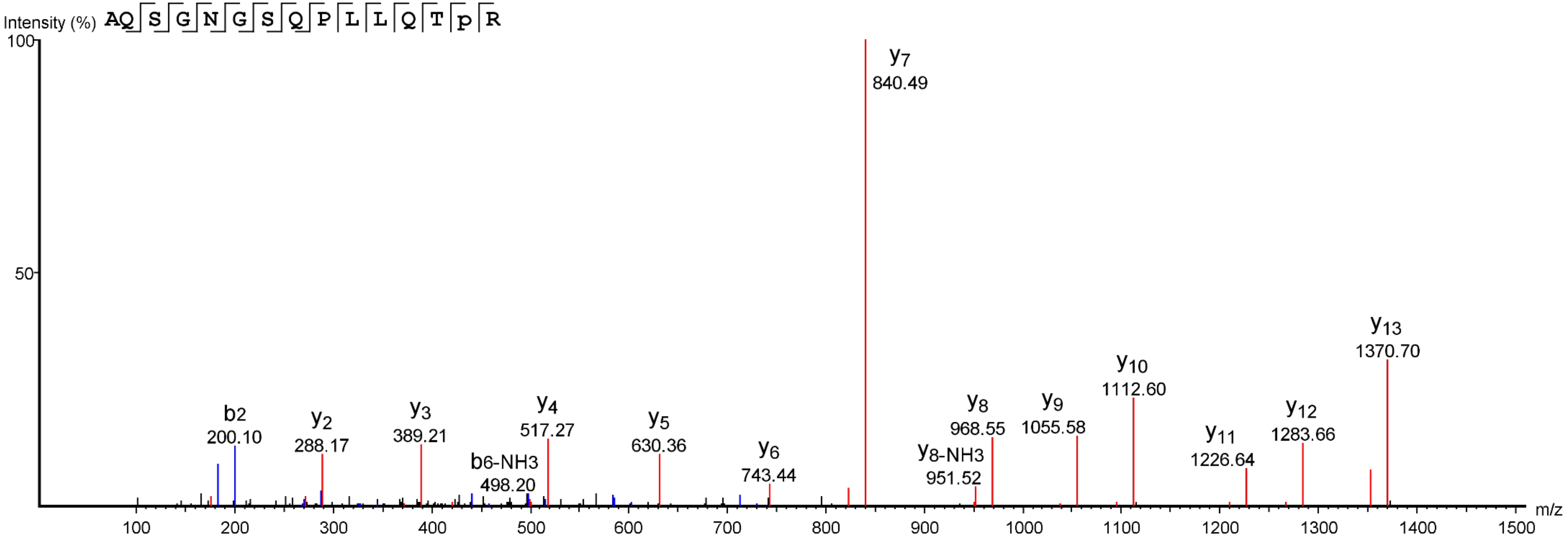

Pep11
Scan #7096

A-249: SPRY2$_{(5-19)}$: AQSGNGSQPLLQTPR

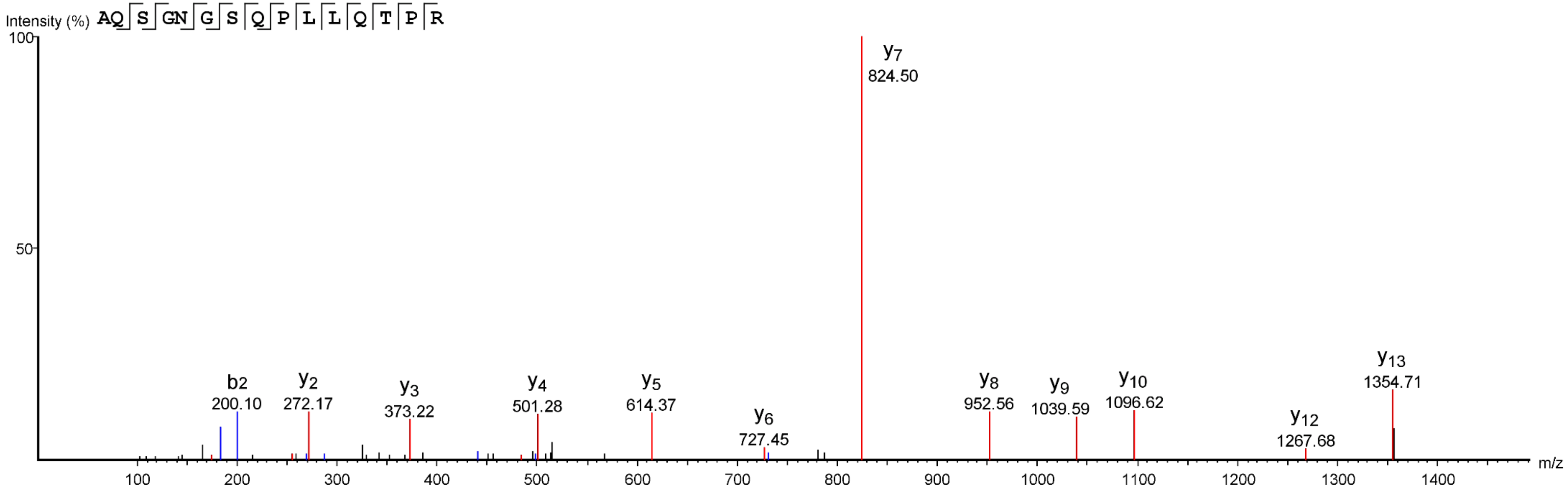

YMT47
Scan #7997

A-250: SPRY2$_{(5-19)}$: AQSGNGSQPLLQTPR

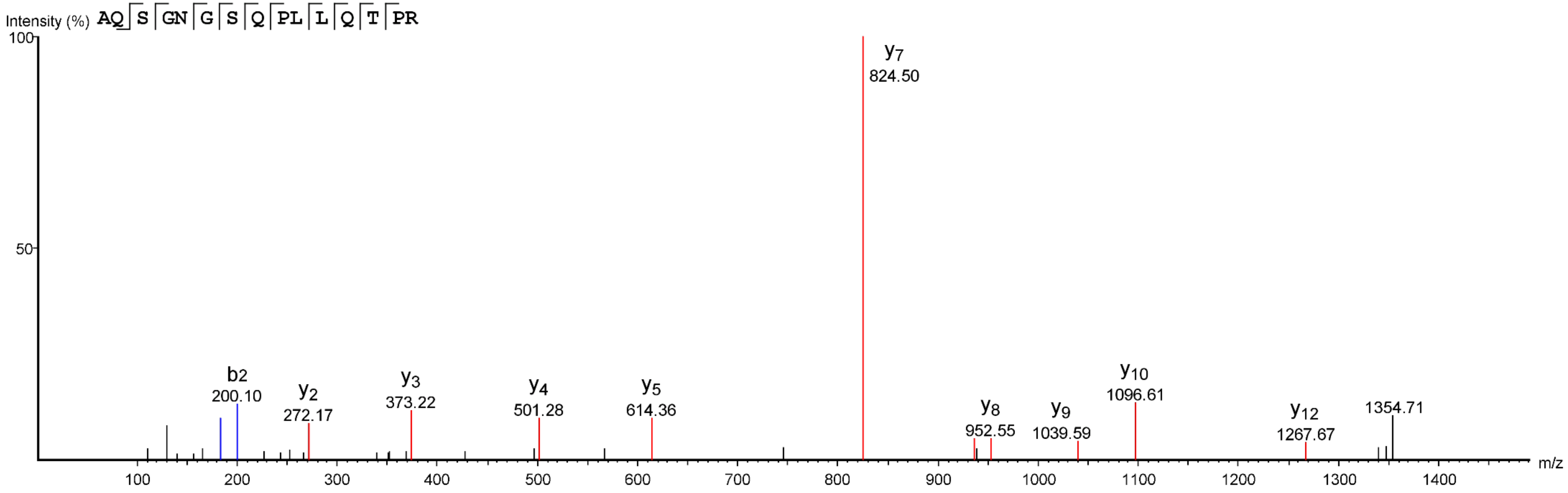

YMT48
Scan #8447

A-251: SPRY2$_{(135\text{-}151)}$: LLGSSFSSGPVADGIIR

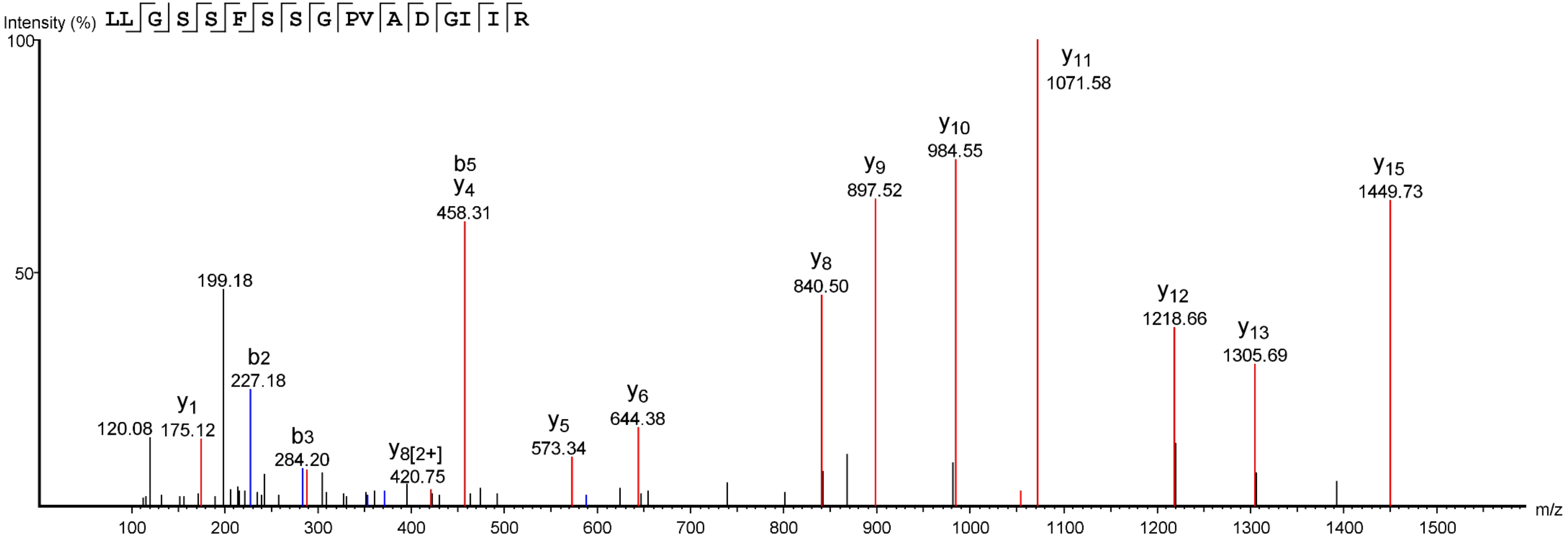

Pep17
Scan #15189

A-252: SPRY2$_{(135-151)}$: LLGSSFSSGP(+15.99)VADGIIR

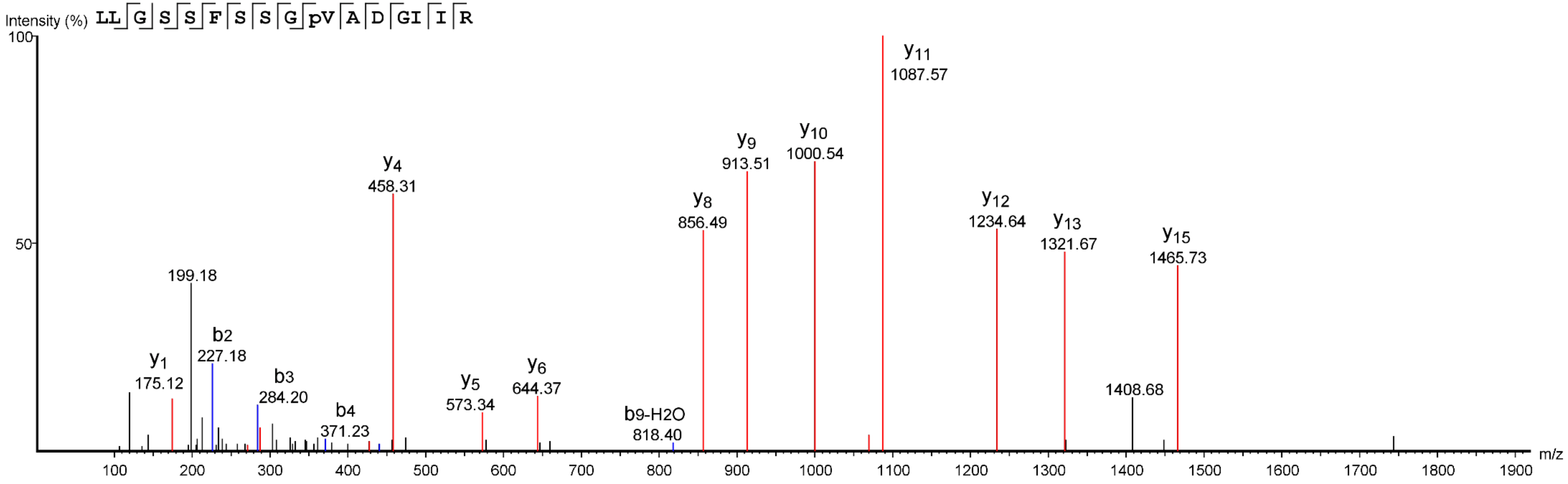

Pep17
Scan #14371

A-253: SPRY2$_{(135-151)}$: LLGSSFSSGPVADGIIR

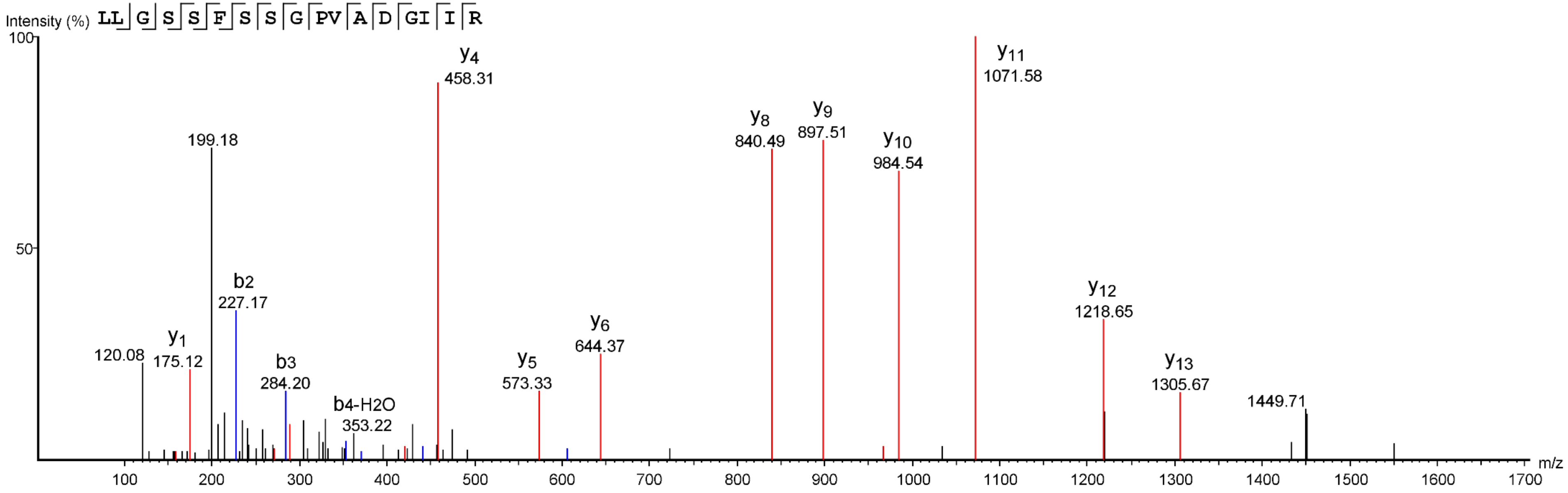

YMT29
Scan #18161

A-254: SPRY2$_{(135-151)}$: LLGSSFSSGPVADGIIR

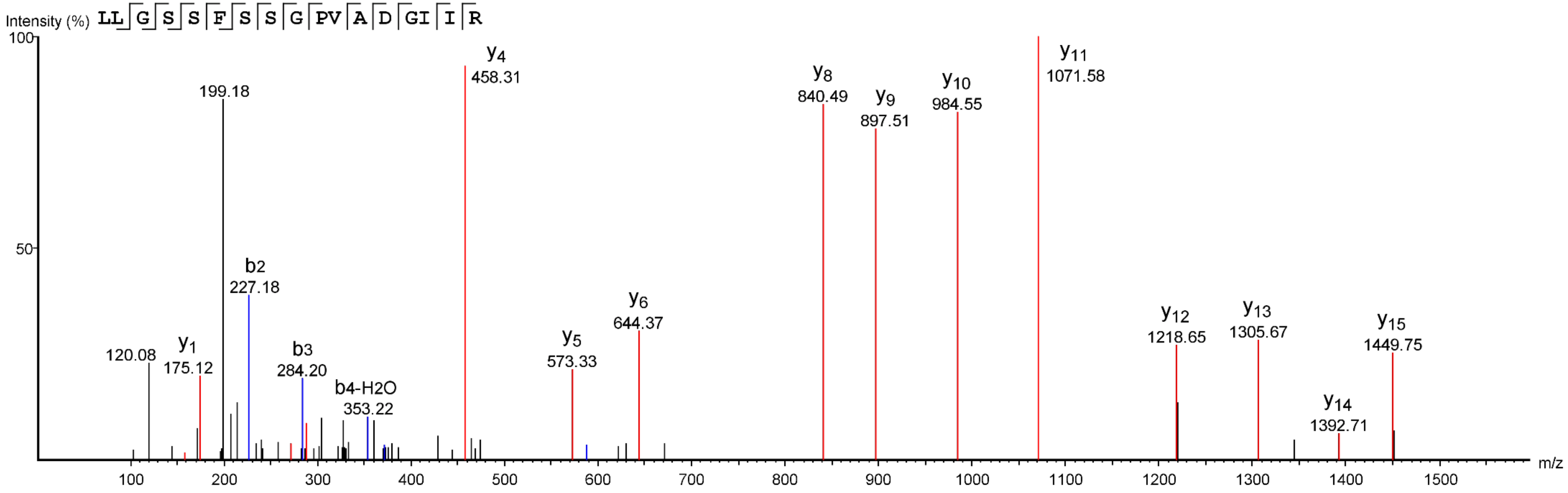

YMT30
Scan #18016

A-257: SPRY2$_{(135-151)}$: LLGSSFSSGPVADGIIR

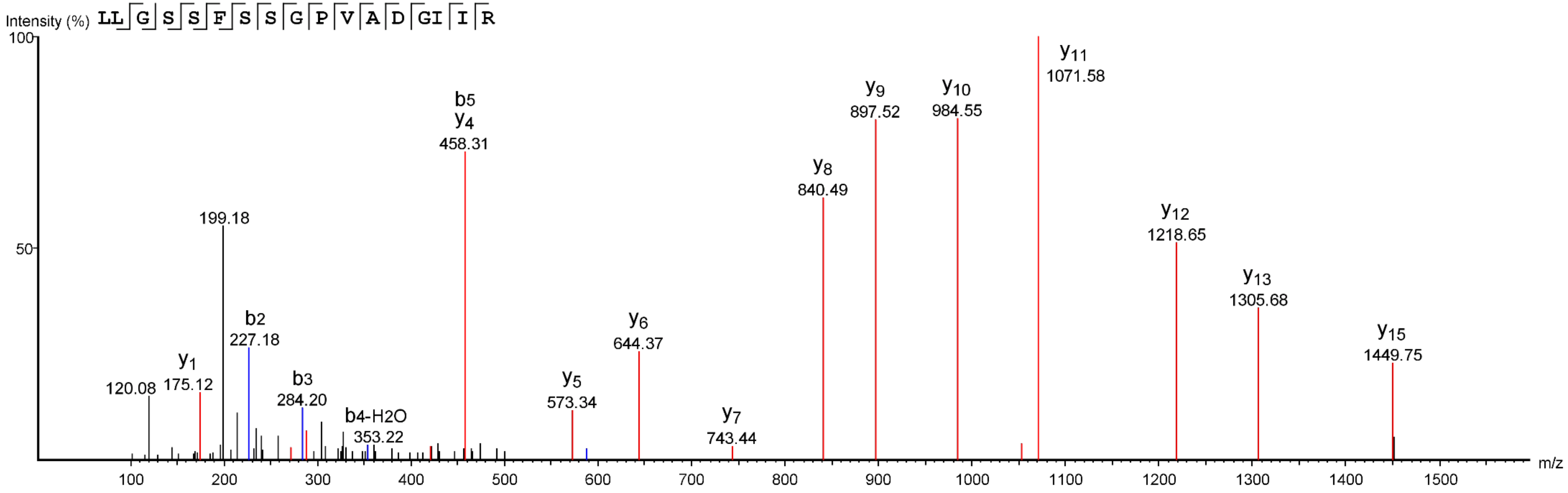

YMT47
Scan #15516

A-258: SPRY2$_{(135\text{-}151)}$: LLGSSFSSGPVADGIIR

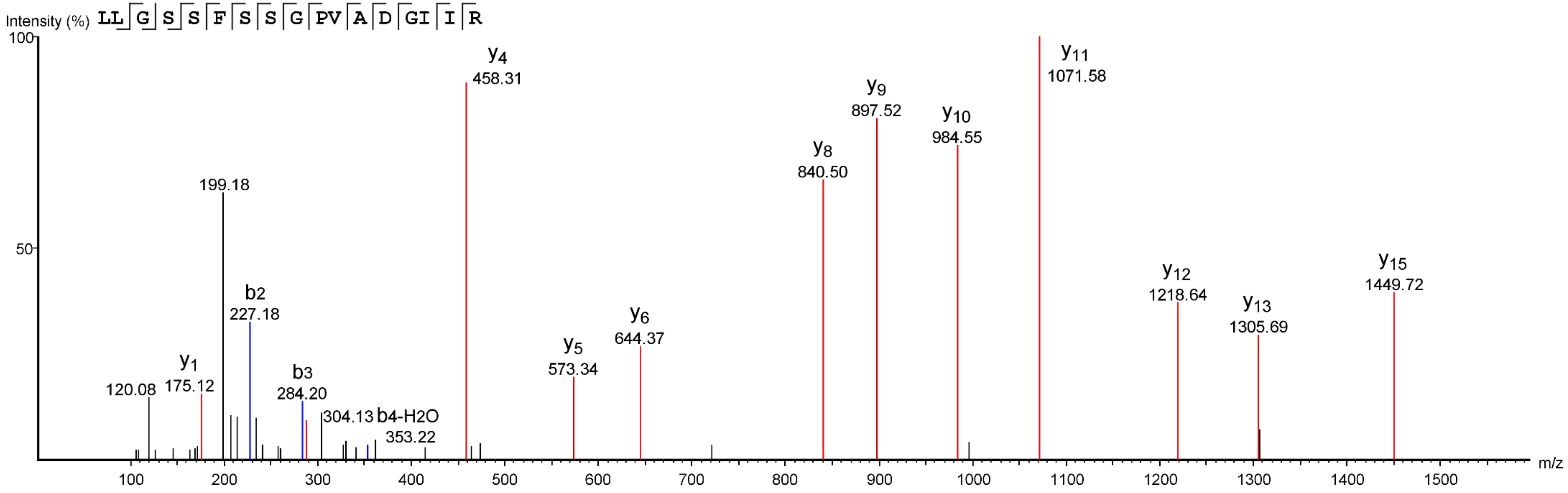

YMT48
Scan #16792

A-259: SPRY2$_{(156-168)}$: SELKPGELKPLSK

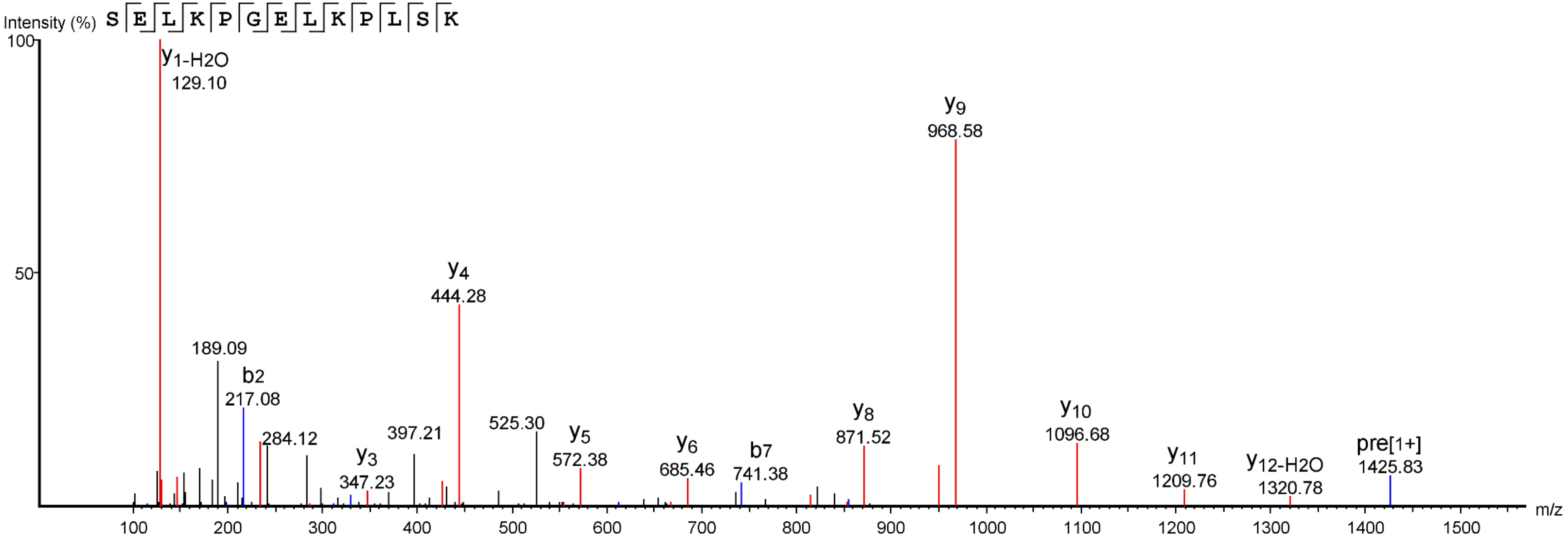

Pep17
Scan #5908

A-260: SPRY2$_{(156-168)}$: SELKP(+15.99)GELKPLSK

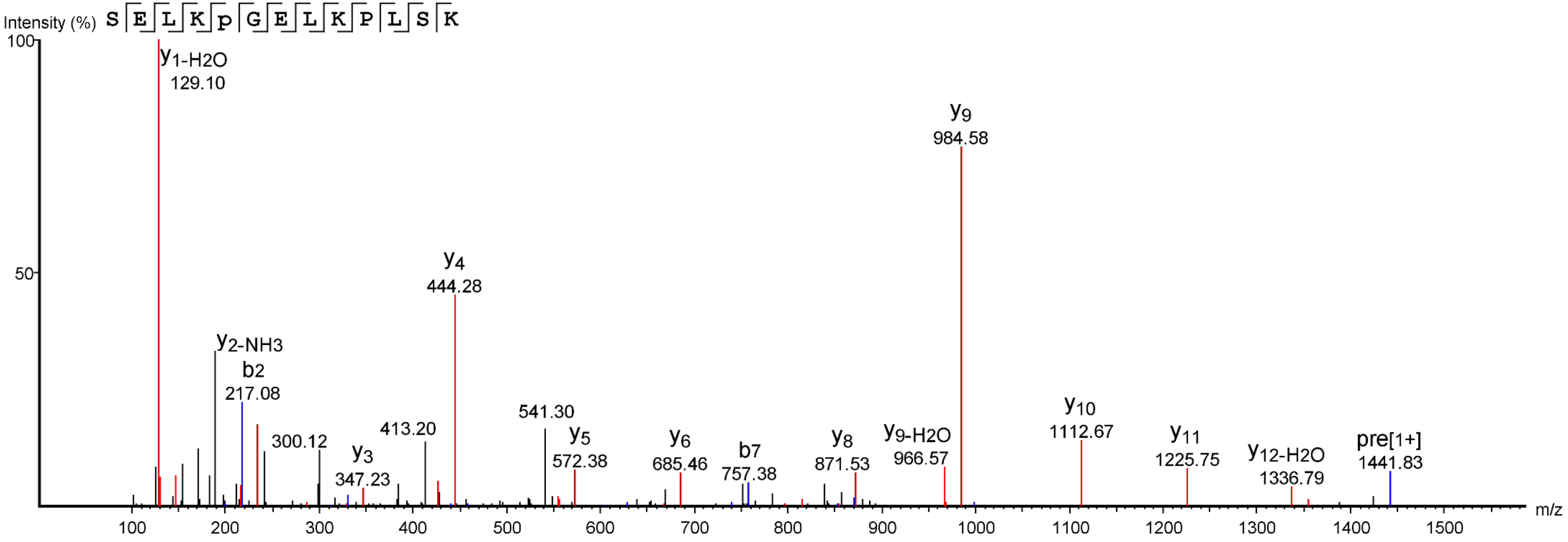

Pep17
Scan #4732

A-261: SPRY2(156-168): SELKPGELKPLSK

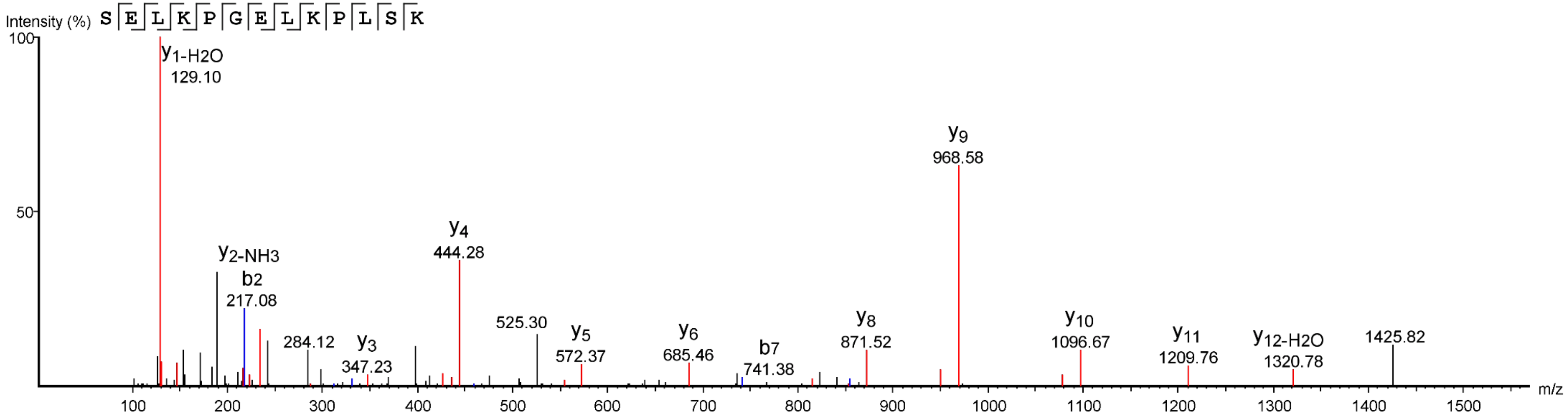

YMT29
Scan #7044

A-262: SPRY2$_{(156-168)}$: SELKPGELKPLSK

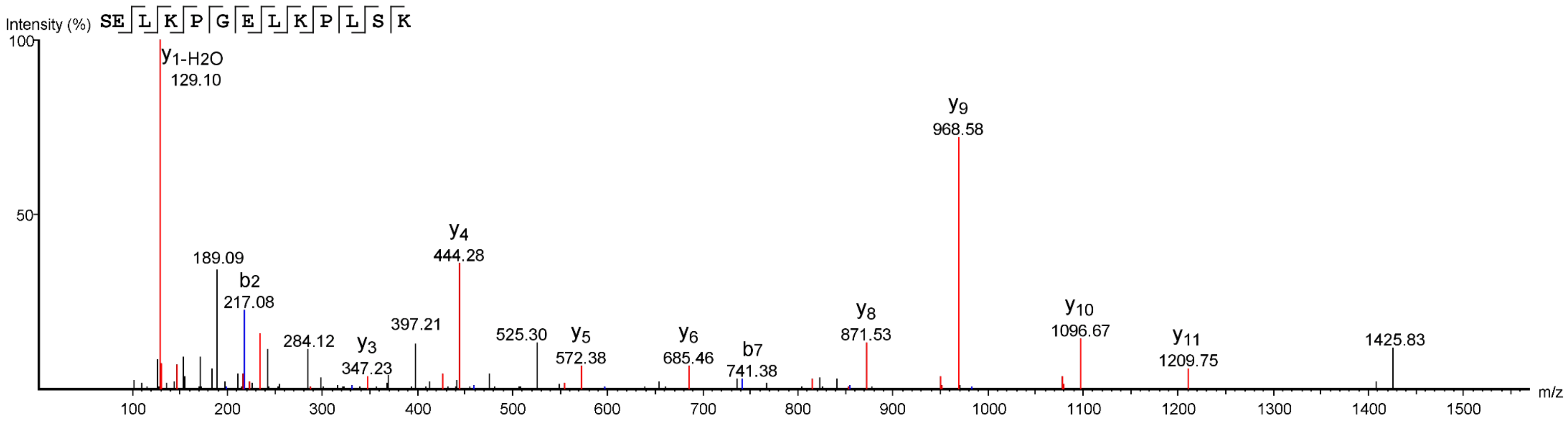

YMT30

Scan #6676

A-265: SPRY2$_{(156-168)}$: SELKPGELKPLSK

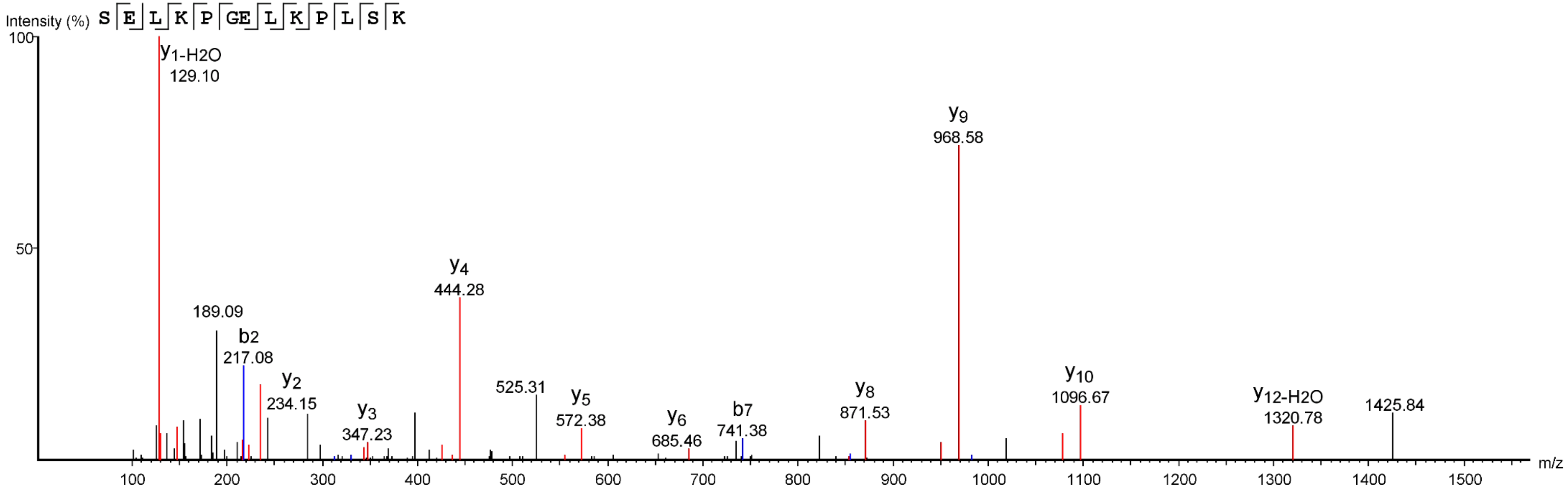

YMT47
Scan #6470

A-266: SPRY2$_{(156-168)}$: SELKPGELKPLSK

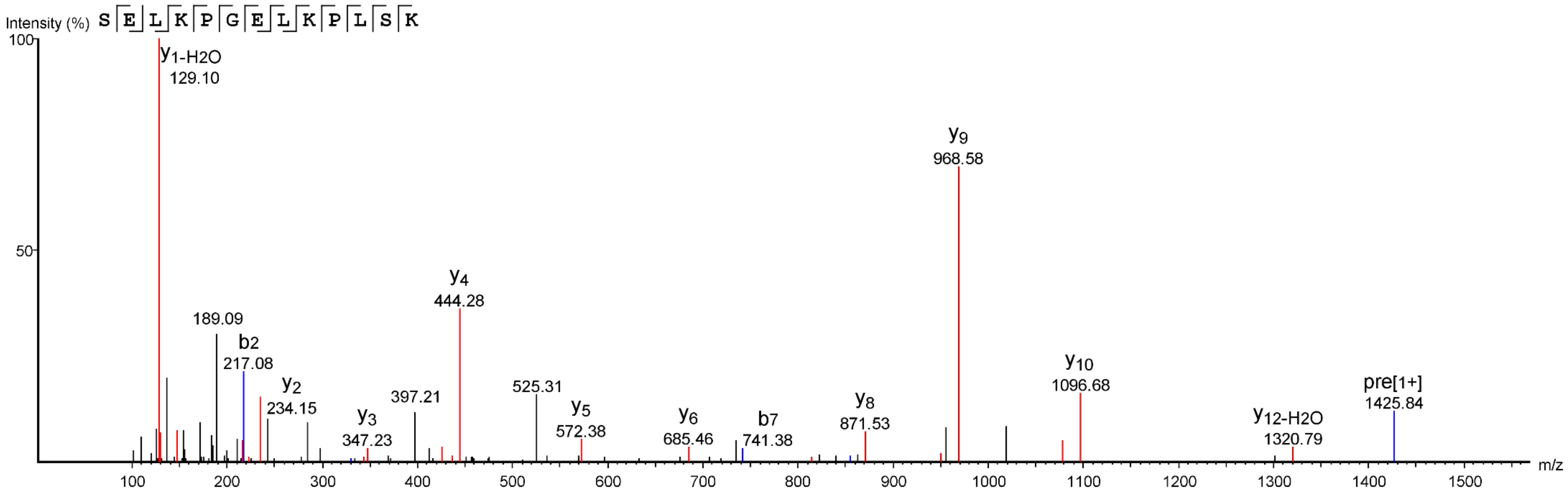

YMT48

Scan #6889

A-267: TELO2(362-377): AVLIC(+57.02)LAQLGEPELRR

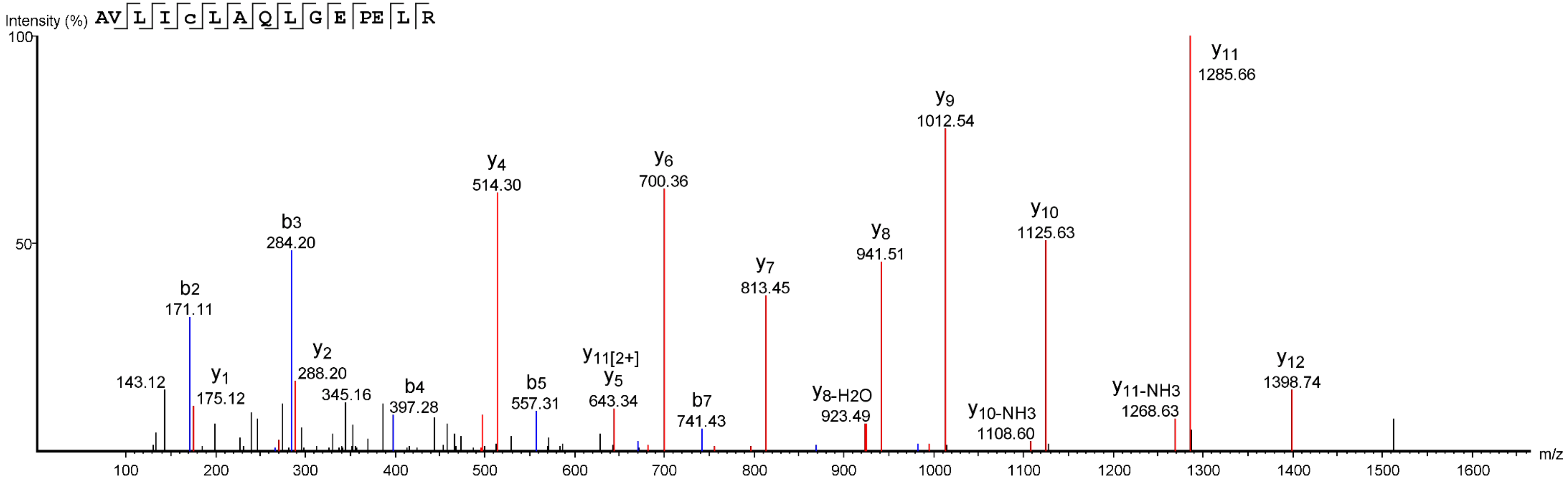

Pep11

Scan #18513

A-268: TELO2$_{(362-377)}$: AVLIC(+57.02)LAQLGEP(+15.99)ELRR

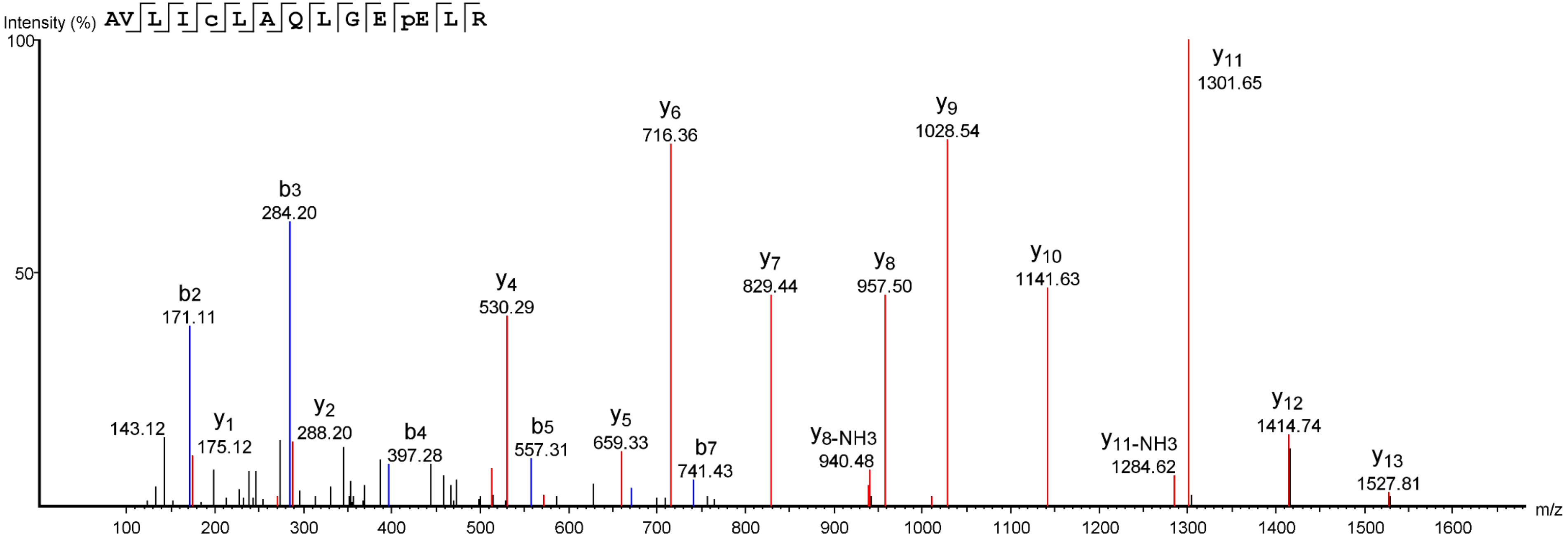

Pep11

Scan #17748

A-269: TELO2$_{(362-377)}$: AVLIC(+57.02)LAQLGEPELRR

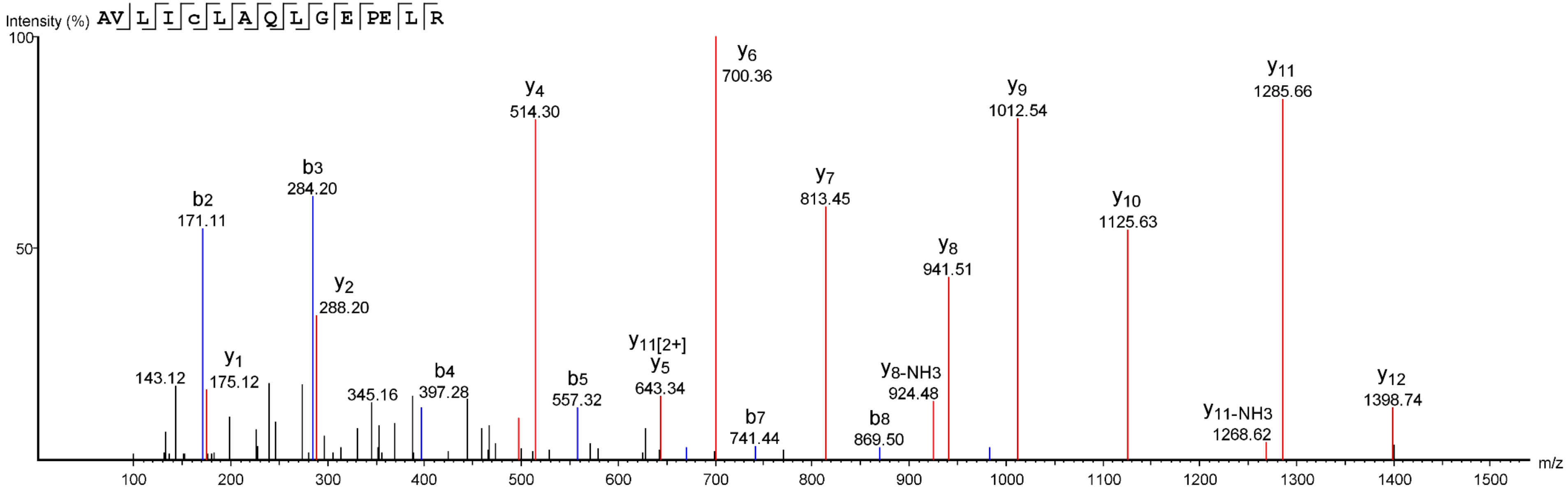

YMT47
Scan #18415

A-270: TELO2$_{(362-377)}$: AVLIC(+57.02)LAQLGEPELRR

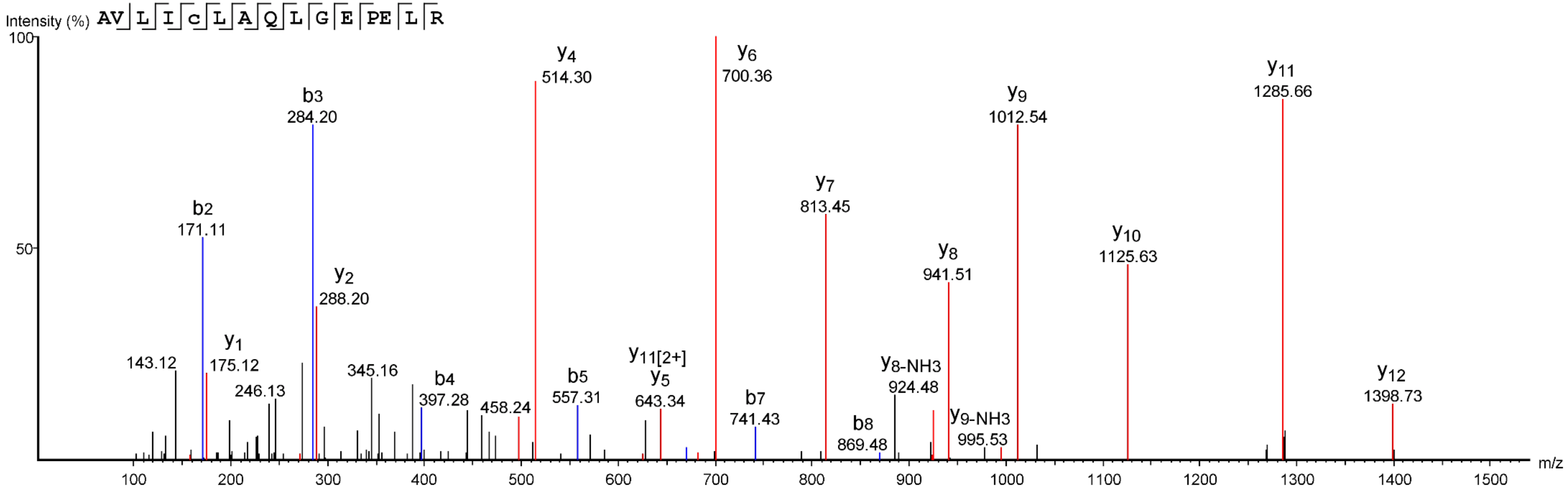

YMT48

Scan #19915

A-271: THRA(153-176): SLQQRPEPTPEEWDLIHIATEAHR

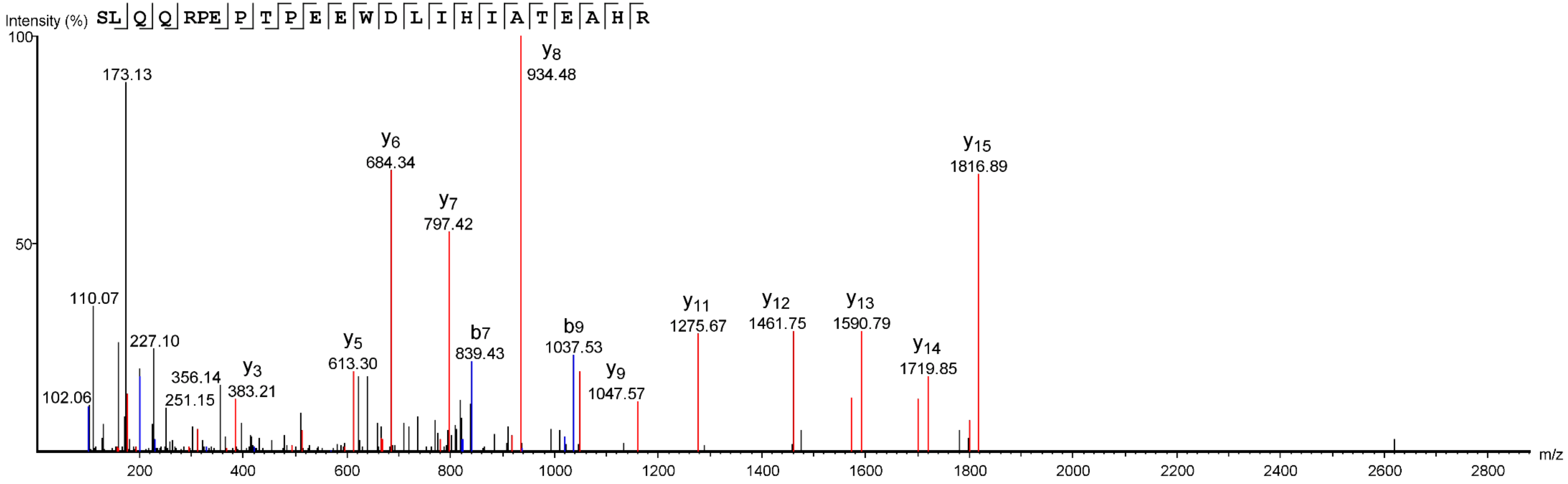

Pep21
Scan #11851

A-272: THRA(153-176): SLQQRPEP(+15.99)TPEEWDLIHIATEAHR

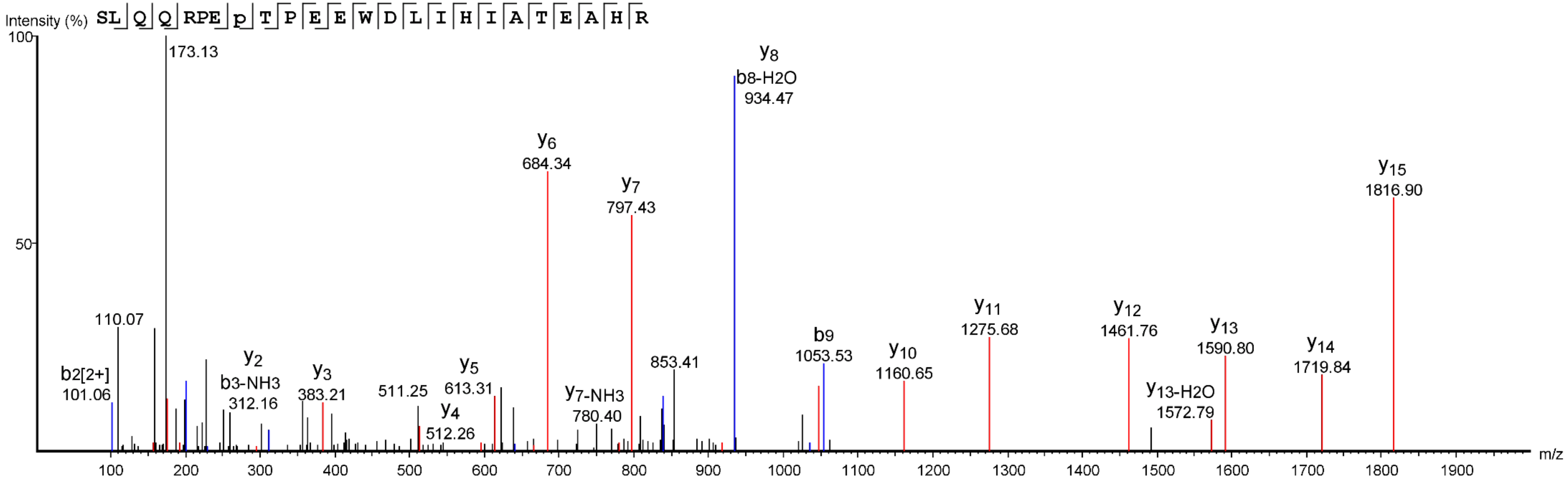

Pep21
Scan #11112

A-273: THRA(153-176): SLQQRPEPTP(+15.99)EEWDLIHIATEAHR

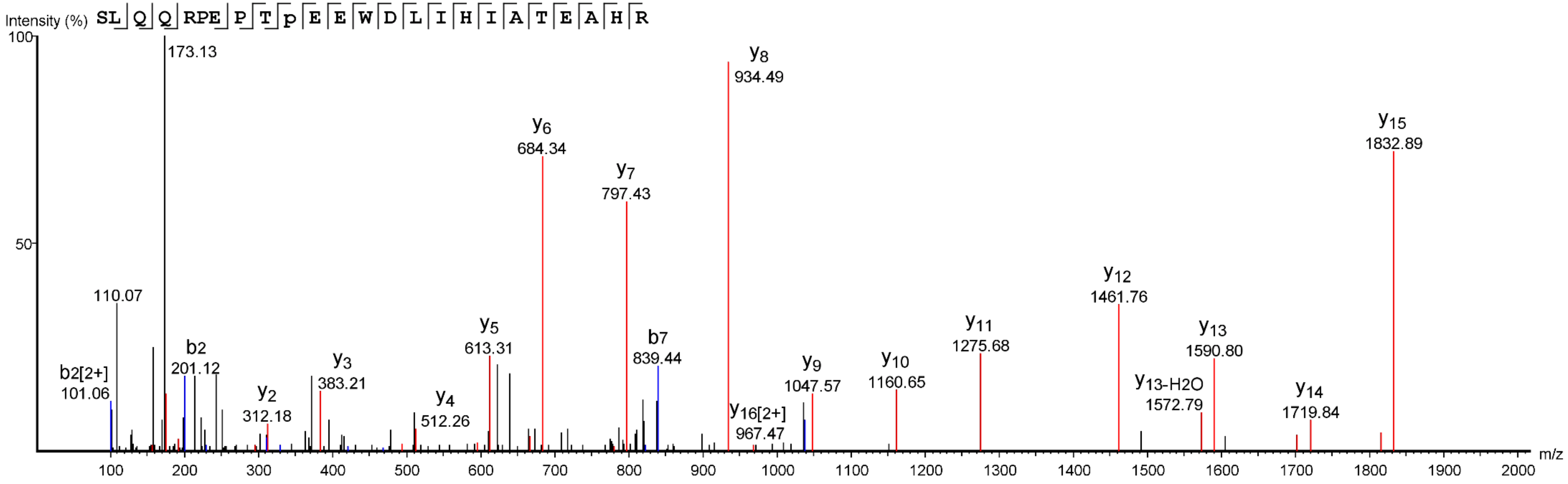

Pep21
Scan #11848

A-274: THRA(153-176): SLQQRPEP(+15.99)TP(+15.99)EEWDLIHIATEAHR

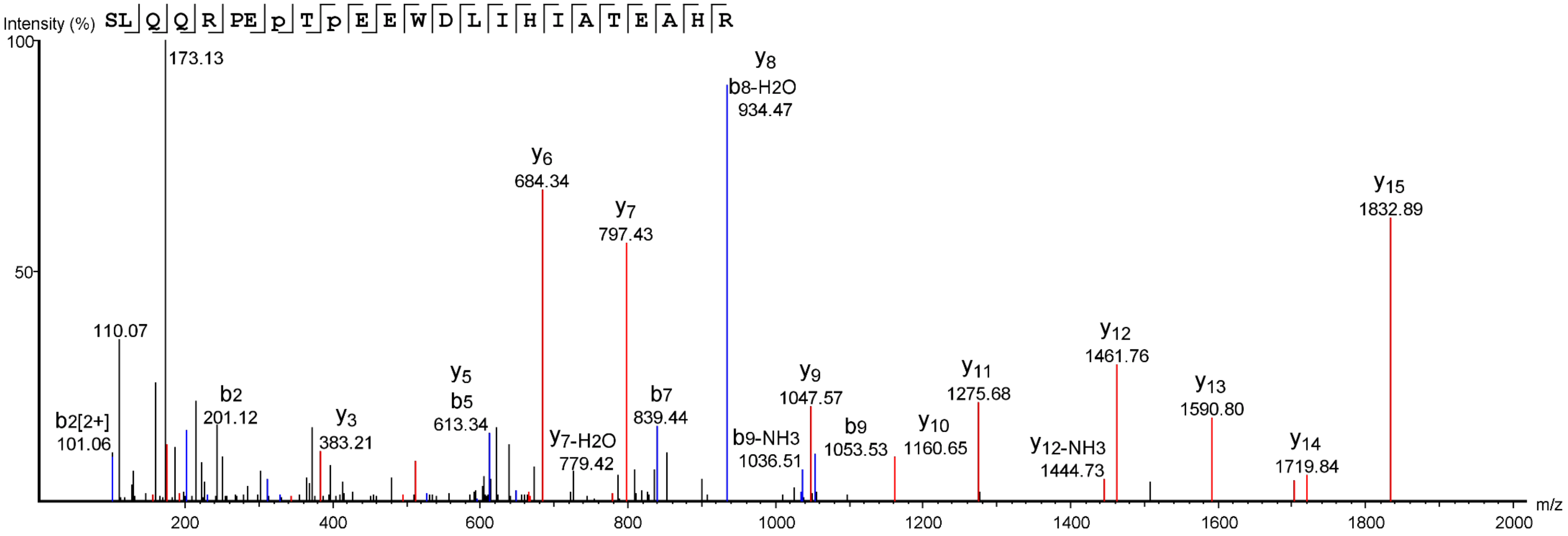

Pep21
Scan #10760

A-275: AKT1$_{(308-328)}$: TFC(+57.02)GTPEYLAPEVLEDNDYGR

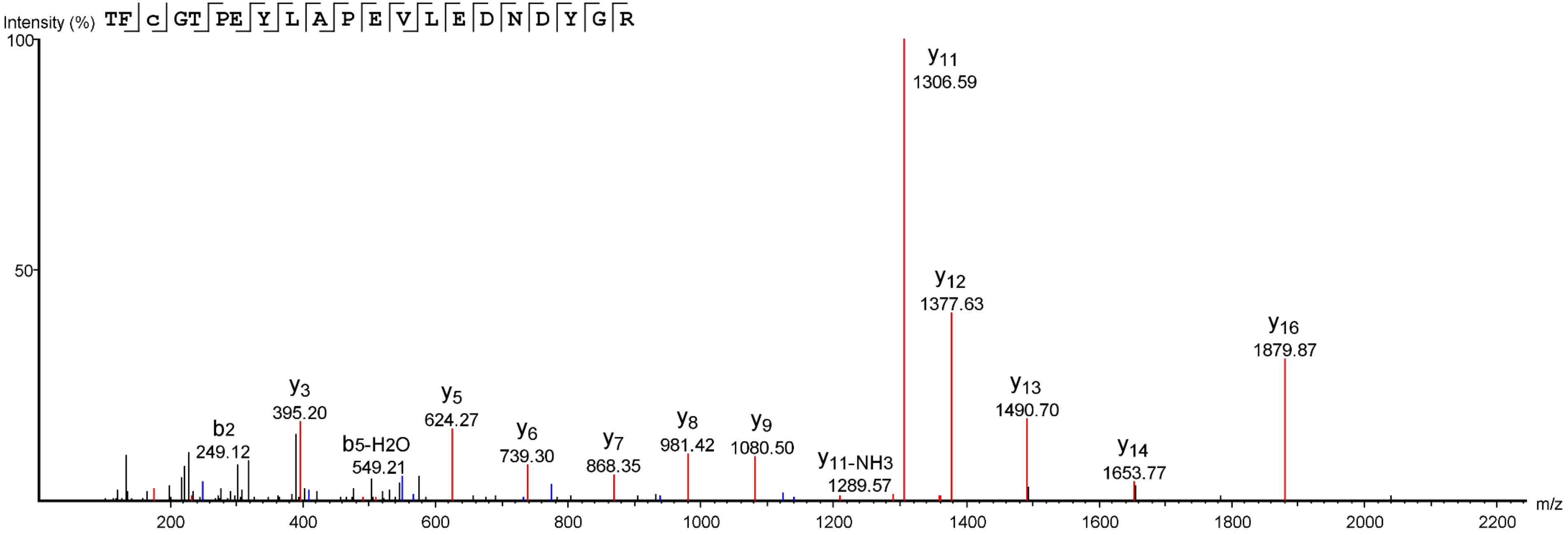

YMT83
Scan #21196

A-276: AKT1(308-328): TFC(+57.02)GTPEYLAPEVLEDNDYGR

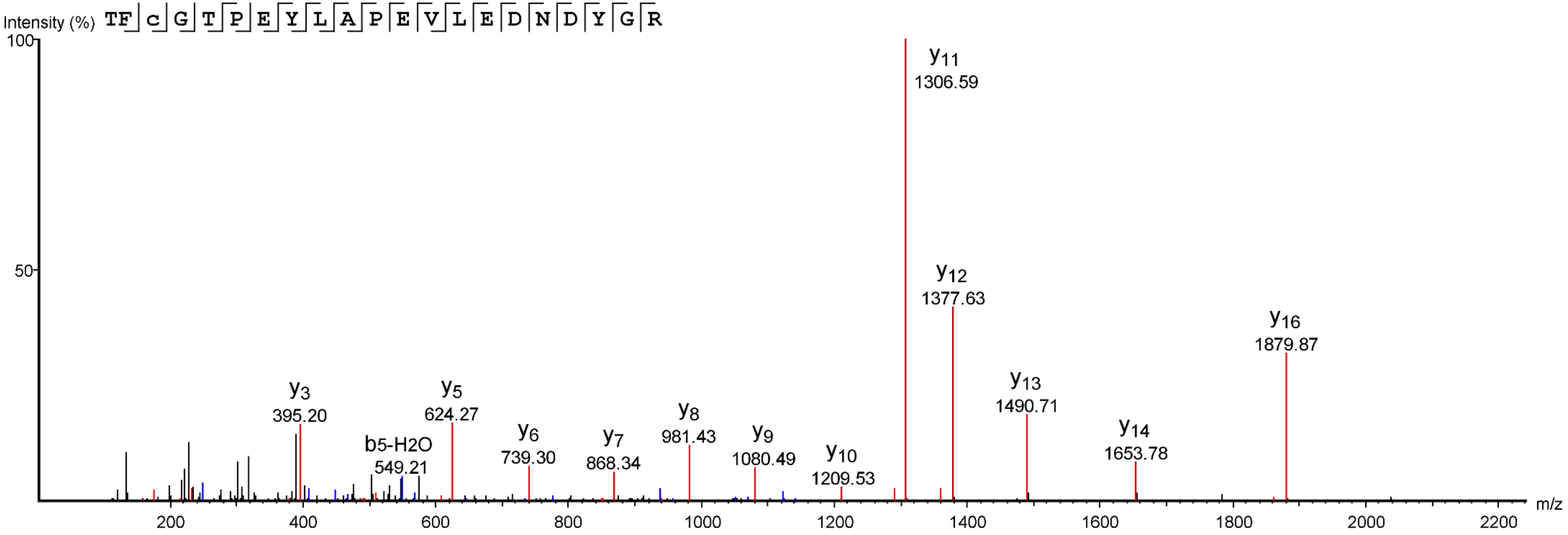

YMT84
Scan #20036

A-277: AKT1$_{(308-328)}$: TFC(+57.02)GTPEY(+15.99)LAPEVLEDNDYGR

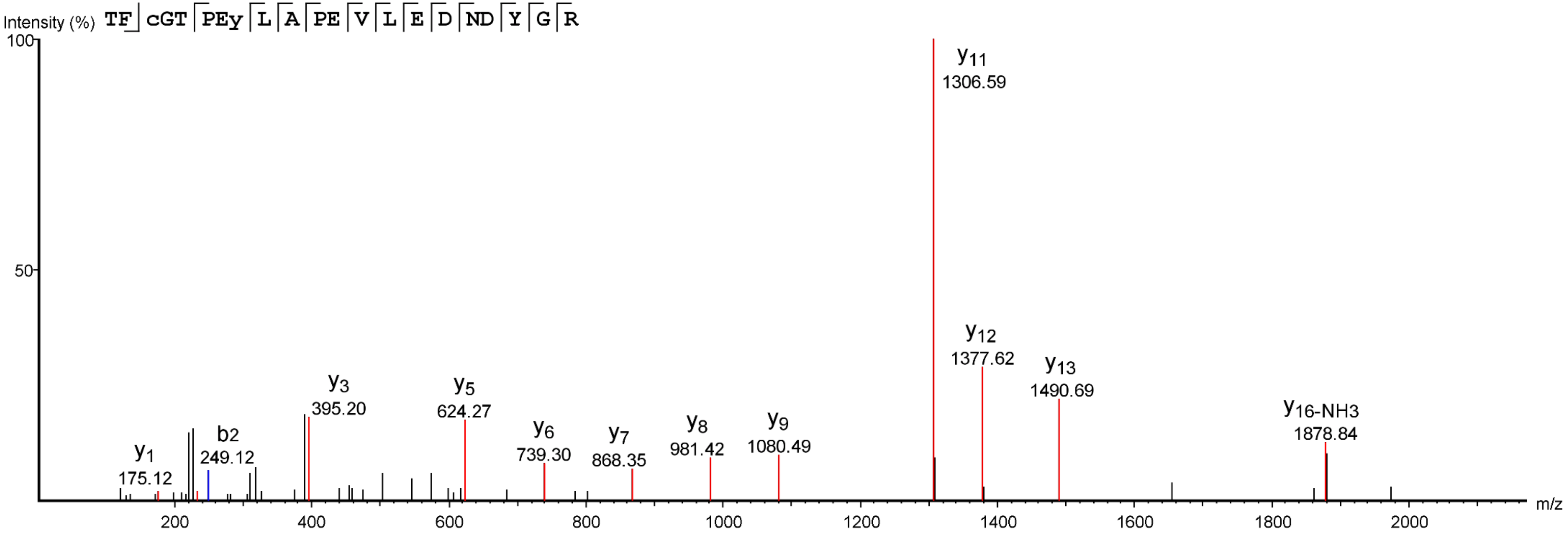

YMT84
Scan #19642

A-282: TP53$_{(140-156)}$: TC(+57.02)PVQLWVDSTPPPGTR

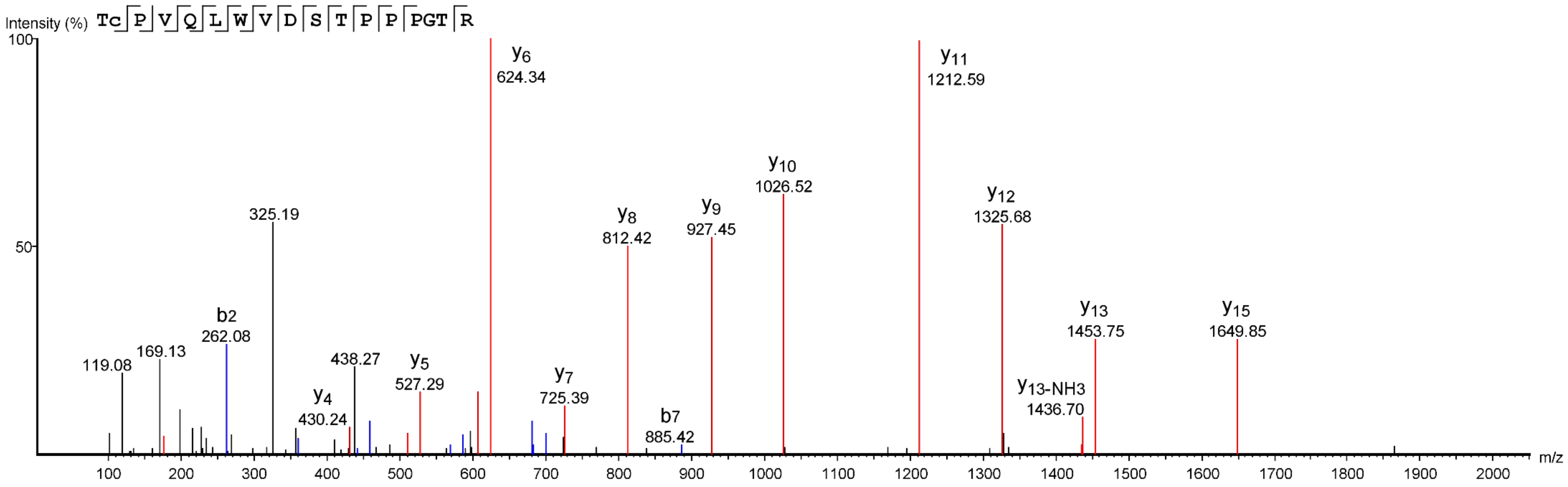

Pep27
Scan #15258

A-283: TP53$_{(140-156)}$: TC(+57.02)P(+15.99)VQLWVDSTPPPGTR

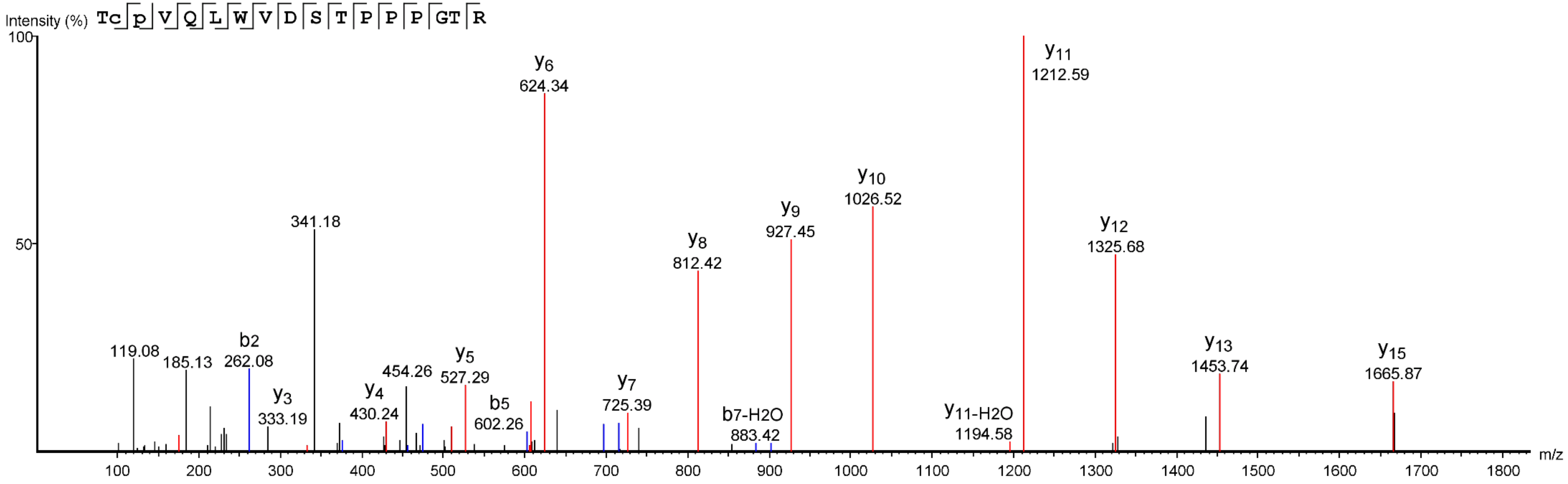

Pep27
Scan #14282

A-284: TP53$_{(140-156)}$: TC(+57.02)PVQLWVDSTPPPGTR

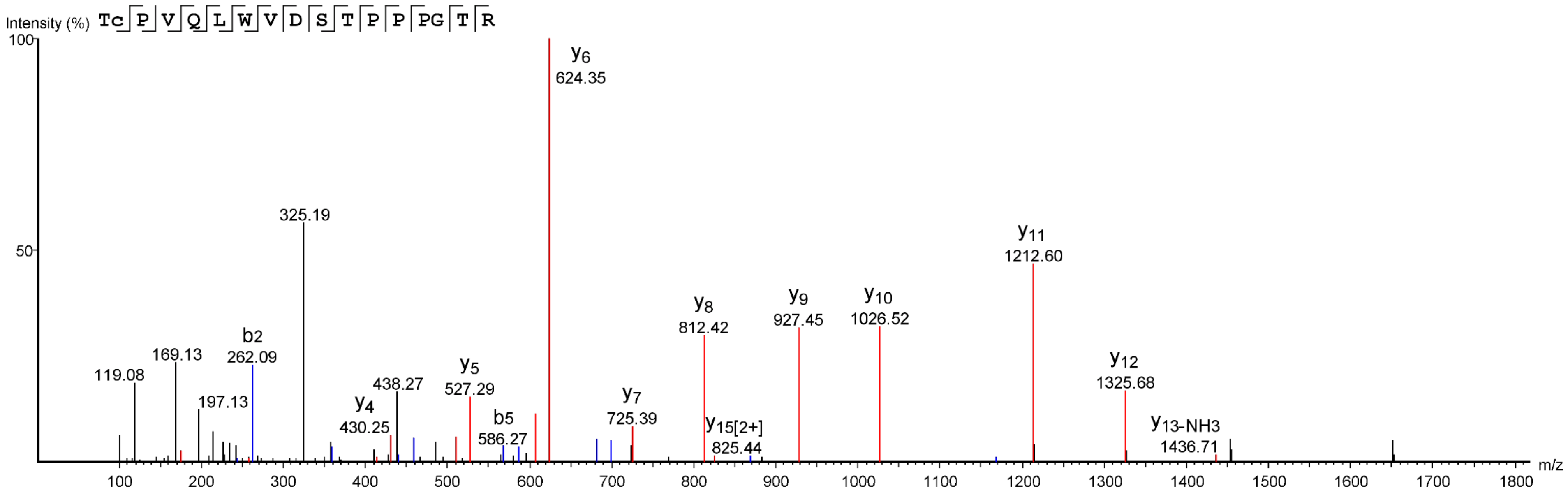

YMT31
Scan #15766

A-285: $TP53_{(140-156)}$: TC(+57.02)PVQLWVDSTPPPGTR

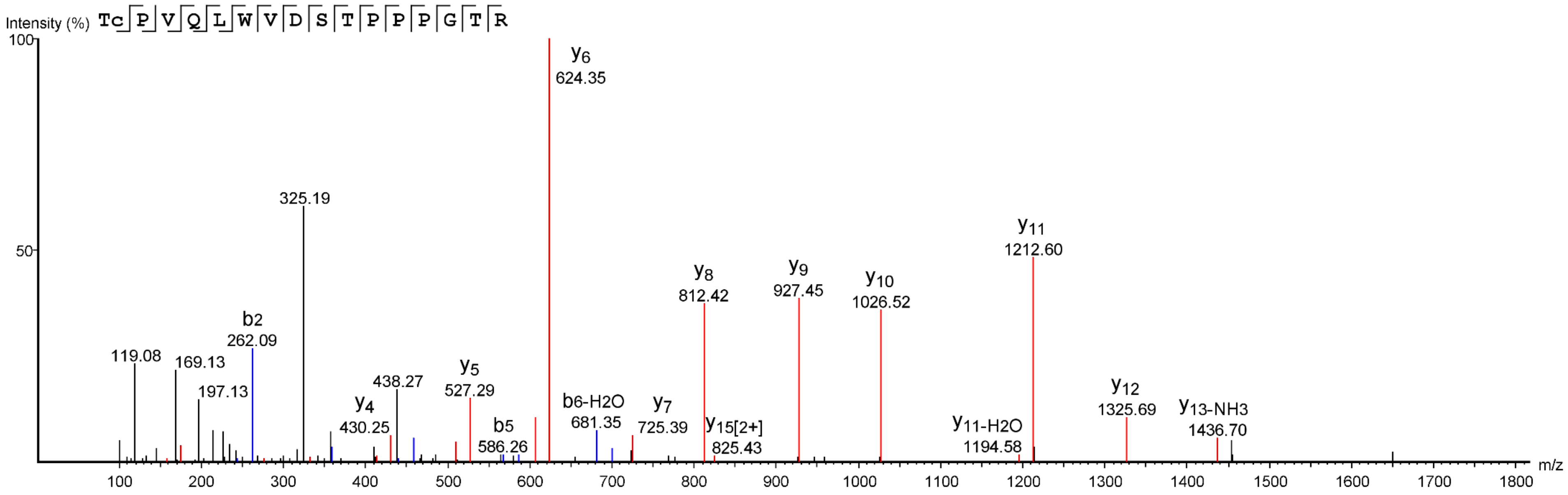

YMT32
Scan #16700

A-286: TRPA1$_{(391-403)}$: NLRPEFMQMQQIK+

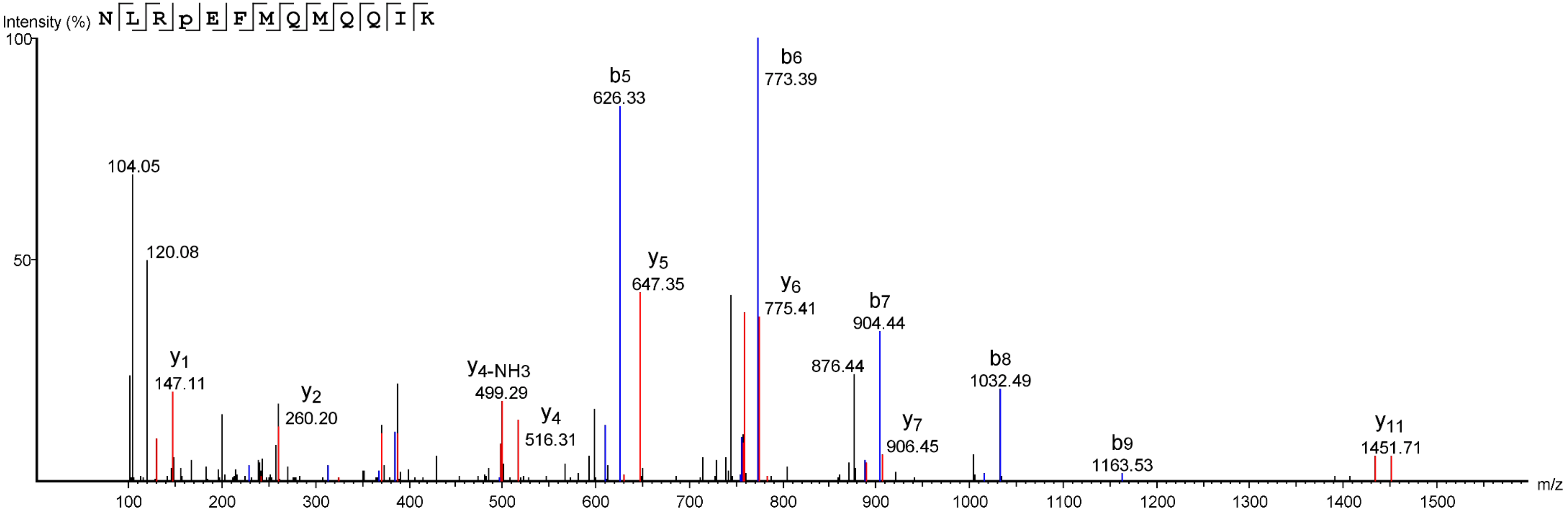

Pep11

Scan #11445

A-287: TRPA1$_{(391-403)}$: NLRP(+15.99)EFMQMQQIK+

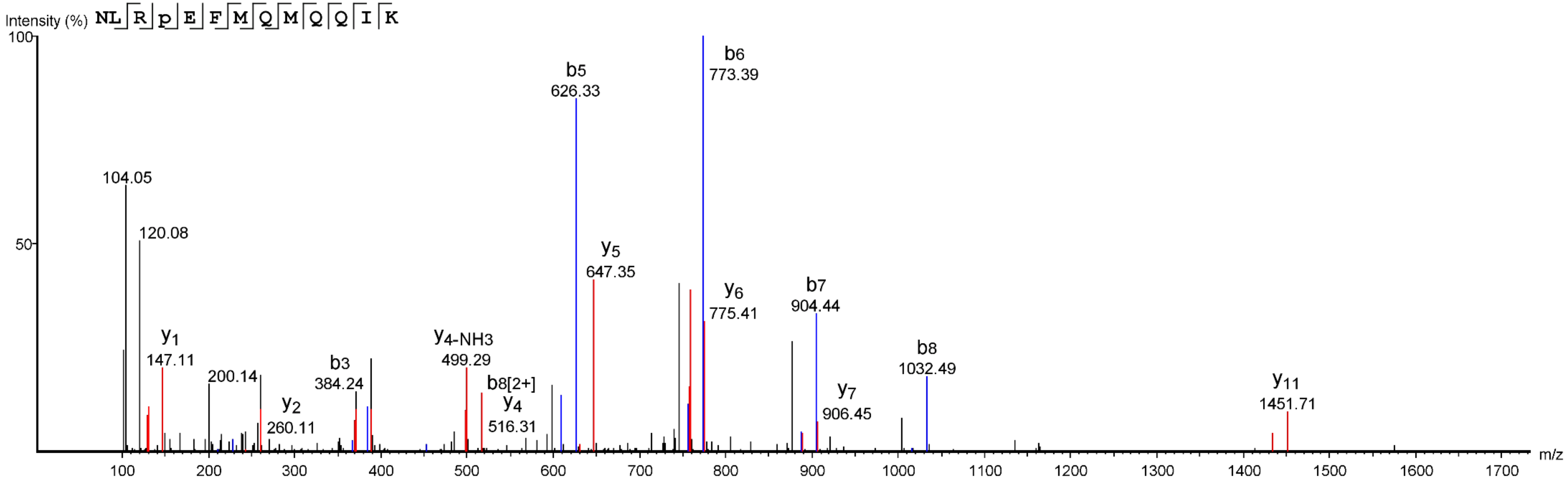

Pep11
Scan #10230

A-288: TRPA1$_{(391-403)}$: NLRPEFM(+15.99)QMQQIK+

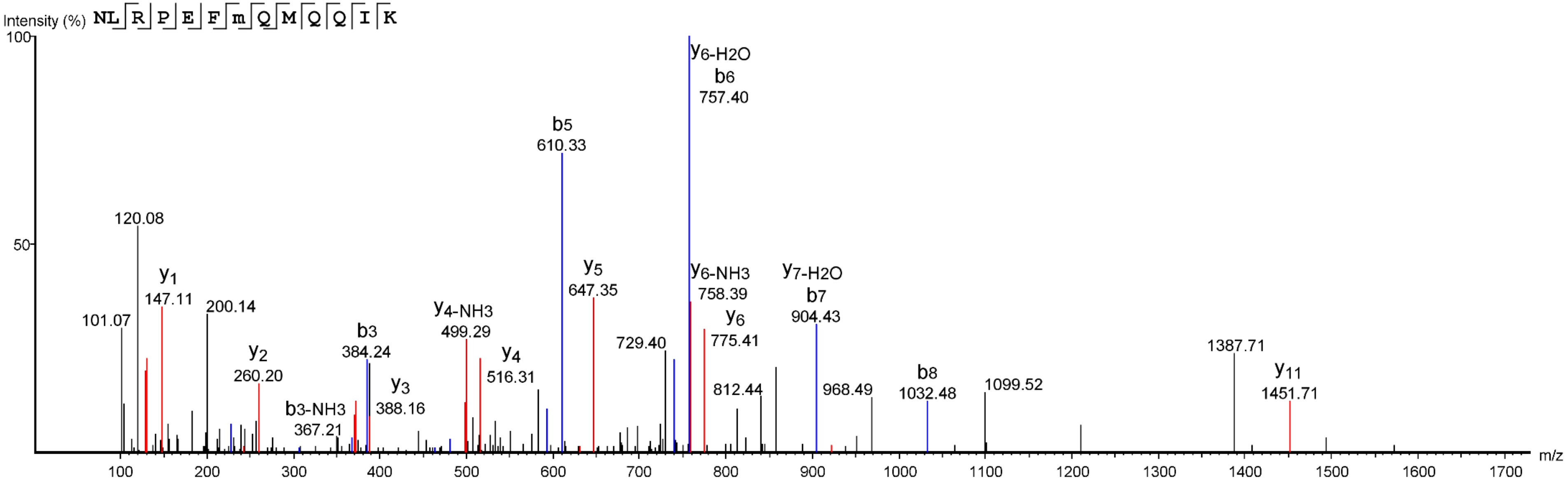

Pep11

Scan #9429

A-289: TRPA1(391-403): NLRPEFMQM(+15.99)QQIK+

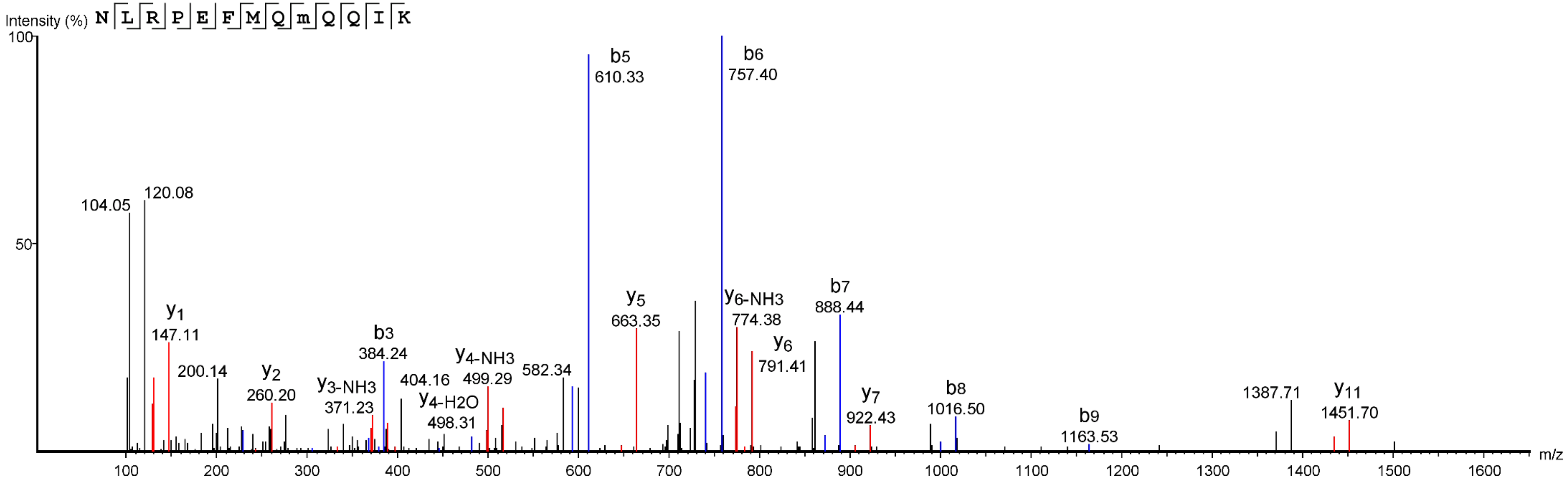

Pep11
Scan #9045

A-290: TRPA1$_{(391-403)}$: NLRP(+15.99)EFM(+15.99)QMQQIK+

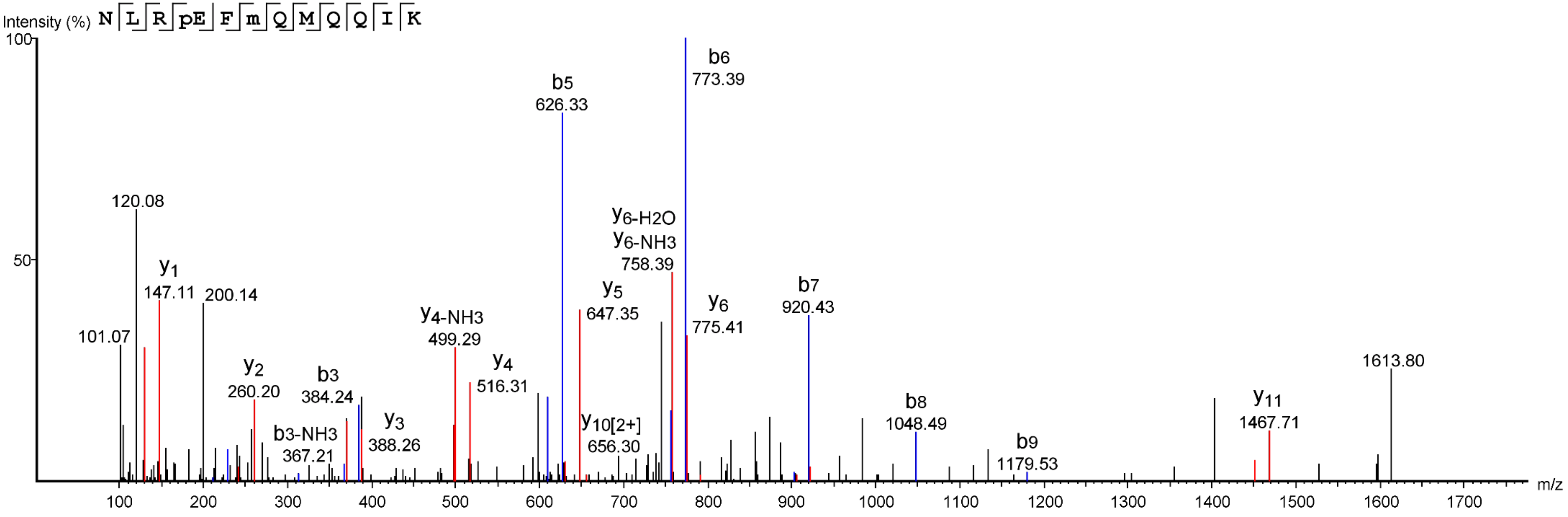

Pep11
Scan #8710

A-291: TRPA1$_{(391-403)}$: NLRP(+15.99)EFMQM(+15.99)QQIK+

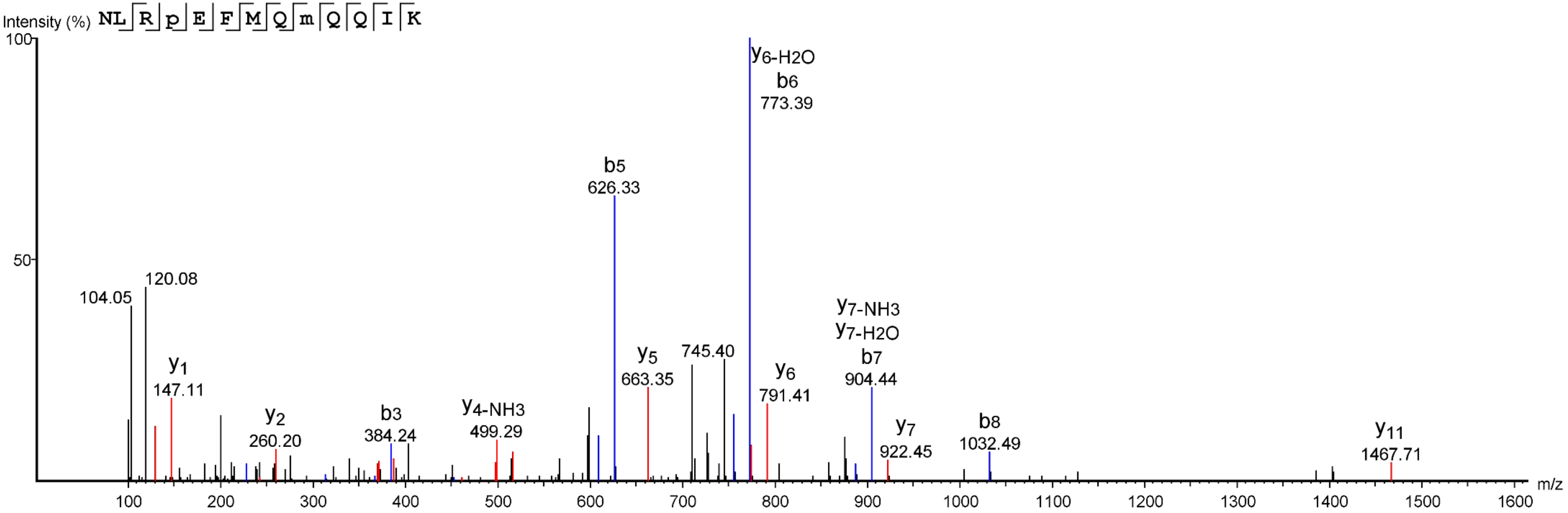

Pep11
Scan #7718

A-292: TRPA1$_{(391-403)}$: NLRPEFM(+15.99)QM(+15.99)QQIK+

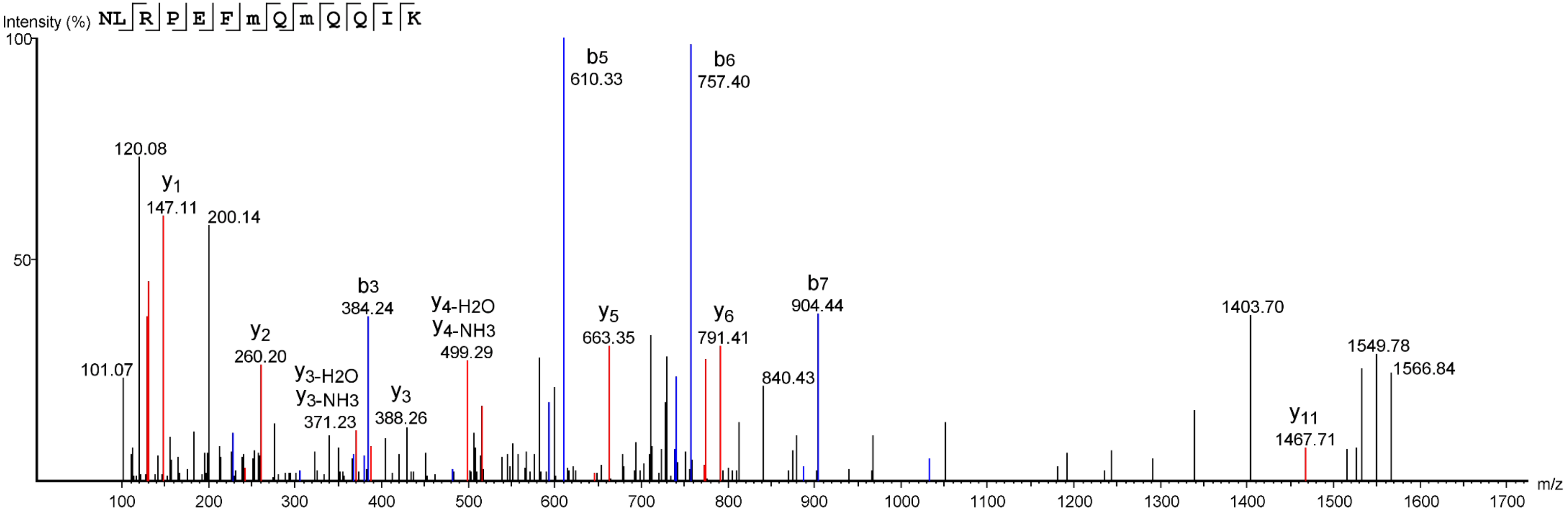

Pep11
Scan #7112

A-293: TRPA1$_{(391-403)}$: NLRP(+15.99)EFM(+15.99)QM(+15.99)QQIK+

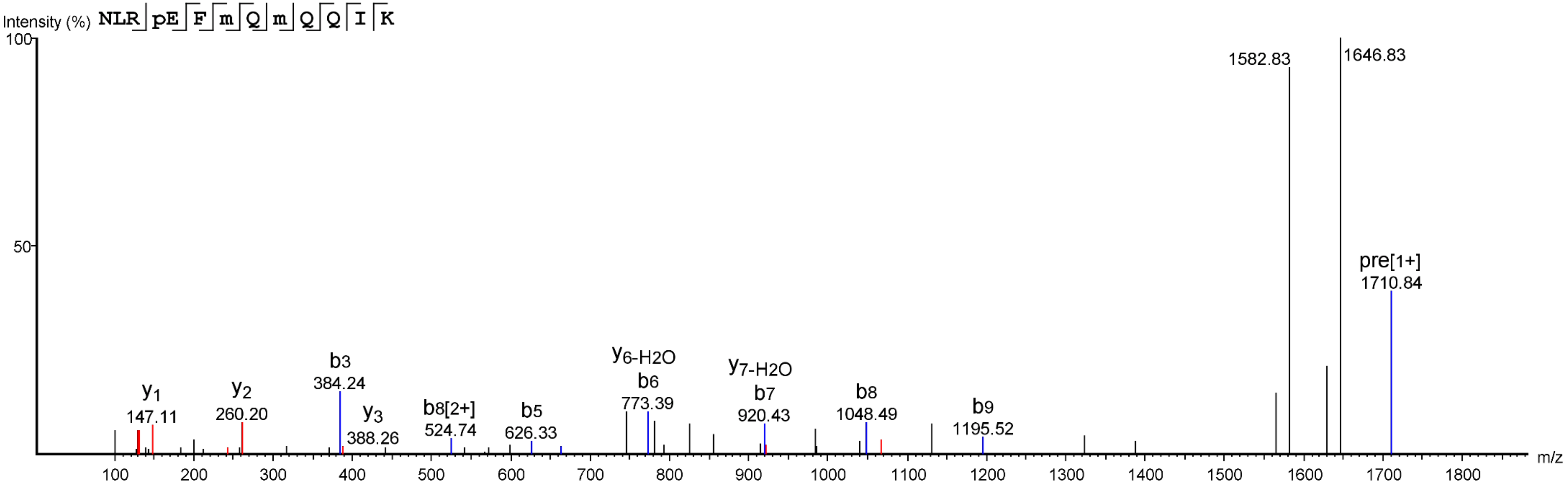

Pep11

Scan #6087

A-294: TRPA1$_{(391-403)}$: NLRPEFMQMQQIK+

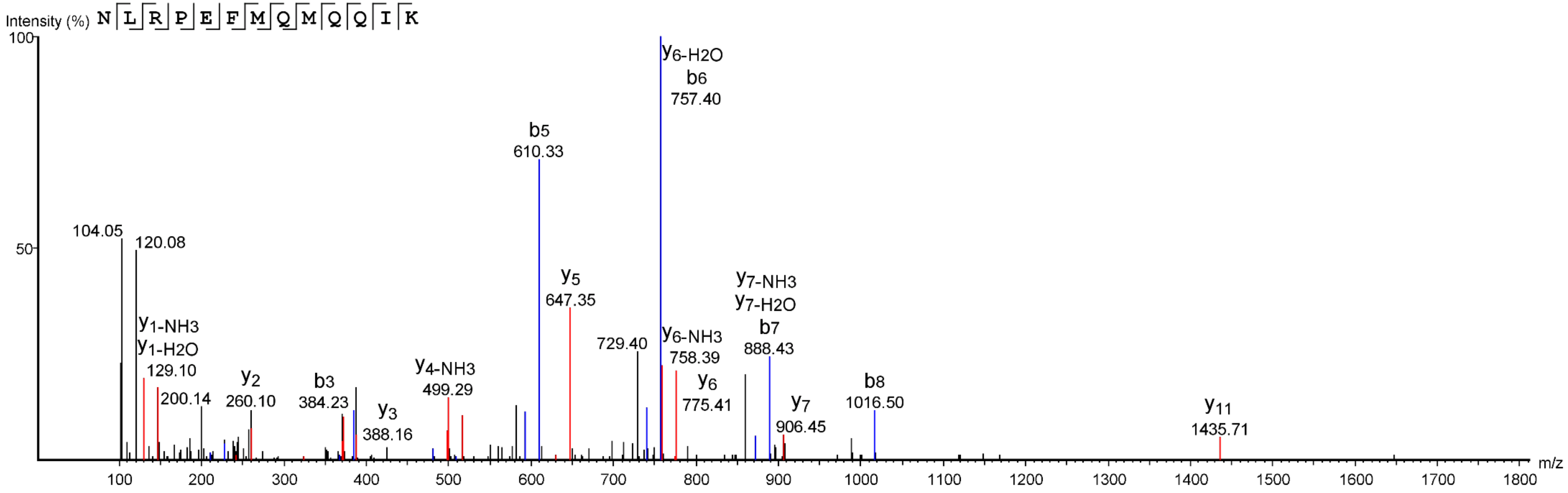

YMT71
Scan #11288

A-295: TRPA1$_{(391-403)}$: NLRPEFMQMQQIK+

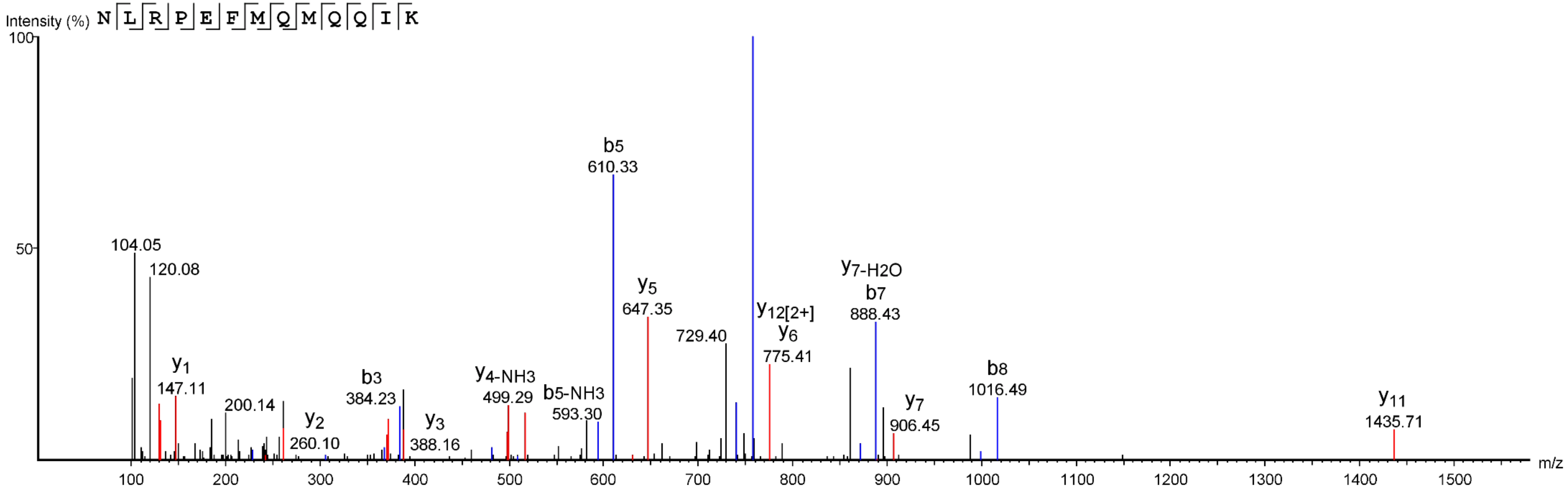

YMT72
Scan #11398

A-296: TRPA1$_{(391-403)}$: NLRPEFM(+15.99)QMQQIK+

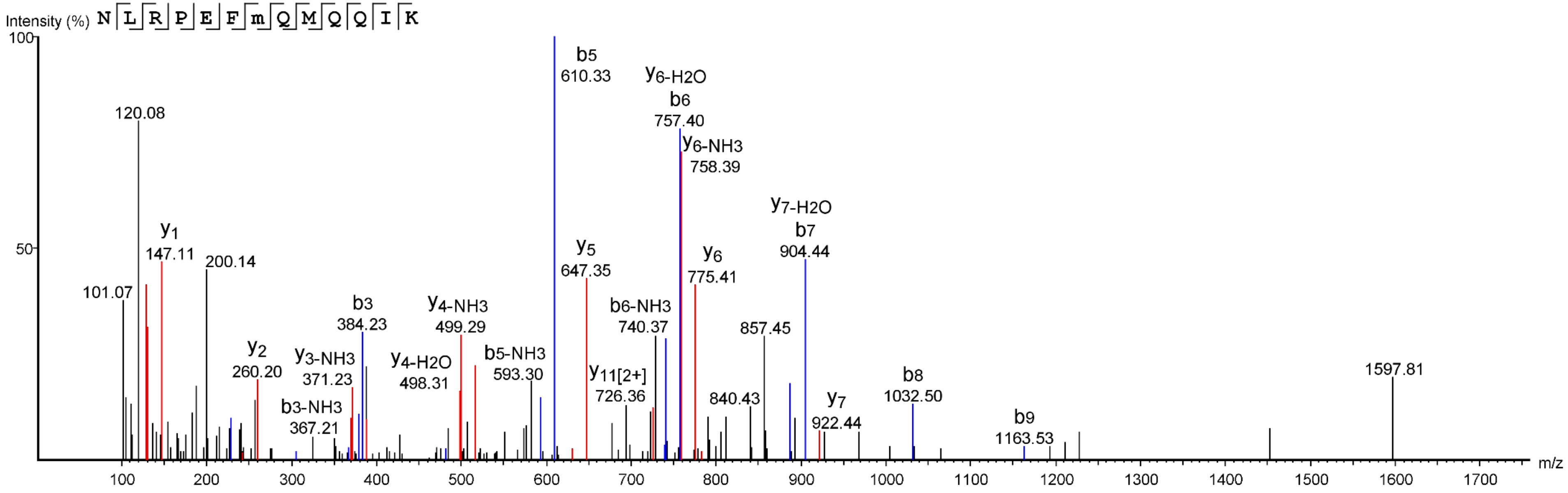

YMT71
Scan #9123

A-297: TRPA1$_{(391-403)}$: NLRPEFM(+15.99)QMQQIK+

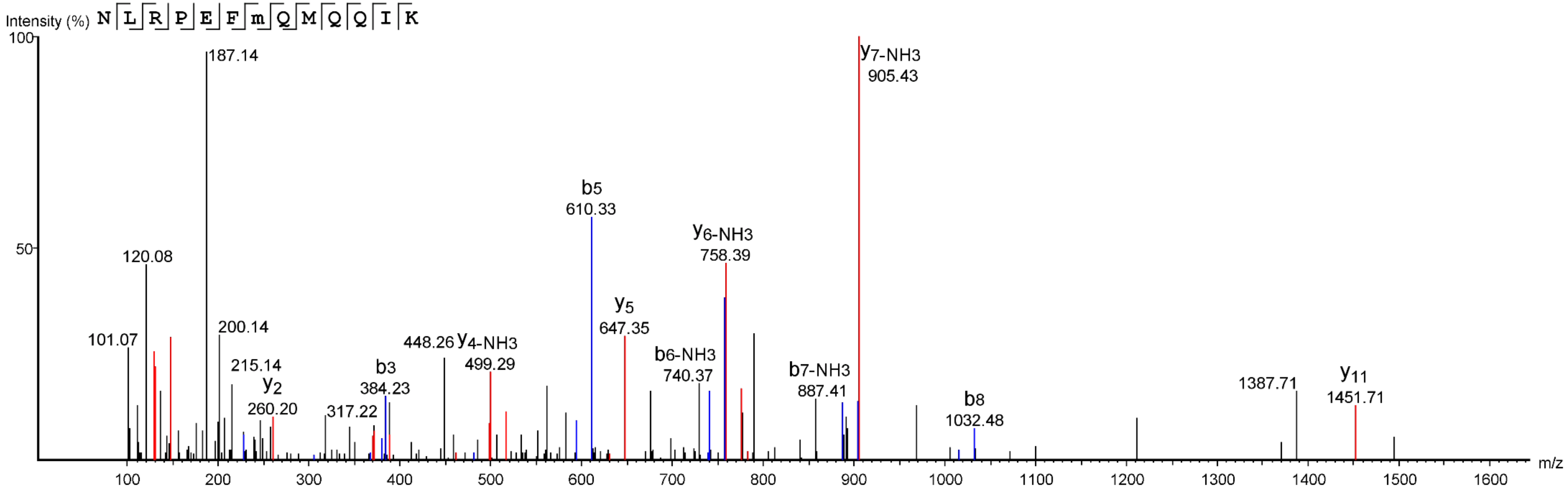

YMT72
Scan #9211

A-298: TRPA1$_{(391-403)}$: NLRPEFMQM(+15.99)QQIK+

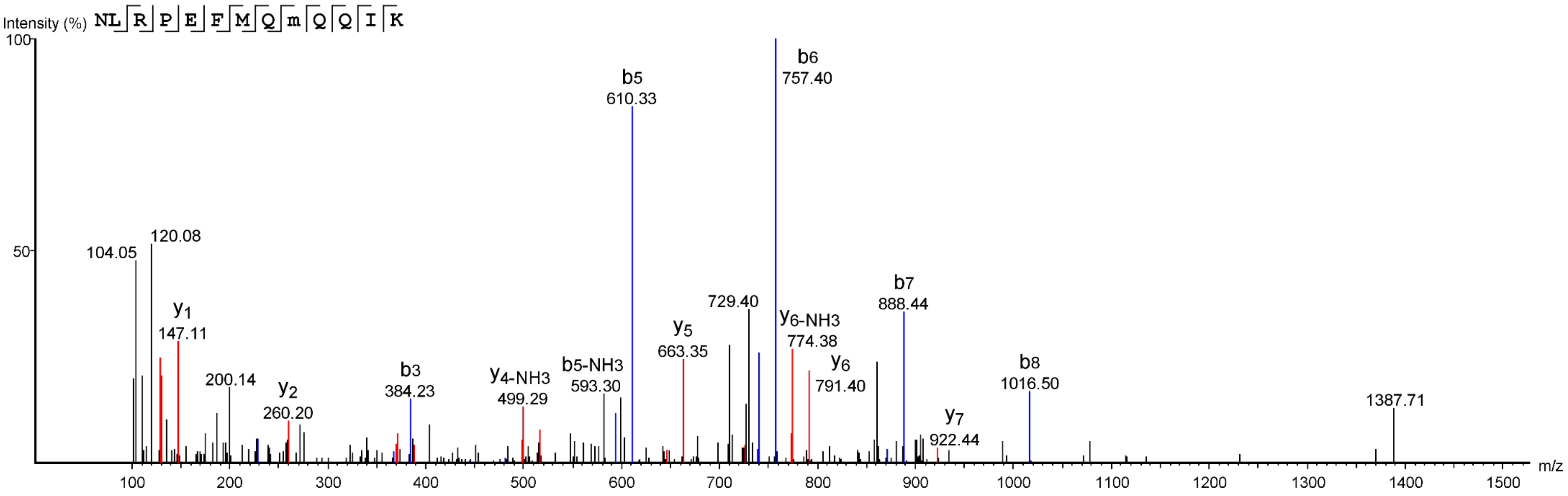

YMT71
Scan #8644

A-299: TRPA1$_{(391-403)}$: NLRPEFMQM(+15.99)QQIK

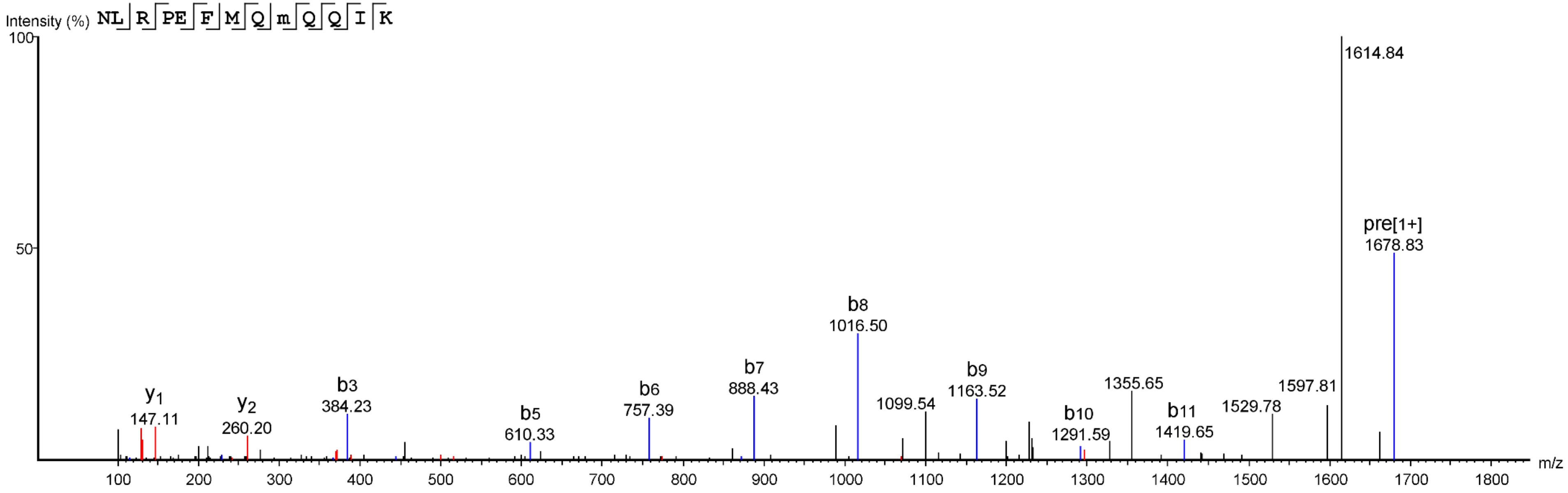

YMT72
Scan #8710

A-300: TP53$_{(358-370)}$: EPGGSRAHSSHLK

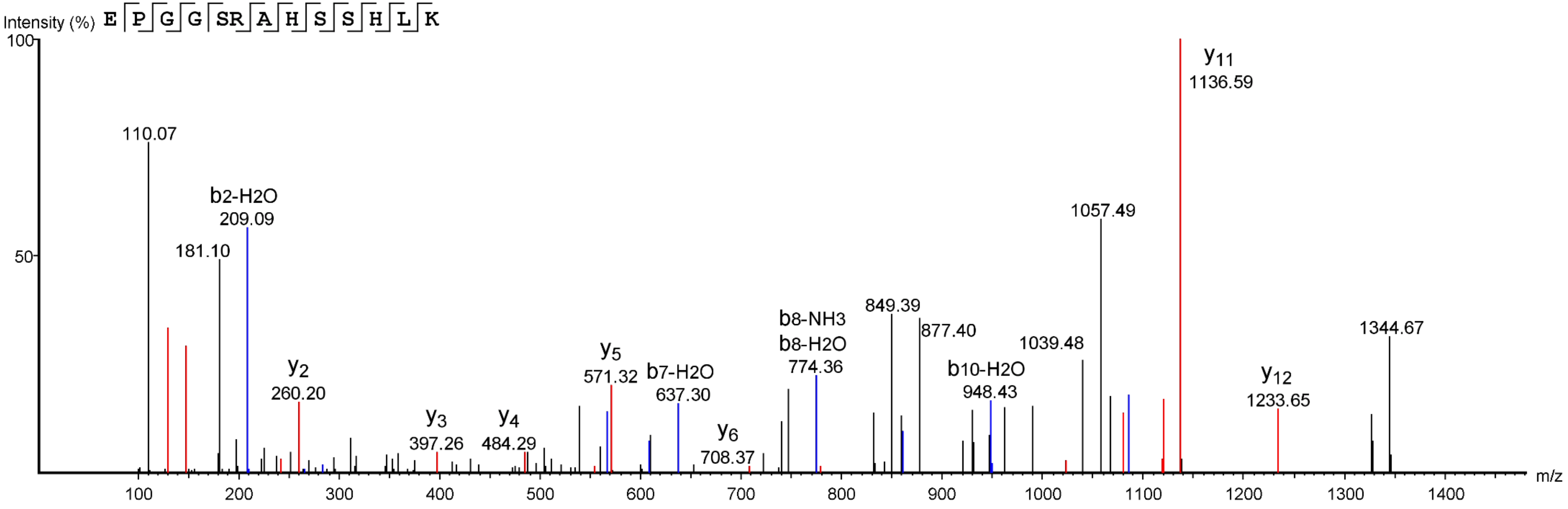

Pep13C
Scan #3194

A-301: TP53$_{(358-370)}$: EP(+15.99)GGSRAHSSHLK

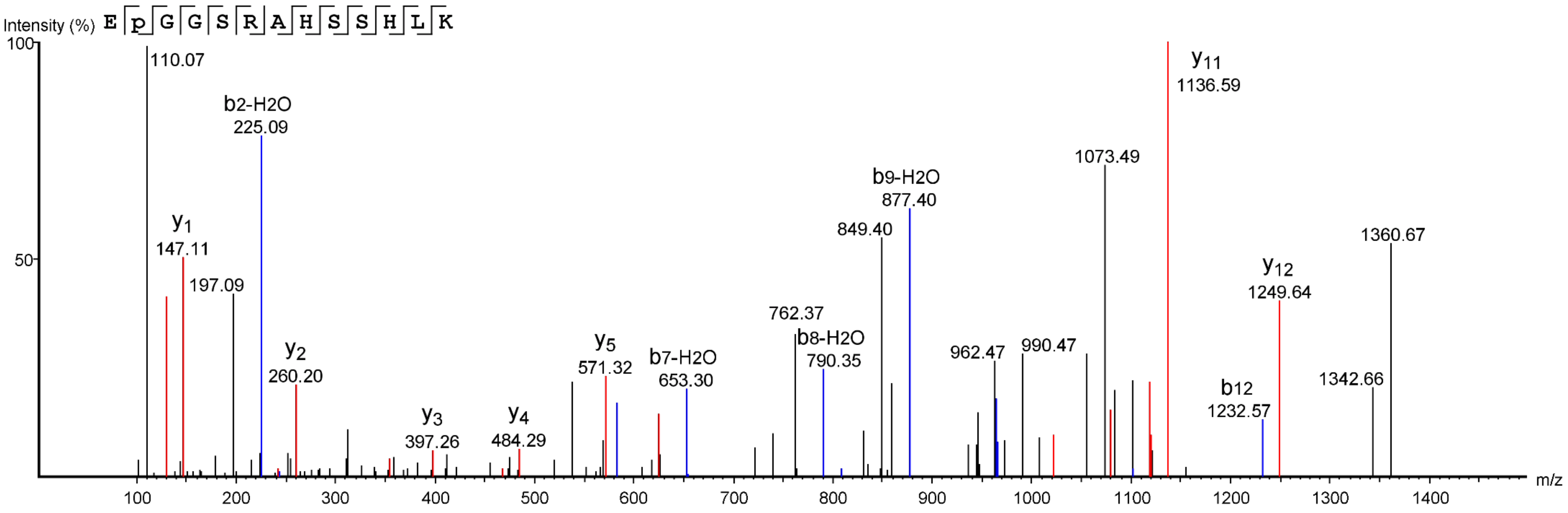

Pep13C
Scan #3186

A-302: TP53$_{(358-370)}$: EPGGSRAHSSHLK

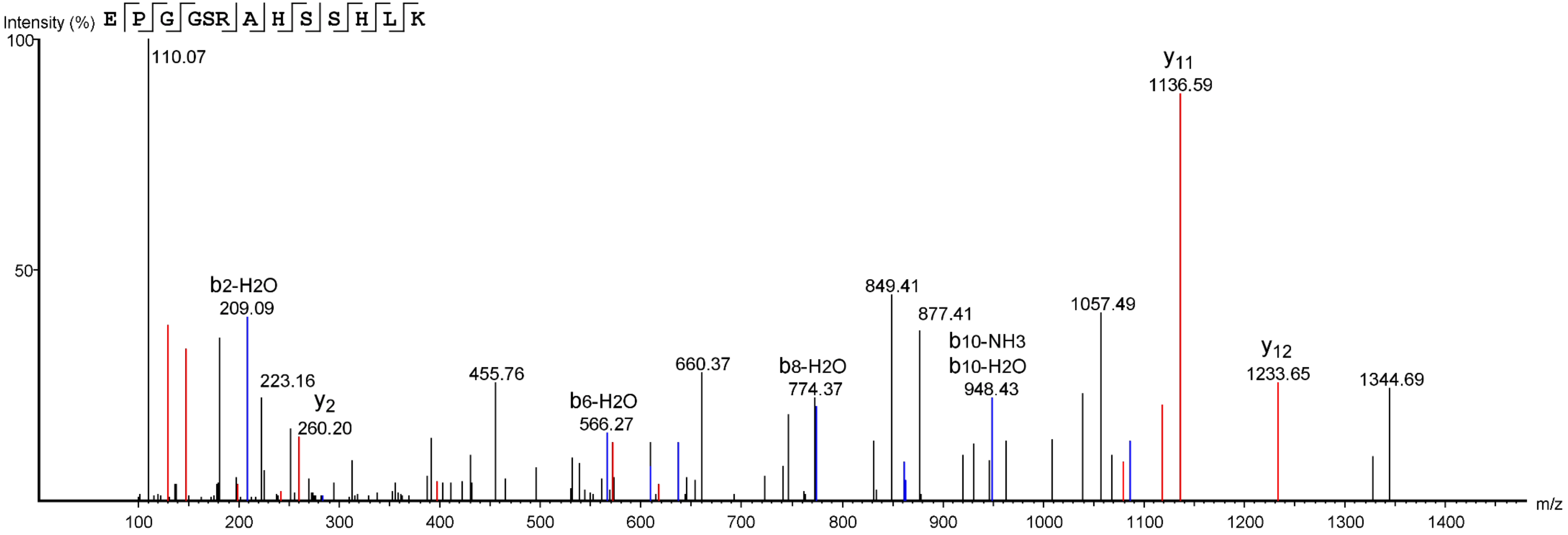

YMT113
Scan #3001

A-303: TP53$_{(358-370)}$: EPGGSRAHSSHLK

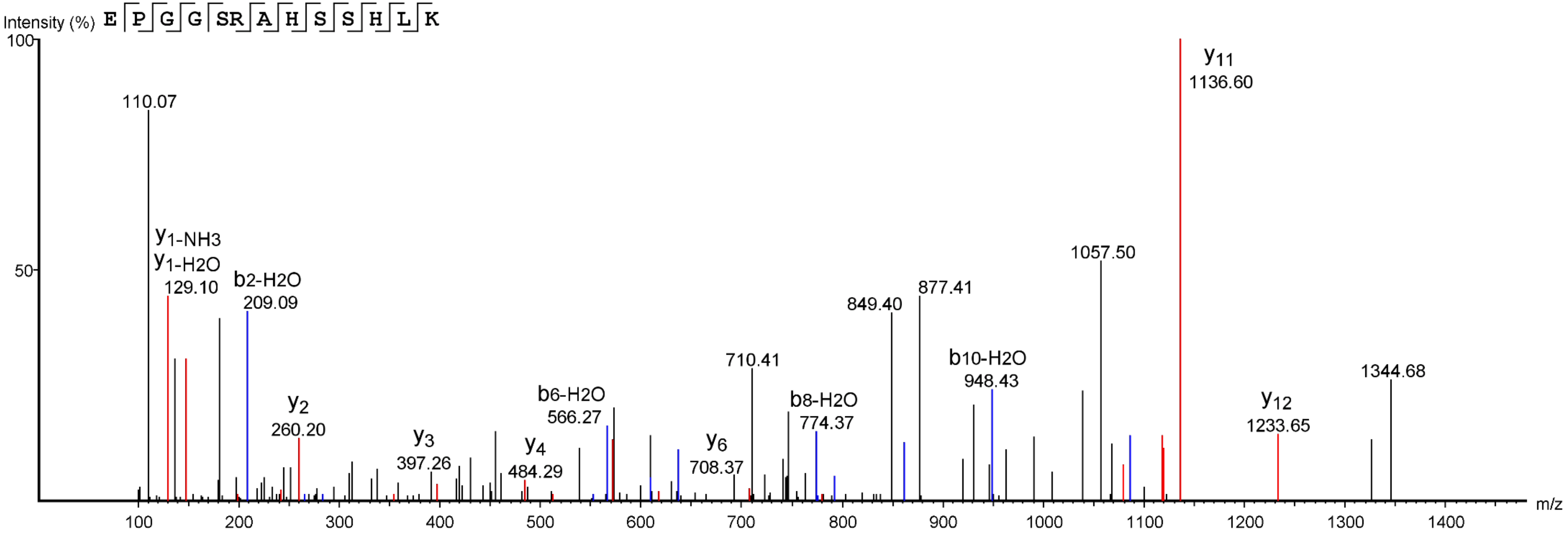

YMT114
Scan #3065

A-304: THRA$_{(153-176)}$: SLQQRPEPTPEEWDLIHIATEAHR

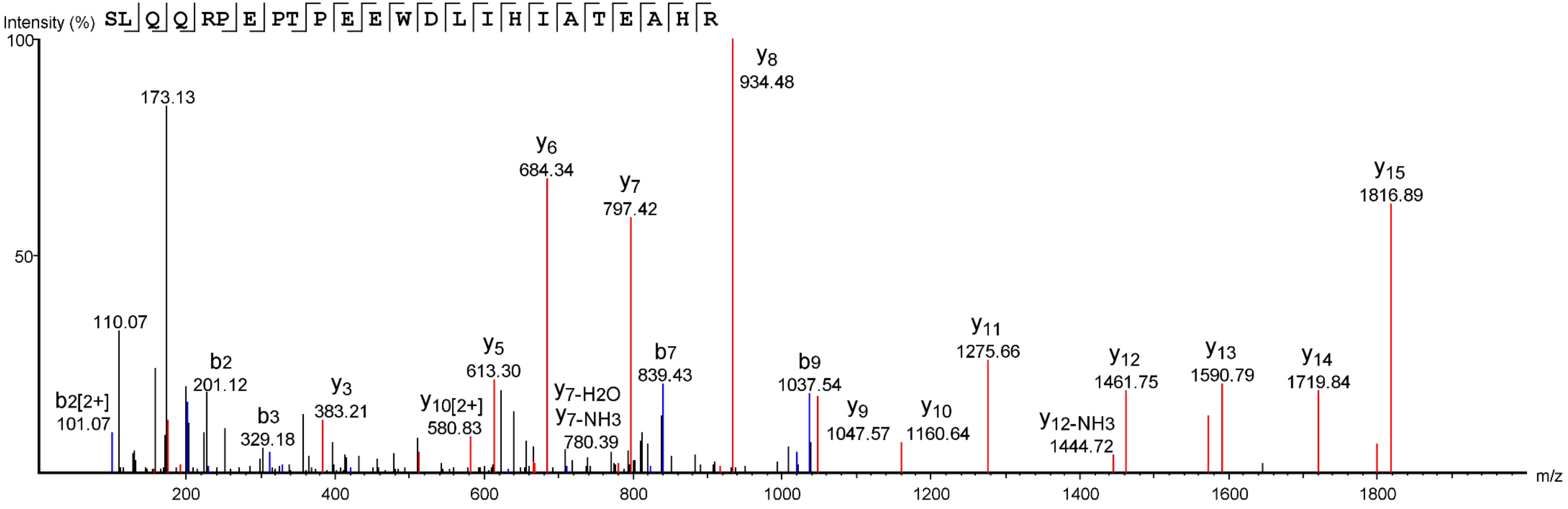

YMT93
Scan #15237

A-305: THRA$_{(153-176)}$: SLQQRPEPTPEEWDLIHIATEAHR

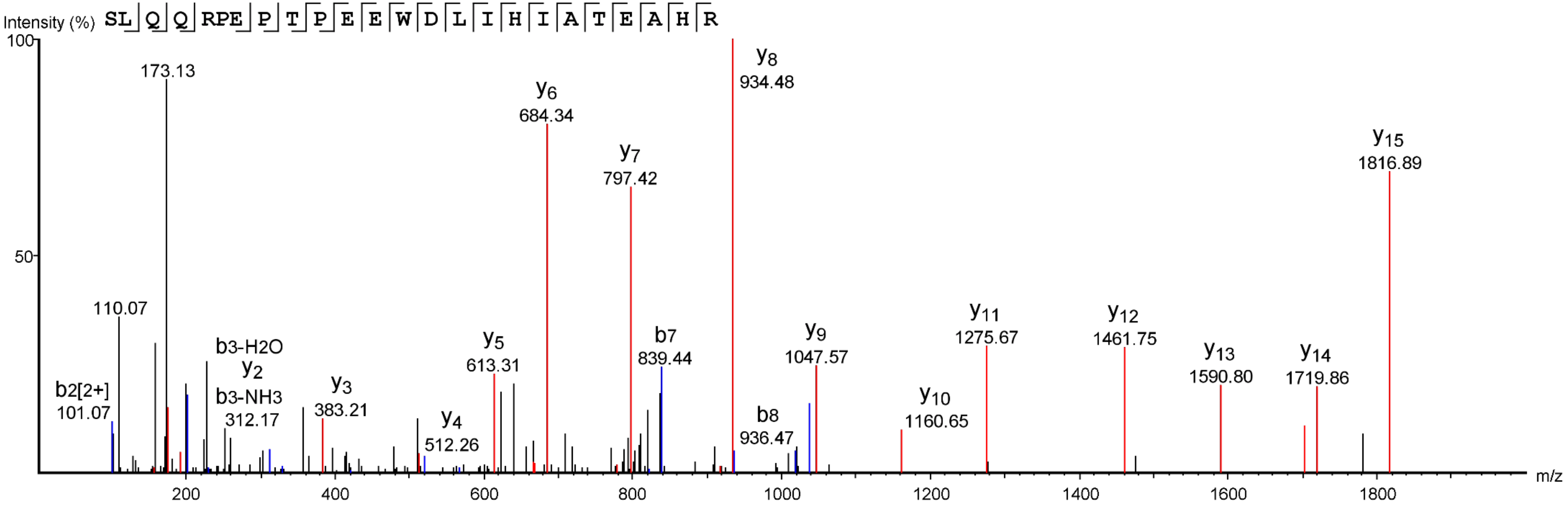

YMT94
Scan #15235

A-306: THRA$_{(153-176)}$: SLQQRPEPTPEEWD(+15.99)LIHIATEAHR

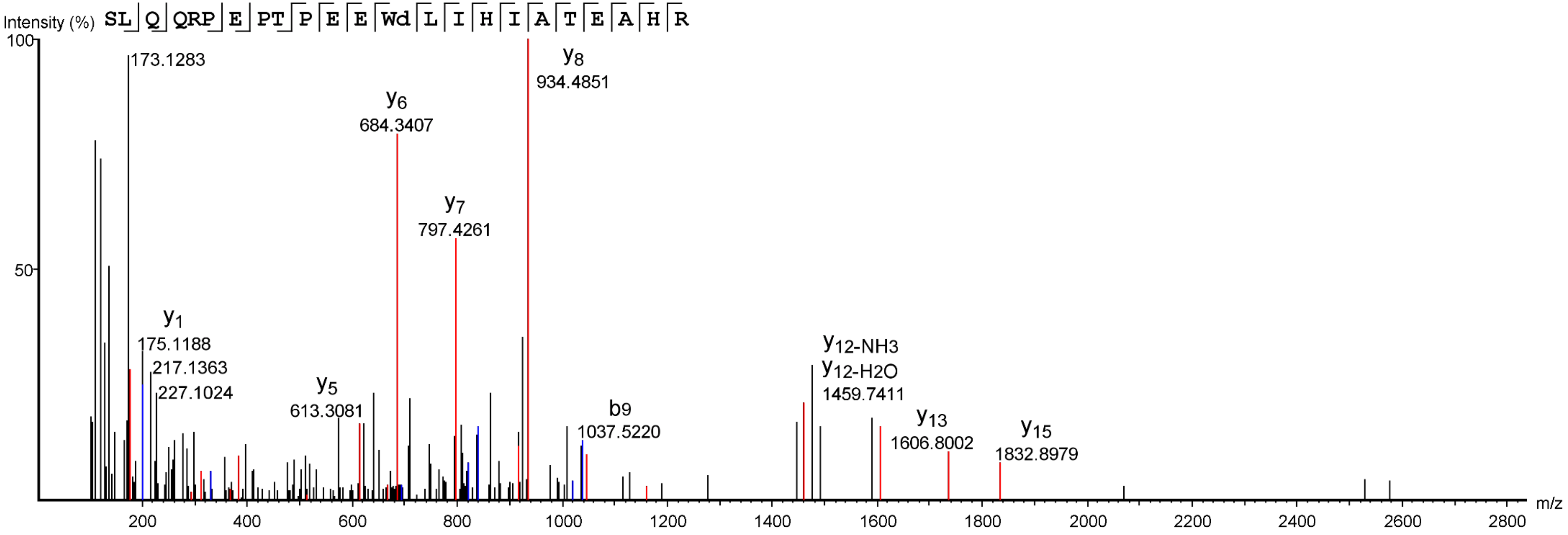

YMT93
Scan #11212

A-307: THRA$_{(153-176)}$: SLQQRPEPTP(+15.99)EEWDLIHIATEAHR

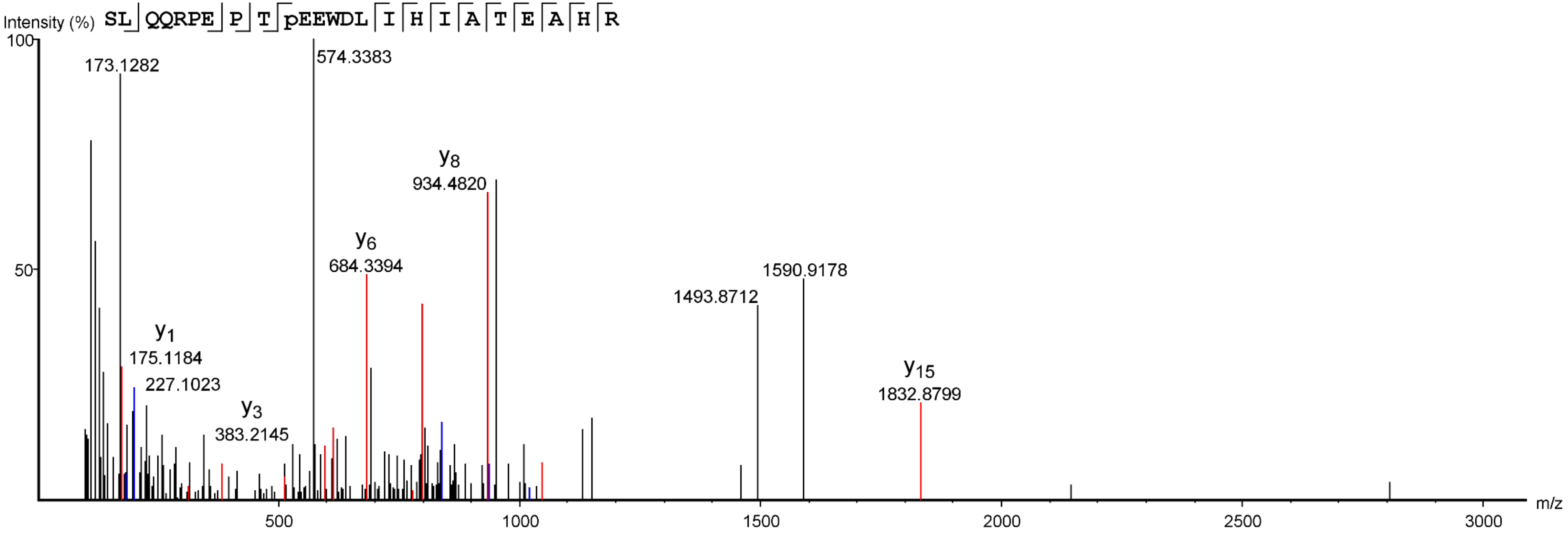

YMT94
Scan #11325

A-308: THRA$_{(153-176)}$: SLQQRPEPTPEEW(+31.99)DLIHIATEAHR

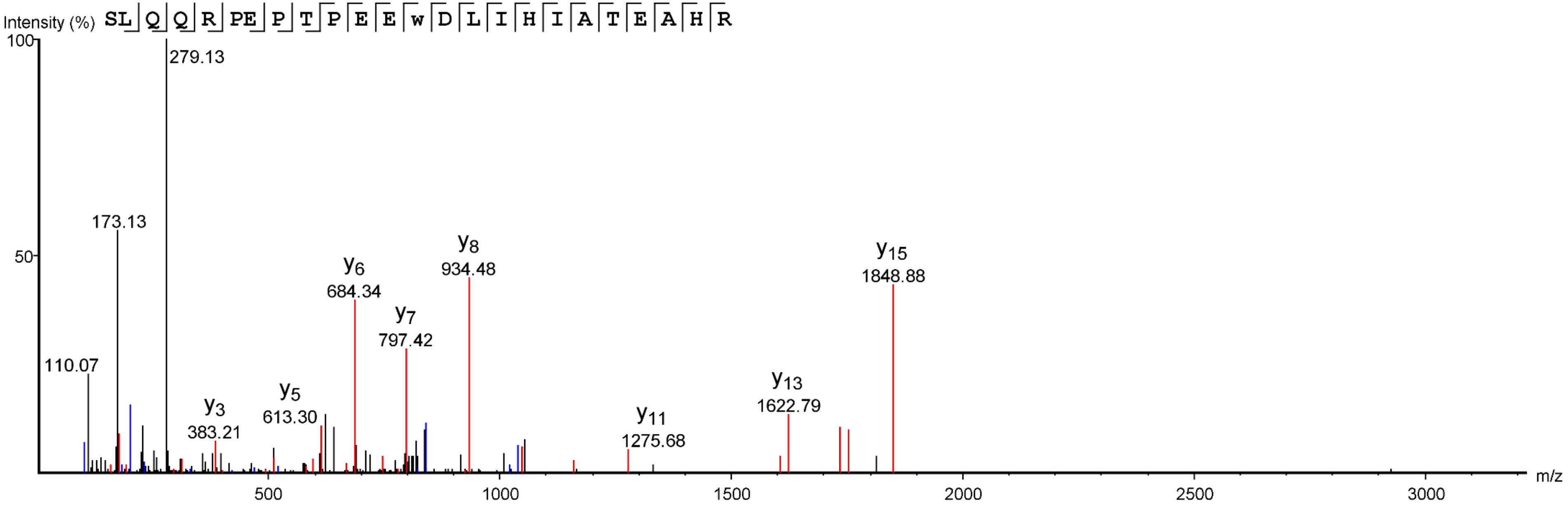

YMT93
Scan #13739

A-309: THRA(153-176): SLQQRPEPTPEEW(+31.99)DLIHIATEAHR

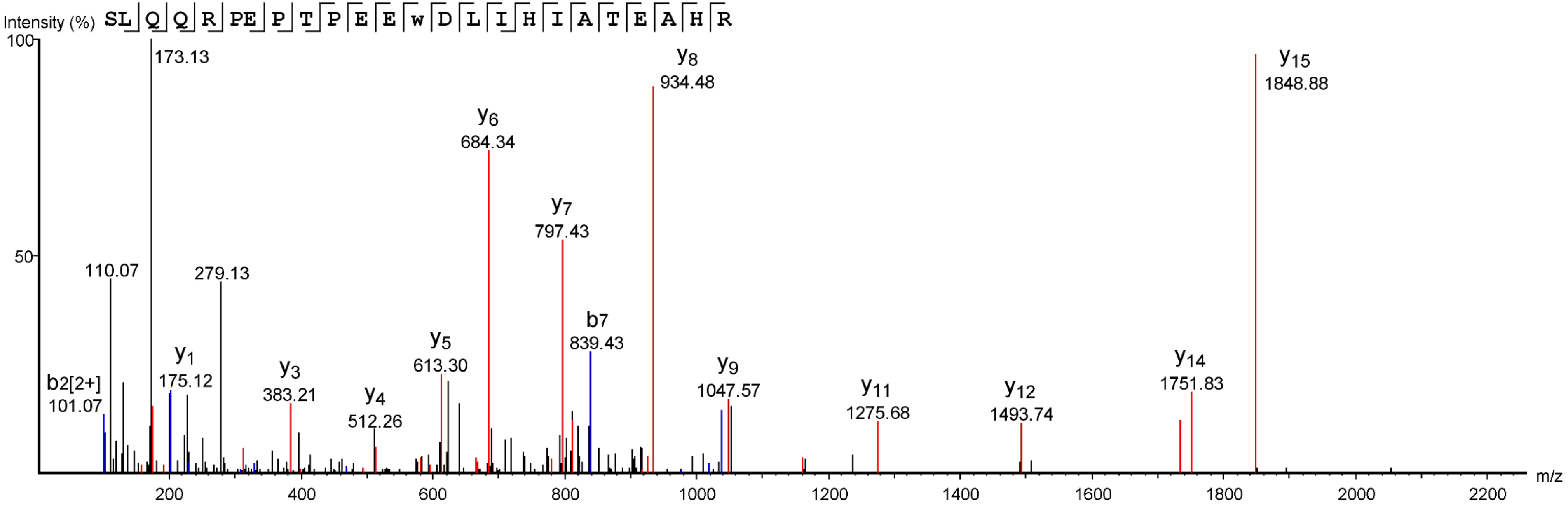

YMT94
Scan #13614

A-310: $THRA_{(153-176)}$: SLQQRPEPTPEEWDLIHIATEAHR

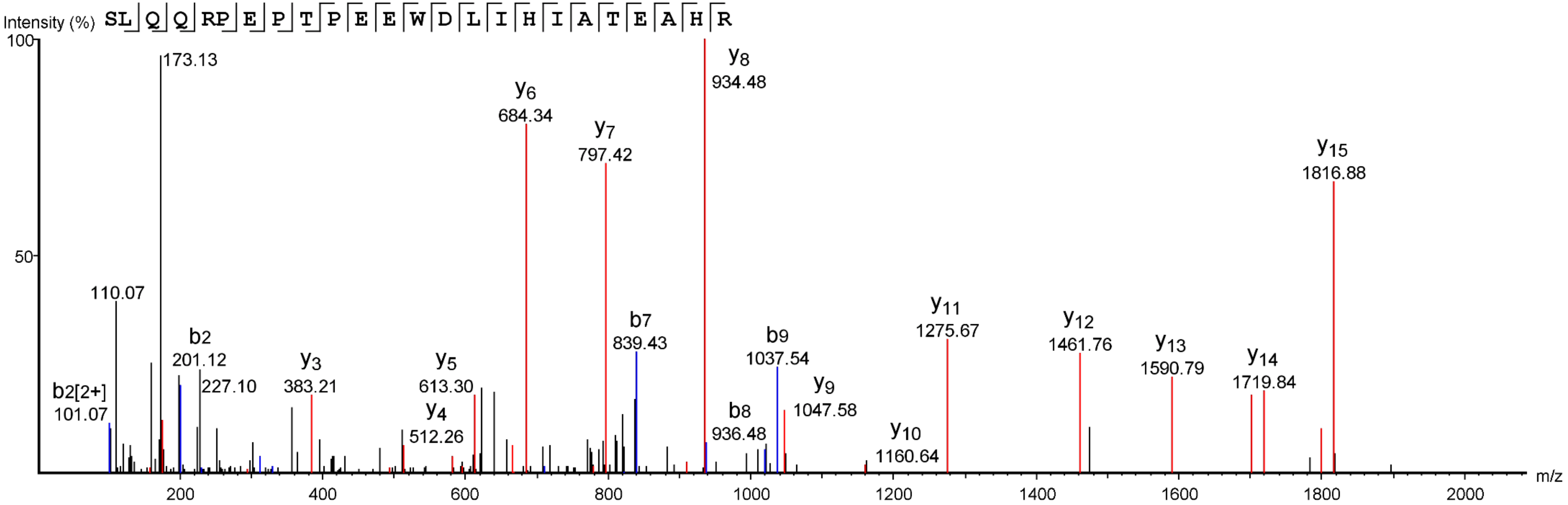

YMT95
Scan #15703

A-311: THRA(153-176): SLQQRPEPTPEEW(+31.99)DLIHIATEAHR

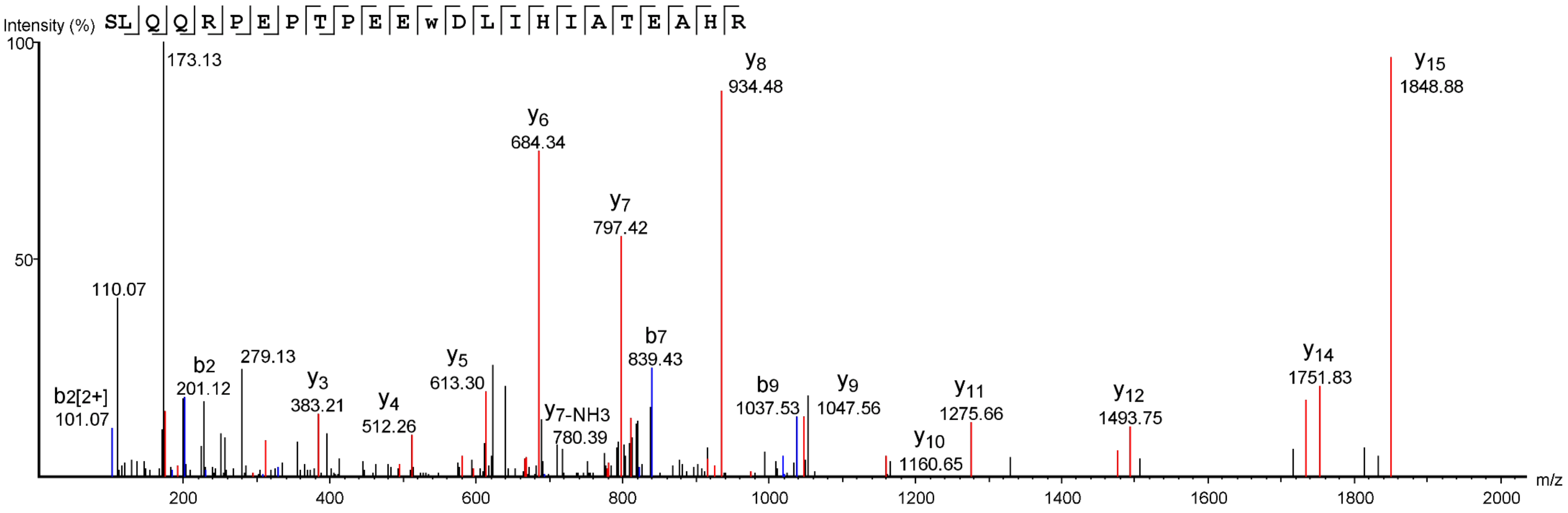

YMT95
Scan #13852

A-312: EPOR$_{(426-453)}$: YTILDPSSQLLRPWTLC(+57.02)PELPPTPPHLK

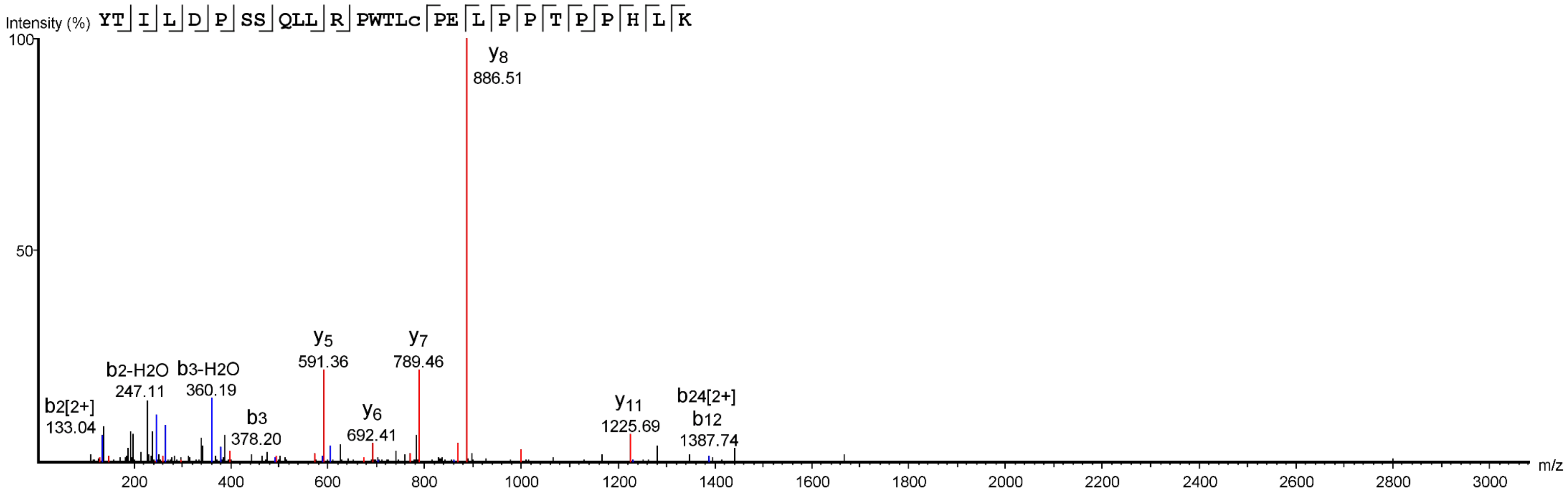

YMT63

Scan #18425

A-313: EPOR(426-453): YTILDPSSQLLRPWTLC(+57.02)PELPPTPPHLK

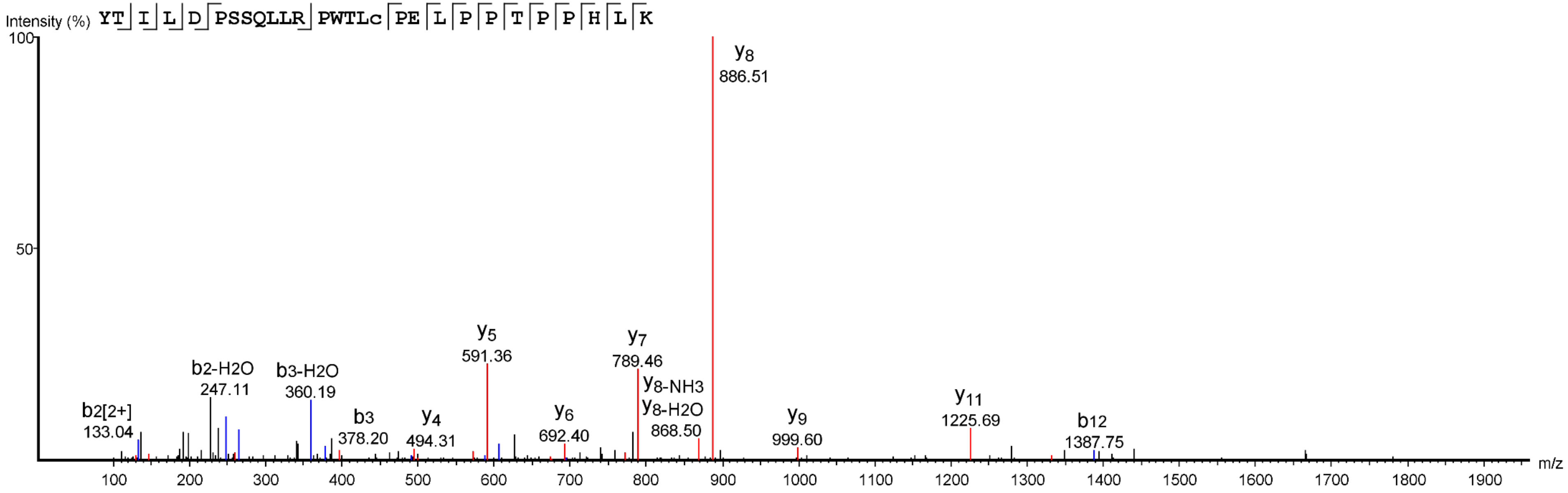

YMT64
Scan #18188

A-314: EPOR$_{(426-453)}$: YTILDPSSQLLRP(+15.99)WTLC(+57.02)PELPPTPPHLK

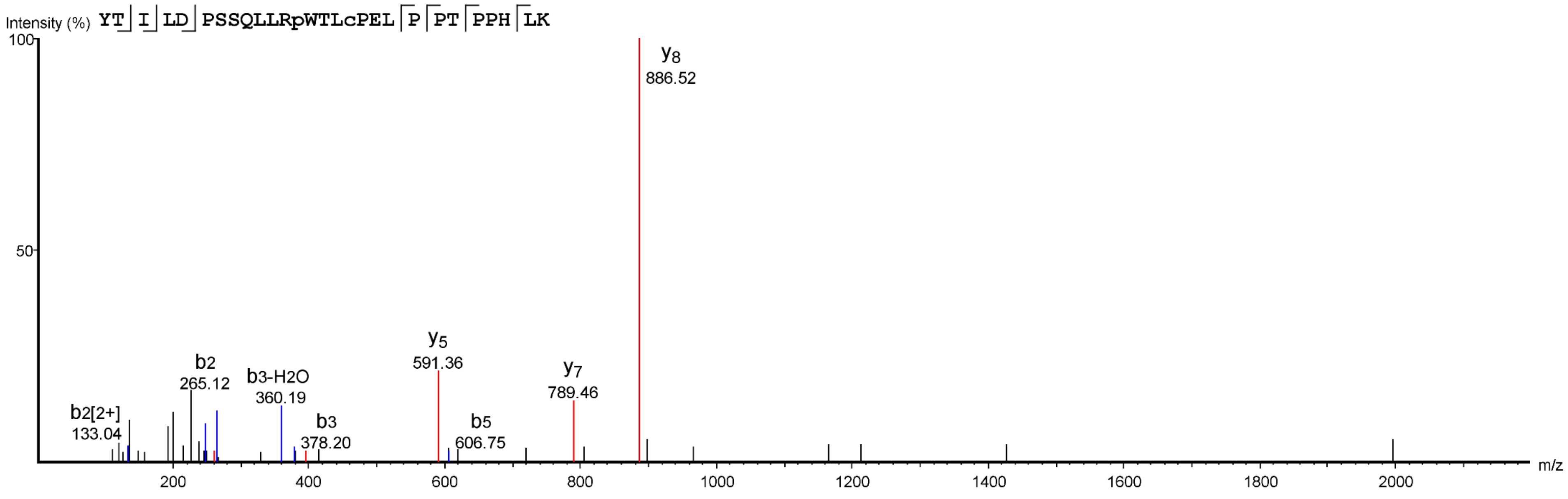

YMT63

Scan #17329

A-315: EPOR(426-453): YTILDPSSQLLRPW(+31.99)TLC(+57.02)PELPPTPPHLK

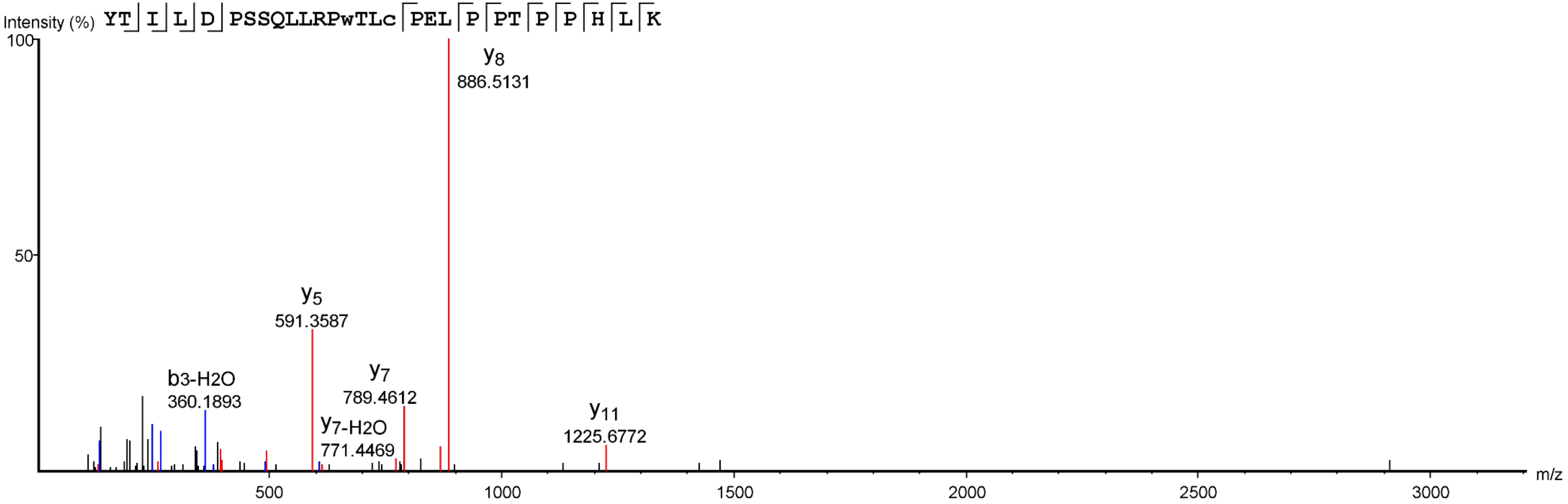

YMT63
Scan #17308

A-316: EPOR(426-453): YTILDPSSQLLRPW(+31.99)TLC(+57.02)PELPPTPPHLK

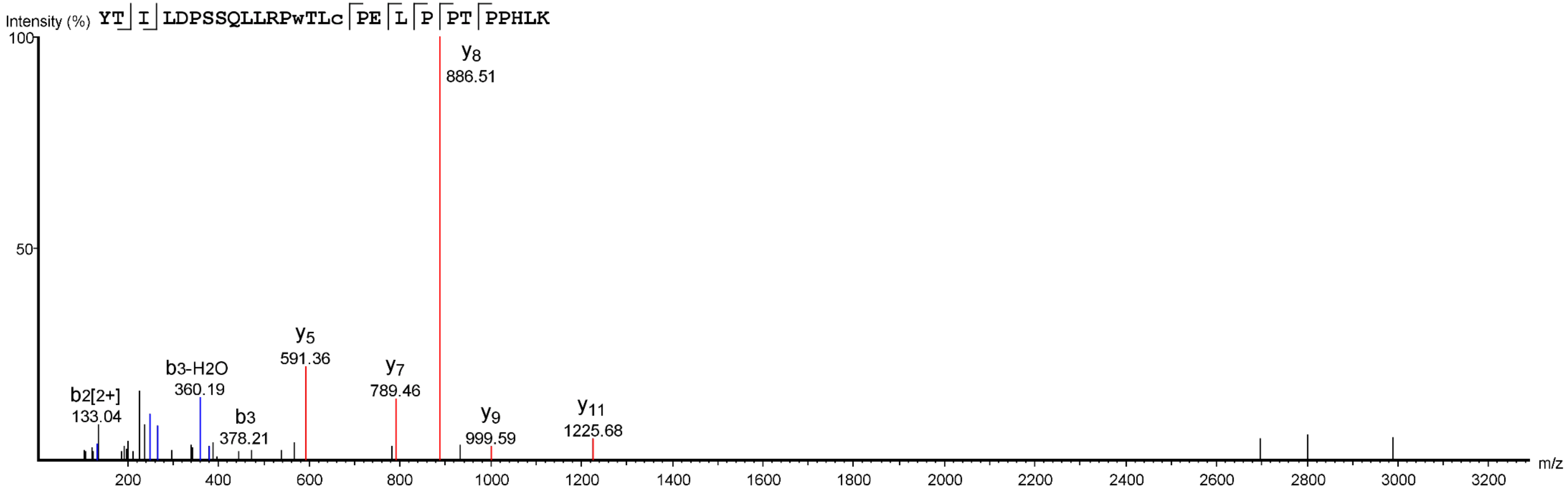

YMT64
Scan #17367

A-317: HIF-1α*(548-575): NPFSTQDTDLDLEALAPYIPADDDFQLR

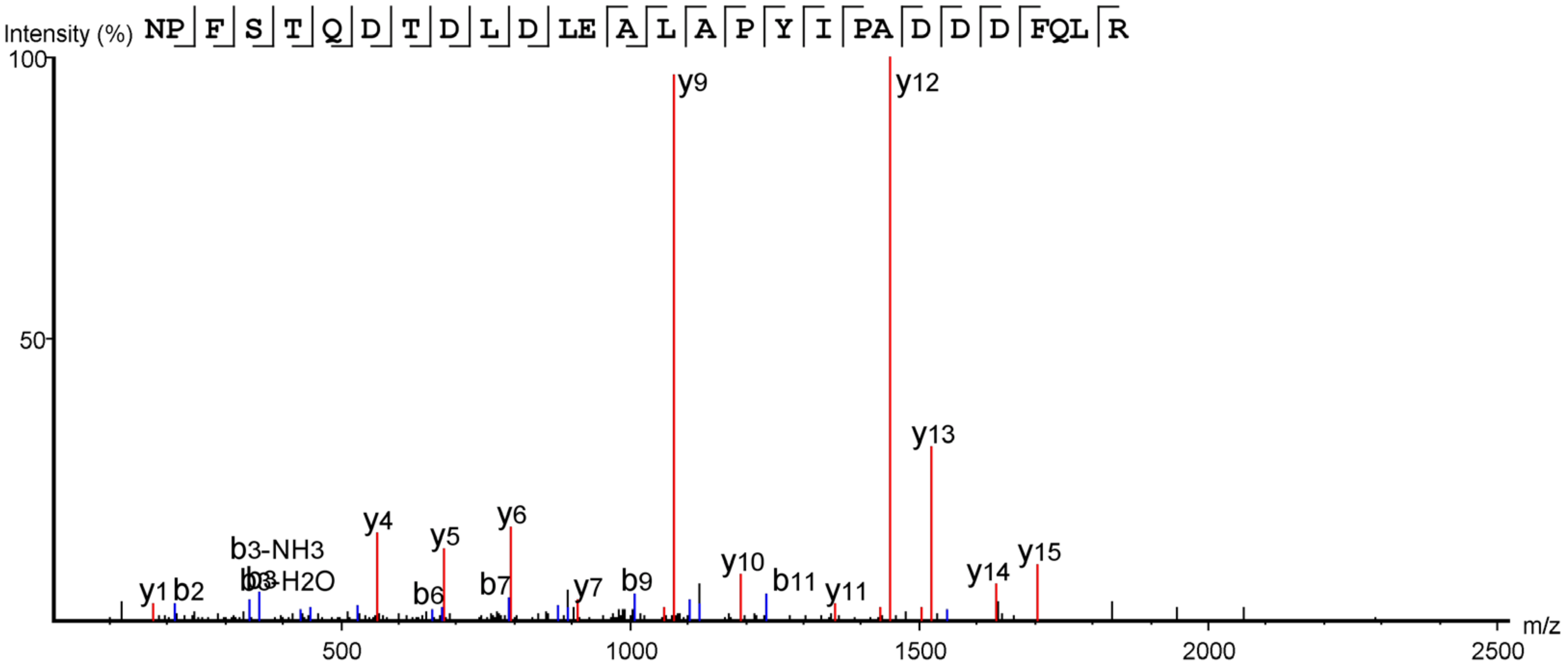

YMT115
Scan #25893

A-318: HIF-1α*$_{(548-575)}$: NPFSTQDTDLDLEALAPYIPADDDFQLR

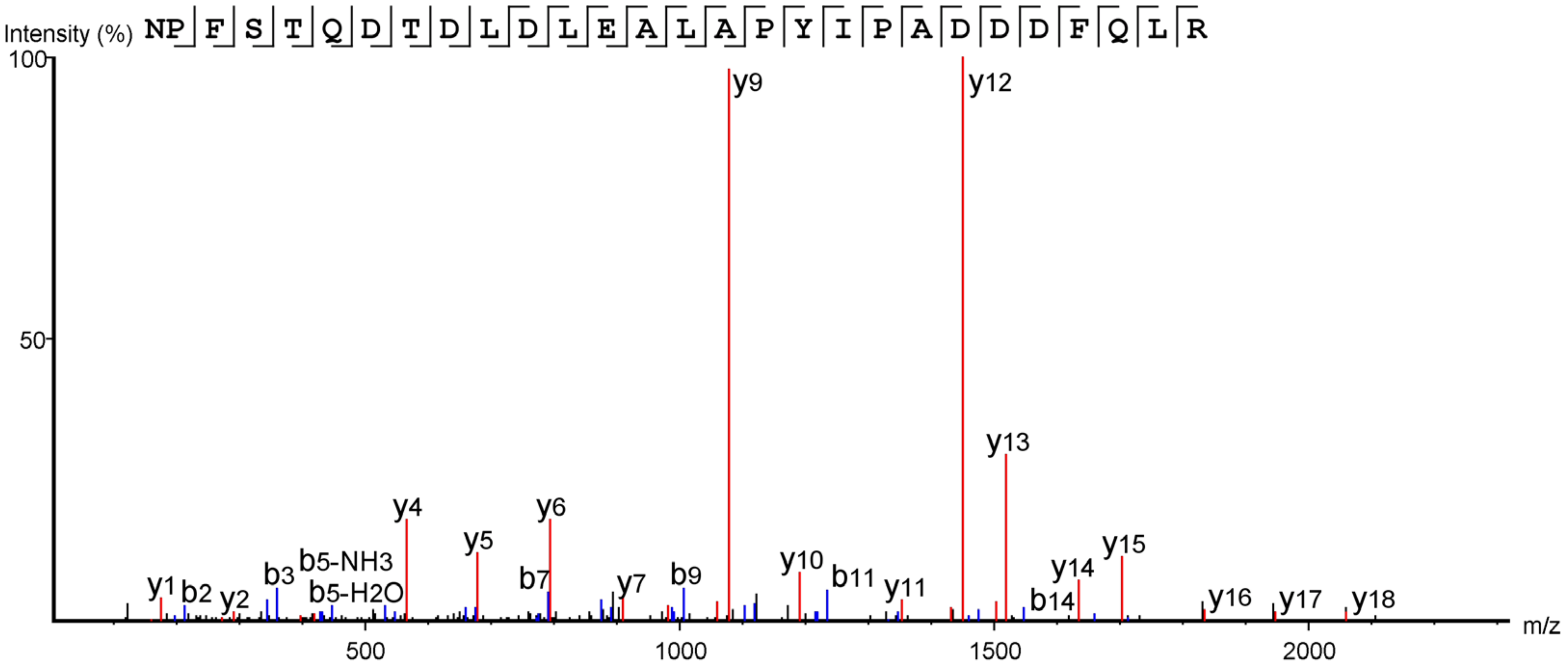

YMT116
Scan #25647

A-319: HIF-1α*(548-575): NPFSTQDTDLDLEALAPYIPADDDFQLR

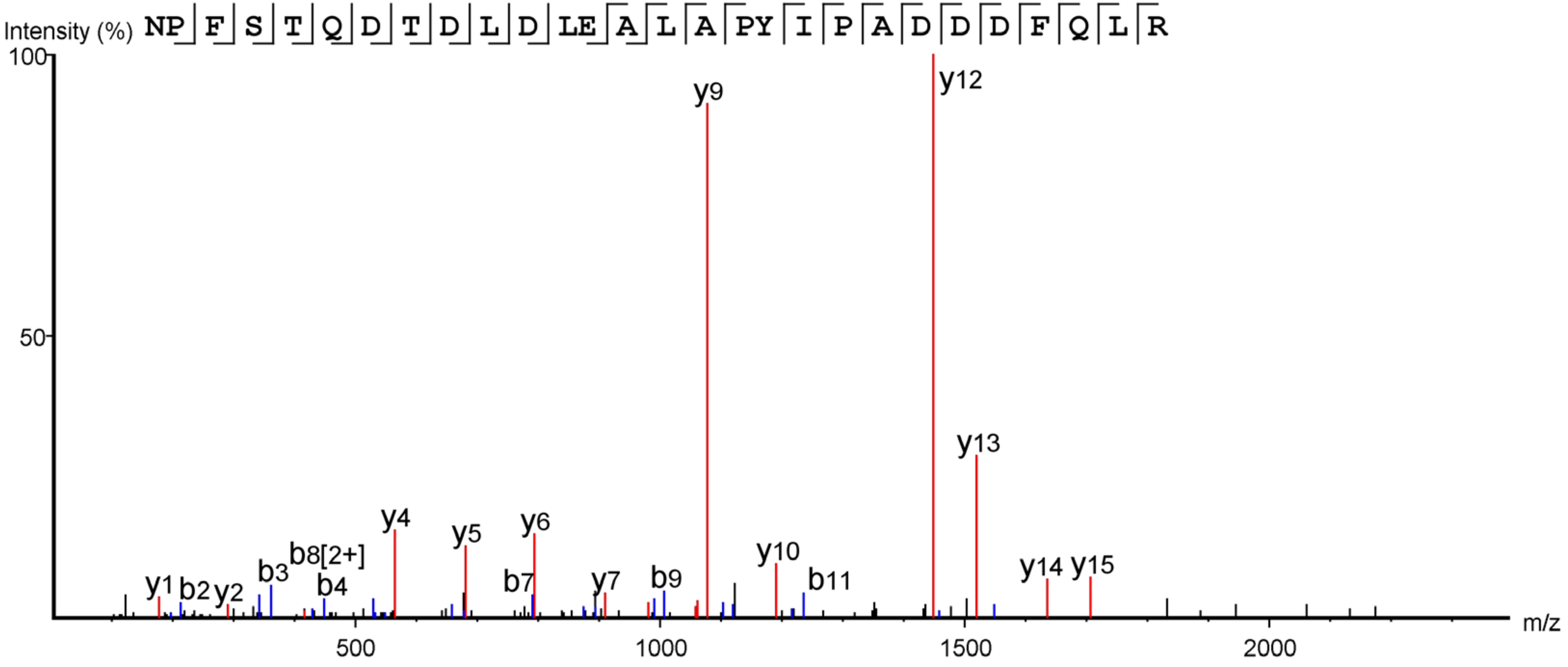

YMT117
Scan #26294

A-320: HIF-1α*$_{(548-575)}$: NPFSTQDTDLDLEALAPYIPADDDFQLR

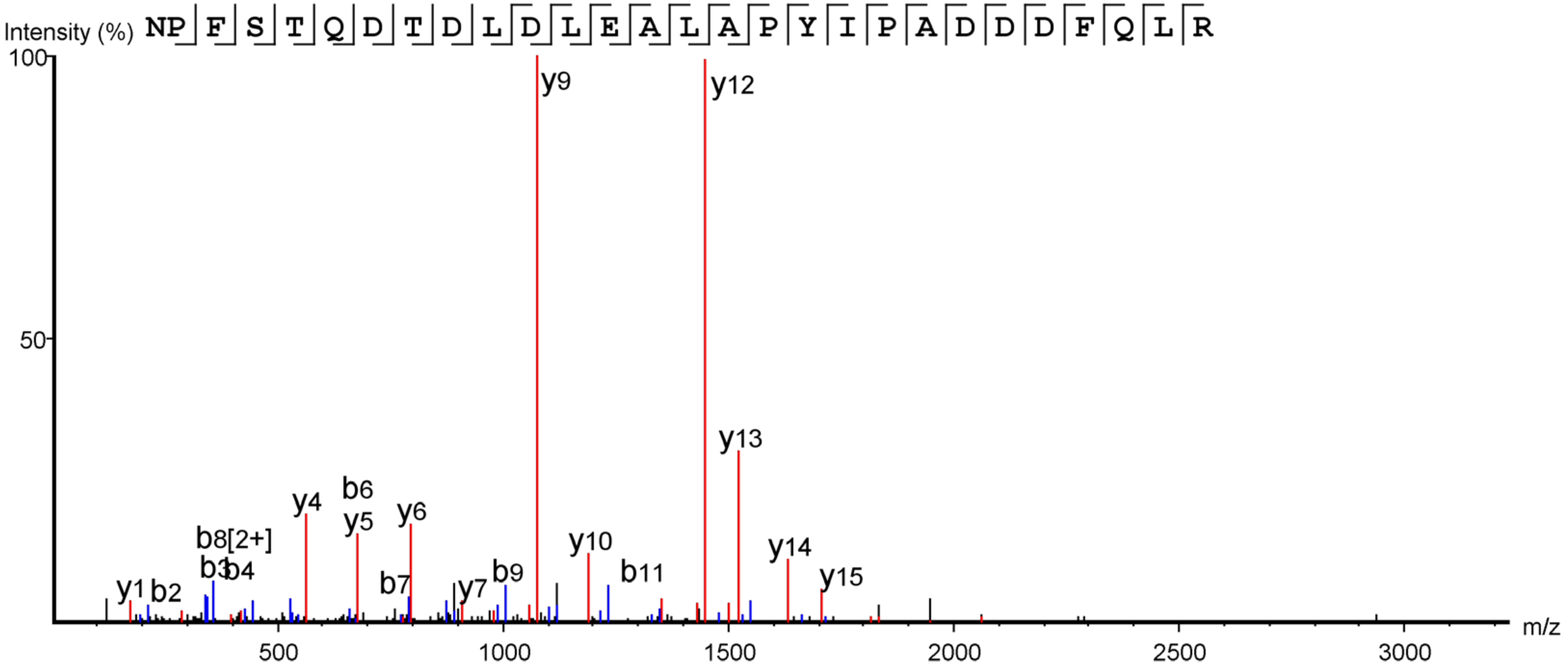

YMT118
Scan #25471

A-321: HIF-1α*$_{(548-575)}$: NPFSTQDTDLDLEALAP(+15.99)YIPADDDFQLR

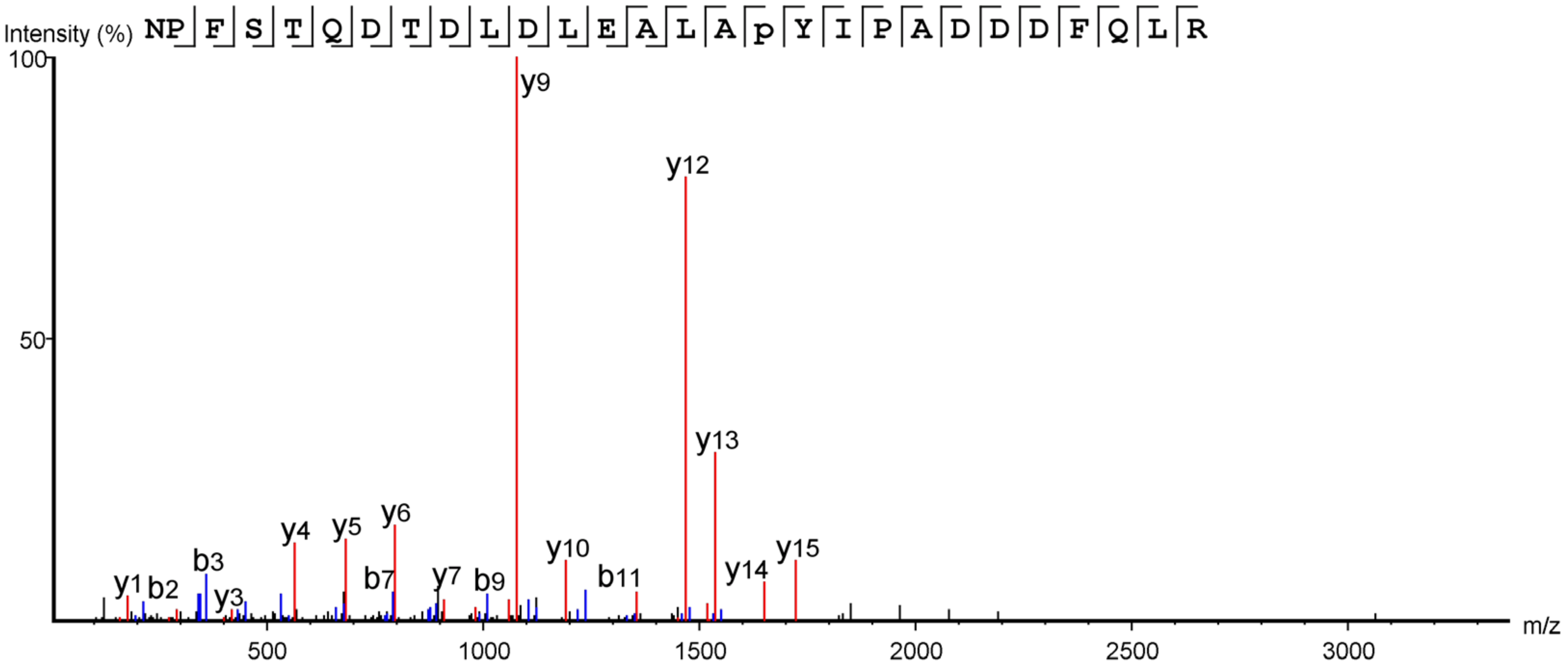

YMT115
Scan #25925

A-322: HIF-1α*$_{(548-575)}$: NPFSTQDTDLDLEALAP(+15.99)YIPADDDFQLR

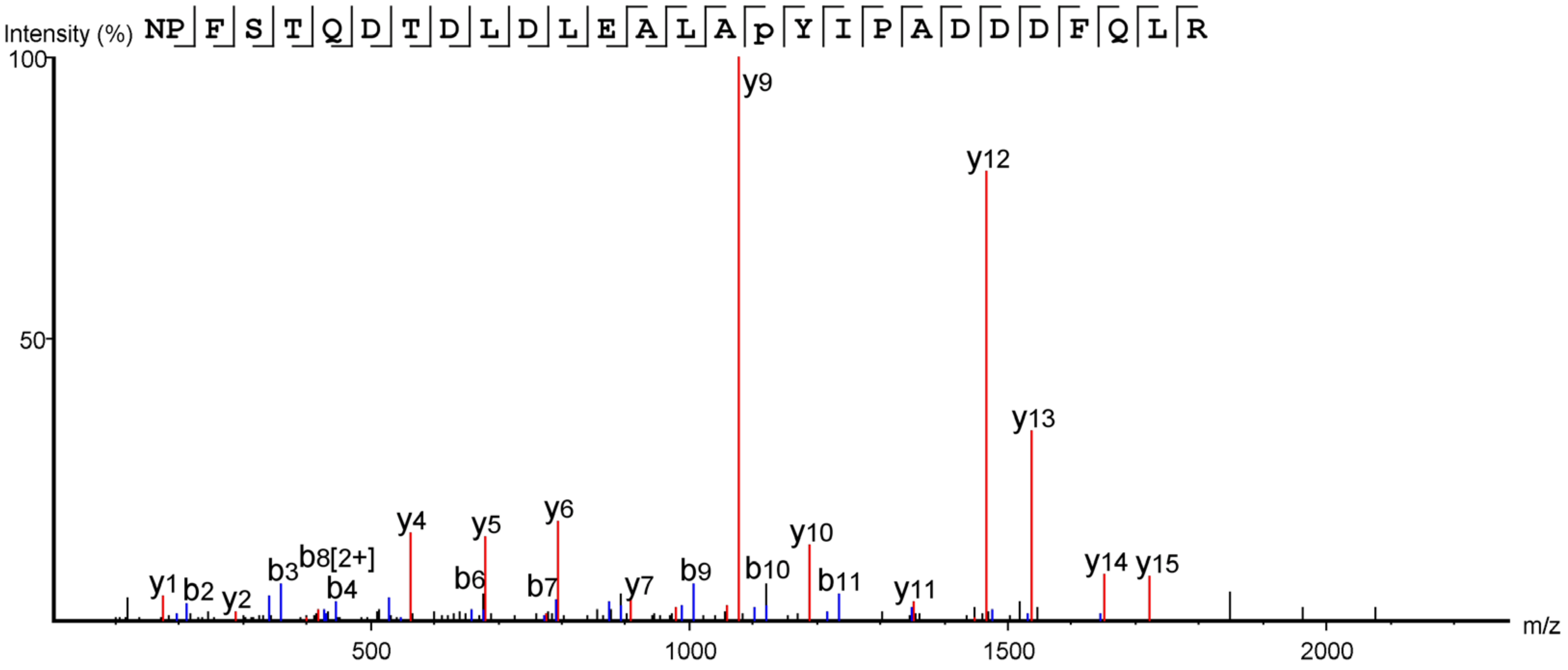

YMT116
Scan #25727

A-323: HIF-1α*$_{(548-575)}$: NPFSTQDTDLDLEALAP(+15.99)YIPADDDFQLR

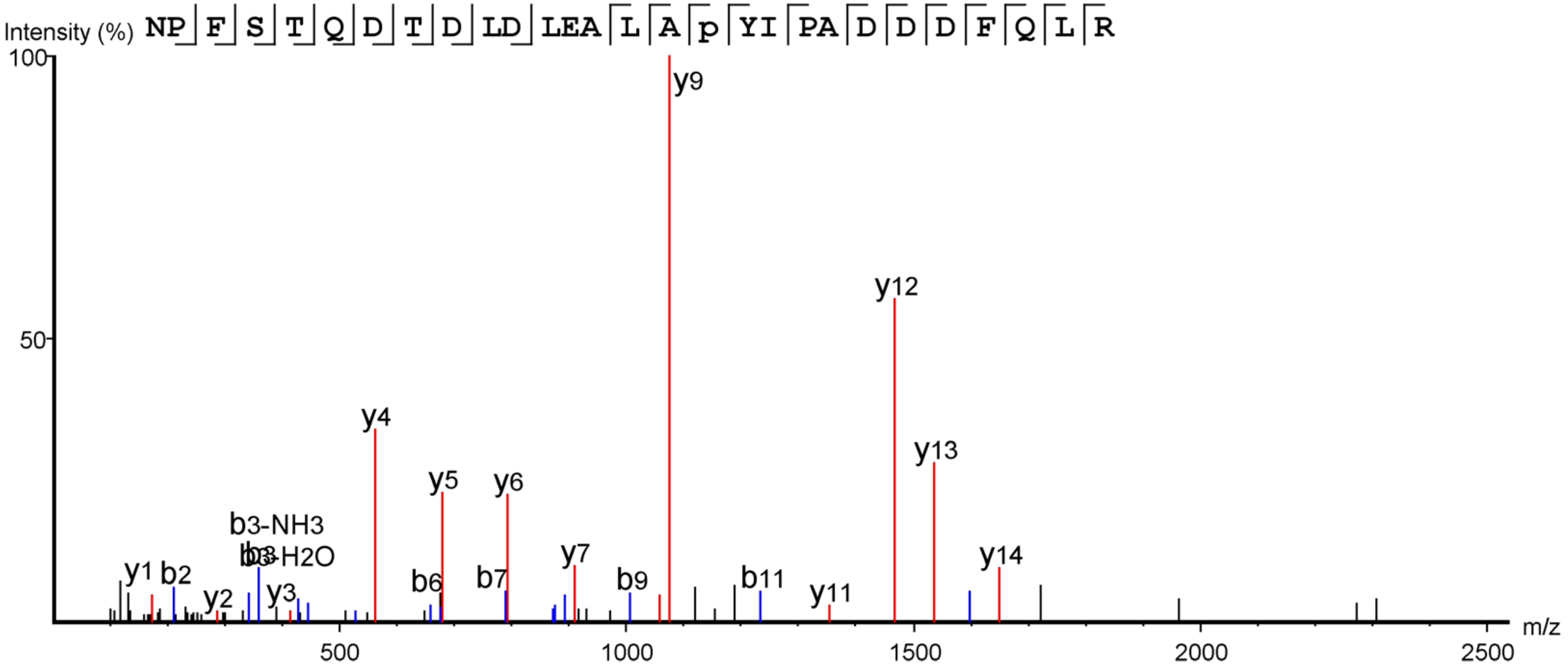

YMT117
Scan #26550

A-324: HIF-1$\alpha^{*}_{(548-575)}$: NPFSTQDTDLDLEALAP(+15.99)YIPADDDFQLR

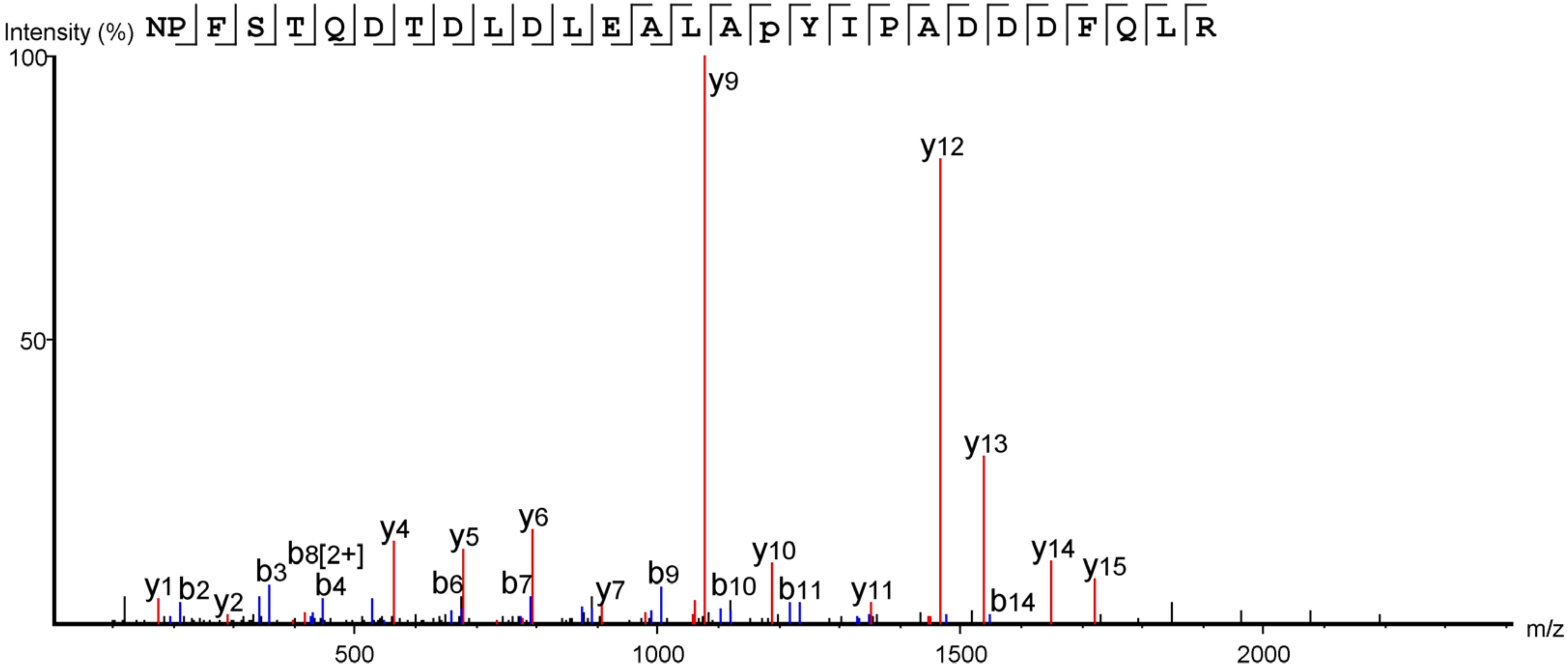

YMT118
Scan #25439

A-325: HIF-1α*$_{(392-411)}$: KEPDALTLLAPAAGDTIISL

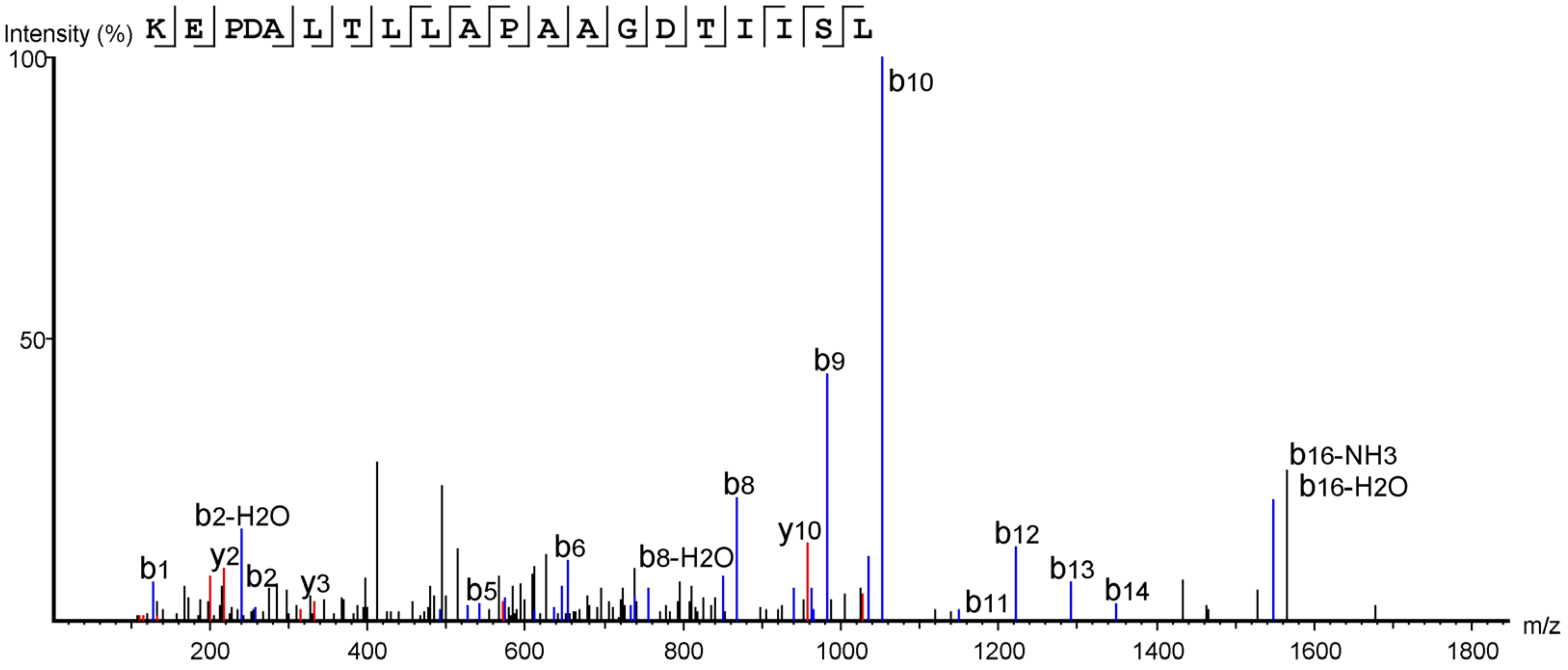

YMT115
Scan #24549

A-326: HIF-1α*(392-411): KEPDALTLLAPAAGDTIISL

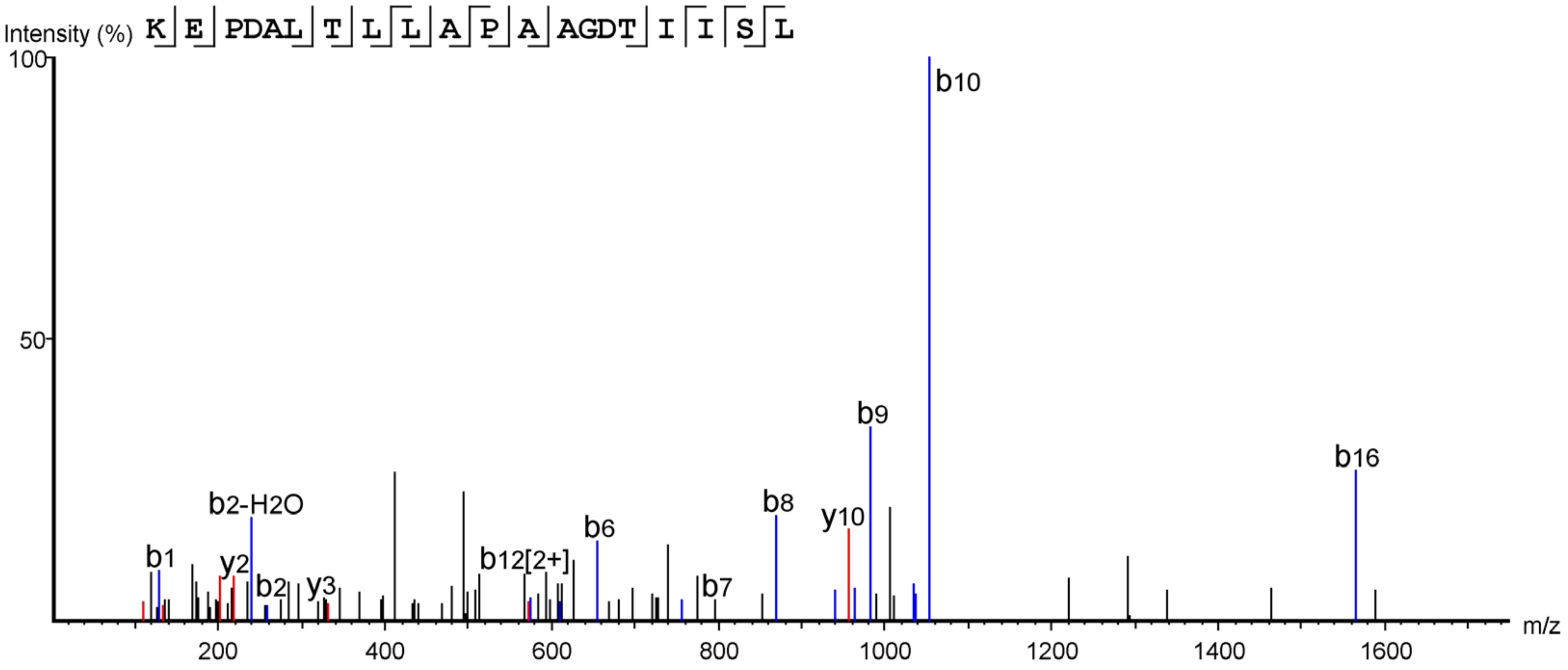

YMT117
Scan #24846

A-327: HIF-1α*(392-411): KEPDALTLLAPAAGDTIISL

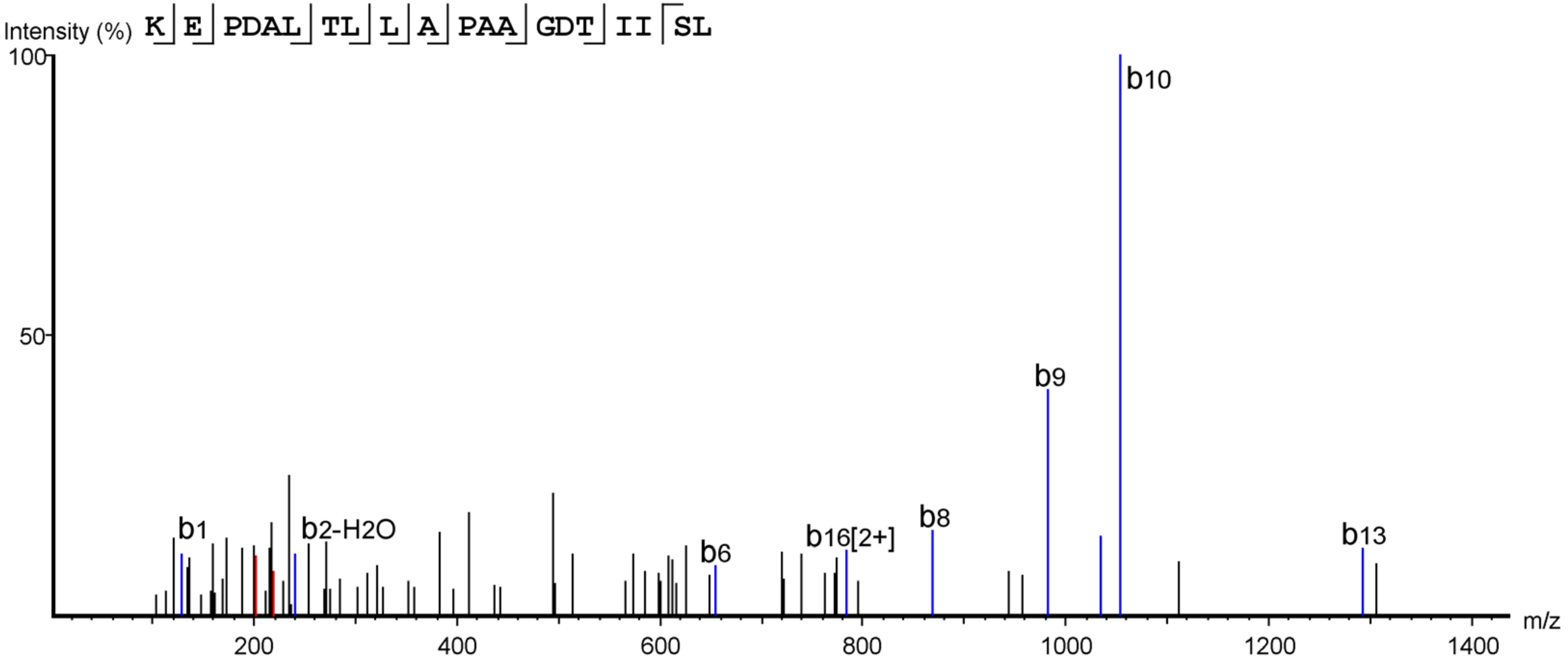

YMT118
Scan #24111

A-328: HIF-1α*(392-411): KEPDALTLLAP(+15.99)AAGDTIISL

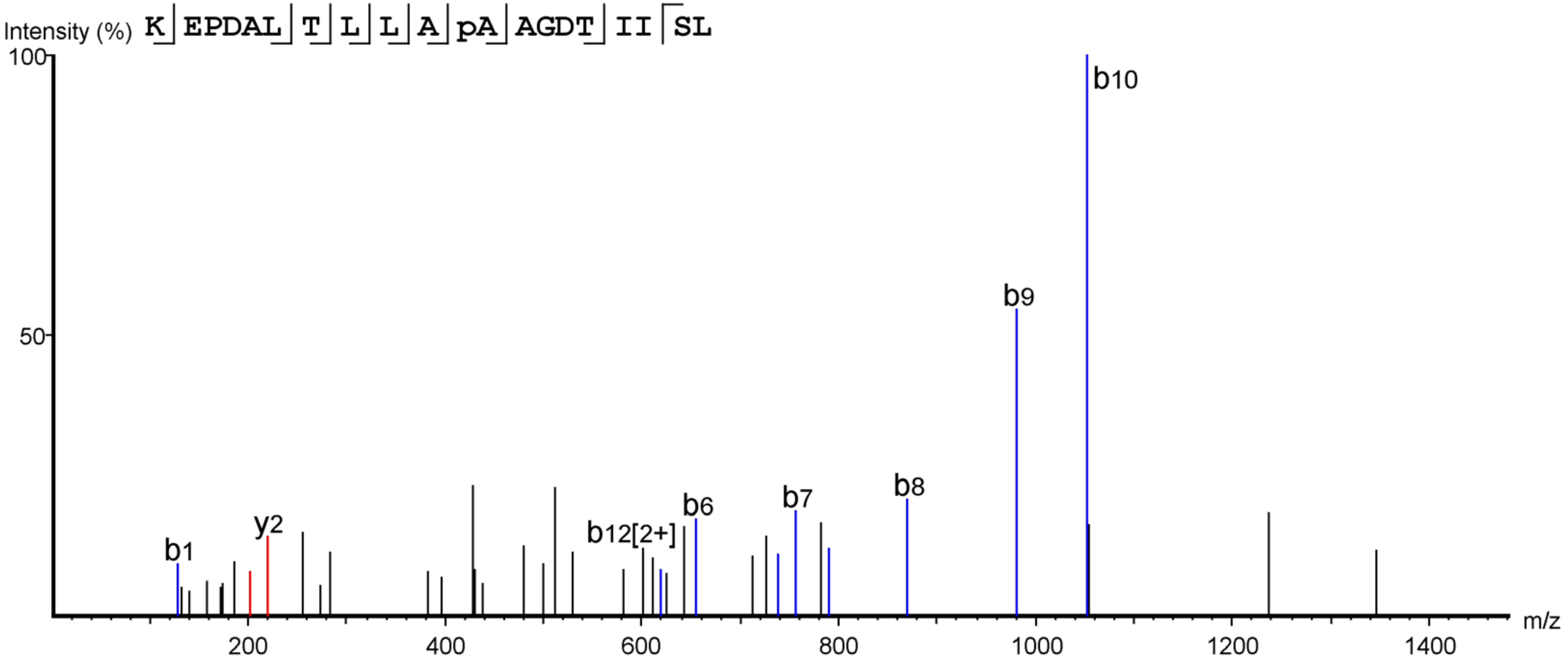

YMT115
Scan #23781

A-329: HIF-1α*(392-411): KEPDALTLLAP(+15.99)AAGDTIISL

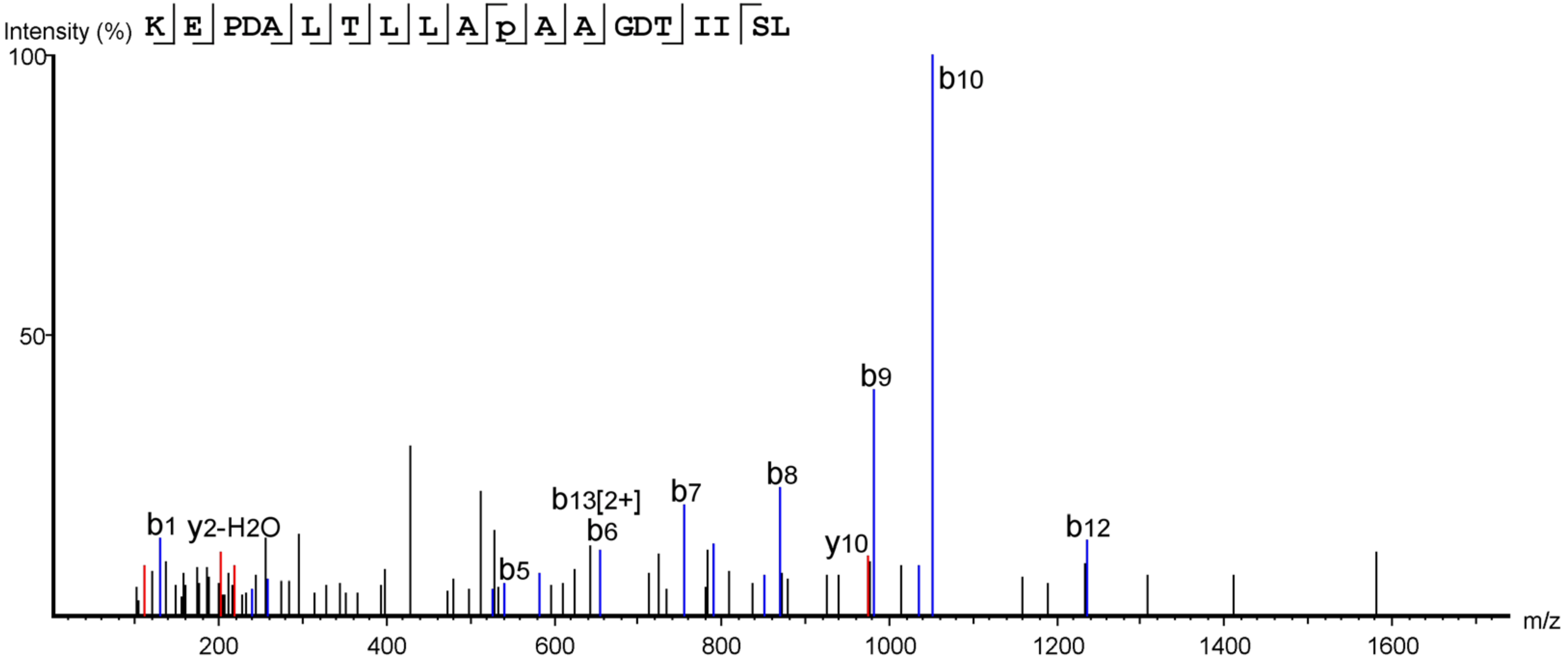

YMT116
Scan #23537

A-330: HIF-1α*(392-411): KEPDALTLLAP(+15.99)AAGDTIISL

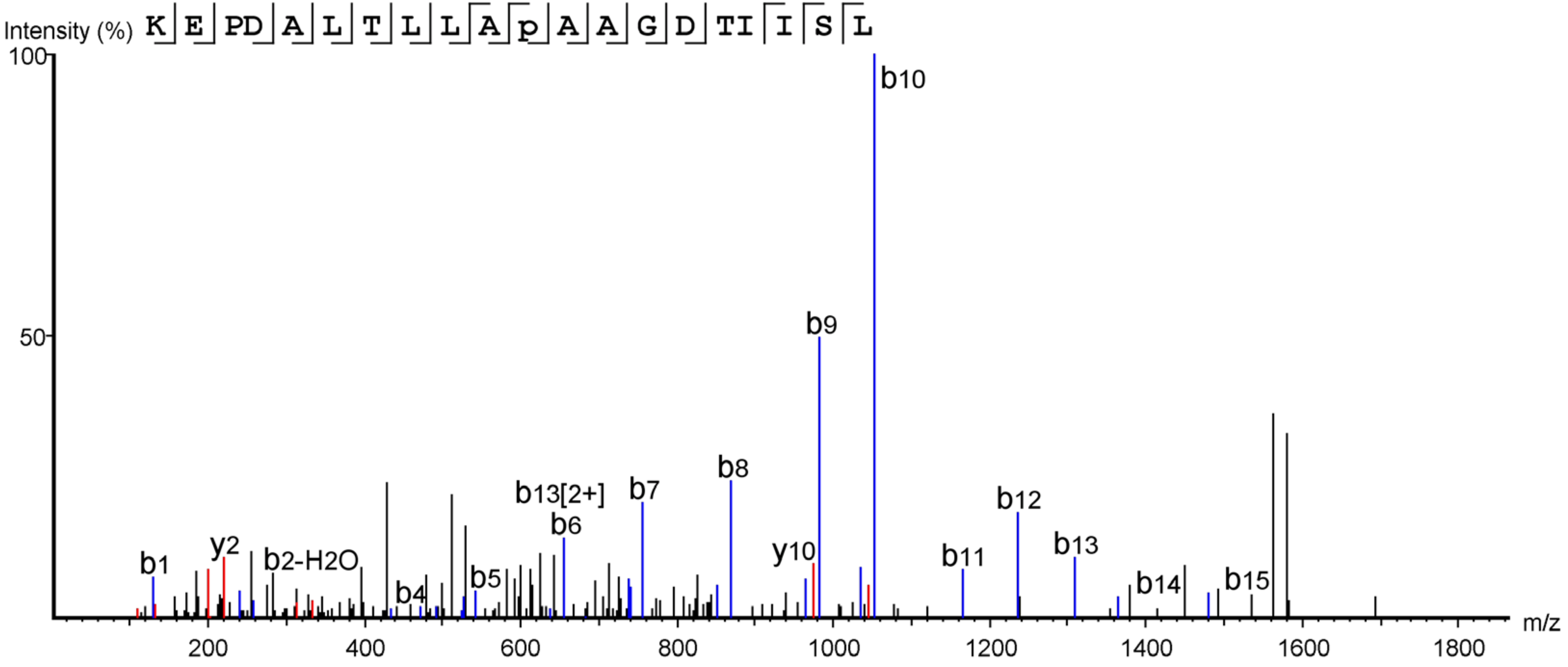

YMT117
Scan #24118

A-331: HIF-2α(513-550): DQC(+57.02)STQTDFNELDLETLAPYIPMDGEDFQLSPIC(+57.02)PEER

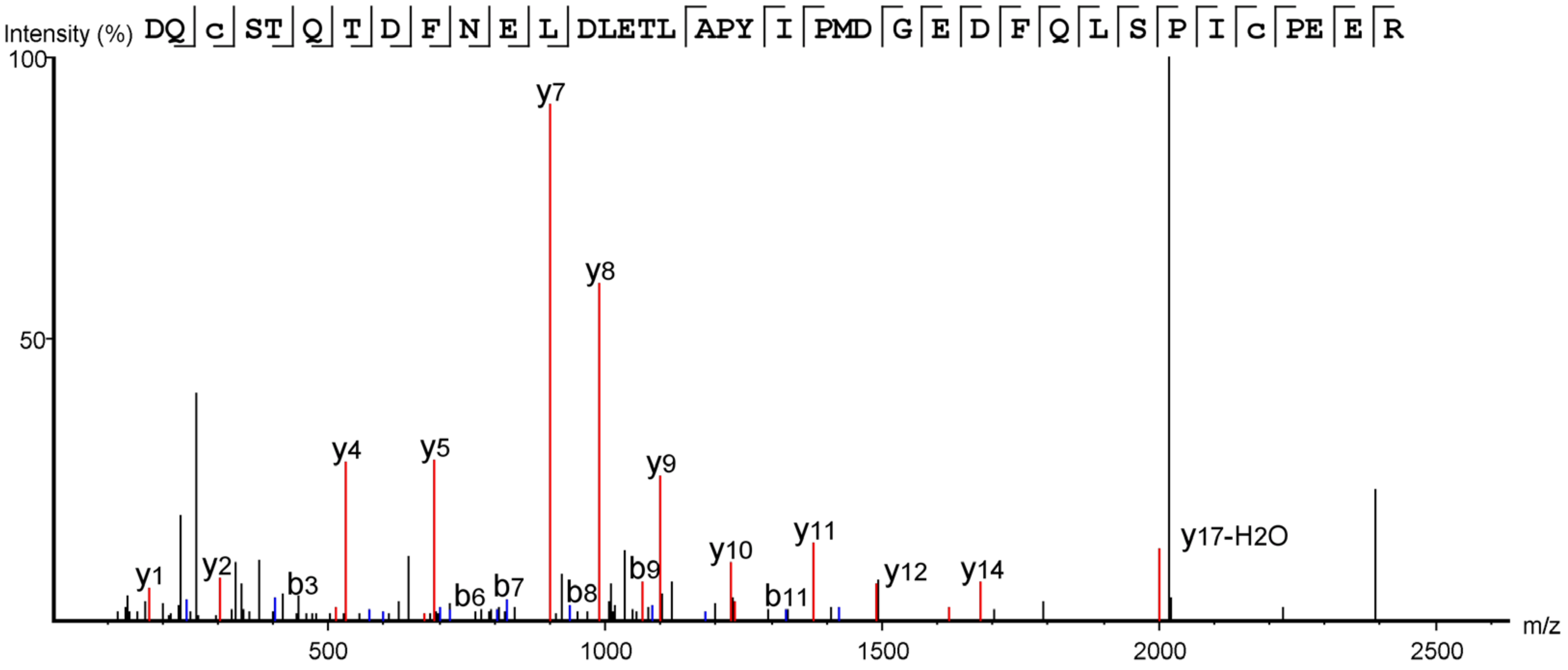

YMT119
Scan #28244

A-332: HIF-2α(513-550): DQC(+57.02)STQTDFNELDLETLAPYIPMDGEDFQLSPIC(+57.02)PEER

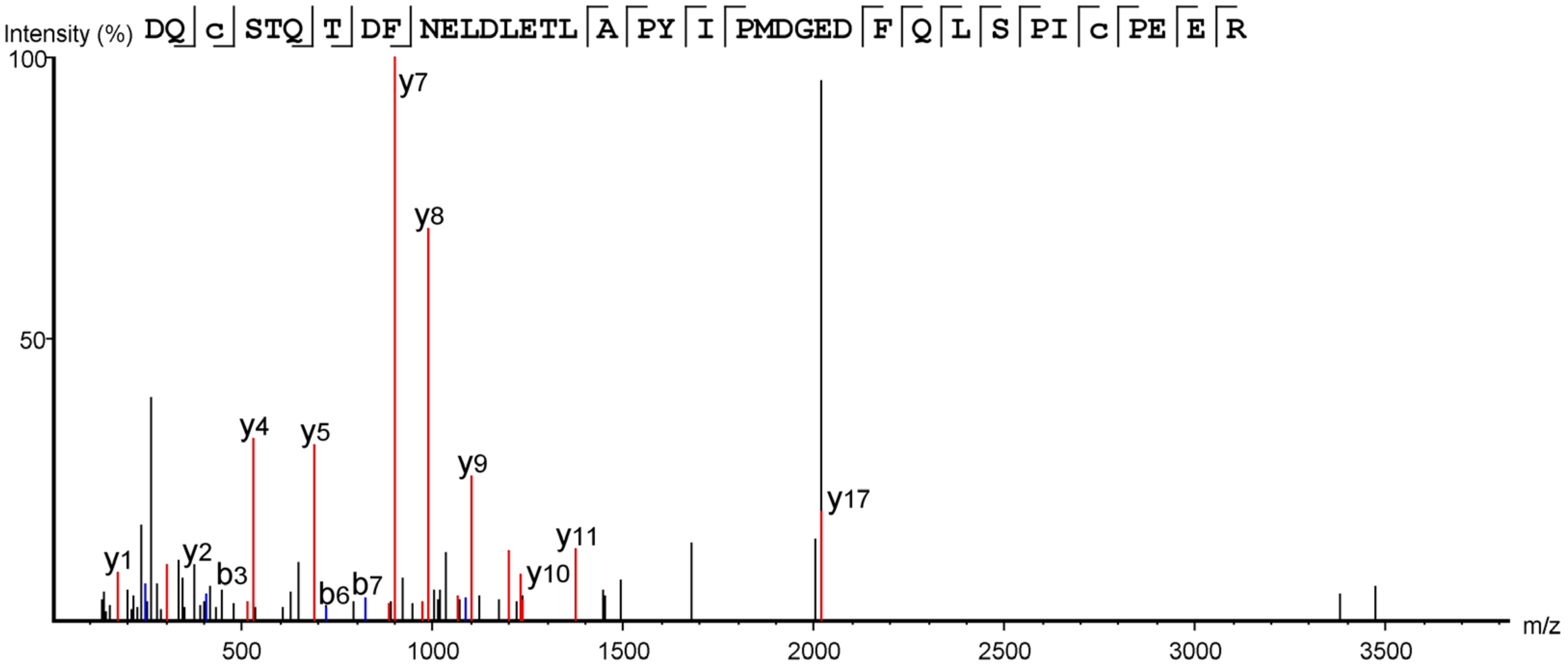

YMT120
Scan #27851

A-333: HIF-2α(513-550): DQC(+57.02)STQTDFNELDLETLAPYIPMDGEDFQLSPIC(+57.02)PEER

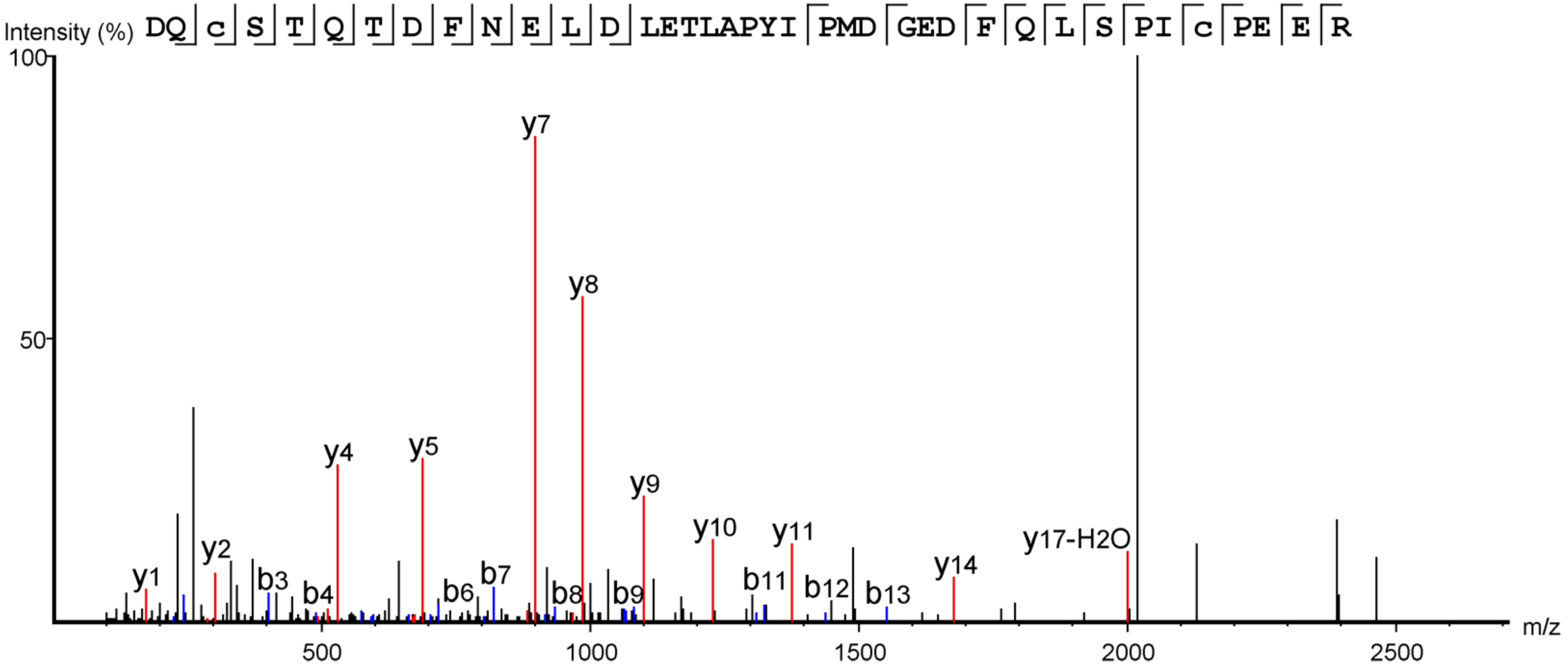

YMT121
Scan #27960

A-334: HIF-2α(513-550): DQC(+57.02)STQTDFNELDLETLAPYIPMDGEDFQLSPIC(+57.02)PEER

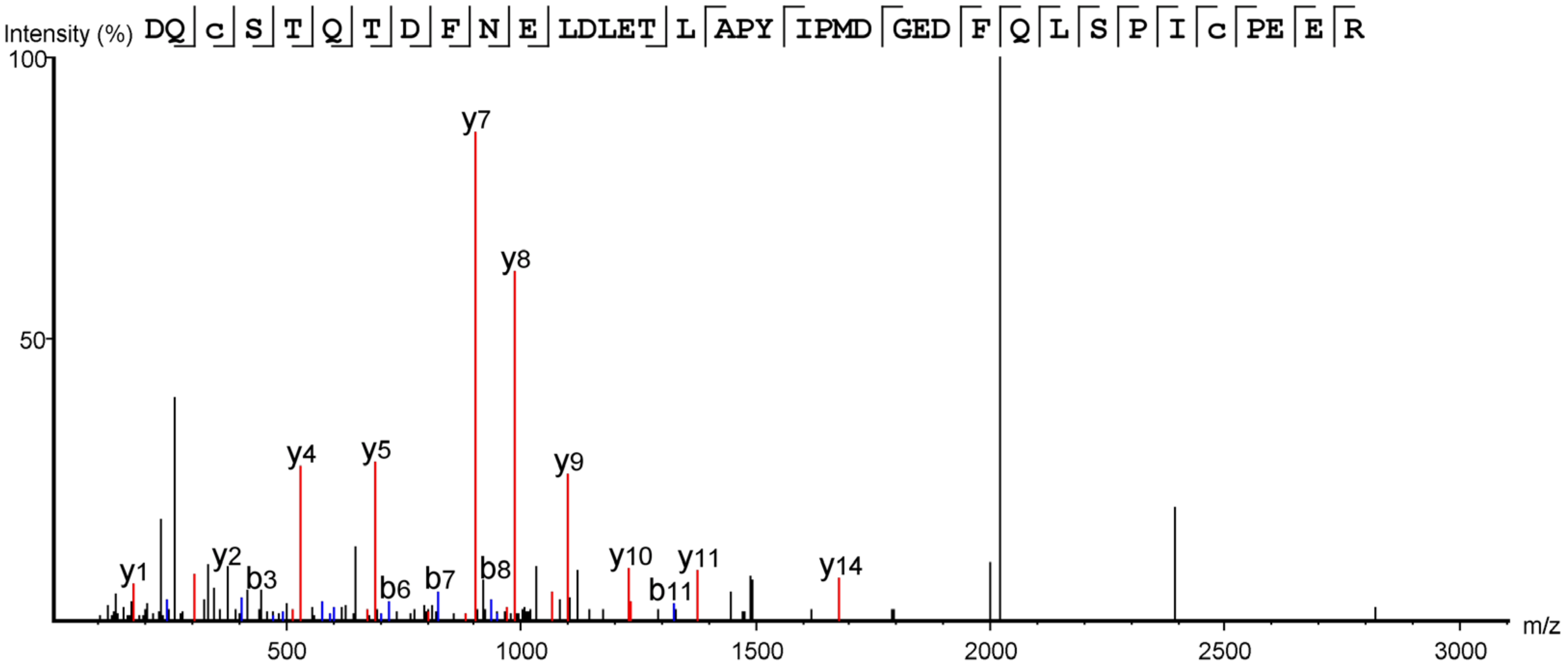

YMT122
Scan #2700

A-335: HIF-2α(513-550): DQC(+57.02)STQTDFNELDLETLAP(+15.99)YIPMDGEDFQLSPIC(+57.02)PEER

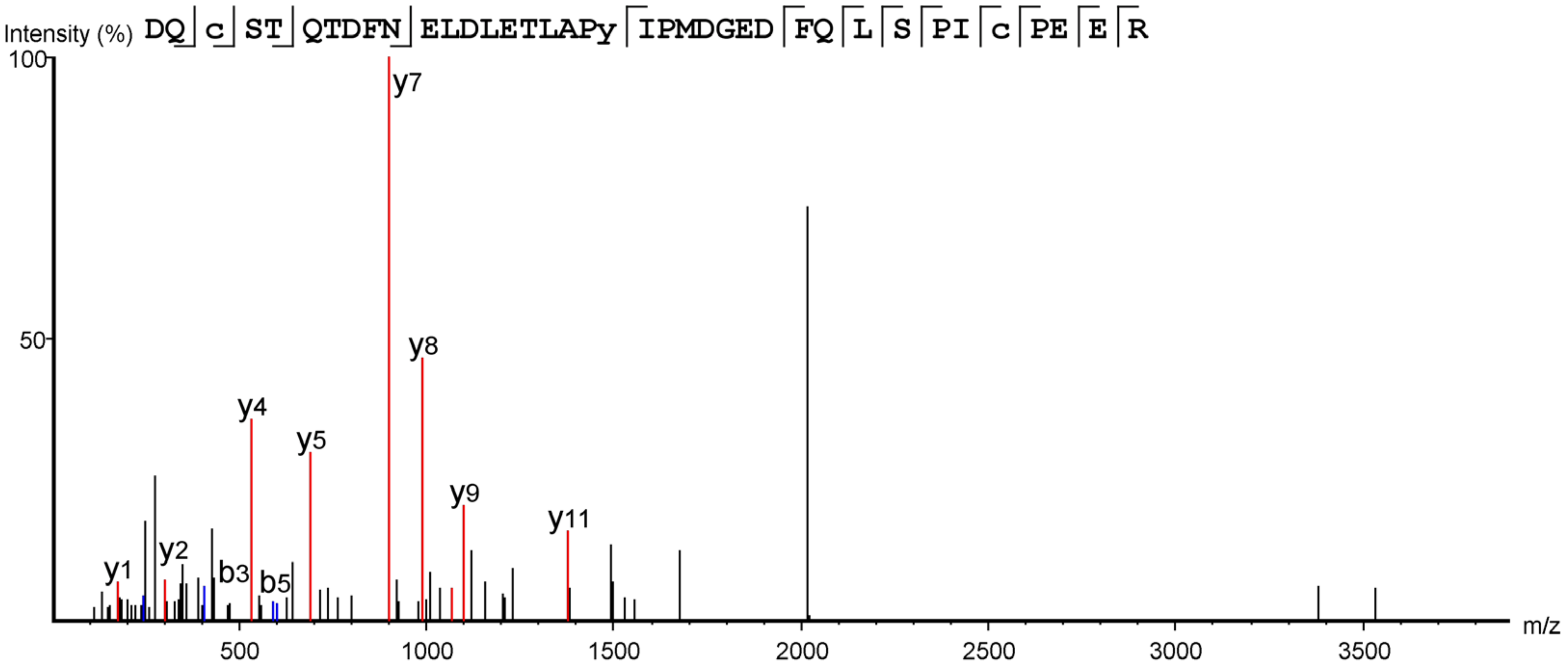

YMT120
Scan #27864

A-336: HIF-2α(513-550): DQC(+57.02)STQTDFNELDLETLAP(+15.99)YIPMDGEDFQLSPIC(+57.02)PEER

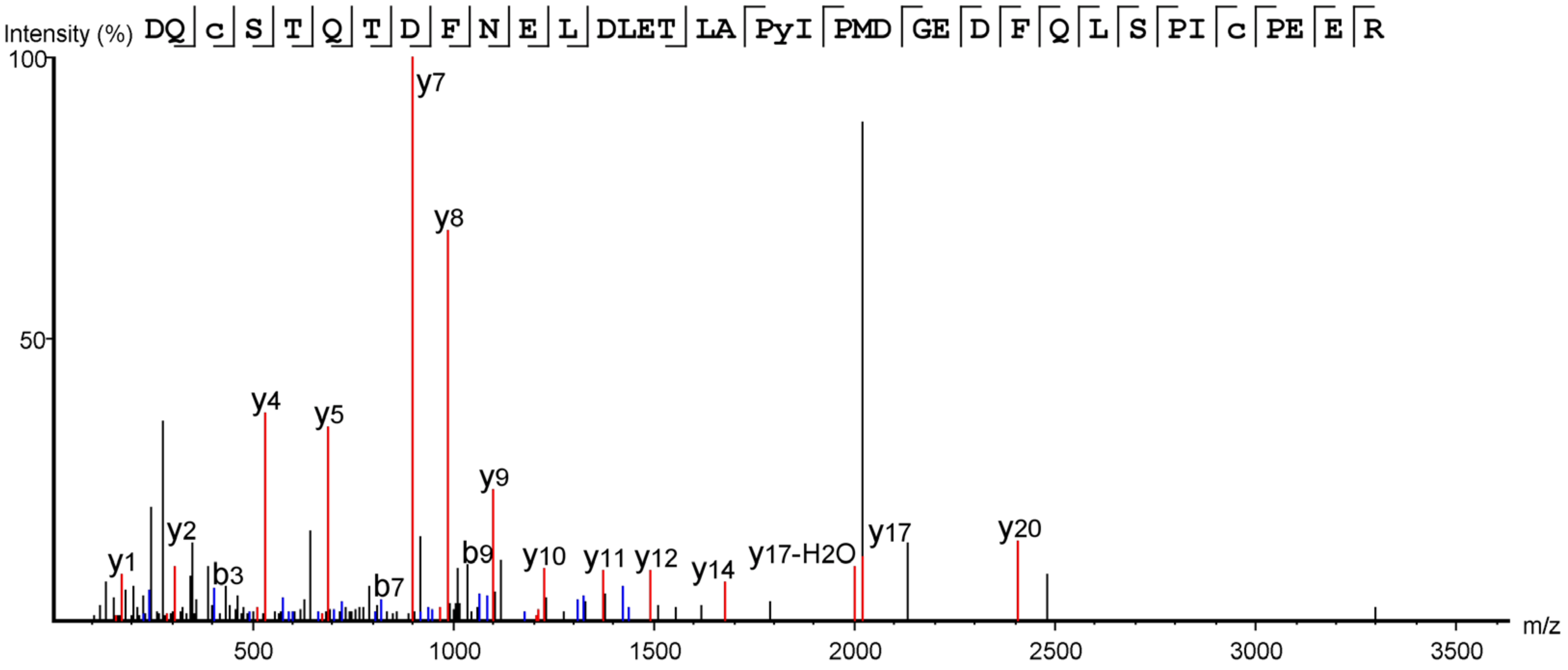

YMT122
Scan #26981

NB: MS2 tolerance increased to 0.04 Da

A-337: HIF-2α(513-550): DQC(+57.02)STQTDFNELDLETLAPYIPM(+15.99)DGEDFQLSPIC(+57.02)PEER

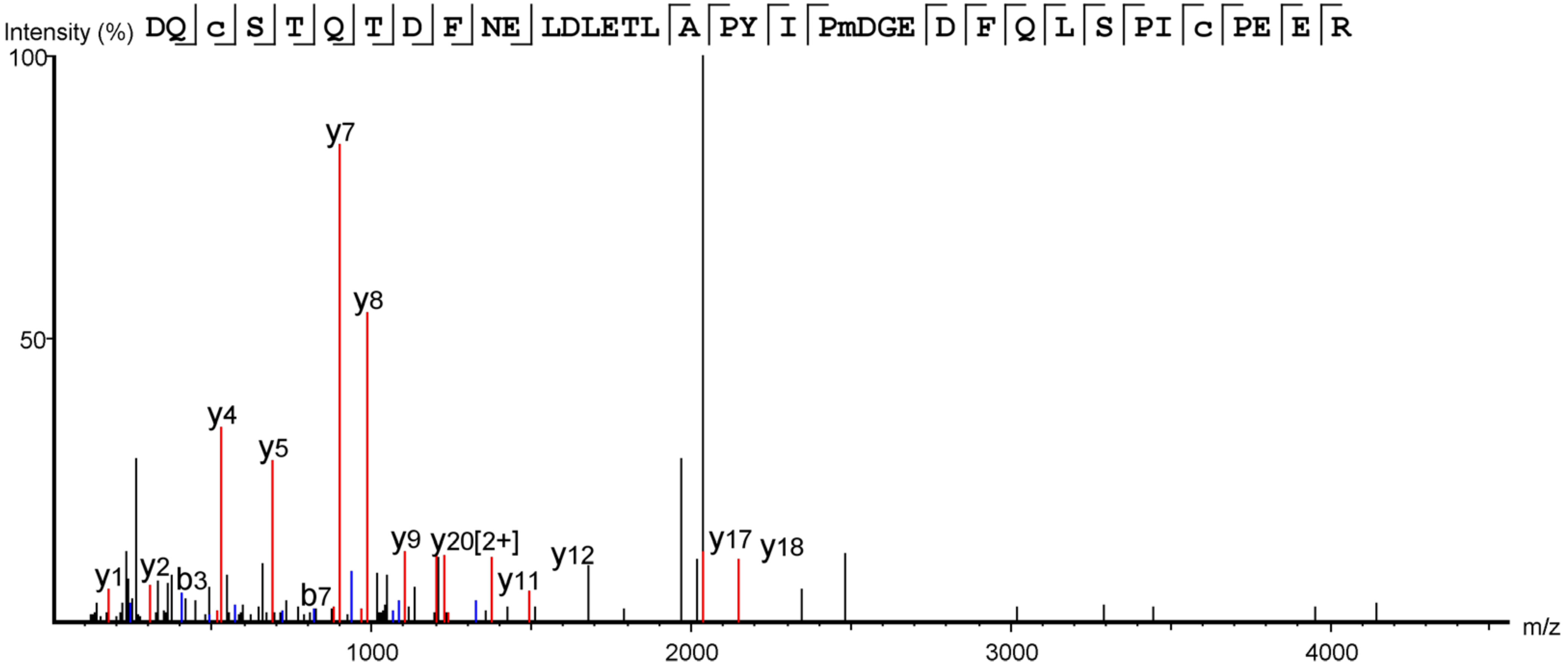

YMT119
Scan #28099

A-338: HIF-2α$_{(513-550)}$: DQC(+57.02)STQTDFNELDLETLAPYIPM(+15.99)DGEDFQLSPIC(+57.02)PEER

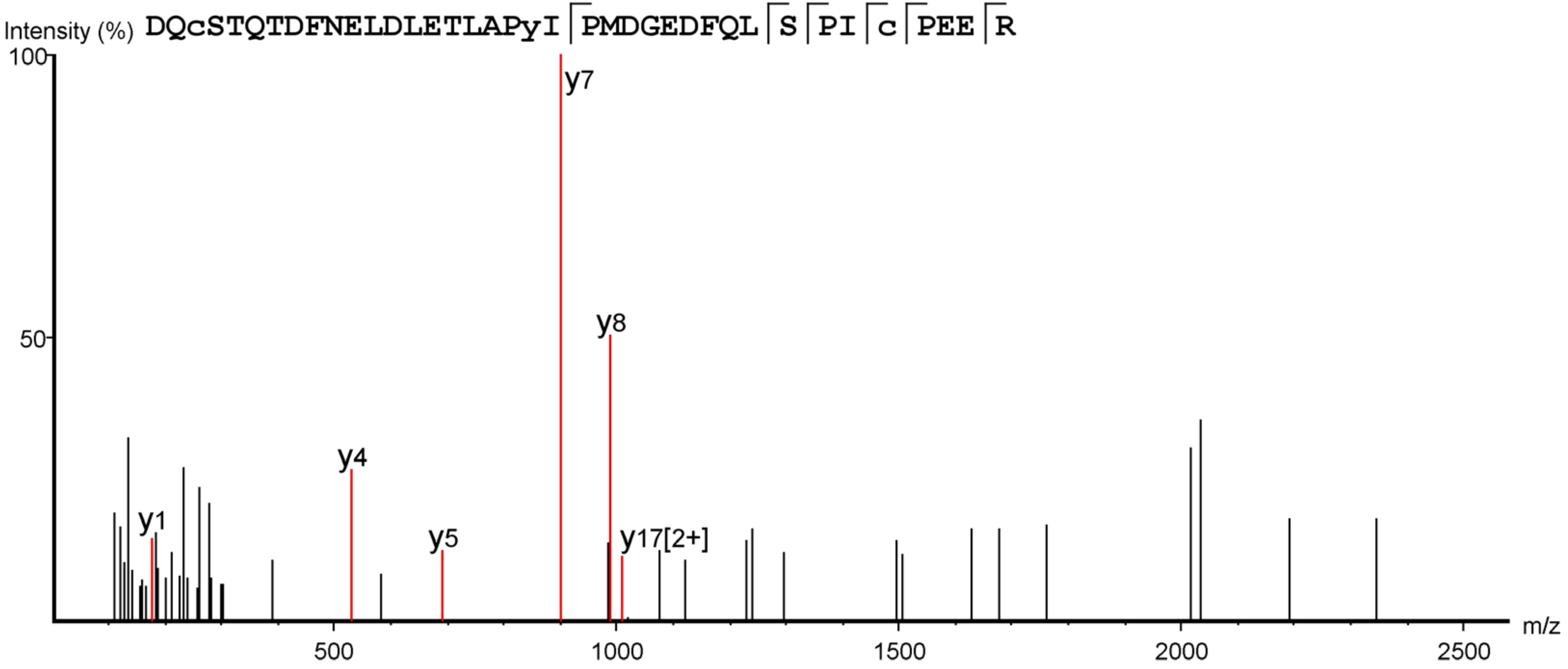

YMT121
Scan #27861

A-339: HIF-2α(513-550): DQC(+57.02)STQTDFNELDLETLAPYIPM(+15.99)DGEDFQLSPIC(+57.02)PEER

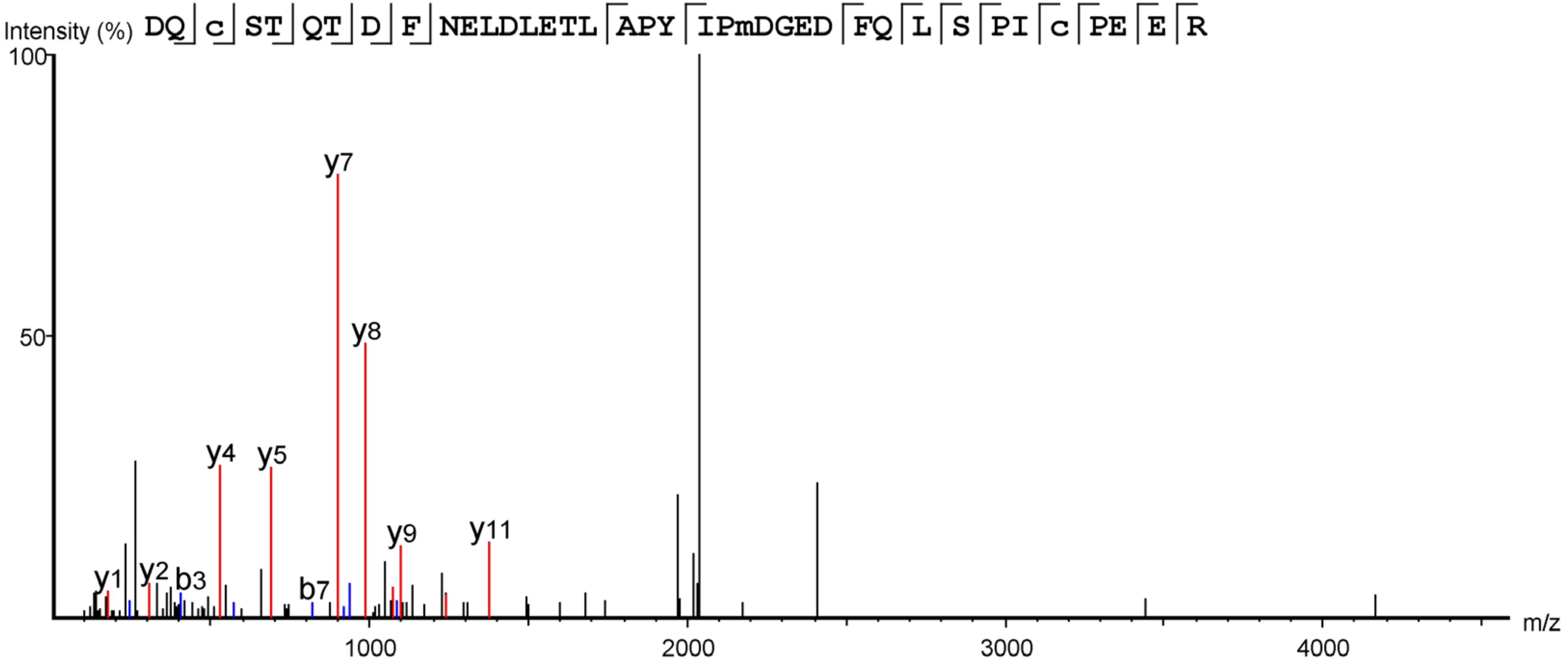

YMT122
Scan #26888

A-340: HIF-2α(513-550): DQC(+57.02)STQTDFNELDLETLAP(+15.99)YIPM(+15.99)DGEDFQLSPIC(+57.02)PEER

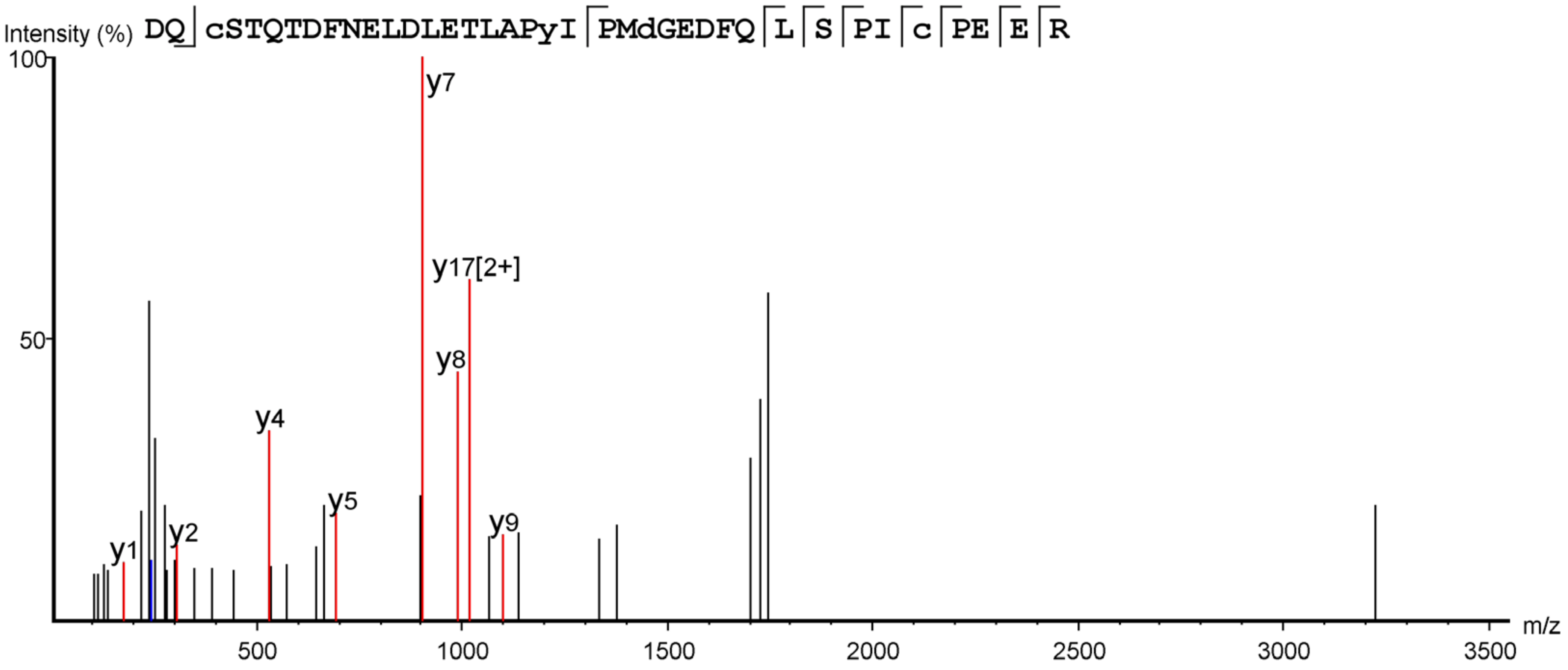

YMT119
Scan #28036

A-341: HIF-2α(513-550): DQC(+57.02)STQTDFNELDLETLAP(+15.99)YIPM(+15.99)DGEDFQLSPIC(+57.02)PEER

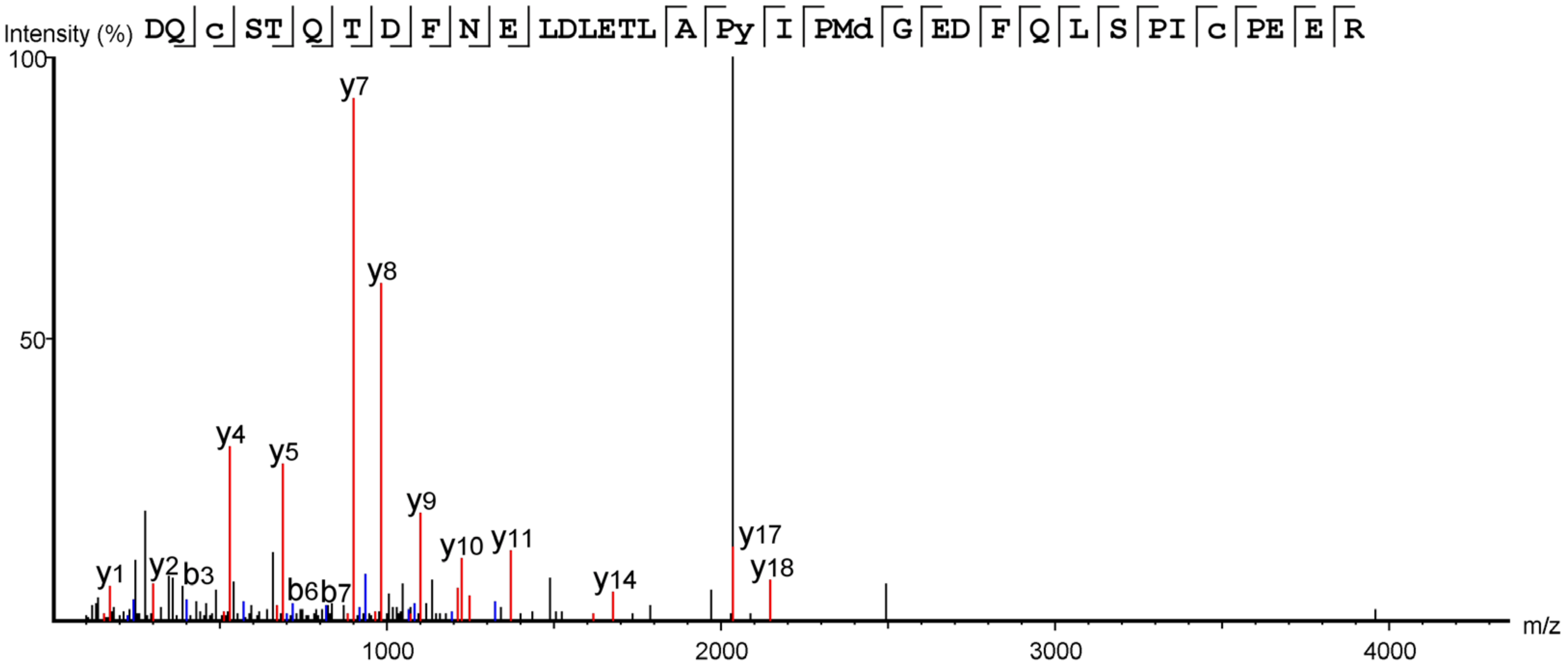

YMT120
Scan #27672

A-342: HIF-2α$_{(513-550)}$: DQC(+57.02)STQTDFNELDLETLAP(+15.99)YIPM(+15.99)DGEDFQLSPIC(+57.02)PEER

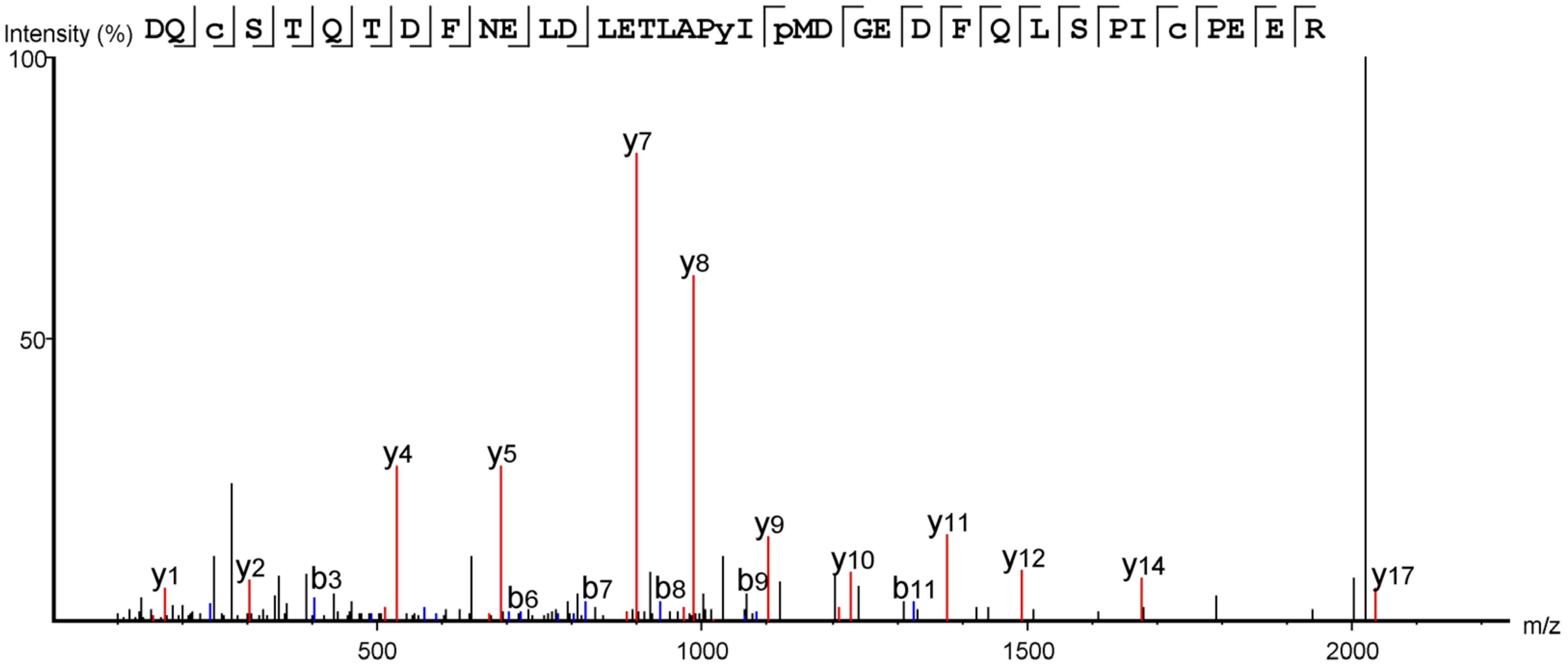

YMT121
Scan #27957

A-343: HIF-2α$_{(513-550)}$: DQC(+57.02)STQTDFNELDLETLAP(+15.99)YIPM(+15.99)DGEDFQLSPIC(+57.02)PEER

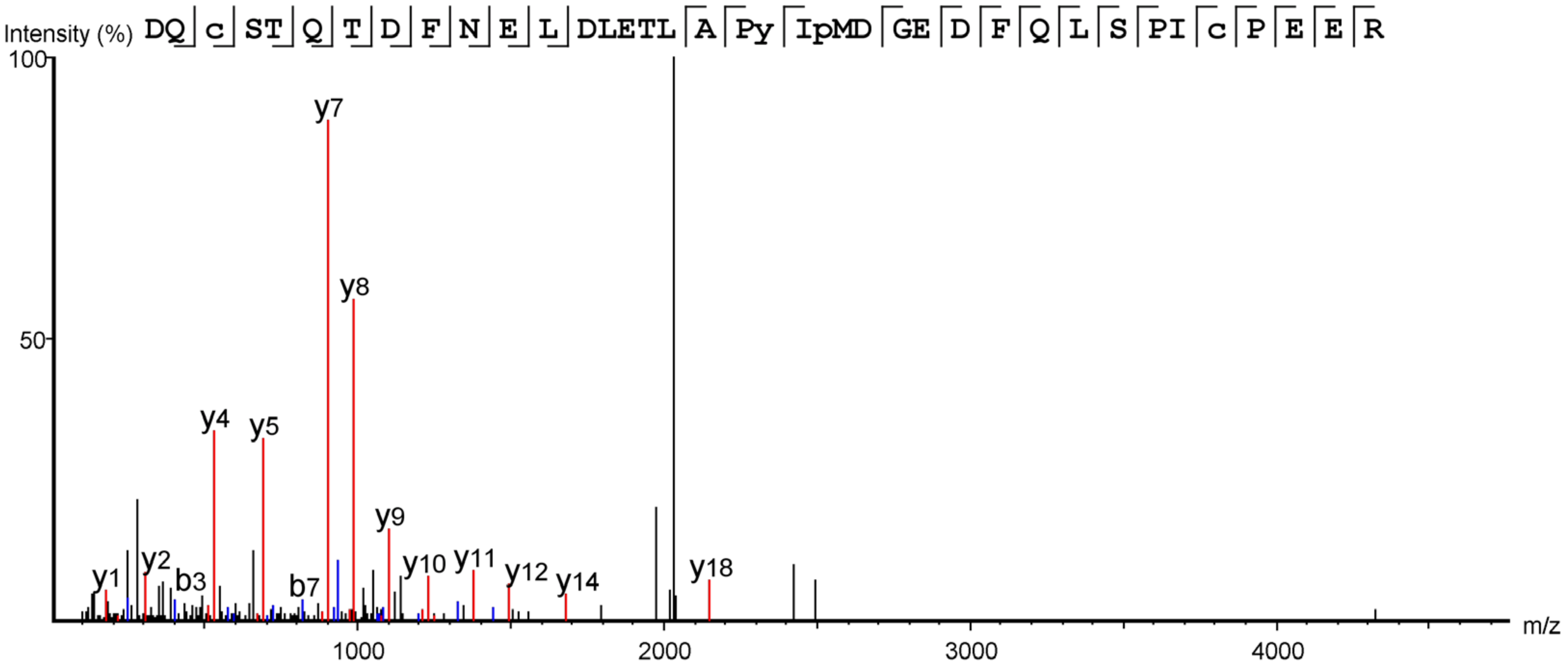

YMT122
Scan #26823

A-344: HIF-2α(393-429): LKEEPEELAQLAPTPGDAIISLDFGNQNFEESSAYGK

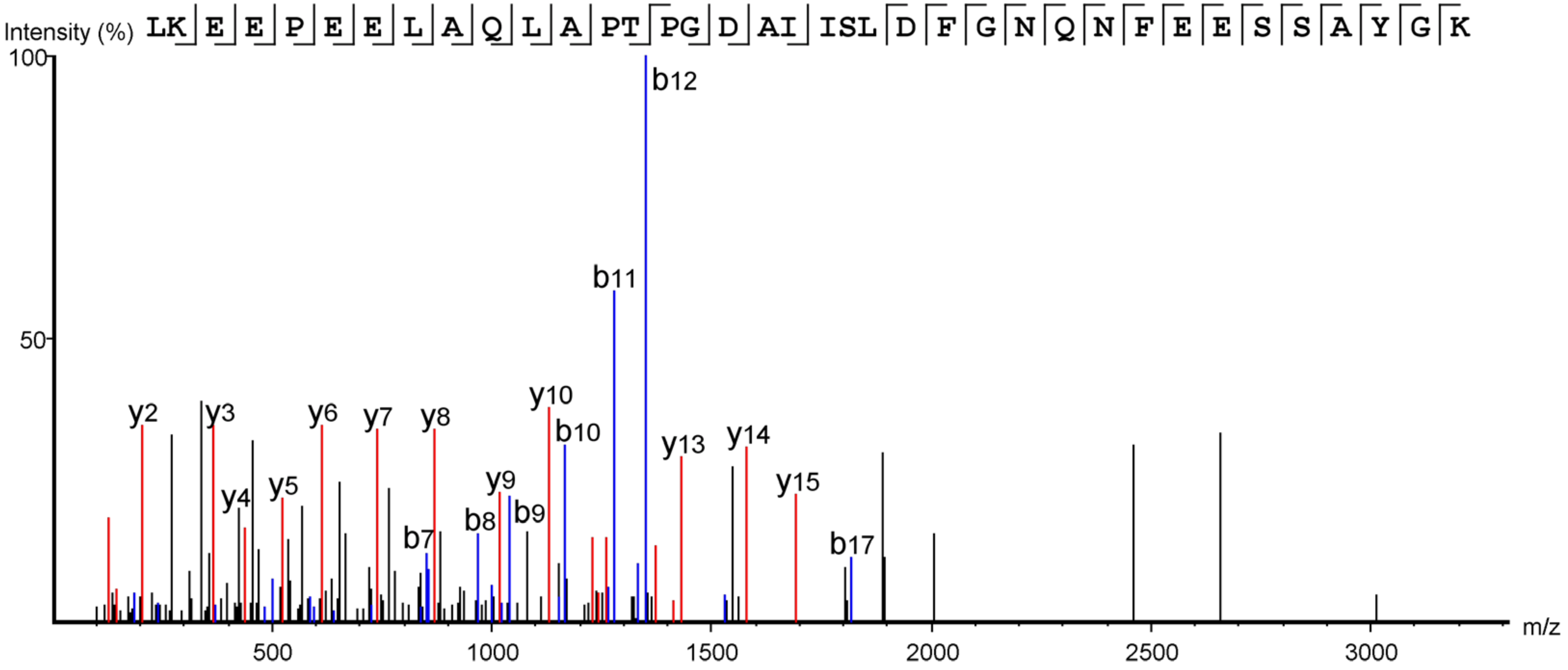

YMT119
Scan #23298

A-345: HIF-2α(393-429): LKEEPEELAQLAPTPGDAIISLDFGNQNFEESSAYGK

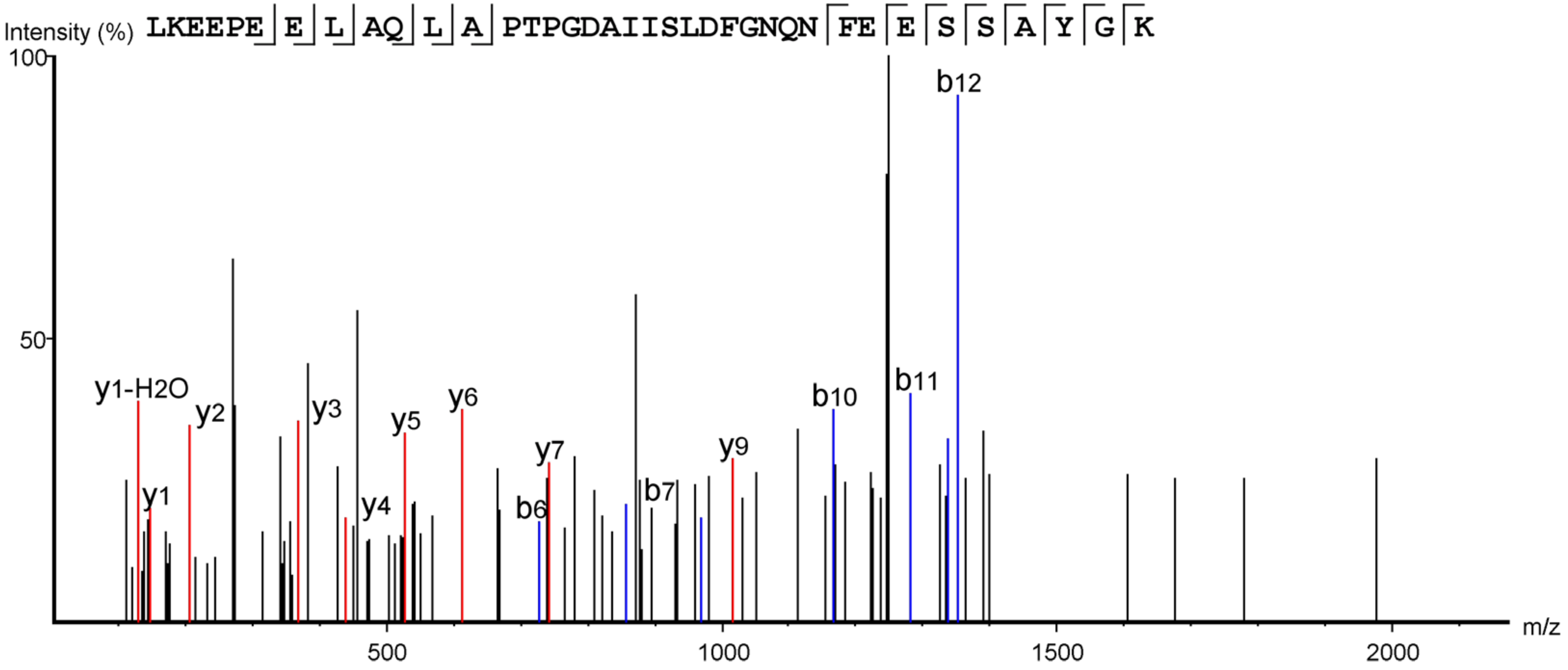

YMT120
Scan #22909

A-346: HIF-2α(393-429): LKEEPEELAQLAPTPGDAIISLDFGNQNFEESSAYGK

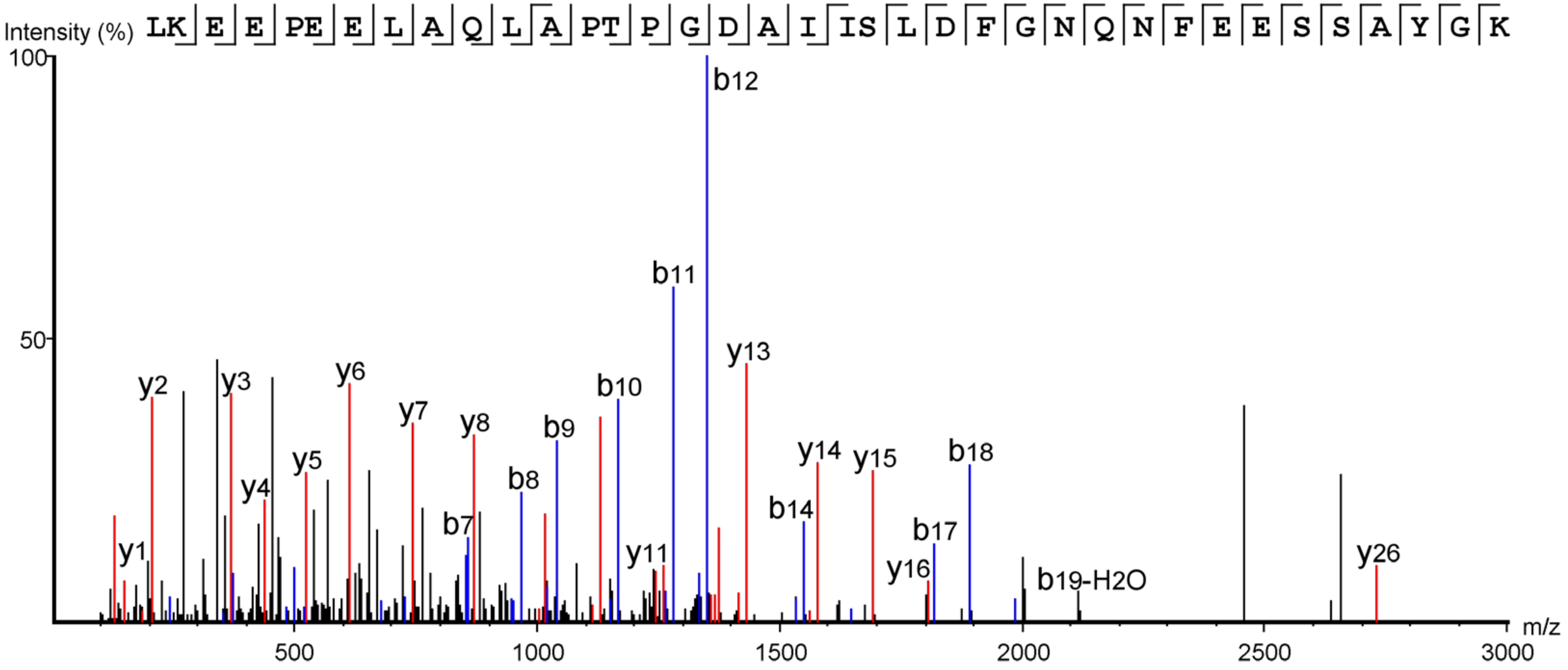

YMT121
Scan #22661

A-347: HIF-2α(393-429): LKEEPEELAQLAPTPGDAIISLDFGNQNFEESSAYGK

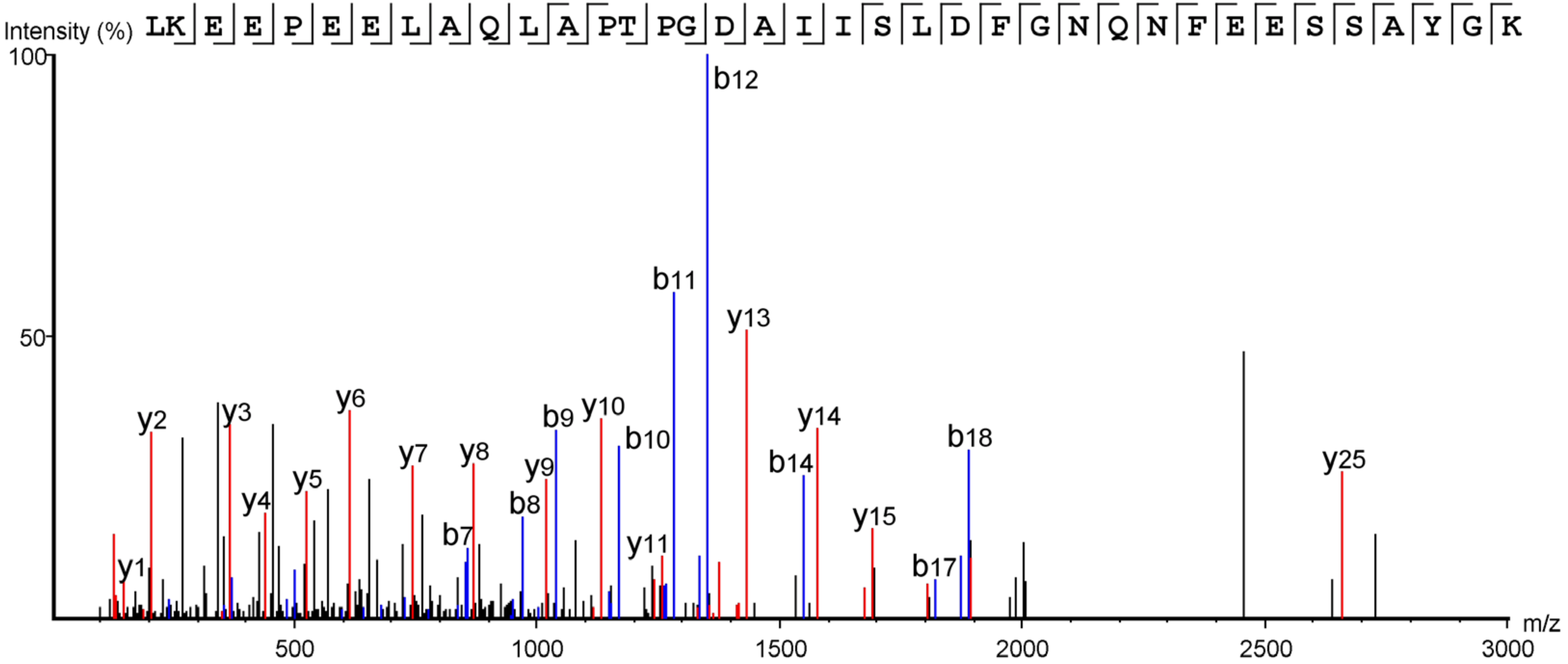

YMT122
Scan #22004

A-348: HIF-2α$_{(393-429)}$: LKEEPEELAQLAP(+15.99)TPGDAIISLDFGNQNFEESSAYGK

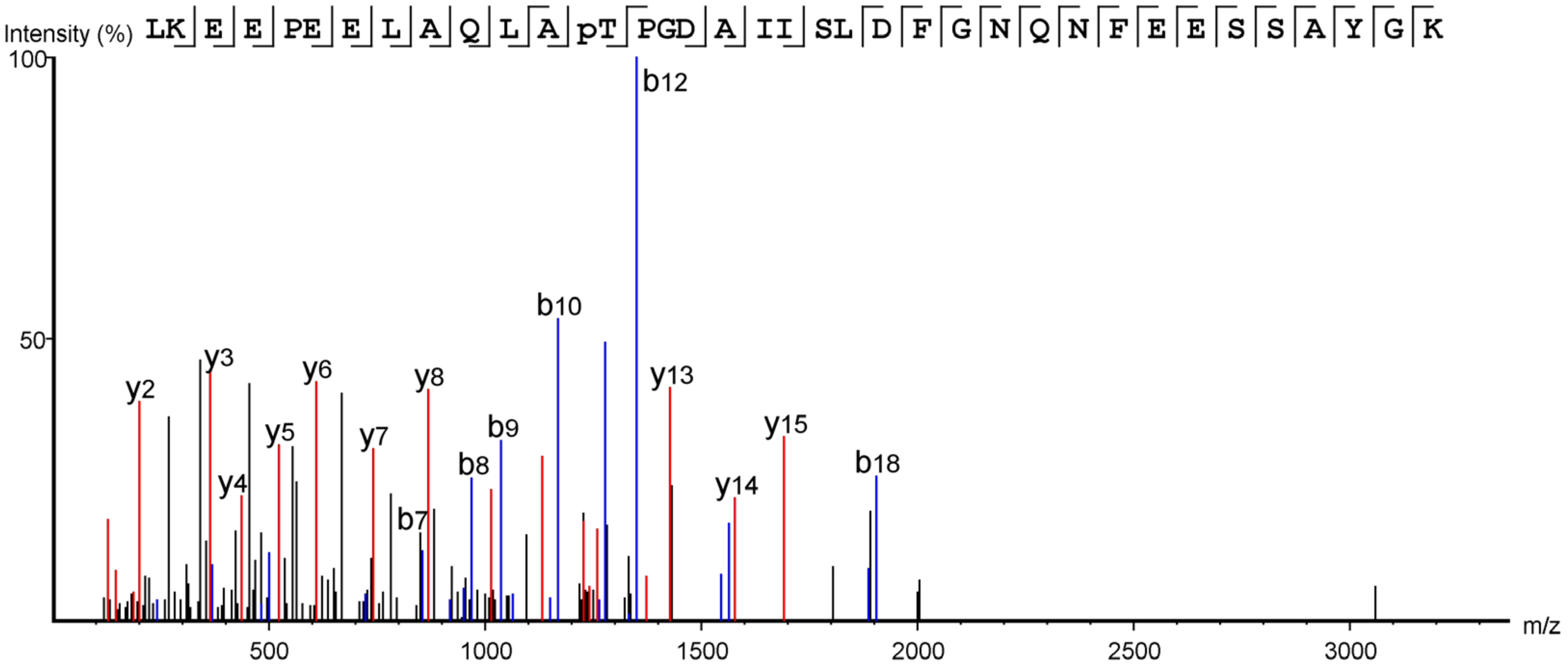

YMT119
Scan #22482

A-349: HIF-2α(393-429): LKEEPEELAQLAP(+15.99)TPGDAIISLDFGNQNFEESSAYGK

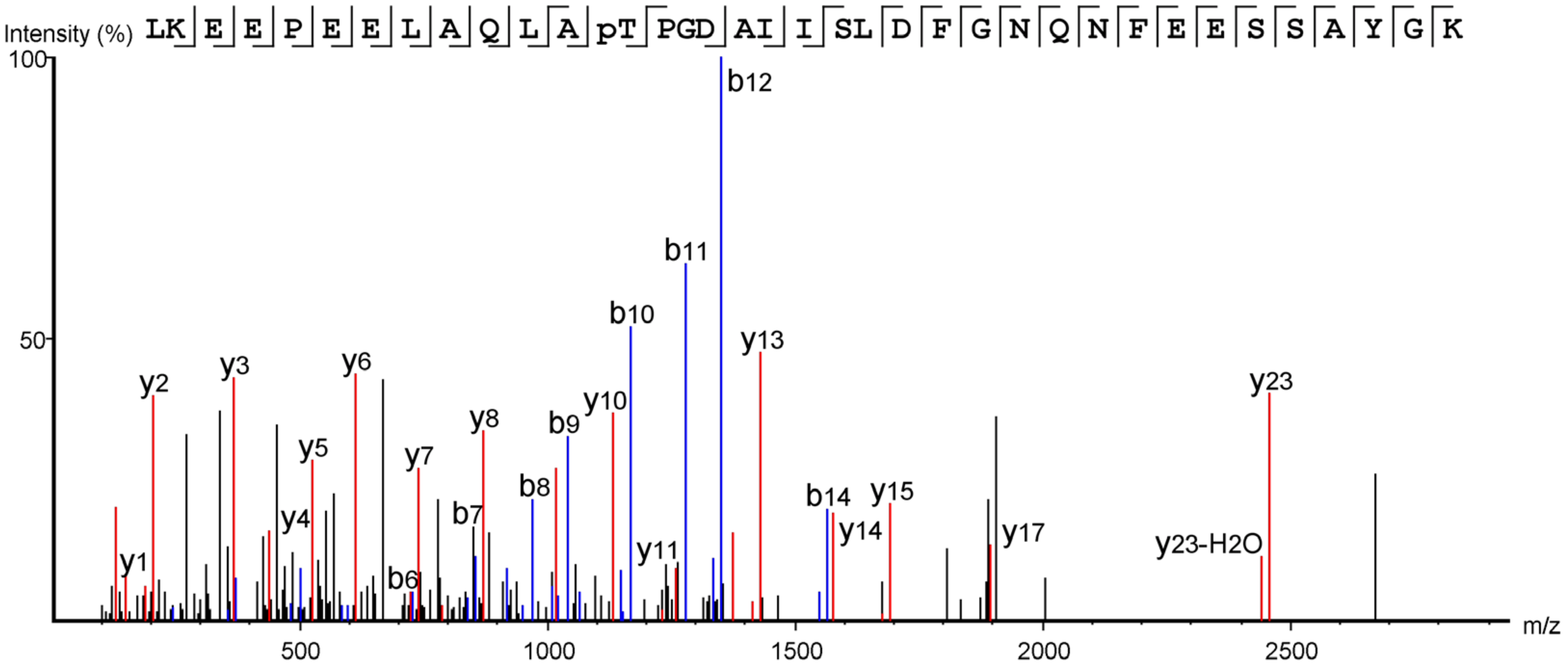

YMT120
Scan #21997

A-350: HIF-2α(393-429): LKEEPEELAQLAP(+15.99)TPGDAIISLDFGNQNFEESSAYGK

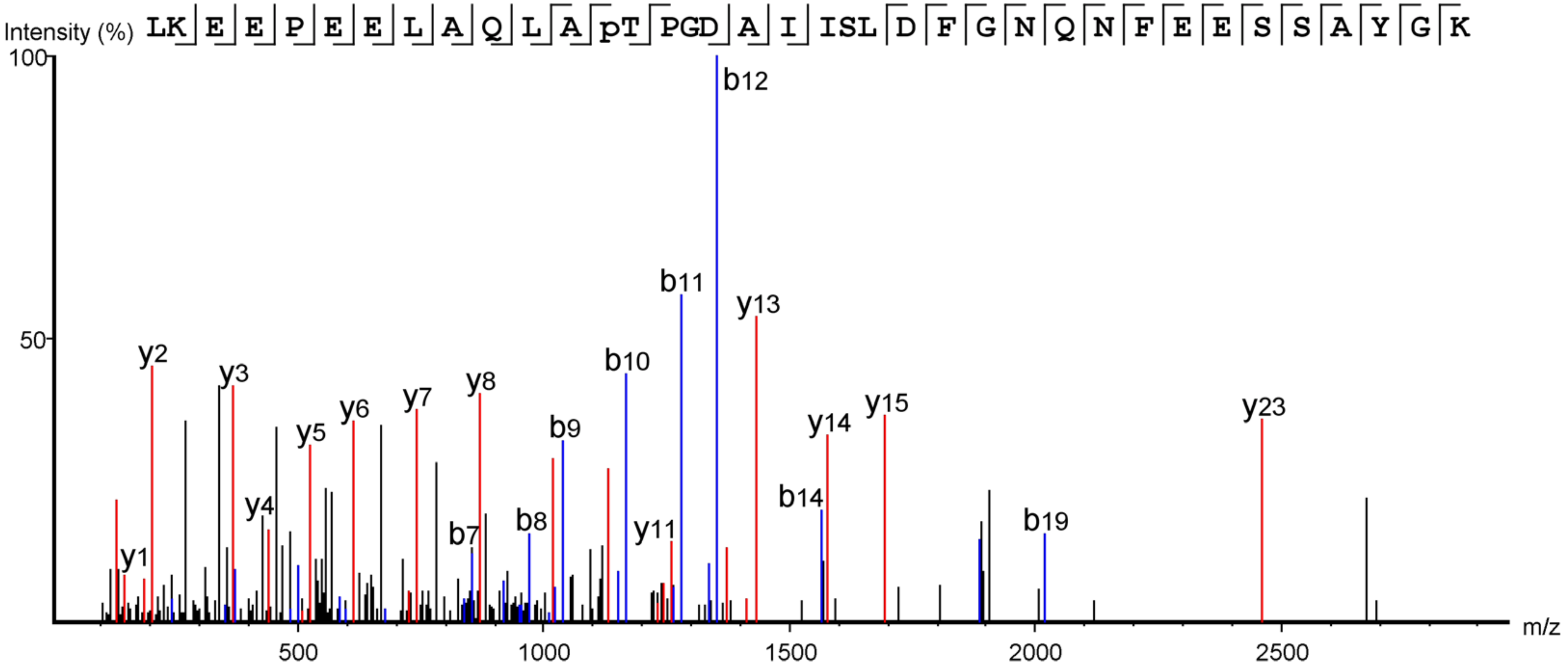

YMT121
Scan #22117

A-351: HIF-2α(393-429): LKEEPEELAQLAP(+15.99)TPGDAIISLDFGNQNFEESSAYGK

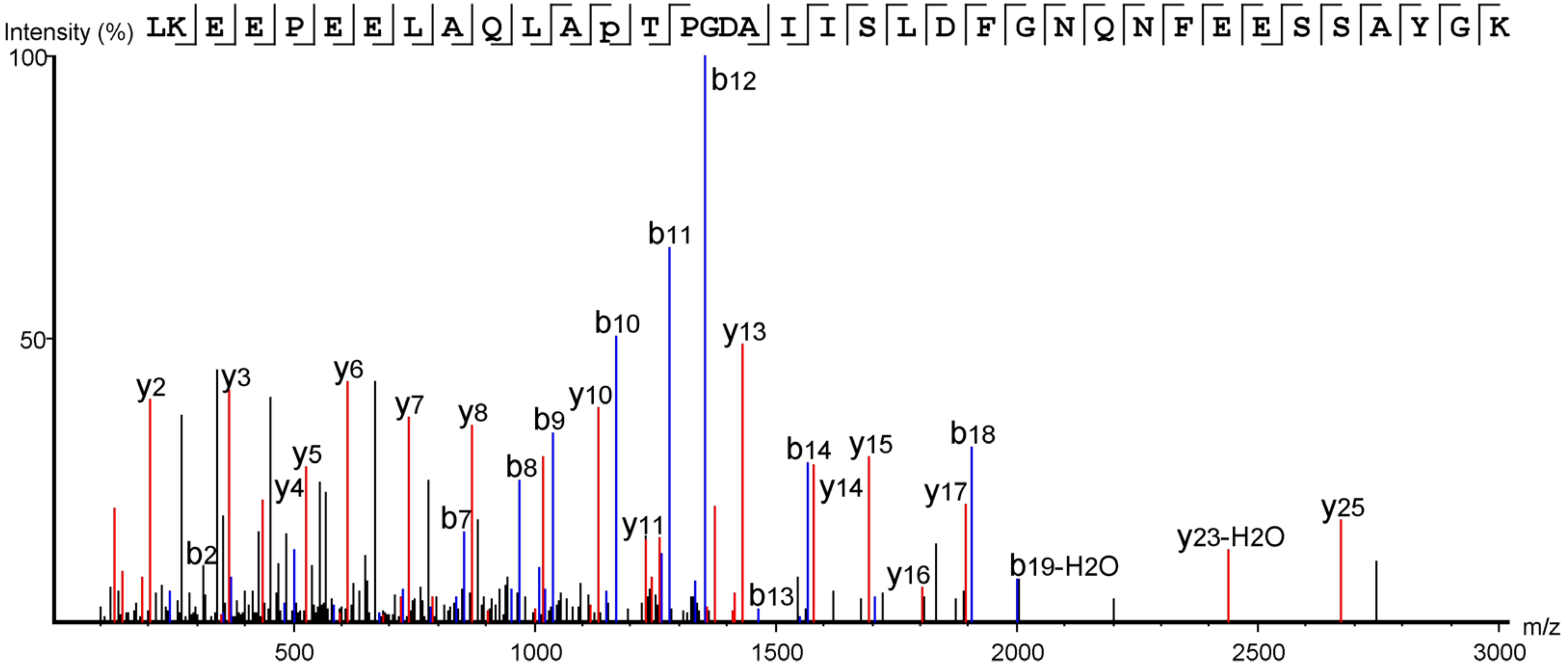

YMT122
Scan #21268

A-352: HIF-3α(486-496): DLEMLAPYISM

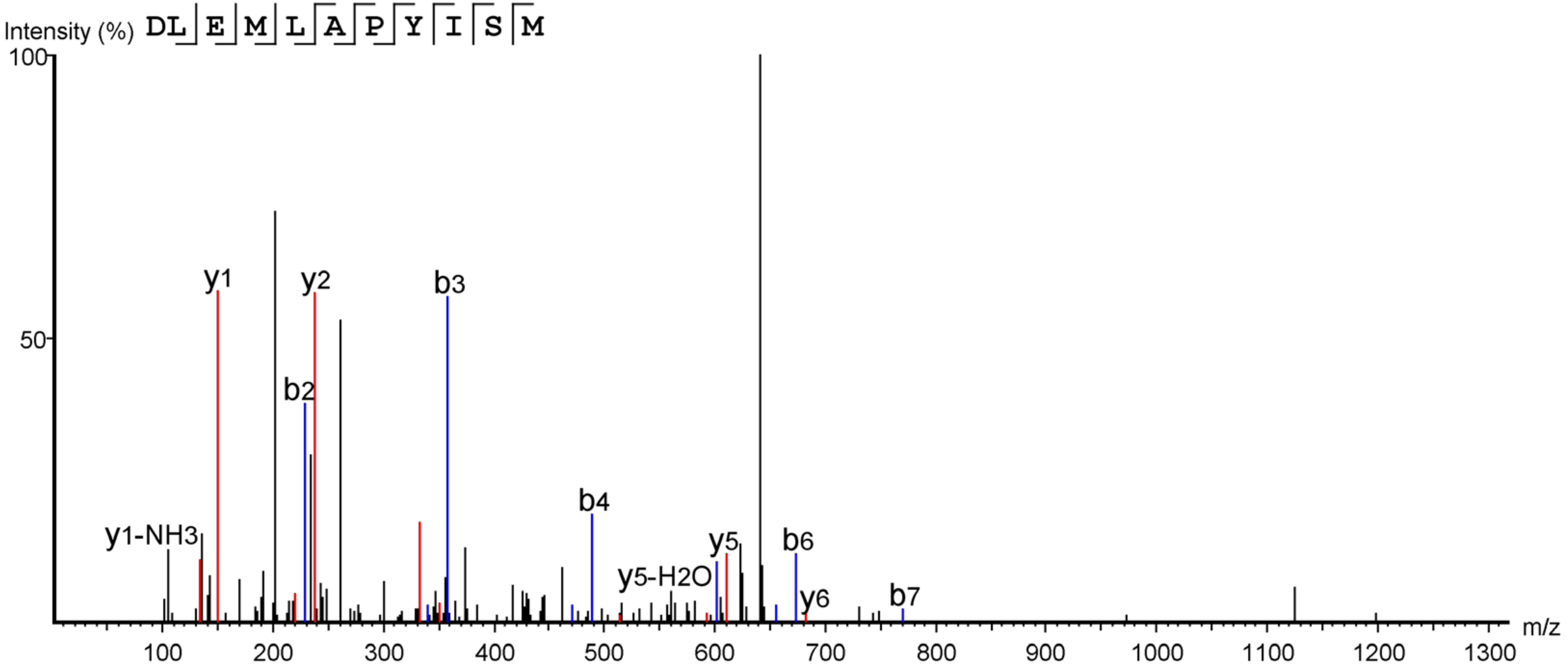

YMT123
Scan #30768

A-353: HIF-3α(486-496): DLEM(+15.99)LAPYISM

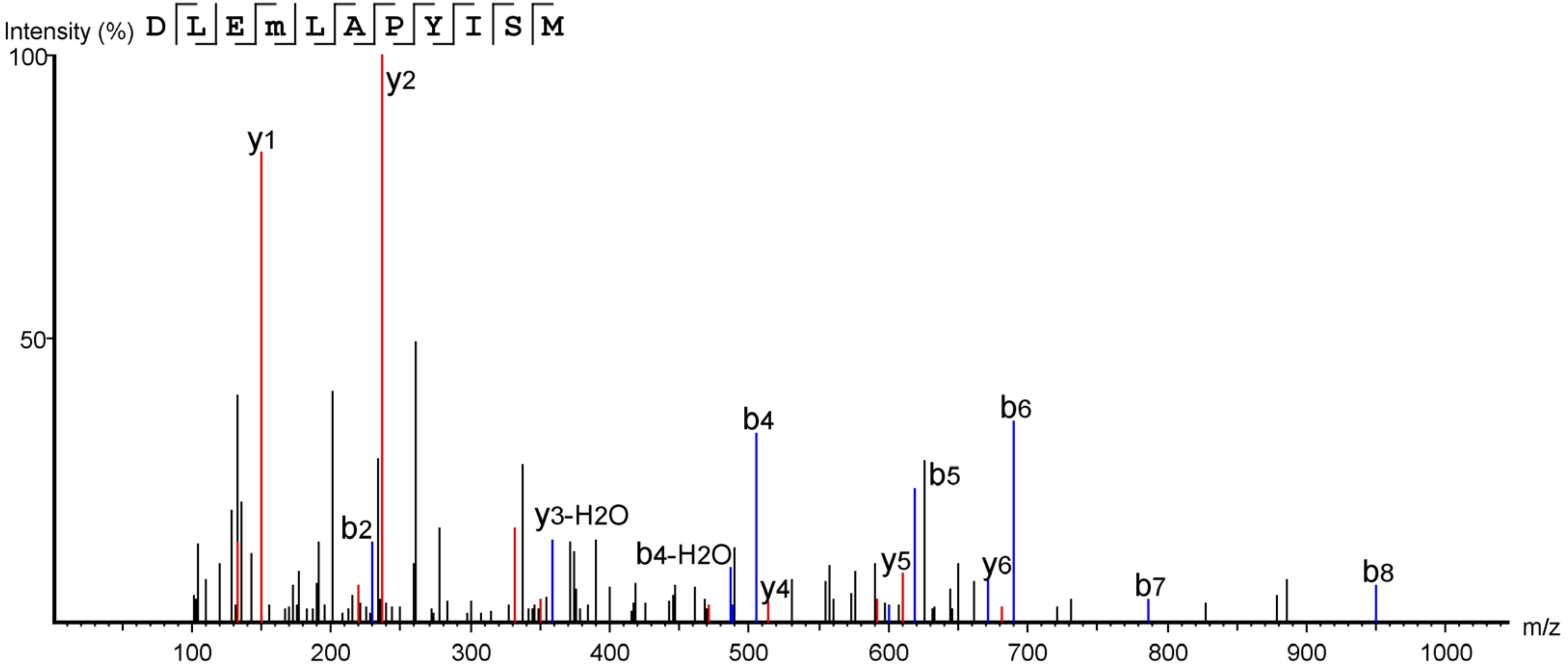

YMT123
Scan #30096

A-354: HIF-3α(486-496): DLEM(+15.99)LAPYISM

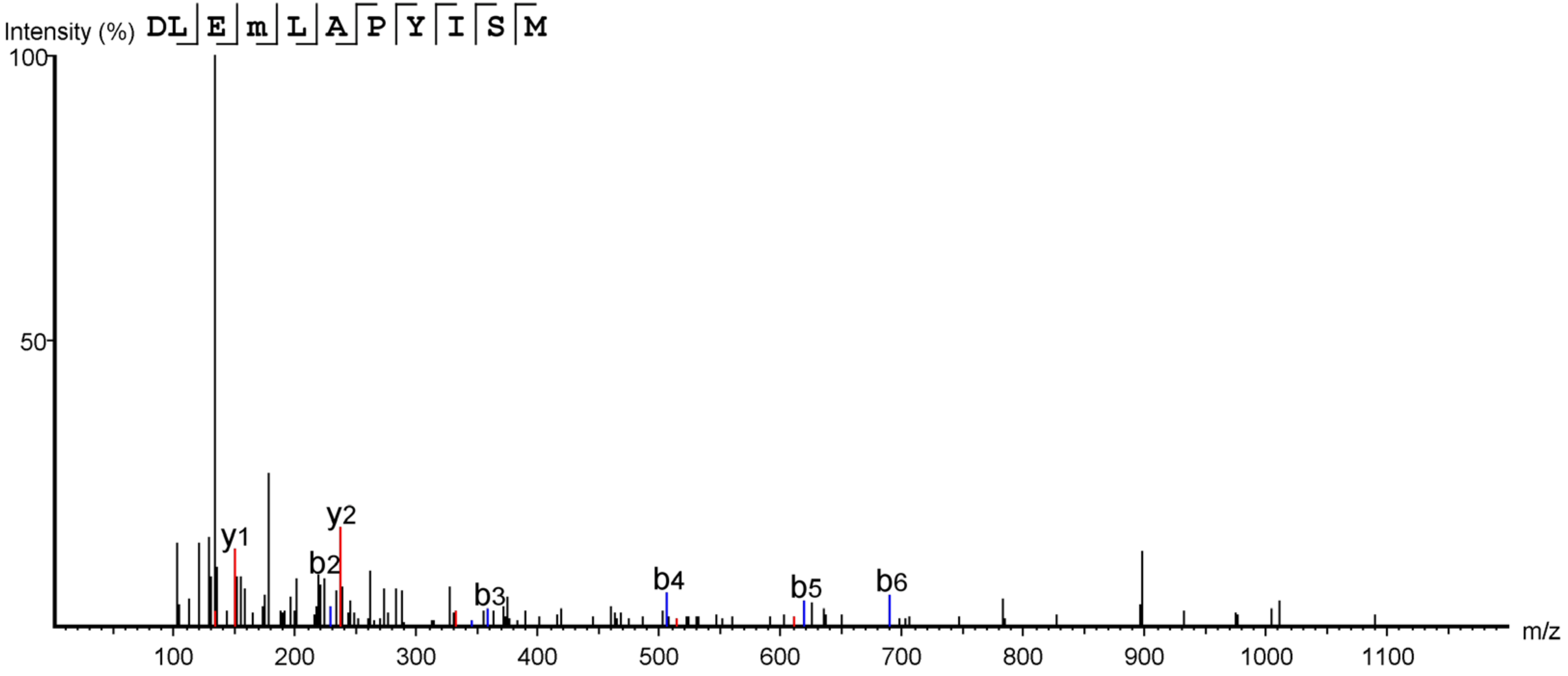

YMT125
Scan #31759

A-355: HIF-3α(486-496): DLEMLAP(+15.99)YISM

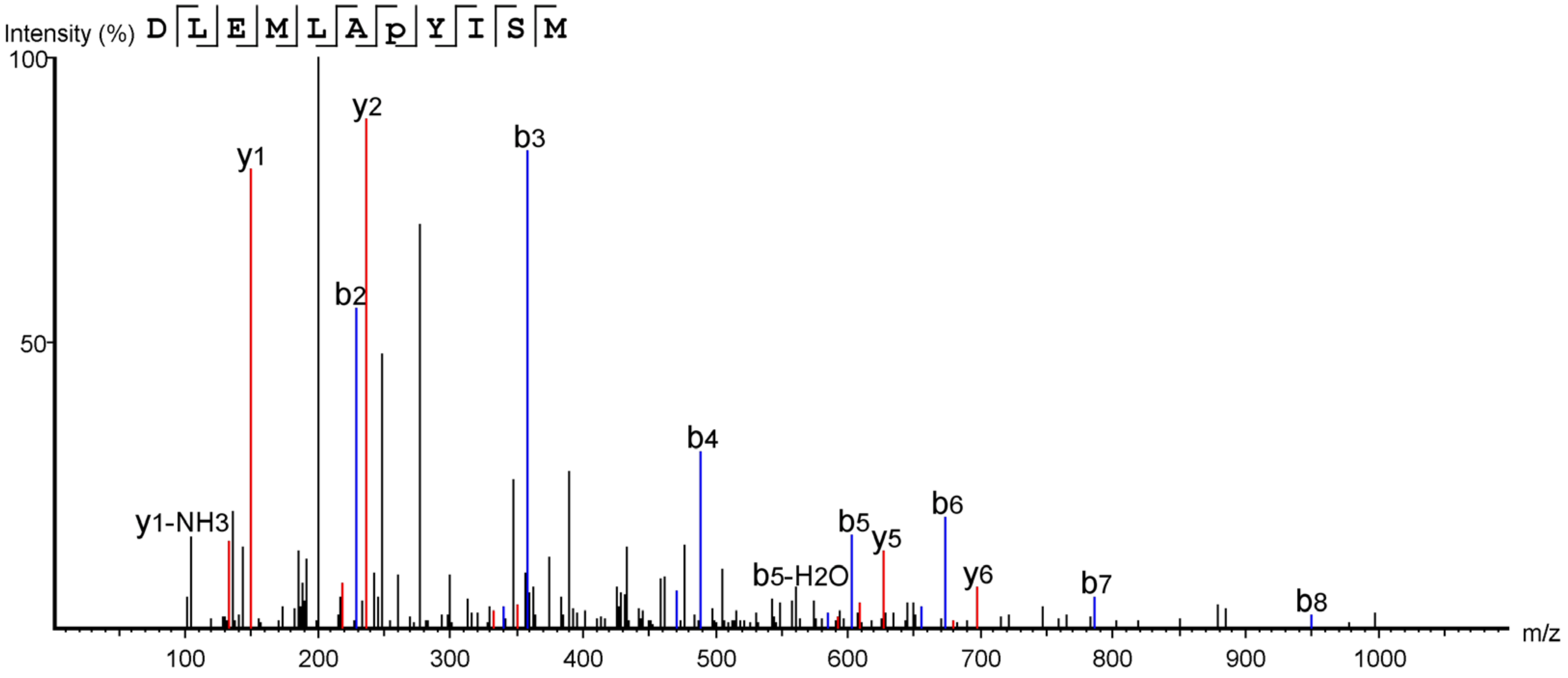

YMT124
Scan #33172

A-356: HIF-3α(486-496): DLEMLAP(+15.99)YISM

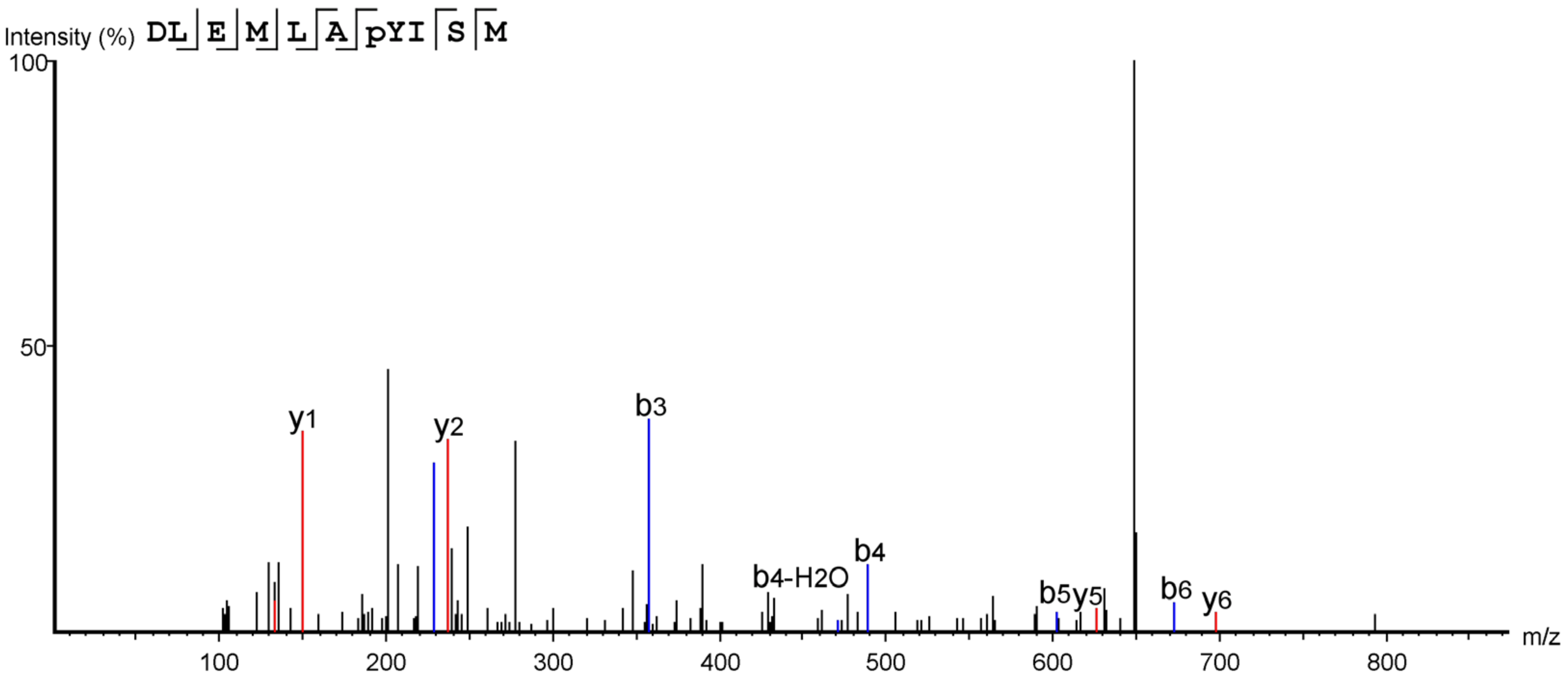

YMT125
Scan #32461

A-357: HIF-3α(486-496): DLEMLAP(+15.99)YISM

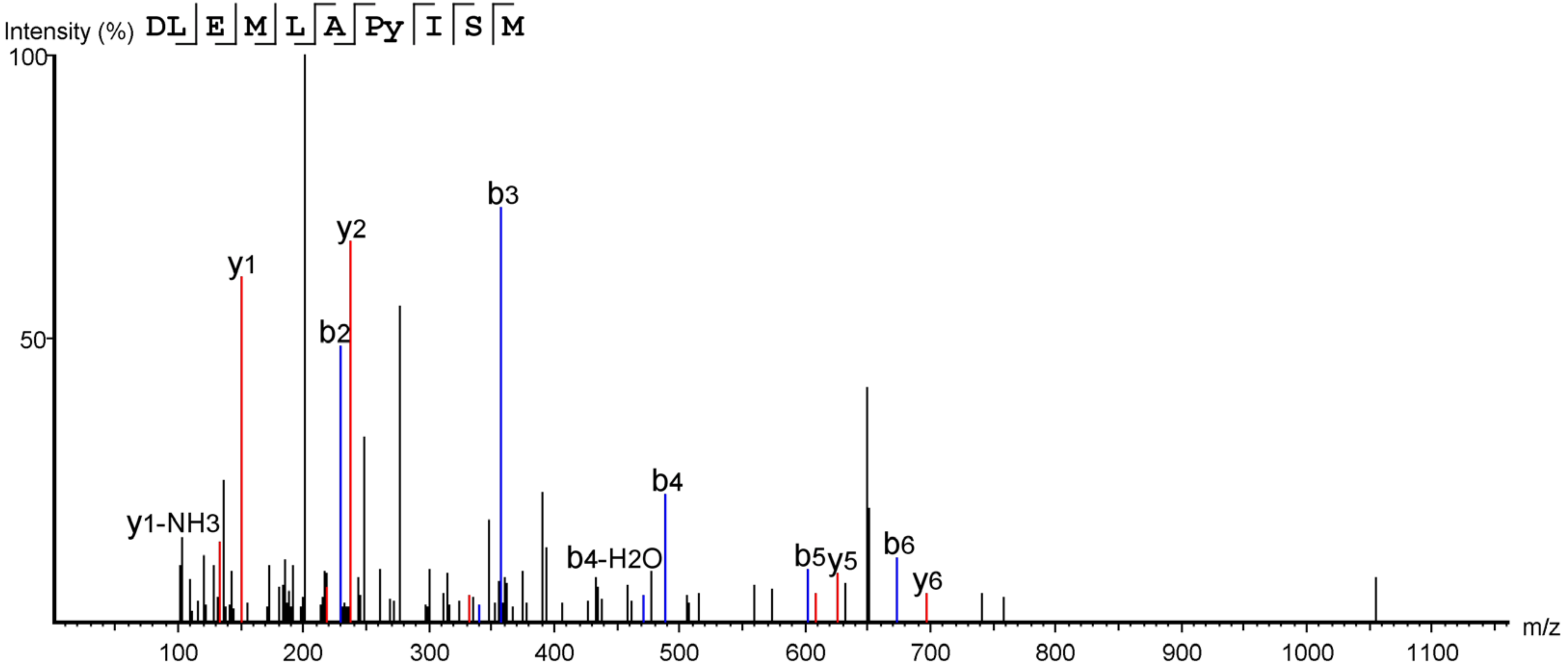

YMT126
Scan #30783

A-358: HIF-3α(486-496): DLEMLAPYISM(+15.99)

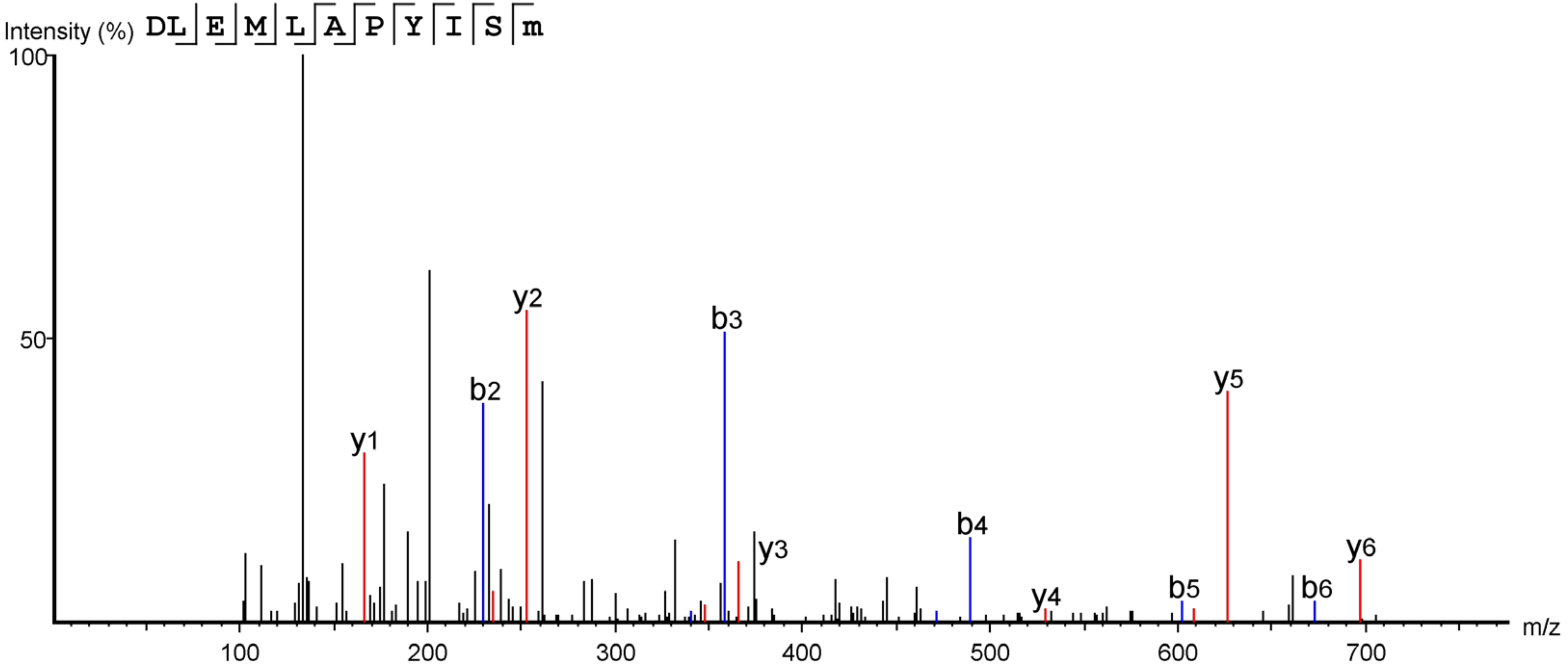

YMT123
Scan #29743

A-359: HIF-3α(486-496): DLEMLAPYISM(+15.99)

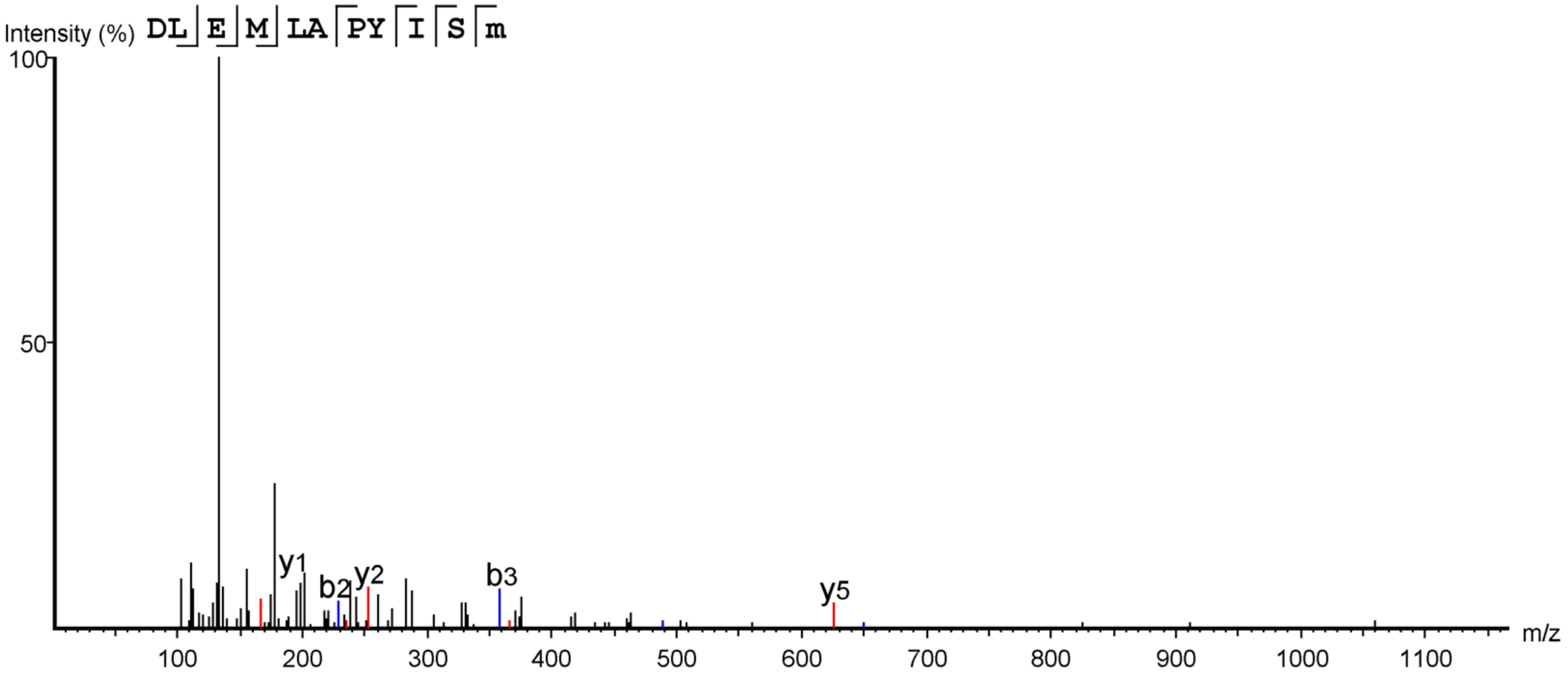

YMT124
Scan #32130

A-360: HIF-3α(486-496): DLEMLAPYISM(+15.99)

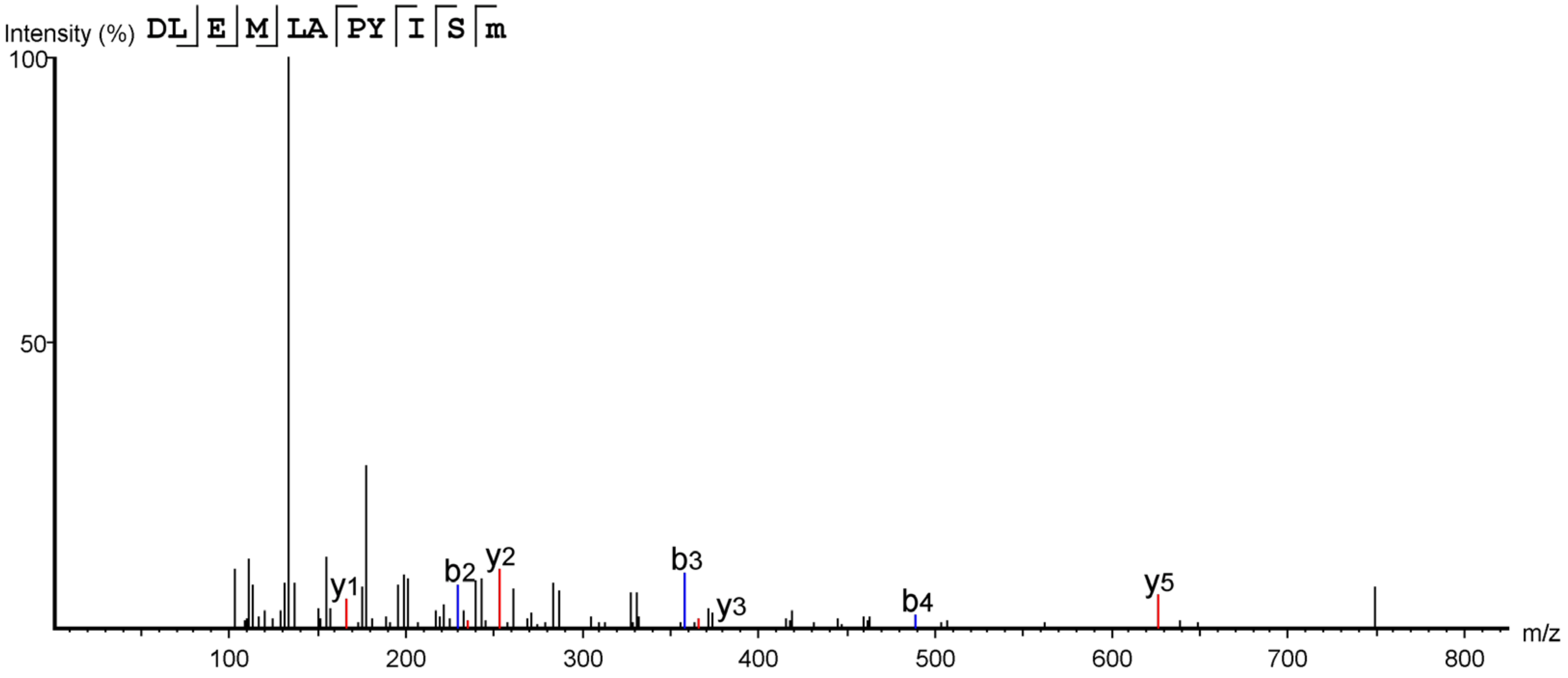

YMT125
Scan #31341

A-361: HIF-3α$_{(486-496)}$: DLEMLAPYISM(+15.99)

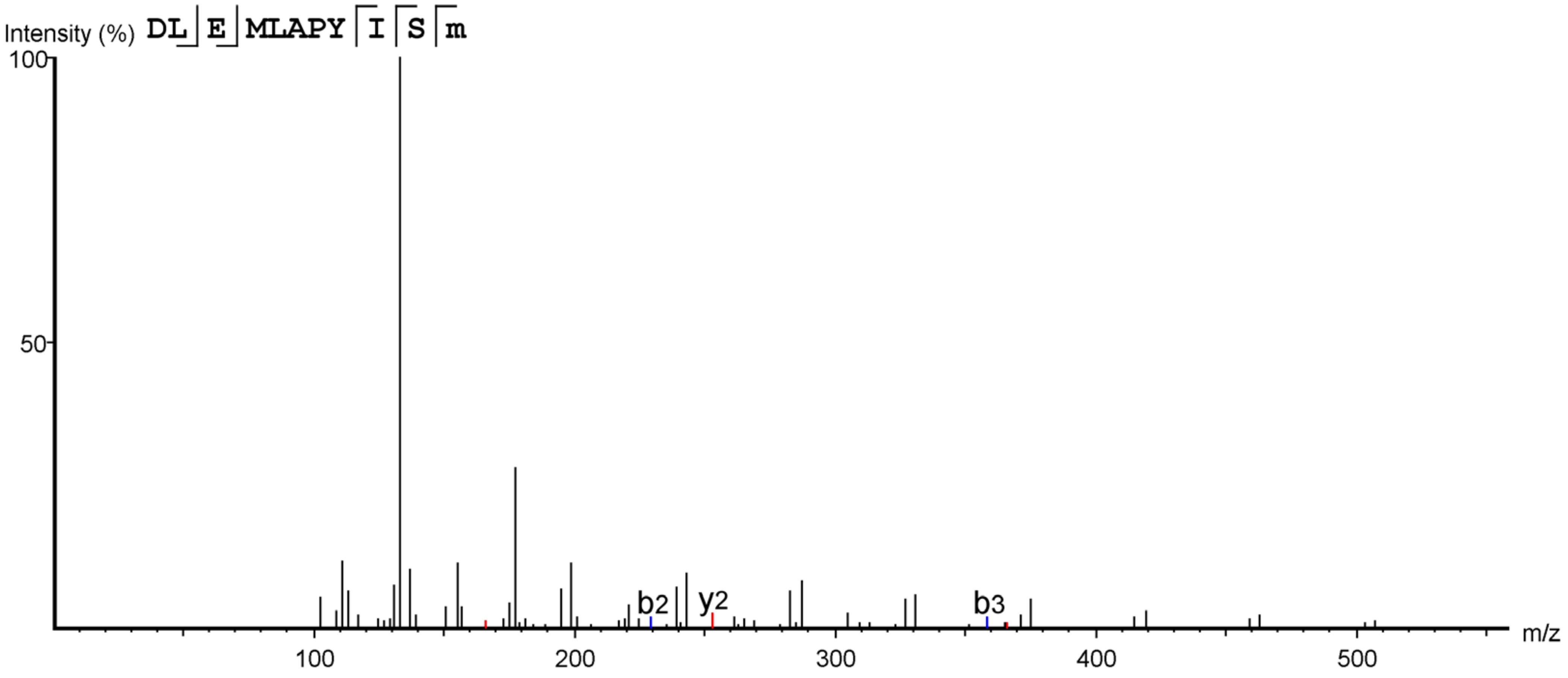

YMT126
Scan #29644

A-362: HIF-3α(486-496): DLEM(+15.99)LAP(+15.99)YISM

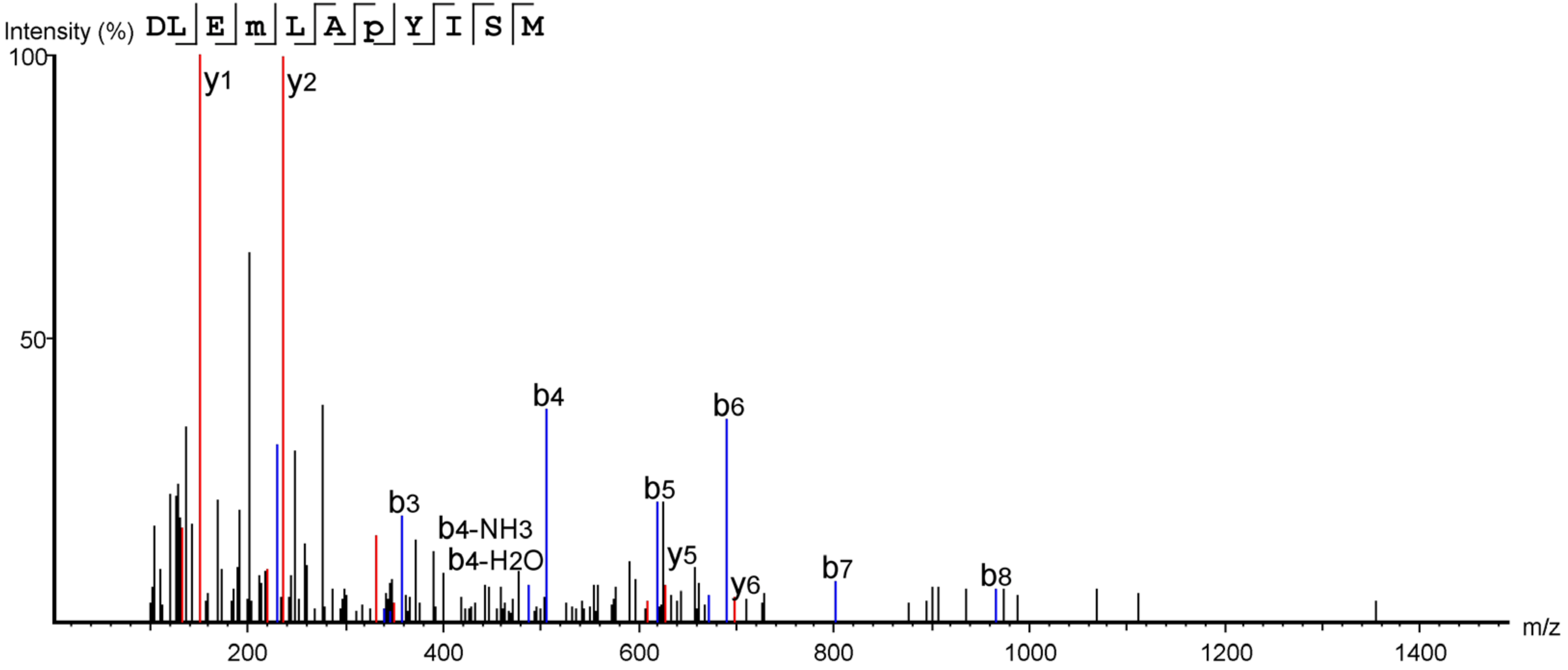

YMT123
Scan #29200

A-363: HIF-3α(486-496): DLEM(+15.99)LAP(+15.99)YISM

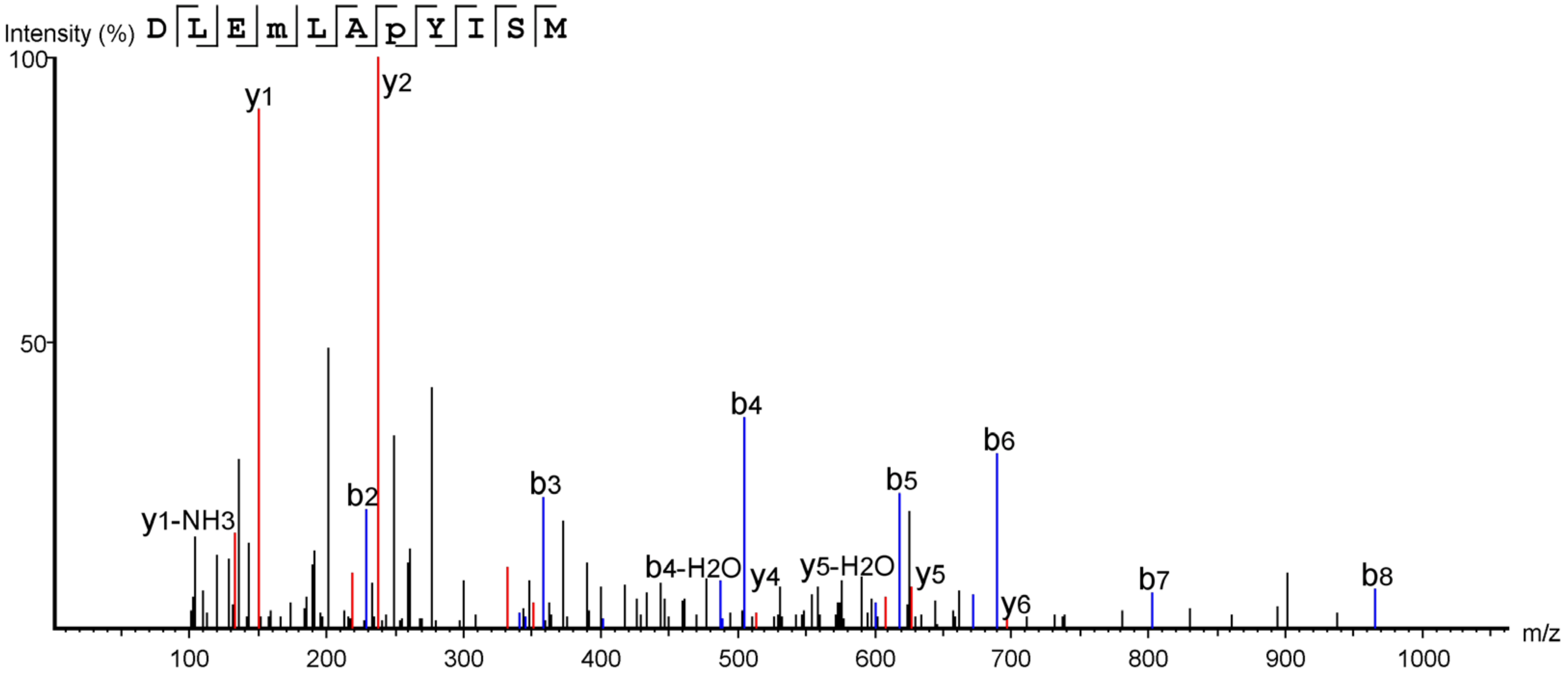

YMT124
Scan #31777

A-364: HIF-3α(486-496): DLEM(+15.99)LAP(+15.99)YISM

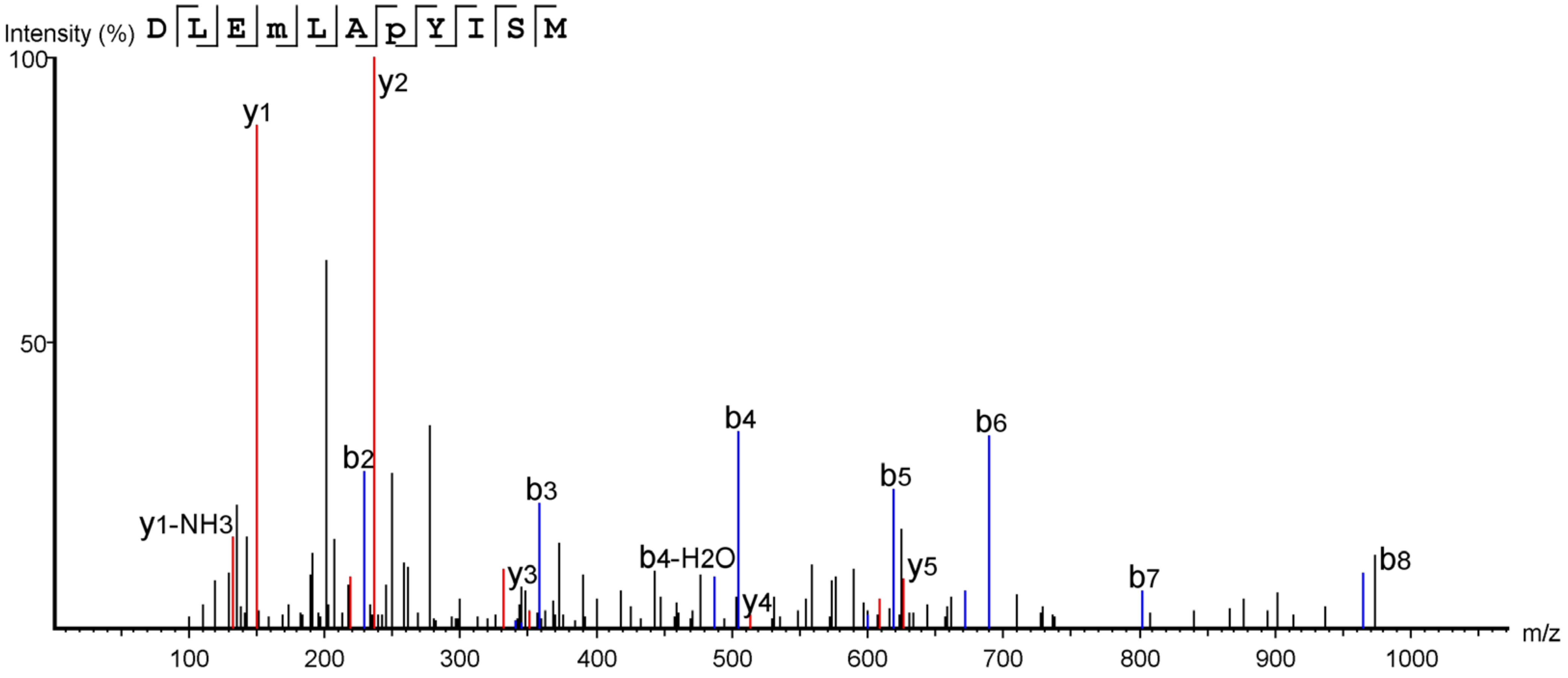

YMT125
Scan #30765

A-365: HIF-3α(486-496): DLEM(+15.99)LAP(+15.99)YISM

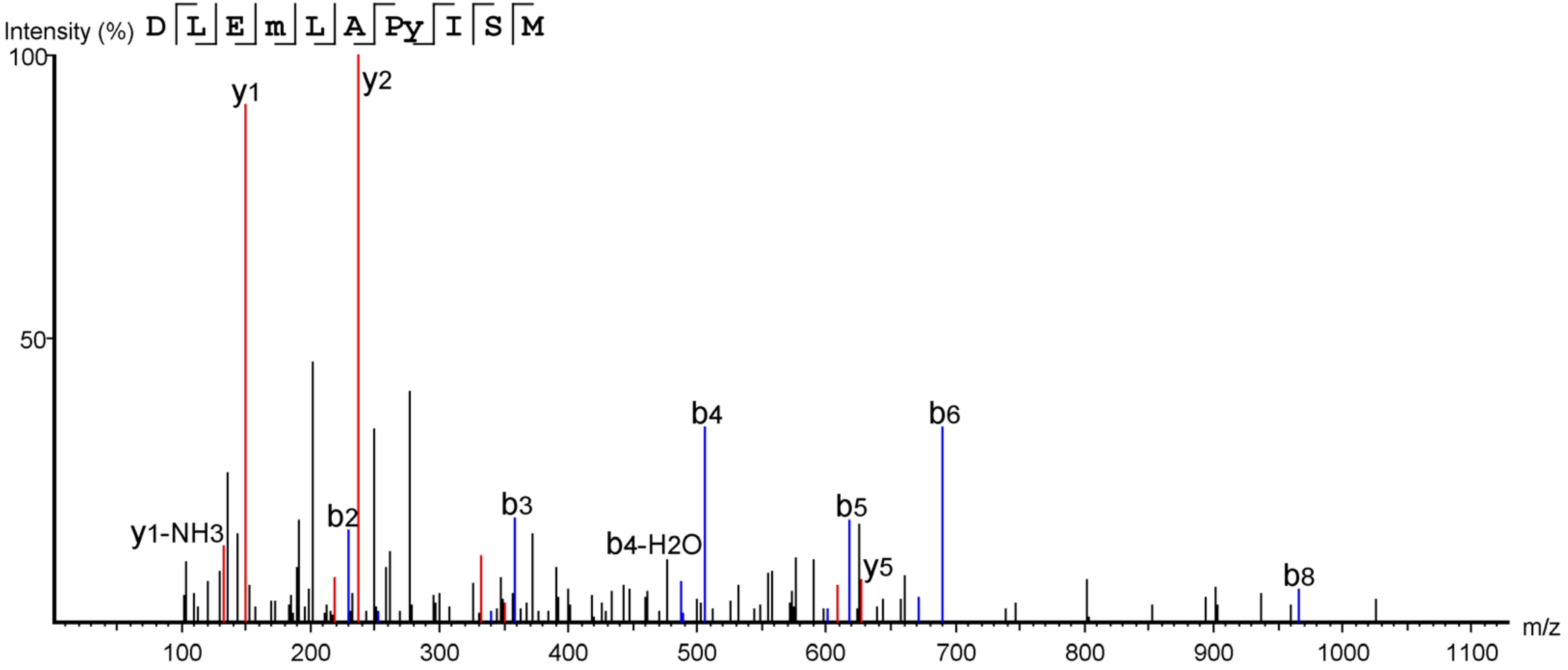

YMT126
Scan #29372

A-366: HIF-3α(486-496): DLEMLAP(+15.99)YISM(+15.99)

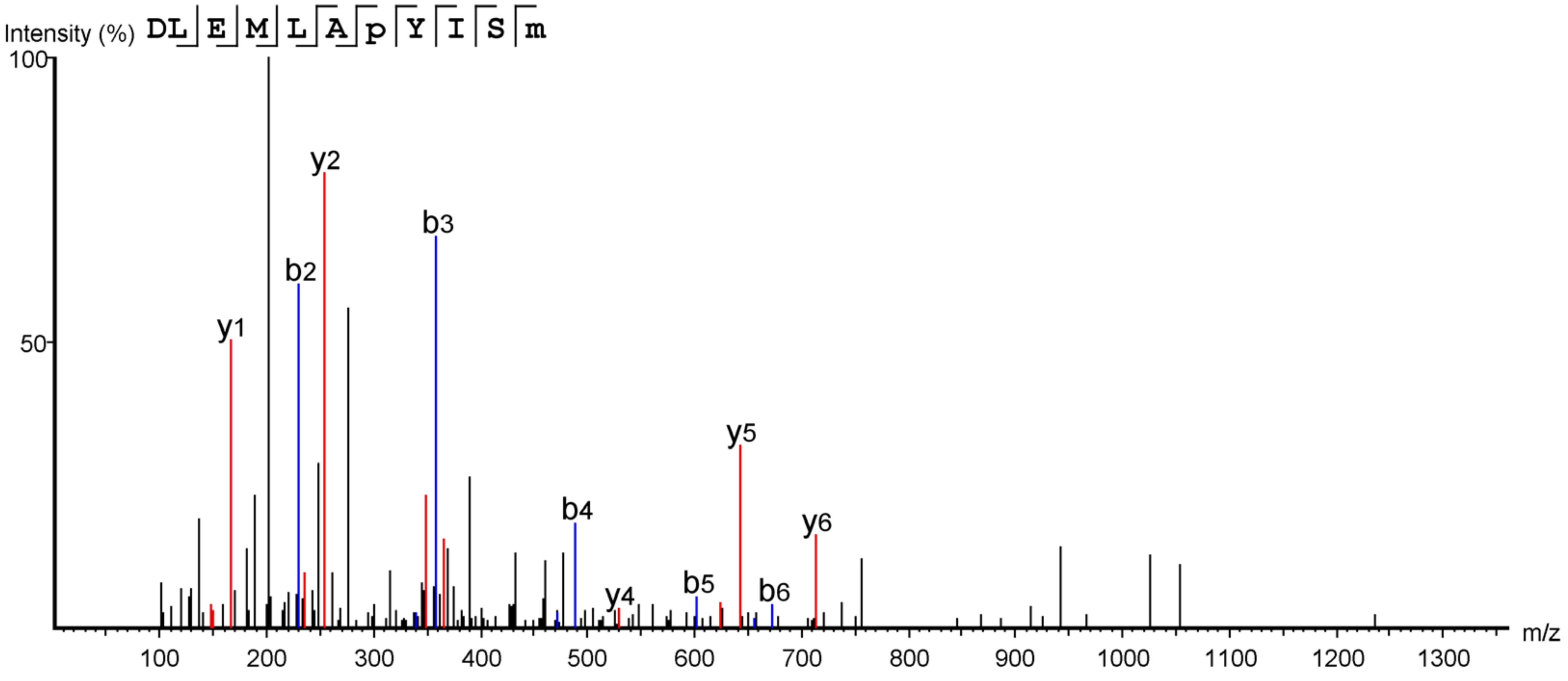

YMT123
Scan #28896

A-367: HIF-3α(486-496): DLEMLAP(+15.99)YISM(+15.99)

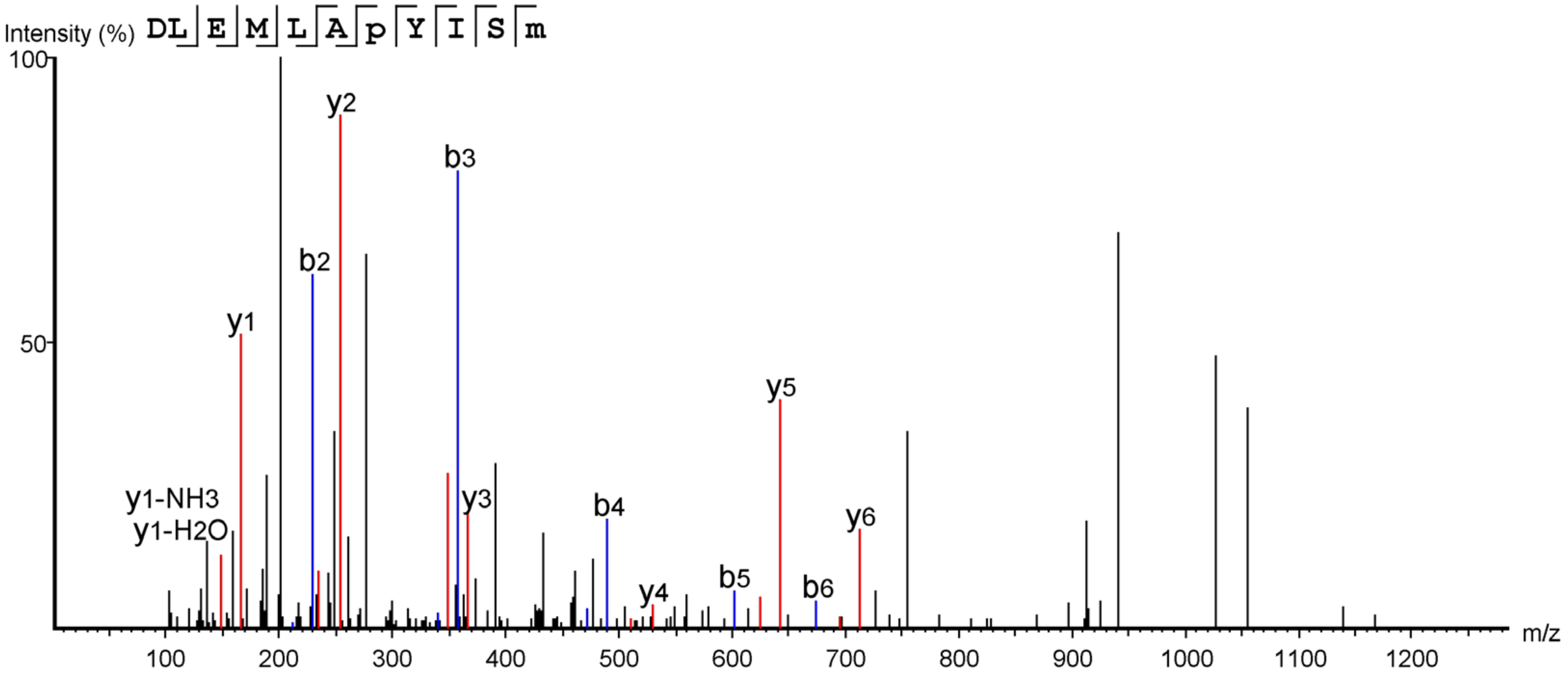

YMT125
Scan #30490

A-368: HIF-3α$_{(486-496)}$: DLEMLAP(+15.99)YISM(+15.99)

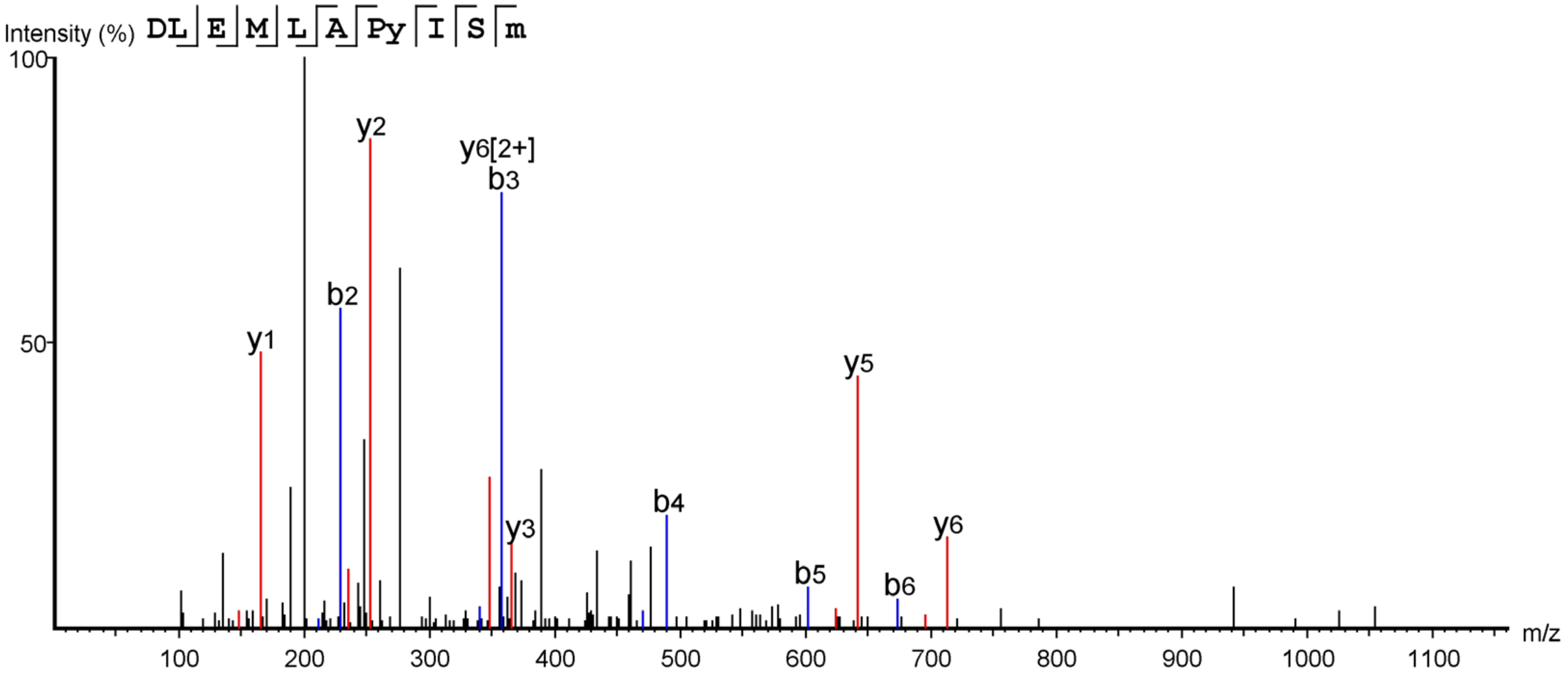

YMT126
Scan #28751

A-369: HIF-3α(486-496): DLEM(+15.99)LAPYISM(+15.99)

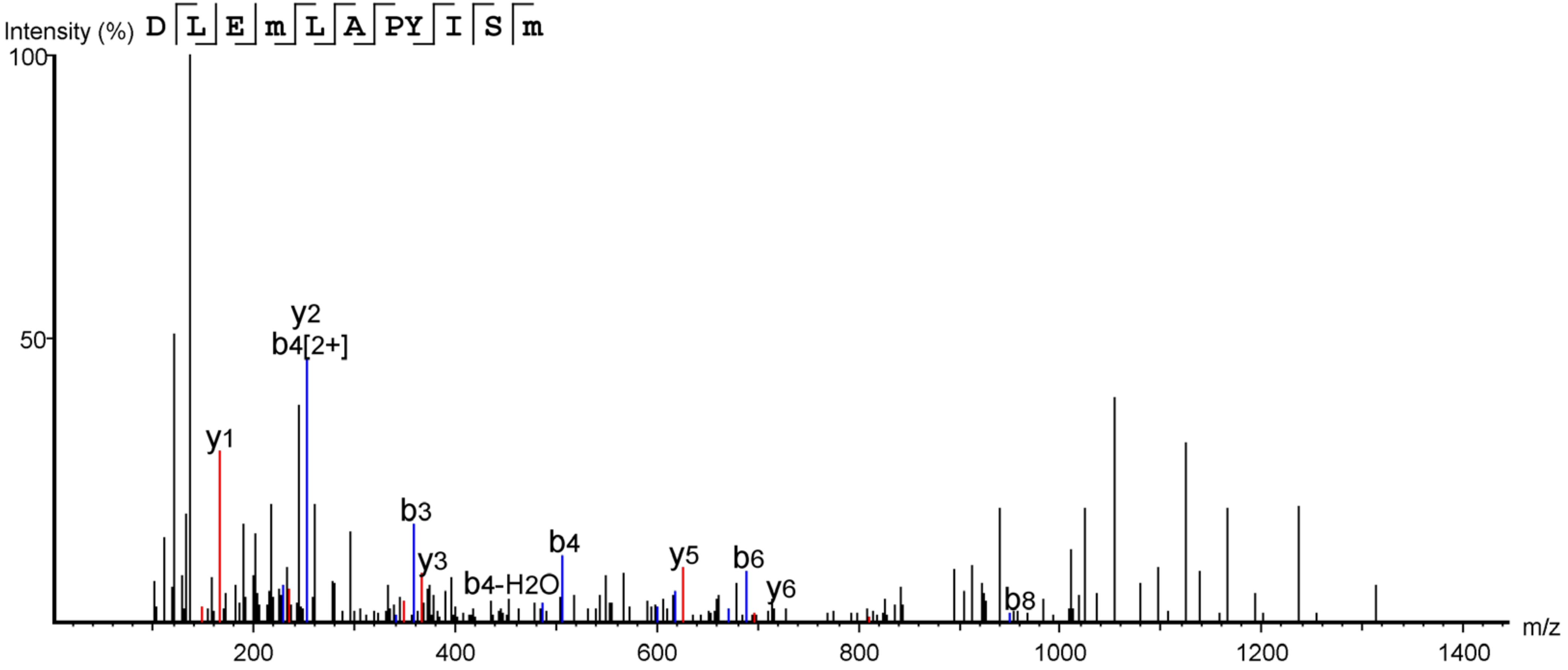

YMT123
Scan #26720

A-370: HIF-3α$_{(486\text{-}496)}$: DLEM(+15.99)LAPYISM(+15.99)

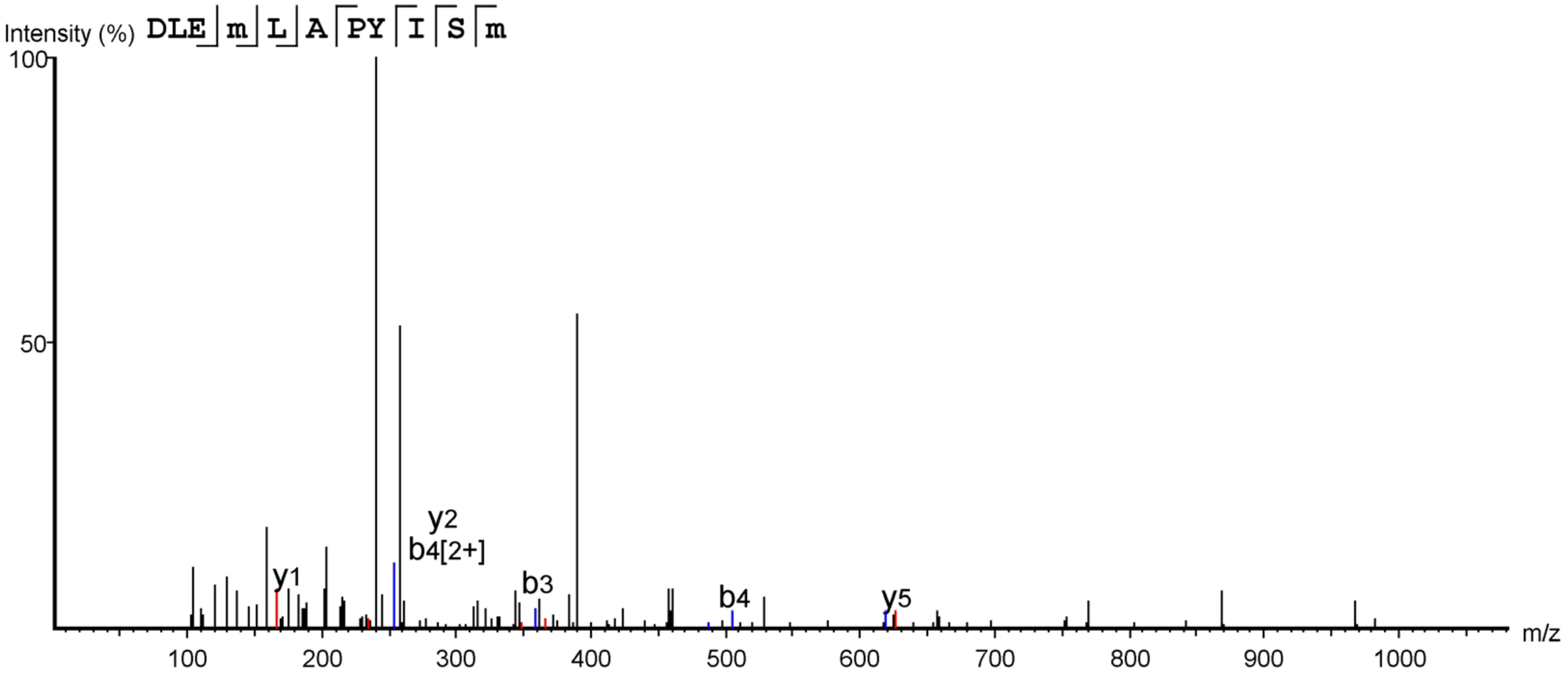

YMT126
Scan #26719

A-371: HIF-3α(486-496): DLEM(+15.99)LAP(+15.99)YISM(+15.99)

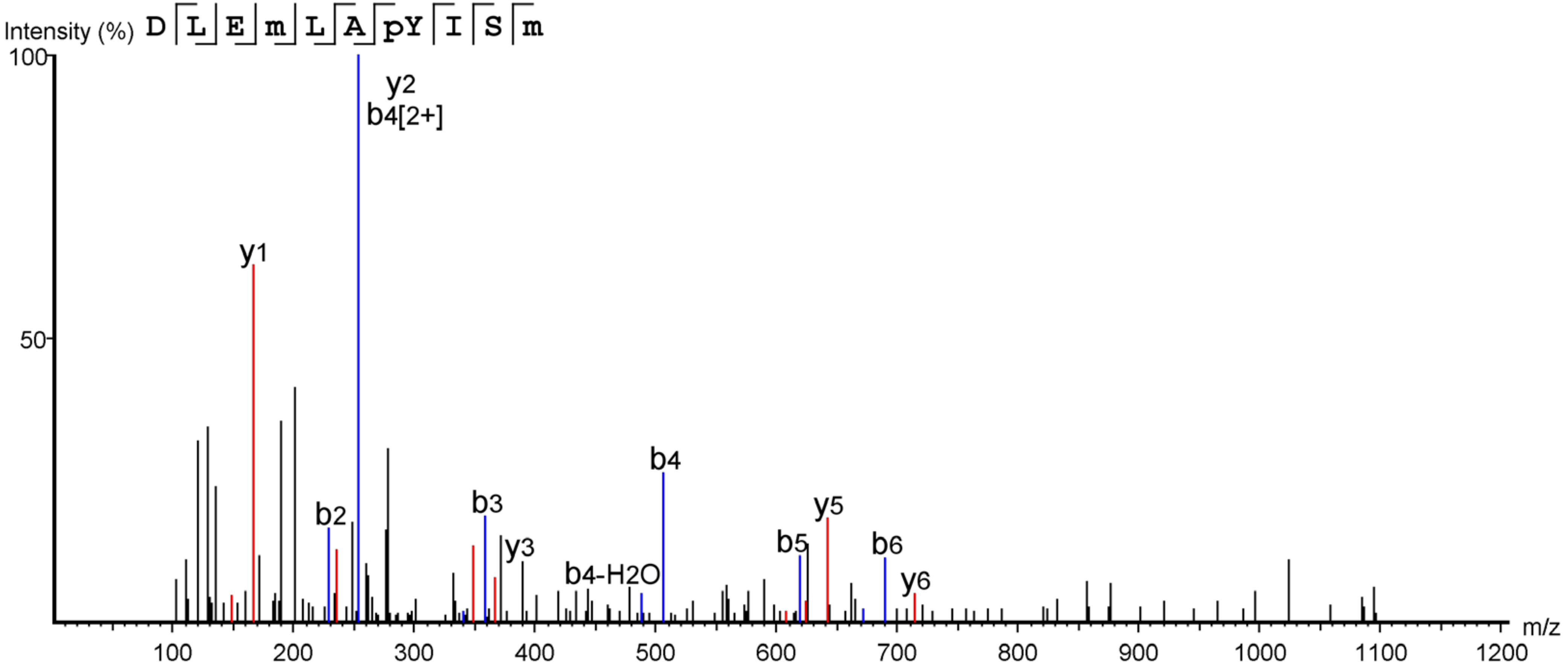

YMT123
Scan #25536

A-372: HIF-3α$_{(486-496)}$: DLEM(+15.99)LAP(+15.99)YISM(+15.99)

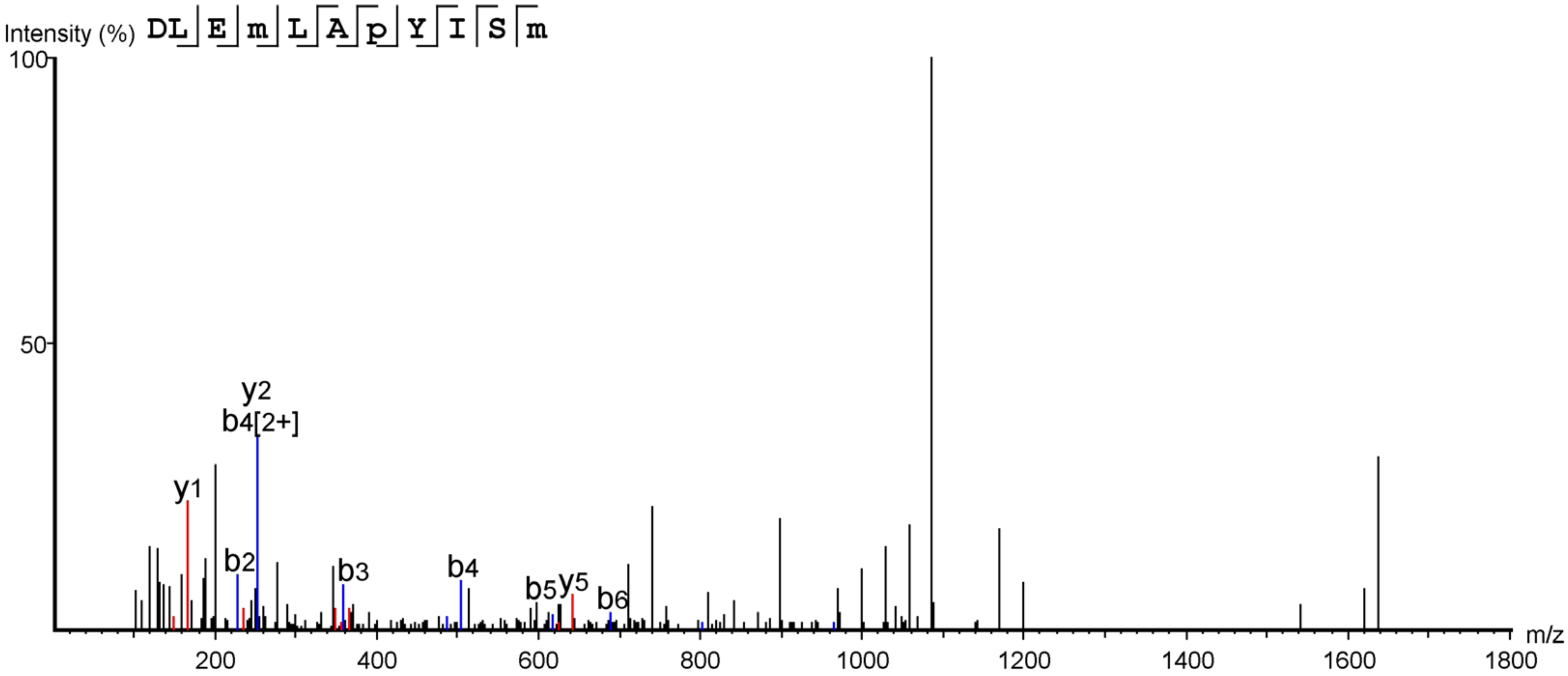

YMT124

Scan #28068

A-373: HIF-3α(486-496): DLEM(+15.99)LAP(+15.99)YISM(+15.99)

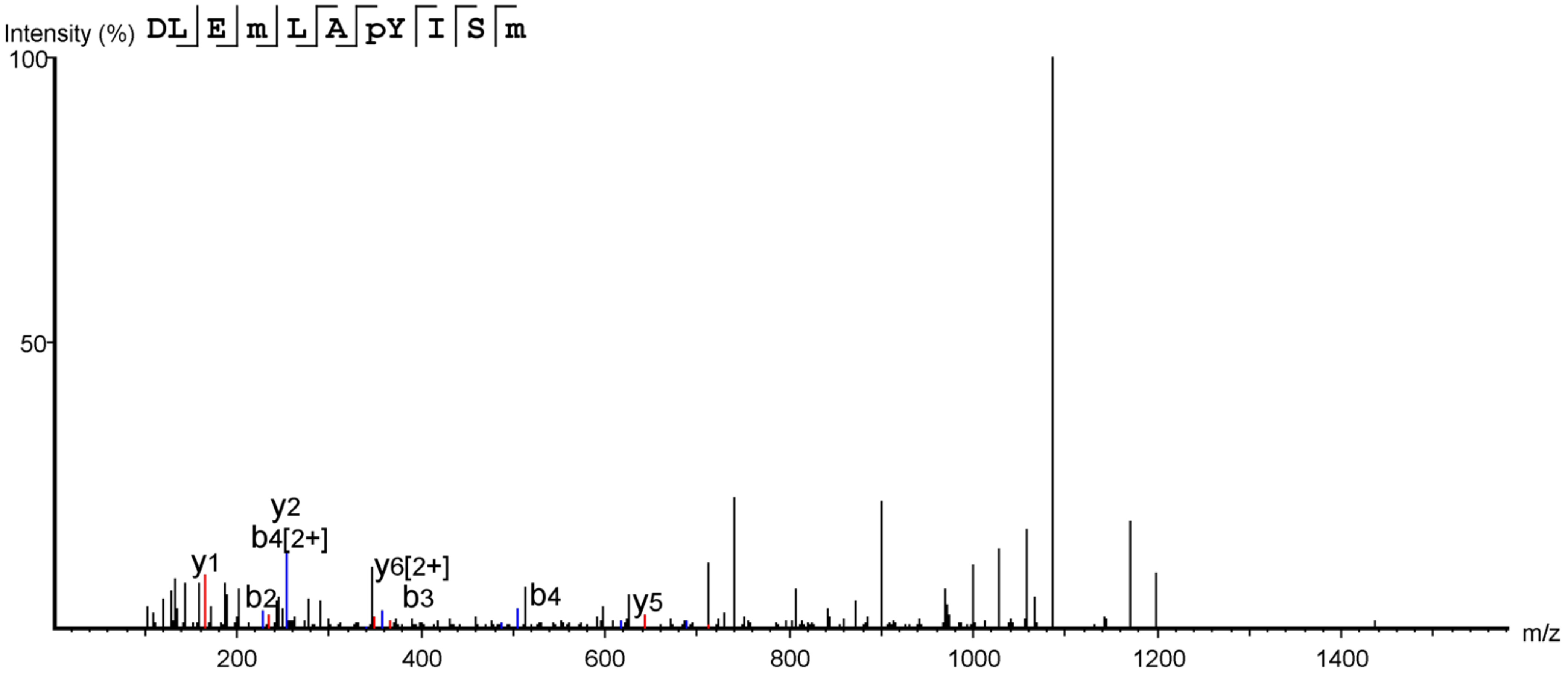

YMT125
Scan #27386

A-374: HIF-3α(486-496): DLEM(+15.99)LAP(+15.99)YISM(+15.99)

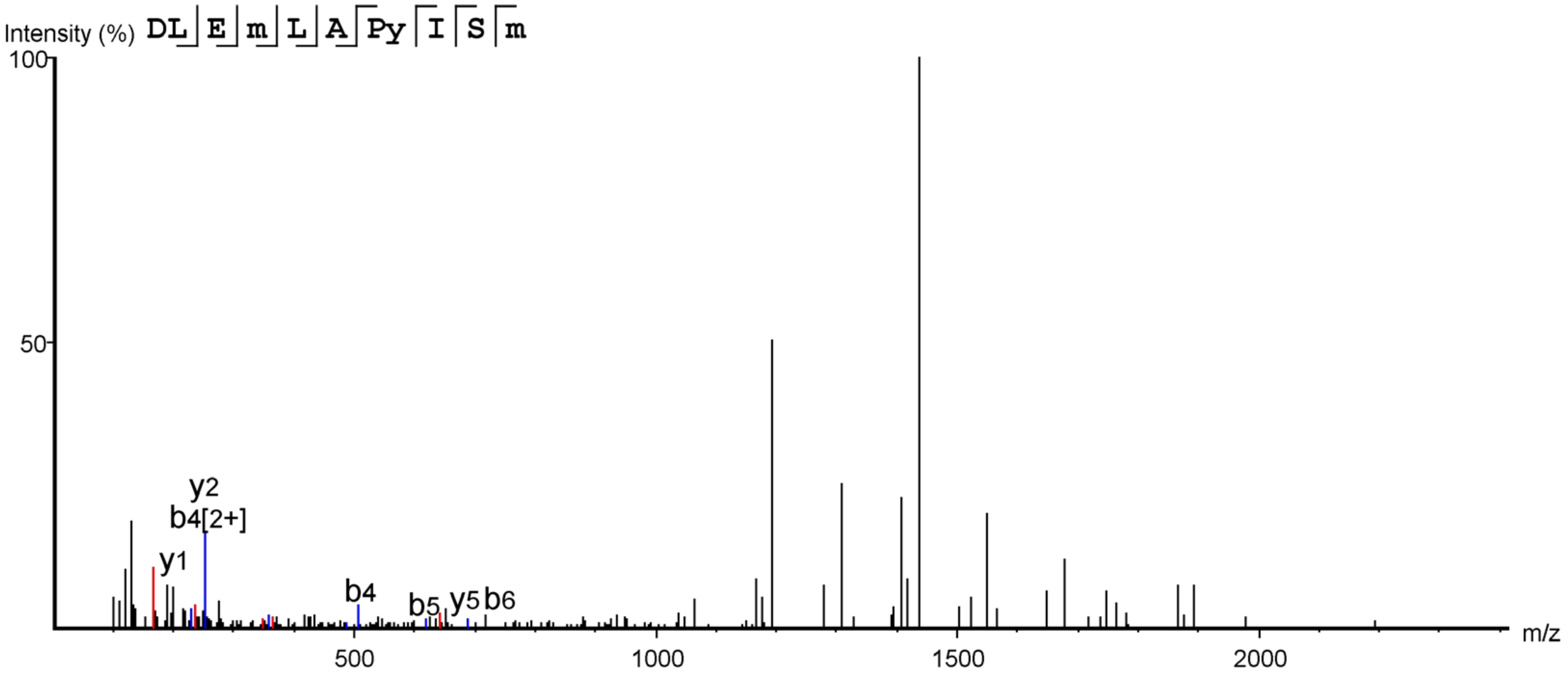

YMT126
Scan #25580
