# Supplementary material for: Lack of activity of recombinant HIF prolyl hydroxylases (PHDs) on reported non-HIF substrates
Source: eLife. 2019 Sep 10;8:e46490. doi: 10.7554/eLife.46490 (PMC6739866; doi:10.7554/eLife.46490)
Supplement: Supplementary file 2. — HIF-1α hydroxylation assays were performed in parallel with alternative substrates to confirm the activity of recombinant PHD enzyme preparations. The relationship between each HIF1A hydroxylation dataset and corresponding alternative substrate assay is indicated above each figure. Full-length HIF-1α protein produced by IVTT was incubated in the absence (Control) or presence of the indicated (PHD) enzyme and digested with trypsin. Endogenous PHD activity in HeLa cell lysate gave rise to a basal (control) level of hydroxylation that markedly increased upon addition of recombinant PHD. To facilitate quantitation of P564ox we used a variant of HIF1A in which adjacent methionine residues that are prone to de novo oxidation were substituted to alanine (HIF1A*: M561A, M568A). This modified form of HIF1A is more amenable to LC-MS based quantitation (peak intensities are not reduced as a consequence of methionine oxidation) and has no appreciable effect on the rate of PHD-dependent catalysis (Tian et al., 2011). Detection of P564 peptide ions was robust across replicate assays and relative quantitation data are presented for all HIF-1α hydroxylation assays. Detection of P402 peptide ions was more variable, requiring an additional (i.e., non-specific) cleavage by trypsin in order to monitor hydroxylation at this site. Relative quantitation of P564ox is presented in panel A, as XIC of m/z 1590.7540 and 1598.7515 corresponding to unoxidised (pink) and P564ox (blue) forms of HIF1A*(548-575). Where present, semi-tryptic peptides encompassing P402 were detected as either HIF1A*(392-403) or HIF1A*(392-411). Panel B shows XIC for precursor ion masses corresponding to unoxidised (pink; (i) m/z 619.8532, (ii) m/z 1005.0619) and P402ox (blue; (i) 627.8506, (ii) 1013.0592) forms of related (i) HIF1A*(392-403) or (ii) HIF1A*(392-411) peptides. Quantitative data for observed species are presented as pie charts with XIC data. Assignment and quantitation data are tabulated below each panel [file elife-46490-supp2.pdf]

Supplementary File 2

B-01: **HIF Control A**

Alt Substrate: NDRG3

HIF1A*$_{(548-575)}$: NPFSTQDTDLDLEALAPYIPADDDFQLR

--- m/z 1590.7540 Unoxidised

···· m/z 1598.7515 P564ox

A

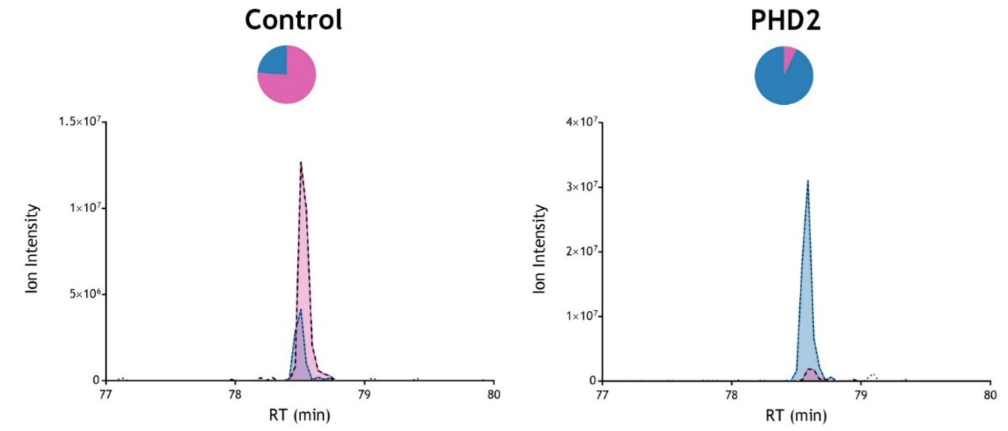

			Assignment					Quantitation			
	Species	Condition	-10 lgP	RT	MSMS	AScore	Expected MH^{+2}	MH^{+2}	RT Win.	Area	RA (%)
0	HIF1A*(548-575)	Control	45.42	78.52	A-013	N/A	1590.7540	1590.7535	78.4-78.8	7.0E+07	76.0
		PHD2	48.20	78.60	A-014	N/A		1590.7527	78.5-78.8	1.2E+07	7.0
16 Da	P564ox: HIF1A*(548-575)	Control	64.66	78.48	A-015	P564: 0	1598.7515	1598.7501	78.4-78.7	2.2E+07	24.0
		PHD2	59.22	78.56	A-016	P564: 0		1598.7518	78.5-78.8	1.6E+08	93.0

HIF1A*$_{(392-403)}$: KEPDALTLLAPA

--- m/z 619.8532 Unoxidised

···· m/z 627.8506 P402ox

B

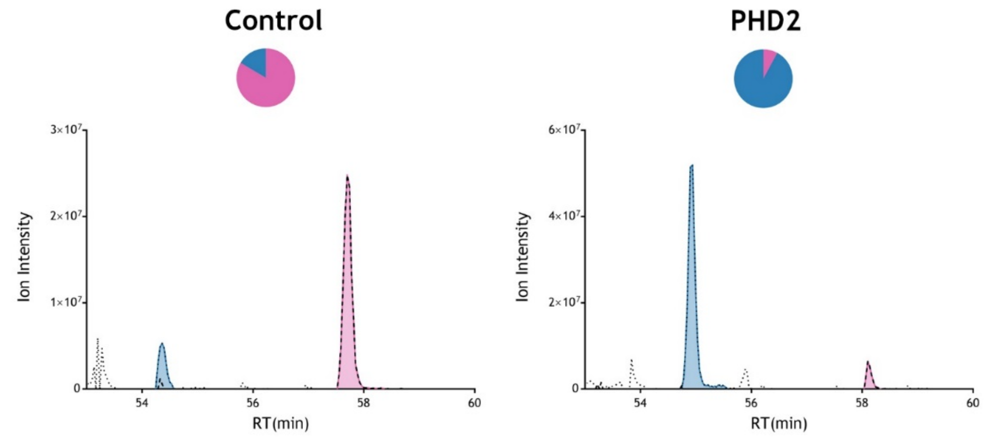

			Assignment					Quantitation			
	Species	Condition	-10 lgP	RT	MSMS	AScore	Expected MH^{+2}	MH^{+2}	RT Win.	Area	RA (%)
0	HIF1A*(392-403)	Control	16.13	57.75	A-017	N/A	619.8532	619.8523	57.5-58.4	2.6E+08	83.5
		PHD2	18.70	58.12	A-018	N/A		619.8512	58.0-58.3	4.2E+07	7.9
16 Da	P402ox: HIF1A*(392-403)	Control	18.68	54.34	A-019	P402: 89.02	627.8506	627.8498	54.3-54.6	5.2E+07	16.5
		PHD2	19.42	54.98	A-020	P402: 89.02		627.8501	54.7-55.5	4.9E+08	92.1

B-02: HIF Control B
Alt Substrate: SPRY2 & TP53 (PHD1); EPOR & MAPK6 (PHD3)

HIF1A*$_{(548-575)}$: NPFSTQDTDLDLEALAPYIPADDDFQLR

A

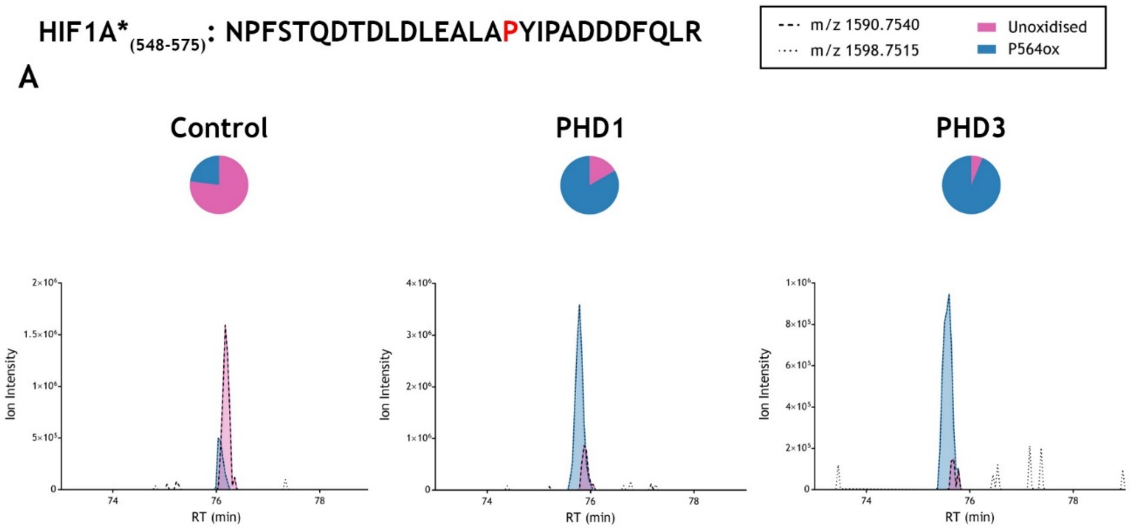

			Assignment					Quantitation			
	Species	Condition	-10 lgP	RT	MSMS	AScore	Expected MH^{+2}	MH^{+2}	RT Win.	Area	RA (%)
0	HIF1A*(548-575)	Control	54.33	76.14	A-021	N/A	1590.7540	1590.7566	76.0-76.4	1.4E+07	76.9
		PHD1	33.32	75.84	A-022	N/A		1590.7517	75.8-76.1	7.3E+06	16.6
		PHD3	ND					1590.7514	75.6-75.7	8.1E+05	6.2
16 Da	P564ox: HIF1A*(548-575)	Control	43.64	76.06	A-023	P564: 0	1598.7515	1598.7590	76.0-76.3	4.17E+06	23.1
		PHD1	43.17	75.84	A-024	P564: 0		1598.7491	75.6-76.1	3.66E+07	83.4
		PHD3	38.54	75.53	A-025	P564: 0		1598.7510	75.3-75.9	1.22E+07	93.8

HIF1A*$_{(392-403)}$: KEPDALTLLAPA

B

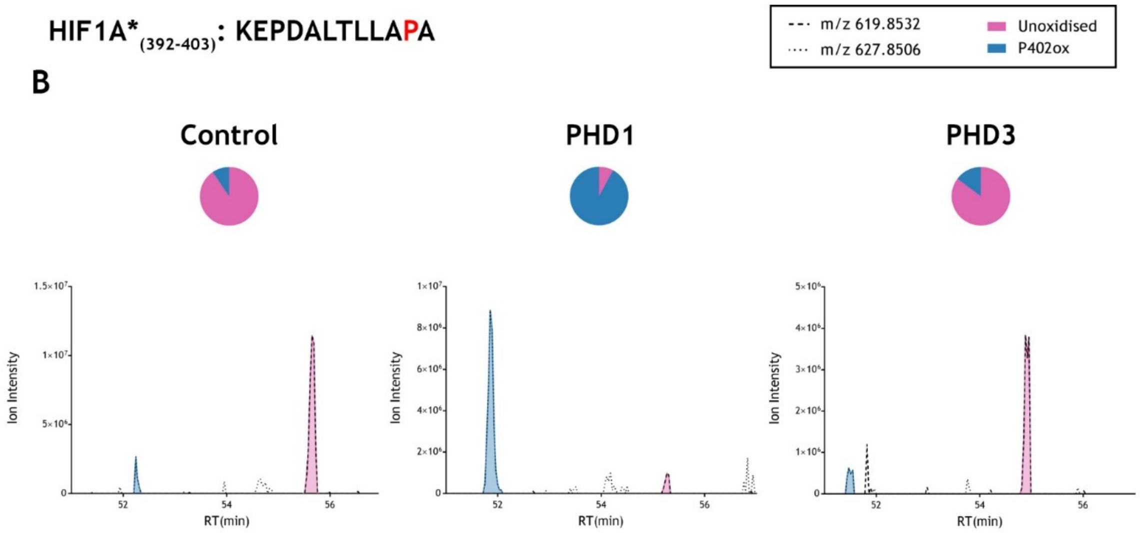

			Assignment					Quantitation			
	Species	Condition	-10 lgP	RT	MSMS	AScore	Expected MH^{+2}	MH^{+2}	RT Win.	Area	RA (%)
0	HIF1A*(392-403)	Control	16.96	55.61	A-026	N/A	619.8532	619.8524	55.5-55.7	8.3E+07	90.5
		PHD1	ND					619.8528	55.2-55.3	5.5E+06	7.9
		PHD3	18.11	54.94	A-027	N/A		619.8519	54.8-54.9	2.6E+07	85.2
16 Da	P402ox: HIF1A*(392-403)	Control	ND				627.8506	627.8477	52.2-52.3	8.67E+06	9.5
		PHD1	18.30	51.82	A-028	P402: 73.62		627.8496	51.7-52.1	6.43E+07	92.1
		PHD3	ND					627.8491	51.4-51.5	4.44E+06	14.8

B-03: HIF Control C

Alt Substrate: CENPN & FLNA

HIF1A*$_{(548\text{-}575)}$: NPFSTQDTDLDLEALAPYIPADDDFQLR

A

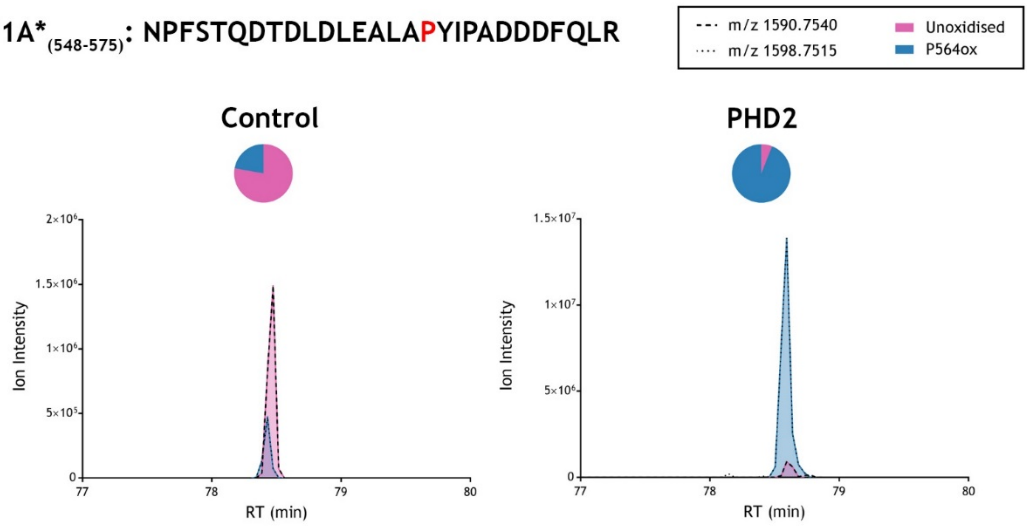

			Assignment					Quantitation			
	Species	Condition	-10 lgP	RT	MSMS	AScore	Expected MH^{+2}	MH^{+2}	RT Win.	Area	RA (%)
0	HIF1A*(548-575)	Control	45.13	78.44	A-029	N/A	1590.7540	1590.7552	78.3-78.5	6.3E+06	77.8
		PHD2	53.17	78.61	A-030	N/A		1590.7517	78.5-78.7	4.4E+06	6.0
16 Da	P564ox: HIF1A*(548-575)	Control	ND				1598.7515	1598.7479	78.3-78.6	1.8E+06	22.2
		PHD2	54.65	78.56	A-031	P564: 0		1598.7507	78.5-78.8	6.9E+07	94.0

HIF1A*$_{(392\text{-}403)}$: KEPDALTLLAPA

m/z 619.8532	Unoxidised
m/z 627.8506	P402ox

B

Peptide Not Detected

B-04: **HIF Control D**

Alt Substrate: ACTB, ADRB2, ATF4 & PKM

HIF1A*$_{(548-575)}$: NPFSTQDTDLDLEALAPYIPADDDFQLR

A

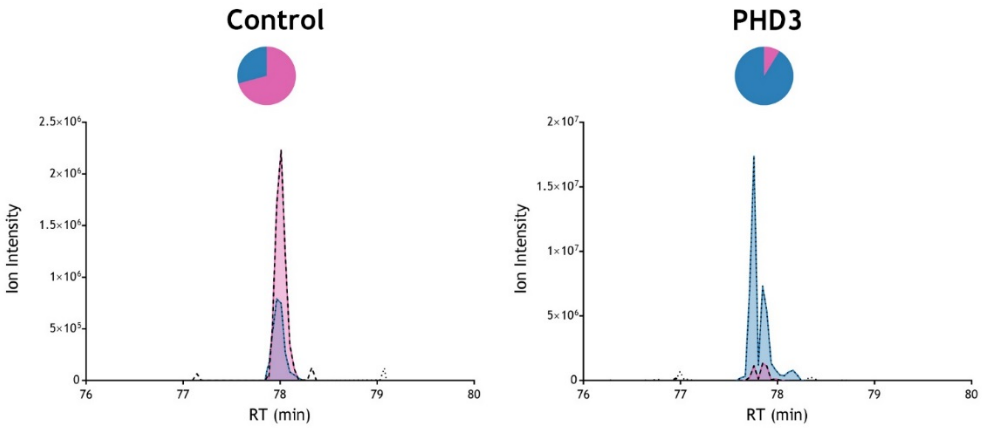

	Species	Condition	Assignment -10 lgP	MSMS	AScore	Expected MH^{+2}	Quantitation MH^{+2}	RT Win.	Area	RA (%)
0	HIF1A*(548-575)	Control	53.33	A-032	N/A	1590.7540	1590.7554	77.9-78.2	1.6E+07	70.8
		PHD3	56.70	A-033	N/A		1590.7519	77.6-78.1	1.1E+07	8.8
16 Da	P564ox: HIF1A*(548-575)	Control	32.77	A-034	P564: 0	1598.7515	1598.7524	77.7-78.4	6.79E+06	29.2
		PHD3	59.97	A-035	P564: 0		1598.7494	77.6-78.3	1.17E+08	91.2

HIF1A*$_{(392-403)}$: KEPDALTLLAPA

B

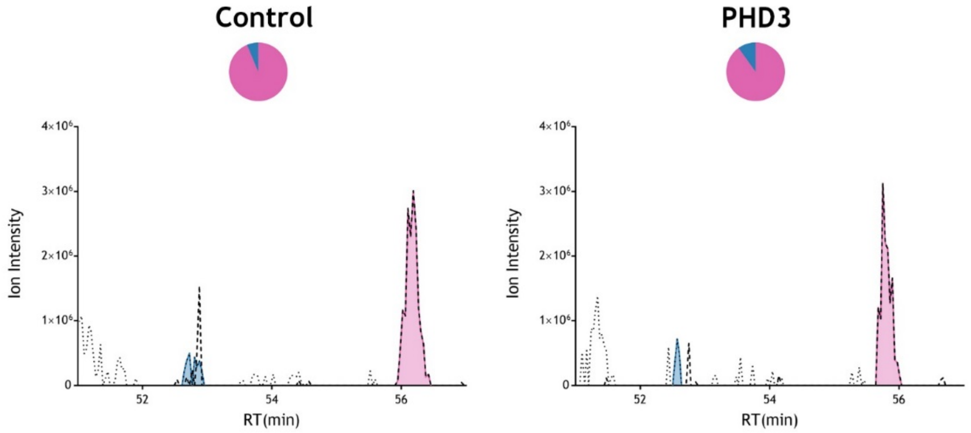

	Species	Condition	Assignment -10 lgP	MSMS	AScore	Expected MH^{+2}	Quantitation MH^{+2}	Peak Width	Area	RA (%)
0	HIF1A*(392-403)	Control	15.34	A-036	N/A	619.8532	619.8538	55.9-56.4	3.9E+07	93.7
		PHD3	ND				619.8531	55.6-56.0	3.0E+07	90.1
16 Da	P402ox: HIF1A*(392-403)	Control	ND			627.8506	627.8503	52.6-52.9	2.7E+06	6.3
		PHD3	ND				627.8480	52.5-52.6	3.3E+06	9.9

B-05: HIF Control E

Alt Substrate: SPRY2 & TELO2

HIF1A*$_{(548-575)}$: NPFSTQDTDLDLEALAPYIPADDDFQLR

--- m/z 1590.7540 | Unoxidised
···· m/z 1598.7515 | P564ox

A

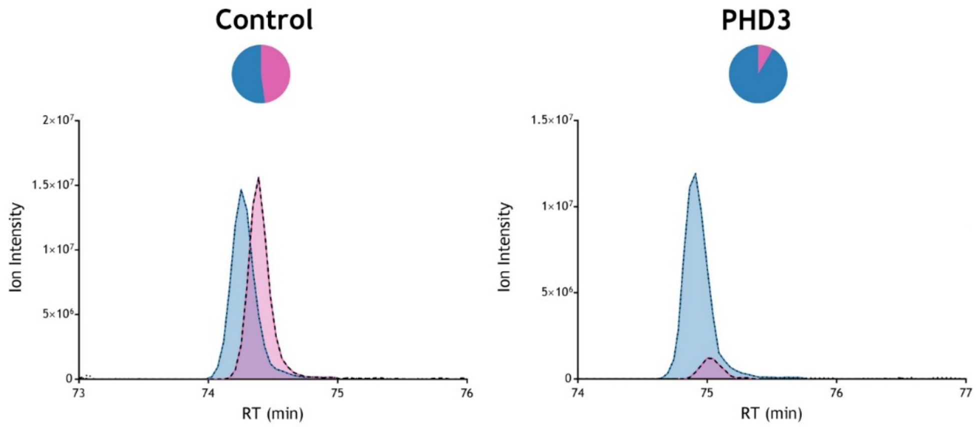

			Assignment					Quantitation			
	Species	Condition	-10 lgP	RT	MSMS	AScore	Expected MH^{+2}	MH^{+2}	RT Win.	Area	RA (%)
0	HIF1A*(548-575)	Control	59.23	74.35	A-037	N/A	1590.7540	1590.7556	74.1-75.0	1.8E+08	47.8
		PHD3	40.58	75.05	A-038	N/A		1590.7560	74.8-75.4	1.5E+07	8.4
16 Da	P564ox: HIF1A*(548-575)	Control	56.99	74.30	A-039	P564: 14.02	1598.7515	1598.7547	74.0-75.0	1.9E+08	52.2
		PHD3	58.94	74.87	A-040	P564: 0		1598.7547	74.7-75.8	1.6E+08	91.6

HIF1A*$_{(392-403)}$: KEPDALTLLAPA

--- m/z 619.8532 | Unoxidised
···· m/z 627.8506 | P402ox

B

Peptide Not Detected

B-06: HIF Control F

Alt Substrate: CEP192

HIF1A*$_{(548\text{-}575)}$: NPFSTQDTDLDLEALAPYIPADDDFQLR

A

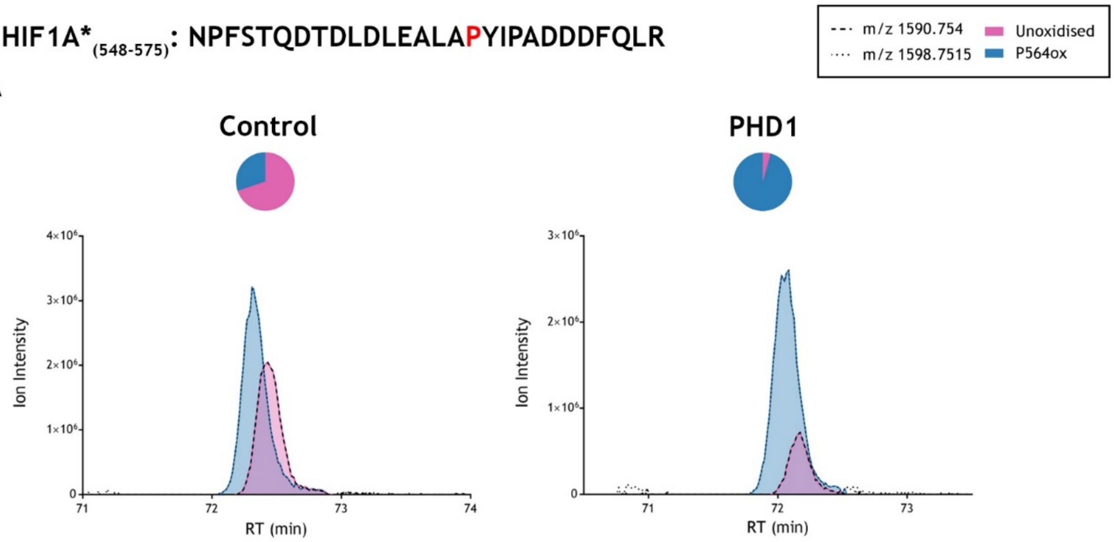

	Species	Condition	Assignment: -10 lgP	Assignment: RT	Assignment: MSMS	Assignment: AScore	Expected MH^{+2}	Quantitation: MH^{+2}	Quantitation: RT Win.	Quantitation: Area	Quantitation: RA (%)
0	HIF1A*(548-575)	Control	43.91	72.41	A-041	N/A	1590.7540	1590.7566	72.2-72.9	2.8E+07	39.9
		PHD1	16.76	72.16	A-042	N/A		1590.7567	72.0-72.5	8.3E+06	18.4
16 Da	P564ox: HIF1A*(548-575)	Control	52.82	72.38	A-043	P564: 0	1598.7515	1598.7535	72.1-72.9	4.2E+07	60.1
		PHD1	49.04	72.11	A-044	P564: 0		1598.7538	71.8-72.5	3.7E+07	81.6

HIF1A*$_{(392\text{-}403)}$: KEPDALTLLAPA

B

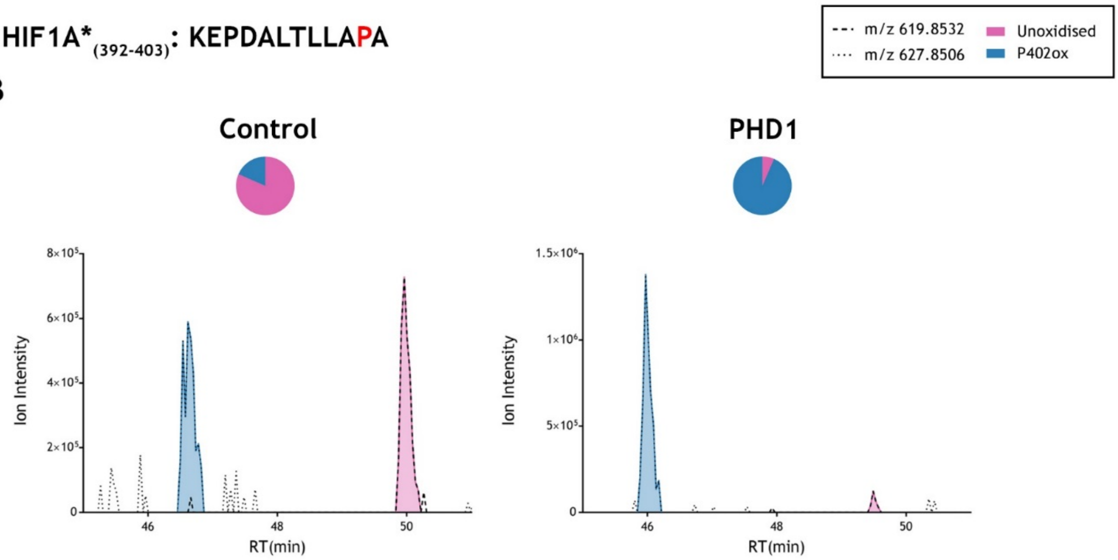

	Species	Condition	Assignment: -10 lgP	Assignment: RT	Assignment: MSMS	Assignment: AScore	Expected MH^{+2}	Quantitation: MH^{+2}	Quantitation: RT Win.	Quantitation: Area	Quantitation: RA (%)
0	HIF1A*(392-403)	Control	ND				619.8532	619.8527	49.9-50.2	7.3E+06	49.4
		PHD1	ND					619.8521	49.4-49.6	6.2E+05	5.0
16 Da	P402ox: HIF1A*(392-403)	Control	ND				627.8506	627.8506	46.5-46.8	7.5E+06	50.6
		PHD1	15.48	46.01	A-045	P402: 73.62		627.8510	45.9-46.2	1.2E+07	95.0

B-07: **HIF Control G**

Alt Substrate: FOXO3 & IKBKB

HIF1A*$_{(548-575)}$: NPFSTQDTDLDLEALAPYIPADDDFQLR

A

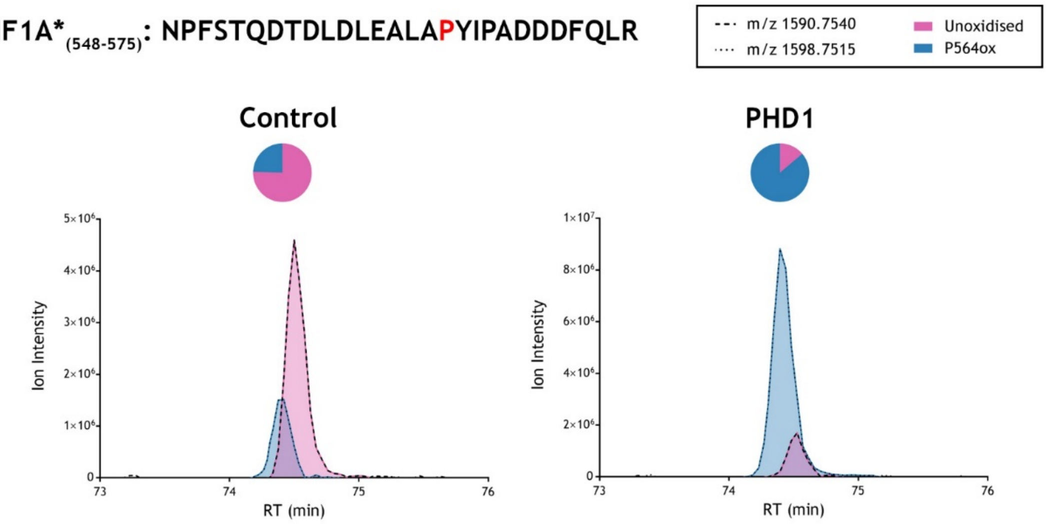

			Assignment					Quantitation			
	Species	Condition	-10 lgP	RT	MSMS	AScore	Expected MH^{+2}	MH^{+2}	RT Win.	Area	RA (%)
0	HIF1A*(548-575)	Control	45.06	74.54	A-048	N/A	1590.7540	1590.7502	74.3-75.0	5.0E+07	75.5
		PHD1	36.82	74.53	A-049	N/A		1590.7526	74.3-74.8	1.6E+07	13.8
16 Da	P564ox: HIF1A*(548-575)	Control	26.75	74.42	A-050	P564: 0	1598.7515	1598.7496	74.2-74.8	1.6E+07	24.5
		PHD1	53.54	74.40	A-051	P564: 0		1598.7502	74.1-75.-1	1.0E+08	86.2

HIF1A*$_{(392-403)}$: KEPDALTLLAPA

B

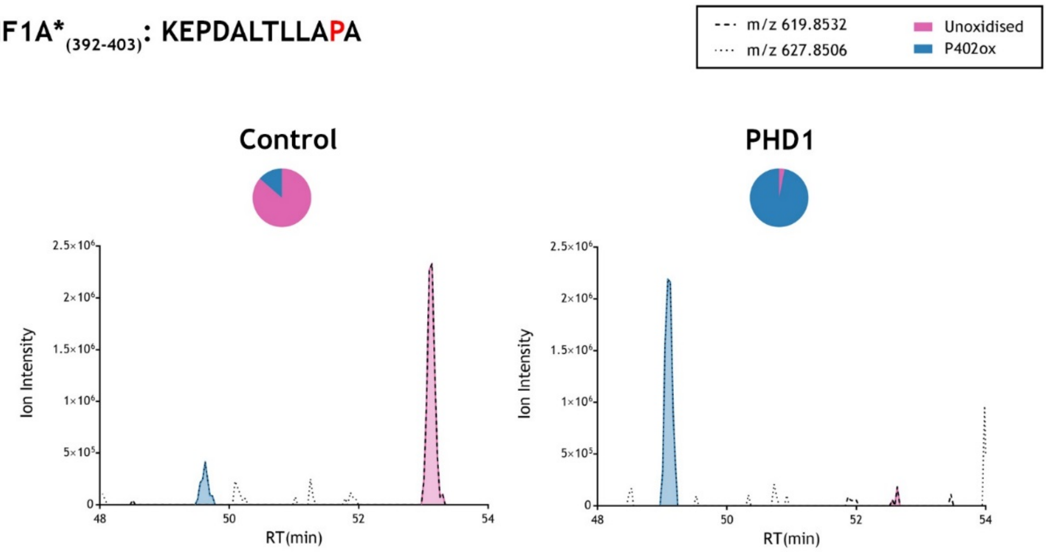

			Assignment					Quantitation			
	Species	Condition	-10 lgP	RT	MSMS	AScore	Expected MH^{+2}	MH^{+2}	RT Win.	Area	RA (%)
0	HIF1A*(392-403)	Control	14.79	53.09	A-052	N/A	619.8532	619.8532	53.0-53.3	2.0E+07	86.4
		PHD1	ND					619.8509	52.5-52.6	5.6E+05	3.0
16 Da	P402ox: HIF1A*(392-403)	Control	ND				627.8506	627.8503	49.5-49.7	3.1E+06	13.6
		PHD1	19.51	49.08	A-053	P402: 73.62		627.8515	49.0-49.2	1.8E+07	97.0

B-08: **HIF Control H**

Alt Substrate: EEF2K, PDE4D & TRPA1

HIF1A*$_{(548\text{-}575)}$: NPFSTQDTDLDLEALAPYIPADDDFQLR

A

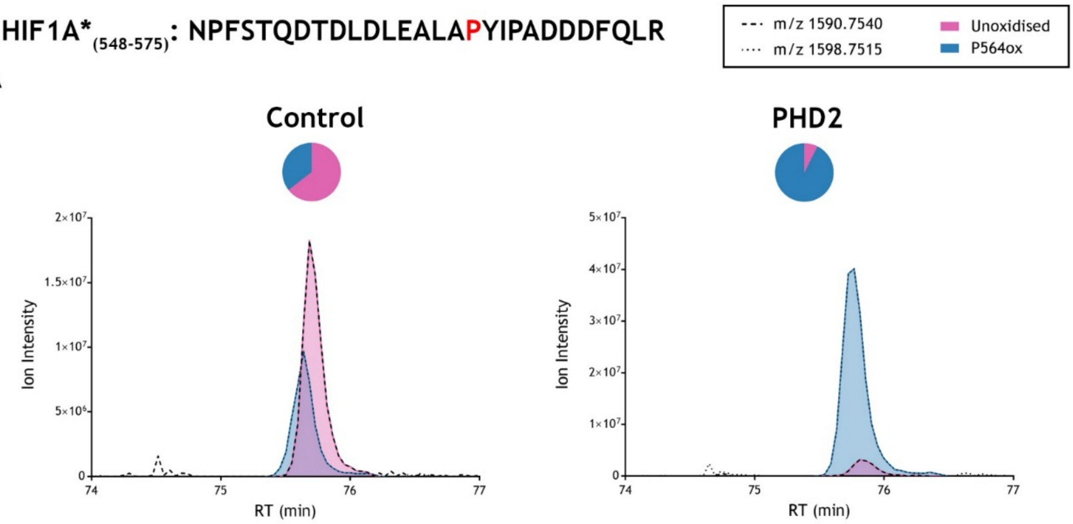

	Species	Condition	Assignment: -10 lgP	RT	MSMS	AScore	Expected MH^{+2}	Quantitation: MH^{+2}	RT Win.	Area	RA (%)
0	HIF1A*(548-575)	Control	62.03	75.91	A-054	N/A	1590.7540	1590.7564	75.5-76.2	2.0E+08	64.5
		PHD2	53.10	75.82	A-055	N/A		1590.7574	75.6-76.2	4.1E+07	7.4
16 Da	P564ox: HIF1A*(548-575)	Control	71.54	75.73	A-056	P564: 8.69	1598.7515	1598.7527	75.4-76.2	1.1E+08	35.5
		PHD2	60.86	75.68	A-057	P564: 17.01		1598.7544	75.5-76.5	5.2E+08	92.6

HIF1A*$_{(392\text{-}411)}$: KEPDALTLLAPAAGDTIISL

B

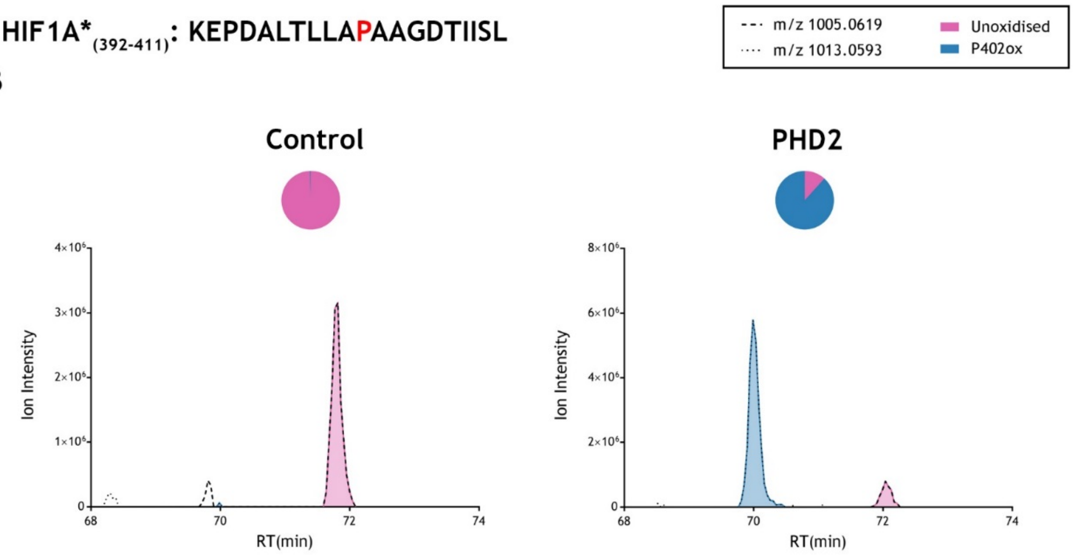

	Species	Condition	Assignment: -10 lgP	RT	MSMS	AScore	Expected MH^{+2}	Quantitation: MH^{+2}	RT Win.	Area	RA (%)
0	HIF1A*(392-411)	Control	35.45	71.70	A-058	N/A	1005.0619	1005.0638	71.6-72.0	3.5E+07	99.5
		PHD2	23.08	71.97	A-059	N/A		1005.0605	71.9-72.2	8.8E+06	11.7
16 Da	P402ox: HIF1A*(392-411)	Control	ND				1013.0593	1013.0688	69.9-70.0	1.7E+05	0.5
		PHD2	38.13	69.92	A-060	P402: 28.70		1013.0597	69.8-70.4	6.6E+07	88.3

B-09: HIF Control I

Alt Substrate: PPP2R2A

HIF1A*$_{(548-575)}$: NPFSTQDTDLDLEALAPYIPADDDFQLR

A

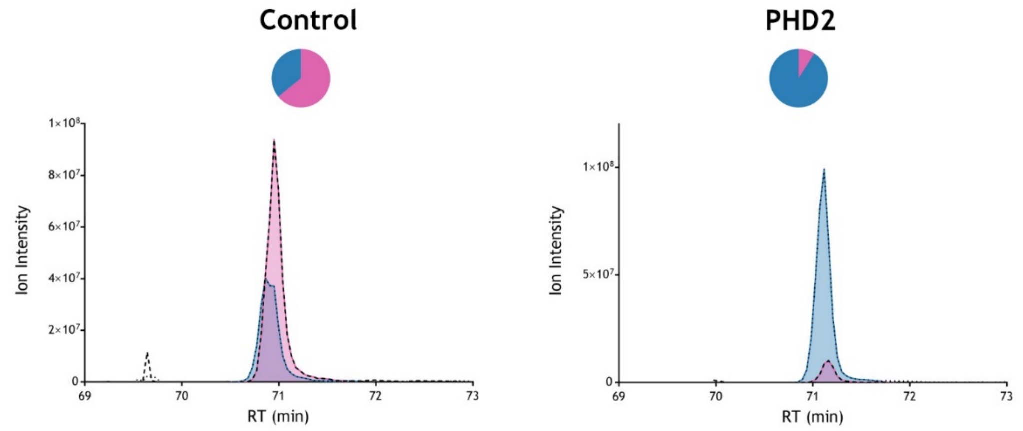

	Species	Condition	Assignment: -10 lgP	RT	MSMS	AScore	Expected MH^{+2}	Quantitation: MH^{+2}	RT Win.	Area	RA (%)
0	HIF1A*(548-575)	Control	67.45	70.87	A-061	N/A	1590.7540	1590.7586	70.7-71.6	1.0E+09	64.1
		PHD2	57.12	71.17	A-062	N/A		1590.7556	70.9-71.8	1.0E+08	9.0
16 Da	P564ox: HIF1A*(548-575)	Control	68.21	70.82	A-063	P564: 17.01	1598.7515	1598.7523	70.6-71.8	5.8E+08	35.9
		PHD2	66.95	71.17	A-064	P564: 17.01		1598.7528	70.9-71.7	1.0E+09	91.0

HIF1A*$_{(392-411)}$: KEPDALTLLAPAAGDTIISL

B

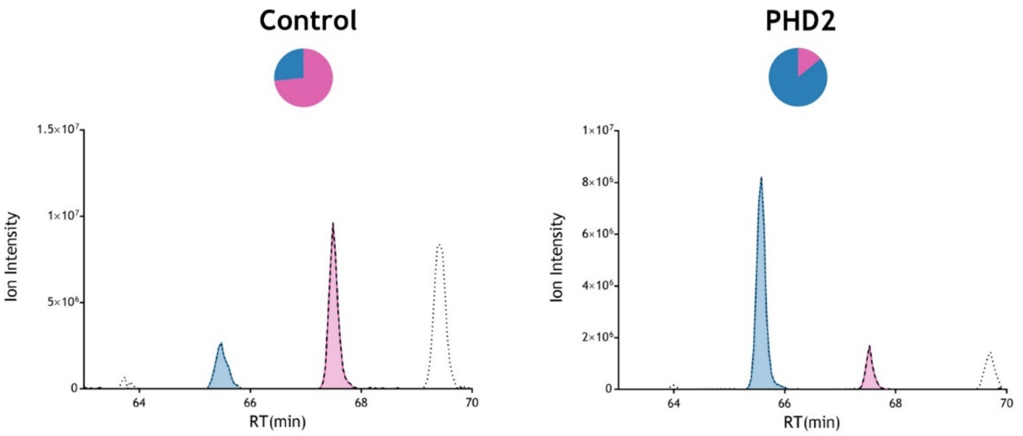

	Species	Condition	Assignment: -10 lgP	RT	MSMS	AScore	Expected MH^{+2}	Quantitation: MH^{+2}	RT Win.	Area	RA (%)
0	HIF1A*(392-411)	Control	26.25	67.50	A-065	N/A	1005.0619	1005.0630	67.2-67.9	1.1E+08	73.4
		PHD2	34.34	67.58	A-066	N/A		1005.0624	67.4-67.9	1.5E+07	14.1
16 Da	P402ox: HIF1A*(392-411)	Control	28.6	65.36	A-067	P402: 25.70	1013.0593	1013.0604	65.2-65.8	3.9E+07	26.6
		PHD2	33.50	65.58	A-068	P402: 42.68		1013.0606	65.3-66.1	9.4E+07	85.9

B-10: **HIF Control J**

Alt Substrate: AKT1

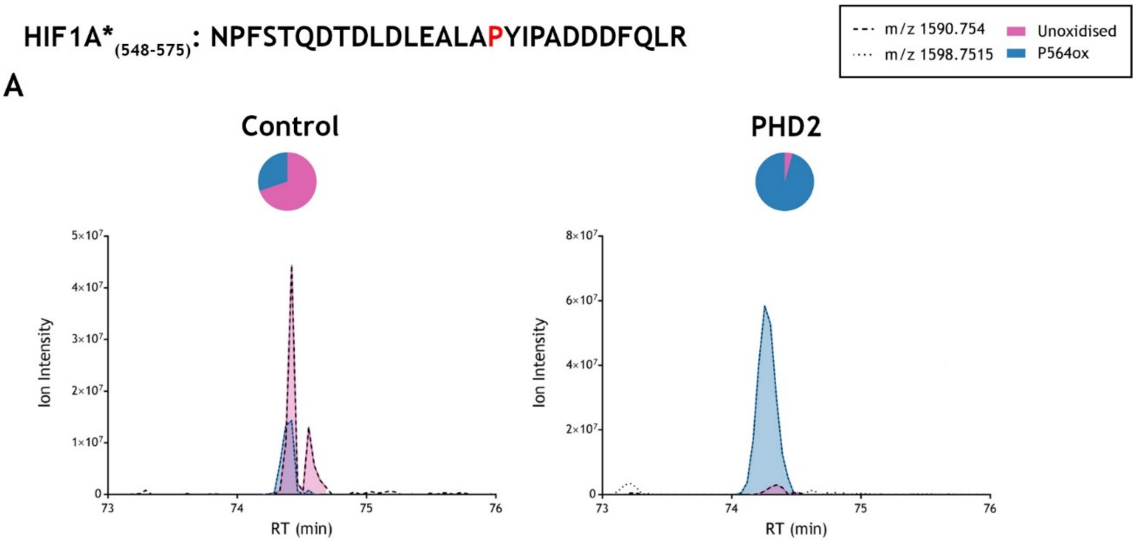

	Species	Condition	-10 lgP	RT	MSMS	AScore	Expected MH^{+2}	MH^{+2}	RT Win.	Area	RA (%)
			Assignment					Quantitation			
0	HIF1A*(548-575)	Control	62.70	74.60	A-069	N/A	1590.7540	1590.7472	74.3-74.7	2.2E+08	69.8
		PHD2	47.88	74.26	A-070	N/A		1590.7518	74.1-74.6	2.7E+07	4.3
16 Da	P564ox: HIF1A*(548-575)	Control	71.20	74.43	A-071	P564: 12.28	1598.7515	1598.7491	74.2-74.5	9.4E+07	30.2
		PHD2	56.74	74.40	A-072	P564: 17.01		1598.7476	74.0-74.5	6.0E+08	95.7

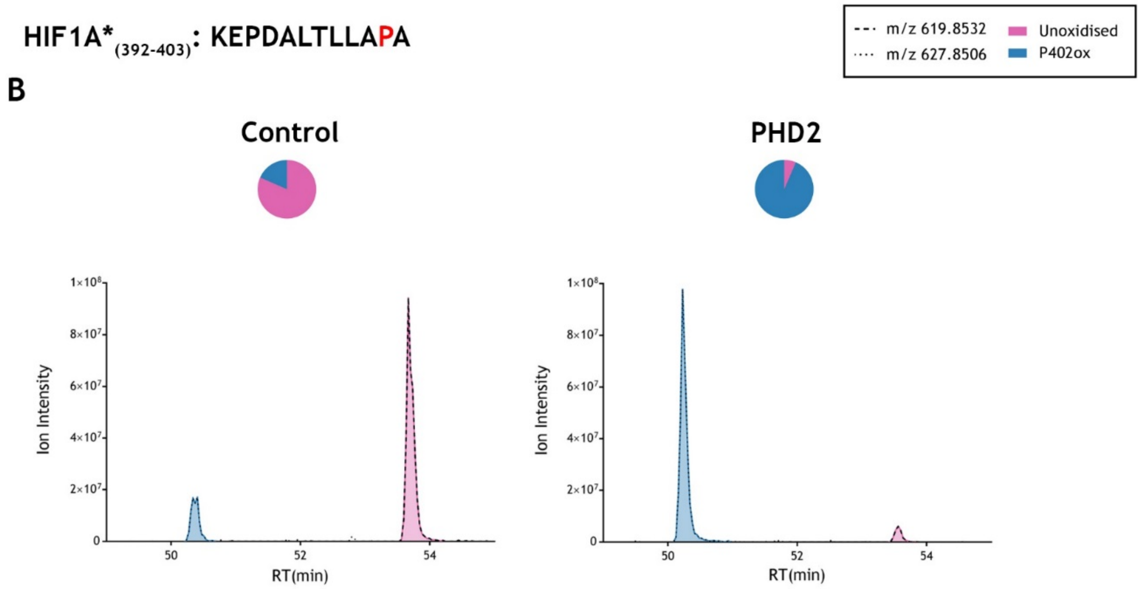

	Species	Condition	-10 lgP	RT	MSMS	AScore	Expected MH^{+2}	MH^{+2}	RT Win.	Area	RA (%)
			Assignment					Quantitation			
0	HIF1A*(392-403)	Control	20.28	53.74	A-073	N/A	619.8532	619.8528	53.6-54.2	6.9E+08	81.6
		PHD2	11.93	53.33	A-074	N/A		619.8531	53.4-53.9	4.6E+07	6.5
16 Da	P402ox: HIF1A*(392-403)	Control	18.49	50.38	A-075	P402: 64.33	627.8506	627.8496	50.2-50.7	1.6E+08	18.4
		PHD2	16.28	50.31	A-076	P402: 89.40		627.8505	50.1-50.9	6.7E+08	93.5

B-11: HIF Control K

Alt Substrate: ACACB

HIF1A*$_{(548-575)}$: NPFSTQDTDLDLEALAPYIPADDDFQLR

A

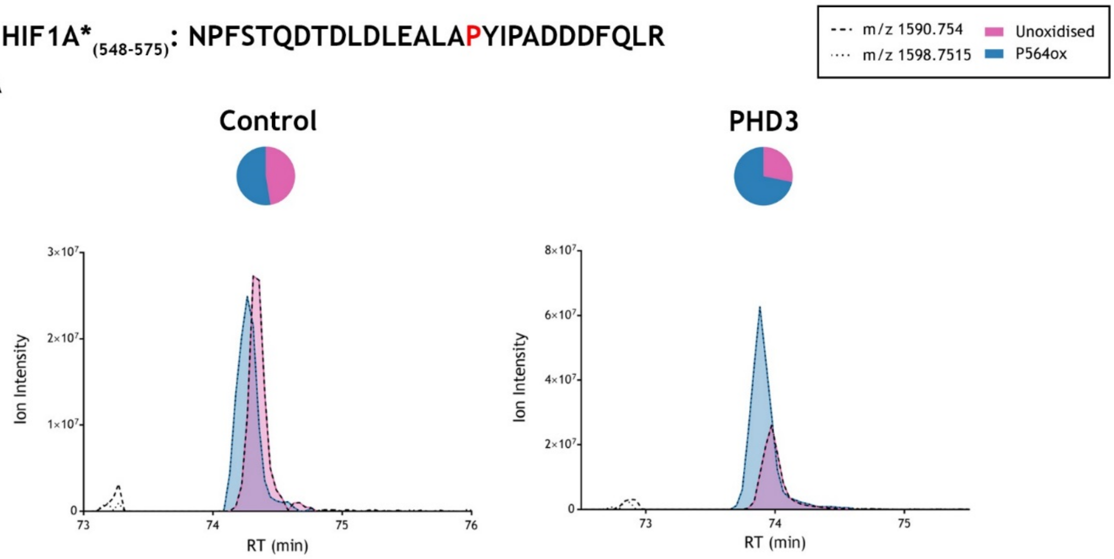

			Assignment					Quantitation			
	Species	Condition	-10 lgP	RT	MSMS	AScore	Expected MH^{+2}	MH^{+2}	RT Win.	Area	RA (%)
0	HIF1A*(548-575)	Control	64.29	74.36	A-077	N/A	1590.7540	1590.7546	74.1-74.8	2.5E+08	47.4
		PHD3	66.53	73.98	A-078	N/A		1590.7556	74.0-74.6	2.6E+08	28.2
16 Da	P564ox: HIF1A*(548-575)	Control	60.72	74.23	A-079	P564: 12.28	1598.7515	1598.7521	74.1-74.8	2.8E+08	52.6
		PHD3	63.04	73.84	A-080	P564: 14.02		1598.7531	73.7-74.7	6.7E+08	71.8

HIF1A*$_{(392-403)}$: KEPDALTLLAPA

B

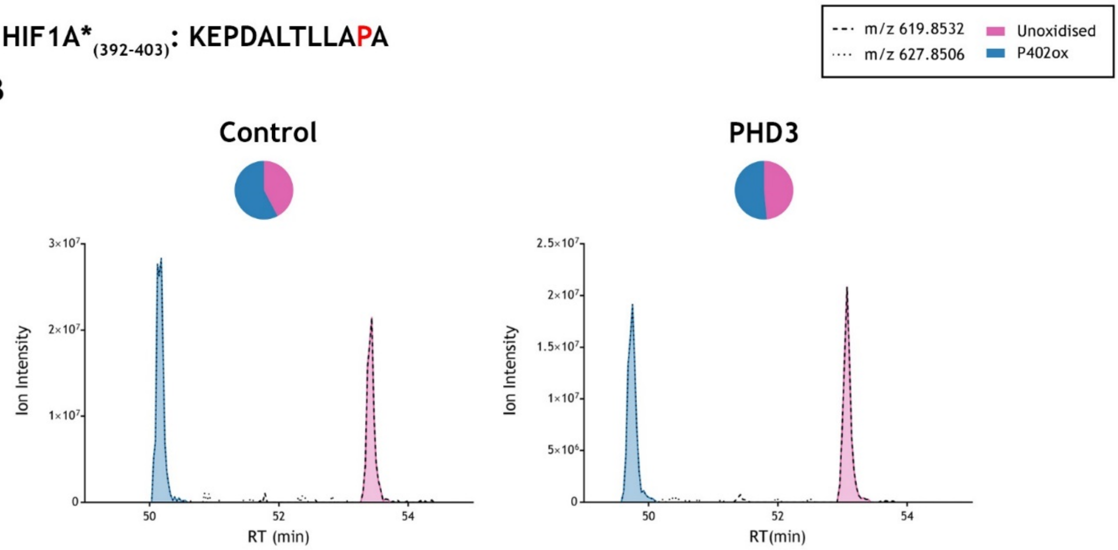

			Assignment					Quantitation			
	Species	Condition	-10 lgP	RT	MSMS	AScore	Expected MH^{+2}	MH^{+2}	RT Win.	Area	RA (%)
0	HIF1A*(392-403)	Control	14.39	53.88	A-081	N/A	619.8532	619.8527	53.3-53.7	1.6E+08	42.3
		PHD3	16.88	53.01	A-082	N/A		619.8536	53.0-53.4	1.6E+08	48.5
16 Da	P402ox: HIF1A*(392-403)	Control	17.88	50.11	A-083	P402: 89.02	627.8506	627.8496	50.1-50.5	2.2E+08	57.7
		PHD3	15.83	49.67	A-084	P402: 89.02		627.8503	49.6-50.1	1.7E+08	51.5

B-12: HIF Control L

Alt Substrate: THRA

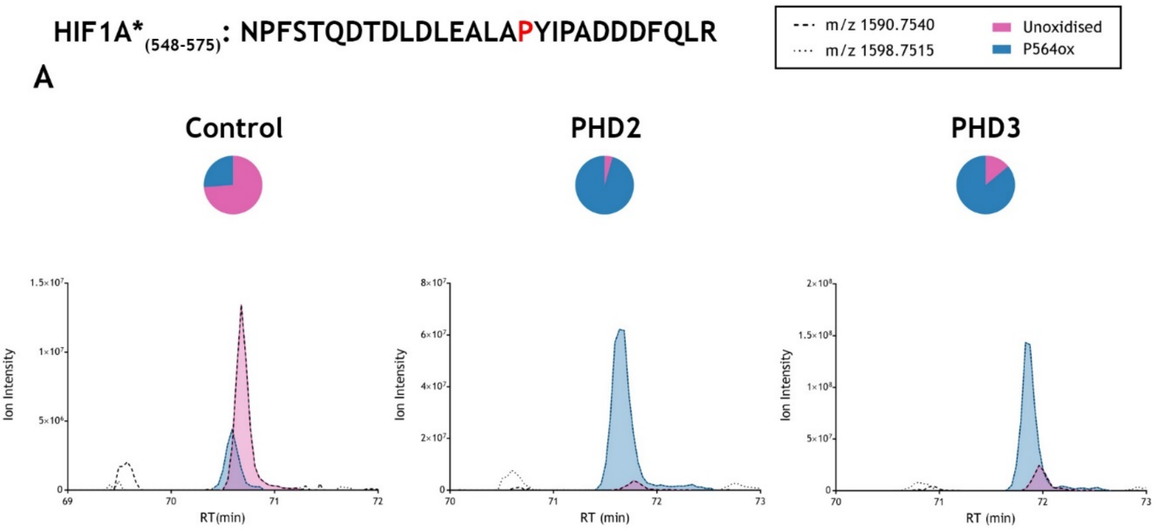

			Assignment					Quantitation			
	Species	Condition	-10 lgP	RT	MSMS	AScore	Expected MH^{+2}	MH^{+2}	RT Win.	Area	RA (%)
0	HIF1A*(548-575)	Control	56.93	70.73	A-092	N/A	1590.7540	1590.7555	70.5-71.3	1.2E+08	73.9
		PHD2	48.29	71.82	A-093	N/A		1590.7540	71.6-72.4	4.0E+07	4.4
		PHD3	62.31	72.01	A-094	N/A		1590.7533	71.8-72.5	2.4E+08	14.0
16 Da	P564ox: HIF1A*(548-575)	Control	55.98	70.64	A-095	P564: 0	1598.7515	1598.7516	70.4-70.9	4.2E+07	26.1
		PHD2	56.49	71.47	A-096	P564: 17.01		1598.7519	71.4-72.6	8.6E+08	95.6
		PHD3	66.58	71.84	A-097	P564: 14.02		1598.7507	71.7-72.7	1.5E+09	86.0

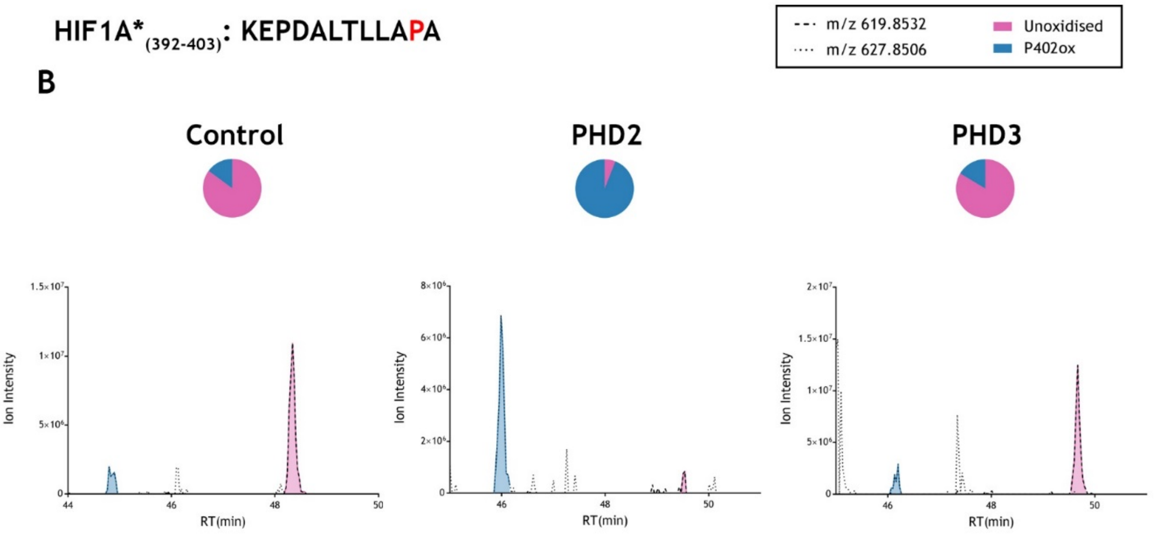

			Assignment					Quantitation			
	Species	Condition	-10 lgP	RT	MSMS	AScore	Expected MH^{+2}	MH^{+2}	RT Win.	Area	RA (%)
0	HIF1A*(392-403)	Control	16.06	48.29	A-098	N/A	619.8532	619.8533	48.2-48.6	8.5E+07	85.1
		PHD2	ND					619.8543	49.5	3.2E+06	6.0
		PHD3	12.43	49.68	A-099	N/A		619.8521	49.5-49.9	7.8E+07	83.6
16 Da	P402ox: HIF1A*(392-403)	Control	ND				627.8506	627.8512	44.7-45.0	1.5E+07	14.9
		PHD2	22.48	45.97	A-100	P402: 73.62		627.8510	45.9-46.2	4.9E+07	94.0
		PHD3	ND					627.8490	46.0-46.3	1.5E+07	16.4

B-13: **HIF Control M**

Alt Substrate: TP53

HIF1A*$_{(548-575)}$: NPFSTQDTDLDLEALAPYIPADDDFQLR

--- m/z 1590.754 ···· m/z 1598.7515 | Unoxidised | P564ox

A

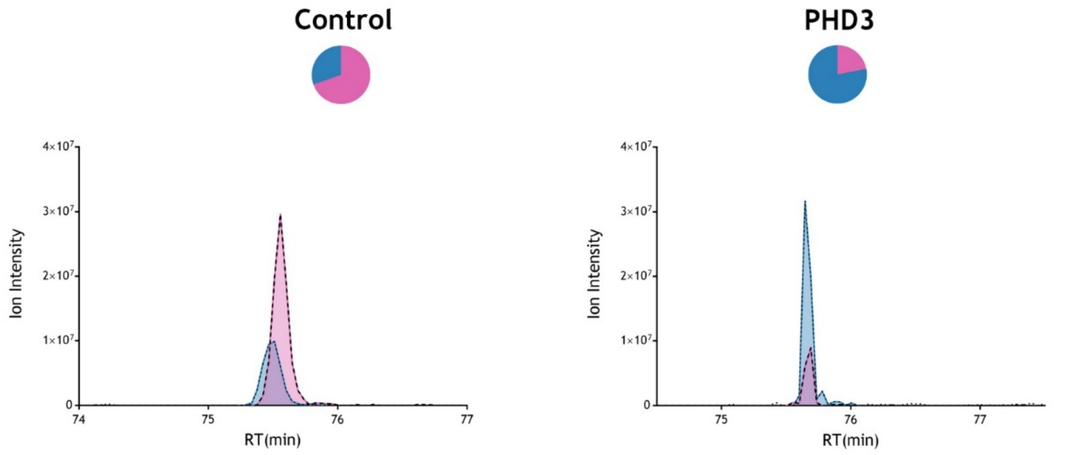

	Species	Condition	Assignment: -10 lgP	RT	MSMS	AScore	Expected MH^{+2}	Quantitation: MH^{+2}	RT Win.	Area	RA (%)
0	HIF1A*(548-575)	Control	84.10	75.56	A-085	N/A	1590.7540	1590.7595	75.4-76.0	2.4E+08	69.6
		PHD3	74.58	75.74	A-086	N/A		1590.7613	75.5-75.7	4.4E+07	21.9
16 Da	P564ox: HIF1A*(548-575)	Control	77.19	75.47	A-087	P564: 14.02	1598.7515	1598.7571	75.3-75.9	1.0E+08	30.4
		PHD3	64.14	75.88	A-088	P564: 12.28		1598.7599	75.6-76.1	1.6E+08	78.1

HIF1A*$_{(392-411)}$: KEPDALTLLAPAAGDTIISL

--- m/z 1005.0619 ···· m/z 1013.0593 | Unoxidised | P402ox

B

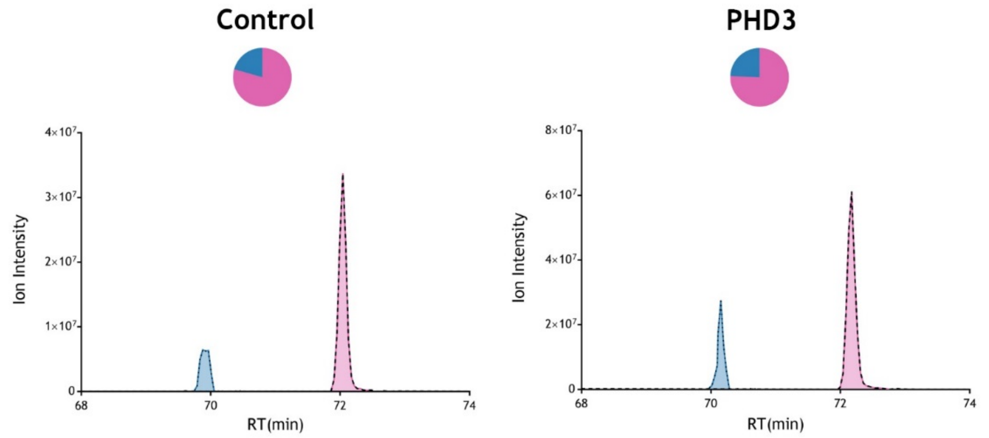

	Species	Condition	Assignment: -10 lgP	RT	MSMS	AScore	Expected MH^{+2}	Quantitation: MH^{+2}	RT Win.	Area	RA (%)
0	HIF1A*(392-411)	Control	42.65	72.05	A-089	N/A	1005.0619	1005.0620	71.9-72.5	2.8E+08	79.4
		PHD3	43.79	72.18	A-090	N/A		1005.0635	72.0-72.7	5.5E+08	75.7
16 Da	P402ox: HIF1A*(392-411)	Control	39.77	69.97	A-046	P402: 42.68	1013.0593	1013.0584	69.7-70.0	7.3E+07	20.6
		PHD3	43.41	70.16	A-047	P402: 42.68		1013.0606	69.9-70.2	1.8E+08	24.3
