# Supplementary material for: Lack of activity of recombinant HIF prolyl hydroxylases (PHDs) on reported non-HIF substrates
Source: eLife. 2019 Sep 10;8:e46490. doi: 10.7554/eLife.46490 (PMC6739866; doi:10.7554/eLife.46490)
Supplement: Supplementary file 3. [file elife-46490-supp3.pdf]

Supplementary file 3

(i)

Substrate	PHD Isoform	Peptide Resid.	MS Run: Control	MS Run: Enzyme
HIF-1α*	PHD1	392-411	YMT115	YMT116
		548-575		
	PHD2	392-411		YMT117
		548-575		
	PHD3	392-411		YMT118
		548-575		
HIF-2α	PHD1	393-429	YMT119	YMT120
		513-550		
	PHD2	393-429		YMT121
		513-550		
	PHD3	393-429		YMT122
		513-550		
HIF-3α	PHD1	486-496	YMT123	YMT124
	PHD2			YMT125
	PHD3			YMT126

(ii)

Substrate	PHD Isoform	Peptide Resid.	Peptide Standard	Alt. Substrate Control	Alt. Substrate Enzyme	HIF-1α Control Control	HIF-1α Control Enzyme
ACACB	PHD3	341-361	PEP17	YMT86	YMT88	YMT86	YMT88
		436-454					
ACTB	PHD3	292-312	PEP20	YMT41	YMT42	YMT41	YMT42
		316-326					
ADRB2	PHD3	376-404	PEP11	YMT65	YMT66	YMT41	YMT42
AKT1	PHD2	122-140	PEP17	YMT83	YMT84	YMT83	YMT84
		308-328					
		421-436					
ATF4	PHD3	NT	-	YMT45	YMT46	YMT41	YMT42
CENPN	PHD2	308-329	PEP14	YMT39	YMT40	YMT51	YMT52
CEP192	PHD1	2306-2317	PEP11	YMT61	YMT62	YMT61	YMT62
EEF2K	PHD2	94-111	PEP17	YMT69	YMT70	YMT69	YMT70
EPOR	PHD3	426-453	PEP14	YMT63	YMT64	YMT25	YMT28
FLNA	PHD2	2311-2333	PEP20	YMT65	YMT66	YMT51	YMT52
FOXO3	PHD1	420-444	PEP14	YMT59	YMT60	YMT59	YMT60
IKBKB	PHD1	172-198	PEP27	YMT59	YMT60	YMT59	YMT60
MAPK6	PHD3	20-45	PEP17	YMT31	YMT32	YMT25	YMT28
NDRG3	PHD2	287-301	PEP11	YMT23	YMT24	YMT57	YMT58
PDE4D	PHD2	370-383	PEP11	YMT69	YMT70	YMT69	YMT70
		411-431	PEP17				
PKM	PHD3	401-422	PEP20	YMT55	YMT56	YMT41	YMT42
PPP2R2A	PHD2	310-330	PEP27	YMT79	YMT80	YMT82	PEP14
SPRY2	PHD1	5-19	PEP11	YMT29	YMT30	YMT25	YMT26
		131-151	PEP17				
		156-168					
	PHD3	5-19	PEP11	YMT47	YMT48	YMT65	YMT66
		131-151	PEP17				
		156-168					
TELO2	PHD3	363-377	PEP11	YMT47	YMT48	YMT65	YMT66
THRA	PHD2	153-176	PEP21	YMT93	YMT94	YMT93	YMT94
	PHD3			YMT93	YMT95	YMT93	YMT95
TP53	PHD1	140-156	PEP27	YMT31	YMT32	YMT25	YMT26
	PHD3	358-370	PEP13	YMT113	YMT114	YMT109	YMT110
TRPA1	PHD2	391-403	PEP11	YMT71	YMT72	YMT69	YMT70
