# Supplementary material for: Lack of activity of recombinant HIF prolyl hydroxylases (PHDs) on reported non-HIF substrates
Source: eLife. 2019 Sep 10;8:e46490. doi: 10.7554/eLife.46490 (PMC6739866; doi:10.7554/eLife.46490)
Supplement: Supplementary file 4. [file elife-46490-supp4.pdf]

# Supplementary file 4

| | $^{3}$H Hyp assay | IVTT MSMS Hydroxylation assay | | Peptide MS Hydroxylation assay | |
|---|---|---|---|---|---|
| **Substrate** | **Replicate #** | **Target Residue** | **Replicate #** | **Peptide Name** | **Replicate #** |
| ACACB | 2 | P343 | 2 | **NT** | **-** |
| | | P450 | 2 | ACACB/438-462 | 1 |
| | | | | ACACB/445-462 | 1 |
| ACTB | 2 | P307 | 2 | ACTB/295-319 | 3 |
| | | P322 | 2 | ACTB/310-334 | 3 |
| ADRB2 | 1 | P382 | 1 | ADRB2/370-394 | 3 |
| | | P395 | 1 | ADRB2/383-407 | 3 |
| AKT1 | | P125 | 2 | AKT1/113-137 | 3 |
| | | | | AKT1/115-134 | 3 |
| | | P313, P318 | 2 | AKT1/301-325 | 3 |
| | | | | AKT1/307-328 | 3 |
| | | | | AKT1/311-330 | 3 |
| | | P423 | 2 | NT | - |
| ATF4 | 2 | P156, P162, P164, P167 | 1 | ATF4/144-168 | 3 |
| | | P174 | 1 | ATF4/157-182 | 3 |
| CENPN/iso1 | 2 | P311 | 1 | CENPN/299-323_iso1 | 1 |
| | | | | CENPN/299-323_iso3 | 1 |
| | | | | CENPN/308-317_iso3 | 1 |
| CEP192 | 2 | P2313 | 1 | CEP192/2303-2326 | 3 |
| | | | | CEP192/2306-2317 | 3 |
| EEF2K | 2 | P98 | 2 | EEF2K/86-110 | 3 |
| EPOR | 2 | P443, P450 | 2 | EPOR/434-458 | 3 |
| | | | | EPOR/441-455 | 3 |
| FLNA | 2 | P2317, P2324 | 1 | FLNA/P2310-2333 | 3 |
| FOXO3 | 2 | P426, P437 | 2 | FOXO3/414-438 | 3 |
| | | | | FOXO3/425-448 | 3 |
| IKBKB | 2 | P191 | 1 | IKBKB/180-204 | 3 |
| MAPK6 | 2 | P25 | 1 | MAPK6/13-37 | 1 |
| NDRG3 | 2 | P294 | 2 | NDRG3/282-306 | 3 |
| PDE4D | 2 | P19 | - | PDE4D/17-41 | 3 |
| | | P381 | 1 | PDE4D/370-394 | 3 |
| | | P419 | 1 | PDE4D/407-431 | 3 |
| PKM | 1 | P403, P408 | 1 | PKM/391-415 | 3 |
| | | | | PKM/396-422 | 3 |
| PPP2R2A | 2 | P319 | 1 | PPP2R2A/308-332 | 1 |
| | | | | PPP2R2A/311-329 | 1 |
| POLR2A | NT | P1465 | NT | POLR2A/1456-1476 | 3 |
| SPRY2 | 2 | P18, P144, P160 | 1 | SPRY2/6-30 | 3 |
| | | | | SPRY2/132-156 | 3 |
| | | | | SPRY2/148-172 | 3 |
| TELO2 | 1 | P374 | 1 | TELO2/362-386 | 3 |
| | | P419, P422 | - | TELO2/409-433 | 3 |
| THRA | 2 | P160, P162 | 2 | THRA/149-173 | 1 |
| TP53 | 2 | P142 | 1 | NT | - |
| | | P359 | 1 | TP53/347-371 | 1 |
| TRPA1 | 1 | P394 | 1 | TRPA1/382-406 | 3 |
| | | | | TRPA1/386-405 | 3 |
